# Tools for *Anopheles gambiae* Transgenesis

**DOI:** 10.1534/g3.115.016808

**Published:** 2015-04-13

**Authors:** Gloria Volohonsky, Olivier Terenzi, Julien Soichot, Daniel A. Naujoks, Tony Nolan, Nikolai Windbichler, Delphine Kapps, Andrea L. Smidler, Anaïs Vittu, Giulia Costa, Stefanie Steinert, Elena A. Levashina, Stéphanie A. Blandin, Eric Marois

**Affiliations:** * INSERM U963, CNRS UPR9022, Université de Strasbourg, Institut de Biologie Moléculaire et Cellulaire, 67084 Strasbourg, France; † Imperial College London, Division of Cell and Molecular Biology, Imperial College Road, London SW7 2AZ, United Kingdom; ‡ Department of Vector Biology, Max-Planck Institute for Infection Biology, Charitéplatz 1, 10117 Berlin, Germany

**Keywords:** phage ΦC31 *att*P docking sites, *cre* recombinase, transgenesis vectors, puromycin, codon usage, circumsporozoite protein

## Abstract

Transgenesis is an essential tool to investigate gene function and to introduce desired characters in laboratory organisms. Setting-up transgenesis in non-model organisms is challenging due to the diversity of biological life traits and due to knowledge gaps in genomic information. Some procedures will be broadly applicable to many organisms, and others have to be specifically developed for the target species. Transgenesis in disease vector mosquitoes has existed since the 2000s but has remained limited by the delicate biology of these insects. Here, we report a compilation of the transgenesis tools that we have designed for the malaria vector *Anopheles gambiae*, including new docking strains, convenient transgenesis plasmids, a puromycin resistance selection marker, mosquitoes expressing *cre* recombinase, and various reporter lines defining the activity of cloned promoters. This toolbox contributed to rendering transgenesis routine in this species and is now enabling the development of increasingly refined genetic manipulations such as targeted mutagenesis. Some of the reagents and procedures reported here are easily transferable to other nonmodel species, including other disease vector or agricultural pest insects.

Routine transgenesis in *Drosophila melanogaster* has led to a degree of understanding of the fruit fly biology that is unparalleled in any other complex organism (St. Johnston 2013). This scientific saga began with the use of transposon-based transgenic constructs, which have served as a foundation to develop increasingly sophisticated genetic engineering tools that were instrumental in unraveling *Drosophila* biology. The fruit fly is not only easy to rear but also very amenable to germline transformation by embryonic microinjection. Human disease vector mosquitoes, although belonging to the same insect order (*Diptera*), are more delicate to handle. Although dengue vector mosquitoes of the genus *Aedes*, and also the Indian malaria vector *Anopheles stephensi*, are relatively easily transformed (*e.g.,* James *et al.* 1999; Catteruccia *et al.* 2000; Kokoza *et al.* 2001; Nimmo *et al.* 2006; Anderson *et al.* 2010; Labbé *et al.* 2010; Fu *et al.* 2010; Dong *et al.* 2011; Kokoza and Raikhel 2011; Marinotti *et al.* 2013), the major African malaria vector *Anopheles gambiae* is notoriously difficult to manipulate genetically. However, developing efficient mosquito transgenic technologies will greatly facilitate the study of vector/pathogen interactions, as well as other aspects of mosquito biology relevant to vector competence such as olfaction, reproduction, and immunity. In addition, as recently illustrated by encouraging results of transgenic approaches in fighting *Aedes aegypti* (Alphey 2014), genetically modified *Anopheles* mosquitoes could also eventually become a tool in the fight against malaria (Wang and Jacobs-Lorena 2013).

In the past 8 years, we have strived to develop procedures and reagents to improve *A. gambiae* transgenesis. These new tools have enabled us to generate more than 50 distinct transgenic lines expressing various constructs from basic reporter genes to advanced targeted mutagenesis reagents, as illustrated by the first mutant *A. gambiae* lines generated by TALEN-mediated mutagenesis (Smidler *et al.* 2013). Here, we report our accumulated transgenesis toolbox and accompanying information to benefit the broader mosquito/insect research community and transgenesis facilities. Tools that we make available include four new *A. gambiae* docking lines for phage ΦC31 integrase-based transgenesis, a Cre recombinase-expressing line for *lox*P cassette excision, and six fluorescent reporter lines reflecting the expression pattern of tissue-specific promoters. To accompany the docking lines, we describe a kit of insect transgenesis plasmids allowing efficient multiple-insert cloning and harboring either of seven possible transgenesis reporter markers, including a novel puromycin resistance cassette for transgenic larva selection in antibiotic-containing water. Additionally, we report a computer program to evaluate the suitability of heterologous genes for expression in *A. gambiae* based on codon usage. Taken together, these technological advances streamline establishment of loss-of-function, gain-of-function, and mutant mosquito lines for further functional analyses.

## Materials and Methods

### Plasmid construction

Plasmids for transgenesis were prepared by a combination of standard molecular cloning (Sambrook *et al.* 1989) and either multisite Gateway cloning (Invitrogen/Life Technologies) or GoldenGate Cloning (Engler and Marillonnet 2013; Geissler *et al.* 2011). Cloning details are available on request and the annotated sequences of major plasmids of interest are provided in Supporting Information, File S2, File S3, and File S5. For Gateway cloning, the various inserts to assemble were first cloned in the appropriate Multisite Gateway vectors. For GoldenGate cloning, the various inserts to assemble were first PCR-amplified using Phusion polymerase (ThermoScientific) with primers adding *Bsa*I sites on their extremities. These PCR products were cloned into the *Sma*I site of a pBluescriptSK (Stratagene) plasmid (in which we had mutated the endogenous *Bsa*I site) by performing a restriction-ligation reaction in a volume of 10 µl with 10 ng of PCR product, 10 ng of plasmid, 0.5 µl of T4 DNA ligase, and 0.5 µl of *Sma*I for 1 to 16 hr at 25°. Ligase was inactivated for 10 min at 70°, and 0.5 µl of additional *Sma*I was added to re-open empty vectors. Following transformation, blue/white screening was used to identify positive *E. coli* clones. Plasmid DNA from white colonies was sequence-verified and used in a restriction-ligation reaction containing 40 fmol of destination transgenesis vector (carrying a phage ΦC31 *att*B site and a *LacZ* cassette flanked by *Bsa*I sites), 40 fmol of each insert-containing plasmid, 1 µl T4 DNA ligase, 1 µl *BsaI*, 2 µl 10× *BsaI* buffer, and 1 mM ATP in a total volume of 20 µl. The reaction was cycled three times at 37° (10 min) and at 20° (10 min), followed by one 50-min step at 20°, one 20-min step at 50°, and one 10-min step at 70°. The last two steps were omitted if reactions involved modules carrying internal *BsaI* sites. The variable *BsaI* overhangs in the destination vector and in each of the modules’ extremities were designed to allow seamless ligation of all inserts in the desired sequential order. Enzymes used were from Fermentas/Thermofisher and New England Biolabs (*Bsa*I). The puromycin N-acetyl transferase resistance gene was amplified from plasmid pTG6529 (Dimarcq *et al.* 1997). The *Op*IE2 promoter was amplified from pIB/V5-His (Invitrogen). Cre recombinase was amplified from pxCANCre (Kanegae *et al.* 1995). Transposase was subcloned from the helper plasmid used by Catteruccia *et al.* (2000); integrase was from p3xP3-eGFP/vas-phiattB (Bischof *et al.* 2007). The kanamycin-resistant pDSAG transgenesis vector and its derivatives were assembled in a pENTR backbone (Invitrogen). The sequence of the *rrnB* transcription terminator is caaataaaacgaaaggctcagtcgaaagactgggcctttcgttttatctgt.

### Mosquito rearing and transgenesis

**Figure 1:**
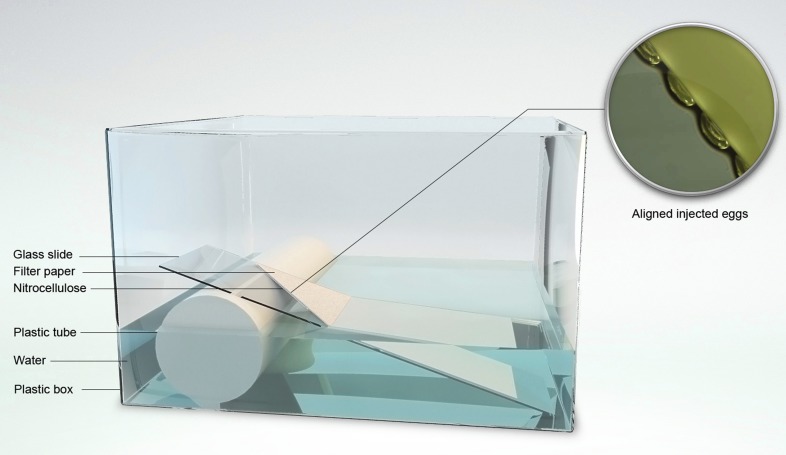
Set-up for handling mosquito eggs following micro-injection. Aligned injected eggs are left undisturbed on the microscope slide, which is dipped in water as shown, in a square plastic box (10 cm side). Capillarity through the Whatman filter paper and nitrocellulose membrane keeps the eggs wet and larvae will spontaneously crawl into the water on hatching.

*A. gambiae* mosquitoes were maintained in standard insectary conditions (28°, 75–80% humidity, 12-hr/12-hr light/dark cycle). Larvae were raised in deionized water and fed finely ground TetraMin fish food. Embryo microinjection was performed essentially as described (Fuchs *et al.* 2013; Pondeville *et al.* 2014). Freshly laid eggs were directly aligned against the edge of a nitrocellulose membrane kept wet with overlaying filter paper soaked with demineralized water. A mix of plasmids totaling 400 ng/µl of DNA (0, 1 mM NaHPO_4_ buffer pH 6.8, 5 mM KCl, 60 ng/µl helper plasmid, and generally 85 ng/µl of each of four distinct transgenesis plasmids) was injected under a Nikon Eclipse TE2000-S inverted microscope using an Eppendorf Femtojet injector and TransferMan NK2 micromanipulator. Injections were performed using the compensation pressure of the device, which was kept at 6000 hPa to promote a constant moderate flow of the DNA solution out of the quartz capillary. Microinjected eggs were left undisturbed on the injection slides, which were placed diagonally in a container with 1-cm-deep demineralized water, the part of the filter paper most distant from the eggs was dipped in water so that eggs remained wet by capillarity (Figure 1). Adult mosquitoes that survived microinjection were separated according to sex and crossed *en masse* to an excess of fresh wild-type adults. Neonate progeny larvae from several successive gonotrophic cycles were screened by spotting groups of 50–80 onto the wells of a 24-well teflon-coated diagnostic slide (Erie Scientific, Menzel GmbH, Braunschweig, Germany) under a Zeiss Axiovert 200M fluorescence microscope. When a fluorescent larva was detected, it was carefully isolated from the remainder larvae with the cut tip of a P200 pipette.

### Puromycin selection

Puromycin hydrochloride powder (Sigma) was dissolved to 25 mg/ml in water to make a stock solution and stored at −20°. To kill nontransgenic larvae, unfed neonate larvae were placed in a 6-cm-diameter Petri dish with 8 ml of water. Eight µl of puromycin solution were added; larvae were fed a small amount of ground TetraMin fish food and placed at 28°. Three to 4 d later, surviving larvae were transferred to a larger container without drug selection and raised normally.

### Microscopy pictures

Fluorescent larvae were observed with a Zeiss Axiovert 200M fluorescence microscope mostly using a 5× objective. To immobilize larvae for photography with the Axiovision software, they were placed in a drop of water containing 10% tricaine and 1% tetramisole.

### Flow cytometry sorting of mosquito larvae with the COPAS system

Complex object parametric analyzer and sorter (COPAS) sorting was used as described (Marois *et al.* 2012). Briefly, newly hatched larvae were transferred to the reservoir of the large particle flow cytometry COPAS SELECT instrument (Union Biometrica, Holliston, MA, USA) equipped with a multiline argon laser (488, 514 nm) and a diode laser (670 nm) and analyzed and sorted with the Biosort5281 software with following acquisition parameters: Green PMT, 500; Yellow PMT, 500; Red PMT, 600; delay, 8; width, 6; and pure mode selection with superdrops. The flow rate was kept below 15 detected objects per second when dispensing larvae into Petri dishes for line purification.

### Immunoprecipitation and Triton X-114 fractionation

Mosquito extracts for immunoprecipitation were prepared as follows: 20 *P. berghei*–infected or control females were anesthetized 14 d after infection and bled by severing their abdomens with forceps and soaking in 350 µl IP buffer (50 mM TRIS pH 7.9, 100 mM NaCl, 2 mM EDTA, 0.1 µg/ml BSA, complete protease inhibitors; Roche). Debris and cells were spun for 5 min at 1000 × *g* at 4°; the supernatant was recovered and spun again for 5 min at maximum speed. Supernatant was transferred to a new tube and precleared with a nonspecific IgG2a antibody (4 µl ascites, ∼20 µg of antibody) for 1 hr at 4° with gentle shaking. The nonspecific antibody was removed with 40 µl of protein A–coupled Sepharose beads (GE Healthcare), which were recovered by centrifugation, washed 5 times for 10 min in 50 mM TRIS pH 7.9 successively with and without 500 mM NaCl, resuspended in 25 µl protein gel loading buffer, and regarded as the IP control with nonspecific antibody. The supernatant was then subjected to the same treatment with anti-lipophorin monoclonal IgG2a antibody 2H5 (Rono *et al.* 2010). Ten µl of each sample were loaded on a 12% SDS-PAGE gel for Western blotting with monoclonal antibodies against lipophorin (2H5) and CSP (3D11) (Yoshida *et al.* 1980).

For fractionation, a solution of 10% Triton X-114 was stirred at 4° in PBS and preconditioned at 37° overnight. The next day, the upper phase was discarded and replaced with the same volume of cold PBS and homogenized. This step was repeated twice with overnight incubation at 32°. The last homogenate was stored at 4°. Triton X-114 concentration was then determined to be 15% by reading the optical density at 260 nm and comparing with that of the initial 10% solution. Mosquito extracts were prepared as above by bleeding 175 infected females in 2 ml of IP buffer. An aliquot of extract was kept as input control; the rest was subjected to immunoprecipitation with 2H5. Beads were resuspended in 100 µl PBS; 15% preconditioned Triton X-114 was added to a final concentration of 1%; and 200 µl of input extract or post-IP supernatant were subjected to the same treatment. Solutions were cooled on ice, homogenized and kept on ice for 1 hr with periodic vortexing, then incubated at 37° for 10 min for phase separation, rehomogenized and cooled on ice, and centrifuged at 14,000 × *g* for 15 min at 4°. The beads were collected and resuspended in protein gel loading buffer. The supernatant was then subjected to phase separation at 37° for 10 min and centrifuged. The detergent phase was saved and the aqueous phase was re-extracted as above with a new addition of Triton X-114. The two detergent phases were pooled, as well as the two aqueous phases, and supplemented with protein gel sample buffer.

## Results and Discussion

### Lessons from transposon-based *A. gambiae* transgenesis

**Figure 2:**
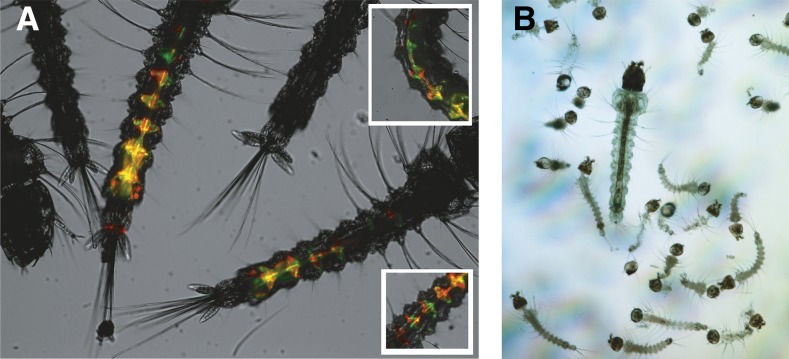
(A) Red/green mosaic transient expression following embryo micro-injection. *A. gambiae* embryos were injected with a mix of transgenesis plasmids expressing either GFP or RFP at a concentration of 100 ng/µl each. In surviving larvae showing transient expression of the fluorescent markers, subsets of cells frequently express only one of the two fluorescent markers. Insets show additional transiently expressing mosaic larvae. (B) Puromycin resistance as a new transgenesis selection marker for mosquito larvae. Neonate transgenic *A. gambiae* larvae expressing a puromycin resistance gene, and their nontransgenic siblings, were placed in a Petri dish containing 8 ml water, 25 µg/ml puromycin, and a small amount of ground fish food. Nontransgenic larvae died within 3 d. The only puromycin-resistant larva is the large surviving one.

Initial success in germline transformation of *Anopheline* mosquitoes has been achieved using constructs made in a disarmed *piggyBac* transposon, with the *piggyBac* transposase enzyme being expressed from a separate "helper" plasmid co-delivered with the transgenesis plasmid during embryo microinjection (Catteruccia *et al.* 2000; Grossman *et al.* 2001). Several dozens of transgenic *A. gambiae* lines have been generated in a few laboratories using this technique, but efficiency remained low (with remarkable exceptions, *e.g.,* Lynd and Lycett 2012). We hypothesize that transgenesis efficiency is low because only few plasmid copies are incorporated into individual nascent germ cells of the microinjected embryo. As a result, the transposase helper and the transposon-containing plasmids may not always be present in the same germ cell. This notion is supported by the observation of episomal expression of fluorescent markers in larvae obtained from injected embryos. In those injected with a mix of plasmids encoding RFP or GFP, we observed many cells with transient expression of only one of the two fluorescent proteins in a red/green mosaic pattern (Figure 2A).

**Figure 3:**
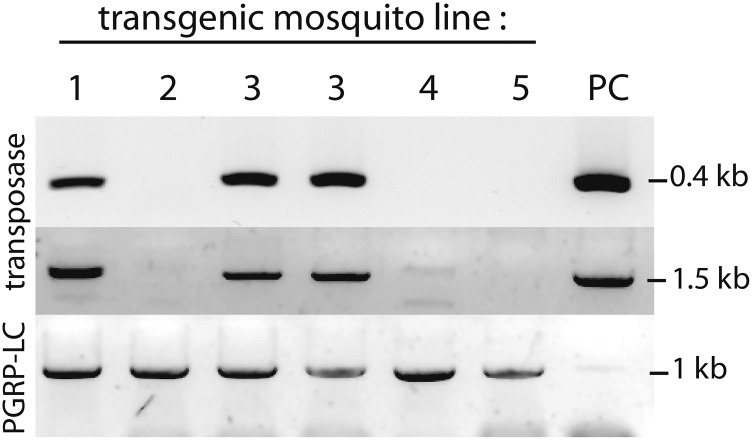
Inadvertent integration of transposase in some transgenic lines. PCR on genomic DNA extracted from five transgenic *A. gambiae* lines (two independent genomic DNA preparations from line 3 were tested here) obtained by injecting a *piggyBac* construct harboring the transposase gene in its plasmid backbone reveals that the transposase gene has co-integrated in some lines. The upper panel shows PCR products obtained with primers 5′-CCACTCCGCCTTTAGTTTGA-3′ and 5′-GGGAAGAGGAACACAGACCA-3′, amplifying a 401-bp fragment internal to the transposase ORF. The middle panel shows PCR products obtained with primers 5′- GTCTGGTGTCGGGGATCCT -3′ and 5′- ACATGAGCCTGACGTCATCG -3′, amplifying 1.5 kb from the transposase ORF. Bottom panel shows quality control of the genomic DNA samples with primers amplifying the endogenous *A. gambiae* gene *PGRP-LC*.

To ensure that *piggyBac* transposase was consistently co-delivered with modified transposons and to increase the transgenesis success rate, we introduced the transposase-coding gene into the backbone of the transposon-containing transgenesis vector itself. To block undesired transposase activity within *E. coli*, it was necessary to insert the *E. coli rrnB* transcription terminator sequence (Mindrinos *et al.* 1994) (sequence in *Materials and Methods*) between the *hsp70* promoter and the transcription start of the transposase gene, resulting in a stable plasmid. We noted a dramatic improvement in transgenesis success rates when microinjecting transgenesis constructs using this new approach. In one experiment, we simultaneously injected both transposase-containing and noncontaining *piggyBac* plasmids. Transgenic larvae were recovered only from the former, suggesting that the transposase helper gene acts more efficiently when physically associated with the transposon. Interestingly, PCR analyses revealed that in two independent transgenic lines out of five tested, the transposase gene itself had also integrated into the genome of mosquitoes transformed with this type of plasmid (Figure 3). In one line, *transposase* segregated independently from the fluorescently marked transgene. In the other, *transposase* co-segregated with the transgene and must be embedded between two copies of the transposon integrated as a single block, as inverse PCR revealed standard insertion of the *piggyBac* borders in a genomic TTAA site (data not shown). Further work is required to clarify the transposase co-integration events, but possible explanations of its presence include: (i) transposase-encoding vector backbones flanked by unnaturally oriented TR acting as an independent transposon and (ii) transpositions from plasmid to plasmid or rolling circle plasmid multimerization before integration into the genome, resulting in vector backbone sequences being flanked by two transposons whose outermost borders mediated clean integration. This would imply that the integration of vector backbones, even devoid of transposase, may be a frequent but generally overlooked event in *piggyBac* transformation.

We sought to exploit the transposase transgene, but transposon re-mobilization attempts were not successful. This could result from the inefficiency of the *Drosophila* *hsp70* promoter to drive germline transposase expression once embedded in the mosquito genome, as opposed to injected as naked plasmid DNA into an embryo. Of note, the same promoter used to express *piggyBac* transposase in *Ae. aegypti* or *Minos* transposase in *An. stephensi* also failed to mobilize transposable elements in the germline (Sethuraman *et al.* 2007; Scali *et al.* 2007).

### From transposon to docking site-based transgenesis

Classical *piggyBac*-based transgenesis paved the way for development in *Anopheline* mosquitoes of a next-generation–type of transgenesis based on docking site integration (Meredith *et al.* 2011) in the wake of its development in *Drosophila* (Bischof *et al.* 2007; Fish *et al.* 2007; Groth *et al.* 2004) and in *Aedes aegypti* (Nimmo *et al.* 2006; Franz *et al.* 2011). *Streptomyces* phage ΦC31 integrase catalyzes recombination between specific *att*P and *att*B DNA sequences (Thorpe and Smith 1998). Transgenesis technology exploits this system for site-directed integration of *att*B-bearing plasmids into *att*P sites that were previously inserted into the genome by *piggyBac*-based transgenesis. Several such *A. gambiae att*P docking lines have been established and made available to the community (Meredith *et al.* 2011). Further refinement has been performed to associate a ΦC31 integrase transgene to the *att*P site to circumvent the need for co-injection of integrative plasmids with integrase helper plasmid or mRNA (Meredith *et al.* 2013). We developed additional *A. gambiae* docking lines (Table 1), which differ from the published ones by their genetic background (mainly G3 rather than KIL), by their genomic locations, and by the absence of a visible screening marker. Although CFP or DsRed in the published docking lines allow positive selection of the *att*P site—a major advantage to remedy accidental strain contaminations— they bar the possibility of using the same selectable markers for transgenesis. Therefore, when generating new docking lines, we flanked selectable markers with *lox*P sites. After obtaining homozygous transgenic lines, we excised the lox cassette by injecting transgenic embryos with a plasmid transiently expressing Cre recombinase (more recently, we also expressed Cre recombinase transgenically; see below). By backcrossing Cre-injected mosquitoes to their fluorescent parental line, the unlabeled docking sites were initially heterozygous over their fluorescent progenitor and subsequently made homozygous by counterselecting fluorescence. The markerless *att*P docking site recovered from these injections is linked to a single remaining *lox*P site and flanked by the *piggyBac* borders. Two lines (termed X1 and X13) harbor the *att*P docking site on chromosome 2L. The close proximity of the respective loci (<800 kb apart) allows transgene combinations that can subsequently be tracked as a single locus (recombining in <1% of mosquitoes). The X1 locus is located 12 kb away from the nearest predicted gene. The X13 locus is embedded within a >44-kb intron of gene AGAP005170. In line X6, *att*P nears the centromere on chromosome 3R and is located 20 kb away from the nearest gene. In the XK line (discussed below), *att*P is embedded within the ∼32-kb intron of gene AGAP001069 on chromosome X. The annotated sequence of these new docking lines in their genomic context is provided in File S1. The major advantage of marker-free docking lines is that new constructs carrying any fluorescent marker can now be inserted. They allow simultaneous micro-injection of at least five mixed distinct integrative constructs, each labeled by a distinct selectable marker (see below).

**Table 1:** New docking lines for *A. gambiae* transgenesis

Docking Line and Chromosome		Genomic Location	Parental Line (Genetic Background); Mode of Generation
XK	X	X: 22463468*^a^*	FK (Ngousso); crossed to Cre-expressing line C2S
X1	2L	2L: 10526503	VFS1 (G3); transient Cre expression
X13	2L	2L: 11322315	VFS13 (G3); transient Cre expression
X6	3R	3R: 53037011	W62 (G3); transient Cre expression

Lines derive from *piggyBac* construct insertions at the shown genomic loci and were obtained by subsequent Cre-mediated excision of a *lox* cassette encompassing the fluorescent marker and other transgenes. The docking sites are therefore marker-free, flanked by *piggyBac* borders, and contain a single residual *lox*P site. The full, annotated sequence of each insertion is available in File S1.

a Docking site is located within a 232-bp sequence element found in strain Ngousso and not present at this position in the PEST reference genome.

**Figure 4:**
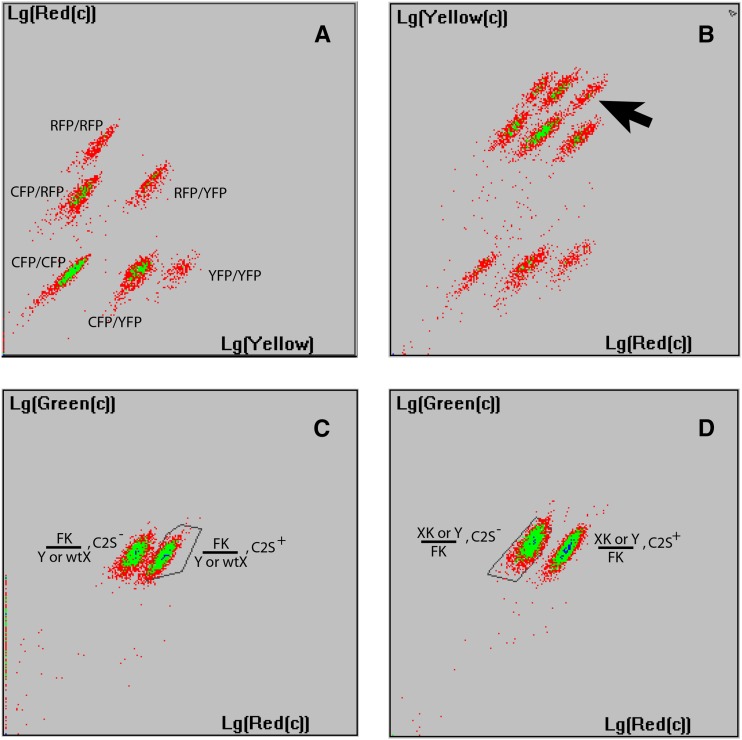
Examples of transgenic larvae population analysis with COPAS. Axes represent fluorescence intensity in a logarithmic scale. (A) Mosquito population carrying three transgenic constructs behaving as different alleles at the X1 docking locus. The different constructs are labeled with CFP (fluorescence undetectable by the sorter), RFP, and YFP. The six detected populations correspond to larvae carrying CFP/CFP (lowest fluorescence level), CFP/YFP, CFP/RFP, YFP/RFP (intermediate fluorescence levels), and YFP/YFP, RFP/RFP (strongest fluorescence levels). Note the absence of populations containing more than two transgenes due to the diploid nature of mosquitoes and a single docking locus. (B) F_2_ progeny of a cross between a GFP and an RFP transgenic line at two different loci. The nine larval clouds represent all possible genotype combinations (from left to right and bottom to top: +/+; +/+. RFP/+ ; +/+. RFP/RFP; +/+. +/+; GFP/+. RFP/+; GFP/+. RFP/RFP; GFP/+. +/+; GFP/GFP. RFP/+; GFP/GFP. RFP/RFP; GFP/GFP.). The black arrow points to larvae homozygous for both transgenes. (C) F_1_ progeny of homozygous FK (GFP) females crossed to heterozygous C2S (RFP) males. Segregating RFP yields a cloud of red positive larvae and a cloud of red negative larvae. All larvae inherited an intact GFP-positive copy of FK. (D) The larvae gated in C (one copy of C2S, one copy of FK undergoing *lox* cassette excision in the germ line) were raised to adulthood and males were backcrossed to FK females. The progeny shows the level of fluorescence of a single FK copy, indicating that the copy inherited from the father has now lost the fluorescence-coding gene. A single female from the larvae gated in (D) was selected to found the XK line.

It is widely recognized that docking-site transgenesis offers many advantages over transposons (Bischof *et al.* 2007; Meredith *et al.* 2013). First, transgenes land in a well-defined locus chosen to be homozygous viable and without fitness cost when loaded with exogenous sequences. Second, the phenotypes of different lines carrying a series of transgenes (*e.g.*, encoding variants of a protein of interest) can be directly compared because all are inserted at the same locus and subjected to identical positional effects. Third, should a transgenic line be lost, it can be regenerated using the same docking line and plasmid. Fourth, different transgenic constructs inserted in the same docking site and labeled with distinct screening markers can be maintained floating within a single mosquito population. A given transgenic construct required for an experiment can then be extracted from this population when needed. Considering the large amount of space, handling, and resources required for maintaining mosquito lines, this strategy optimizes these budgets by a factor of four. We routinely maintain mixed populations carrying up to four different transgenic constructs inserted at the same locus and labeled with CFP, YFP, GFP, and RFP (Figure 4A). Extraction of the desired homozygous genotype when needed and occasional verification/readjustment of the frequency of all transgenes present in the population are greatly facilitated by the automated sorting technology discussed below.

### Screening marker genes

We commonly inject docking line embryos with mixes of four plasmids carrying cyan fluorescent protein-encoding (CFP), green fluorescent protein-encoding (GFP), yellow fluorescent protein-encoding (YFP), and red fluorescent protein-encoding (RFP) marker genes. Successful transgenesis sessions will usually yield transgenic larvae for each distinct construct. This approach therefore cuts the time and work required to obtain transgenic mosquito lines by a factor of four. Furthermore, we established an additional novel selection marker for transgenic mosquitoes based on puromycin resistance, which allows screening for transgenic larvae by adding the antibiotic directly to the larval culture water (Figure 2B and Table 2). Larvae carrying the resistance transgene are able to grow in the presence of puromycin, whereas their wild-type siblings die within 3 d posthatching. This positive selection circumvents initial larval screening by fluorescence microscopy and may serve as a selectable marker for rare events produced by complex genetic engineering procedures such as homologous recombination. It is also useful as a nonfluorescent transgenesis screening marker, thus saving fluorescent protein options for tissue-specific reporter genes, or for combinations with multiple transgenes. A drawback of the antibiotic resistance marker is that no striking difference in antibiotic resistance exists between homozygous and heterozygous marker-expressing larvae (Table 2). This impedes the direct separation of homozygous and heterozygous larvae that is usually possible with fluorescent markers whose copy number correlates with fluorescence intensity. To circumvent this drawback, heterozygous puromycin-resistant mosquitoes can be crossed to any available line carrying a fluorescent marker docked within the same *att*P locus, selecting F_1_ offspring that are both puromycin-resistant and fluorescent. In the F_2_, the nonfluorescent larvae (25%) are homozygous for puromycin resistance.

**Table 2:** Puromycin resistance cassette allows selection of transgenic larvae

	Puromycin Concentration (µg/ml)					
Larval Genotype	0	5	10	20	40	80
X1/X1 (no *pac*)	170	93*	14**	0	0	0
SG1/X1 (one copy of *pac*)	n.a.	169	145	137	116	51**
SG1/SG1 (two copies of *pac*)	n.a.	188	183	177	168*	80**
LRIM-G/X1 (one copy of *pac*)	n.a.	160	150	163	130	105**
LRIM-G/LRIM-G (two copies of *pac*)	n.a.	172	179	137	152	126**
DG2/+ (one copy of *pac* on *piggyBac* transposon)	n.a.	173	177	180	179	164*

The SG1, LRIM-G, and DG2 transgenic constructs carry the puromycin acetyltransferase (*pac*) gene under the control of the baculovirus OpIE2 promoter as a selection marker for transgenesis. Two hundred neonate larvae of each of the shown genotypes (heterozygous or homozygous *pac*) were distributed with the COPAS machine in 8 ml of demineralized water containing the indicated concentration of puromycin. Larvae were fed a small amount of finely ground TetraMin fish food daily. The number of surviving larvae was scored after 6 d of larval growth (although results were almost identical by 4 d after puromycin exposure). n.a. = not analyzed. Asterisks denote larvae that were visibly delayed in their development (*=moderate delay, **=strong delay). Note that 20 µg/µl of puromycin is sufficient for the full elimination of control larvae. The SG1 and LRIM-G transgenic constructs were prepared in the Gateway pattBRfB2 (File S2) and pDSAP transgenesis vectors, respectively, and inserted into the X1 docking locus, whereas the DG2 construct was prepared in a *piggyBac* plasmid whose integration site was mapped to a different locus (chromosome 3L: 3708611).

### Flow cytometry sorting of transgenic mosquito larvae

The COPAS flow cytometer allows accurate selection of live larvae based on their fluorescence (Marois *et al.* 2012) and speeds the process of obtaining stable transgenic lines. With the exception of one chromosomal inversion balancing two-thirds of the second chromosome (Benedict *et al.* 1999), no efficient balancer lines are yet available for *A. gambiae*. Still, transgenic lines can rapidly be stabilized if coupling docking-site–based transgenesis with the use of automated sorting. Docking site transgenesis virtually always results in insertion at the defined unique genomic location [to date, we have never observed accidental integration at secondary genomic sites in *A. gambiae* in contrast to *Aedes aegypti* (Nimmo *et al.* 2006) and *Drosophila* S2 cells (Groth *et al.* 2004)]. Where a COPAS sorter is available, homozygous larvae (containing two copies of the fluorescence marker) can be retrieved immediately from the F_2_ progeny, whereas less fluorescent heterozygotes and nonfluorescent wild-types can be discarded. Stable homozygous populations of thousands of larvae are thus obtained within minutes, rendering the process simpler than in *Drosophila*, where larvae or adults are usually selected manually (although COPAS sorting of embryos/larvae is also possible in this species). Furthermore, distinct fluorescent markers can be combined to establish doubly-homozygous lines (Figure 4B) or to select compound heterozygous lines if constructs are inserted at the same docking site. This technological advance is particularly interesting for the prospect of mass production of single-sex mosquito populations. Many field interventions against insect pests, including the sterile insect technique (SIT) (Dyck *et al.* 2005; Scott *et al.* 2014), the release of insects carrying a dominant lethal (RIDL) (Alphey *et al.* 2014), or the prospective release of males carrying genes conferring resistance to *Plasmodium*, rely on mass production of purely male populations that this tool could facilitate. Any transgene carrying a fluorescent marker located on a sex chromosome can be exploited for single-sex population sorting (Marois *et al.* 2012; Bernardini *et al.* 2014).

### A transgenesis vector kit for convenient construct assembly

**Figure 5:**
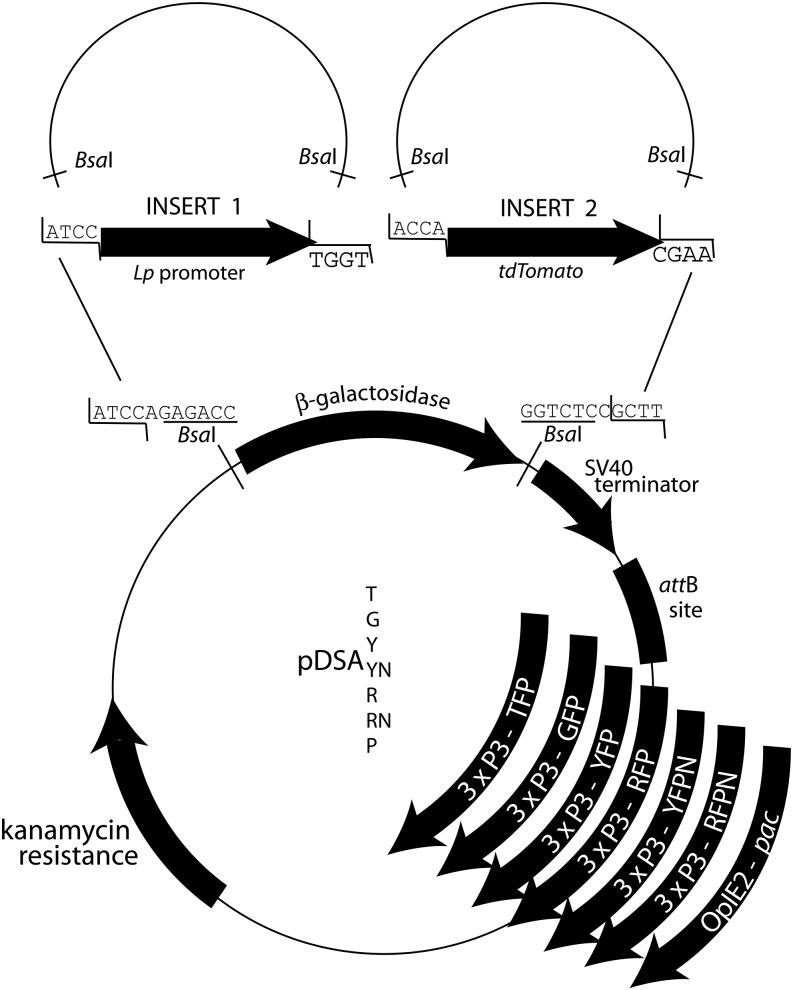
GoldenGate Cloning plasmids for transgenesis. Scheme of the pDSA plasmid series and an example of GoldenGate cloning. The final letter of the plasmid name indicates the identity of the transgenesis selection marker (T: mTurquoise2; G: eGFP; Y: YFPvenus; N: with NLS; R: DsRed2; P: puromycin acetyl transferase). The *att*B site mediates insertion at a genomic *att*P docking site. Constructs of interest are assembled between the two *Bsa*I sites (sequence underlined), which cut, leaving the indicated cohesive ends. The example shows the assembly of the *Lp* promoter with the td*Tomato* reporter gene that yielded the reporter line shown in Figure 6C. Each component is provided by a donor plasmid carrying appropriate *Bsa*I sites.

To facilitate the assembly of complex transgenic constructs in an *att*B site–containing transgenesis plasmid carrying the desired screening marker, we generated a collection of destination vectors compatible with GoldenGate cloning, a powerful technique allowing the seamless ligation of any number of inserts in the desired order (Engler and Marillonnet 2013). GoldenGate cloning is based on ligation of multiple inserts in sequential order through the action of type II restriction enzyme *Bsa*I and of T4 DNA ligase in a single restriction-ligation reaction, followed by transformation into competent *E. coli* cells. For correct assembly, each insert is flanked by appropriate *Bsa*I sites generating variable nonpalindromic cohesive ends designed for ligation to the next adjacent insert (Figure 5). The 5′-most insert (often a tissue-specific promoter) and the 3′-most insert (often the 3′ end of a gene of interest) are designed to produce *Bsa*I overhangs compatible with each of the plasmid’s two *Bsa*I cut sites. Successful sequential ligation removes a *LacZ*-containing cassette from the vector, enabling blue/white screening of positive *E. coli* transformants. Our vectors pDSAT, pDSAG, pDSAY, pDSAR, pDSAYN, pDSARN, and pDSAP (Figure 5) carry one of the following selectable markers: mTurquoise2, eGFP, YFPvenus, DsRed, YFPnls, DsRed2nls, or puromycin acetyl transferase (*pac*), respectively. mTurquoise2 is an improved version of CFP (Goedhart *et al.* 2012); YFPnls and DsRednls contain a nuclear localization signal (NLS). These fluorescent markers are expressed in the eyes and nervous system under the control of the 3xP3 promoter element (Berghammer *et al.* 1999). They allow visual screening under a fluorescence microscope or via COPAS sorting, whereas the puromycin resistance *pac* gene expressed under the control of the viral *OpIE2* promoter allows the selection of transgenic larvae by adding 25 µg/ml puromycin in culture water (Figure 2B and Table 3). In addition, the plasmids contain an SV40 terminator to stop the transcription of transgenes. We used pDSAY and pDSAR to sequentially assemble the seven inserts needed to generate each of the two TALEN transgenes used to obtain mutant mosquitoes in the immunity gene *TEP1* (Smidler *et al.* 2013). All vectors except the most recent NLS derivatives have been used to generate various transgenic lines. File S2 provides the annotated DNA sequence of each. All have been deposited in the Addgene repository for distribution to the research community. Notably, these plasmids will not only be useful for *Anopheline* mosquito transgenesis but also may be used for transgenesis in any organism in the genome of which *att*P sites have been inserted and supporting fluorescence expression from the large-spectrum 3xP3 promoter, for example, *Aedes* mosquitoes (Franz *et al.* 2011; Labbé *et al.* 2010; Nimmo *et al.* 2006; Kokoza *et al.* 2001).

**Table 3:**
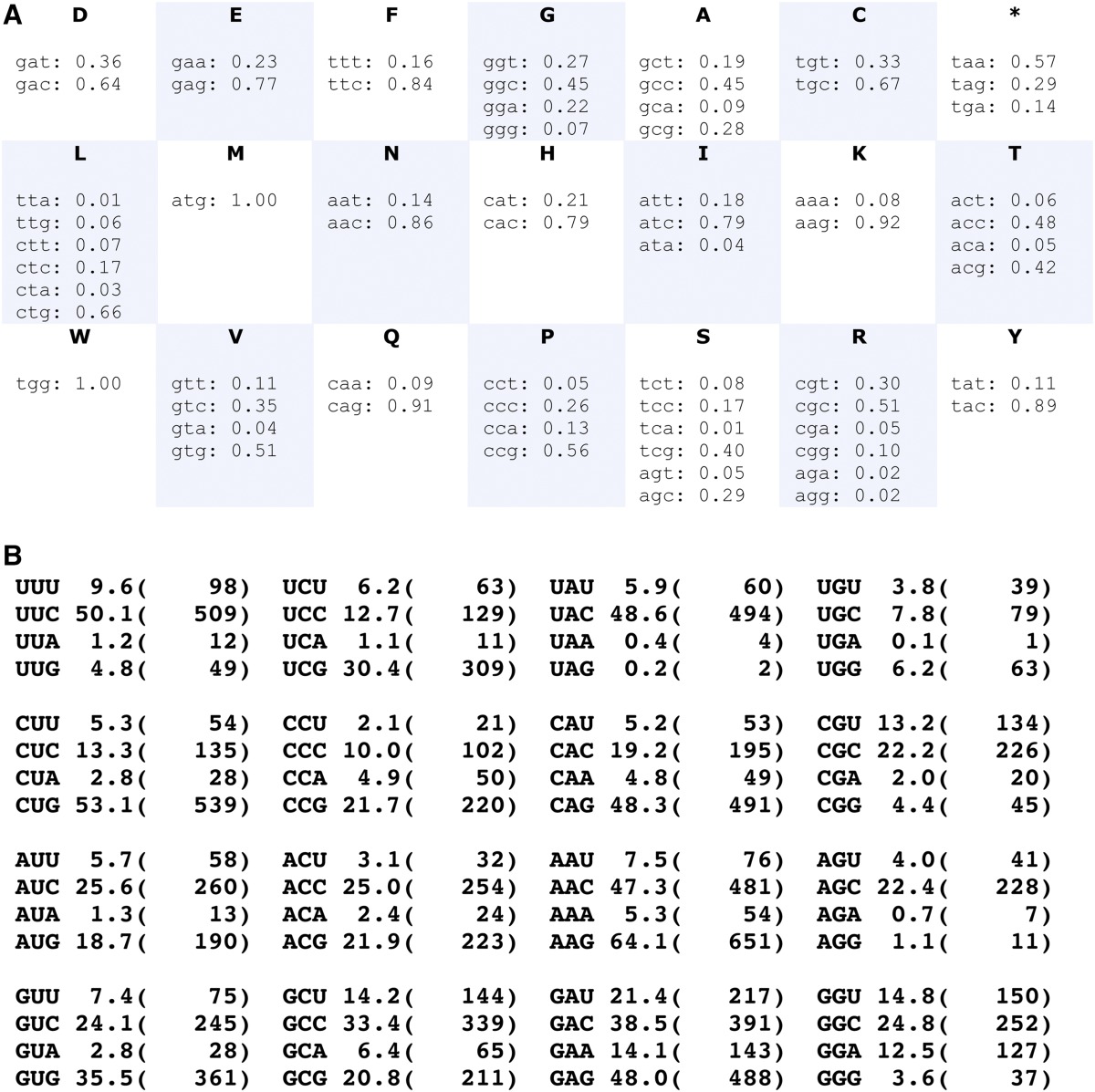
Codon usage preference in a subset of medium to highly expressed *Anopheles gambiae* genes

This codon preference table was designed to favor expression of codon-optimized heterologous transgenes in transgenic mosquitoes. It does not reflect global codon usage preference across the genome (which contains many genes expressed at very low levels), but rather codon preference in a small set of medium to highly expressed genes totalizing 10,160 codons (see text). A: Frequency at which a codon is used in the selected coding sequences for a given amino acid. Note that some codons are particularly rare, such as Leucine TTA or Arginine AGA and AGG. B: The first number indicates the frequency of each codon per 1000 codons (any amino acid) in our selected coding sequences. Numbers in parentheses indicate the number of times a given codon was used in a total of 10,160 codons. Table was generated by the GENEius online tool; the latter is needed as input for codon optimization.

We have also made available our *piggyBac* cloning vectors compatible with the Gateway system as well as our preferred integrase, transposase, and Cre recombinase-coding helper plasmids (annotated vector sequences provided in File S2). These were constructed by placing phage ΦC31 *integrase*, *piggyBac transposase*, or *Cre recombinase* under the control of the germline-specific *vasa* promoter *vas2* (Papathanos *et al.* 2009), yielding pENTR R4-vas2-integrase-R3, pENTR R4-vas2-Transposase-R3, and pENTR L1-vas2-Cre-L4, respectively. We noted improvement in transgenesis efficiency when integrase or transposase were expressed under the control of the *vasa* promoter rather than the *Drosophila hsp70* promoter (Table S1 and data not shown).

### Cre recombinase–expressing mosquitoes for novel applications

Direct injection of the Cre-encoding plasmid into mosquito embryos to generate marker-free docking lines X1, X13, and X6 (Table 1) resulted in very rare lox cassette excision events, which we recovered by tracking the loss of one copy of the fluorescent marker. COPAS sorting was crucial for isolating a few heterozygous larvae with an intermediate level of fluorescence among thousands of highly fluorescent homozygous siblings. We aimed to create a more efficient lox cassette excision system by transgenically expressing Cre recombinase. Cre recombinase, N-terminally fused to I-SceI nuclease via a self-cleavable 2A peptide link (Fang *et al.* 2005), was cloned under the control of the *vasa* promoter in the pDSAR vector. We inserted this transgene into the EE docking line (Meredith *et al.* 2011), chosen for its absence of endogenous *lox*P site (in contrast to our “X-lines”). CFP and DsRed doubly fluorescent larvae founded the C2S (Cre-2A-I-SceI) line. To test Cre activity and generate an X-linked marker-free docking line, we crossed homozygous C2S males to homozygous FK females. The FK line has been described as a powerful tool for sex-specific high-throughput larvae sorting (Marois *et al.* 2012). This *piggyBac* insertion on the X chromosome contains an *att*P docking site and a lox cassette encompassing the 3xP3-GFP screening marker and additional transgenes. In the progeny of the cross, we found that all F_1_ larvae had retained one copy of GFP and, therefore, the *lox* cassette (Figure 4C). This result indicated that sperm cells from Cre-expressing fathers did not transfer significant amounts of active Cre protein or mRNA to their offspring. Thus, Cre activity was only expected to begin in the germ cells of F_1_ larvae, where Cre expression is directed by the *vasa* promoter. We backcrossed F_1_ males (carrying C2S on the second chromosome, FK on the X chromosome) to FK females and analyzed the progeny with the COPAS instrument. It appeared that 100% of larvae carried only one copy of GFP (Figure 4D), indicating that all X chromosomes from the F_1_ males had now lost the *lox* cassette. A specific PCR product corresponding to the excised *lox* cassette could be amplified in 17 out of 19 F_2_ female individuals; sequencing the PCR products confirmed clean cassette excision, leaving a single remaining *lox*P site (data not shown). The lack of PCR amplification in the remaining two females despite their loss of GFP fluorescence was likely due to inefficient PCR.

We backcrossed a single F_2_ female containing the expected excision event to FK males to generate a marker-less X-linked docking line termed XK (Table 1). The F_2_ progeny from this XK/FK × FK/Y cross was sorted by COPAS to remove all FK chromosomes. The XK line is, to our knowledge, the first X-linked docking line available in *A. gambiae* and will be useful for studies and technology development involving the sex chromosomes. XK complements the recently reported Y-chromosome linked docking line T4 (Bernardini *et al.* 2014). Intriguingly, while the parental line FK, and XK itself, are both homozygous and hemizygous viable, we observed that some transgene insertions into the docking site result in homozygous lethality. This is not due to the nature of the inserted transgenes, which did not encode any toxic factor. The cause of this phenomenon is currently unknown, but we speculate that the addition of certain sequences may perturb proper splicing of the ∼32-kb intron of gene AGAP001069 (ortholog of *Drosophila* *Surfeit 4*) in which the docking site is embedded.

The Cre-expressing and I-SceI–expressing C2S line will be useful in other approaches requiring cassette excision. In particular, it may be used to develop gene exchange systems based on homologous recombination as in *Drosophila* (Rong and Golic 2000; Baena-Lopez *et al.* 2013). The high efficiency of transgenically expressed Cre recombinase also paves the way for developing conditional knockout approaches similar to those used in mouse genetics (Feil 2007). We verified that the I-*Sce*I moiety of the construct is also functional, cleaving and mutating at least 9% of chromosomes harboring an I-*Sce*I site on a transgene. Experiments using an I-*Sce*I site embedded within the GFP marker of a transgene will be needed to quantify I-*Sce*I activity more precisely.

### Characterization of the expression pattern of cloned promoters

**Figure 6:**
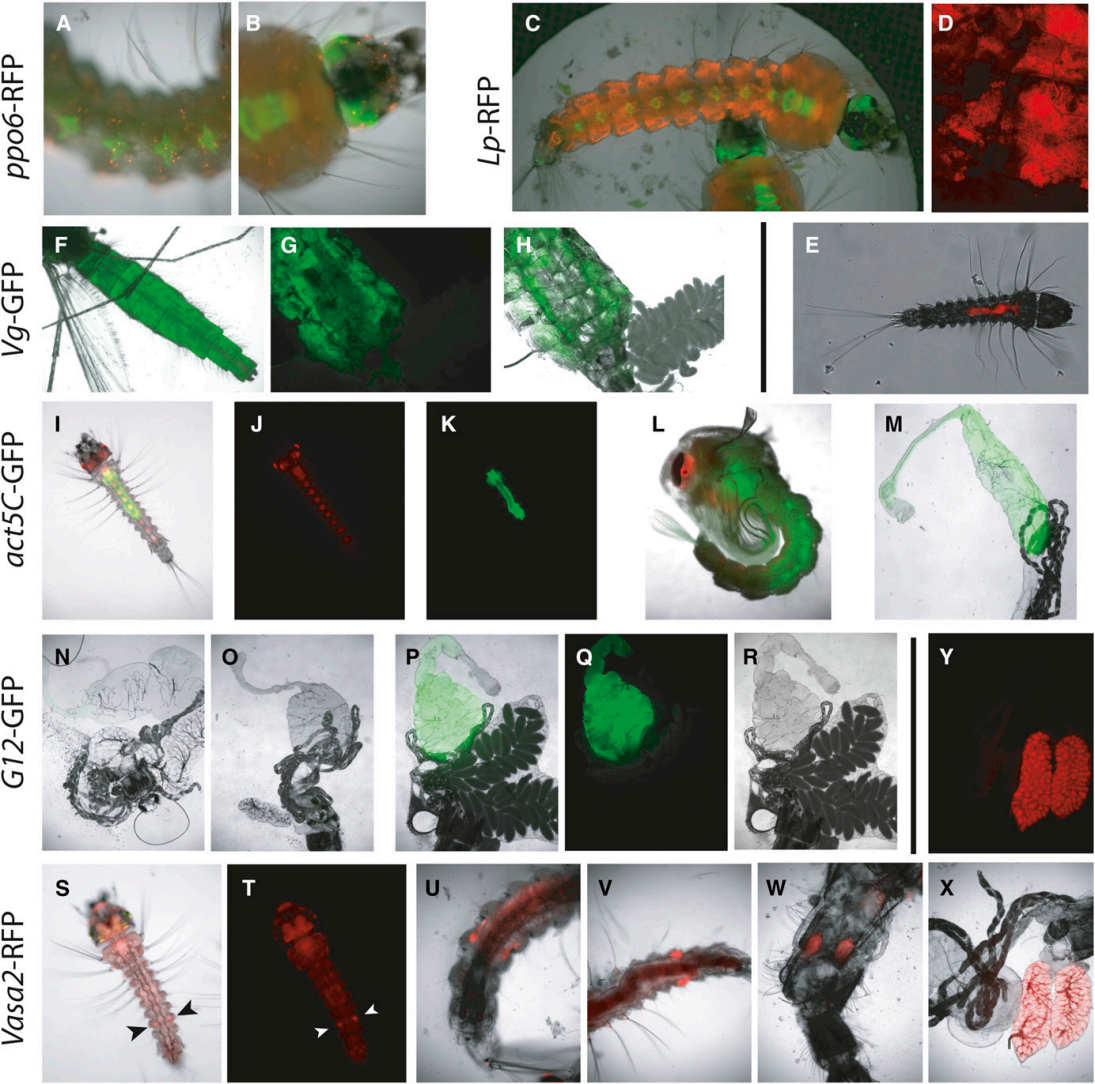
Transgenic reporter *Anopheles gambiae* lines. All pictures were taken with the 5× objective of a Zeiss Axiovert fluorescence microscope yielding an 80× visual magnification, unless otherwise noted. (A and B) *ppo6*-RFP fourth instar larva, central ventral abdominal region, and head. Note the red fluorescent hemocytes throughout the larval body. Green fluorescence reflects the activity of the 3xP3-YFP transgenesis selection marker. (C–E) *Lp*-RFP. (C) Composite of two pictures showing the entire body of a fourth instar larva. Fat body tissue is revealed by red fluorescence. (D) Fat body tissue dissected from a transgenic adult female, observed with a 10× objective. (E) Neonate larva showing initial *Lp* promoter activity in the intestine. (F–H) *Vg*-GFP adult blood-fed female mosquitoes. GFP expression in the fat body renders the whole abdomen fluorescent (F). (G and H) Dissected abdomen with GFP and (GFP + bright field) overlay showing the ovary. (I–M) *actin5C*-GFP mosquitoes with 3xP3-RFP transgenesis marker. (I–K) Larva pictured in merged, red, and green channels. (L) Pupa (merged channels). (M) Dissected intestine from an adult female. (N–R) *G12*-GFP, intestine dissected from adult females without blood feeding (N and O, merged bright field + green channels) or with blood feeding (P–R, merged bright field + green and split channels). (S and T) *vas2*-tdTomato neonate larva showing systemic red fluorescence inherited from maternal deposition in the oocyte in addition to endogenous gonad expression (arrowheads point to gonads). (U and V) tdTomato-expressing gonad in female (U) and male (V) fourth instar larvae. (W) Testis in dissected adult male abdomen. (X and Y) Ovary in dissected adult female abdomen. (U–X) Merged bright field and red channels. (Y) Red channel.

We present six novel transgenic mosquito reporter lines inserted in the X1 docking site, reflecting promoter expression in four different tissues: hemocytes, fat body, midgut, and germline (Figure 6; annotated sequences of transgenesis plasmids in File S3).

Studies on the immune system would greatly benefit from transgene expression in the mosquito hemocytes. We tested the ability of the promoter of the *A. gambiae* prophenoloxidase gene *PPO6* (AGAP004977) to drive expression of the *tdTomato* reporter gene in hemocytes. *tdTomato* was placed downstream of a 1.7-kb fragment amplified from the 5′ flanking region of the *PPO6* coding sequence and inserted at the X1 locus. This putative promoter fragment additionally contains some of the 5′-flanking region of *PPO9* (AGAP004978), another prophenoloxidase gene located upstream and in the opposite orientation to *PPO6*. Hemocytes were visible by intense red fluorescence in transgenic larvae (Figure 6, A and B) from the fourth instar until late adult life, suggesting that *PPO6* is expressed in hemocytes. Fluorescent hemocyte numbers varied greatly from individual to individual, with some larvae appearing to have none at all. This variability suggests that only a subset of hemocytes express *PPO6* and/or that expression is induced by a stimulus such as encounters with certain microorganisms. Alternatively, the variability of the individual expression level in hemocytes could also reflect positional variegation of that particular locus in this particular cell type. Further research is needed to fully characterize the activity of this promoter.

Lipophorin (Lp, AGAP001826) is the major insect fatty acid and cholesterol transporter protein. It scaffolds lipoprotein particles that shuttle between the midgut (where they collect digested lipid), fat body (a lipid storage and immune tissue), developing larval tissues, flight muscles, and the female ovaries after a blood meal. *Lp* is constitutively expressed in the fat body and its expression further increases in females 12 hr to 48 hr after a blood meal (Atella *et al.* 2006; Marinotti *et al.* 2006; Rono *et al.* 2010). t*dTomato* was cloned under the control of a 1.6-kb fragment from the *Lp* promoter region. The resulting transgenics display fat body–specific reporter expression in both larvae and adults (Figure 6, C and D). Thus, the *Lp* promoter can be used to express transgenes specifically in the fat body during most of the mosquito lifecycle. Interestingly, in neonate larvae that do not yet have a developed fat body, the Lp promoter appears to be active in the gut (Figure 6E).

Similar to Lp, Vitellogenin (Vg) is also a nutrient transporter highly expressed in the fat body, but only in adult females following a blood meal. We cloned 1.7 kb of the *Vg* (AGAP004203) regulatory region upstream of *GFP*. The resulting transgenics faithfully recapitulate the predicted *Vg* expression pattern: no detectable expression was seen during development, but it became intense 18 hr after the blood meal (Figure 6, F–H). Therefore, this promoter can be used to express transgenes in the female fat body in an inducible manner, which could enable the creation of innovative transgenic lines to reduce *Plasmodium* infection or for mosquito control. Unlike Vg protein, which is secreted and rapidly taken-up by the developing ovary, GFP stably accumulates in fat body cells, so that blood-fed females can be selected under a fluorescence microscope for at least 10 d postfeeding. In addition, the activity of the *Vg* promoter inserted at the X1 locus was similar to its expression when inserted on the X chromosome in the FK line. Besides the GFP transgenesis marker, FK expresses YFP under control of the *Vg* promoter. Using immunoblotting and immunofluorescence analysis, we showed that reporter expression in these mosquitoes reproduces the expression of endogenous *Vg* (Figure S1, A–C). Furthermore, RNAi experiments showed that key regulatory elements are present in the cloned *Vg* promoter because, similar to endogenous *Vg*, *YFP* expression was repressed by depletion of the NF-κB negative regulator Cactus (Rono *et al.* 2010) (Figure S1, D and E).

Just as the *Lp* and *Vg* promoters provide the possibility to respectively express transgenes in a constitutive and inducible fashion in the fat body, another pair of promoters enables constitutive or inducible expression in the midgut. *Drosophila Actin5C* is midgut-specific in *A. gambiae* and shows *GFP* expression at all stages of development (Figure 6, I–M). In contrast, the *A. gambiae* midgut-specific *G12* promoter that was tested previously in *A. stephensi* (Nolan *et al.* 2011) shows very low levels of basal expression (Figure 6, N and O) but becomes strongly induced after the blood meal (Figure 6, P–R). Interestingly, in contrast to the mosaic pattern observed in *A. stephensi*, all midgut cells homogeneously expressed GFP in *A. gambiae*. This makes it an attractive promoter to induce anti-*Plasmodium* factors in the midgut of transgenic mosquitoes designed to fight malaria.

Because many of our ongoing gene engineering experiments rely on the *vasa* promoter (Papathanos *et al.* 2009) for expression of enzymes in the germ cells, we wanted to ascertain the expression pattern of this promoter when inserted at the X1 docking site. For technical simplicity, our recombinant genes are terminated by SV40 terminator sequences rather than by the endogenous *vasa* 3′-UTR, making our constructs distinct from the published ones and giving rise to the possibility of distinct expression patterns due to this change. We inserted a *vas2-tdTomato*-SV40 reporter transgene at the X1 locus. Red fluorescence was observed in both male and female gonads during all developmental stages (Figure 6, S–Y), similar to the GFP pattern expressed from other genomic locations and with the native *vasa* 3′UTR (Papathanos *et al.* 2009). Like GFP, tdTomato mRNA and/or protein was deposited in oocytes, resulting in strong persistent red fluorescence throughout the body of larvae originating from a transgenic female (Figure 6, S and T; Figure S2), even in larvae that had not themselves inherited the transgene. In contrast, transmission of the transgene from a male did not result in inherited systemic expression of red fluorescence in nontransgenic larvae (Figure S2). In addition to gonadal red fluorescence due to the *vasa* promoter, some red fluorescence was also observed in larval eyes (Figure S2). This suggests that reporter gene expression is also influenced by the neighboring 3xP3 promoter element driving the YFP transgenesis marker, located immediately downstream of the *Vas2-tdTomato-SV40* terminator cassette in tandem orientation. Thus, the 3xP3 promoter element can act at some distance.

We generated additional reporter lines to characterize the expression pattern of antiparasitic genes *TEP1*, *LRIM1*, and *APL1-C* (G. Volohonsky and E. Marois., unpublished data). Collectively, the nine reporter lines show that the X1 docking locus is amenable to the expression of a diverse array of promoter-specific patterns, without obvious leakiness in nontarget tissues other than that due to enhancers incorporated in the construct.

### Codon optimization of heterologous transgenes

Transgenes derived from unrelated species may be poorly expressed if the coding sequence was not first adjusted to the codon usage preference of the target organism. For example, scientists working with the Nematode *Caenorhabditis elegans* commonly “wormify” heterologous genes before introducing them in the worm (Redemann *et al.* 2011). In *Drosophila*, codon optimization is sometimes performed (Bischof *et al.* 2007; Pfeiffer *et al.* 2010) but is not generally necessary because enzymes such as FLP recombinase, Gal4, and others from yeasts have been successfully expressed in their native form. In this section, we describe an example of a transgene derived from *Plasmodium berghei* that we could only express in *Anopheles gambiae* following codon optimization and the procedure we used for codon adjustment.

We initially observed that *P. berghei* sporozoites release large quantities of their major surface protein, circumsporozoite protein (CSP), into the hemolymph of infected mosquitoes. We discovered that a fraction of released CSP associates with mosquito lipoprotein particles, because some CSP was co-immunoprecipitated by anti-lipophorin antibodies (Figure S3). Fractionation assays in a Triton X-114 solution, which separates hydrophobic or lipid-linked proteins from soluble proteins (Bordier 1981), revealed that the majority of released CSP has lost its glycosylphosphatidylinositol (GPI) anchor (Figure S3, hemolymph extract), whereas all Lp-associated CSP is still GPI-anchored (Figure S3, immunoprecipitate). Conversely, all GPI-anchored CSP appears to be associated with Lp, because depletion of Lp particles by immunoprecipitation completely removes the detergent-fractionating form of CSP (Figure S3, post-IP supernatant compared to hemolymph extract). Association of a GPI-linked protein with insect lipophorin is reminiscent of previous findings in *Drosophila* (Panáková *et al.* 2005). Schistosoma parasites also release their GPI-anchored surface proteins onto mammalian host lipoprotein, causing perturbations in neutrophil function (Sprong *et al.* 2006). We hypothesized that the shedding of either (or both) forms of CSP by *Plasmodium* into mosquito hemolymph may benefit parasite development or transmission, for example by promoting sporozoite escape from the mosquito immune system or by affecting mosquito behavior. As these experiments initiated, a transcriptome profiling study reported an effect of CSP on immune gene expression when expressed transgenically in *Drosophila* (Yan *et al.* 2009). To examine the potential effects of CSP in mosquitoes in the absence of other confounding parasite factors, we attempted to express CSP transgenically in *A. gambiae*. Native *P. berghei CSP*, or a version thereof without GPI anchor, was placed under the control of the strong blood meal–inducible *Vitellogenin* promoter. Western blot analyses did not reveal any CSP expression after blood meal in any of three independent transgenic mosquito lines obtained by random *piggyBac* insertion (data not shown). We reasoned that because *P. berghei* genes are extremely AT-rich, the *CSP* codon composition might hamper expression by mosquitoes. To determine a codon frequency matrix favoring high protein expression in *A. gambiae*, we compiled the coding sequences of nine *A. gambiae* housekeeping, nutrient transporter, and immune genes encoding medium to highly expressed proteins: *Rab5*, *RPL19*, *Actin5C*, *Lipophorin*, *Vitellogenin*, *TEP1*, *APL1C*, *LRIM1*, and *PPO2*. From this total of 10,160 codons, we derived the codon frequency table shown in Table 3A. The frequency of each codon can also be represented per 1000 codons (Table 3B). The latter format was used as input codon preference table to optimize CSP using the online program GENEius (http://www.geneius.de/GENEius/Security_login.action). Furthermore, to evaluate the suitability of any exogenous coding sequence to be efficiently expressed in *A. gambiae*, we generated a Perl script (File S4) that attributes a “translation penalty index” for any input coding sequence. Codons of a given amino acid used more rarely in our reference table than a given input threshold (*e.g.*, 10%) contribute to elevating the penalty index of the input sequence. The global penalty index is the sum of individual penalties for all rare codons present in a gene. The output index is expressed per 1000 amino acids to standardize against protein length. If evaluated in this manner, then the *A. gambiae* genes that we used to define rare codons themselves show penalty indices that range between 8 (*actin5C*) and 21 (*Lipophorin*). The index for heterologous genes commonly expressed in *A. gambiae* can be as low as 1 or 4 (DsRed; GFP/YFP, which have been codon-optimized for human) or 6 (*Cre* recombinase), 26 (phage ΦC31 integrase), to as high as 126 (yeast Flippase) or 105 (*piggyBac* transposase), suggesting that the outcome of experiments involving *piggyBac* transposase may be improved if its coding sequence were codon-optimized.

Considering codons less frequent than 10% for a given amino acid, native *CSP* has a penalty index of 65. Codon optimization by the GENEius software resulted in a new penalty of 21. The optimized synthetic *CSP* sequence was inserted into the mosquito genome again under the control of the *Vitellogenin* (blood meal–induced) or *Lipophorin* (constitutive and further increased by a blood meal) fat body–specific promoters. For each promoter, we prepared a CSP version with and without GPI anchor, replacing the *Plasmodium* GPI anchoring signal (which may not be recognized as such in mosquitoes) with the GPI anchoring signal of the *Drosophila* Fasciclin protein. All versions were provided with the N-terminal secretion signal of the lipophorin protein (annotated sequence files of each transgene are provided in File S5). These four constructs assembled in our CFP-, GFP-, YFP-, and RFP-marked transgenesis vectors were injected simultaneously into 455 embryos of the X1 docking line. Approximately 65 of these survived to adulthood. Females and males were separated and crossed *en masse* to wild-type mosquitoes. We isolated all four desired transgenic lines from this single round of injections by recovering neonate larvae of each fluorescence color. CSP protein from the codon-optimized transgenes could easily be detected in transgenic mosquito tissues and hemolymph using the CSP monoclonal antibody 3D11 (Figure S3), indicating that codon optimization was sufficient to convert an inactive gene into an efficiently expressed gene. As expected of a functional GPI anchor, directing secreted protein attachment to the outer leaflet of plasma membranes, the GPI-containing versions of CSP remained tightly associated with carcass tissues, whereas the GPI-free version was released more efficiently into hemolymph (Figure S3). Subsequent analyses did not reveal any major impact of the transgenically expressed CSP on mosquito fitness, behavior, or susceptibility to the parasite (data not shown). Thus, the presence of CSP had no obvious effect on the susceptibility of transgenic mosquitoes to *P. berghei*, although we cannot rule out the possibility that any CSP activity was abolished by the absence of *Plasmodium*-specific post-translational CSP modifications in the mosquito, or by unnatural modifications added by the mosquito translational or secretory machinery onto the transgenic protein. Nevertheless, besides the importance of codon optimization, we conclude that the fasciclin GPI-anchoring signal and the lipophorin secretion signal can be used to manipulate the cellular localization of transgenically expressed proteins of interest.

## Conclusions

The tools we report here have permitted an unprecedented degree of genetic manipulation of the *Anopheles gambiae* genome, moving this species a step closer to the status of a model organism for genetic and biological studies. These tools have already proven instrumental for the successful establishment of targeted gene knockout using both TALENs (Smidler *et al.* 2013) and the CRISPR-Cas9 system in *A. gambiae*, which we have recently used to mutate seven target mosquito genes (E. Marois, unpublished data). These tools will assist the development of increasingly precise technologies for studies in vector biology and mosquito control. This work was conceived as a source of reagents and information for the mosquito community at large, and it is likely to benefit the study and control of other nonmodel insects, including disease vectors and agricultural pests.

## Supplementary Material

Supporting Information
