# Supplementary material for: Tools for Anopheles gambiae Transgenesis
Source: G3 (Bethesda). 2015 Apr 13;5(6):1151–63. doi: 10.1534/g3.115.016808 (PMC4478545; doi:10.1534/g3.115.016808)
Supplement: Supporting Information [file supp_5_6_1151__index.html]

Tools for Anopheles gambiae Transgenesis — Supporting Information 

# Tools for *Anopheles gambiae* Transgenesis

## Supporting Information for Volohonsky *et al.*, 2015

**Files in this Data Supplement:**

- Supporting Information - Figures S1-S3, Files S1-S5, and Table S1 (PDF, 1 MB)
- Figure S1 - Expression of the *Vg::YFP* reporter in the FK line reproduces endogenous *Vg* expression. (PDF, 348 KB)
- Figure S2 - Neonate larvae from heterozygous *vas2-tdTomato* x wild type crosses, red channel (top panels) and merged red plus bright field channels (bottom panels). (PDF, 402 KB)
- Figure S3 - *Plasmodium berghei* CSP in mosquitoes. (PDF, 566 KB)
- File S1 - Docking lines. (PDF, 168 KB)
- File S2 - DNA sequence of transgenesis vectors. (PDF, 236 KB)
- File S3 - DNA sequence of transgenesis plasmids used to generate reporter lines. (PDF, 184 KB)
- File S4 - perl script analyzing codon usage in an input sequence to evaluate how efficiently it will be expressed in *Anopheles gambiae*. (PDF, 192 KB)
- File S5 - Annotated DNA sequence of the four plasmids used to generate transgenic *A. gambiae* lines expressing codon-optimized CSP (with and without Fasciclin gpi anchor, with Lipophorin (Lp) or Vitellogenin (Vg) promoter). (PDF, 183 KB)
- Table S1 - Comparison of transgenesis efficiency using two promoters controlling *piggyBac* transposase expression. (PDF, 168 KB)
