# Supplementary material for: Tools for Anopheles gambiae Transgenesis
Source: G3 (Bethesda). 2015 Apr 13;5(6):1151–63. doi: 10.1534/g3.115.016808 (PMC4478545; doi:10.1534/g3.115.016808)
Supplement: Supporting Information [file supp_g3.115.016808_016808SI.pdf]

G3·Genes | Genomes | Genetics

Tools for *Anopheles gambiae* transgenesis

Gloria Volohonsky*[1], Olivier Terenzi*[1], Julien Soichot*[1], Daniel A. Naujoks[2], Tony Nolan[2], Nikolai Windbichler[2], Delphine Kapps[1], Andrea L. Smidler[1], Anaïs Vittu[1], Giulia Costa[3], Stefanie Steinert[1], Elena A. Levashina[3], Stéphanie A. Blandin[1] and Eric Marois[1]

*equal contributions

[1] INSERM U963, CNRS UPR9022, Université de Strasbourg, Institut de Biologie Moléculaire et Cellulaire, 15 rue René Descartes, 67084 Strasbourg, France

[2] Imperial College London, Division of Cell and Molecular Biology, Imperial College Road, London SW7 2AZ, UK

[3] Department of Vector Biology, Max-Planck Institute for Infection Biology, Charitéplatz 1, 10117 Berlin, Germany

Corresponding author: Eric Marois, IBMC, 15 rue René Descartes, 67084 Strasbourg,

France. Phone: +33 388 41 7113. E-mail: e.marois@unistra.fr

DOI: 10.1534/g3.115.016808

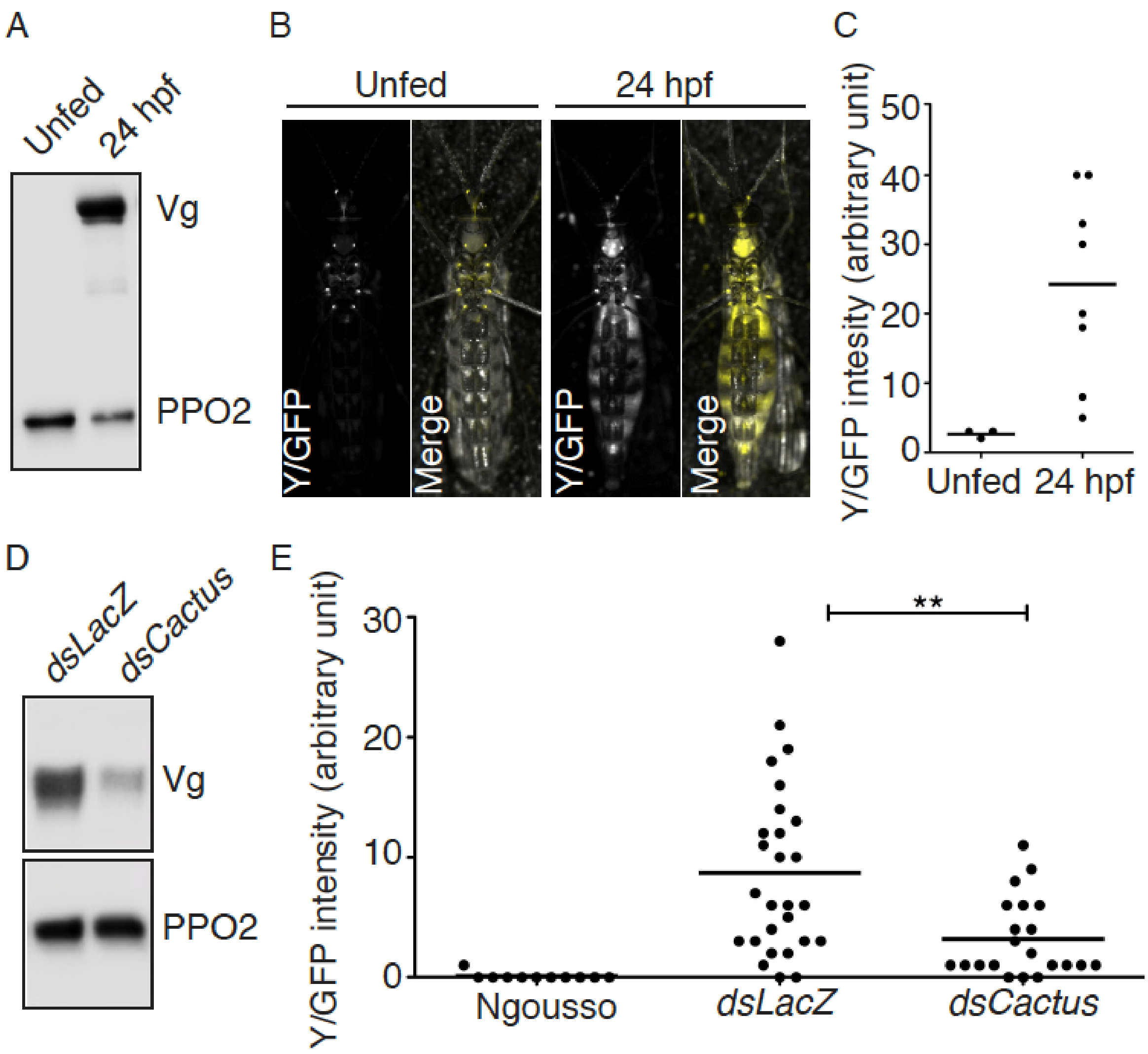

Figure S1 Expression of the *Vg::YFP* reporter in the FK line reproduces endogenous *Vg* expression. A: Expression of Vitellogenin (Vg) monitored by immunoblotting of mosquito hemolymph extracts from unfed and 24 h blood-fed FK mosquitoes. Anti-PPO2 antibodies were used as a loading control. B: Fluorescence analysis of FK mosquitoes reveals expression of the *Vg-YFP* reporter in the fat body 24 h post feeding while expression of the transgenesis marker *3xP3-GFP* in mosquito neurons is seen in both unfed and fed mosquitoes. C: Quantification of the YFP/GFP signal in the mosquito bodies expressed as mean of intensity. D: FK mosquitoes were injected with a control dsRNA (*dsLacZ*) or *dsCactus* and offered a blood meal infected with *P. berghei*. Immunoblotting was performed on the hemolymph extracted 52 h after infection using anti-Vitellogenin and anti-PPO2 antibodies. E: Quantification of YFP/GFP intensities from the same mosquitoes as in D 52 h after infection. Non-transgenic Ngousso mosquitoes were used as a control. **: p≤0.01 (1 way ANOVA). Pictures were acquired with a Leica AF6000E fluorescent stereomicroscope equipped with an M205 FA module. Images were analyzed with FIJI, by quantification of ROI in the delineated regions corresponding to the mosquito abdomen/thorax. Mean fluorescence intensity of the YFP/GFP signal was measured for each individual mosquito.

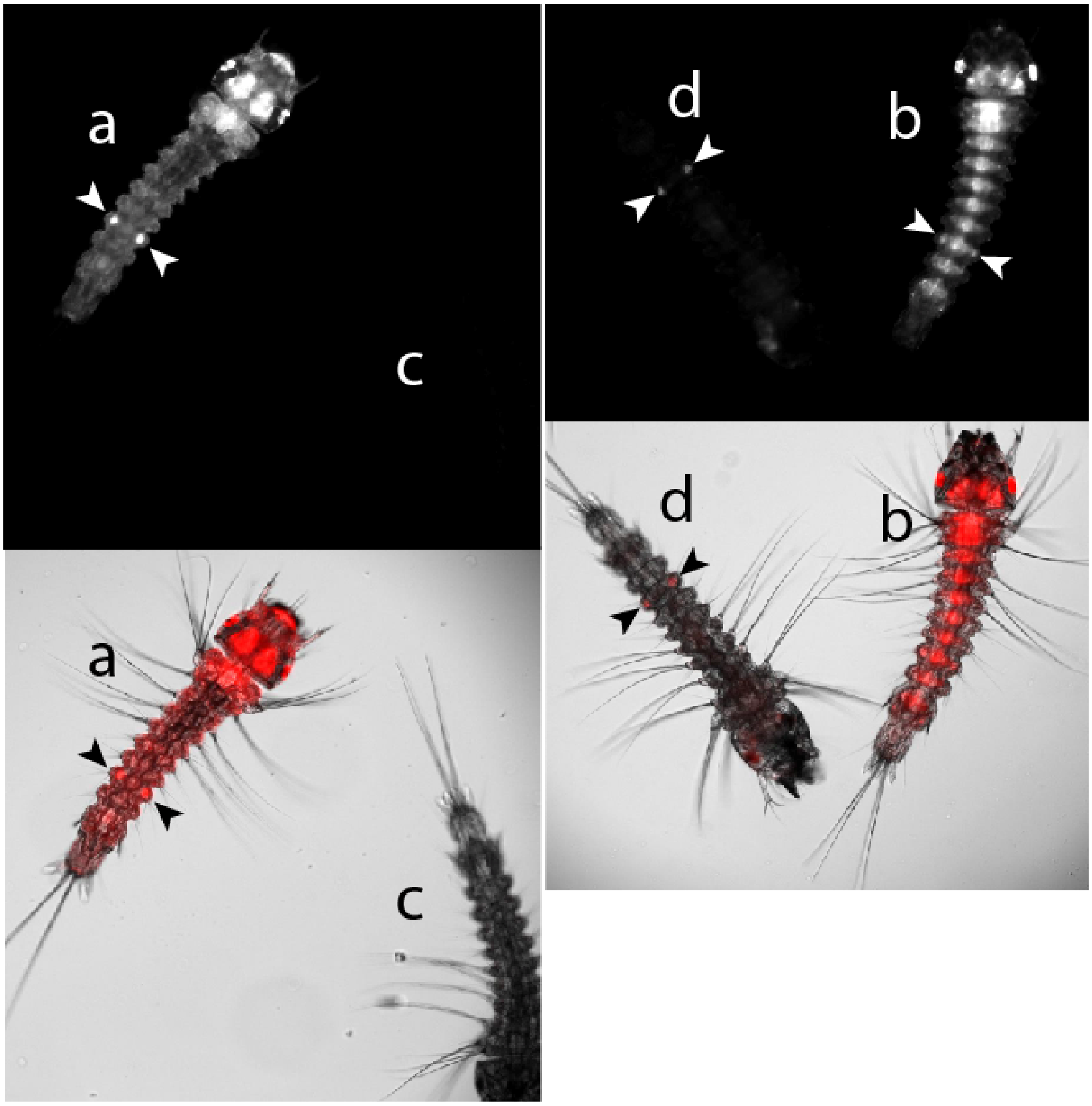

Figure S2 Neonate larvae from heterozygous *vas2-tdTomato* x wild type crosses, red channel (top panels) and merged red plus bright field channels (bottom panels). Larva (a), seen from the dorsal side, inherited the transgene and systemic red fluorescence from its mother. Larva (b) same, ventral side. Larva (c) had a transgenic heterozygous father but did not inherit the transgene. Larva (d) inherited the transgene from its father, expression is mainly restricted to the gonad and eyes. Expression in the eyes likely results from the nearby 3xP3 element.

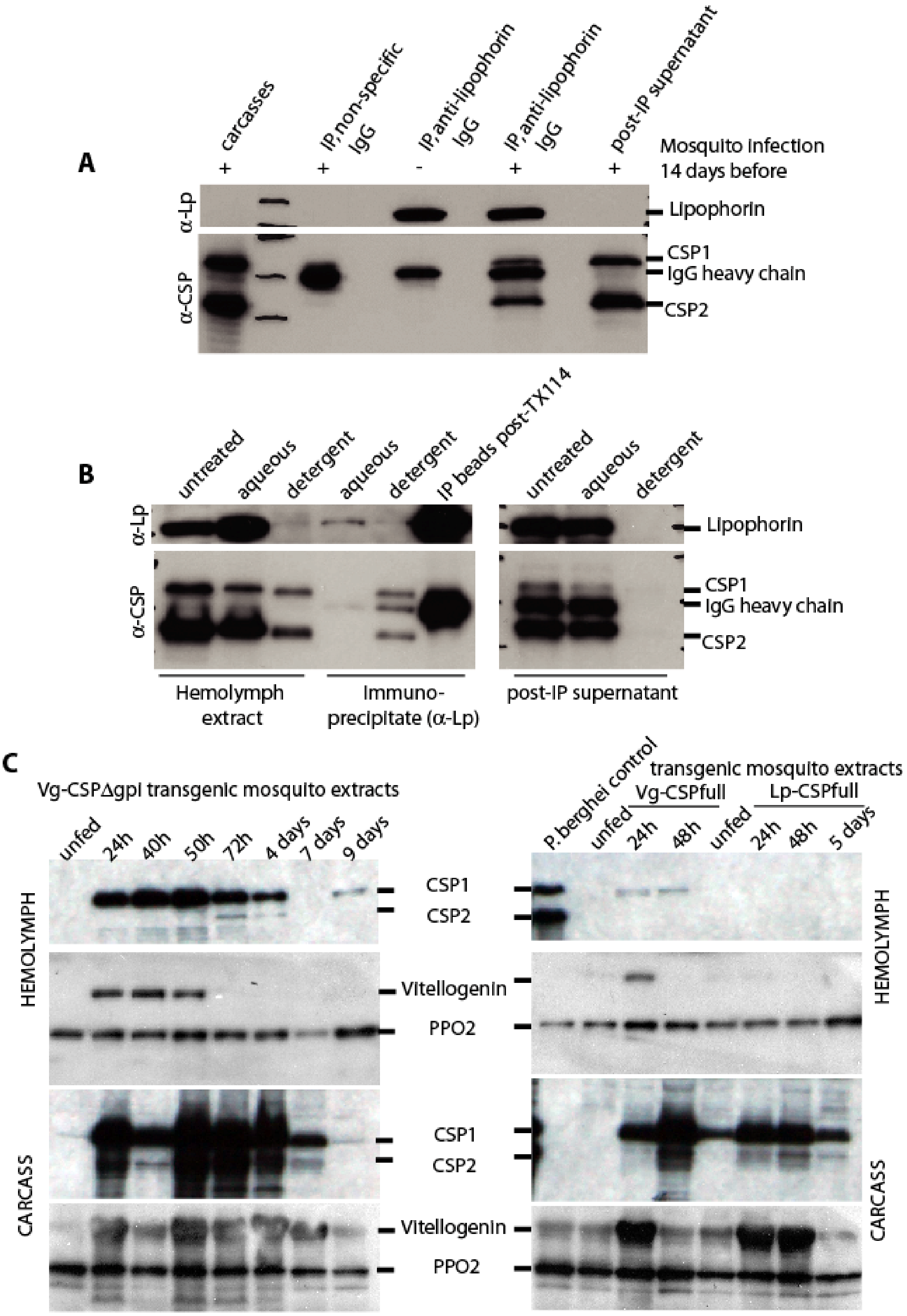
A
carcasses
IP, non-specific IgG
IP, anti-lipophorin IgG
IP, anti-lipophorin IgG
post-IP supernatant
Mosquito infection 14 days before
+ + - + +
α-Lp
α-CSP
Lipophorin
CSP1
IgG heavy chain
CSP2
B
untreated
aqueous
detergent
aqueous
detergent
IP beads post-TX114
untreated
aqueous
detergent
α-Lp
α-CSP
Lipophorin
CSP1
IgG heavy chain
CSP2
Hemolymph extract
Immuno-precipitate (α-Lp)
post-IP supernatant
C
Vg-CSPΔgpi transgenic mosquito extracts
unfed
24h
40h
50h
72h
4 days
7 days
9 days
P. berghei control
transgenic mosquito extracts
Vg-CSPfull
Lp-CSPfull
unfed
24h
48h
unfed
24h
48h
5 days
HEMOLYMPH
CARCASS
CSP1
CSP2
Vitellogenin
PPO2
CSP1
CSP2
Vitellogenin
PPO2

Figure S3 ***Plasmodium berghei*** **CSP in mosquitoes. A: CSP associates with lipophorin particles in mosquito hemolymph.** Twenty infected females were anaesthetized and bled in 350 µl buffer. The extract was centrifuged and subjected to lipophorin immunoprecipitation (see Methods). Western blot with anti-lipophorin antibody (top panel) or CSP antibody (bottom panel), note that the secondary antibody reveals the mouse primary antibody used in the IP (IgG heavy chain) in addition to the CSP signal. Some released CSP associates with Lipophorin particles, while a large fraction remains in lipophorin-depleted supernatant. **B: CSP is released both without and with a GPI anchor and GPI-linked released CSP associates with lipophorin.** Triton X-114 protein fractionation (see Methods) of raw mosquito hemolymph extracts, immunoprecipitated lipophorin captured on sepharose beads and post-immunoprecipitation supernatant. CSP present in hemolymph extracts partitions in both aqueous (no GPI anchor) and detergent (GPI anchor) phases. CSP present on immunoprecipitated lipophorin exclusively partitions in the detergent phase (GPI anchored). Most lipophorin protein bound to antibodies and sepharose beads remained attached to the beads throughout the detergent fractionation process. No GPI-linked CSP remains in the lipophorin-depleted post-IP supernatant. **C: Codon-optimized *CSP* is expressed in transgenic mosquitoes**. Western blot was performed on mosquito hemolymph and whole body (carcass) extracts prepared at the indicated times after blood feeding with antibodies recognizing CSP, Vitellogenin (to monitor *Vg* promoter activity) and PPO2 (used as a loading control). Note that although both CSPΔGPI (left panel) and full-length CSP (right panel) expressions are highly induced by the blood meal (in addition to constitutive expression under the control of the *Lp* promoter, right panel) in tissues (carcasses), only CSPΔGPI is released to high levels in hemolymph. Also note that CSP appears to be cleaved inefficiently in transgenic mosquitoes compared to the *P. berghei* control, suggesting that mosquitoes lack the protease that usually cleaves CSP. Antibodies to Lipophorin, Vitellogenin and PPO2 are described in Rono Rono *et al.* (2010) and Fraiture *et al.* (2009).

File S1

Docking lines

DNA sequence of *Anopheles gambiae att*P docking lines X1, X13, X6 and XK. *att*P sites are embedded in a small *piggyBac* transposon; sequence is provided in its genomic context with flanking segments from the mosquito chromosome.

X1 line (2L: 1802562)

```
LOCUS       X1 locus       12061 bp    DNA     circular     20-Jan-2010
FEATURES             Location/Qualifiers
     misc_feature    1193..1328
                     /note="PiggyBac 5'region"
     misc_feature    798..1018
                     /note="attP'"
     misc_feature    504..661
                     /note="PiggyBac 3'region"
     misc_feature    754..787
                     /note="loxP"
     misc_feature    1..503
                     /note="A. gambiae chromosome 2L"
     misc_feature    1329..1745
                     /note="A. gambiae chromosome 2L"

ORIGIN
        1 ctcaataaca tgcccgtcat gagttcaagc ctgaaataga ccgtcccccc gcagcaaaga
       61 tttgacttat ctggctgctt cgtaattaat taagtctcca aagcctgtat aggccggcat
      121 gtccgcgtag ggcgttacgc caaatagaag aagagaagac acgtatgtca taccctcgta
      181 tttatgtata gataaatatt cttaccttcc agaaaaaagt ttgtgtcatc atctgattct
      241 accgtggata caaagcttta tttatttctc ttttacaact acaatagtat ttttaatgat
      301 tctttaaaat agtgtatgta gtttcagact catcgcaaaa aaaaataaat aaagtttaac
      361 tgcttccagg agaacttgaa gctaccaata atttataggg aagattggaa tccatccatt
      421 ggacgatgat attatttgct ttaagaagag atatattacg gcaaacatta gtgaagataa
      481 caccAttttg tattttgttt taaccctaga aagataatca tattgtgacg tacgttaaag
      541 ataatcatgc gtaaaattga cgcatgtgtt ttatcggtct gtatatcgag gtttatttat
      601 taatttgaat agatattaag ttttattata tttacactta catactaata ataaattcaa
      661 caaacaattt atttatgttt atttatttat taaaaaaaac aaaaactcaa aatttcttct
      721 ataaagtaac aaaactttta tcgaattgag ctcataactt cgtataatgt atgctatacg
      781 aagttatggt acctgcagta ctgacggaca caccgaagcc ccggcggcaa ccctcagcgg
      841 atgccccggg gcttcacgtt ttcccaggtc agaagcggtt ttcgggagta gtgccccaac
      901 tggggtaacc tttgagttct ctcagttggg ggcgtagggt cgccgacatg acacaagggg
      961 ttgtgaccgg ggtggacacg tacgcgggtg cttacgaccg tcagtcgcgc gagcgcgatc
     1021 taatctataa caagaaaata tatatataat aagttatcac gtaagtagaa catgaaataa
     1081 caatataatt atcgtatgag ttaaatctta aaagtcacgt aaaagataat catgcgtcat
     1141 tttgactcac gcggtcgtta tagttcaaaa tcagtgacac ttaccgcatt gacaagcacg
     1201 cctcacggga gctccaagcg gcgactgaga tgtcctaaat gcacagcgac ggattcgcgc
     1261 tatttagaaa gagagagcaa tatttcaaga atgcatgcgt caattttacg cagactatct
     1321 ttctagggtt aagtAtcatg aatatgtggt aatGaTatta atCgaatgag cattAaggga
     1381 ccaaccataa attacgtaac aatatcagtg ggccaacgat attttagacc gaacaaatag
     1441 ataggagagt tctagactgt gTatcaaaac atgaacataa ttAaaaaaaa cttatttaat
     1501 ttctgttgga aaaCactttg gttaatcaag atagttttga aagtttattt caaaagtcta
     1561 agctatcacg atagttgtgc aattactttg tctctaaaag caacagtgtg aatgggttgc
     1621 agttgttctt gtttggatga gttattttca cctccttcgg cttatttctt tcttattttt
     1681 agttattgga tttacatatg tcgagggagc tggcgtttta aaggcgaaag aattgtaccg
     1741 gaatt
//
```

X13 line (2L: 2798380)

```
LOCUS       X13 locus       12772 bp    DNA     circular     20-Jan-2010
FEATURES             Location/Qualifiers
     misc_feature    1099..1319
                     /note= "attP'"
     misc_feature    805..962
                     /note="PiggyBac 3'region"
     misc_feature    1055..1088
                     /note="loxP"
     misc_feature    1494..1629
                     /note="PiggyBac 5' region"
     misc_feature    1630..2459
                     /note="A. gambiae chromosome 2L"
     misc_feature    1..804
                     /note="A. gambiae chromosome 2L"

ORIGIN
        1 gggtggacaa gtcaacgctt attggtatgc agtggtttcg ctcgttggtc aaacctcggt
       61 ttgacctcta cggaaagggc ggaaaggagg aaaagagcga aagcaacagg catgttgatg
      121 caccacacac gcactgacgc aagatgccac agggatggga aacttaactg gccaatattg
      181 acaattttcc gcttacgtgt gtaaacaatg agcgccgaga gtatattgat tttctatcga
      241 ttgtgcatcc ctttaacctg cacggactga ctttaaacga gtgggtgttt atcgaagacg
      301 agtgcgtaac gagtgtgaag gcagcatgct ggaagcaaag cgacgttctt ctaactatcc
      361 tcatcacatc aagcaatccg acacatctaa acggaaacat tttatggtgg gctctgtcat
      421 cgtgcatcgc cgctcgccaa atgtctatat tgatggctat aaacacataa agcaatcgtg
      481 gccgcttgcc ctcaccgggg ctccactctc agctctcgaa gcttttaccc ggcgcacaga
      541 cgaatggcgt atttatgcga ccaacgaatg gcccaaccct tggttggctg gtgttttatt
      601 ttactttttt ttgcgacgct tggcttcctg gatgcgagcg gagccgtaaa agtggtcgaa
      661 aatgaaaata caccccggaa atgccggagt ccacggagtc acgaaatcaa tttccaccag
      721 ctgaaggatg cttgggttcc tttttatgt gtttgcctgc agtcgttgcc ggtatttgac
      781 gcattgcccc tgggaggttt ttaaccctag aaagataatc atattgtgac gtacgttaaa
      841 gataatcatg cgtaaaattg acgcatgtgt tttatcggtc tgtatatcga ggtttattta
      901 ttaatttgaa tagatattaa gttttattat atttacactt acatactaat aataaattca
      961 acaaacaatt tatttatgtt tatttattta ttaaaaaaaa caaaaactca aaatttcttc
     1021 tataaagtaa caaaactttt atcgaattga gctcataact tcgtataatg tatgctatac
     1081 gaagttatgg tacctgcagt actgacggac acaccgaagc cccggcggca accctcagcg
     1141 gatgccccgg ggcttcacgt tttcccaggt cagaagcggt tttcgggagt agtgccccaa
     1201 ctggggtaac ctttgagttc tctcagttgg gggcgtaggg tcgccgacat gacacaaggg
     1261 gttgtgaccg gggtggacac gtacgcgggt gcttacgacc gtcagtcgcg cgagcgcgat
     1321 ctaatctata acaagaaaat atatatataa taagttatca cgtaagtaga acatgaaata
     1381 acaatataat tatcgtatga gttaaatctt aaaagtcacg taaaagataa tcatgcgtca
     1441 ttttgactca cgcggtcgtt atagttcaaa atcagtgaca cttaccgcat tgacaagcac
     1501 gcctcacggg agctccaagc ggcgactgag atgtcctaaa tgcacagcga cggattcgcg
     1561 ctatttagaa agagagagca atatttcaag aatgcatgcg tcaattttac gcagactatc
     1621 tttctagggt taagggggac tgggtggaag catttgtttt tgctcgcttc ttttctctcg
     1681 ttCTGTCTCT ATCTatctct gctttgtgcg tagggcgtag ggatgtatga aaaagcttaT
     1741 tgcgtgtgat ggagttacac aagtacgtac atattcacaa cgacctggag tggggttgat
     1801 aattttagat ttcgaggatg gttttgaagg ctttataatc cccatgagtg gggatgttct
     1861 gggcatgatg ttgtaattat tttattaagg gtttccgact gacatgtgtg ttaacagcga
     1921 gtgtggcggt gttttgtgcc aggcgcttaa atgaaatgca ggaaatattt tcaattggcg
     1981 aaacatccta tgctgctttt tattctgtag tatgaagaca attaaaattt taattcaatt
     2041 tcaattgcat gccttaatgc gctccaaaca atgaacggag ggcctcttta tttacttttg
     2101 tagaataatt actttaattt tgatttgcag ctactaacgc ttatgggcct tgttttgtac
     2161 atagtacatt ctacatttga atcacaaaga atactattat aatcattctt gtacataaaa
     2221 actgtgttaa aaaagaggtg tcaaagcgtc tttcctaatc acacatgaac tatcatgtat
     2281 taaagagttc atactttttt taaataattg aaacttattg cagttgattt tatcagtatt
     2341 gtttttttgat ataacagata agctcttgac ttaatctaca tattaaagaa cttatcatca
     2401 tttttctgac ttacctgaat gactatgact aatgtcactt gacaggtgaa agtggatgg
//
```

X6 line (3R: 53037011)

```
LOCUS       X6 locus               2127 bp    DNA     linear       12-MAY-2009
FEATURES             Location/Qualifiers
     misc_feature    691..724
                     /note="loxP"
     misc_feature    735..955
                     /note="attP'"
     misc_feature    complement(1130..1265)
                     /note="PiggyBac 5' region"
     misc_feature    complement(441..598)
                     /note="PiggyBac 3'region"
     misc_feature    1..440
                     /note="A. gambiae chromosome 3R"
     misc_feature    1266..2127
                     /note="A. gambiae chromosome 3R"
     source          1..2127
                     /dnas_title="X6"
ORIGIN
        1 GCAAAACGGA AGGAGGAAAT GTAACAAAGA ATAGGTTTCA ACTAGGCATG GGCAAAAGAA
       61 TTCTTTTCAC CGATCGCGAG CGTACTAGTT CGCTCAGTAA AAAGAACCGA ACGCGAACGG
      121 CGATTCTATC GTTAATTTTT TAATCAATAT GACCCAGCCA TATCCCTAGC AGGATTTTCT
      181 ATTAACAAAT TATTTATTCG TTATTTAGCC TGCATTAATA ATTTGCATTA CCCATGATTG
      241 TGCATAGTAA AGTTGCAGAA GTGTATACAA CCACGCGGTT GAACCAAAAG CAAACAAGAA
      301 ATTTAACTAA CAAACCGCGT TGTCTTATTT CCAATTTTAC AATATATTTA CATACTAGTT
      361 GATATCATAG AGTGTGCCTG GCAAAATGTA TCATTCATTT AGTTGTGTTT GAAAATTATT
      421 TCATAGTTCA TTATGtTTAA CCCTAGAAAG ATAATCATAT TGTGACGTAC GTTAAAGATA
      481 ATCATGCGTA AAATTGACGC ATGTGTTTTA TCGGTCTGTA TATCGAGGTT TATTTATTAA
      541 TTTGAATAGA TATTAAGTTT TATTATATTT ACACTTACAT ACTAATAATA AATTCAACAA
      601 ACAATTTATT TATGTTTATT TATTTATTAA AAAAAACAAA AACTCAAAAT TTCTTCTATA
      661 AAGTAACAAA ACTTTTATCG AATTGAGCTC ataacttcgt ataatgtatg ctatacgaag
      721 ttatGGTACC TGCAGTACTG ACGGACACAC CGAAGCCCCG GCGGCAACCC TCAGCGGATG
      781 CCCCGGGGCT TCACGTTTTC CCAGGTCAGA AGCGGTTTTC GGGAGTAGTG CCCCAACTGG
      841 GGTAACCTTT GAGTTCTCTC AGTTGGGGGC GTAGGGTCGC CGACATGACA CAAGGGGTTG
      901 TGACCGGGGT GGACACGTAC GCGGGTGCTT ACGACCGTCA GTCGCGCGAG CGCGATCTAA
      961 TCTATAACAA GAAAATATAT ATATAATAAG TTATCACGTA AGTAGAACAT GAAATAACAA
     1021 TATAATTATC GTATGAGTTA AATCTTAAAA GTCACGTAAA AGATAATCAT GCGTCATTTT
     1081 GACTCACGCG GTCGTTATAG TTCAAAATCA GTGACACTTA CCGCATTGAC AAGCACGCCT
     1141 CACGGGAGCT CCAAGCGGCG ACTGAGATGT CCTAAATGCA CAGCGACGGA TTCGCGCTAT
     1201 TTAGAAAGAG AGAGCAATAT TTCAAGAATG CATGCGTCAA TTTTACGCAG ACTATCTTTC
     1261 TAGGGTTAAC AATTATTAAC TCTTcACAAA ATTTAAAAAA AAAGCTCAAC TATACCTTGA
     1321 CTGTCTTACT AAGAGAAAAA ATCAAAAGAA TATATTATGA ATTGTGTTGT AAGAAAACTA
     1381 ATTCGTGTAG TTTTTTAGGT AGTAATCTTG TTCGTTTTTC TGAAAATATC TAGCCTGTTT
     1441 TGGAGAAAAC TCTCTCAAGA GGGTTTTCCA TTGCTAAAAT GTACAATGTT GGATATTTCA
     1501 TACGATTTTC TTGCCACCAT GATAGAGGAT CTCGTTCCAA TGCAATTGGA TTTACCTTAA
     1561 GGTACAAATT AATTTCGGCT TCCGCAAGTT CCCTAAGAGT ATAAGATGTT TCCAATTCAG
     1621 AATGACAGAC TATGTCGCCA TATAATAAGT TTGCATCAGT ATTAGATTTG AAAGCATCCT
     1681 CTCTTACATT ACATCCTCTT ATATTTGTTT GCTCTCCTGA AGGGGAAGCA TATCCGTTAG
     1741 TATCAAATCA TGTGTTTTTT TTAGTTTATC GGGTTCATTC TGAAACCCCG CTTTCATCAC
     1801 ACGTGGATCT AGGAACATTG CTTTCCAAAT TTGATGGTTA TCTCTATACA ATTTAAGTTT
     1861 TTCCTCTGTG TTTTCAACTA GTAAAGATAT CAAAGTCTTT ACTTCTGGGA CTAATTCGTT
     1921 GTTCAATAGC AAAGTTGAAG TTTTACGCAA TAATACATTA GCTAACGTGT GAAAGGGTCG
     1981 TATGTTGTTC CGCCGAAACT AATTTAGTTT CTGATGCAAA ATATTTGAGT GCCTCCAATC
     2041 ATTGCTCGAT AATTACCCAA TCAGTGGGTG ACAAGTTGGT TTTCATTTTT AAGCTATCTA
     2101 CGCACTAAAG AATGGGTATT TTATTTT
//
```

XK line (X: 22463468)

```
LOCUS       XK                      1666 bp    DNA     linear
```

```
FEATURES             Location/Qualifiers
     misc_feature    1248..1383
                     /note="PiggyBac 5'TR"
     misc_feature    853..1073
                     /note="attP'"
     misc_feature    809..842
                     /note="loxP"
     misc_feature    1..558
                     /note="A. gambiae X chromosome"
     misc_feature    559..804
                     /note="PiggyBac 3' region"
     misc_feature    1384..1666
                     /note="A. gambiae X chromosome"
     source          1..1666
                     /dnas_title="XK"
ORIGIN
        1 TATCTCGAAT TTCTGCGTAG GTCGAGCCTC TTAATTTTTA GCCAAAATAT CACTAATTTC
       61 GTTTAATTGT GCAAGGGGGT GGTTTTAGCC ACGTAATCTA TTATTTGATA TGAGTCTGAT
      121 TAAATTTAAC AGTTTTAATC CTGTTGATTT GGTTTTAAAC ATTCGATCAA AAAGGTAATT
      181 ATTTTGCATT TGACAAAAAT TGATGAGTCA AATCAGTACA ATTTGCTCAA AGAACTGTCA
      241 TATTTAGAAT CTCCTATTCC GCGTATTAGA GGTACCGTGT ATCTCAGGGA CTTCCTGTAC
      301 TTTTGTATCT TGTTGTTTGT TACACGGAAT GATTTTTCCT GAGTGCACCA TGAATTATGC
      361 TGCTACTCGC ACGACGCTGA TGATGCACCT CACGAATAGA TACAAGAGAT TTCGGGAGTT
      421 TTCTTAATTT TGGGACACTT TCTTGATTGT GTTTTACGAG TAGTGAGAAC TTTATGTGAT
      481 AAGAAGTGAA GTCTAAGTGT TGAAAAACGC GGTGGATTTT TTAGAGAAAA CACTTTATTT
      541 ACTGTGGTAT GAGGTTAAcc ctagaaagat aatcatattg tgacgtacgt taaagataat
      601 catgcgtaaa attgacgcat gtgttttatc ggtctgtata tcgaggttta tttattaatt
      661 tgaatagata ttaagtttta ttatatttac acttacatac taataataaa ttcaacaaac
      721 aatttattta tgtttattta tttattaaaa aaaacaaaaa ctcaaaattt cttctataaa
      781 gtaacaaaac ttttatcgaa ttgagctcat aacttcgtat aatgtatgct atacgaagtt
      841 atggtacctg cagtactgac ggacacaccg aagccccggc ggcaacccctc agcggatgcc
      901 ccggggcttc acgttttccc aggtcagaag cggttttcgg gagtagtgcc ccaactgggg
      961 taacctttga gttctctcag ttgggggcgt agggtcgccg acatgacaca aggggttgtg
     1021 accggggtgg acacgtacgc gggtgcttac gaccgtcagt cgcgcgagcg cgatctaatc
     1081 tataacaaga aaatatatat ataataagtt atcacgtaag tagaacatga aataacaata
     1141 taattatcgt atgagttaaa tcttaaaagt cacgtaaaag ataatcatgc gtcattttga
     1201 ctcacgcggt cgttatagtt caaaatcagt gacacttacc gcattgacaa gcacgcctca
     1261 cgggagctcc aagcggcgac tgagatgtcc taaatgcaca gcgacggatt cgcgctattt
     1321 agaaagagag agcaatattt caagaatgca tgcgtcaatt ttacgcagac tatctttcta
     1381 gggTTAAATC CAAAAATTAG TCCACTTGTA CCGATTGTCG AATTTCAACT AACTGGTTAG
     1441 GAGCGGACCA ACCCTTCTCC TTATCTCCTA ATTTACCGTT TTTATCCTCC CTCTCACTCT
     1501 CTTCTCTAGC GTTGTCACCT GACAGACTGT TGGGCACTCA CGAGTTTCCA ACACTAAGAA
     1561 TAAGAAGTCT ACGGCACCCT TTTTGAAACG GCTTTTATAT ATTTACTCCA GCGTAGAAAC
     1621 ACACGCTCTT GGATTCTTTT TCACCCATAC CACTCCCATT CCCGGG
//
```

File S2

DNA sequence of transgenesis vectors

Note: the pDSAx vector series carries kanamycin resistance.

pDSAG (GFP):

```
LOCUS       pDSAG                   5143 bp    DNA     circular
FEATURES             Location/Qualifiers
     misc_feature    complement(3258..3353)
                     /note="attL2"
     misc_feature    complement(3404..3422)
                     /note="M13R"
     misc_feature    1972..2021
                     /note="3xP3 element"
     misc_feature    2061..2068
                     /note="TATA"
                     /note="attR4"
     misc_feature    271..286
                     /note="M13F"
     misc_feature    600..1174
                     /note="LacZ"
     misc_feature    479..599
                     /note="lac promoter"
     misc_feature    590..608
                     /note="M13R"
     misc_feature    625..644
                     /note="T3"
     misc_feature    677..696
                     /note="SK primer"
     misc_feature    complement(731..747)
                     /note="KS primer"
     misc_feature    complement(800..816)
                     /note="M13F"
     misc_feature    complement(771..790)
                     /note="T7"
     misc_feature    1395..1621
                     /note="SV40 term"
     misc_feature    1622..1898
                     /note="attB"
     misc_feature    2965..3194
                     /note="SV40 term"
     misc_feature    2232..2951
                     /note="GFP"
     source          1..5143
                     /dnas_title="pDSAG"
ORIGIN
        1 CTTTCCTGCG TTATCCCCTG ATTCTGTGGA TAACCGTATT ACCGCCTTTG AGTGAGCTGA
       61 TACCGCTCGC CGCAGCCGAA CGACCGAGCG CAGCGAGTCA GTGAGCGAGG AAGCGGAAGA
      121 GCGCCCAATA CGCAAACCGC CTCTCCCCGC GCGTTGGCCG ATTCATTAAT GCAGCTGGCA
      181 CGACAGGTTT CCCGACTGGA AAGCGGGCAG TGAGCGCAAC GCAATTAATA CGCGTACCGC
      241 TAGCATGGAT GTTTTCCCAG TCACGACGTT GTAAAACGAC GGCCAGTCTT AAGCTCGGGC
      301 CCCTACAGGT CACTAATACC ATCTAAGTAG TTGATTCATA GTGACTGGAT ATGTTGTGTT
      361 TTACAGTATT ATGTAGTCTG TTTTTTATGC AAAATCTAAT TTAATATATT GATATTTATA
      421 TCATTTTACG TTTCTCGTTC AACTTTTCTA TACAAAGTTg gtACcggatc cagagaccCG
      481 CAACGCAATT AATGTGAGTT AGCTCACTCA TTAGGCACCC CAGGCTTTAC ACTTTATGCT
      541 TCCGGCTCGT ATGTTGTGTG GAATTGTGAG CGGATAACAA TTTCACACAG GAAACAGCTA
      601 TGACCATGAT TACGCCAAGC GCGCAATTAA CCCTCACTAA AGGGAACAAA AGCTGGAGCT
      661 CCACCGCGGT GGCGGCCGCT CTAGAACTAG TGGATCCCCC GGGCTGCAGG AATTCGATAT
      721 CAAGCTTATC GATACCGTCG ACCTCGAGGG GGGGCCCGGT ACCCAATTCG CCCTATAGTG
      781 AGTCGTATTA CGCGCGCTCA CTGGCCGTCG TTTTACAACG TCGTGACTGG GAAAACCCTG
```

841 GCGTTACCCA ACTTAATCGC CTTGCAGCAC ATCCCCCTTT CGCCAGCTGG CGTAATAGCG
901 AAGAGGCCCG CACCGATCGC CCTTCCCAAC AGTTGCGCAG CCTGAATGGC GAATGGGACG
961 CGCCCTGTAG CGGCGCATTA AGCGCGGCGG GTGTGGTGGT TACGCGCAGC GTGACCGCTA
1021 CACTTGCCAG CGCCCTAGCG CCCGCTCCTT TCGCTTTCTT CCCTTCCTTT CTCGCCACGT
1081 TCGCCGGCTT TCCCCGTCAA GCTCTAAATC GGGGGCTCCC TTTAGGGTTC CGATTTAGTG
1141 CTTTACGGCA CCTCGACCCC AAAAAACTTG ATTAGGGTGA TGGTTCACGT AGTGGGCCAT
1201 CGCCCTGATA GACGGTTTTT CGCCCTTTGA CGTTGGAGTC CACGTTCTTT AATAGTGGAC
1261 TCTTGTTCCA AACTGGAACA ACACTCAACC CTATCTCGGT CTATTCTTTT GATTTATAAG
1321 GGATTTTGCC GATTTCGGCC TATTGGTTAA AAAATGAGCT GATTTAACAA AAATTTAACG
1381 CGggtctccg cttCTAGACA TAATCAGCCA TACCACATTT GTAGAGGTTT TACTTGCTTT
1441 AAAAAACCTC CCACACCTCC CCCTGAACCT GAAACATAAA ATGAATGCAA TTGTTGTTGT
1501 TAACTTGTTT ATTGCAGCTT ATAATGGTTA CAAATAAAGC AATAGCATCA CAAATTTCAC
1561 AAATAAAGCA TTTTTTCTTCA CTGCATTCTA GTTGTGGTTT GTCCAAACTC ATCAATGTAT
1621 CtcgaCGATG TAGGTCACaG TCTCGAAGCC GCGGTGCGGG TGCCAGGGCG TGCCCTTGGG
1681 CTCCCCGGGC GCGTACTCCA CCTCACCCAT CTGGTCCATC ATGATGAACG GGTCGAGGTG
1741 GCGGTAGTTG ATCCCGGCGA ACGCGCGGCG CACCGGGAAG CCCTCGCCCT CGAAACCGCT
1801 GGGCGCGGTG GTCACGGTGA GCACGGGACG TGCGACGGCG TCGGCGGGTG CGGATACGCG
1861 GGGCAGCGTC AGCGGGTTCT CGACGGTCAC GGCGGGCAat tCCTGCAGAC TTCCGGTATC
1921 TCGCGTTTGT TTGATCGCAC GGTTCCCACA ATGGTTAATT CGAGCTCGCC CGGGGATCTA
1981 ATTCAATTAG AGACTAATTC AATTAGAGCT AATTCAATTA GGATCCAAGC TTATCGATTT
2041 CGAACCCTCG ACCGCCGGAG TATAAATAGA GGCGCTTCGT CTACGGAGCG ACAATTCAAT
2101 TCAAACAAGC AAAGTGAACA CGTCGCTAAG CGAAAGCTAA GCAAATAAAC AAGCGCAGCT
2161 GAACAAGCTA AACAATCGGG GTACCGCTAG AGTCGACGGT ACCGCGGGCC CGGGATCCAC
2221 CGGTCGCCAC CATGGTGAGC AAGGGCGAGG AGCTGTTCAC CGGGGTGGTG CCCATCCTGG
2281 TCGAGCTGGA CGGCGACGTA AACGGCCACA AGTTCAGCGT GTCCGGCGAG GGCGAGGGCG
2341 ATGCCACCTA CGGCAAGCTG ACCCTGAAGT TCATCTGCAC CACCGGCAAG CTGCCCGTGC
2401 CCTGGCCCAC CCTCGTGACC ACCCTGACCT ACGGCGTGCA GTGCTTCAGC CGCTACCCCG
2461 ACCACATGAA GCAGCACGAC TTCTTCAAGT CCGCCATGCC CGAAGGCTAC GTCCAGGAGC
2521 GCACCATCTT CTTCAAGGAC GACGGCAACT ACAAGACCCG CGCCGAGGTG AAGTTCGAGG
2581 GCGACACCCT GGTGAACCGC ATCGAGCTGA AGGGCATCGA CTTCAAGGAG GACGGCAACA
2641 TCCTGGGGCA CAAGCTGGAG TACAACTACA ACAGCCACAA CGTCTATATC ATGGCCGACA
2701 AGCAGAAGAA CGGCATCAAG GTGAACTTCA AGATCCGCCA CAACATCGAG GACGGCAGCG
2761 TGCAGCTCGC CGACCACTAC CAGCAGAACA CCCCCATCGG CGACGGCCCC GTGCTGCTGC
2821 CCGACAACCA CTACCTGAGC ACCCAGTCCG CCCTGAGCAA AGACCCCAAC GAGAAGCGCG
2881 ATCACATGGT CCTGCTGGAG TTCGTGACCG CCGCCGGGAT CACTCTCGGC ATGGACGAGC
2941 TGTACAAGTA AAGCGGCCGC GACTctagat caAATCAGCC ATACCACATT TGTAGAGGTT
3001 TTACTTGCTT TAAAAAACCT CCCACACCTC CCCCTGAACC TGAAACATAA AATGAATGCA
3061 ATTGTTGTTG TTAACTTGTT TATTGCAGCT TATAATGGTT ACAAATAAAG CAATAGCATC
3121 ACAAATTTCA CAAATAAAGC ATTTTTTTCA CTGCATTCTA GTTGTGGTTT GTCCAAACTC
3181 ATCAATGTAT CTTAAAGCTT ATCGATACGC GTACGGCGCG CCTAGAGCGG CCGCCACCGC
3241 GGTGGAGCTC GAGTACCCAG CTTTCTTGTA CAAAGTTGGC ATTATAAGAA AGCATTGCTT
3301 ATCAATTTGT TGCAACGAAC AGGTCACTAT CAGTCAAAAT AAAATCATTA TTTGCCATCC
3361 AGCTGCAGGG CGGCCGCGAT ATCCCCTATA GTGAGTCGTA TTACATGGTC ATAGCTGTTT
3421 CCTGGCAGCT CTGGCCCGTG TCTCAAAATC TCTGATGTTA CATTGCACAA GATAAAAATA
3481 TATCATCATG AACAATAAAA CTGTCTGCTT ACATAAACAG TAATACAAGG GGTGTTATGA
3541 GCCATATTCA ACGGGAAACG TCGAGGCCGC GATTAAATTC CAACATGGAT GCTGATTTAT
3601 ATGGGTATAA ATGGGCTCGC GATAATGTCG GGCAATCAGG TGCGACAATC TATCGCTTGT
3661 ATGGGAAGCC CGATGCGCCA GAGTTGTTTC TGAAACATGG CAAAGGTAGC GTTGCCAATG
3721 ATGTTACAGA TGAGATGGTC AGACTAAACT GGCTGACGGA ATTTATGCCT CTTCCGACCA
3781 TCAAGCATTT TATCCGTACT CCTGATGATG CATGGTTACT CACCACTGCG ATCCCCGGAA
3841 AAACAGCATT CCAGGTATTA GAAGAATATC CTGATTCAGG TGAAAATATT GTTGATGCGC
3901 TGGCAGTGTT CCTGCGCCGG TTGCATTCGA TTCCTGTTTG TAATTGTCCT TTTAACAGCG
3961 ATCGCGTATT TCGTCTCGCT CAGGCGCAAT CACGAATGAA TAACGGTTTG GTTGATGCGA
4021 GTGATTTTGA TGACGAGCGT AATGGCTGGC CTGTTGAACA AGTCTGGAAA GAAATGCATA
4081 AACTTTTGCC ATTCTCACCG GATTCAGTCG TCACTCATGG TGATTTCTCA CTTGATAACC
4141 TTATTTTTGA CGAGGGGAAA TTAATAGGTT GTATTGATGT TGGACGAGTC GGAATCGCAG
4201 ACCGATACCA GGATCTTGCC ATCCTATGGA ACTGCCTCGG TGAGTTTTCT CCTTCATTAC
4261 AGAAACGGCT TTTTCAAAAA TATGGTATTG ATAATCCTGA TATGAATAAA TTGCAGTTTC
4321 ATTTGATGCT CGATGAGTTT TTCTAATCAG AATTGGTTAA TTGGTTGTAA CACTGGCAGA
4381 GCATTACGCT GACTTGACGG GACGGCGCAA GCTCATGACC AAAATCCCTT AACGTGAGTT
4441 ACGCGTCGTT CCACTGAGCG TCAGACCCCG TAGAAAAGAT CAAAGGATCT TCTTGAGATC
4501 CTTTTTTTCT GCGCGTAATC TGCTGCTTGC AAACAAAAAA ACCACCGCTA CCAGCGGTGG
4561 TTTGTTTGCC GGATCAAGAG CTACCAACTC TTTTTCCGAA GGTAACTGGC TTCAGCAGAG
4621 CGCAGATACC AAATACTGTT CTTCTAGTGT AGCCGTAGTT AGGCCACCAC TTCAAGAACT
4681 CTGTAGCACC GCCTACATAC CTCGCTCTGC TAATCCTGTT ACCAGTGGCT GCTGCCAGTG

```
     4741 GCGATAAGTC GTGTCTTACC GGGTTGGACT CAAGACGATA GTTACCGGAT AAGGCGCAGC
     4801 GGTCGGGCTG AACGGGGGGT TCGTGCACAC AGCCCAGCTT GGAGCGAACG ACCTACACCG
     4861 AACTGAGATA CCTACAGCGT GAGCTATGAG AAAGCGCCAC GCTTCCCGAA GGGAGAAAGG
     4921 CGGACAGGTA TCCGGTAAGC GGCAGGGTCG GAACAGGAGA GCGCACGAGG GAGCTTCCAG
     4981 GGGGAAACGC CTGGTATCTT TATAGTCCTG TCGGGTTTCG CCACCTCTGA CTTGAGCGTC
     5041 GATTTTTGTG ATGCTCGTCA GGGGGGCGGA GCCTATGGAA AAACGCCAGC AACGCGGCCT
     5101 TTTTACGGTT CCTGGCCTTT TGCTGGCCTT TTGCTCACAT GTT
//
```

<u>pDSAT (mTurquoise2):</u>

```
LOCUS       pDSAT      5147 bp    DNA     circular
FEATURES             Location/Qualifiers

     misc_feature    complement(3408..3426)
                     /note="M13R"
     misc_feature    1972..2021
                     /note="3x Pax6 binding sites"
     misc_feature    2061..2068
                     /note="TATA"
     misc_feature    271..286
                     /note="M13F"
     misc_feature    600..1174
                     /note="LacZ"
     misc_feature    479..599
                     /note="lac promoter"
     misc_feature    590..608
                     /note="M13R"
     misc_feature    625..644
                     /note="T3"
     misc_feature    677..696
                     /note="SK primer"
     misc_feature    complement(731..747)
                     /note="KS primer"
     misc_feature    complement(800..816)
                     /note="M13F"
     misc_feature    complement(771..790)
                     /note="T7"
     misc_feature    1395..1621
                     /note="SV40 term"
     misc_feature    1622..1898
                     /note="attB"
     misc_feature    2977..3198
                     /note="SV40 term"
     misc_feature    2232..2948
                     /note="mTurquoise2"
     misc_feature    complement(3452..3470)
                     /note="DONR-RP"

ORIGIN
        1 CTTTCCTGCG TTATCCCCTG ATTCTGTGGA TAACCGTATT ACCGCCTTTG AGTGAGCTGA
       61 TACCGCTCGC CGCAGCCGAA CGACCGAGCG CAGCGAGTCA GTGAGCGAGG AAGCGGAAGA
      121 GCGCCCAATA CGCAAACCGC CTCTCCCCGC GCGTTGGCCG ATTCATTAAT GCAGCTGGCA
      181 CGACAGGTTT CCCGACTGGA AAGCGGGCAG TGAGCGCAAC GCAATTAATA CGCGTACCGC
      241 TAGCATGGAT GTTTTCCCAG TCACGACGTT GTAAAACGAC GGCCAGTCTT AAGCTCGGGC
      301 CCCTACAGGT CACTAATACC ATCTAAGTAG TTGATTCATA GTGACTGGAT ATGTTGTGTT
      361 TTACAGTATT ATGTAGTCTG TTTTTTATGC AAAATCTAAT TTAATATATT GATATTTATA
      421 TCATTTTACG TTTCTCGTTC AACTTTTCTA TACAAAGTTg gtACcggatc cagagaccCG
      481 CAACGCAATT AATGTGAGTT AGCTCACTCA TTAGGCACCC CAGGCTTTAC ACTTTATGCT
      541 TCCGGCTCGT ATGTTGTGTG GAATTGTGAG CGGATAACAA TTTCACACAG GAAACAGCTA
      601 TGACCATGAT TACGCCAAGC GCGCAATTAA CCCTCACTAA AGGGAACAAA AGCTGGAGCT
```

```
 661 CCACCGCGGT GGCGGCCGCT CTAGAACTAG TGGATCCCCC GGGCTGCAGG AATTCGATAT
 721 CAAGCTTATC GATACCGTCG ACCTCGAGGG GGGGCCCGGT ACCCAATTCG CCCTATAGTG
 781 AGTCGTATTA CGCGCGCTCA CTGGCCGTCG TTTTACAACG TCGTGACTGG GAAAACCCTG
 841 GCGTTACCCA ACTTAATCGC CTTGCAGCAC ATCCCCCTTT CGCCAGCTGG CGTAATAGCG
 901 AAGAGGCCCG CACCGATCGC CCTTCCCAAC AGTTGCGCAG CCTGAATGGC GAATGGACG
 961 CGCCCTGTAG CGGCGCATTA AGCGCGGCGG GTGTGGTGGT TACGCGCAGC GTGACCGCTA
1021 CACTTGCCAG CGCCCTAGCG CCCGCTCCTT TCGCTTTCTT CCCTTCCTTT CTCGCCACGT
1081 TCGCCGGCTT TCCCCGTCAA GCTCTAAATC GGGGGCTCCC TTTAGGGTTC CGATTTAGTG
1141 CTTTACGGCA CCTCGACCCC AAAAAACTTG ATTAGGGTGA TGGTTCACGT AGTGGGCCAT
1201 CGCCCTGATA GACGGTTTTT CGCCCTTTGA CGTTGGAGTC CACGTTCTTT AATAGTGGAC
1261 TCTTGTTCCA AACTGGAACA ACACTCAACC CTATCTCGGT CTATTCTTTT GATTTATAAG
1321 GGATTTTGCC GATTTCGGCC TATTGGTTAA AAAATGAGCT GATTTAACAA AAATTTAACG
1381 CGggtctccg cttCTAGACA TAATCAGCCA TACCACATTT GTAGAGGTTT TACTTGCTTT
1441 AAAAAACCTC CCACACCTCC CCCTGAACCT GAAACATAAA ATGAATGCAA TTGTTGTTGT
1501 TAACTTGTTT ATTGCAGCTT ATAATGGTTA CAAATAAAGC AATAGCATCA CAAATTTCAC
1561 AAATAAAGCA TTTTTTCTTCA CTGCATTCTA GTTGTGGTTT GTCCAAACTC ATCAATGTAT
1621 CtcgaCGATG TAGGTCACaG TCTCGAAGCC GCGGTGCGGG TGCCAGGGCG TGCCCTTGGG
1681 CTCCCCGGGC GCGTACTCCA CCTCACCCAT CTGGTCCATC ATGATGAACG GGTCGAGGTG
1741 GCGGTAGTTG ATCCCGGCGA ACGCGCGGCG CACCGGGAAG CCCTCGCCCT CGAAACCGCT
1801 GGGCGCGGTG GTCACGGTGA GCACGGGACG TGCGACGGCG TCGGCGGGTG CGGATACGCG
1861 GGGCAGCGTC AGCGGGTTCT CGACGGTCAC GGCGGGCAat tCCTGCAGAC TTCCGGTATC
1921 TCGCGTTTGT TTGATCGCAC GGTTCCCACA ATGGTTAATT CGAGCTCGCC CGGGGATCTA
1981 ATTCAATTAG AGACTAATTC AATTAGAGCT AATTCAATTA GGATCCAAGC TTATCGATTT
2041 CGAACCCTCG ACCGCCGGAG TATAAATAGA GGCGCTTCGT CTACGGAGCG ACAATTCAAT
2101 TCAAACAAGC AAAGTGAACA CGTCGCTAAG CGAAAGCTAA GCAAATAAAC AAGCGCAGCT
2161 GAACAAGCTA AACAATCGGG GTACCGCTAG AGTCGACGGT ACCGCGGGCC CGGGATCCAC
2221 CGGTCGCCAC catggtgagc aagggcgagg agctgttcac cggggtggtg cccatcctgg
2281 tcgagctgga cggcgacgta aacggccaca agttcagcgt gtccggcgag ggcgagggcg
2341 atgccaccta cGGCAAGCTG ACCCTGAAGT TCatctgcac caccggcaag ctgcccgtgc
2401 cctggcccac cctcgtgacc accctgtcct ggggcgtgca gtgcttcgcc cgctaccccg
2461 accacatgaa gcagcacgac ttcttcaagt ccgccatgcc cgaaggctac gtccaggagc
2521 gcaccatctt cttcaaggac gacggcaact acaagacccg cgccgaggtg aagttcgagg
2581 gcgacaccct ggtgaaccgc atcgagctga agggcatcga cttcaaggag gacggcaaca
2641 tcctggggca caagctggag tacaactacT tTagcgacaa cgtctatatc accgccgaca
2701 agcagaagaa cggcatcaag gccaacttca agatccgcca caacatcgag gacggcggcg
2761 tgcagctcgc cgaccactac cagcagaaca cccccatcgg cgacggcccc gtgctgctgc
2821 ccgacaacca ctacctgagc acccagtcca agctgagcaa agaccccaac gagaagcgcg
2881 atcacatggt cctgctggag ttcgtgaccg ccgccgggat cactctcggc atggacgagc
2941 tgtacaagtc cggaTGAtag atctgacggg tgatcaAATC AGCCATACCA CATTTGTAGA
3001 GGTTTTACTT GCTTTAAAAA ACCTCCCACA CCTCCCCCTG AACCTGAAAC ATAAAATGAA
3061 TGCAATTGTT GTTGTTAACT TGTTTATTGC AGCTTATAAT GGTTACAAAT AAAGCAATAG
3121 CATCACAAAT TTCACAAATA AAGCATTTTT TTCACTGCAT TCTAGTTGTG GTTTGTCCAA
3181 ACTCATCAAT GTATCTTAAA GCTTATCGAT ACGCGTACGG CGCGCCTAGA GCGGCCGCCA
3241 CCGCGGTGGA GCTCGAGTAC CCAGCTTTCT TGTACAAAGT TGGCATTATA AGAAAGCATT
3301 GCTTATCAAT TTGTTGCAAC GAACAGGTCA CTATCAGTCA AAATAAAATC ATTATTTGCC
3361 ATCCAGCTGC AGGGCGGCCG CGATATCCCC TATAGTGAGT CGTATTACAT GGTCATAGCT
3421 GTTTCCTGGC AGCTCTGGCC CGTGTCTCAA AATCTCTGAT GTTACATTGC ACAAGATAAA
3481 AATATATCAT CATGAACAAT AAAACTGTCT GCTTACATAA ACAGTAATAC AAGGGGTGTT
3541 ATGAGCCATA TTCAACGGGA AACGTCGAGG CCGCGATTAA ATTCCAACAT GGATGCTGAT
3601 TTATATGGGT ATAAATGGGC TCGCGATAAT GTCGGGCAAT CAGGTGCGAC AATCTATCGC
3661 TTGTATGGGA AGCCCGATGC GCCAGAGTTG TTTCTGAAAC ATGGCAAAGG TAGCGTTGCC
3721 AATGATGTTA CAGATGAGAT GGTCAGACTA AACTGGCTGA CGGAATTTAT GCCTCTTCCG
3781 ACCATCAAGC ATTTTATCCG TACTCCTGAT GATGCATGGT TACTCACCAC TGCGATCCCC
3841 GGAAAAACAG CATTCCAGGT ATTAGAAGAA TATCCTGATT CAGGTGAAAA TATTGTTGAT
3901 GCGCTGGCAG TGTTCCTGCG CCGGTTGCAT TCGATTCCTG TTTGTAATTG TCCTTTTAAC
3961 AGCGATCGCG TATTTCGTCT CGCTCAGGCG CAATCACGAA TGAATAACGG TTTGGTTGAT
4021 GCGAGTGATT TTGATGACGA GCGTAATGGC TGGCCTGTTG AACAAGTCTG GAAAGAAATG
4081 CATAAACTTT TGCCATTCTC ACCGGATTCA GTCGTCACTC ATGGTGATTT CTCACTTGAT
4141 AACCTTATTT TTGACGAGGG GAAATTAATA GGTTGTATTG ATGTTGGACG AGTCGGAATC
4201 GCAGACCGAT ACCAGGATCT TGCCATCCTA TGGAACTGCC TCGGTGAGTT TTCTCCTTCA
4261 TTACAGAAAC GGCTTTTTCA AAAATATGGT ATTGATAATC CTGATATGAA TAAATTGCAG
4321 TTTCATTTGA TGCTCGATGA GTTTTTCTAA TCAGAATTGG TTAATTGGTT GTAACACTGG
4381 CAGAGCATTA CGCTGACTTG ACGGGACGGC GCAAGCTCAT GACCAAAATC CCTTAACGTG
4441 AGTTACGCGT CGTTCCACTG AGCGTCAGAC CCCGTAGAAA AGATCAAAGG ATCTTCTTGA
4501 GATCCTTTTT TTCTGCGCGT AATCTGCTGC TTGCAAACAA AAAAACCACC GCTACCAGCG
```

```
        4561 GTGGTTTGTT TGCCGGATCA AGAGCTACCA ACTCTTTTTC CGAAGGTAAC TGGCTTCAGC
        4621 AGAGCGCAGA TACCAAATAC TGTTCTTCTA GTGTAGCCGT AGTTAGGCCA CCACTTCAAG
        4681 AACTCTGTAG CACCGCCTAC ATACCTCGCT CTGCTAATCC TGTTACCAGT GGCTGCTGCC
        4741 AGTGGCGATA AGTCGTGTCT TACCGGGTTG GACTCAAGAC GATAGTTACC GGATAAGGCG
        4801 CAGCGGTCGG GCTGAACGGG GGGTTCGTGC ACACAGCCCA GCTTGGAGCG AACGACCTAC
        4861 ACCGAACTGA GATACCTACA GCGTGAGCTA TGAGAAAGCG CCACGCTTCC CGAAGGGAGA
        4921 AAGGCGGACA GGTATCCGGT AAGCGGCAGG GTCGGAACAG GAGAGCGCAC GAGGGAGCTT
        4981 CCAGGGGGAA ACGCCTGGTA TCTTTATAGT CCTGTCGGGT TTCGCCACCT CTGACTTGAG
        5041 CGTCGATTTT TGTGATGCTC GTCAGGGGGG CGGAGCCTAT GGAAAAACGC CAGCAACGCG
        5101 GCCTTTTTAC GGTTCCTGGC CTTTTGCTGG CCTTTTGCTC ACATGTT
//
```

pDSAY (YFP):

```
LOCUS       pDSAY       5145 bp    DNA     circular
FEATURES             Location/Qualifiers
     misc_feature    complement(3406..3424)
                     /note="M13R"
     misc_feature    271..286
                     /note="M13F"
     misc_feature    600..1174
                     /note="LacZ"
     misc_feature    479..599
                     /note="lac promoter"
     misc_feature    590..608
                     /note="M13R"
     misc_feature    625..644
                     /note="T3"
     misc_feature    677..696
                     /note="SK primer"
     misc_feature    complement(731..747)
                     /note="KS primer"
     misc_feature    complement(800..816)
                     /note="M13F"
     misc_feature    complement(771..790)
                     /note="T7"
     misc_feature    1395..1621
                     /note="SV40 term"
     misc_feature    1622..1898
                     /note="attB"
     misc_feature    1972..2021
                     /note="3x Pax6 binding sites"
     misc_feature    2061..2068
                     /note="TATA"
     gene            2232..2948
                     /note="EYFP (Clontech)"
     misc_feature    2955..3198
                     /note="SV40 term sequences"

ORIGIN
           1 CTTTCCTGCG TTATCCCCTG ATTCTGTGGA TAACCGTATT ACCGCCTTTG AGTGAGCTGA
          61 TACCGCTCGC CGCAGCCGAA CGACCGAGCG CAGCGAGTCA GTGAGCGAGG AAGCGGAAGA
         121 GCGCCCAATA CGCAAACCGC CTCTCCCCGC GCGTTGGCCG ATTCATTAAT GCAGCTGGCA
         181 CGACAGGTTT CCCGACTGGA AAGCGGGCAG TGAGCGCAAC GCAATTAATA CGCGTACCGC
         241 TAGCATGGAT GTTTTCCCAG TCACGACGTT GTAAAACGAC GGCCAGTCTT AAGCTCGGGC
         301 CCCTACAGGT CACTAATACC ATCTAAGTAG TTGATTCATA GTGACTGGAT ATGTTGTGTT
         361 TTACAGTATT ATGTAGTCTG TTTTTTATGC AAAATCTAAT TTAATATATT GATATTTATA
         421 TCATTTTACG TTTCTCGTTC AACTTTTCTA TACAAAGTTg gtACcggatc cagagaccCG
         481 CAACGCAATT AATGTGAGTT AGCTCACTCA TTAGGCACCC CAGGCTTTAC ACTTTATGCT
         541 TCCGGCTCGT ATGTTGTGTG GAATTGTGAG CGGATAACAA TTTCACACAG GAAACAGCTA
         601 TGACCATGAT TACGCCAAGC GCGCAATTAA CCCTCACTAA AGGGAACAAA AGCTGGAGCT
         661 CCACCGCGGT GGCGGCCGCT CTAGAACTAG TGGATCCCCC GGGCTGCAGG AATTCGATAT
         721 CAAGCTTATC GATACCGTCG ACCTCGAGGG GGGGCCCGGT ACCCAATTCG CCCTATAGTG
```

```
 781 AGTCGTATTA CGCGCGCTCA CTGGCCGTCG TTTTACAACG TCGTGACTGG GAAAACCCTG
 841 GCGTTACCCA ACTTAATCGC CTTGCAGCAC ATCCCCCTTT CGCCAGCTGG CGTAATAGCG
 901 AAGAGGCCCG CACCGATCGC CCTTCCCAAC AGTTGCGCAG CCTGAATGGC GAATGGACG
 961 CGCCCTGTAG CGGCGCATTA AGCGCGGCGG GTGTGGTGGT TACGCGCAGC GTGACCGCTA
1021 CACTTGCCAG CGCCCTAGCG CCCGCTCCTT TCGCTTTCTT CCCTTCCTTT CTCGCCACGT
1081 TCGCCGGCTT TCCCCGTCAA GCTCTAAATC GGGGGCTCCC TTTAGGGTTC CGATTTAGTG
1141 CTTTACGGCA CCTCGACCCC AAAAAACTTG ATTAGGGTGA TGGTTCACGT AGTGGGCCAT
1201 CGCCCTGATA GACGGTTTTT CGCCCTTTGA CGTTGGAGTC CACGTTCTTT AATAGTGGAC
1261 TCTTGTTCCA AACTGGAACA ACACTCAACC CTATCTCGGT CTATTCTTTT GATTTATAAG
1321 GGATTTTGCC GATTTCGGCC TATTGGTTAA AAAATGAGCT GATTTAACAA AAATTTAACG
1381 CGggtctccg cttCTAGACA TAATCAGCCA TACCACATTT GTAGAGGTTT TACTTGCTTT
1441 AAAAAACCTC CCACACCTCC CCCTGAACCT GAAACATAAA ATGAATGCAA TTGTTGTTGT
1501 TAACTTGTTT ATTGCAGCTT ATAATGGTTA CAAATAAAGC AATAGCATCA CAAATTTCAC
1561 AAATAAAGCA TTTTTCTTCA CTGCATTCTA GTTGTGGTTT GTCCAAACTC ATCAATGTAT
1621 CtcgaCGATG TAGGTCACaG TCTCGAAGCC GCGGTGCGGG TGCCAGGGCG TGCCCTTGGG
1681 CTCCCCGGGC GCGTACTCCA CCTCACCCAT CTGGTCCATC ATGATGAACG GGTCGAGGTG
1741 GCGGTAGTTG ATCCCGGCGA ACGCGCGGCG CACCGGGAAG CCCTCGCCCT CGAAACCGCT
1801 GGGCGCGGTG GTCACGGTGA GCACGGGACG TGCGACGGCG TCGGCGGGTG CGGATACGCG
1861 GGGCAGCGTC AGCGGGTTCT CGACGGTCAC GGCGGGCAAT TCCTGCAGAC TTCCGGTATC
1921 TCGCGTTTGT TTGATCGCAC GGTTCCCACA ATGGTTAATT CGAGCTCGCC CGGGGATCTA
1981 ATTCAATTAG AGACTAATTC AATTAGAGCT AATTCAATTA GGATCCAAGC TTATCGATTT
2041 CGAACCCTCG ACCGCCGGAG TATAAATAGA GGCGCTTCGT CTACGGAGCG ACAATTCAAT
2101 TCAAACAAGC AAAGTGAACA CGTCGCTAAG CGAAAGCTAA GCAAATAAAC AAGCGCAGCT
2161 GAACAAGCTA AACAATCGGG GTACCGCTAG AGTCGACGGT ACCGCGGGCC CGGGATCCAC
2221 CGGTCGCCAC CATGGTGAGC AAGGGCGAGG AGCTGTTCAC CGGGGTGGTG CCCATCCTGG
2281 TCGAGCTGGA CGGCGACGTA AACGGCCACA AGTTCAGCGT GTCCGGCGAG GGCGAGGGCG
2341 ATGCCACCTA CGGCAAGCTG ACCCTGAAGT TCATCTGCAC CACCGGCAAG CTGCCCGTGC
2401 CCTGGCCCAC CCTCGTGACC ACCTTCGGCT ACGGCCTGCA GTGCTTCGCC CGCTACCCCG
2461 ACCACATGAA GCAGCACGAC TTCTTCAAGT CCGCCATGCC CGAAGGCTAC GTCCAGGAGC
2521 GCACCATCTT CTTCAAGGAC GACGGCAACT ACAAGACCCG CGCCGAGGTG AAGTTCGAGG
2581 GCGACACCCT GGTGAACCGC ATCGAGCTGA AGGGCATCGA CTTCAAGGAG GACGGCAACA
2641 TCCTGGGGCA CAAGCTGGAG TACAACTACA ACAGCCACAA CGTCTATATC ATGGCCGACA
2701 AGCAGAAGAA CGGCATCAAG GTGAACTTCA AGATCCGCCA CAACATCGAG GACGGCAGCG
2761 TGCAGCTCGC CGACCACTAC CAGCAGAACA CCCCCATCGG CGACGGCCCC GTGCTGCTGC
2821 CCGACAACCA CTACCTGAGC TACCAGTCCG CCCTGAGCAA AGACCCCAAC GAGAAGCGCG
2881 ATCACATGGT CCTGCTGGAG TTCGTGACCG CCGCCGGGAT CACTCTCGGC ATGGACGAGC
2941 TGTACAAGTA AAGCGGCCGC GACTCTAGAT CATAATCAGC CATACCACAT TTGTAGAGGT
3001 TTTACTTGCT TTAAAAAACC TCCCACACCT CCCCCTGAAC CTGAAACATA AAATGAATGC
3061 AATTGTTGTT GTTAACTTGT TTATTGCAGC TTATAATGGT TACAAATAAA GCAATAGCAT
3121 CACAAATTTC ACAAATAAAG CATTTTTCTT CACTGCATTC TAGTTGTGGT TTGTCCAAAC
3181 TCATCAATGT ATCTTAAAGC TTATCGATAC GCGTACGGCG CGCCTAGAGC GGCCGCCACC
3241 GCGGTGGAGC TCGAGTACCC AGCTTTCTTG TACAAAGTTG GCATTATAAG AAAGCATTGC
3301 TTATCAATTT GTTGCAACGA ACAGGTCACT ATCAGTCAAA ATAAAATCAT TATTTGCCAT
3361 CCAGCTGCAG GGCGGCCGCG ATATCCCCTA TAGTGAGTCG TATTACATGG TCATAGCTGT
3421 TTCCTGGCAG CTCTGGCCCG TGTCTCAAAA TCTCTGATGT TACATTGCAC AAGATAAAAA
3481 TATATCATCA TGAACAATAA AACTGTCTGC TTACATAAAC AGTAATACAA GGGGTGTTAT
3541 GAGCCATATT CAACGGGAAA CGTCGAGGCC GCGATTAAAT TCCAACATGG ATGCTGATTT
3601 ATATGGGTAT AAATGGGCTC GCGATAATGT CGGGCAATCA GGTGCGACAA TCTATCGCTT
3661 GTATGGGAAG CCCGATGCGC CAGAGTTGTT TCTGAAACAT GGCAAAGGTA GCGTTGCCAA
3721 TGATGTTACA GATGAGATGG TCAGACTAAA CTGGCTGACG GAATTTATGC CTCTTCCGAC
3781 CATCAAGCAT TTTATCCGTA CTCCTGATGA TGCATGGTTA CTCACCACTG CGATCCCCGG
3841 AAAAACAGCA TTCCAGGTAT TAGAAGAATA TCCTGATTCA GGTGAAAATA TTGTTGATGC
3901 GCTGGCAGTG TTCCTGCGCC GGTTGCATTC GATTCCTGTT TGTAATTGTC CTTTTAACAG
3961 CGATCGCGTA TTTCGTCTCG CTCAGGCGCA ATCACGAATG AATAACGGTT TGGTTGATGC
4021 GAGTGATTTT GATGACGAGC GTAATGGCTG GCCTGTTGAA CAAGTCTGGA AAGAAATGCA
4081 TAAACTTTTG CCATTCTCAC CGGATTCAGT CGTCACTCAT GGTGATTTCT CACTTGATAA
4141 CCTTATTTTT GACGAGGGGA AATTAATAGG TTGTATTGAT GTTGGACGAG TCGGAATCGC
4201 AGACCGATAC CAGGATCTTG CCATCCTATG GAACTGCCTC GGTGAGTTTT CTCCTTCATT
4261 ACAGAAACGG CTTTTTCAAA AATATGGTAT TGATAATCCT GATATGAATA AATTGCAGTT
4321 TCATTTGATG CTCGATGAGT TTTTCTAATC AGAATTGGTT AATTGGTTGT AACACTGGCA
4381 GAGCATTACG CTGACTTGAC GGGACGGCGC AAGCTCATGA CCAAAATCCC TTAACGTGAG
4441 TTACGCGTCG TTCCACTGAG CGTCAGACCC CGTAGAAAAG ATCAAAGGAT CTTCTTGAGA
4501 TCCTTTTTTT CTGCGCGTAA TCTGCTGCTT GCAAACAAAA AAACCACCGC TACCAGCGGT
4561 GGTTTGTTTG CCGGATCAAG AGCTACCAAC TCTTTTTCCG AAGGTAACTG GCTTCAGCAG
4621 AGCGCAGATA CCAAATACTG TTCTTCTAGT GTAGCCGTAG TTAGGCCACC ACTTCAAGAA
```

```
     4681 CTCTGTAGCA CCGCCTACAT ACCTCGCTCT GCTAATCCTG TTACCAGTGG CTGCTGCCAG
     4741 TGGCGATAAG TCGTGTCTTA CCGGGTTGGA CTCAAGACGA TAGTTACCGG ATAAGGCGCA
     4801 GCGGTCGGGC TGAACGGGGG GTTCGTGCAC ACAGCCCAGC TTGGAGCGAA CGACCTACAC
     4861 CGAACTGAGA TACCTACAGC GTGAGCTATG AGAAAGCGCC ACGCTTCCCG AAGGGAGAAA
     4921 GGCGGACAGG TATCCGGTAA GCGGCAGGGT CGGAACAGGA GAGCGCACGA GGGAGCTTCC
     4981 AGGGGGAAAC GCCTGGTATC TTTATAGTCC TGTCGGGTTT CGCCACCTCT GACTTGAGCG
     5041 TCGATTTTTG TGATGCTCGT CAGGGGGGCG GAGCCTATGG AAAAACGCCA GCAACGCGGC
     5101 CTTTTTACGG TTCCTGGCCT TTTGCTGGCC TTTTGCTCAC ATGTT
//
```

<u>pDSAR (DsRed2):</u>

```
LOCUS       pDSAR       5103 bp    DNA     linear       17-JAN-2013
FEATURES                Location/Qualifiers
     misc_feature       complement(3364..3382)
                        /note="M13R"
     misc_feature       2231..2912
                        /note="DsRed"
     misc_feature       1972..2021
                        /note="3x Pax6 binding sites"
     misc_feature       2061..2068
                        /note="TATA"
     misc_feature       271..286
                        /note="M13F"
     misc_feature       600..1174
                        /note="LacZ"
     misc_feature       479..599
                        /note="lac promoter"
     misc_feature       590..608
                        /note="M13R"
     misc_feature       625..644
                        /note="T3"
     misc_feature       677..696
                        /note="SK primer"
     misc_feature       complement(731..747)
                        /note="KS primer"
     misc_feature       complement(800..816)
                        /note="M13F"
     misc_feature       complement(771..790)
                        /note="T7"
     misc_feature       1395..1621
                        /note="SV40 term"
     misc_feature       1622..1898
                        /note="attB"
     misc_feature       2914..3154
                        /note="SV40 terminator"
ORIGIN
        1 CTTTCCTGCG TTATCCCCTG ATTCTGTGGA TAACCGTATT ACCGCCTTTG AGTGAGCTGA
       61 TACCGCTCGC CGCAGCCGAA CGACCGAGCG CAGCGAGTCA GTGAGCGAGG AAGCGGAAGA
      121 GCGCCCAATA CGCAAACCGC CTCTCCCCGC GCGTTGGCCG ATTCATTAAT GCAGCTGGCA
      181 CGACAGGTTT CCCGACTGGA AAGCGGGCAG TGAGCGCAAC GCAATTAATA CGCGTACCGC
      241 TAGCATGGAT GTTTTCCCAG TCACGACGTT GTAAAACGAC GGCCAGTCTT AAGCTCGGGC
      301 CCCTACAGGT CACTAATACC ATCTAAGTAG TTGATTCATA GTGACTGGAT ATGTTGTGTT
      361 TTACAGTATT ATGTAGTCTG TTTTTTATGC AAAATCTAAT TTAATATATT GATATTTATA
      421 TCATTTTACG TTTCTCGTTC AACTTTTCTA TACAAAGTTg gtACcggatc cagagaccCG
      481 CAACGCAATT AATGTGAGTT AGCTCACTCA TTAGGCACCC CAGGCTTTAC ACTTTATGCT
      541 TCCGGCTCGT ATGTTGTGTG GAATTGTGAG CGGATAACAA TTTCACACAG GAAACAGCTA
      601 TGACCATGAT TACGCCAAGC GCGCAATTAA CCCTCACTAA AGGGAACAAA AGCTGGAGCT
      661 CCACCGCGGT GGCGGCCGCT CTAGAACTAG TGGATCCCCC GGGCTGCAGG AATTCGATAT
      721 CAAGCTTATC GATACCGTCG ACCTCGAGGG GGGGCCCGGT ACCCAATTCG CCCTATAGTG
      781 AGTCGTATTA CGCGCGCTCA CTGGCCGTCG TTTTACAACG TCGTGACTGG GAAAACCCTG
      841 GCGTTACCCA ACTTAATCGC CTTGCAGCAC ATCCCCCTTT CGCCAGCTGG CGTAATAGCG
```

```
 901 AAGAGGCCCG CACCGATCGC CCTTCCCAAC AGTTGCGCAG CCTGAATGGC GAATGGGACG
 961 CGCCCTGTAG CGGCGCATTA AGCGCGGCGG GTGTGGTGGT TACGCGCAGC GTGACCGCTA
1021 CACTTGCCAG CGCCCTAGCG CCCGCTCCTT TCGCTTTCTT CCCTTCCTTT CTCGCCACGT
1081 TCGCCGGCTT TCCCCGTCAA GCTCTAAATC GGGGGCTCCC TTTAGGGTTC CGATTTAGTG
1141 CTTTACGGCA CCTCGACCCC AAAAAACTTG ATTAGGGTGA TGGTTCACGT AGTGGGCCAT
1201 CGCCCTGATA GACGGTTTTT CGCCCTTTGA CGTTGGAGTC CACGTTCTTT AATAGTGGAC
1261 TCTTGTTCCA AACTGGAACA ACACTCAACC CTATCTCGGT CTATTCTTTT GATTTATAAG
1321 GGATTTTGCC GATTTCGGCC TATTGGTTAA AAAATGAGCT GATTTAACAA AAATTTAACG
1381 CGggtctccg cttCTAGACA TAATCAGCCA TACCACATTT GTAGAGGTTT TACTTGCTTT
1441 AAAAAACCTC CCACACCTCC CCCTGAACCT GAAACATAAA ATGAATGCAA TTGTTGTTGT
1501 TAACTTGTTT ATTGCAGCTT ATAATGGTTA CAAATAAAGC AATAGCATCA CAAATTTCAC
1561 AAATAAAGCA TTTTTTTCA CTGCATTCTA GTTGTGGTTT GTCCAAACTC ATCAATGTAT
1621 CtcgaCGATG TAGGTCACaG TCTCGAAGCC GCGGTGCGGG TGCCAGGGCG TGCCCTTGGG
1681 CTCCCCGGGC GCGTACTCCA CCTCACCCAT CTGGTCCATC ATGATGAACG GGTCGAGGTG
1741 GCGGTAGTTG ATCCCGGCGA ACGCGCGGCG CACCGGGAAG CCCTCGCCCT CGAAACCGCT
1801 GGGCGCGGTC GTCACGGTGA GCACGGGACG TGCGACGGCG TCGGCGGGTG CGGATACGCG
1861 GGGCAGCGTC AGCGGGTTCT CGACGGTCAC GGCGGGCAat tCCTGCAGAC TTCCGGTATC
1921 TCGCGTTTGT TTGATCGCAC GGTTCCCACA ATGGTTAATT CGAGCTCGCC CGGGGATCTA
1981 ATTCAATTAG AGACTAATTC AATTAGAGCT AATTCAATTA GGATCCAAGC TTATCGATTT
2041 CGAACCCTCG ACCGCCGGAG TATAAATAGA GGCGCTTCGT CTACGGAGCG ACAATTCAAT
2101 TCAAACAAGC AAAGTGAACA CGTCGCTAAG CGAAAGCTAA GCAAATAAAC AAGCGCAGCT
2161 GAACAAGCTA AACAATCGGG GTACCGCTAG AGTCGACGGT ACCGCGGGCC CGGGATCCAC
2221 CGGTCGCCAC CATGGTGCGC TCCTCCAAGA ACGTCATCAA GGAGTTCATG CGCTTCAAGG
2281 TGCGCATGGA GGGCACCGTG AACGGCCACG AGTTCGAGAT CGAGGGCGAG GGCGAGGGCC
2341 GCCCCTACGA GGGCCACAAC ACCGTGAAGC TGAAGGTGAC CAAGGGCGGC CCCCTGCCCT
2401 TCGCCTGGGA CATCCTGTCC CCCCAGTTCC AGTACGGCTC CAAGGTGTAC GTGAAGCACC
2461 CCGCCGACAT CCCCGACTAC AAGAAGCTGT CCTTCCCCGA GGGCTTCAAG TGGGAGCGCG
2521 TGATGAACTT CGAGGACGGC GGCGTGGTGA CCGTGACCCA GGACTCCTCC CTGCAGGACG
2581 GCTGCTTCAT CTACAAGGTG AAGTTCATCG GCGTGAACTT CCCCTCCGAC GGCCCCGTAA
2641 TGCAGAAGAA GACCATGGGC TGGGAGGCCT CCACCGAGCG CCTGTACCCC CGCGACGGCG
2701 TGCTGAAGGG CGAGATCCAC AAGGCCCTGA AGCTGAAGGA CGGCGGCCAC TACCTGGTGG
2761 AGTTCAAGTC CATCTACATG GCCAAGAAGC CCGTGCAGCT GCCCGGCTAC TACTACGTGG
2821 ACTCCAAGCT GGACATCACC TCCCACAACG AGGACTACAC CATCGTGGAg CAGTACGAGC
2881 GCACCGAGGG CCGCCACCAC CTGTTCCTGT AGCGGCCGCG ACTCTAGATC ATAATCAGCC
2941 ATACCACATT TGTAGAGGTT TTACTTGCTT TAAAAAACCT CCCACACCTC CCCCTGAACC
3001 TGAAACATAA AATGAATGCA ATTGTTGTTG TTAACTTGTT TATTGCAGCT TATAATGGTT
3061 ACAAATAAAG CAATAGCATC ACAAATTTCA CAAATAAAGC ATTTTTTTCA CTGCATTCTA
3121 GTTGTGGTTT GTCCAAACTC ATCAATGTAT CTTAAAGCTT ATCGATACGC GTACGGCGCG
3181 CCTAGAGCGG CCGCCACCGC GGTGGAGCTC GAGTACCCAG CTTTCTTGTA CAAAGTTGGC
3241 ATTATAAGAA AGCATTGCTT ATCAATTTGT TGCAACGAAC AGGTCACTAT CAGTCAAAAT
3301 AAAATCATTA TTTGCCATCC AGCTGCAGGG CGGCCGCGAT ATCCCCTATA GTGAGTCGTA
3361 TTACATGGTC ATAGCTGTTT CCTGGCAGCT CTGGCCCGTG TCTCAAAATC TCTGATGTTA
3421 CATTGCACAA GATAAAAATA TATCATCATG AACAATAAAA CTGTCTGCTT ACATAAACAG
3481 TAATACAAGG GGTGTTATGA GCCATATTCA ACGGGAAACG TCGAGGCCGC GATTAAATTC
3541 CAACATGGAT GCTGATTTAT ATGGGTATAA ATGGGCTCGC GATAATGTCG GGCAATCAGG
3601 TGCGACAATC TATCGCTTGT ATGGGAAGCC CGATGCGCCA GAGTTGTTTC TGAAACATGG
3661 CAAAGGTAGC GTTGCCAATG ATGTTACAGA TGAGATGGTC AGACTAAACT GGCTGACGGA
3721 ATTTATGCCT CTTCCGACCA TCAAGCATTT TATCCGTACT CCTGATGATG CATGGTTACT
3781 CACCACTGCG ATCCCCGGAA AAACAGCATT CCAGGTATTA GAAGAATATC CTGATTCAGG
3841 TGAAAATATT GTTGATGCGC TGGCAGTGTT CCTGCGCCGG TTGCATTCGA TTCCTGTTTG
3901 TAATTGTCCT TTTAACAGCG ATCGCGTATT TCGTCTCGCT CAGGCGCAAT CACGAATGAA
3961 TAACGGTTTG GTTGATGCGA GTGATTTTGA TGACGAGCGT AATGGCTGGC CTGTTGAACA
4021 AGTCTGGAAA GAAATGCATA AACTTTTGCC ATTCTCACCG GATTCAGTCG TCACTCATGG
4081 TGATTTCTCA CTTGATAACC TTATTTTTGA CGAGGGGAAA TTAATAGGTT GTATTGATGT
4141 TGGACGAGTC GGAATCGCAG ACCGATACCA GGATCTTGCC ATCCTATGGA ACTGCCTCGG
4201 TGAGTTTTCT CCTTCATTAC AGAAACGGCT TTTTCAAAAA TATGGTATTG ATAATCCTGA
4261 TATGAATAAA TTGCAGTTTC ATTTGATGCT CGATGAGTTT TTCTAATCAG AATTGGTTAA
4321 TTGGTTGTAA CACTGGCAGA GCATTACGCT GACTTGACGG GACGGCGCAA GCTCATGACC
4381 AAAATCCCTT AACGTGAGTT ACGCGTCGTT CCACTGAGCG TCAGACCCCG TAGAAAAGAT
4441 CAAAGGATCT TCTTGAGATC CTTTTTTTCT GCGCGTAATC TGCTGCTTGC AAACAAAAAA
4501 ACCACCGCTA CCAGCGGTGG TTTGTTTGCC GGATCAAGAG CTACCAACTC TTTTTCCGAA
4561 GGTAACTGGC TTCAGCAGAG CGCAGATACC AAATACTGTT CTTCTAGTGT AGCCGTAGTT
4621 AGGCCACCAC TTCAAGAACT CTGTAGCACC GCCTACATAC CTCGCTCTGC TAATCCTGTT
4681 ACCAGTGGCT GCTGCCAGTG GCGATAAGTC GTGTCTTACC GGGTTGGACT CAAGACGATA
4741 GTTACCGGAT AAGGCGCAGC GGTCGGGCTG AACGGGGGGT TCGTGCACAC AGCCCAGCTT
```

```
        4801 GGAGCGAACG ACCTACACCG AACTGAGATA CCTACAGCGT GAGCTATGAG AAAGCGCCAC
        4861 GCTTCCCGAA GGGAGAAAGG CGGACAGGTA TCCGGTAAGC GGCAGGGTCG GAACAGGAGA
        4921 GCGCACGAGG GAGCTTCCAG GGGGAAACGC CTGGTATCTT TATAGTCCTG TCGGGTTTCG
        4981 CCACCTCTGA CTTGAGCGTC GATTTTTGTG ATGCTCGTCA GGGGGGCGGA GCCTATGGAA
        5041 AAACGCCAGC AACGCGGCCT TTTTACGGTT CCTGGCCTTT TGCTGGCCTT TTGCTCACAT
        5101 GTT
//
```

pDSAP (puromycin resistance):

```
LOCUS       pDSAP                  5456 bp    DNA     circular
FEATURES             Location/Qualifiers
     misc_feature    complement(3717..3735)
                     /note="M13R"
     misc_feature    1972..2021
                     /note="3x Pax6 binding sites"
     misc_feature    271..286
                     /note="M13F"
     misc_feature    600..1174
                     /note="LacZ"
     misc_feature    479..599
                     /note="lac promoter"
     misc_feature    590..608
                     /note="M13R"
     misc_feature    625..644
                     /note="T3"
     misc_feature    677..696
                     /note="SK primer"
     misc_feature    complement(731..747)
                     /note="KS primer"
     misc_feature    complement(800..816)
                     /note="M13F"
     misc_feature    complement(771..790)
                     /note="T7"
     misc_feature    1395..1621
                     /note="SV40 terminator"
     misc_feature    1622..1898
                     /note="attB"
     misc_feature    2114..2762
                     /note="OpIE2 promoter"
     misc_feature    2763..3364
                     /note="puromycin resistance ORF"
     misc_feature    3364..3523
                     /note="SV40 terminator"
     misc_feature    complement(3761..3779)
                     /note="pDONR-RP"

ORIGIN
           1 CTTTCCTGCG TTATCCCCTG ATTCTGTGGA TAACCGTATT ACCGCCTTTG AGTGAGCTGA
          61 TACCGCTCGC CGCAGCCGAA CGACCGAGCG CAGCGAGTCA GTGAGCGAGG AAGCGGAAGA
         121 GCGCCCAATA CGCAAACCGC CTCTCCCCGC GCGTTGGCCG ATTCATTAAT GCAGCTGGCA
         181 CGACAGGTTT CCCGACTGGA AAGCGGGCAG TGAGCGCAAC GCAATTAATA CGCGTACCGC
         241 TAGCATGGAT GTTTTCCCAG TCACGACGTT GTAAAACGAC GGCCAGTCTT AAGCTCGGGC
         301 CCCTACAGGT CACTAATACC ATCTAAGTAG TTGATTCATA GTGACTGGAT ATGTTGTGTT
         361 TTACAGTATT ATGTAGTCTG TTTTTTATGC AAAATCTAAT TTAATATATT GATATTTATA
         421 TCATTTTACG TTTCTCGTTC AACTTTTCTA TACAAAGTTg gtACcggatc cagagacccG
         481 CAACGCAATT AATGTGAGTT AGCTCACTCA TTAGGCACCC CAGGCTTTAC ACTTTATGCT
         541 TCCGGCTCGT ATGTTGTGTG GAATTGTGAG CGGATAACAA TTTCACACAG GAAACAGCTA
         601 TGACCATGAT TACGCCAAGC GCGCAATTAA CCCTCACTAA AGGGAACAAA AGCTGGAGCT
         661 CCACCGCGGT GGCGGCCGCT CTAGAACTAG TGGATCCCCC GGGCTGCAGG AATTCGATAT
         721 CAAGCTTATC GATACCGTCG ACCTCGAGGG GGGGCCCGGT ACCCAATTCG CCCTATAGTG
         781 AGTCGTATTA CGCGCGCTCA CTGGCCGTCG TTTTACAACG TCGTGACTGG GAAAACCCTG
```

```
 841 GCGTTACCCA ACTTAATCGC CTTGCAGCAC ATCCCCCTTT CGCCAGCTGG CGTAATAGCG
 901 AAGAGGCCCG CACCGATCGC CCTTCCCAAC AGTTGCGCAG CCTGAATGGC GAATGGACG
 961 CGCCCTGTAG CGGCGCATTA AGCGCGGCGG GTGTGGTGGT TACGCGCAGC GTGACCGCTA
1021 CACTTGCCAG CGCCCTAGCG CCCGCTCCTT TCGCTTTCTT CCCTTCCTTT CTCGCCACGT
1081 TCGCCGGCTT TCCCCGTCAA GCTCTAAATC GGGGGCTCCC TTTAGGGTTC CGATTTAGTG
1141 CTTTACGGCA CCTCGACCCC AAAAAACTTG ATTAGGGTGA TGGTTCACGT AGTGGGCCAT
1201 CGCCCTGATA GACGGTTTTT CGCCCTTTGA CGTTGGAGTC CACGTTCTTT AATAGTGGAC
1261 TCTTGTTCCA AACTGGAACA ACACTCAACC CTATCTCGGT CTATTCTTTT GATTTATAAG
1321 GGATTTTGCC GATTTCGGCC TATTGGTTAA AAAATGAGCT GATTTAACAA AAATTTAACG
1381 CGggtctccg cttCTAGACA TAATCAGCCA TACCACATTT GTAGAGGTTT TACTTGCTTT
1441 AAAAAACCTC CCACACCTCC CCCTGAACCT GAAACATAAA ATGAATGCAA TTGTTGTTGT
1501 TAACTTGTTT ATTGCAGCTT ATAATGGTTA CAAATAAAGC AATAGCATCA CAAATTTCAC
1561 AAATAAAGCA TTTTTTTCA CTGCATTCTA GTTGTGGTTT GTCCAAACTC ATCAATGTAT
1621 CtcgaCGATG TAGGTCACaG TCTCGAAGCC GCGGTGCGGG TGCCAGGGCG TGCCCTTGGG
1681 CTCCCCGGGC GCGTACTCCA CCTCACCCAT CTGGTCCATC ATGATGAACG GGTCGAGGTG
1741 GCGGTAGTTG ATCCCGGCGA ACGCGCGGCG CACCGGGAAG CCCTCGCCCT CGAAACCGCT
1801 GGGCGCGGTG GTCACGGTGA GCACGGGACG TGCGACGGCG TCGGCGGGTG CGGATACGCG
1861 GGGCAGCGTC AGCGGGTTCT CGACGGTCAC GGCGGGCAat tCCTGCAGAC TTCCGGTATC
1921 TCGCGTTTGT TTGATCGCAC GGTTCCCACA ATGGTTAATT CGAGCTCGCC CGGGGATCTA
1981 ATTCAATTAG AGACTAATTC AATTAGAGCT AATTCAATTA GGATCCAAGC TTATCGATTT
2041 CGATAAGCTT GATATCGAAT TCCTGCAGCC CGGGGGATCC Actagtggat cccccgggct
2101 gcaggaattc gatCATGATG ATAAACAATG TATGGTGCTA ATGTTGCTTC AACAACAATT
2161 CTGTTGAACT GTGTTTTCAT GTTTGCCAAC AAGCACCTTT ATACTCGGTG GCCTCCCCAC
2221 CACCAACTTT TTTGCACTGC AAAAAAACAC GCTTTTGCAC GCGGGCCCAT ACATAGTACA
2281 AACTCTACGT TTCGTAGACT ATTTTACATA AATAGTCTAC ACCGTTGTAT ACGCTCCAAA
2341 TACACTACCA CACATTGAAC CTTTTTGCAG TGCAAAAAAG TACGTGTCGG CAGTCACGTA
2401 GGCCGGCCTT ATCGGGTCGC GTCCTGTCAC GTACGAATCA CATTATCGGA CCGGACGAGT
2461 GTTGTCTTAT CGTGACAGGA CGCCAGCTTC CTGTGTTGCT AACCGCAGCC GGACGCAACT
2521 CCTTATCGGA ACAGGACGCG CCTCCATATC AGCCGCGCGT TATCTCATGC GCGTGACCGG
2581 ACACGAGGCG CCCGTCCCGC TTATCGCGCC TATAAATACA GCCCGCAACG ATCTGGTAAA
2641 CACAGTTGAA CAGCATCTGT TCGAATTAAT TCgGGATCTg ccgggCTGCA GCACGTGTTG
2701 ACAATTAATC ATCGGCATAG TATATCGGCA TAGTATAATA CGACTCACTA TAGGAGGGCC
2761 ACcATGaccg agtacaagcc cacggtgcgc ctcgccaccc gcgacgacgt ccccAgggcc
2821 gtacgcaccc tcgccgccgc gttcgccgac taccccgcca cgcgccacac cgtcgaTccg
2881 gaccgccaca tcgagcgggt caccgagctg caagaactct tcctcacgcg cgtcgggctc
2941 gacatcggca aggtgtgggt cgcggacgac ggcgccgcgg tggcggtctg gaccacgccg
3001 gagagcgtcg aagcgggggc ggtgttcgcc gagatcggcc cgcgcatggc cgagttgagc
3061 ggttcccggc tggccgcgca gcaacagatg gaaggcctcc tggcgccgca ccggcccaag
3121 gagcccgcgt ggttcctggc caccgtcggc gtctcgcccg accaccaggg caagggtctg
3181 ggcagcgccg tcgtgctccc cggagtggag gcggccgagc gcgccggggt gcccgccttc
3241 ctggagacct ccgcgccccg caacctcccc ttctacgagc ggctcggctt caccgtcacc
3301 gccgacgtcg agGtgcccga aggaccgcgc acctggtgca tgacccgcaa gcccggtgcc
3361 tgaCTCGACC TCGAAACTTG TTTATTGCAG CTTATAATGG TTACAAATAA AGCAATAGCA
3421 TCACAAATTT CACAAATAAA GCATTTTTTT CACTGCATTC TAGTTGTGGT TTGTCCAAAC
3481 TCATCAATGT ATCTTATCAT GTCTggatca tggggggggc ccgGTACGGC GCGCCTAGAG
3541 CGGCCGCCAC CGCGGTGGAG CTCGAGTACC CAGCTTTCTT GTACAAAGTT GGCATTATAA
3601 GAAAGCATTG CTTATCAATT TGTTGCAACG AACAGGTCAC TATCAGTCAA AATAAAATCA
3661 TTATTTGCCA TCCAGCTGCA GGGCGGCCGC GATATCCCCT ATAGTGAGTC GTATTACATG
3721 GTCATAGCTG TTTCCTGGCA GCTCTGGCCC GTGTCTCAAA ATCTCTGATG TTACATTGCA
3781 CAAGATAAAA ATATATCATC ATGAACAATA AAACTGTCTG CTTACATAAA CAGTAATACA
3841 AGGGGTGTTA TGAGCCATAT TCAACGGGAA ACGTCGAGGC CGCGATTAAA TTCCAACATG
3901 GATGCTGATT TATATGGGTA TAAATGGGCT CGCGATAATG TCGGGCAATC AGGTGCGACA
3961 ATCTATCGCT TGTATGGGAA GCCCGATGCG CCAGAGTTGT TTCTGAAACA TGGCAAAGGT
4021 AGCGTTGCCA ATGATGTTAC AGATGAGATG GTCAGACTAA ACTGGCTGAC GGAATTTATG
4081 CCTCTTCCGA CCATCAAGCA TTTTATCCGT ACTCCTGATG ATGCATGGTT ACTCACCACT
4141 GCGATCCCCG GAAAAACAGC ATTCCAGGTA TTAGAAGAAT ATCCTGATTC AGGTGAAAAT
4201 ATTGTTGATG CGCTGGCAGT GTTCCTGCGC CGGTTGCATT CGATTCCTGT TTGTAATTGT
4261 CCTTTTAACA GCGATCGCGT ATTTCGTCTC GCTCAGGCGC AATCACGAAT GAATAACGGT
4321 TTGGTTGATG CGAGTGATTT TGATGACGAG CGTAATGGCT GGCCTGTTGA ACAAGTCTGG
4381 AAAGAAATGC ATAAACTTTT GCCATTCTCA CCGGATTCAG TCGTCACTCA TGGTGATTTC
4441 TCACTTGATA ACCTTATTTT TGACGAGGGG AAATTAATAG GTTGTATTGA TGTTGGACGA
4501 GTCGGAATCG CAGACCGATA CCAGGATCTT GCCATCCTAT GGAACTGCCT CGGTGAGTTT
4561 TCTCCTTCAT TACAGAAACG GCTTTTTCAA AAATATGGTA TTGATAATCC TGATATGAAT
4621 AAATTGCAGT TTCATTTGAT GCTCGATGAG TTTTTCTAAT CAGAATTGGT TAATTGGTTG
4681 TAACACTGGC AGAGCATTAC GCTGACTTGA CGGGACGGCG CAAGCTCATG ACCAAAATCC
```

```
     4741 CTTAACGTGA GTTACGCGTC GTTCCACTGA GCGTCAGACC CCGTAGAAAA GATCAAAGGA
     4801 TCTTCTTGAG ATCCTTTTTT TCTGCGCGTA ATCTGCTGCT TGCAAACAAA AAAACCACCG
     4861 CTACCAGCGG TGGTTTGTTT GCCGGATCAA GAGCTACCAA CTCTTTTTCC GAAGGTAACT
     4921 GGCTTCAGCA GAGCGCAGAT ACCAAATACT GTTCTTCTAG TGTAGCCGTA GTTAGGCCAC
     4981 CACTTCAAGA ACTCTGTAGC ACCGCCTACA TACCTCGCTC TGCTAATCCT GTTACCAGTG
     5041 GCTGCTGCCA GTGGCGATAA GTCGTGTCTT ACCGGGTTGG ACTCAAGACG ATAGTTACCG
     5101 GATAAGGCGC AGCGGTCGGG CTGAACGGGG GGTTCGTGCA CACAGCCCAG CTTGGAGCGA
     5161 ACGACCTACA CCGAACTGAG ATACCTACAG CGTGAGCTAT GAGAAAGCGC CACGCTTCCC
     5221 GAAGGGAGAA AGGCGGACAG GTATCCGGTA AGCGGCAGGG TCGGAACAGG AGAGCGCACG
     5281 AGGGAGCTTC CAGGGGGAAA CGCCTGGTAT CTTTATAGTC CTGTCGGGTT TCGCCACCTC
     5341 TGACTTGAGC GTCGATTTTT GTGATGCTCG TCAGGGGGGC GGAGCCTATG GAAAAACGCC
     5401 AGCAACGCGG CCTTTTTACG GTTCCTGGCC TTTTGCTGGC CTTTTGCTCA CATGTT
//
```

pDSAYN (YFPnls):

```
LOCUS       pDSAYnls                5187 bp    DNA     circular
FEATURES             Location/Qualifiers
     misc_feature    complement(3448..3466)
                     /note="M13R"
     misc_feature    1972..2021
                     /note="3x Pax6 binding sites"
     misc_feature    2061..2068
                     /note="TATA"
     misc_feature    271..286
                     /note="M13F"
     misc_feature    600..1174
                     /note="LacZ"
     misc_feature    479..599
                     /note="lac promoter"
     misc_feature    590..608
                     /note="M13R"
     misc_feature    625..644
                     /note="T3"
     misc_feature    677..696
                     /note="SK primer"
     misc_feature    complement(731..747)
                     /note="KS primer"
     misc_feature    complement(800..816)
                     /note="M13F"
     misc_feature    complement(771..790)
                     /note="T7"
     misc_feature    1395..1621
                     /note="SV40 terminator"
     misc_feature    1622..1898
                     /note="attB"
     CDS             2232..2951
                     /dnas_title="YFP"
                     /vntifkey="4"
                     /label=YFP
     3'UTR           3007..3234
                     /dnas_title="SV40 3'UTR"
                     /vntifkey="50"
                     /label=SV40 3'UTR
     sig_peptide     2949..2987
                     /dnas_title="NLS"
                     /vntifkey="94"
                     /label=NLS
     source          1..5187
                     /dnas_title="pDSAYnls"

ORIGIN
```

```
   1 CTTTCCTGCG TTATCCCCTG ATTCTGTGGA TAACCGTATT ACCGCCTTTG AGTGAGCTGA
  61 TACCGCTCGC CGCAGCCGAA CGACCGAGCG CAGCGAGTCA GTGAGCGAGG AAGCGGAAGA
 121 GCGCCCAATA CGCAAACCGC CTCTCCCCGC GCGTTGGCCG ATTCATTAAT GCAGCTGGCA
 181 CGACAGGTTT CCCGACTGGA AAGCGGGCAG TGAGCGCAAC GCAATTAATA CGCGTACCGC
 241 TAGCATGGAT GTTTTCCCAG TCACGACGTT GTAAAACGAC GGCCAGTCTT AAGCTCGGGC
 301 CCCTACAGGT CACTAATACC ATCTAAGTAG TTGATTCATA GTGACTGGAT ATGTTGTGTT
 361 TTACAGTATT ATGTAGTCTG TTTTTTATGC AAAATCTAAT TTAATATATT GATATTTATA
 421 TCATTTTACG TTTCTCGTTC AACTTTTCTA TACAAAGTTg gtACcggatc cagagaccCG
 481 CAACGCAATT AATGTGAGTT AGCTCACTCA TTAGGCACCC CAGGCTTTAC ACTTTATGCT
 541 TCCGGCTCGT ATGTTGTGTG GAATTGTGAG CGGATAACAA TTTCACACAG GAAACAGCTA
 601 TGACCATGAT TACGCCAAGC GCGCAATTAA CCCTCACTAA AGGGAACAAA AGCTGGAGCT
 661 CCACCGCGGT GGCGGCCGCT CTAGAACTAG TGGATCCCCC GGGCTGCAGG AATTCGATAT
 721 CAAGCTTATC GATACCGTCG ACCTCGAGGG GGGGCCCGGT ACCCAATTCG CCCTATAGTG
 781 AGTCGTATTA CGCGCGCTCA CTGGCCGTCG TTTTACAACG TCGTGACTGG GAAAACCCTG
 841 GCGTTACCCA ACTTAATCGC CTTGCAGCAC ATCCCCCTTT CGCCAGCTGG CGTAATAGCG
 901 AAGAGGCCCG CACCGATCGC CCTTCCCAAC AGTTGCGCAG CCTGAATGGC GAATGGGACG
 961 CGCCCTGTAG CGGCGCATTA AGCGCGGCGG GTGTGGTGGT TACGCGCAGC GTGACCGCTA
1021 CACTTGCCAG CGCCCTAGCG CCCGCTCCTT TCGCTTTCTT CCCTTCCTTT CTCGCCACGT
1081 TCGCCGGCTT TCCCCGTCAA GCTCTAAATC GGGGGCTCCC TTTAGGGTTC CGATTTAGTG
1141 CTTTACGGCA CCTCGACCCC AAAAAACTTG ATTAGGGTGA TGGTTCACGT AGTGGGCCAT
1201 CGCCCTGATA GACGGTTTTT CGCCCTTTGA CGTTGGAGTC CACGTTCTTT AATAGTGGAC
1261 TCTTGTTCCA AACTGGAACA ACACTCAACC CTATCTCGGT CTATTCTTTT GATTTATAAG
1321 GGATTTTGCC GATTTCGGCC TATTGGTTAA AAAATGAGCT GATTTAACAA AAATTTAACG
1381 CGggtctccg cttCTAGACA TAATCAGCCA TACCACATTT GTAGAGGTTT TACTTGCTTT
1441 AAAAAACCTC CCACACCTCC CCCTGAACCT GAAACATAAA ATGAATGCAA TTGTTGTTGT
1501 TAACTTGTTT ATTGCAGCTT ATAATGGTTA CAAATAAAGC AATAGCATCA CAAATTTCAC
1561 AAATAAAGCA TTTTTCTTCA CTGCATTCTA GTTGTGGTTT GTCCAAACTC ATCAATGTAT
1621 CtcgaCGATG TAGGTCACaG TCTCGAAGCC GCGGTGCGGG TGCCAGGGCG TGCCCTTGGG
1681 CTCCCCGGGC GCGTACTCCA CCTCACCCAT CTGGTCCATC ATGATGAACG GGTCGAGGTG
1741 GCGGTAGTTG ATCCCGGCGA ACGCGCGGCG CACCGGGAAG CCCTCGCCCT CGAAACCGCT
1801 GGGCGCGGTG GTCACGGTGA GCACGGGACG TGCGACGGCG TCGGCGGGTG CGGATACGCG
1861 GGGCAGCGTC AGCGGGTTCT CGACGGTCAC GGCGGGCAat tCCTGCAGAC TTCCGGTATC
1921 TCGCGTTTGT TTGATCGCAC GGTTCCCACA ATGGTTAATT CGAGCTCGCC CGGGGATCTA
1981 ATTCAATTAG AGACTAATTC AATTAGAGCT AATTCAATTA GGATCCAAGC TTATCGATTT
2041 CGAACCCTCG ACCGCCGGAG TATAAATAGA GGCGCTTCGT CTACGGAGCG ACAATTCAAT
2101 TCAAACAAGC AAAGTGAACA CGTCGCTAAG CGAAAGCTAA GCAAATAAAC AAGCGCAGCT
2161 GAACAAGCTA AACAATCGGG GTACCGCTAG AGTCGACGGT ACCGCGGGCC CGGGATCCAc
2221 cggtcgccac catggtgagc aagggcgagg agctgttcac cggggtggtg cccatcctgg
2281 tcgagctgga cggcgacgta aacggccaca agttcagcgt gtccggcgag ggcgagggcg
2341 atgccaccta cggcaagctg accctgaagt tcatctgcac caccggcaag ctgcccgtgc
2401 cctggcccac cctcgtgacc accttcggct acggcctgca gtgcttcgcc cgctaccccg
2461 accacatgaa gcagcacgac ttcttcaagt ccgccatgcc cgaaggctac gtccaggagc
2521 gcaccatctt cttcaaggac gacggcaact acaagacccg cgccgaggtg aagttcgagg
2581 gcgacaccct ggtgaaccgc atcgagctga agggcatcga cttcaaggag gacggcaaca
2641 tcctggggca caagctggag tacaactaca acagccacaa cgtctatatc atggccgaca
2701 agcagaagaa cggcatcaag gtgaacttca agatccgcca caacatcgag gacggcagcg
2761 tgcagctcgc cgaccactac cagcagaaca cccccatcgg cgacggcccc gtgctgctgc
2821 ccgacaacca ctacctgagc taccagtccg ccctgagcaa agaccccaac gagaagcgcg
2881 atcacatggt cctgctggag ttcgtgaccg ccgccgggat cactctcggc atggacgagc
2941 tgtacaagag atctcgaccc aagaaaaagc ggaaggtgga ggacccgtaa agcggccgcg
3001 actctagatc ataatcagcc ataccacatt tgtagaggtt ttacttgctt taaaaaacct
3061 cccacacctc cccctgaacc tgaaacataa aatgaatgca attgttgttg ttaacttgtt
3121 tattgcagct tataatggtt acaaataaag caatagcatc acaaatttca caaataaagc
3181 atttttttca ctgcattcta gttgtggttt gtccaaactc atcaatgtat cttaaagctt
3241 atcgatacgc GTACGtacgG CGCGCCTAGA GCGGCCGCCA CCGCGGTGGA GCTCGAGTAC
3301 CCAGCTTTCT TGTACAAAGT TGGCATTATA AGAAAGCATT GCTTATCAAT TTGTTGCAAC
3361 GAACAGGTCA CTATCAGTCA AAATAAAATC ATTATTTGCC ATCCAGCTGC AGGGCGGCCG
3421 CGATATCCCC TATAGTGAGT CGTATTACAT GGTCATAGCT GTTTCCTGGC AGCTCTGGCC
3481 CGTGTCTCAA AATCTCTGAT GTTACATTGC ACAAGATAAA AATATATCAT CATGAACAAT
3541 AAAACTGTCT GCTTACATAA ACAGTAATAC AAGGGGTGTT ATGAGCCATA TTCAACGGGA
3601 AACGTCGAGG CCGCGATTAA ATTCCAACAT GGATGCTGAT TTATATGGGT ATAAATGGGC
3661 TCGCGATAAT GTCGGGCAAT CAGGTGCGAC AATCTATCGC TTGTATGGGA AGCCCGATGC
3721 GCCAGAGTTG TTTCTGAAAC ATGGCAAAGG TAGCGTTGCC AATGATGTTA CAGATGAGAT
3781 GGTCAGACTA AACTGGCTGA CGGAATTTAT GCCTCTTCCG ACCATCAAGC ATTTTATCCG
3841 TACTCCTGAT GATGCATGGT TACTCACCAC TGCGATCCCC GGAAAAACAG CATTCCAGGT
```

```
     3901 ATTAGAAGAA TATCCTGATT CAGGTGAAAA TATTGTTGAT GCGCTGGCAG TGTTCCTGCG
     3961 CCGGTTGCAT TCGATTCCTG TTTGTAATTG TCCTTTTAAC AGCGATCGCG TATTTCGTCT
     4021 CGCTCAGGCG CAATCACGAA TGAATAACGG TTTGGTTGAT GCGAGTGATT TTGATGACGA
     4081 GCGTAATGGC TGGCCTGTTG AACAAGTCTG GAAAGAAATG CATAAACTTT TGCCATTCTC
     4141 ACCGGATTCA GTCGTCACTC ATGGTGATTT CTCACTTGAT AACCTTATTT TTGACGAGGG
     4201 GAAATTAATA GGTTGTATTG ATGTTGGACG AGTCGGAATC GCAGACCGAT ACCAGGATCT
     4261 TGCCATCCTA TGGAACTGCC TCGGTGAGTT TTCTCCTTCA TTACAGAAAC GGCTTTTTCA
     4321 AAAATATGGT ATTGATAATC CTGATATGAA TAAATTGCAG TTTCATTTGA TGCTCGATGA
     4381 GTTTTTCTAA TCAGAATTGG TTAATTGGTT GTAACACTGG CAGAGCATTA CGCTGACTTG
     4441 ACGGGACGGC GCAAGCTCAT GACCAAAATC CCTTAACGTG AGTTACGCGT CGTTCCACTG
     4501 AGCGTCAGAC CCCGTAGAAA AGATCAAAGG ATCTTCTTGA GATCCTTTTT TTCTGCGCGT
     4561 AATCTGCTGC TTGCAAACAA AAAAACCACC GCTACCAGCG GTGGTTTGTT TGCCGGATCA
     4621 AGAGCTACCA ACTCTTTTTC CGAAGGTAAC TGGCTTCAGC AGAGCGCAGA TACCAAATAC
     4681 TGTTCTTCTA GTGTAGCCGT AGTTAGGCCA CCACTTCAAG AACTCTGTAG CACCGCCTAC
     4741 ATACCTCGCT CTGCTAATCC TGTTACCAGT GGCTGCTGCC AGTGGCGATA AGTCGTGTCT
     4801 TACCGGGTTG GACTCAAGAC GATAGTTACC GGATAAGGCG CAGCGGTCGG GCTGAACGGG
     4861 GGGTTCGTGC ACACAGCCCA GCTTGGAGCG AACGACCTAC ACCGAACTGA GATACCTACA
     4921 GCGTGAGCTA TGAGAAAGCG CCACGCTTCC CGAAGGGAGA AAGGCGGACA GGTATCCGGT
     4981 AAGCGGCAGG GTCGGAACAG GAGAGCGCAC GAGGGAGCTT CCAGGGGGAA ACGCCTGGTA
     5041 TCTTTATAGT CCTGTCGGGT TTCGCCACCT CTGACTTGAG CGTCGATTTT TGTGATGCTC
     5101 GTCAGGGGGG CGGAGCCTAT GGAAAAACGC CAGCAACGCG GCCTTTTTAC GGTTCCTGGC
     5161 CTTTTGCTGG CCTTTTGCTC ACATGTT
//
```

pDSARN (DsRed2NLS):

```
LOCUS       pDSARed2nls             5247 bp    DNA     circular
FEATURES             Location/Qualifiers
     misc_feature    complement(3508..3526)
                     /note="M13R"
     misc_feature    1972..2021
                     /note="3x Pax6 binding sites"
     misc_feature    2061..2068
                     /note="TATA"
     misc_feature    271..286
                     /note="M13F"
     misc_feature    600..1174
                     /note="LacZ"
     misc_feature    479..599
                     /note="lac promoter"
     misc_feature    590..608
                     /note="M13R"
     misc_feature    625..644
                     /note="T3"
     misc_feature    677..696
                     /note="SK primer"
     misc_feature    complement(731..747)
                     /note="KS primer"
     misc_feature    complement(800..816)
                     /note="M13F"
     misc_feature    complement(771..790)
                     /note="T7"
     misc_feature    1395..1621
                     /note="SV40 terminator"
     misc_feature    1622..1898
                     /note="attB"
     polyA_signal    2961..3208
                     /dnas_title="SV40"
                     /vntifkey="25"
                     /label=SV40
     misc_feature    complement(3259..3292)
                     /dnas_title="lox66"
```

```
                     /vntifkey="21"
                     /label=lox66
     CDS             2220..2953
                     /dnas_title="DsRed2nls"
                     /vntifkey="4"
                     /label=DsRed2nls

ORIGIN
        1 CTTTCCTGCG TTATCCCCTG ATTCTGTGGA TAACCGTATT ACCGCCTTTG AGTGAGCTGA
       61 TACCGCTCGC CGCAGCCGAA CGACCGAGCG CAGCGAGTCA GTGAGCGAGG AAGCGGAAGA
      121 GCGCCCAATA CGCAAACCGC CTCTCCCCGC GCGTTGGCCG ATTCATTAAT GCAGCTGGCA
      181 CGACAGGTTT CCCGACTGGA AAGCGGGCAG TGAGCGCAAC GCAATTAATA CGCGTACCGC
      241 TAGCATGGAT GTTTTCCCAG TCACGACGTT GTAAAACGAC GGCCAGTCTT AAGCTCGGGC
      301 CCCTACAGGT CACTAATACC ATCTAAGTAG TTGATTCATA GTGACTGGAT ATGTTGTGTT
      361 TTACAGTATT ATGTAGTCTG TTTTTTATGC AAAATCTAAT TTAATATATT GATATTTATA
      421 TCATTTTACG TTTCTCGTTC AACTTTTCTA TACAAAGTTg gtACcggatc cagagaccCG
      481 CAACGCAATT AATGTGAGTT AGCTCACTCA TTAGGCACCC CAGGCTTTAC ACTTTATGCT
      541 TCCGGCTCGT ATGTTGTGTG GAATTGTGAG CGGATAACAA TTTCACACAG GAAACAGCTA
      601 TGACCATGAT TACGCCAAGC GCGCAATTAA CCCTCACTAA AGGGAACAAA AGCTGGAGCT
      661 CCACCGCGGT GGCGGCCGCT CTAGAACTAG TGGATCCCCC GGGCTGCAGG AATTCGATAT
      721 CAAGCTTATC GATACCGTCG ACCTCGAGGG GGGGCCCGGT ACCCAATTCG CCCTATAGTG
      781 AGTCGTATTA CGCGCGCTCA CTGGCCGTCG TTTTACAACG TCGTGACTGG GAAAACCCTG
      841 GCGTTACCCA ACTTAATCGC CTTGCAGCAC ATCCCCCTTT CGCCAGCTGG CGTAATAGCG
      901 AAGAGGCCCG CACCGATCGC CCTTCCCAAC AGTTGCGCAG CCTGAATGGC GAATGGACG
      961 CGCCCTGTAG CGGCGCATTA AGCGCGGCGG GTGTGGTGGT TACGCGCAGC GTGACCGCTA
     1021 CACTTGCCAG CGCCCTAGCG CCCGCTCCTT TCGCTTTCTT CCCTTCCTTT CTCGCCACGT
     1081 TCGCCGGCTT TCCCCGTCAA GCTCTAAATC GGGGGCTCCC TTTAGGGTTC CGATTTAGTG
     1141 CTTTACGGCA CCTCGACCCC AAAAAACTTG ATTAGGGTGA TGGTTCACGT AGTGGGCCAT
     1201 CGCCCTGATA GACGGTTTTT CGCCCTTTGA CGTTGGAGTC CACGTTCTTT AATAGTGGAC
     1261 TCTTGTTCCA AACTGGAACA ACACTCAACC CTATCTCGGT CTATTCTTTT GATTTATAAG
     1321 GGATTTTGCC GATTTCGGCC TATTGGTTAA AAAATGAGCT GATTTAACAA AAATTTAACG
     1381 CGggtctccg cttCTAGACA TAATCAGCCA TACCACATTT GTAGAGGTTT TACTTGCTTT
     1441 AAAAAACCTC CCACACCTCC CCCTGAACCT GAAACATAAA ATGAATGCAA TTGTTGTTGT
     1501 TAACTTGTTT ATTGCAGCTT ATAATGGTTA CAAATAAAGC AATAGCATCA CAAATTTCAC
     1561 AAATAAAGCA TTTTTCTTCA CTGCATTCTA GTTGTGGTTT GTCCAAACTC ATCAATGTAT
     1621 CtcgaCGATG TAGGTCACaG TCTCGAAGCC GCGGTGCGGG TGCCAGGGCG TGCCCTTGGG
     1681 CTCCCCGGGC GCGTACTCCA CCTCACCCAT CTGGTCCATC ATGATGAACG GGTCGAGGTG
     1741 GCGGTAGTTG ATCCCGGCGA ACGCGCGGCG CACCGGGAAG CCCTCGCCCT CGAAACCGCT
     1801 GGGCGCGGTG GTCACGGTGA GCACGGGACG TGCGACGGCG TCGGCGGGTG CGGATACGCG
     1861 GGGCAGCGTC AGCGGGTTCT CGACGGTCAC GGCGGGCAat tCCTGCAGAC TTCCGGTATC
     1921 TCGCGTTTGT TTGATCGCAC GGTTCCCACA ATGGTTAATT CGAGCTCGCC CGGGGATCTA
     1981 ATTCAATTAG AGACTAATTC AATTAGAGCT AATTCAATTA GGATCCAAGC TTATCGATTT
     2041 CGAACCCTCG ACCGCCGGAG TATAAATAGA GGCGCTTCGT CTACGGAGCG ACAATTCAAT
     2101 TCAAACAAGC AAAGTGAACA CGTCGCTAAG CGAAAGCTAA GCAAATAAAC AAGCGCAGCT
     2161 GAACAAGCTA AACAATCGGG GTACCGCTAG AGTCGACGGT ACCGCGGGCC CGGGATCCAc
     2221 cggtcgccac catggcctcc tccgagaacg tcatcaccga gttcatgcgc ttcaaggtgc
     2281 gcatggaggg caccgtgaac ggccacgagt tcgagatcga gggcgagggc gagggccgcc
     2341 cctacgaggg ccacaacacc gtgaagctga aggtgaccaa gggcggcccc ctgcccttcg
     2401 cctgggacat cctgtccccc cagttccagt acggctccaa ggtgtacgtg aagcaccccg
     2461 ccgacatccc cgactacaag aagctgtcct tccccgaggg cttcaagtgg gagcgcgtga
     2521 tgaacttcga ggacggcggc gtggcgaccg tgacccagga ctcctccctg caggacggct
     2581 gcttcatcta caaggtgaag ttcatcggcg tgaacttccc ctccgacggc cccgtgatgc
     2641 agaagaagac catgggctgg gaggcctcca ccgagcgcct gtaccccgc gacggcgtgc
     2701 tgaagggcga gacccacaag gccctgaagc tgaaggacgg cggccactac ctggtggagt
     2761 tcaagtccat ctacatggcc aagaagcccg tgcagctgcc cggctactac tacgtggacg
     2821 ccaagctgga catcacctcc cacaacgagg actacaccat cgtggagcag tacgagcgca
     2881 ccgagggccg ccaccacctg ttcctgagat ctcgacccaa gaaaaagcgg aaggtggagg
     2941 acccgtaaga tccaccggat ctagataact gatcataatc agccatacca catttgtaga
     3001 ggttttactt gctttaaaaa acctcccaca cctccccctg aacctgaaac ataaaatgaa
     3061 tgcaattgtt gttgttaact tgtttattgc agcttataat ggttacaaat aaagcaatag
     3121 catcacaaat ttcacaaata aagcattttt ttcactgcat tctagttgtg gtttgtccaa
     3181 actcatcaat gtatcttatc atgtctggat cccgtttgac ggtatcgata agcttgatgg
     3241 ggatccggaa cccttaatta ccgttcgtat aatgtatgct atacgaagtt attaggtccc
     3301 tcgacctgca gcccGTACGG CGCGCCTAGA GCGGCCGCCA CCGCGGTGGA GCTCGAGTAC
     3361 CCAGCTTTCT TGTACAAAGT TGGCATTATA AGAAAGCATT GCTTATCAAT TTGTTGCAAC
```

```
     3421 GAACAGGTCA CTATCAGTCA AAATAAAATC ATTATTTGCC ATCCAGCTGC AGGGCGGCCG
     3481 CGATATCCCC TATAGTGAGT CGTATTACAT GGTCATAGCT GTTTCCTGGC AGCTCTGGCC
     3541 CGTGTCTCAA AATCTCTGAT GTTACATTGC ACAAGATAAA AATATATCAT CATGAACAAT
     3601 AAAACTGTCT GCTTACATAA ACAGTAATAC AAGGGGTGTT ATGAGCCATA TTCAACGGGA
     3661 AACGTCGAGG CCGCGATTAA ATTCCAACAT GGATGCTGAT TTATATGGGT ATAAATGGGC
     3721 TCGCGATAAT GTCGGGCAAT CAGGTGCGAC AATCTATCGC TTGTATGGGA AGCCCGATGC
     3781 GCCAGAGTTG TTTCTGAAAC ATGGCAAAGG TAGCGTTGCC AATGATGTTA CAGATGAGAT
     3841 GGTCAGACTA AACTGGCTGA CGGAATTTAT GCCTCTTCCG ACCATCAAGC ATTTTATCCG
     3901 TACTCCTGAT GATGCATGGT TACTCACCAC TGCGATCCCC GGAAAAACAG CATTCCAGGT
     3961 ATTAGAAGAA TATCCTGATT CAGGTGAAAA TATTGTTGAT GCGCTGGCAG TGTTCCTGCG
     4021 CCGGTTGCAT TCGATTCCTG TTTGTAATTG TCCTTTTAAC AGCGATCGCG TATTTCGTCT
     4081 CGCTCAGGCG CAATCACGAA TGAATAACGG TTTGGTTGAT GCGAGTGATT TTGATGACGA
     4141 GCGTAATGGC TGGCCTGTTG AACAAGTCTG GAAAGAAATG CATAAACTTT TGCCATTCTC
     4201 ACCGGATTCA GTCGTCACTC ATGGTGATTT CTCACTTGAT AACCTTATTT TTGACGAGGG
     4261 GAAATTAATA GGTTGTATTG ATGTTGGACG AGTCGGAATC GCAGACCGAT ACCAGGATCT
     4321 TGCCATCCTA TGGAACTGCC TCGGTGAGTT TTCTCCTTCA TTACAGAAAC GGCTTTTTCA
     4381 AAAATATGGT ATTGATAATC CTGATATGAA TAAATTGCAG TTTCATTTGA TGCTCGATGA
     4441 GTTTTTCTAA TCAGAATTGG TTAATTGGTT GTAACACTGG CAGAGCATTA CGCTGACTTG
     4501 ACGGGACGGC GCAAGCTCAT GACCAAAATC CCTTAACGTG AGTTACGCGT CGTTCCACTG
     4561 AGCGTCAGAC CCCGTAGAAA AGATCAAAGG ATCTTCTTGA GATCCTTTTT TTCTGCGCGT
     4621 AATCTGCTGC TTGCAAACAA AAAAACCACC GCTACCAGCG GTGGTTTGTT TGCCGGATCA
     4681 AGAGCTACCA ACTCTTTTTC CGAAGGTAAC TGGCTTCAGC AGAGCGCAGA TACCAAATAC
     4741 TGTTCTTCTA GTGTAGCCGT AGTTAGGCCA CCACTTCAAG AACTCTGTAG CACCGCCTAC
     4801 ATACCTCGCT CTGCTAATCC TGTTACCAGT GGCTGCTGCC AGTGGCGATA AGTCGTGTCT
     4861 TACCGGGTTG GACTCAAGAC GATAGTTACC GGATAAGGCG CAGCGGTCGG GCTGAACGGG
     4921 GGGTTCGTGC ACACAGCCCA GCTTGGAGCG AACGACCTAC ACCGAACTGA GATACCTACA
     4981 GCGTGAGCTA TGAGAAAGCG CCACGCTTCC CGAAGGGAGA AAGGCGGACA GGTATCCGGT
     5041 AAGCGGCAGG GTCGGAACAG GAGAGCGCAC GAGGGAGCTT CCAGGGGGAA ACGCCTGGTA
     5101 TCTTTATAGT CCTGTCGGGT TTCGCCACCT CTGACTTGAG CGTCGATTTT TGTGATGCTC
     5161 GTCAGGGGGG CGGAGCCTAT GGAAAAACGC CAGCAACGCG GCCTTTTTAC GGTTCCTGGC
     5221 CTTTTGCTGG CCTTTTGCTC ACATGTT
//
```

pattB-RfB2 (ampicillin resistant attB site-containing vector for docking site-based transgenesis, compatible with Gateway and Multisite Gateway cloning. Transform a *ccdB* resistant *E. coli* strain!)

```
LOCUS       pattB-RfB2              4884 bp    DNA     circular
FEATURES             Location/Qualifiers
     misc_feature    2385..2669
                     /note="attB"
     misc_feature    complement(2693..2712)
                     /note="T3"
     misc_feature    complement(2729..2747)
                     /note="M13R"
     misc_feature    622..642
                     /note="T7"
     misc_feature    complement(661..2373)
                     /note="RfB Gateway cassette"
     misc_feature    666..789
                     /note="attR2"
     misc_feature    complement(2245..2369)
                     /note="attR1"
     misc_feature    600..616
                     /note="M13F"
     source          1..4884
                     /dnas_title="pattB-RfB2"

ORIGIN
        1 CTAAATTGTA AGCGTTAATA TTTTGTTAAA ATTCGCGTTA AATTTTTGTT AAATCAGCTC
       61 ATTTTTTAAC CAATAGGCCG AAATCGGCAA AATCCCTTAT AAATCAAAAG AATAGACCGA
```

121 GATAGGGTTG AGTGTTGTTC CAGTTTGGAA CAAGAGTCCA CTATTAAAGA ACGTGGACTC
181 CAACGTCAAA GGGCGAAAAA CCGTCTATCA GGGCGATGGC CCACTACGTG AACCATCACC
241 CTAATCAAGT TTTTTGGGGT CGAGGTGCCG TAAAGCACTA AATCGGAACC CTAAAGGGAG
301 CCCCCGATTT AGAGCTTGAC GGGGAAAGCC GGCGAACGTG GCGAGAAAGG AAGGGAAGAA
361 AGCGAAAGGA GCGGGCGCTA GGGCGCTGGC AAGTGTAGCG GTCACGCTGC GCGTAACCAC
421 CACACCCGCC GCGCTTAATG CGCCGCTACA GGGCGCGTCC CATTCGCCAT TCAGGCTGCG
481 CAACTGTTGG GAAGGGCGAT CGGTGCGGGC CTCTTCGCTA TTACGCCAGC TGGCGAAAGG
541 GGGATGTGCT GCAAGGCGAT TAAGTTGGGT AACGCCAGGG TTTTCCCAGT CACGACGTTG
601 TAAAACGACG GCCAGTGAAT TGTAATACGA CTCACTATAG GGCGAATTGG GTACGTACCG
661 atcaaccact ttgtacaaga aagctgaacg agaaacgtaa aatgatataa atatcaatat
721 attaaattag attttgcata aaaaacagac tacataatac tgtaaaacac aacatatcca
781 gtcactatgg tcgacctgca gactggctgt gtataaggga gcctgacatt tatattcccc
841 agaacatcag gttaatggcg tttttgatgt cattttcgcg gtggctgaga tcagccactt
901 cttccccgat aacggagacc ggcacactgg ccatatcggt ggtcatcatg cgccagcttt
961 catccccgat atgcaccacc gggtaaagtt cacgggagac tttatctgac agcagacgtg
1021 cactggccag ggggatcacc atccgtcgcc cgggcgtgtc aataatatca ctctgtacat
1081 ccacaaacag acgataacgg ctctctcttt tataggtgta aaccttaaac tgcatttcac
1141 cagcccctgt tctcgtcagc aaaagagccg ttcatttcaa taaaccgggc gacctcagcc
1201 atcccttcct gattttccgc tttccagcgt tcggcacgca gacgacgggc ttcattctgc
1261 atggttgtgc ttaccagacc ggagatattg acatcatata tgccttgagc aactgatagc
1321 tgtcgctgtc aactgtcact gtaatacgct gcttcatagc atacctcttt ttgacatact
1381 tcgggtatac atatcagtat atattcttat accgcaaaaa tcagcgcgca aatacgcata
1441 ctgttatctg gcttttagta agccggatcc agatctttac gccccgccct gccactcatc
1501 gcagtactgt tgtaattcat taagcattct gccgacatgg aagccatcac aaacggcatg
1561 atgaacctga atcgccagcg gcatcagcac cttgtcgcct tgcgtataat atttgcccat
1621 ggtgaaaacg ggggcgaaga agttgtccat attggccacg tttaaatcaa aactggtgaa
1681 actcacccag ggattggctg agacgaaaaa catattctca ataaaccctt tagggaaata
1741 ggccaggttt tcaccgtaac acgccacatc ttgcgaatat atgtgtagaa actgccggaa
1801 atcgtcgtgg tattcactcc agagcgatga aaacgtttca gtttgctcat ggaaaacggt
1861 gtaacaaggg tgaacactat cccatatcac cagctcaccg tctttcattg ccatacggaa
1921 ttccggatga gcattcatca ggcgggcaag aatgtgaata aaggccggat aaaacttgtg
1981 cttatttttc tttacggtct ttaaaaaggc cgtaatatcc agctgaacgg tctggttata
2041 ggtacattga gcaactgact gaaatgcctc aaaatgttct ttacgatgcc attgggatat
2101 atcaacggtg gtatatccag tgattttttt ctccatttta gcttccttag ctcctgaaaa
2161 tctcgacgga tcctaactca aaatccacac attatacgag ccggaagcat aaagtgtaaa
2221 gcctggggtg cctaatgcgg ccgccatagt gactggatat gttgtgtttt acagtattat
2281 gtagtctgtt ttttatgcaa aatctaattt aatatattga tatttatatc attttacgtt
2341 tctcgttcag ctttttttgta caaacttgtt gatgatcCAC TAGTGTCGAC GATGTAGGTC
2401 ACGGTCTCGA AGCCGCGGTG CGGGTGCCAG GGCGTGCCCT TGGGCTCCCC GGGCGCGTAC
2461 TCCACCTCAC CCATCTGGTC CATCATGATG AACGGGTCGA GGTGGCGGTA GTTGATCCCG
2521 GCGAACGCGC GGCGCACCGG GAAGCCCTCG CCCTCGAAAC CGCTGGGCGC GGTGGTCACG
2581 GTGAGCACGG GACGTGCGAC GGCGTCGGCG GGTGCGGATA CGCGGGGCAG CGTCAGCGGG
2641 TTCTCGACGG TCACGGCGGG CATGTCGACA CTAGTTCTAG CCAGCTTTTG TTCCCTTTAG
2701 TGAGGGTTAA TTTCGAGCTT GGCGTAATCA TGGTCATAGC TGTTTCCTGT GTGAAATTGT
2761 TATCCGCTCA CAATTCCACA CAACATACGA GCCGGAAGCA TAAAGTGTAA AGCCTGGGGT
2821 GCCTAATGAG TGAGCTAACT CACATTAATT GCGTTGCGCT CACTGCCCGC TTTCCAGTCG
2881 GGAAACCTGT CGTGCCAGCT GCATTAATGA ATCGGCCAAC GCGCGGGGAG AGGCGGTTTG
2941 CGTATTGGGC GCTCTTCCGC TTCCTCGCTC ACTGACTCGC TGCGCTCGGT CGTTCGGCTG
3001 CGGCGAGCGG TATCAGCTCA CTCAAAGGCG GTAATACGGT TATCCACAGA ATCAGGGGAT
3061 AACGCAGGAA AGAACATGTG AGCAAAAGGC CAGCAAAAGG CCAGGAACCG TAAAAAGGCC
3121 GCGTTGCTGG CGTTTTTCCA TAGGCTCCGC CCCCCTGACG AGCATCACAA AAATCGACGC
3181 TCAAGTCAGA GGTGGCGAAA CCCGACAGGA CTATAAAGAT ACCAGGCGTT TCCCCCTGGA
3241 AGCTCCCTCG TGCGCTCTCC TGTTCCGACC CTGCCGCTTA CCGGATACCT GTCCGCCTTT
3301 CTCCCTTCGG GAAGCGTGGC GCTTTCTCAT AGCTCACGCT GTAGGTATCT CAGTTCGGTG
3361 TAGGTCGTTC GCTCCAAGCT GGGCTGTGTG CACGAACCCC CCGTTCAGCC CGACCGCTGC
3421 GCCTTATCCG GTAACTATCG TCTTGAGTCC AACCCGGTAA GACACGACTT ATCGCCACTG
3481 GCAGCAGCCA CTGGTAACAG GATTAGCAGA GCGAGGTATG TAGGCGGTGC TACAGAGTTC
3541 TTGAAGTGGT GGCCTAACTA CGGCTACACT AGAAGGACAG TATTTGGTAT CTGCGCTCTG
3601 CTGAAGCCAG TTACCTTCGG AAAAAGAGTT GGTAGCTCTT GATCCGGCAA ACAAACCACC
3661 GCTGGTAGCG GTGGTTTTTT TGTTTGCAAG CAGCAGATTA CGCGCAGAAA AAAAGGATCT
3721 CAAGAAGATC CTTTGATCTT TTCTACGGGG TCTGACGCTC AGTGGAACGA AAACTCACGT
3781 TAAGGGATTT TGGTCATGAG ATTATCAAAA AGGATCTTCA CCTAGATCCT TTTAAATTAA
3841 AAATGAAGTT TTAAATCAAT CTAAAGTATA TATGAGTAAA CTTGGTCTGA CAGTTACCAA
3901 TGCTTAATCA GTGAGGCACC TATCTCAGCG ATCTGTCTAT TTCGTTCATC CATAGTTGCC
3961 TGACTCCCCG TCGTGTAGAT AACTACGATA CGGGAGGGCT TACCATCTGG CCCCAGTGCT

```
     4021 GCAATGATAC CGCGAGACCC ACGCTCACCG GCTCCAGATT TATCAGCAAT AAACCAGCCA
     4081 GCCGGAAGGG CCGAGCGCAG AAGTGGTCCT GCAACTTTAT CCGCCTCCAT CCAGTCTATT
     4141 AATTGTTGCC GGGAAGCTAG AGTAAGTAGT TCGCCAGTTA ATAGTTTGCG CAACGTTGTT
     4201 GCCATTGCTA CAGGCATCGT GGTGTCACGC TCGTCGTTTG GTATGGCTTC ATTCAGCTCC
     4261 GGTTCCCAAC GATCNAAGGC GAGTTACATG ATNCCCCCAT GTTGTGCAAA AAAGCGGTTA
     4321 GCTCCTTCGG TCCTCCGATC GTTGTCAGAA GTAAGTTGGC CGCAGTGTTA TCACTCATGG
     4381 TTATGGCAGC ACTGCATAAT TCTCTTACTG TCATGCCATC CGTAAGATGC TTTTCTGTGA
     4441 CTGGTGAGTA CTCAACCAAG TCATTCTGAG AATAGTGTAT GCGGCGACCG AGTTGCTCTT
     4501 GCCCGGCGTC AATACGGGAT AATACCGCGC CACATAGCAG AACTTTAAAA GTGCTCATCA
     4561 TTGGAAAACG TTCTTCGGGG CGAAAACTCT CAAGGATCTT ACCGCTGTTG AGATCCAGTT
     4621 CGATGTAACC CACTCGTGCA CCCAACTGAT CTTCAGCATC TTTTACTTTC ACCAGCGTTT
     4681 CTGGGTGAGC AAAAACAGGA AGGCAAAATG CCGCAAAAAA GGGAATAAGG GCGACACGGA
     4741 AATGTTGAAT ACTCATACTC TTCCTTTTTC AATATTATTG AAGCATTTAT CAGGGTTATT
     4801 GTCTCATGAG CGGATACATA TTTGAATGTA TTTAGAAAAA TAAACAAATA GGGGTTCCGC
     4861 GCACATTTCC CCGAAAAGTG CCAC

//
```

<u>pXLtTloxattPGat2 (ampicillin resistant *piggyBac* transgenesis vector with transposase in backbone and Gateway cloning cassette. Ampicillin resistant):</u>

```
LOCUS       pXLBacIItTloxatt        8692 bp    DNA     circular
FEATURES             Location/Qualifiers
     misc_feature    3718..3751
                     /note="loxP"
     misc_feature    5522..5742
                     /note="attP'"
     misc_feature    complement(6539..6557)
                     /note="M13R"
     misc_feature    3322..3338
                     /note="M13F"
     misc_feature    complement(339..2405)
                     /note="transposase"
     misc_feature    complement(2462..3049)
                     /note="hsp70 promoter"
     misc_feature    complement(2407..2462)
                     /note="rrnB terminator"
     misc_feature    5478..5511
                     /note="loxP"
     misc_feature    complement(3933..4238)
                     /note="ccdB ORF"
     misc_feature    3768..3892
                     /note="attR2"
     misc_feature    complement(4580..5239)
                     /note="chloramphenicol resistance ORF"
     misc_feature    complement(5348..5472)
                     /note="attR1"
     misc_feature    complement(3468..3625)
                     /note="piggyBac 3'region"
     misc_feature    complement(3768..5472)
                     /note="Gateway cassette"
     misc_feature    5917..6053
                     /note="piggyBac 5' region"
     source          1..8692
                     /dnas_title="pXLBacIItTloxatt"
ORIGIN
        1 CTAAATTGTA AGCGTTAATA TTTTGTTAAA ATTCGCGTTA AATTTTTGTT AAATCAGCTC
       61 ATTTTTTAAC CAATAGGCCG AAATCGGCAA AATCCCTTAT AAATCAAAAG AATAGACCGA
      121 GATAGGGTTG AGTGTTGTTC CAGTTTGGAA CAAGAGTCCA CTATTAAAGA ACGTGGACTC
      181 CAACGTCAAA GGGCGAAAAA CCGTCTATCA GGGCGATGGC CCACTACGTG AACCATCACC
      241 CTAATCAAGT TTTTTGGGGT CGAGGTGCCG TAAAGCACTA AATCGGAACC CTAAAGGGAG
      301 CCCCCGATTT AGAGCTTGAC GGGGAAAGCC TCGACGGATC CAAATTCAAC AAACAATTTA
      361 TGTTTATTTA TTTATTAAAA AAAAACAAAA ACTCAAAATT TCTTCTATAA AGTAACAAAA
```

```
 421 CTTTTAAACA TTCTCTCCTT TACAAAAATA AACTTATTTT GTACTTTAAA AACAGTCATG
 481 TTGTATTATA AAATAAGTAA TTAGTTTAAC TTATACATAA TAGAAACAAA TTATACTTAT
 541 TAGTCAGTCA GAAACAACTT TGGCACATAT CAATATTATG CTCTCGACAA ATAACTTTTT
 601 TGCATTTTTT GCACGATGCA TTTGCCTTTC GCCTTATTTT AGAGGGGCAG TAAGTACAGT
 661 AAGTACGTTT TTTCATTACT GGCTCTTCAG TACTGTCATC TGATGTACCA GGCACTTCAT
 721 TTGGCAAAAT ATTAGAGATA TTATCGCGCA AATATCTCTT CAAAGTAGGA GCTTCTAAAC
 781 GCTTACGCAT AAACGATGAC GTCAGGCTCA TGTAAAGGTT TCTCATAAAT TTTTTGCGAC
 841 TTTGAACCTT TTCTCCCTTG CTACTGACAT TATGGCTGTA TATAATAAAA GAATTTATGC
 901 AGGCAATGTT TATCATTCCG TACAATAATG CCATAGGCCA CCTATTCGTC TTCCTACTGC
 961 AGGTCATCAC AGAACACATT TGGTCTAGCG TGTCCACTCC GCCTTTAGTT TGATTATAAT
1021 ACATAACCAT TTGCGGTTTA CCGGTACTTT CGTTGATAGA AGCATCCTCA TCACAAGATG
1081 ATAATAAGTA TACCATCTTA GCTGGCTTCG GTTTATATGA GACGAGAGTA AGGGGTCCGT
1141 CAAAACAAAA CATCGATGTT CCCACTGGCC TGGAGCGACT GTTTTTCAGT ACTTCCGGTA
1201 TCTCGCGTTT GTTTGATCGC ACGGTTCCCA CAATGGTTAA CTTATACGGT TCTTGTAGTA
1261 AGTTTTTTGC CAAAGGGATT GAGGTGAACC AATTGTCACA CGTAATATTA CGACAACTAC
1321 CGTGCACAGG CTTTGATAAC TCCTTCACGT AGTATTCACC GAGTGGTACT CCGTTGGTCT
1381 GTGTTCCTCT TCCCAAATAA GGCATTCCAT TTATCATATA CTTCGTACCA CTGTCACACA
1441 TCATGAGGAT TTTTATTCCA TACTTACTTG GCTTGTTTGG GATATACATC CTAAACGGAC
1501 ACCGTCCTCT AAAACCAAGT AACTGTTCAT CTATGGTCAA ATGAGCCCCT GGAGTGTAAT
1561 TTTGTATGCA CTGATGGATA AAGAGATCCC ATATTTTTCT AACAGGAGTA AATACATCGT
1621 TTTCTCGAAG TGTGGGCCGT ATACTTTTGT CATCCATTCT AAGACATCGT ATCAAAAAAT
1681 CAAAACGATC ACGACTCATT ACAGAGACGT ACACCATTGA CAAAGATCGA TCAAAGAGGT
1741 CATCTGTGGA CATGTGGTTA TCTTTTCTCA CTGCTGTCAT TACCAGAATA CCAAAGAAAG
1801 CATAGATTTC ATCTTCATTC GTGTCACGAA ATGTAGCACC TGTCATAGAT TCCCGACGTT
1861 TCAATGATAT CTCAGCATTT GTCCATTTTA CAATTTCCGA AATTATCTCA TCAGTAAAAA
1921 ATAGTTTGAA GCATAAAAGT GGGTCATATA TATTGCGGCA CATACGCGTC GGACCTCTTT
1981 GAGATCTGAC AATGTTCAGT GCAGAGACTC GGCTACGCCT CGTGGACTTT GAAGTTGACC
2041 AACAATGTTT ATTCTTACCT CTAATAGTCC TCTGTGGCAA GGTCAAGATT CTGTTAGAAG
2101 CCAATGAAGA ACCTGGTTGT TCAATAACAT TTTGTTCGTC TAATATTTCA CTACCGCTTG
2161 ACGTTGGCTG CACTTCATGT ACCTCATCTA TAAACGCTTC TTCTGTATCG CTCTGGACGT
2221 CATCTTCACT TACGTGATCT GATATTTCAC TGTCAGAATC CTCACCAACA AGCTCGTCAT
2281 CGCTTTGCAG AAGAGCAGAG AGGATATGCT CATCGTCTAA AGAACTACCC ATTTTATTAT
2341 ATAGGATCCC CGACACCAGA CCAACTGGTA ATGGTAGCGA CCGGCGCTCA GCTGGAATTA
2401 GGCCTTCTAG acagataaaa cgaaaggccc agtctttcga ctgagccttt cgttttattt
2461 gAAATTCCCA ATTCCCTATT CAGAGTTCTC TTCTTGTATT CAATAATTAC TTCTTGGCAG
2521 ATTTCAGTAG TTGCAGTTGA TTTACTTGGT TGCTGGTTAC TTTTAATTGA TTCACTTTAA
2581 CTTGCACTTT ACTGCAGATT GTTTAGCTTG TTCAGCTGCG CTTGTTTATT TGCTTAGCTT
2641 TCGCTTAGCG ACGTGTTCAC TTTGCTTGTT TGAATTGAAT TGTCGCTCCG TAGACGAAGC
2701 GCCTCTATTT ATACTCCGGC GCTCTTTTCG CGAACATTCG AGGCGCGCTC TCTCGAACCA
2761 ACGAGAGCAG TATGCCGTTT ACTGTGTGAC AGAGTGAGAG AGCATTAGTG CAGAGAGGGA
2821 GAGACCCAAA AAGAAAAGAG AGAATAACGA ATAACGGCCA GAGAAATTTC TCGAGTTTTC
2881 TTTCTGCCAA ACAAATGACC TACCACAATA ACCAGTTTGT TTTGGGATTC TAGGGGGATC
2941 GGGGATCAAT TCTAGTATGT ATGTAAGTTA ATAAAACCCA TTTTTGCGGA AAGTAGATAA
3001 AAAAAACATT TTTTTTTTTT ACTGCACTGG ATATCATTGA ACTTATCTGA TCGGCGAACG
3061 TGGCGAGAAA GGAAGGGAAG AAAGCGAAAG GAGCGGGCGC TAGGGCGCTG GCAAGTGTAG
3121 CGGTCACGCT GCGCGTAACC ACCACACCCG CCGCGCTTAA TGCGCCGCTA CAGGGCGCGT
3181 CCCATTCGCC ATTCAGGCTG CGCAACTGTT GGGAAGGGCG ATCGGTGCGG GCCTCTTCGC
3241 TATTACGCCA GCTGGCGAAA GGGGGATGTG CTGCAAGGCG ATTAAGTTGG GTAACGCCAG
3301 GGTTTTCCCA GTCACGACGT TGTAAAACGA CGGCCAGTGA GCGCGCCTCG TTCATTCACG
3361 TTTTTGAACC CGTGGAGGAC GGGCAGACTC GCGGTGCAAA TGTGTTTTAC AGCGTGATGG
3421 AGCAGATGAA GATGCTCGAC ACGCTGCAGA ACACGCAGCT AGATTAACCC TAGAAAGATA
3481 ATCATATTGT GACGTACGTT AAAGATAATC ATGCGTAAAA TTGACGCATG TGTTTTATCG
3541 GTCTGTATAT CGAGGTTTAT TTATTAATTT GAATAGATAT TAAGTTTTAT TATATTTACA
3601 CTTACATACT AATAATAAAT TCAACAAACA ATTTATTTAT GTTTATTTAT TTATTAAAAA
3661 AAACAAAAAC TCAAAATTTC TTCTATAAAG TAACAAAACT TTTATCGAAT TGAGCTCata
3721 acttcgtata atgtatgcta tacgaagtta tGTCGACGAA TTACATCACC ACTTTGTACA
3781 AGAAAGCTGA ACGAGAAACG TAAAATGATA TAAATATCAA TATATTAAAT TAGATTTTGC
3841 ATAAAAAACA GACTACATAA TACTGTAAAA CACAACATAT CCAGTCACTA TGGTCGACCT
3901 GCAGACTGGC TGTGTATAAG GGAGCCTGAC ATTTATATTC CCCAGAACAT CAGGTTAATG
3961 GCGTTTTTGA TGTCATTTTC GCGGTGGCTG AGATCAGCCA CTTCTTCCCC GATAACGGAG
4021 ACCGGCACAC TGGCCATATC GGTGGTCATC ATGCGCCAGC TTTCATCCCC GATATGCACC
4081 ACCGGGTAAA GTTCACGGGA GACTTTATCT GACAGCAGAC GTGCACTGGC CAGGGGGATC
4141 ACCATCCGTC GCCCGGGCGT GTCAATAATA TCACTCTGTA CATCCACAAA CAGACGATAA
4201 CGGCTCTCTC TTTTATAGGT GTAAACCTTA AACTGCATTT CACCAGCCCC TGTTCTCGTC
4261 AGCAAAAGAG CCGTTCATTT CAATAAACCG GGCGACCTCA GCCATCCCTT CCTGATTTTC
```

```
4321 CGCTTTCCAG CGTTCGGCAC GCAGACGACG GGCTTCATTC TGCATGGTTG TGCTTACCAG
4381 ACCGGAGATA TTGACATCAT ATATGCCTTG AGCAACTGAT AGCTGTCGCT GTCAACTGTC
4441 ACTGTAATAC GCTGCTTCAT AGCATACCTC TTTTTGACAT ACTTCGGGTA TACATATCAG
4501 TATATATTCT TATACCGCAA AAATCAGCGC GCAAATACGC ATACTGTTAT CTGGCTTTTA
4561 GTAAGCCGGA TCCACGCGTT TACGCCCCGC CCTGCCACTC ATCGCAGTAC TGTTGTAATT
4621 CATTAAGCAT TCTGCCGACA TGGAAGCCAT CACAAACGGC ATGATGAACC TGAATCGCCA
4681 GCGGCATCAG CACCTTGTCG CCTTGCGTAT AATATTTGCC CATGGTGAAA ACGGGGGCGA
4741 AGAAGTTGTC CATATTGGCC ACGTTTAAAT CAAAACTGGT GAAACTCACC CAGGGATTGG
4801 CTGAGACGAA AAACATATTC TCAATAAACC CTTTAGGGAA ATAGGCCAGG TTTTCACCGT
4861 AACACGCCAC ATCTTGCGAA TATATGTGTA GAAACTGCCG GAAATCGTCG TGGTATTCAC
4921 TCCAGAGCGA TGAAAACGTT TCAGTTTGCT CATGGAAAAC GGTGTAACAA GGGTGAACAC
4981 TATCCCATAT CACCAGCTCA CCGTCTTTCA TTGCCATACG GAATTCCGGA TGAGCATTCA
5041 TCAGGCGGGC AAGAATGTGA ATAAAGGCCG GATAAAACTT GTGCTTATTT TTCTTTACGG
5101 TCTTTAAAAA GGCCGTAATA TCCAGCTGAA CGGTCTGGTT ATAGGTACAT TGAGCAACTG
5161 ACTGAAATGC CTCAAAATGT TCTTTACGAT GCCATTGGGA TATATCAACG GTGGTATATC
5221 CAGTGATTTT TTTCTCCATT TTAGCTTCCT TAGCTCCTGA AAATCTCGAC GGATCCTAAC
5281 TCAAAATCCA CACATTATAC GAGCCGGAAG CATAAAGTGT AAAGCCTGGG GTGCCTAATG
5341 CGGCCGCCAT AGTGACTGGA TATGTTGTGT TTTACAGTAT TATGTAGTCT GTTTTTTATG
5401 CAAAATCTAA TTTAATATAT TGATATTTAT ATCATTTTAC GTTTCTCGTT CAGCTTTTTT
5461 GTACAAACTT GTGATGTata acttcgtata atgtatgcta tacgaagtta tGGTACCTGC
5521 AGTACTGACG GACACACCGA AGCCCCGGCG GCAACCCTCA GCGGATGCCC CGGGGCTTCA
5581 CGTTTTCCCA GGTCAGAAGC GGTTTTCGGG AGTAGTGCCC CAACTGGGGT AACCTTTGAG
5641 TTCTCTCAGT TGGGGGCGTA GGGTCGCCGA CATGACACAA GGGGTTGTGA CCGGGGTGGA
5701 CACGTACGCG GGTGCTTACG ACCGTCAGTC GCGCGAGCGC GATCTAATCT ATAACAAGAA
5761 AATATATATA TAATAAGTTA TCACGTAAGT AGAACATGAA ATAACAATAT AATTATCGTA
5821 TGAGTTAAAT CTTAAAAGTC ACGTAAAAGA TAATCATGCG TCATTTTGAC TCACGCGGTC
5881 GTTATAGTTC AAAATCAGTG ACACTTACCG CATTGACAAG CACGCCTCAC GGGAGCTCCA
5941 AGCGGCGACT GAGATGTCCT AAATGCACAG CGACGGATTC GCGCTATTTA GAAAGAGAGA
6001 GCAATATTTC AAGAATGCAT GCGTCAATTT TACGCAGACT ATCTTTCTAG GGTTAATCTA
6061 GCTGCATCAG GATCATATCG TCGGGTCTTT TTTCCGGCTC AGTCATCGCC CAAGCTGGCG
6121 CTATCTGGGC ATCGGGGAGG AAGAAGCCCG TGCCTTTTCC CGCGAGGTTG AAGCGGCATG
6181 GAAAGAGTTT GCCGAGGATG ACTGCTGCTG CATTGACGTT GAGCGAAAAC GCACGTTTAC
6241 CATGATGATT CGGGAAGGTG TGGCCATGCA CGCCTTTAAC GGTGAACTGT TCGTTCAGGC
6301 CACCTGGGAT ACCAGTTCGT CGCGGCTTTT CCGGACACAG TTCCGGATGG TCAGCCCGAA
6361 GCGCATCAGC AACCCGAACA ATACCGGCGA CAGCCGGAAC TGCCGTGCCG GTGTGCAGAT
6421 TAATGACAGC GGTGCGGCGC TGGGATATTA CGTCAGCGAG GACGGGTATC CTGGCTGGAT
6481 GCCGCAGAAA TGGACATGGA TACCCCGTGA GTTACCCGGC GGGCGCGCTT GGCGTAATCA
6541 TGGTCATAGC TGTTTCCTGT GTGAAATTGT TATCCGCTCA CAATTCCACA CAACATACGA
6601 GCCGGAAGCA TAAAGTGTAA AGCCTGGGGT GCCTAATGAG TGAGCTAACT CACATTAATT
6661 GCGTTGCGCT CACTGCCCGC TTTCCAGTCG GGAAACCTGT CGTGCCAGCT GCATTAATGA
6721 ATCGGCCAAC GCGCGGGGAG AGGCGGTTTG CGTATTGGGC GCTCTTCCGC TTCCTCGCTC
6781 ACTGACTCGC TGCGCTCGGT CGTTCGGCTG CGGCGAGCGG TATCAGCTCA CTCAAAGGCG
6841 GTAATACGGT TATCCACAGA ATCAGGGGAT AACGCAGGAA AGAACATGTG AGCAAAAGGC
6901 CAGCAAAAGG CCAGGAACCG TAAAAAGGCC GCGTTGCTGG CGTTTTTCCA TAGGCTCCGC
6961 CCCCCTGACG AGCATCACAA AAATCGACGC TCAAGTCAGA GGTGGCGAAA CCCGACAGGA
7021 CTATAAAGAT ACCAGGCGTT TCCCCCTGGA AGCTCCCTCG TGCGCTCTCC TGTTCCGACC
7081 CTGCCGCTTA CCGGATACCT GTCCGCCTTT CTCCCTTCGG GAAGCGTGGC GCTTTCTCAT
7141 AGCTCACGCT GTAGGTATCT CAGTTCGGTG TAGGTCGTTC GCTCCAAGCT GGGCTGTGTG
7201 CACGAACCCC CCGTTCAGCC CGACCGCTGC GCCTTATCCG GTAACTATCG TCTTGAGTCC
7261 AACCCGGTAA GACACGACTT ATCGCCACTG GCAGCAGCCA CTGGTAACAG GATTAGCAGA
7321 GCGAGGTATG TAGGCGGTGC TACAGAGTTC TTGAAGTGGT GGCCTAACTA CGGCTACACT
7381 AGAAGGACAG TATTTGGTAT CTGCGCTCTG CTGAAGCCAG TTACCTTCGG AAAAAGAGTT
7441 GGTAGCTCTT GATCCGGCAA ACAAACCACC GCTGGTAGCG GTGGTTTTTT TGTTTGCAAG
7501 CAGCAGATTA CGCGCAGAAA AAAAGGATCT CAAGAAGATC CTTTGATCTT TTCTACGGGG
7561 TCTGACGCTC AGTGGAACGA AAACTCACGT TAAGGGATTT TGGTCATGAG ATTATCAAAA
7621 AGGATCTTCA CCTAGATCCT TTTAAATTAA AAATGAAGTT TTAAATCAAT CTAAAGTATA
7681 TATGAGTAAA CTTGGTCTGA CAGTTACCAA TGCTTAATCA GTGAGGCACC TATCTCAGCG
7741 ATCTGTCTAT TTCGTTCATC CATAGTTGCC TGACTCCCCG TCGTGTAGAT AACTACGATA
7801 CGGGAGGGCT TACCATCTGG CCCCAGTGCT GCAATGATAC CGCGAGACCC ACGCTCACCG
7861 GCTCCAGATT TATCAGCAAT AAACCAGCCA GCCGGAAGGG CCGAGCGCAG AAGTGGTCCT
7921 GCAACTTTAT CCGCCTCCAT CCAGTCTATT AATTGTTGCC GGGAAGCTAG AGTAAGTAGT
7981 TCGCCAGTTA ATAGTTTGCG CAACGTTGTT GCCATTGCTA CAGGCATCGT GGTGTCACGC
8041 TCGTCGTTTG GTATGGCTTC ATTCAGCTCC GGTTCCCAAC GATCAAGGCG AGTTACATGA
8101 TCCCCCATGT TGTGCAAAAA AGCGGTTAGC TCCTTCGGTC CTCCGATCGT TGTCAGAAGT
8161 AAGTTGGCCG CAGTGTTATC ACTCATGGTT ATGGCAGCAC TGCATAATTC TCTTACTGTC
```

```
     8221 ATGCCATCCG TAAGATGCTT TTCTGTGACT GGTGAGTACT CAACCAAGTC ATTCTGAGAA
     8281 TAGTGTATGC GGCGACCGAG TTGCTCTTGC CCGGCGTCAA TACGGGATAA TACCGCGCCA
     8341 CATAGCAGAA CTTTAAAAGT GCTCATCATT GGAAAACGTT CTTCGGGGCG AAAACTCTCA
     8401 AGGATCTTAC CGCTGTTGAG ATCCAGTTCG ATGTAACCCA CTCGTGCACC CAACTGATCT
     8461 TCAGCATCTT TTACTTTCAC CAGCGTTTCT GGGTGAGCAA AAACAGGAAG GCAAAATGCC
     8521 GCAAAAAAGG GAATAAGGGC GACACGGAAA TGTTGAATAC TCATACTCTT CCTTTTTCAA
     8581 TATTATTGAA GCATTTATCA GGGTTATTGT CTCATGAGCG GATACATATT TGAATGTATT
     8641 TAGAAAAATA AACAAATAGG GGTTCCGCGC ACATTTCCCC GAAAAGTGCC AC
//
```

pENTR R4-vas2-integrase-R3 (helper plasmid expressing phage ΦC31 integrase under the control of the vasa promoter). Kanamycin resistant

```
LOCUS       pENTRR4-Vas2-INT-R3        6847 bp    DNA     circular
FEATURES             Location/Qualifiers
     misc_feature    complement(603..727)
                     /dnas_title="attR4"
                     /vntifkey=21
                     /label=attR4
                     /note="attR4"
     misc_feature    6052..6176
                     /dnas_title="attR3"
                     /vntifkey=21
                     /label=attR3
                     /note="attR3"
     misc_feature    complement(6295..6313)
                     /dnas_title="pDONR-RP"
                     /vntifkey=21
                     /label=pDONR-RP
                     /note="pDONR-RP"
     misc_feature    537..552
                     /dnas_title="M13F"
                     /vntifkey=21
                     /label=M13F
                     /note="M13F"
     misc_feature    760..3050
                     /dnas_title="Vasa promoter"
                     /vntifkey=21
                     /label=Vasa promoter
     misc_feature    5046..6046
                     /dnas_title="3'UTR_Vasa"
                     /vntifkey=21
                     /label=3'UTR_Vasa
     misc_feature    3213..5030
                     /note="integrase"
     misc_feature    3157..3181
                     /note="HA"
     misc_feature    3121..3134
                     /note="NLS"
     misc_feature    complement(6251..6269)
                     /note="M13R"

BASE COUNT     1986 a   1866 c   2060 g   2078 t
ORIGIN
        1 ctttcctgcg ttatcccctg attctgtgga taaccgtatt accgcctttg agtgagctga
       61 taccgctcgc cgcagccgaa cgaccgagcg cagcgagtca gtgagcgagg aagcggaaga
      121 gcgcccaata cgcaaaccgc ctctccccgc gcgttggccg attcattaat gcagctggca
      181 cgacaggttt cccgactgga aagcgggcag tgagcgcaac gcaattaata cgcgtaccgc
      241 tagccaggaa gagtttgtag aaacgcaaaa aggccatccg tcaggatggc cttctgctta
      301 gtttgatgcc tggcagttta tggcgggcgt cctgcccgcc accctccggg ccgttgcttc
      361 acaacgttca aatccgctcc cggcggattt gtcctactca ggagagcgtt caccgacaaa
```

```
 421 caacagataa aacgaaaggc ccagtcttcc gactgagcct ttcgttttat ttgatgcctg
 481 gcagttccct actctcgcgt taacgctagc atggatgttt tcccagtcac gacgttgtaa
 541 aacgacggcc agtcttaagc tcgggcccct acaggtcact aataccatct aagtagttga
 601 ttcatagtga ctggatatgt tgtgttttac agtattatgt agtctgtttt ttatgcaaaa
 661 tctaatttaa tatattgata tttatatcat tttacgtttc tcgttcaact tttctataca
 721 aagttggtac cgggcccccc gctagcgtcg acggtatcga tgtagaacgc gagcaaattc
 781 ttttccttcc atgacagcag cagctacagt gggaagccga acgtcagacg tgtttgacat
 841 gccgaactgg gcgggaaaat tacagcgtgc gctttgtttt caagcaaatc acaactcgct
 901 gcaaacaaaa ccgttgagaa attgattgtt ttataatttg tattgtattt tatttgttat
 961 aataaactaa aaagacatac tttttgcata ttttatacat aaaaacatac atgcagcatt
1021 ataaaacaca tataaaccct ccctgtagag tcccgtatcg aaatcttcca tcctagttgc
1081 acagtacgac ggacgagtag gccgtgtccg tgcaaattcc agcttttagc agtcttttgc
1141 tcggagcact cgcggcgagt cggaggtttc tgctgaggtg cttagcgcta aattagccaa
1201 ttgcttttgc aagtgaaata accagccgaa tagtacttca aaactcaggt aagtgaacta
1261 gttttataga acaaatgttt gtttgttaga agttagtgaa gtgtttgtga aaaaaatctc
1321 tcatttcggc aaaactaacg taactgattt caaattgaat tattgttttg tgatgttata
1381 ttatttcatc cagttgatta gtattttctt agttatgttc aaaatacagt taaattaaat
1441 ttcatttcat ttactcataa aataatctct tggcttattt aatttttctc gaattcgctt
1501 gtattgttca gtagcacgcg ccattcgccc tttgtttcat tttgtacctg ctcccactaa
1561 cacactggca gtgcgaaaca aaagccttcg cacgcgttgc tggtattaga gtgtgtgcgt
1621 gtgtgtgttg agcgctctgt caaaatcggc tgttgccgcc ggtaccgaaa ttgcctgttc
1681 gcacgctgtt cgtaaacatt ccgtggtgtg tatcgtgtgt tgtgcatgtt gcgcgcctcc
1741 ccccttttga tagcaggctg ccgtggctgc cgtggtgtgt ggcgcagttg agtttttgga
1801 ttaattttct aaggaaatgg cacgagaaga gcggtggcag tgtgttggtt tgctctgtcc
1861 cttcctttct gtgtgaagtg ttcttacagc acagcacgta tccaccaccg cacacagagc
1921 aggcaaggaa gtggaagtga acaagtgtgc tgcgcatgca tgtgtgtggg gggcatttta
1981 gctgagatcg tcgttatttg agaagcggta taggggccag tcggtgtcga cgtacggaag
2041 cggtttagtt ttaatccaag cgtatcccgt cgtggagtgg ttgtgtggct ctgtgtgctc
2101 tcatatcagt tccagagtga ggttagtaga atcacagtcc ttggcctttt tcgttacaag
2161 atatccagaa ggatggcgtt atttccacag cttaccatgg tgctcttgtt tgctcgaatc
2221 aggggagaaa aacagtttcg tgtttcatga accgcagttg gcactggagc ggattcaaaa
2281 gtcttcgata tgcaatagat aagagagtcg ttggggcata gttgggaagc ctttccgaga
2341 tgtggagttt ccgagaggag aaatggtgct ttcgtgcacg ttccgggaca gcgggccccg
2401 cgaagagcat ctcgttgtcg ttcatccggc aataattgat gcgaaaagcg cgcgcgccac
2461 tggcttagcg cagtgtacac agtgatattc acctacacac acagaggcac acgccttcac
2521 acgcgcgcgt gcttcaaagg ctacttcggt ggcggtgtgt gaggtcgctt gcaatggaca
2581 atgaaaattt cgctggaaaa taccatcgtc tctttaggtt gcaatgggtg cgggtagagc
2641 ggtggtcgtc gatattggtg gtgtagtgtg tgtgtgtgtg tgtgtgtgtg tgtgtgtgtg
2701 tgtgtgtgtg tgtgtgtgtg tgtgtgtgtg tgtgtgtgtg tgtgtgtgtg tgtgtgtgtg
2761 tgcaacggca attatttttt gtaatatttc gaccatcttt ctttctctct ctccacgtgc
2821 tgctgctgtt gctgctgctg ctgcattgca tgttccacta ttcctctcgg tttgtgcctg
2881 cggacgccat tgctagtcga aagagagtcg ccgttagtcg cgcttcgagc aacggacacg
2941 tttttggtt gaaaccaaca gctttttca tcttcgggag acacacagat ctcgaatcgt
3001 acattcccat aaggagaatt gtcatcttcc ggtgaataaa gaaaggaaac ggatccggaa
3061 acatgggatc atcatcagac gacgaagcta cggccgatgc gcagcatgca gcaccaccaa
3121 aaaaaaaacg aaaagtagaa gacccacgat ttatgtaccc atacgatgtt cctgactatg
3181 cgggtatgaa aaacatcaaa aaaaaccagg tTATGGACAC GTACGCGGGT GCTTACGACC
3241 GTCAGTCGCG CGAGCGCGAA AATTCGAGCG CAGCAAGCCC AGCGACACAG CGTAGCGCCA
3301 ACGAAGACAA GGCGGCCGAC CTTCAGCGCG AAGTCGAGCG CGACGGGGGC CGGTTCAGGT
3361 TCGTCGGGCA TTTCAGCGAA GCGCCGGGCA CGTCGGCGTT CGGGACGGCG GAGCGCCCGG
3421 AGTTCGAACG CATCCTGAAC GAATGCCGCG CCGGGCGGCT CAACATGATC ATTGTCTATG
3481 ACGTGTCGCG CTTCTCGCGC CTGAAGGTCA TGGACGCGAT TCCGATTGTC TCGGAATTGC
3541 TCGCCCTGGG CGTGACGATT GTTTCCACTC AGGAAGGCGT CTTCCGGCAG GGAAACGTCA
3601 TGGACCTGAT TCACCTGATT ATGCGGCTCG ACGCGTCGCA CAAAGAATCT TCGCTGAAGT
3661 CGGCGAAGAT TCTCGACACG AAGAACCTTC AGCGCGAATT GGGCGGGTAC GTCGGCGGGA
3721 AGGCGCCTTA CGGCTTCGAG CTTGTTTCGG AGACGAAGGA GATCACGCGC AACGGCCGAA
3781 TGGTCAATGT CGTCATCAAC AAGCTTGCGC ACTCGACCAC TCCCCTTACC GGACCCTTCG
3841 AGTTCGAGCC CGACGTAATC CGGTGGTGGT GGCGTGAGAT CAAGACGCAC AAACACCTTC
3901 CCTTCAAGCC GGGCAGTCAA GCCGCCATTC ACCCGGGCAG CATCACGGGG CTTTGTAAGC
3961 GCATGGACGC TGACGCCGTG CCGACCCGGG GCGAGACGAT TGGGAAGAAG ACCGCTTCAA
4021 GCGCCTGGGA CCCGGCAACC GTTATGCGAA TCCTTCGGGA CCCGCGTATT GCGGGCTTCG
4081 CCGCTGAGGT GATCTACAAG AAGAAGCCGG ACGGCACGCC GACCACGAAG ATTGAGGGTT
4141 ACCGCATTCA GCGCGACCCG ATCACGCTCC GGCCGGTCGA GCTTGATTGC GGACCGATCA
4201 TCGAGCCCGC TGAGTGGTAT GAGCTTCAGG CGTGGTTGGA CGGCAGGGGG CGCGGCAAGG
4261 GGCTTTCCCG GGGGCAAGCC ATTCTGTCCG CCATGGACAA GCTGTACTGC GAGTGTGGCG
```

```
4321 CCGTCATGAC TTCGAAGCGC GGGGAAGAAT CGATCAAGGA CTCTTACCGC TGCCGTCGCC
4381 GGAAGGTGGT CGACCCGTCC GCACCTGGGC AGCACGAAGG CACGTGCAAC GTCAGCATGG
4441 CGGCACTCGA CAAGTTCGTT GCGGAACGCA TCTTCAACAA GATCAGGCAC GCCGAAGGCG
4501 ACGAAGAGAC GTTGGCGCTT CTGTGGGAAG CCGCCCGACG CTTCGGCAAG CTCACTGAGG
4561 CGCCTGAGAA GAGCGGCGAA CGGGCGAACC TTGTTGCGGA GCGCGCCGAC GCCCTGAACG
4621 CCCTTGAAGA GCTGTACGAA GACCGCGCGG CAGGCGCGTA CGACGGACCC GTTGGCAGGA
4681 AGCACTTCCG GAAGCAACAG GCAGCGCTGA CGCTCCGGCA GCAAGGGGCG GAAGAGCGGC
4741 TTGCCGAACT TGAAGCCGCC GAAGCCCCGA AGCTTCCCCT TGACCAATGG TTCCCCGAAG
4801 ACGCCGACGC TGACCCGACC GGCCCTAAGT CGTGGTGGGG GCGCGCGTCA GTAGACGACA
4861 AGCGCGTGTT CGTCGGGCTC TTCGTAGACA AGATCGTTGT CACGAAGTCG ACTACGGGCA
4921 GGGGGCAGGG AACGCCCATC GAGAAGCGCG CTTCGATCAC GTGGGCGAAG CCGCCGACCG
4981 ACGACGACGA AGACGACGCC CAGGACGGCA CGGAAGACGT AGCGGCGTAG tgtacCCCcc
5041 tcgagcttgg ggtggggttg ttatgtgttg cgaacgagag tggatctctc tcgacatttc
5101 cttatttttt ttttctattg ttaacactta caacgaaact tcggaagaga agtttcctca
5161 ttcgaaacga ggagtcaaac tcctttcctt gcttttgtga catgcatgat tattctttca
5221 tttactgacg taacgatgta aaacacacag aagaagcatg acacacagca aagaattgtt
5281 cgttaataaa acgttgatga aaactttgaa aacataagaa cttgcacttt tattctataa
5341 ttcgtgaaag cttgcaccga ttgtctttca ttattcaatg taatgtactg aaaggtgatt
5401 tttcgcactt gtatactcta gaactgaagt attctaacaa tacgtcacct ttaggtccat
5461 tccaggataa aatacacaag tgaaggtagt tgtacaaagt acttagacta acccaggttt
5521 caaaaaagat aacgacgaat tgagcgtaac tgcacaaagc ccgttcattg tcaacatatc
5581 gttcaggggt cttcaaactt tattatcagc ccgcgggccg caacaacgtt gcttagagca
5641 ccatcttgat gtatccttta tctagtggct tatcgtttat gtctcgtttg gctaagaaaa
5701 tactaataac atattcaatt tacggagcgg taccatggta caaacgtcat actgctagac
5761 ttgacaacat gcccatcgag ggttcttacc tcaaatggac cgatcccttg taactggcgg
5821 cgtaaactca gtaaattcca aaagccagta taggccacca tgaccgtcta actattccaa
5881 aaaggagaat atttgaaatt tctatatcta tagtattatt actatatcta tatgtttctt
5941 tagtacaata ttagagcttg attattttcc tgaaaattgt aaaatatgca tgttagatgg
6001 ttgatacatc gttttaaaaa aaactgctgt taatggccac attttctcga gacaactttg
6061 tataataaag ttgaacgaga aacgtaaaat gatataaata tcaatatatt aaattagatt
6121 ttgcataaaa aacagactac ataatactgt aaaacacaac atatccagtc actatgaatc
6181 aactacttag atggtattag tgacctgtac tgcagggcgg ccgcgatatc ccctatagtg
6241 agtcgtatta catggtcata gctgtttcct ggcagctctg gcccgtgtct caaaatctct
6301 gatgttacat tgcacaagat aaaaatatat catcatgaac aataaaactg tctgcttaca
6361 taaacagtaa tacaaggggt gttatgagcc atattcaacg ggaaacgtcg aggccgcgat
6421 taaattccaa catggatgct gatttatatg ggtataaatg ggctcgcgat aatgtcgggc
6481 aatcaggtgc gacaatctat cgcttgtatg ggaagcccga tgcgccagag ttgtttctga
6541 aacatggcaa aggtagcgtt gccaatgatg ttacagatga gatggtcaga ctaaactggc
6601 tgacggaatt tatgcctctt ccgaccatca agcattttat ccgtactcct gatgatgcat
6661 ggttactcac cactgcgatc cccggaaaaa cagcattcca ggtattagaa gaatatcctg
6721 attcaggtga aaatattgtt gatgcgctgg cagtgttcct gcgccggttg cattcgattc
6781 ctgtttgtaa ttgtcctttt aacagcgatc gcgtatttcg tctcgctcag gcgcaatcac
6841 gaatgaataa cggtttggtt gatgcgagtg attttgatga cgagcgtaat ggctggcctg
6901 ttgaacaagt ctggaaagaa atgcataaac ttttgccatt ctcaccggat tcagtcgtca
6961 ctcatggtga tttctcactt gataacctta tttttgacga ggggaaatta ataggttgta
7021 ttgatgttgg acgagtcgga atcgcagacc gataccagga tcttgccatc ctatggaact
7081 gcctcggtga gttttctcct tcattacaga aacggctttt tcaaaaatat ggtattgata
7141 atcctgatat gaataaattg cagtttcatt tgatgctcga tgagtttttc taatcagaat
7201 tggttaattg gttgtaacac tggcagagca ttacgctgac ttgacgggac ggcgcaagct
7261 catgaccaaa atcccttaac gtgagttacg cgtcgttcca ctgagcgtca gaccccgtag
7321 aaaagatcaa aggatcttct tgagatcctt tttttctgcg cgtaatctgc tgcttgcaaa
7381 caaaaaaacc accgctacca gcggtggttt gtttgccgga tcaagagcta ccaactcttt
7441 ttccgaaggt aactggcttc agcagagcgc agataccaaa tactgttctt ctagtgtagc
7501 cgtagttagg ccaccacttc aagaactctg tagcaccgcc tacatacctc gctctgctaa
7561 tcctgttacc agtggctgct gccagtggcg ataagtcgtg tcttaccggg ttggactcaa
7621 gacgatagtt accggataag gcgcagcggt cgggctgaac ggggggttcg tgcacacagc
7681 ccagcttgga gcgaacgacc tacaccgaac tgagatacct acagcgtgag ctatgagaaa
7741 gcgccacgct tcccgaaggg agaaaggcgg acaggtatcc ggtaagcggc agggtcggaa
7801 caggagagcg cacgagggag cttccagggg gaaacgcctg gtatctttat agtcctgtcg
7861 ggtttcgcca cctctgactt gagcgtcgat ttttgtgatg ctcgtcaggg gggcggagcc
7921 tatggaaaaa cgccagcaac gcggcctttt tacggttcct ggccttttgc tggccttttg
7981 ctcacatgtt
//
```

pENTR R4-vas2-Transposase-R3 (helper plasmid expressing *piggyBac* transposase under the control of the vasa promoter). Kanamycin resistant

```
LOCUS       pENTRR4-Vas2-Transp-R3        6847 bp    DNA     circular
FEATURES             Location/Qualifiers
     misc_feature    complement(603..727)
                     /dnas_title="attR4"
                     /vntifkey="21"
                     /label=attR4
                     /note="attR4"
     misc_feature    5945..6069
                     /dnas_title="attR3"
                     /vntifkey="21"
                     /label=attR3
                     /note="attR3"
     misc_feature    complement(6188..6206)
                     /dnas_title="pDONR-RP"
                     /vntifkey="21"
                     /label=pDONR-RP
                     /note="pDONR-RP"
     misc_feature    537..552
                     /dnas_title="M13F"
                     /vntifkey="21"
                     /label=M13F
                     /note="M13F"
     misc_feature    760..3050
                     /dnas_title="Vasa promoter"
                     /vntifkey="21"
                     /label=Vasa promoter
     misc_feature    4939..5939
                     /dnas_title="3'UTR_Vasa"
                     /vntifkey="21"
                     /label=3'UTR_Vasa
     misc_feature    3068..4852
                     /note="transposase ORF"
     misc_feature    complement(6144..6162)
                     /note="M13R"
     misc_feature    complement(6124..6143)
                     /note="T7"

ORIGIN
        1 ctttcctgcg ttatcccctg attctgtgga taaccgtatt accgcctttg agtgagctga
       61 taccgctcgc cgcagccgaa cgaccgagcg cagcgagtca gtgagcgagg aagcggaaga
      121 gcgcccaata cgcaaaccgc ctctccccgc gcgttggccg attcattaat gcagctggca
      181 cgacaggttt cccgactgga aagcgggcag tgagcgcaac gcaattaata cgcgtaccgc
      241 tagccaggaa gagtttgtag aaacgcaaaa aggccatccg tcaggatggc cttctgctta
      301 gtttgatgcc tggcagttta tggcgggcgt cctgcccgcc accctccggg ccgttgcttc
      361 acaacgttca aatccgctcc cggcggattt gtcctactca ggagagcgtt caccgacaaa
      421 caacagataa aacgaaaggc ccagtcttcc gactgagcct ttcgttttat ttgatgcctg
      481 gcagttccct actctcgcgt taacgctagc atggatgttt tcccagtcac gacgttgtaa
      541 aacgacggcc agtcttaagc tcgggcccct acaggtcact aataccatct aagtagttga
      601 ttcatagtga ctggatatgt tgtgttttac agtattatgt agtctgtttt ttatgcaaaa
      661 tctaatttaa tatattgata tttatatcat tttacgtttc tcgttcaact tttctataca
      721 aagttggtac cgggcccccc gctagcgtcg acggtatcga tgtagaacgc gagcaaattc
      781 ttttccttcc atgacagcag cagctacagt gggaagccga acgtcagacg tgtttgacat
      841 gccgaactgg gcgggaaaat tacagcgtgc gctttgtttt caagcaaatc acaactcgct
      901 gcaaacaaaa ccgttgagaa attgattgtt ttataatttg tattgtattt tatttgttat
      961 aataaactaa aaagacatac tttttgcata ttttatacat aaaaacatac atgcagcatt
     1021 ataaaacaca tataaaccct ccctgtagag tcccgtatcg aaatcttcca tcctagttgc
     1081 acagtacgac ggacgagtag gccgtgtccg tgcaaattcc agcttttagc agtcttttgc
     1141 tcggagcact cgcggcgagt cggaggtttc tgctgaggtg cttagcgcta aattagccaa
     1201 ttgcttttgc aagtgaaata accagccgaa tagtacttca aaactcaggt aagtgaacta
     1261 gttttataga acaaatgttt gtttgttaga agttagtgaa gtgtttgtga aaaaaatctc
     1321 tcatttcggc aaaactaacg taactgattt caaattgaat tattgttttg tgatgttata
```

1381 ttatttcatc cagttgatta gtattttctt agttatgttc aaaatacagt taaattaaat
1441 ttcatttcat ttactcataa aataatctct tggcttattt aatttttctc gaattcgctt
1501 gtattgttca gtagcacgcg ccattcgccc tttgtttcat tttgtacctg ctcccactaa
1561 cacactggca gtgcgaaaca aaagccttcg cacgcgttgc tggtattaga gtgtgtgcgt
1621 gtgtgtgttg agcgctctgt caaaatcggc tgttgccgcc ggtaccgaaa ttgcctgttc
1681 gcacgctgtt cgtaaacatt ccgtggtgtg tatcgtgtgt tgtgcatgtt gcgcgcctcc
1741 cccctttga tagcaggctg ccgtggctgc cgtggtgtgt ggcgcagttg agtttttgga
1801 ttaatttct aaggaaatgg cacgagaaga gcggtggcag tgtgttggtt tgctctgtcc
1861 cttcctttct gtgtgaagtg ttcttacagc acagcacgta tccaccaccg cacacagagc
1921 aggcaaggaa gtggaagtga acaagtgtgc tgcgcatgca tgtgtgtggg gggcatttta
1981 gctgagatcg tcgttatttg agaagcggta taggggccag tcggtgtcga cgtacggaag
2041 cggtttagtt ttaatccaag cgtatcccgt cgtggagtgg ttgtgtggct ctgtgtgctc
2101 tcatatcagt tccagagtga ggttagtaga atcacagtcc ttggcctttt tcgttacaag
2161 atatccagaa ggatggcgtt atttccacag cttaccatgg tgctcttgtt tgctcgaatc
2221 aggggagaaa aacagtttcg tgtttcatga accgcagttg gcactggagc ggattcaaaa
2281 gtcttcgata tgcaatagat aagagagtcg ttggggcata gttgggaagc ctttccgaga
2341 tgtggagttt ccgagaggag aaatggtgct ttcgtgcacg ttccgggaca gcgggccccg
2401 cgaagagcat ctcgttgtcg ttcatccggc aataattgat gcgaaaagcg cgcgcgccac
2461 tggcttagcg cagtgtacac agtgatattc acctacacac acagaggcac acgccttcac
2521 acgcgcgcgt gcttcaaagg ctacttcggt ggcggtgtgt gaggtcgctt gcaatggaca
2581 atgaaaattt cgctggaaaa taccatcgtc tctttaggtt gcaatgggtg cgggtagagc
2641 ggtggtcgtc gatattggtg gtgtagtgtg tgtgtgtgtg tgtgtgtgtg tgtgtgtgtg
2701 tgtgtgtgtg tgtgtgtgtg tgtgtgtgtg tgtgtgtgtg tgtgtgtgtg tgtgtgtgtg
2761 tgcaacggca attatttttt gtaatatttc gaccatcttt ctttctctct ctccacgtgc
2821 tgctgctgtt gctgctgctg ctgcattgca tgttccacta ttcctctcgg tttgtgcctg
2881 cggacgccat tgctagtcga aagagagtcg ccgttagtcg cgcttcgagc aacggacacg
2941 tttttggtt gaaaccaaca gctttttca tcttcgggag acacacagat ctcgaatcgt
3001 acattcccat aaggagaatt gtcatcttcc ggtgaataaa gaaaggaaac gGATCCTATA
3061 TAATAAAATG GGTAGTTCTT TAGACGATGA GCATATCCTC TCTGCTCTTC TGCAAAGCGA
3121 TGACGAGCTT GTTGGTGAGG ATTCTGACAG TGAAATATCA GATCACGTAA GTGAAGATGA
3181 CGTCCAGAGC GATACAGAAG AAGCGTTTAT AGATGAGGTA CATGAAGTGC AGCCAACGTC
3241 AAGCGGTAGT GAAATATTAG ACGAACAAAA TGTTATTGAA CAACCAGGTT CTTCATTGGC
3301 TTCTAACAGA ATCTTGACCT TGCCACAGAG GACTATTAGA GGTAAGAATA AACATTGTTG
3361 GTCAACTTCA AAGTCCACGA GGCGTAGCCG AGTCTCTGCA CTGAACATTG TCAGATCTCA
3421 AAGAGGTCCG ACGCGTATGT GCCGCAATAT ATATGACCCA CTTTTATGCT TCAAACTATT
3481 TTTTACTGAT GAGATAATTT CGGAAATTGT AAAATGGACA AATGCTGAGA TATCATTGAA
3541 ACGTCGGGAA TCTATGACAG GTGCTACATT TCGTGACACG AATGAAGATG AAATCTATGC
3601 TTTCTTTGGT ATTCTGGTAA TGACAGCAGT GAGAAAAGAT AACCACATGT CCACAGATGA
3661 CCTCTTTGAT CGATCTTTGT CAATGGTGTA CGTCTCTGTA ATGAGTCGTG ATCGTTTTGA
3721 TTTTTTGATA CGATGTCTTA GAATGGATGA CAAAAGTATA CGGCCCACAC TTCGAGAAAA
3781 CGATGTATTT ACTCCTGTTA GAAAAATATG GGATCTCTTT ATCCATCAGT GCATACAAAA
3841 TTACACTCCA GGGGCTCATT TGACCATAGA TGAACAGTTA CTTGGTTTTA GAGGACGGTG
3901 TCCGTTTAGG ATGTATATCC CAAACAAGCC AAGTAAGTAT GGAATAAAAA TCCTCATGAT
3961 GTGTGACAGT GGTACGAAGT ATATGATAAA TGGAATGCCT TATTTGGGAA GAGGAACACA
4021 GACCAACGGA GTACCACTCG GTGAATACTA CGTGAAGGAG TTATCAAAGC CTGTGCACGG
4081 TAGTTGTCGT AATATTACGT GTGACAATTG GTTCACCTCA ATCCCTTTaG CAAAAAACTT
4141 ACTACAAGAA CCGTATAAGT TAACCATTGT GGGAACCGTG CGATCAAACA AACGCGAGAT
4201 ACCGGAAGTA CTGAAAAACA GTCGCTCCAG GCCAGTGGGA ACATCGATGT TTTGTTTTGA
4261 CGGACCCCTT ACTCTCGTCT CATATAAACC GAAGCCAGCT AAGATGGTAT ACTTATTATC
4321 ATCTTGTGAT GAGGATGCTT CTATCAACGA AAGTACCGGT AAACCGCAAA TGGTTATGTA
4381 TTATAATCAA ACTAAAGGCG GAGTGGACAC GCTAGACCAA ATGTGTTCTG TGATGACCTG
4441 CAGTAGGAAG ACGAATAGGT GGCCTATGGC ATTATTGTAC GGAATGATAA ACATTGCCTG
4501 CATAAATTCT TTTATTATAT ACAGCCATAA TGTCAGTAGC AAGGGAGAAA AGGTTCAAAG
4561 TCGCAAAAAA TTTATGAGAA ACCTTTACAT GAGCCTGACG TCATCGTTTA TGCGTAAGCG
4621 TTTAGAAGCT CCTACTTTGA AGAGATATTT GCGCGATAAT ATCTCTAATA TTTTGCCAAA
4681 TGAAGTGCCT GGTACATCAG ATGACAGTAC TGAAGAGCCA GTAATGAAAA AACGTACTTA
4741 CTGTACTTAC TGCCCCTCTA AAATAAGGCG AAAGGCAAAT GCATCGTGCA AAAAATGCAA
4801 AAAAGTTATT TGTCGAGAGC ATAATATTGA TATGTGCCAA AGTTGTTTCT GACTGACTAA
4861 TAAGTATAAT TTGTTTCTAT TATGTATAAG TTAAgCTAAT TACTTATTTT ATAATACAAC
4921 ATGACTGTTT TTctcgagct tggggtgggg ttgttatgtg ttgcgaacga gagtggatct
4981 ctctcgacat ttccttattt ttttttcta ttgttaacac ttacaacgaa acttcggaag
5041 agaagtttcc tcattcgaaa cgaggagtca aactcctttc cttgcttttg tgacatgcat
5101 gattattctt tcatttactg acgtaacgat gtaaaacaca cagaagaagc atgacacaca
5161 gcaaagaatt gttcgttaat aaaacgttga tgaaaacttt gaaaacataa gaacttgcac
5221 ttttattcta taattcgtga aagcttgcac cgattgtctt tcattattca atgtaatgta

```
     5281 ctgaaaggtg atttttcgca cttgtatact ctagaactga agtattctaa caatacgtca
     5341 cctttaggtc cattccagga taaaatacac aagtgaaggt agttgtacaa agtacttaga
     5401 ctaacccagg tttcaaaaaa gataacgacg aattgagcgt aactgcacaa agcccgttca
     5461 ttgtcaacat atcgttcagg ggtcttcaaa ctttattatc agcccgcggg ccgcaacaac
     5521 gttgcttaga gcaccatctt gatgtatcct ttatctagtg gcttatcgtt tatgtctcgt
     5581 ttggctaaga aaatactaat aacatattca atttacggag cggtaccatg gtacaaacgt
     5641 catactgcta gacttgacaa catgcccatc gagggttctt acctcaaatg gaccgatccc
     5701 ttgtaactgg cggcgtaaac tcagtaaatt ccaaaagcca gtataggcca ccatgaccgt
     5761 ctaactattc caaaaaggag aatatttgaa atttctatat ctatagtatt attactatat
     5821 ctatatgttt ctttagtaca atattagagc ttgattattt tcctgaaaat tgtaaaatat
     5881 gcatgttaga tggttgatac atcgttttaa aaaaaactgc tgttaatggc cacattttct
     5941 cgagacaact ttgtataata aagttgaacg agaaacgtaa aatgatataa atatcaatat
     6001 attaaattag attttgcata aaaaacagac tacataatac tgtaaaacac aacatatcca
     6061 gtcactatga atcaactact tagatggtat tagtgacctg tactgcaggg cggccgcgat
     6121 atcccctata gtgagtcgta ttacatggtc atagctgttt cctggcagct ctggcccgtg
     6181 tctcaaaatc tctgatgtta cattgcacaa gataaaaata tatcatcatg aacaataaaa
     6241 ctgtctgctt acataaacag taatacaagg ggtgttatga gccatattca acgggaaacg
     6301 tcgaggccgc gattaaattc caacatggat gctgatttat atgggtataa atgggctcgc
     6361 gataatgtcg ggcaatcagg tgcgacaatc tatcgcttgt atgggaagcc cgatgcgcca
     6421 gagttgtttc tgaaacatgg caaaggtagc gttgccaatg atgttacaga tgagatggtc
     6481 agactaaact ggctgacgga atttatgcct cttccgacca tcaagcattt tatccgtact
     6541 cctgatgatg catggttact caccactgcg atccccggaa aaacagcatt ccaggtatta
     6601 gaagaatatc ctgattcagg tgaaaatatt gttgatgcgc tggcagtgtt cctgcgccgg
     6661 ttgcattcga ttcctgtttg taattgtcct tttaacagcg atcgcgtatt tcgtctcgct
     6721 caggcgcaat cacgaatgaa taacggtttg gttgatgcga gtgattttga tgacgagcgt
     6781 aatggctggc ctgttgaaca agtctggaaa gaaatgcata aacttttgcc attctcaccg
     6841 gattcagtcg tcactcatgg tgatttctca cttgataacc ttatttttga cgaggggaaa
     6901 ttaataggtt gtattgatgt tggacgagtc ggaatcgcag accgatacca ggatcttgcc
     6961 atcctatgga actgcctcgg tgagttttct ccttcattac agaaacggct ttttcaaaaa
     7021 tatggtattg ataatcctga tatgaataaa ttgcagtttc atttgatgct cgatgagttt
     7081 ttctaatcag aattggttaa ttggttgtaa cactggcaga gcattacgct gacttgacgg
     7141 gacggcgcaa gctcatgacc aaaatccctt aacgtgagtt acgcgtcgtt ccactgagcg
     7201 tcagaccccg tagaaaagat caaaggatct tcttgagatc ctttttttct gcgcgtaatc
     7261 tgctgcttgc aaacaaaaaa accaccgcta ccagcggtgg tttgtttgcc ggatcaagag
     7321 ctaccaactc tttttccgaa ggtaactggc ttcagcagag cgcagatacc aaatactgtt
     7381 cttctagtgt agccgtagtt aggccaccac ttcaagaact ctgtagcacc gcctacatac
     7441 ctcgctctgc taatcctgtt accagtggct gctgccagtg gcgataagtc gtgtcttacc
     7501 gggttggact caagacgata gttaccggat aaggcgcagc ggtcgggctg aacggggggt
     7561 tcgtgcacac agcccagctt ggagcgaacg acctacaccg aactgagata cctacagcgt
     7621 gagctatgag aaagcgccac gcttcccgaa gggagaaagg cggacaggta tccggtaagc
     7681 ggcagggtcg gaacaggaga gcgcacgagg gagcttccag ggggaaacgc ctggtatctt
     7741 tatagtcctg tcgggtttcg ccacctctga cttgagcgtc gatttttgtg atgctcgtca
     7801 ggggggcgga gcctatggaa aaacgccagc aacgcggcct ttttacggtt cctggccttt
     7861 tgctggcctt ttgctcacat gtt
//
```

pENTR L1-vas2-Cre-L4 (helper plasmid expressing *Cre recombinase* under the control of the vasa promoter). Kanamycin resistant

```
LOCUS       pENTR L1-vasaCre-L4                  7138 bp    DNA     circular
FEATURES             Location/Qualifiers
     misc_feature    complement(5443..5461)
                     /note="DONR-RP"
     misc_feature    570..665
                     /note="attL1"
     misc_feature    complement(5253..5348)
                     /note="attL4"
     misc_feature    537..553
                     /note="M13F"
     misc_feature    complement(5399..5417)
```

```
                     /note="M13R"
     misc_feature    703..2991
                     /dnas_title="Vasa promoter"
                     /vntifkey="21"
                     /label=vasa promoter
     misc_feature    4263..5241
                     /dnas_title="3'UTR_Vasa"
                     /vntifkey="21"
                     /label=3'UTR_Vasa
     misc_feature    complement(3061..3074)
                     /note="NLS"
     misc_feature    3098..3123
                     /note="HA epitope"
     misc_feature    3166..4248
                     /note="Cre ORF"

ORIGIN
        1 CTTTCCTGCG TTATCCCCTG ATTCTGTGGA TAACCGTATT ACCGCCTTTG AGTGAGCTGA
       61 TACCGCTCGC CGCAGCCGAA CGACCGAGCG CAGCGAGTCA GTGAGCGAGG AAGCGGAAGA
      121 GCGCCCAATA CGCAAACCGC CTCTCCCCGC GCGTTGGCCG ATTCATTAAT GCAGCTGGCA
      181 CGACAGGTTT CCCGACTGGA AAGCGGGCAG TGAGCGCAAC GCAATTAATA CGCGTACCGC
      241 TAGCCAGGAA GAGTTTGTAG AAACGCAAAA AGGCCATCCG TCAGGATGGC CTTCTGCTTA
      301 GTTTGATGCC TGGCAGTTTA TGGCGGGCGT CCTGCCCGCC ACCCTCCGGG CCGTTGCTTC
      361 ACAACGTTCA AATCCGCTCC CGGCGGATTT GTCCTACTCA GGAGAGCGTT CACCGACAAA
      421 CAACAGATAA AACGAAAGGC CCAGTCTTCC GACTGAGCCT TTCGTTTTAT TTGATGCCTG
      481 GCAGTTCCCT ACTCTCGCGT TAACGCTAGC ATGGATGTTT TCCCAGTCAC GACGTTGTAA
      541 AACGACGGCC AGTCTTAAGC TCGGGCCCCA AATAATGATT TTATTTTGAC TGATAGTGAC
      601 CTGTTCGTTG CAACAAATTG ATGAGCAATG CTTTTTTATA ATGCCAACTT TGTACAAAAA
      661 AGCAGGCTGG TACCGGGCCC CCCGCTAGCG TCGACGGTAT cgatgtagaa cgcgagcaaa
      721 ttcttttcct tccatgacag cagcagctac agtgggaagc cgaacgtcag acgtgtttga
      781 catgccgaac tgggcgggaa aattacagcg tgcgctttgt tttcaagcaa atcacaactc
      841 gctgcaaaca aaaccgttga gaaattgatt gttttataat ttgtattgta ttttatttgt
      901 tataataaac taaaaagaca tactttttgc atattttata cataaaaaca tacatgcagc
      961 attataaaac acatataaac cctccctgta gagtcccgta tcgaaatctt ccatcctagt
     1021 tgcacagtac gacggacgag taggccgtgt ccgtgcaaat tccagctttt agcagtcttt
     1081 tgctcggagc actcgcggcg agtcggaggt ttctgctgag gtgcttagcg ctaaattagc
     1141 caattgcttt tgcaagtgaa ataaccagcc gaatagtact tcaaaactca ggtaagtgaa
     1201 ctagttttat agaacaaatg tttgtttgtt agaagttagt gaagtgtttg tgaaaaaaat
     1261 ctctcatttc ggcaaaacta acgtaactga tttcaaattg aattattgtt ttgtgatgtt
     1321 atattatttc atccagttga ttagtatttt cttagttatg ttcaaaatac agttaaatta
     1381 aatttcattt catttactca taaaataatc tcttggctta tttaattttt ctcgaattcg
     1441 cttgtattgt tcagtagcac gcgccattcg ccctttgttt cattttgtac ctgctcccac
     1501 taacacactg gcagtgcgaa acaaaagcct tcgcacgcgt tgctggtatt agagtgtgtg
     1561 cgtgtgtgtg ttgagcgctc tgtcaaaatc ggctgttgcc gccggtaccg aaattgcctg
     1621 ttcgcacgct gttcgtaaac attccgtggt gtgtatcgtg tgttgtgcat gttgcgcgcc
     1681 tcccccсttt tgatagcagg ctgccgtggc tgccgtggtg tgtggcgcag ttgagttttt
     1741 ggattaattt tctaaggaaa tggcacgaga agagcggtgg cagtgtgttg gtttgctctg
     1801 tcccttcctt tctgtgtgaa gtgttcttac agcacagcac gtatccacca ccgcacacag
     1861 agcaggcaag gaagtggaag tgaacaagtg tgctgcgcat gcatgtgtgt gggggggcatt
     1921 ttagctgaga tcgtcgttat ttgagaagcg gtataggggc cagtcggtgt cgacgtacgg
     1981 aagcggttta gttttaatcc aagcgtatcc cgtcgtggag tggttgtgtg gctctgtgtg
     2041 ctctcatatc agttccagag tgaggttagt agaatcacag tccttggcct ttttcgttac
     2101 aagatatcca gaaggatggc gttatttcca cagcttacca tggtgctctt gtttgctcga
     2161 atcaggggag aaaaacagtt tcgtgtttca tgaaccgcag ttggcactgg agcggattca
     2221 aaagtcttcg atatgcaata gataagagag tcgttggggc atagttggga agcctttccg
     2281 agatgtggag tttccgagag gagaaatggt gctttcgtgc acgttccggg acagcgggcc
     2341 ccgcgaagag catctcgttg tcgttcatcc ggcaataatt gatgcgaaaa gcgcgcgcgc
     2401 cactggctta gcgcagtgta cacagtgata ttcacctaca cacacagagg cacacgcctt
     2461 cacacgcgcg cgtgcttcaa aggctacttc ggtggcggtg tgtgaggtcg cttgcaatgg
     2521 acaatgaaaa tttcgctgga aaataccatc gtctctttag gttgcaatgg gtgcgggtag
     2581 agcggtggtc gtcgatattg gtggtgtagt gtgtgtgtgt gtgtgtgtgt gtgtgtgtgt
     2641 gtgtgtgtgt gtgtgtgtgt gtgtgtgtgt gtgtgtgtgt gtgtgtgtgt gtgtgtgtgt
     2701 gtgcaacggc aattattttt tgtaatattt cgaccatctt tctttctctc tctccacgtg
     2761 ctgctgctgt tgctgctgct gctgcattgc atgttccact attcctctcg gtttgtgcct
     2821 gcggacgcca ttgctagtcg aaagagagtc gccgttagtc gcgcttcgag caacggacac
```

```
2881 gtttttttggt tgaaaccaac agcttttttc atcttcggga gacacacaga tctcgaatcg
2941 tacattccca taaggagaat tgtcatcttc cggtgaataa agaaaggaaa cggatccgga
3001 aacatgggat catcatcaga cgacgaagct acggccgatg cgcagcatgc agcaccacca
3061 aaaaaaaaac gaaaagtaga agacccacga tttatgtacc catacgatgt tcctgactat
3121 gcgggtatga aaaacatcaa aaaaaaccag gGAATTCGCC CTTTCATGAG CGGCCCTCCA
3181 AAAAAGAAGA GAAAGGTAGA AGACCCGGGC GGCCGCATGT CCAATTTACT GACCGTACAC
3241 CAAAATTTGC CTGCATTACC GGTCGATGCA ACGAGTGATG AGGTTCGCAA GAACCTGATG
3301 GACATGTTCA GGGATCGCCA GGCGTTTTCT GAGCATACCT GGAAAATGCT TCTGTCCGTT
3361 TGCCGGTCGT GGGCGGCATG GTGCAAGTTG AATAACCGGA AATGGTTTCC CGCAGAACCT
3421 GAAGATGTTC GCGATTATCT TCTATATCTT CAGGCGCGCG GTCTGGCAGT AAAAACTATC
3481 CAGCAACATT TGGGCCAGCT AAACATGCTT CATCGTCGGT CCGGGCTGCC ACGACCAAGT
3541 GACAGCAATG CTGTTTCACT GGTTATGCGG CGGATCCGAA AAGAAAACGT TGATGCCGGT
3601 GAACGTGCAA AACAGGCTCT AGCGTTCGAA CGCACTGATT TCGACCAGGT TCGTTCACTC
3661 ATGGAAAATA GCGATCGCTG CCAGGATATA CGTAATCTGG CATTTCTGGG GATTGCTTAT
3721 AACACCCTGT TACGTATAGC CGAAATTGCC AGGATCAGGG TTAAAGATAT CTCACGTACT
3781 GACGGTGGGA GAATGTTAAT CCATATTGGC AGAACGAAAA CGCTGGTTAG CACCGCAGGT
3841 GTAGAGAAGG CACTTAGCCT GGGGGTAACT AAACTGGTCG AGCGATGGAT TTCCGTCTCT
3901 GGTGTAGCTG ATGATCCGAA TAACTACCTG TTTTGCCGGG TCAGAAAAAA TGGTGTTGCC
3961 GCGCCATCTG CCACCAGCCA GCTATCAACT CGCGCCCTGG AAGGGATTTT TGAAGCAACT
4021 CATCGATTGA TTTACGGCGC TAAGGATGAC TCTGGTCAGA GATACCTGGC CTGGTCTGGA
4081 CACAGTGCCC GTGTCGGAGC CGCGCGAGAT ATGGCCCGCG CTGGAGTTTC AATACCGGAG
4141 ATCATGCAAG CTGGTGGCTG GACCAATGTA AATATTGTCA TGAACTATAT CCGTAACCTG
4201 GATAGTGAAA CAGGGGCAAT GGTGCGCCTG CTGGAAGATG GCGATTAGCC ATgtacCTCg
4261 agcttggggt ggggttgtta tgtgttgcga acgagagtgg atctctctcg acatttcctt
4321 atttttttttt tctattgtta acacttacaa cgaaacttcg gaagagaagt ttcctcattc
4381 gaaacgagga gtcaaactcc tttccttgct tttgtgacat gcatgattat tctttcattt
4441 actgacgtaa cgatgtaaaa cacacagaag aagcatgaca cacagcaaag aattgttcgt
4501 taataaaacg ttgatgaaaa ctttgaaaac ataagaactt gcacttttat tctataattc
4561 gtgaaagctt gcaccgattg tctttcatta ttcaatgtaa tgtactgaaa ggtgattttt
4621 cgcacttgta tactctagaa ctgaagtatt ctaacaatac gtcacctttta ggtccattcc
4681 aggataaaat acacaagtga aggtagttgt acaaagtact tagactaacc caggtttcaa
4741 aaaagataac gacgaattga gcgtaactgc acaaagcccg ttcattgtca acatatcgtt
4801 caggggtctt caaactttat tatcagcccg cgggccgctt agagcaccat cttgatgtat
4861 cctttatcta gtggcttatc gtttatgtct cgtttggcta agaaaatact aataacatat
4921 tcaatttacg gagcggtacc atggtacaaa cgtcatactg ctagacttga caacatgccc
4981 atcgagggtt cttacctcaa atggaccgat cccttgtaac tggcggcgta aactcagtaa
5041 attccaaaag ccagtatagg ccaccatgac cgtctaacta ttccaaaaag gagaatattt
5101 gaaatttcta tatctatagt attattacta tatctatatg tttctttagt acaatattag
5161 agcttgatta ttttcctgaa aattgtaaaa tatgcatgtt agatggttga tacatcgttt
5221 taaaaaaaac tgctgttaat ggCTCGAGCA CCCAACTTTT CTATACAAAG TTGGCATTAT
5281 AAGAAAGCAT TGCTTATCAA TTTGTTGCAA CGAACAGGTC ACTATCAGTC AAAATAAAAT
5341 CATTATTTGC CATCCAGCTG CAGGGCGGCC GCGATATCCC CTATAGTGAG TCGTATTACA
5401 TGGTCATAGC TGTTTCCTGG CAGCTCTGGC CCGTGTCTCA AAATCTCTGA TGTTACATTG
5461 CACAAGATAA AAATATATCA TCATGAACAA TAAAACTGTC TGCTTACATA AACAGTAATA
5521 CAAGGGGTGT TATGAGCCAT ATTCAACGGG AAACGTCGAG GCCGCGATTA AATTCCAACA
5581 TGGATGCTGA TTTATATGGG TATAAATGGG CTCGCGATAA TGTCGGGCAA TCAGGTGCGA
5641 CAATCTATCG CTTGTATGGG AAGCCCGATG CGCCAGAGTT GTTTCTGAAA CATGGCAAAG
5701 GTAGCGTTGC CAATGATGTT ACAGATGAGA TGGTCAGACT AAACTGGCTG ACGGAATTTA
5761 TGCCTCTTCC GACCATCAAG CATTTTATCC GTACTCCTGA TGATGCATGG TTACTCACCA
5821 CTGCGATCCC CGGAAAAACA GCATTCCAGG TATTAGAAGA ATATCCTGAT TCAGGTGAAA
5881 ATATTGTTGA TGCGCTGGCA GTGTTCCTGC GCCGGTTGCA TTCGATTCCT GTTTGTAATT
5941 GTCCTTTTAA CAGCGATCGC GTATTTCGTC TCGCTCAGGC GCAATCACGA ATGAATAACG
6001 GTTTGGTTGA TGCGAGTGAT TTTGATGACG AGCGTAATGG CTGGCCTGTT GAACAAGTCT
6061 GGAAAGAAAT GCATAAACTT TTGCCATTCT CACCGGATTC AGTCGTCACT CATGGTGATT
6121 TCTCACTTGA TAACCTTATT TTTGACGAGG GGAAATTAAT AGGTTGTATT GATGTTGGAC
6181 GAGTCGGAAT CGCAGACCGA TACCAGGATC TTGCCATCCT ATGGAACTGC CTCGGTGAGT
6241 TTTCTCCTTC ATTACAGAAA CGGCTTTTTC AAAAATATGG TATTGATAAT CCTGATATGA
6301 ATAAATTGCA GTTTCATTTG ATGCTCGATG AGTTTTTCTA ATCAGAATTG GTTAATTGGT
6361 TGTAACACTG GCAGAGCATT ACGCTGACTT GACGGGACGG CGCAAGCTCA TGACCAAAAT
6421 CCCTTAACGT GAGTTACGCG TCGTTCCACT GAGCGTCAGA CCCCGTAGAA AAGATCAAAG
6481 GATCTTCTTG AGATCCTTTT TTTCTGCGCG TAATCTGCTG CTTGCAAACA AAAAAACCAC
6541 CGCTACCAGC GGTGGTTTGT TTGCCGGATC AAGAGCTACC AACTCTTTTT CCGAAGGTAA
6601 CTGGCTTCAG CAGAGCGCAG ATACCAAATA CTGTTCTTCT AGTGTAGCCG TAGTTAGGCC
6661 ACCACTTCAA GAACTCTGTA GCACCGCCTA CATACCTCGC TCTGCTAATC CTGTTACCAG
6721 TGGCTGCTGC CAGTGGCGAT AAGTCGTGTC TTACCGGGTT GGACTCAAGA CGATAGTTAC
```

```
     6781 CGGATAAGGC GCAGCGGTCG GGCTGAACGG GGGGTTCGTG CACACAGCCC AGCTTGGAGC
     6841 GAACGACCTA CACCGAACTG AGATACCTAC AGCGTGAGCT ATGAGAAAGC GCCACGCTTC
     6901 CCGAAGGGAG AAAGGCGGAC AGGTATCCGG TAAGCGGCAG GGTCGGAACA GGAGAGCGCA
     6961 CGAGGGAGCT TCCAGGGGGA AACGCCTGGT ATCTTTATAG TCCTGTCGGG TTTCGCCACC
     7021 TCTGACTTGA GCGTCGATTT TTGTGATGCT CGTCAGGGGG GCGGAGCCTA TGGAAAAACG
     7081 CCAGCAACGC GGCCTTTTTA CGGTTCCTGG CCTTTTGCTG GCCTTTTGCT CACATGTT
//
```

pDSAR-Cre-F2A-IsceI (transgenesis plasmid that produced the C2S transgenic line, expressing Cre and I-SceI)

```
LOCUS       pDSAR-Cre-F2A-Is        9463 bp    DNA     circular
FEATURES             Location/Qualifiers
     misc_feature    complement(7724..7742)
                     /note="M13R"
     misc_feature    6591..7272
                     /note="DsRed"
     misc_feature    6332..6381
                     /note="3xP3 element"
     misc_feature    6421..6428
                     /note="TATA"
     misc_feature    5755..5981
                     /note="SV40 term"
     misc_feature    5982..6258
                     /note="attB"
     misc_feature    7274..7514
                     /note="SV40 term"
     misc_feature    complement(286..1275)
                     /dnas_title="3'UTR_Vasa"
                     /vntifkey="21"
                     /label=3'UTR_Vasa
     misc_feature    complement(1285..2111)
                     /dnas_title="I-SceI CDS"
                     /vntifkey="21"
                     /label=I-SceI CDS
     misc_feature    complement(2197..3276)
                     /note="Cre ORF"
     misc_feature    complement(2044..2057)
                     /note="NLS"
     misc_feature    complement(1996..2022)
                     /note="HA"
     misc_feature    complement(2186..2195)
                     /note="furin site"
     misc_feature    complement(2116..2178)
                     /note="2A peptide"
     misc_feature    complement(3451..5739)
                     /dnas_title="5'Vas_Int"
                     /vntifkey="21"
                     /label=5'Vas_Int
     misc_feature    3368..3381
                     /note="NLS"
     misc_feature    complement(3319..3344)
                     /note="HA epitope"
     source          1..9463
                     /dnas_title="pDSAR-Cre-F2A-Isce-2"

ORIGIN
        1 CTTTCCTGCG TTATCCCCTG ATTCTGTGGA TAACCGTATT ACCGCCTTTG AGTGAGCTGA
       61 TACCGCTCGC CGCAGCCGAA CGACCGAGCG CAGCGAGTCA GTGAGCGAGG AAGCGGAAGA
      121 GCGCCCAATA CGCAAACCGC CTCTCCCCGC GCGTTGGCCG ATTCATTAAT GCAGCTGGCA
      181 CGACAGGTTT CCCGACTGGA AAGCGGGCAG TGAGCGCAAC GCAATTAATA CGCGTACCGc
      241 tagCGGGGGG CCCGGTACCA ACTTTATTAT ACaaagttgt ctcgagaaaa tgtggccatt
      301 aacagcagtt ttttttaaaa cgatgtatca accatctaac atgcatattt tacaattttc
      361 aggaaaataa tcaagctcta atattgtact aaagaaacat atagatatag taataatact
```

```
 421 atagatatag aaatttcaaa tattctcctt tttggaatag ttagacggtc atggtggcct
 481 atactggctt ttggaattta ctgagtttac gccgccagtt acaagggatc ggtccatttg
 541 aggtaagaac cctcgatggg catgttgtca agtctagcag tatgacgttt gtaccatggt
 601 accgctccgt aaattgaata tgttattagt attttcttag ccaaacgaga cataaacgat
 661 aagccactag ataaaggata catcaagatg gtgctctaag cggcccgcgg gctgataata
 721 aagtttgaag accccctgaac gatatgttga caatgaacgg gctttgtgca gttacgctca
 781 attcgtcgtt atcttttttg aaacctgggt tagtctaagt actttgtaca actaccttca
 841 cttgtgtatt ttatcctgga atggacctaa aggtgacgta ttgttagaat acttcagttc
 901 tagagtatac aagtgcgaaa aatcaccttt cagtacatta cattgaataa tgaaagacaa
 961 tcggtgcaag ctttcacgaa ttatagaata aaagtgcaag ttcttatgtt ttcaaagttt
1021 tcatcaacgt tttattaacg aacaattctt tgctgtgtgt catgcttctt ctgtgtgttt
1081 tacatcgtta cgtcagtaaa tgaaagaata atcatgcatg tcacaaaagc aaggaaagga
1141 gtttgactcc tcgtttcgaa tgaggaaact tctcttccga agtttcgttg taagtgttaa
1201 caatagaaaa aaaaaataag gaaatgtcga gagagatcca ctctcgttcg caacacataa
1261 caacccccacc ccaagctcga gctcttattt caggaaagtt tcggaggaga tagtgttcgg
1321 cagtttgtac atcatctgcg ggatcaggta cggtttgatc aggttgtaga agatcaggta
1381 agacatagaa tcgatgtaga tgatcggttt gtttttgttg atttttacgt aacagttcag
1441 ttggaatttg ttacgcagac ccttaaccag gtattctact tcttcgaaag tgaaagactg
1501 ggtgttcagt acgatcgatt tgttggtaga gtttttgttg taatcccatt taccaccatc
1561 atccatgaac cagtatgcca gagacatcgg ggtcaggtag ttttcaacca ggttgttcgg
1621 gatggttttt ttgttgttaa cgatgaacag gttagccagt ttgttgaaag cttggtgttt
1681 gaaagtctgg gcgccccagg tgattaccag gttacccagg tggttaacac gttctttttt
1741 gtgcggcggg gacagtaccc actgatcgta cagcagacat acgtggtcca tgtatgcttt
1801 gtttttccac tcgaactgca tacagtaggt tttaccttca tcacgagaac ggatgtaagc
1861 atcacccagg atcagaccga tacctgcttc gaactgttcg atgttcagtt cgatcagctg
1921 ggatttgtat tctttcagca gtttagagtt cggacccagg ttcattacct ggtttttttt
1981 gatgtttttc atacccgcat agtcaggaac atcgtatggg tacataaatc gtgggtcttc
2041 tacttttcgt tttttttttg gtggtgctgc atgctgcgca tcggccgtag cttcgtcgtc
2101 tgatgatgat cCTGGTCCGG GATTCGACTC CACGTCTCCA GCCAGCTTCA GCAGGTCGAA
2161 GTTCAGGGTC TGCTTCACCG GAGCACGCTT GGCGCGATCG CCATCTTCCA GCAGGCGCAC
2221 CATTGCCCCT GTTTCACTAT CCAGGTTACG GATATAGTTC ATGACAATAT TTACATTGGT
2281 CCAGCCACCA GCTTGCATGA TCTCCGGTAT TGAAACTCCA GCGCGGGCCA TATCTCGCGC
2341 GGCTCCGACA CGGGCACTGT GTCCAGACCA GGCCAGGTAT CTCTGACCAG AGTCATCCTT
2401 AGCGCCGTAA ATCAATCGAT GAGTTGCTTC AAAAATCCCT TCCAGGGCGC GAGTTGATAG
2461 CTGGCTGGTG GCAGATGGCG CGGCAACACC ATTTTTTCTG ACCCGGCAAA ACAGGTAGTT
2521 ATTCGGATCA TCAGCTACAC CAGAGACGGA AATCCATCGC TCGACCAGTT TAGTTACCCC
2581 CAGGCTAAGT GCCTTCTCTA CACCTGCGGT GCTAACCAGC GTTTTCGTTC TGCCAATATG
2641 GATTAACATT CTCCCACCGT CAGTACGTGA GATATCTTTA ACCCTGATCC TGGCAATTTC
2701 GGCTATACGT AACAGGGTGT TATAAGCAAT CCCCAGAAAT GCCAGATTAC GTATATCCTG
2761 GCAGCGATCG CTATTTTCCA TGAGTGAACG AACCTGGTCG AAATCAGTGC GTTCGAACGC
2821 TAGAGCCTGT TTTGCACGTT CACCGGCATC AACGTTTTCT TTTCGGATCC GCCGCATAAC
2881 CAGTGAAACA GCATTGCTGT CACTTGGTCG TGGCAGCCCG GACCGACGAT GAAGCATGTT
2941 TAGCTGGCCC AAATGTTGCT GGATAGTTTT TACTGCCAGA CCGCGCGCCT GAAGATATAG
3001 AAGATAATCG CGAACATCTT CAGGTTCTGC GGGAAACCAT TTCCGGTTAT TCAACTTGCA
3061 CCATGCCGCC CACGACCGGC AAACGGACAG AAGCATTTTC CAGGTATGCT CAGAAAACGC
3121 CTGGCGATCC CTGAACATGT CCATCAGGTT CTTGCGAACC TCATCACTCG TTGCATCGAC
3181 CGGTAATGCA GGCAAATTTT GGTGTACGGT CAGTAAATTG GACATGCGGC CGCCCGGGTC
3241 TTCTACCTTT CTCTTCTTTT TTGGAGGGCC GCTCATGAAA GGGCGAATTC cctggttttt
3301 tttgatgttt ttcatacccg catagtcagg aacatcgtat gggtacataa atcgtgggtc
3361 ttctactttt cgtttttttt ttggtggtgc tgcatgctgc gcatcggccg tagcttcgtc
3421 gtctgatgat gatcccatgt ttccggatcc gtttcctttc tttattcacc ggaagatgac
3481 aattctcctt atgggaatgt acgattcgag atctgtgtgt ctcccgaaga tgaaaaaagc
3541 tgttggtttc aaccaaaaaa cgtgtccgtt gctcgaagcg cgactaacgg cgactctctt
3601 tcgactagca atggcgtccg caggcacaaa ccgagaggaa tagtggaaca tgcaatgcag
3661 cagcagcagc aacagcagca gcacgtggag agagagaaag aaagatggtc gaaatattac
3721 aaaaaataat tgccgttgca cacacacaca cacacacaca cacacacaca cacacacaca
3781 cacacacaca cacacacaca cacacacaca cacacacaca cacacacaca cactacacca
3841 ccaatatcga cgaccaccgc tctacccgca cccattgcaa cctaaagaga cgatggtatt
3901 ttccagcgaa attttcattg tccattgcaa gcgacctcac acaccgccac cgaagtagcc
3961 tttgaagcac gcgcgcgtgt gaaggcgtgt gcctctgtgt gtgtaggtga atatcactgt
4021 gtacactgcg ctaagccagt ggcgcgcgcg cttttcgcat caattattgc cggatgaacg
4081 acaacgagat gctcttcgcg gggcccgctg tcccggaacg tgcacgaaag caccatttct
4141 cctctcggaa actccacatc tcggaaaggc ttcccaacta tgccccaacg actctcttat
4201 ctattgcata tcgaagactt ttgaatccgc tccagtgcca actgcggttc atgaaacacg
4261 aaactgtttt tctcccctga ttcgagcaaa caagagcacc atggtaagct gtggaaataa
```

4321 cgccatcctt ctggatatct tgtaacgaaa aaggccaagg actgtgattc tactaacctc
4381 actctggaac tgatatgaga gcacacagag ccacacaacc actccacgac gggatacgct
4441 tggattaaaa ctaaaccgct tccgtacgtc gacaccgact ggcccctata ccgcttctca
4501 aataacgacg atctcagcta aaatgccccc cacacacatg catgcgcagc acacttgttc
4561 acttccactt ccttgcctgc tctgtgtgcg gtggtggata cgtgctgtgc tgtaagaaca
4621 cttcacacag aaaggaaggg acagagcaaa ccaacacact gccaccgctc ttctcgtgcc
4681 atttccttag aaaattaatc caaaaactca actgcgccac acaccacggc agccacggca
4741 gcctgctatc aaaagggggg aggcgcgcaa catgcacaac acacgataca caccacggaa
4801 tgtttacgaa cagcgtgcga acaggcaatt tcggtaccgg cggcaacagc cgattttgac
4861 agagcgctca acacacacac gcacacactc taataccagc aacgcgtgcg aaggcttttg
4921 tttcgcactg ccagtgtgtt agtgggagca ggtacaaaat gaaacaaagg gcgaatggcg
4981 cgtgctactg aacaatacaa gcgaattcga gaaaaattaa ataagccaag agattatttt
5041 atgagtaaat gaaatgaaat ttaatttaac tgtattttga acataactaa gaaaatacta
5101 atcaactgga tgaaataata taacatcaca aaacaataat tcaatttgaa atcagttacg
5161 ttagttttgc cgaaatgaga gatttttttc acaaacactt cactaacttc taacaaacaa
5221 acatttgttc tataaaacta gttcacttac ctgagttttg aagtactatt cggctggtta
5281 tttcacttgc aaaagcaatt ggctaattta gcgctaagca cctcagcaga aacctccgac
5341 tcgccgcgag tgctccgagc aaaagactgc taaaagctgg aatttgcacg gacacggcct
5401 actcgtccgt cgtactgtgc aactaggatg gaagatttcg atacgggact ctacagggag
5461 ggtttatatg tgttttataa tgctgcatgt atgtttttat gtataaaata tgcaaaaagt
5521 atgtcttttt agtttattat aacaaataaa atacaataca aattataaaa caatcaattt
5581 ctcaacggtt ttgtttgcag cgagttgtga tttgcttgaa aacaaagcgc acgctgtaat
5641 tttcccgccc agttcggcat gtcaaacacg tctgacgttc ggcttcccac tgtagctgct
5701 gctgtcatgg aaggaaaaga atttgctcgc gttctacatc gATACCGTCG ACGCTAGACA
5761 TAATCAGCCA TACCACATTT GTAGAGGTTT TACTTGCTTT AAAAAACCTC CCACACCTCC
5821 CCCTGAACCT GAAACATAAA ATGAATGCAA TTGTTGTTGT TAACTTGTTT ATTGCAGCTT
5881 ATAATGGTTA CAAATAAAGC AATAGCATCA CAAATTTCAC AAATAAAGCA TTTTTCTTCA
5941 CTGCATTCTA GTTGTGGTTT GTCCAAACTC ATCAATGTAT CtcgaCGATG TAGGTCACaG
6001 TCTCGAAGCC GCGGTGCGGG TGCCAGGGCG TGCCCTTGGG CTCCCCGGGC GCGTACTCCA
6061 CCTCACCCAT CTGGTCCATC ATGATGAACG GGTCGAGGTG GCGGTAGTTG ATCCCGGCGA
6121 ACGCGCGGCG CACCGGGAAG CCCTCGCCCT CGAAACCGCT GGGCGCGGTG GTCACGGTGA
6181 GCACGGGACG TGCGACGGCG TCGGCGGGTG CGGATACGCG GGGCAGCGTC AGCGGGTTCT
6241 CGACGGTCAC GGCGGGCAat tCCTGCAGAC TTCCGGTATC TCGCGTTTGT TTGATCGCAC
6301 GGTTCCCACA ATGGTTAATT CGAGCTCGCC CGGGGATCTA ATTCAATTAG AGACTAATTC
6361 AATTAGAGCT AATTCAATTA GGATCCAAGC TTATCGATTT CGAACCCTCG ACCGCCGGAG
6421 TATAAATAGA GGCGCTTCGT CTACGGAGCG ACAATTCAAT TCAAACAAGC AAAGTGAACA
6481 CGTCGCTAAG CGAAAGCTAA GCAAATAAAC AAGCGCAGCT GAACAAGCTA AACAATCGGG
6541 GTACCGCTAG AGTCGACGGT ACCGCGGGCC CGGGATCCAC CGGTCGCCAC CATGGTGCGC
6601 TCCTCCAAGA ACGTCATCAA GGAGTTCATG CGCTTCAAGG TGCGCATGGA GGGCACCGTG
6661 AACGGCCACG AGTTCGAGAT CGAGGGCGAG GGCGAGGGCC GCCCCTACGA GGGCCACAAC
6721 ACCGTGAAGC TGAAGGTGAC CAAGGGCGGC CCCCTGCCCT TCGCCTGGGA CATCCTGTCC
6781 CCCCAGTTCC AGTACGGCTC CAAGGTGTAC GTGAAGCACC CCGCCGACAT CCCCGACTAC
6841 AAGAAGCTGT CCTTCCCCGA GGGCTTCAAG TGGGAGCGCG TGATGAACTT CGAGGACGGC
6901 GGCGTGGTGA CCGTGACCCA GGACTCCTCC CTGCAGGACG GCTGCTTCAT CTACAAGGTG
6961 AAGTTCATCG GCGTGAACTT CCCCTCCGAC GGCCCCGTAA TGCAGAAGAA GACCATGGGC
7021 TGGGAGGCCT CCACCGAGCG CCTGTACCCC CGCGACGGCG TGCTGAAGGG CGAGATCCAC
7081 AAGGCCCTGA AGCTGAAGGA CGGCGGCCAC TACCTGGTGG AGTTCAAGTC CATCTACATG
7141 GCCAAGAAGC CCGTGCAGCT GCCCGGCTAC TACTACGTGG ACTCCAAGCT GGACATCACC
7201 TCCCACAACG AGGACTACAC CATCGTGGAg CAGTACGAGC GCACCGAGGG CCGCCACCAC
7261 CTGTTCCTGT AGCGGCCGCG ACTCTAGATC ATAATCAGCC ATACCACATT TGTAGAGGTT
7321 TTACTTGCTT TAAAAAACCT CCCACACCTC CCCCTGAACC TGAAACATAA AATGAATGCA
7381 ATTGTTGTTG TTAACTTGTT TATTGCAGCT TATAATGGTT ACAAATAAAG CAATAGCATC
7441 ACAAATTTCA CAAATAAAGC ATTTTTTTCA CTGCATTCTA GTTGTGGTTT GTCCAAACTC
7501 ATCAATGTAT CTTAAAGCTT ATCGATACGC GTACGGCGCG CCTAGAGCGG CCGCCACCGC
7561 GGTGGAGCTC GAGTACCCAG CTTTCTTGTA CAAAGTTGGC ATTATAAGAA AGCATTGCTT
7621 ATCAATTTGT TGCAACGAAC AGGTCACTAT CAGTCAAAAT AAAATCATTA TTTGCCATCC
7681 AGCTGCAGGG CGGCCGCGAT ATCCCCTATA GTGAGTCGTA TTACATGGTC ATAGCTGTTT
7741 CCTGGCAGCT CTGGCCCGTG TCTCAAAATC TCTGATGTTA CATTGCACAA GATAAAAATA
7801 TATCATCATG AACAATAAAA CTGTCTGCTT ACATAAACAG TAATACAAGG GGTGTTATGA
7861 GCCATATTCA ACGGGAAACG TCGAGGCCGC GATTAAATTC CAACATGGAT GCTGATTTAT
7921 ATGGGTATAA ATGGGCTCGC GATAATGTCG GGCAATCAGG TGCGACAATC TATCGCTTGT
7981 ATGGGAAGCC CGATGCGCCA GAGTTGTTTC TGAAACATGG CAAAGGTAGC GTTGCCAATG
8041 ATGTTACAGA TGAGATGGTC AGACTAAACT GGCTGACGGA ATTTATGCCT CTTCCGACCA
8101 TCAAGCATTT TATCCGTACT CCTGATGATG CATGGTTACT CACCACTGCG ATCCCCGGAA
8161 AAACAGCATT CCAGGTATTA GAAGAATATC CTGATTCAGG TGAAAATATT GTTGATGCGC

```
8221 TGGCAGTGTT CCTGCGCCGG TTGCATTCGA TTCCTGTTTG TAATTGTCCT TTTAACAGCG
8281 ATCGCGTATT TCGTCTCGCT CAGGCGCAAT CACGAATGAA TAACGGTTTG GTTGATGCGA
8341 GTGATTTTGA TGACGAGCGT AATGGCTGGC CTGTTGAACA AGTCTGGAAA GAAATGCATA
8401 AACTTTTGCC ATTCTCACCG GATTCAGTCG TCACTCATGG TGATTTCTCA CTTGATAACC
8461 TTATTTTTGA CGAGGGGAAA TTAATAGGTT GTATTGATGT TGGACGAGTC GGAATCGCAG
8521 ACCGATACCA GGATCTTGCC ATCCTATGGA ACTGCCTCGG TGAGTTTTCT CCTTCATTAC
8581 AGAAACGGCT TTTTCAAAAA TATGGTATTG ATAATCCTGA TATGAATAAA TTGCAGTTTC
8641 ATTTGATGCT CGATGAGTTT TTCTAATCAG AATTGGTTAA TTGGTTGTAA CACTGGCAGA
8701 GCATTACGCT GACTTGACGG GACGGCGCAA GCTCATGACC AAAATCCCTT AACGTGAGTT
8761 ACGCGTCGTT CCACTGAGCG TCAGACCCCG TAGAAAAGAT CAAAGGATCT TCTTGAGATC
8821 CTTTTTTTCT GCGCGTAATC TGCTGCTTGC AAACAAAAAA ACCACCGCTA CCAGCGGTGG
8881 TTTGTTTGCC GGATCAAGAG CTACCAACTC TTTTTCCGAA GGTAACTGGC TTCAGCAGAG
8941 CGCAGATACC AAATACTGTT CTTCTAGTGT AGCCGTAGTT AGGCCACCAC TTCAAGAACT
9001 CTGTAGCACC GCCTACATAC CTCGCTCTGC TAATCCTGTT ACCAGTGGCT GCTGCCAGTG
9061 GCGATAAGTC GTGTCTTACC GGGTTGGACT CAAGACGATA GTTACCGGAT AAGGCGCAGC
9121 GGTCGGGCTG AACGGGGGGT TCGTGCACAC AGCCCAGCTT GGAGCGAACG ACCTACACCG
9181 AACTGAGATA CCTACAGCGT GAGCTATGAG AAAGCGCCAC GCTTCCCGAA GGGAGAAAGG
9241 CGGACAGGTA TCCGGTAAGC GGCAGGGTCG GAACAGGAGA GCGCACGAGG GAGCTTCCAG
9301 GGGGAAACGC CTGGTATCTT TATAGTCCTG TCGGGTTTCG CCACCTCTGA CTTGAGCGTC
9361 GATTTTTGTG ATGCTCGTCA GGGGGGCGGA GCCTATGGAA AAACGCCAGC AACGCGGCCT
9421 TTTTACGGTT CCTGGCCTTT TGCTGGCCTT TTGCTCACAT GTT
//
```

File S3

DNA sequence of transgenesis plasmids used to generate reporter lines.

pAttBRfB2-ppo6Tom: Plasmid used to generate *PPO6*-tdTomato line

```
LOCUS       attBRfB2PPO6_Tom        7852 bp    DNA     circular
FEATURES             Location/Qualifiers
     misc_feature    complement(2216..2500)
                     /note="attB"
     misc_feature    2138..2156
                     /note="M13R"
     misc_feature    7167..7186
                     /note="attB2"
     misc_feature    complement(2541..3492)
                     /note="PPO9 mRNA"
     misc_feature    4014..4052
                     /note="PPO6 5'UTR"
     misc_feature    3968..3975
                     /note="putative TATA box"
     misc_feature    4057..4078
                     /note="attB4"
     misc_feature    5671..5870
                     /note="sv40 terminator"
     gene            6179..6895
                     /note="EYFP (Clontech)"
     misc_feature    6008..6015
                     /note="TATA"
     misc_feature    6902..7156
                     /note="SV40 term sequences"
     misc_feature    4108..5536
                     /note="tdTomato"
     misc_feature    5602..5623
                     /note="attB3"
     misc_feature    2516..2537
                     /note="attB1"
     source          1..7852
                     /dnas_title="attBRfB2PPO6_Tomato_Sv40YFP"
     misc_feature    5919..5968
                     /note="3xP3 binding site"
ORIGIN
        1 GTGGCACTTT TCGGGGAAAT GTGCGCGGAA CCCCTATTTG TTTATTTTTC TAAATACATT
       61 CAAATATGTA TCCGCTCATG AGACAATAAC CCTGATAAAT GCTTCAATAA TATTGAAAAA
      121 GGAAGAGTAT GAGTATTCAA CATTTCCGTG TCGCCCTTAT TCCCTTTTTT GCGGCATTTT
      181 GCCTTCCTGT TTTTGCTCAC CCAGAAACGC TGGTGAAAGT AAAAGATGCT GAAGATCAGT
      241 TGGGTGCACG AGTGGGTTAC ATCGAACTGG ATCTCAACAG CGGTAAGATC CTTGAGAGTT
      301 TTCGCCCCGA AGAACGTTTT CCAATGATGA GCACTTTTAA AGTTCTGCTA TGTGGCGCGG
      361 TATTATCCCG TATTGACGCC GGGCAAGAGC AACTCGGTCG CCGCATACAC TATTCTCAGA
      421 ATGACTTGGT TGAGTACTCA CCAGTCACAG AAAAGCATCT TACGGATGGC ATGACAGTAA
      481 GAGAATTATG CAGTGCTGCC ATAACCATGA GTGATAACAC TGCGGCCAAC TTACTTCTGA
      541 CAACGATCGG AGGACCGAAG GAGCTAACCG CTTTTTTGCA CAACATGGGG GNATCATGTA
      601 ACTCGCCTTN GATCGTTGGG AACCGGAGCT GAATGAAGCC ATACCAAACG ACGAGCGTGA
      661 CACCACGATG CCTGTAGCAA TGGCAACAAC GTTGCGCAAA CTATTAACTG GCGAACTACT
      721 TACTCTAGCT TCCCGGCAAC AATTAATAGA CTGGATGGAG GCGGATAAAG TTGCAGGACC
      781 ACTTCTGCGC TCGGCCCTTC CGGCTGGCTG GTTTATTGCT GATAAATCTG GAGCCGGTGA
      841 GCGTGGGTCT CGCGGTATCA TTGCAGCACT GGGGCCAGAT GGTAAGCCCT CCCGTATCGT
      901 AGTTATCTAC ACGACGGGGA GTCAGGCAAC TATGGATGAA CGAAATAGAC AGATCGCTGA
      961 GATAGGTGCC TCACTGATTA AGCATTGGTA ACTGTCAGAC CAAGTTTACT CATATATACT
     1021 TTAGATTGAT TTAAAACTTC ATTTTTAATT TAAAAGGATC TAGGTGAAGA TCCTTTTTGA
     1081 TAATCTCATG ACCAAAATCC CTTAACGTGA GTTTTCGTTC CACTGAGCGT CAGACCCCGT
     1141 AGAAAAGATC AAAGGATCTT CTTGAGATCC TTTTTTTCTG CGCGTAATCT GCTGCTTGCA
     1201 AACAAAAAAA CCACCGCTAC CAGCGGTGGT TTGTTTGCCG GATCAAGAGC TACCAACTCT
     1261 TTTTCCGAAG GTAACTGGCT TCAGCAGAGC GCAGATACCA AATACTGTCC TTCTAGTGTA
     1321 GCCGTAGTTA GGCCACCACT TCAAGAACTC TGTAGCACCG CCTACATACC TCGCTCTGCT
     1381 AATCCTGTTA CCAGTGGCTG CTGCCAGTGG CGATAAGTCG TGTCTTACCG GGTTGGACTC
     1441 AAGACGATAG TTACCGGATA AGGCGCAGCG GTCGGGCTGA ACGGGGGGTT CGTGCACACA
     1501 GCCCAGCTTG GAGCGAACGA CCTACACCGA ACTGAGATAC CTACAGCGTG AGCTATGAGA
     1561 AAGCGCCACG CTTCCCGAAG GGAGAAAGGC GGACAGGTAT CCGGTAAGCG GCAGGGTCGG
     1621 AACAGGAGAG CGCACGAGGG AGCTTCCAGG GGGAAACGCC TGGTATCTTT ATAGTCCTGT
     1681 CGGGTTTCGC CACCTCTGAC TTGAGCGTCG ATTTTTGTGA TGCTCGTCAG GGGGGCGGAG
     1741 CCTATGGAAA AACGCCAGCA ACGCGGCCTT TTTACGGTTC CTGGCCTTTT GCTGGCCTTT
     1801 TGCTCACATG TTCTTTCCTG CGTTATCCCC TGATTCTGTG GATAACCGTA TTACCGCCTT
     1861 TGAGTGAGCT GATACCGCTC GCCGCAGCCG AACGACCGAG CGCAGCGAGT CAGTGAGCGA
     1921 GGAAGCGGAA GAGCGCCCAA TACGCAAACC GCCTCTCCCC GCGCGTTGGC CGATTCATTA
     1981 ATGCAGCTGG CACGACAGGT TTCCCGACTG GAAAGCGGGC AGTGAGCGCA ACGCAATTAA
     2041 TGTGAGTTAG CTCACTCATT AGGCACCCCA GGCTTTACAC TTTATGCTTC CGGCTCGTAT
```

```
2101 GTTGTGTGGA ATTGTGAGCG GATAACAATT TCACACAGGA AACAGCTATG ACCATGATTA
2161 CGCCAAGCTC GAAATTAACC CTCACTAAAG GGAACAAAAG CTGGCTAGAA CTAGTGTCGA
2221 CATGCCCGCC GTGACCGTCG AGAACCCGCT GACGCTGCCC CGCGTATCCG CACCCGCCGA
2281 CGCCGTCGCA CGTCCCGTGC TCACCGTGAC CACCGCGCCC AGCGGTTTCG AGGGCGAGGG
2341 CTTCCCGGTG CGCCGCGCGT TCGCCGGGAT CAACTACCGC CACCTCGACC CGTTCATCAT
2401 GATGGACCAG ATGGGTGAGG TGGAGTACGC GCCCGGGGAG CCCAAGGGCA CGCCCTGGCA
2461 CCCGCACCGC GGCTTCGAGA CCGTGACCTA CATCGTCGAC ACTAGTGgat catcaacaag
2521 tttGTACAAA AAAGCAGGCT GGACGCAACG GTGCAGATTA CGAGCGGTAA GGCGGCCAGA
2581 AATAGATTGC TAACCTTCTG GCAACGGACG CAAGTTGATC TGGGAACGGG GCTAGATTTC
2641 GGACCGCAAG GTAACGTGTT GGCAACgTTC ACCCACATCC AGCACGCACC GTTTGCGTAC
2701 CAAATTATGG TACAAAACGA AACGGCGGAG CAAAAGAAGG GAACTGTTCG CATTTTCCTC
2761 GCCCCGATCT ACGATGCGAA CGGAGAGCAA CTGTTACTGA GCCAGCAGCG TCGGTACATG
2821 CTGGAGATGG ACAAATTTGT CGTCAAGTGT AAGTATACAT TAAGTTGAGC AATACTGTTA
2881 TGGTGCTGCA ATCGTATCGT GTGTTTCCTT CAAGTACATC CTGGCGATAA cCGGATCATT
2941 CGACGATCGG ACCAGTCAAG CGTAACCATA CCGTACGAAA GGACCTTCCG GCGAGTTGAt
3001 GCTTCCAACA TGCCGGGCAC GGAGAGCTTC CGCTTCTGCA ACTGTGGCTG GCCCGATCAT
3061 ATGCTGCTGC CCAAGGGACA TCCCGATGGT CAACCGTTCG ATCTGTTTAT CATGATTTCT
3121 GATTACAAGG ACGATGCTGT AAGCACCGGA TTtAATGAGT GAGTACATTA CGGAATCAGT
3181 ATTGATGAAC TCGGTTTTAA CTTTTGAGTT TTTTTTAAAT ATCTTATGgT CATGTAGGAA
3241 TGAAAACTGT AACGATTCAC ATTCATACTG TGGTCTACGC GATCAGCTGT ATCCGGACCG
3301 TCGTGCGATG GGTTTTCCCT TTGACCGACA GCCGGTTGCC CAGGATCACT TGATGAAGGA
3361 CTTTGTGGGC AGGTTCCCCA ATATGAGTCG TACCGTAGCG GAAGTTATGT TCACCAACAC
3421 TATCATTTCA CGCACGTAAA TGGCATCACG ATAACCGATC GATGAACGAT ACTTGTGGCT
3481 GACCCGTATG ATCGTTTTTT TTATCAACGA GGAACATTAT ATTAAAATGC AATGGAAAAA
3541 TGAGCAATAA AATATATTCA TAcTCAAGCA AATATATACC GCATTgCATT aCtTTCTTAA
3601 AGTTATTTCT ATTTTTTGCA AGTATTATGA TTTTTTTCAA CAATGCGTtT TATTTTCCTC
3661 AACCACCGAT ATGCAATATG GATAAGGCAC ACACATTCCC AGTTATCGTA ATCGCTTACC
3721 AAATGATTGG AATACCGCAA ACATCCACAA AAATAGAACG GATTGATATG TTTTATCAAA
3781 GCTTCATGCA TGTGCATACG CaTGGTTTAC ATTTGCTTCC CATGTACTGC AACAATTGCA
3841 GATTACAATT GCATTGTACC ATAgCATTAT CGCAGATGCA GTGGTCGTAA ACCGCAAAAA
3901 TGGCGATTGG AATTGGAAGA AATTGCATAC AACCAGGCAC CGGCACGTCA AGTGCAAATC
3961 AGAATGATAT ATAAACTATA CCAACTACTT CTGTAGCATC ACAAACGTGA CTGTAACAGT
4021 GACTGGTGGT TCTCGTTTCG TTCTCGCGCG TCcaccCAAC TTTTCTATAC AAAGTTGGTA
4081 CCGGGCCCCC CGCTAGCGTC GACAACCATG GTGAGCAAGG GCGAGGAGGT CATCAAAGAG
4141 TTCATGCGCT TCAAGGTGCG CATGGAGGGC TCCATGAACG GCCACGAGTT CGAGATCGAG
4201 GGCGAGGGCG AGGGCCGCCC CTACGAGGGC ACCCAGACCG CCAAGCTGAA GGTGACCAAG
4261 GGCGGCCCCC TGCCCTTCGC CTGGGACATC CTGTCCCCCC AGTTCATGTA CGGCTCCAAG
4321 GCGTACGTGA AGCACCCCGC CGACATCCCC GATTACAAGA AGCTGTCCTT CCCCGAGGGC
4381 TTCAAGTGGG AGCGCGTGAT GAACTTCGAG GACGGCGGTC TGGTGACCGT GACCCAGGAC
4441 TCCTCCCTGC AGGACGGCAC GCTGATCTAC AAGGTGAAGA TGCGCGGCAC CAACTTCCCC
4501 CCCGACGGCC CCGTAATGCA GAAGAAGACC ATGGGCTGGG AGGCCTCCAC CGAGCGCCTG
4561 TACCCCCGCG ACGGCGTGCT GAAGGGCGAG ATCCACCAGG CCCTGAAGCT GAAGGACGGC
4621 GGCCACTACC TGGTGGAGTT CAAGACCATC TACATGGCCA AGAAGCCCGT GCAACTGCCC
4681 GGCTACTACT ACGTGGACAC CAAGCTGGAC ATCACCTCCC ACAACGAGGA CTACACCATC
4741 GTGGAACAGT ACGAGCGCTC CGAGGGCCGC CACCACCTGT TCCTGGGGCA TGGCACCGGC
4801 AGCACCGGCA GCGGCAGCTC CGGCACCGCC TCCTCCGAGG ACAACAACAT GGCCGTCATC
4861 AAAGAGTTCA TGCGCTTCAA GGTGCGCATG GAGGGCTCCA TGAACGGCCA CGAGTTCGAG
4921 ATCGAGGGCG AGGGCGAGGG CCGCCCCTAC GAGGGCACCC AGACCGCCAA GCTGAAGGTG
4981 ACCAAGGGCG GCCCCCTGCC CTTCGCCTGG GACATCCTGT CCCCCCAGTT CATGTACGGC
5041 TCCAAGGCGT ACGTGAAGCA CCCCGCCGAC ATCCCCGATT ACAAGAAGCT GTCCTTCCCC
5101 GAGGGCTTCA AGTGGGAGCG CGTGATGAAC TTCGAGGACG GCGGTCTGGT GACCGTGACC
5161 CAGGACTCCT CCCTGCAGGA CGGCACGCTG ATCTACAAGG TGAAGATGCG CGGCACCAAC
5221 TTCCCCCCCG ACGGCCCCGT AATGCAGAAG AAGACCATGG GCTGGGAGGC CTCCACCGAG
5281 CGCCTGTACC CCCGCGACGG CGTGCTGAAG GGCGAGATCC ACCAGGCCCT GAAGCTGAAG
5341 GACGGCGGCC ACTACCTGGT GGAGTTCAAG ACCATCTACA TGGCCAAGAA GCCCGTGCAA
5401 CTGCCCGGCT ACTACTACGT GGACACCAAG CTGGACATCA CCTCCCACAA CGAGGACTAC
5461 ACCATCGTGG AACAGTACGA GCGCTCCGAG GGCCGCCACC ACCTGTTCCT GTACGGCATG
5521 GACGAGCTGT ACAAGTAAGA ATTCCTGCAG CCCGGGGGAT CCACTAGTTC TAGAGCGGCC
5581 GCCACCGCGG TGGAGCTCGA GACAACTTTG TATAATAAAG TTGGTACCGG GCCCCCCGCT
5641 AGCGTCGACG GTATCGATAA GCTTGATATC GAATTCTCTA GATCATAATC AGCCATACCA
5701 CATTTGTAGA GGTTTTACTT GCTTTAAAAA ACCTCCCACA CCTCCCCCTG AACCTGAAAC
5761 ATAAAATGAA TGCAATTGTT GTTGTTAACT TGTTTATTGC AGCTTATAAT GGTTACAAAT
5821 AAAGCAATAG CATCACAAAT TTCACAAATA AAGCATTTTT CTTCACTGCA TTCTAGTTGT
5881 GGTTTGTCCA AACTCATCAA TGTATCAAGG GCGAATTCGG GGATCTAATT CAATTAGAGA
5941 CTAATTCAAT TAGAGCTAAT TCAATTAGGA TCCAAGCTTA TCGATTTCGA ACCCTCGACC
6001 GCCGGAGTAT AAATAGAGGC GCTTCGTCTA CGGAGCGACA ATTCAATTCA AACAAGCAAA
6061 GTGAACACGT CGCTAAGCGA AAGCTAAGCA AATAAACAAG CGCAGCTGAA CAAGCTAAAC
6121 AATCGGGGTA CCGCTAGAGT CGACGGTACC GCGGGCCCGG GATCCACCGG TCGCCACCAT
6181 GGTGAGCAAG GGCGAGGAGC TGTTCACCGG GGTGGTGCCC ATCCTGGTCG AGCTGGACGG
6241 CGACGTAAAC GGCCACAAGT TCAGCGTGTC CGGCGAGGGC GAGGGCGATG CCACCTACGG
6301 CAAGCTGACC CTGAAGTTCA TCTGCACCAC CGGCAAGCTG CCCGTGCCCT GGCCCACCCT
6361 CGTGACCACC TTCGGCTACG GCCTGCAGTG CTTCGCCCGC TACCCCGACC ACATGAAGCA
6421 GCACGACTTC TTCAAGTCCG CCATGCCCGA AGGCTACGTC CAGGAGCGCA CCATCTTCTT
6481 CAAGGACGAC GGCAACTACA AGACCCGCGC CGAGGTGAAG TTCGAGGGCG ACACCCTGGT
6541 GAACCGCATC GAGCTGAAGG GCATCGACTT CAAGGAGGAC GGCAACATCC TGGGGCACAA
6601 GCTGGAGTAC AACTACAACA GCCACAACGT CTATATCATG GCCGACAAGC AGAAGAACGG
6661 CATCAAGGTG AACTTCAAGA TCCGCCACAA CATCGAGGAC GGCAGCGTGC AGCTCGCCGA
6721 CCACTACCAG CAGAACACCC CCATCGGCGA CGGCCCCGTG CTGCTGCCCG ACAACCACTA
6781 CCTGAGCTAC CAGTCCGCCC TGAGCAAAGA CCCCAACGAG AAGCGCGATC ACATGGTCCT
6841 GCTGGAGTTC GTGACCGCCG CCGGGATCAC TCTCGGCATG GACGAGCTGT ACAAGTAAAG
6901 CGGCCGCGAC TCTAGATCAT AATCAGCCAT ACCACATTTG TAGAGGTTTT ACTTGCTTTA
```

```
     6961 AAAAACCTCC CACACCTCCC CCTGAACCTG AAACATAAAA TGAATGCAAT TGTTGTTGTT
     7021 AACTTGTTTA TTGCAGCTTA TAATGGTTAC AAATAAAGCA ATAGCATCAC AAATTTCACA
     7081 AATAAAGCAT TTTTTTCACT GCATTCTAGT TGTGGTTTGT CCAAACTCAT CAATGTATCT
     7141 TAAAGCTTAT CGATACctcg agTACCCAGC TTTcttgtac aaagtggttg atCGGTACGT
     7201 ACCCAATTCG CCCTATAGTG AGTCGTATTA CAATTCACTG GCCGTCGTTT TACAACGTCG
     7261 TGACTGGGAA AACCCTGGCG TTACCCAACT TAATCGCCTT GCAGCACATC CCCCTTTCGC
     7321 CAGCTGGCGT AATAGCGAAG AGGCCCGCAC CGATCGCCCT TCCCAACAGT TGCGCAGCCT
     7381 GAATGGCGAA TGGAAATTGT AAGCGTTAAT ATTTTGTTAA AATTCGCGTT AAATTTTTGT
     7441 TAAATCAGCT CATTTTTTAA CCAATAGGCC GAAATCGGCA AAATCCCTTA TAAATCAAAA
     7501 GAATAGACCG AGATAGGGTT GAGTGTTGTT CCAGTTTGGA ACAAGAGTCC ACTATTAAAG
     7561 AACGTGGACT CCAACGTCAA AGGGCGAAAA ACCGTCTATC AGGGCGATGG CCCACTACGT
     7621 GAACCATCAC CCTAATCAAG TTTTTTGGGG TCGAGGTGCC GTAAAGCACT AAATCGGAAC
     7681 CCTAAAGGGA GCCCCCGATT TAGAGCTTGA CGGGGAAAGC CGGCGAACGT GGCGAGAAAG
     7741 GAAGGGAAGA AAGCGAAAGG AGCGGGCGCT AGGGCGCTGG CAAGTGTAGC GGTCACGCTG
     7801 CGCGTAACCA CCACACCCGC CGCGCTTAAT GCGCCGCTAC AGGGCGCGTC AG
//
```

pAttBRfB2-VgGFP: Plasmid used to generate *Vg*-GFP line

```
LOCUS       attBrfB2Vg_GFP_Sv       7475 bp    DNA     circular
FEATURES             Location/Qualifiers
     misc_feature    complement(6834..6854)
                     /note="T7"
     misc_feature    complement(2216..2500)
                     /note="attB"
     misc_feature    2173..2192
                     /note="T3"
     misc_feature    2138..2156
                     /note="M13R"
     gene            5223..5422
                     /note="sv40 terminator"
     misc_feature    5543..5592
                     /note="3xP3 binding sites"
     misc_feature    5154..5175
                     /note="attB3"
     misc_feature    5802..6483
                     /note="DsRed"
     misc_feature    5632..5639
                     /note="TATA"
     misc_feature    6790..6809
                     /note="attB2"
     misc_feature    2516..2537
                     /note="attB1"
     misc_feature    complement(4378..5126)
                     /note="GFP"
     misc_feature    2563..4264
                     /note="Vitellogenin promoter"
     misc_feature    4279..4300
                     /note="attB4"
     source          1..7475
                     /dnas_title="attBrf2Vg_GFP_Sv40dsRED"
     misc_feature    6496..6687
                     /note="SV40 terminator"
ORIGIN
        1 GTGGCACTTT TCGGGGAAAT GTGCGCGGAA CCCCTATTTG TTTATTTTTC TAAATACATT
       61 CAAATATGTA TCCGCTCATG AGACAATAAC CCTGATAAAT GCTTCAATAA TATTGAAAAA
      121 GGAAGAGTAT GAGTATTCAA CATTTCCGTG TCGCCCTTAT TCCCTTTTTT GCGGCATTTT
      181 GCCTTCCTGT TTTTGCTCAC CCAGAAACGC TGGTGAAAGT AAAAGATGCT GAAGATCAGT
      241 TGGGTGCACG AGTGGGTTAC ATCGAACTGG ATCTCAACAG CGGTAAGATC CTTGAGAGTT
      301 TTCGCCCCGA AGAACGTTTT CCAATGATGA GCACTTTTAA AGTTCTGCTA TGTGGCGCGG
      361 TATTATCCCG TATTGACGCC GGGCAAGAGC AACTCGGTCG CCGCATACAC TATTCTCAGA
      421 ATGACTTGGT TGAGTACTCA CCAGTCACAG AAAAGCATCT TACGGATGGC ATGACAGTAA
      481 GAGAATTATG CAGTGCTGCC ATAACCATGA GTGATAACAC TGCGGCCAAC TTACTTCTGA
      541 CAACGATCGG AGGACCGAAG GAGCTAACCG CTTTTTTGCA CAACATGGGG GNATCATGTA
      601 ACTCGCCTTN GATCGTTGGG AACCGGAGCT GAATGAAGCC ATACCAAACG ACGAGCGTGA
      661 CACCACGATG CCTGTAGCAA TGGCAACAAC GTTGCGCAAA CTATTAACTG GCGAACTACT
      721 TACTCTAGCT TCCCGGCAAC AATTAATAGA CTGGATGGAG GCGGATAAAG TTGCAGGACC
      781 ACTTCTGCGC TCGGCCCTTC CGGCTGGCTG GTTTATTGCT GATAAATCTG GAGCCGGTGA
      841 GCGTGGGTCT CGCGGTATCA TTGCAGCACT GGGGCCAGAT GGTAAGCCCT CCCGTATCGT
      901 AGTTATCTAC ACGACGGGGA GTCAGGCAAC TATGGATGAA CGAAATAGAC AGATCGCTGA
      961 GATAGGTGCC TCACTGATTA AGCATTGGTA ACTGTCAGAC CAAGTTTACT CATATATACT
     1021 TTAGATTGAT TTAAAACTTC ATTTTTAATT TAAAAGGATC TAGGTGAAGA TCCTTTTTGA
     1081 TAATCTCATG ACCAAAATCC CTTAACGTGA GTTTTCGTTC CACTGAGCGT CAGACCCCGT
     1141 AGAAAAGATC AAAGGATCTT CTTGAGATCC TTTTTTTCTG CGCGTAATCT GCTGCTTGCA
     1201 AACAAAAAAA CCACCGCTAC CAGCGGTGGT TTGTTTGCCG GATCAAGAGC TACCAACTCT
     1261 TTTTCCGAAG GTAACTGGCT TCAGCAGAGC GCAGATACCA AATACTGTCC TTCTAGTGTA
     1321 GCCGTAGTTA GGCCACCACT TCAAGAACTC TGTAGCACCG CCTACATACC TCGCTCTGCT
```

```
1381 AATCCTGTTA CCAGTGGCTG CTGCCAGTGG CGATAAGTCG TGTCTTACCG GGTTGGACTC
1441 AAGACGATAG TTACCGGATA AGGCGCAGCG GTCGGGCTGA ACGGGGGGTT CGTGCACACA
1501 GCCCAGCTTG GAGCGAACGA CCTACACCGA ACTGAGATAC CTACAGCGTG AGCTATGAGA
1561 AAGCGCCACG CTTCCCGAAG GGAGAAAGGC GGACAGGTAT CCGGTAAGCG GCAGGGTCGG
1621 AACAGGAGAG CGCACGAGGG AGCTTCCAGG GGGAAACGCC TGGTATCTTT ATAGTCCTGT
1681 CGGGTTTCGC CACCTCTGAC TTGAGCGTCG ATTTTTGTGA TGCTCGTCAG GGGGGCGGAG
1741 CCTATGGAAA AACGCCAGCA ACGCGGCCTT TTTACGGTTC CTGGCCTTTT GCTGGCCTTT
1801 TGCTCACATG TTCTTTCCTG CGTTATCCCC TGATTCTGTG GATAACCGTA TTACCGCCTT
1861 TGAGTGAGCT GATACCGCTC GCCGCAGCCG AACGACCGAG CGCAGCGAGT CAGTGAGCGA
1921 GGAAGCGGAA GAGCGCCCAA TACGCAAACC GCCTCTCCCC GCGCGTTGGC CGATTCATTA
1981 ATGCAGCTGG CACGACAGGT TTCCCGACTG GAAAGCGGGC AGTGAGCGCA ACGCAATTAA
2041 TGTGAGTTAG CTCACTCATT AGGCACCCCA GGCTTTACAC TTTATGCTTC CGGCTCGTAT
2101 GTTGTGTGGA ATTGTGAGCG GATAACAATT TCACACAGGA AACAGCTATG ACCATGATTA
2161 CGCCAAGCTC GAAATTAACC CTCACTAAAG GGAACAAAAG CTGGCTAGAA CTAGTGTCGA
2221 CATGCCCGCC GTGACCGTCG AGAACCCGCT GACGCTGCCC CGCGTATCCG CACCCGCCGA
2281 CGCCGTCGCA CGTCCCGTGC TCACCGTGAC CACCGCGCCC AGCGGTTTCG AGGGCGAGGG
2341 CTTCCCGGTG CGCCGCGCGT TCGCCGGGAT CAACTACCGC CACCTCGACC CGTTCATCAT
2401 GATGGACCAG ATGGGTGAGG TGGAGTACGC GCCCGGGGAG CCCAAGGGCA CGCCCTGGCA
2461 CCCGCACCGC GGCTTCGAGA CCGTGACCTA CATCGTCGAC ACTAGTGgat catcaacaag
2521 tttGTACAAA AAAGCAGGCT GGTACCGGGC CCCCCGCTAG CGTCGAGTTC AACTCGACCA
2581 TAATAATTGA TCCGTCAATC CATATTGGTC CGCAATAATG AAAGTTGCAA GAGTACGACG
2641 GTATGAAAAG AGGTTCAGTA AGTTGTAAAC TAATAGTTTC TTCCCAACGT TCAAATGCTG
2701 GCAAATCTTT TCGCGGGCCG CACTTCGTCG ATCGCTAGTC TTAATGATAA TTTCTGAGAA
2761 AAAGGTGCTA CTGCATCTAC TATATTCTAC TGGATATAAA TGAAATAACA ACGTGAGACT
2821 CACCTACAAC ATGTAATTTA TTGATGGTTT AGTTTAACCA ACCTATGAAA TAATTTGATA
2881 TAGAAATTTG TAGTCGTTTT TCTATGAAGT AAAATTCTAA AATCAAACAT TAAACTGTTT
2941 TGTAGTACCC GGACTCATGG TATGGCTTCT ATTAGCCGTA AACAAAGATT TACAATTGAC
3001 TAAGGTTAGG TCCGACACTG TAGGAGCCAG CGCGTCCTTT CAATACATCA ACGGACCATC
3061 TCGTGTTGTT AAATACTTAT TATTATTATG GTTTGCTAAT TGATATGTTC CAAGACCGAT
3121 TTGGATTTCG AAATAAGTAT TCTCTGATTC ATTTTGGGAG CCGGTCTCGT GATACAGTCG
3181 TCAGCCCGTA CGACTTAACT ACATTCTCGT CATGGGTTCA AGCCCCAGAT GGACCGTGCC
3241 GCCATACGTA GAGTCAGTCC TATCCTGTTA TGGGGGGTAA TACATAAGAC ACTGAAAGCC
3301 AACCCCACAA GTGGTACAGA CAAGCCTTGa CCGACAATTG TTGTTGAGCC AAACAGAAGA
3361 AGAATCCATT TCGGGAAATG ATTTTATCAT TCAATCAAAC CAGTCAATCA TAAACATCAT
3421 AGTTTTAAAT ACTCAAAACT AGTTGAGATC TTTAAAACAC ATTATTTTAG TTTAATTAAA
3481 TGATCTGTTA GCTAGAAGGT AGATACGATA TTTTAGACAT TTCGTAATAG ATCGCAAATC
3541 TCTATTATGT TGGTAATTCA CTTCGTAAAA CTCTTAGGCA AAACTCTTAT TAGTAAACAA
3601 AATACTAATC AAACACTGAT AAACTAACGC GATTTATACA TTGGACAAAG AAGAGGCTGA
3661 TTTTAAAAAT ACTCGCTTTA AAATTTGCTT CATTCATCAA TGTATTGTAA AGCACATAAA
3721 GAACACAATC ATTGACTGAA AACAATTCCA CGTCTCAGCC AACTTCCAGG ATCAATGAAA
3781 TAGCAAGTTC CAAGTTCCAT TTCATTGATT ATGGTAACTA CTGATTATTT TCAATAACAA
3841 ATACTTCGAA GACTGCACAA TTCAAAAGTA TGCCAGAAAG AAAGGATTAC TATCAATTGT
3901 GGGTTAATCA AACTAAGACA GGTGGCAAAA ATGGAACCAT TGATTAAGGC AGCCACTGAC
3961 CGATTTCATT TAAAAAACAC ACTCTTGGAA GTTTCCACAC AATCTCACCT TTTGCCAATT
4021 TTAGCAAAGA CGTTGTGCTG CACTGATAAG AATCGAACTG TAAACATGTG GGCAGTAAAA
4081 ATTATTTCAT CGTTCAACAC GGCGGTCATT ACACTATTCG AAGCAGCTGA AAAGATTTGA
4141 TGATAGCAGG ACCGTGAGAT CAGCAAATTT GAGGTATAAA AGATGATCCT GCGACCACCA
4201 GAAGGCACAT TCGAGCTTTG GAGTGCATTC AAAGCATCCG GGCAACTGCG AACAACCGAA
4261 CCatCGATCT CGAGCACCCA ACTTTTCTAT ACAAAGTTGG TACCGGGCCC CCCGCTAGCG
4321 TCGACGGTAT CGATAAGCTT GATATCGAAT TCGTTAACAG ATCCACCGGT CGCCACCATG
4381 GTGAGCAAGG GCGAGGAGCT GTTCACCGGG GTGGTGCCCA TCCTGGTCGA GCTGGACGGC
4441 GACGTAAACG GCCACAAGTT CAGCGTGTCC GGCGAGGGCG AGGGCGATGC CACCTACGGC
4501 AAGCTGACCC TGAAGTTCAT CTGCACCACC GGCAAGCTGC CCGTGCCCTG GCCCACCCTC
4561 GTGACCACCC TGACCTACGG CGTGCAGTGC TTCAGCCGCT ACCCCGACCA CATGAAGCAG
4621 CACGACTTCT TCAAGTCCGC CATGCCCGAA GGCTACGTCC AGGAGCGCAC CATCTTCTTC
4681 AAGGACGACG GCAACTACAA GACCCGCGCC GAGGTGAAGT TCGAGGGCGA CACCCTGGTG
4741 AACCGCATCG AGCTGAAGGG CATCGACTTC AAGGAGGACG GCAACATCCT GGGGCACAAG
4801 CTGGAGTACA ACTACAACAG CCACAACGTC TATATCATGG CCGacAAGCA GAAGAACGGC
4861 ATCAAGGTGA ACTTCAAGAT CCGCCACAAC ATCGAGGaCG GCAGCGTGCA GCTCGCCGAC
4921 CACTACCAGC AGAACACCCC CATCGGCGac GGCCCCGTGC TGCTGCCCGa CAACCACTAC
4981 CTGAGCACCC AGTCCGCCCT GAGCAAagaC CCCAACGAga agCGCGATCA CATGGTCCTG
5041 CTGgaGTTCG TGACCGCCGC CGGgatCacT CTCGGCATGG acGAgcTGTa caAGTaAAgC
5101 gGCCgcGGcT cGAGGGtACC TctAGaGCGG CCGCCACCGC GGTGGAGCTC GAGACAACTT
5161 TGTATAATAA AGTTGGTACC GGGCCCCCCG CTAGCGTCGA CGGTATCGAT AAGCTTGATA
5221 TCGAATTCTC TAGATCATAA TCAGCCATAC CACATTTGTA GAGGTTTTAC TTGCTTTAAA
5281 AAACCTCCCA CACCTCCCCC TGAACCTGAA ACATAAAATG AATGCAATTG TTGTTGTTAA
5341 CTTGTTTATT GCAGCTTATA ATGGTTACAA ATAAAGCAAT AGCATCACAA ATTTCACAAA
5401 TAAAGCATTT TTCTTCACTG CATTCTAGTT GTGGTTTGTC CAAACTCATC AATGTATCAA
5461 GGGCGAATTC CTGCAGCCCA CTTCCGGTAT CTCGCGTTTG TTTGATCGCA CGGTTCCCAC
5521 AATGGTTAAT TCGAGCTCGC CCGGGGATCT AATTCAATTA GAGACTAATT CAATTAGAGC
5581 TAATTCAATT AGGATCCAAG CTTATCGATT TCGAACCCTC GACCGCCGGA GTATAAATAG
5641 AGGCGCTTCG TCTACGGAGC GACAATTCAA TTCAAACAAG CAAAGTGAAC ACGTCGCTAA
5701 GCGAAAGCTA AGCAAATAAA CAAGCGCAGC TGAACAAGCT AAACAATCGG GGTACCGCTA
5761 GAGTCGACGG TACCGCGGGC CCGGGATCCA CCGGTCGCCA CCATGGTGCG CTCCTCCAAG
5821 AACGTCATCA AGGAGTTCAT GCGCTTCAAG GTGCGCATGG AGGGCACCGT GAACGGCCAC
5881 GAGTTCGAGA TCGAGGGCGA GGGCGAGGGC CGCCCCTACG AGGGCCACAA CACCGTGAAG
5941 CTGAAGGTGA CCAAGGGCGG CCCCCTGCCC TTCGCCTGGG ACATCCTGTC CCCCCAGTTC
6001 CAGTACGGCT CCAAGGTGTA CGTGAAGCAC CCCGCCGACA TCCCCGACTA CAAGAAGCTG
6061 TCCTTCCCCG AGGGCTTCAA GTGGGAGCGC GTGATGAACT TCGAGGACGG CGGCGTGGTG
6121 ACCGTGACCC AGGACTCCTC CCTGCAGGAC GGCTGCTTCA TCTACAAGGT GAAGTTCATC
6181 GGCGTGAACT TCCCCTCCGA CGGCCCCGTA ATGCAGAAGA AGACCATGGG CTGGGAGGCC
```

```
     6241 TCCACCGAGC GCCTGTACCC CCGCGACGGC GTGCTGAAGG GCGAGATCCA CAAGGCCCTG
     6301 AAGCTGAAGG ACGGCGGCCA CTACCTGGTG GAGTTCAAGT CCATCTACAT GGCCAAGAAG
     6361 CCCGTGCAGC TGCCCGGCTA CTACTACGTG GACTCCAAGC TGGACATCAC CTCCCACAAC
     6421 GAGGACTACA CCATCGTGGA CCAGTACGAG CGCACCGAGG GCCGCCACCA CCTGTTCCTG
     6481 TAGCGGCCGC GACTCTAGAT CATAATCAGC CATACCACAT TTGTAGAGGT TTTACTTGCT
     6541 TTAAAAAACC TCCCACACCT CCCCCTGAAC CTGAAACATA AAATGAATGC AATTGTTGTT
     6601 GTTAACTTGT TTATTGCAGC TTATAATGGT TACAAATAAA GCAATAGCAT CACAAATTTC
     6661 ACAAATAAAG CATTTTTCTT CACTGCATTC TAGTTGTGGT TTGTCCAAAC TCATCAATGT
     6721 ATCTTAAAGC TTATCGATAC GCGTACGGCG CGCCTAGAGC GGCCGCCACC GCGGTGGAGC
     6781 TCGAGTACCC AGCTTTcttg tacaaagtgg ttgatCGGTA CGTACCCAAT TCGCCCTATA
     6841 GTGAGTCGTA TTACAATTCA CTGGCCGTCG TTTTACAACG TCGTGACTGG GAAAACCCTG
     6901 GCGTTACCCA ACTTAATCGC CTTGCAGCAC ATCCCCCTTT CGCCAGCTGG CGTAATAGCG
     6961 AAGAGGCCCG CACCGATCGC CCTTCCCAAC AGTTGCGCAG CCTGAATGGC GAATGGAAAT
     7021 TGTAAGCGTT AATATTTTGT TAAAATTCGC GTTAAATTTT TGTTAAATCA GCTCATTTTT
     7081 TAACCAATAG GCCGAAATCG GCAAAATCCC TTATAAATCA AAAGAATAGA CCGAGATAGG
     7141 GTTGAGTGTT GTTCCAGTTT GGAACAAGAG TCCACTATTA AAGAACGTGG ACTCCAACGT
     7201 CAAAGGGCGA AAAACCGTCT ATCAGGGCGA TGGCCCACTA CGTGAACCAT CACCCTAATC
     7261 AAGTTTTTTG GGGTCGAGGT GCCGTAAAGC ACTAAATCGG AACCCTAAAG GGAGCCCCCG
     7321 ATTTAGAGCT TGACGGGGAA AGCCGGCGAA CGTGGCGAGA AAGGAAGGGA AGAAAGCGAA
     7381 AGGAGCGGGC GCTAGGGCGC TGGCAAGTGT AGCGGTCACG CTGCGCGTAA CCACCACACC
     7441 CGCCGCGCTT AATGCGCCGC TACAGGGCGC GTCAG
//
```

pDSAT-LpRFP: Plasmid used to generate *Lp*-tdTomato line

```
LOCUS       pDSAT                   7466 bp    DNA     circular
FEATURES             Location/Qualifiers
     misc_feature    complement(5727..5745)
                     /note="M13R"
     misc_feature    4291..4340
                     /note="3x Pax6 binding sites"
     misc_feature    4380..4387
                     /note="TATA"
     misc_feature    271..286
                     /note="M13F"
     misc_feature    3714..3905
                     /note="SV40 term"
     misc_feature    3941..4217
                     /note="attB"
                     /note="SV40 term"
     misc_feature    4551..5267
                     /note="mTurquoise2"
     misc_feature    468..2098
                     /note="LpPromoter"
     misc_feature    2117..3707
                     /note="tdTomato"
     misc_feature    3509..3682
                     /note="ADDED seq"
     source          1..7466
                     /dnas_title="pDSAT Lp_Tomat"
ORIGIN
        1 CTTTCCTGCG TTATCCCCTG ATTCTGTGGA TAACCGTATT ACCGCCTTTG AGTGAGCTGA
       61 TACCGCTCGC CGCAGCCGAA CGACCGAGCG CAGCGAGTCA GTGAGCGAGG AAGCGGAAGA
      121 GCGCCCAATA CGCAAACCGC CTCTCCCCGC GCGTTGGCCG ATTCATTAAT GCAGCTGGCA
      181 CGACAGGTTT CCCGACTGGA AAGCGGGCAG TGAGCGCAAC GCAATTAATA CGCGTACCGC
      241 TAGCATGGAT GTTTTCCCAG TCACGACGTT GTAAAACGAC GGCCAGTCTT AAGCTCGGGC
      301 CCCTACAGGT CACTAATACC ATCTAAGTAG TTGATTCATA GTGACTGGAT ATGTTGTGTT
      361 TTACAGTATT ATGTAGTCTG TTTTTTATGC AAAATCTAAT TTAATATATT GATATTTATA
      421 TCATTTTACG TTTCTCGTTC AACTTTTCTA TACAAAGTTg gtACcggATC CAgcTTGCGG
      481 GGAAGACACA TTCGAGATAC GCTAAGTGAT TGAGCGATTA CGATCTAGCA AAACATACGT
      541 TCAGCTGTGA GAATAATCAT CCATCTTCCT GCAATGAGCA GTTCATTCCC GATTGAGGGA
      601 TTTTATTCCC CGGGGGCCTT TTCAAACGGC TTAATATAAG CAATTAATAG TATTTTTTCT
      661 TTCAGGTTAG TTTACTGTAA TGGTGTAATT GTCATCTTAC ACCTCCGTCT GATAAGAGAT
      721 TACGAAGCTC AGTATGATGA AATAAATAAG ATAAATTTAT TTAAAAAAGA ACAATTGCTA
      781 TGAGAGTGAA ATACAACAGT GGCGTTCACA ATATTCGAAA AACAATAAAA TTAAAAAAAA
      841 AACAAGAAAA CATTCACAAA CATATCAATC TGCTTTCATC GACACCGAAC TGCTAGCCTC
      901 CCCAGTCTAA CCGCGGTGGG GACGTTTAAT TGCCTTTGTT CTCGCACCCG GTCAAACATA
      961 CACTTCGGAC CTTGCTCCGA ACCCCACTGT GATCCCTAGC TCGTCATCAT CATTGCCGGC
     1021 ATCATGCTAA GCGTGCATTA TTTTCACAAC TTAGCGTAAT GCTAGCGTGC GCTAGCAACA
     1081 AACTCGCCCG CAGACTCGTC ACAGCACCGG TACGATCGAT CGTTTACCGT TCCCTTTCCC
     1141 GATCGGGTTG GCTGCGATAT CCGTGTCCGG GTAGAAAACT TCCCCTTTTA CACACACACA
     1201 CTCACATACA CACAGAGCTG AATAGCAACT TACCTTATCT GTTCGTCATC GCTCGGGCGG
     1261 ATCTGGACGA ATCTTCGCAC CGATAACCAT GTGGATCTAC GACCTCCGCT TGGCTGTCTC
     1321 TCTGCTCATG TGTATGTCTG TGTGTGTGTG TATGTGAGCT TCTTCCCTCA AATCCCTCGA
     1381 TCTCGCTGTG SCAACAATCA AACGTGCAAG TGCAAACATT GCACCCCATT GATTATACAC
     1441 CCAACACCAA CCAATTCCCC TTGCGGAGGC ATCTCTGTGC TCGGCAGCAT GTTTACCGCA
     1501 GATCTACAGA GAACTTCAAT TGAGGTCCTT TCCACCCCCA GCCCTCAACC GGCAATCCGG
     1561 CAGCCACTGG ATCATCAGCG AAAGAGAGAG AGAGCAGAGC AGAACAGAGG TGACCAACTG
     1621 TGGTATCGCT TCCCGCGCGC CGGTGTGTTG GTGTCCATTT CGGTGATCGC GATCCCGGCC
```

```
1681 GCTTCCAGCA CCGTCCACCG ATCAGTCACA AAAACGCTCT CCAAACCCCT TATCAGCACC
1741 GTTCGCTGAT GTGAACCCCC GTTCAAACCC CAAATGCAGT GTTTGTATTG CTGTGTGTAT
1801 GTGTACGTGC GTGTGTGTGG AAATTCTATA AAAGTAGGCA CCCGTGGCCG GGATCCGTTA
1861 TTCCCGTCCT GAGGCCCGCC CGGGATCGCT GGTGACGACA GACGAGCCGC TGTGTGACGT
1921 ACGTAGTGCC CGATCGGTAA AGAGTGAACC GTCTTCTCTG CAGTGTAGGA GAGAACGGTT
1981 TCATCTTTTT CGCCCACACC CCCCCGGTTA CATTCCATGT TGAACTGTAA GGTCTAGTGA
2041 ACATTTCCTG AGTGTGGAAA GTGTGGTTTA GTGCGTGAGA GTGCACGGAC ACGACACTAC
2101 CACCATGGTG AGCAAGGGCG AGGAGGTCAT CAAAGAGTTC ATGCGCTTCA AGGTGCGCAT
2161 GGAGGGCTCC ATGAACGGCC ACGAGTTCGA GATCGAGGGC GAGGGCGAGG GCCGCCCCTA
2221 CGAGGGCACC CAGACCGCCA AGCTGAAGGT GACCAAGGGC GGCCCCCTGC CCTTCGCCTG
2281 GGACATCCTG TCCCCCCAGT TCATGTACGG CTCCAAGGCG TACGTGAAGC ACCCCGCCGA
2341 CATCCCCGAT TACAAGAAGC TGTCCTTCCC CGAGGGCTTC AAGTGGGAGC GCGTGATGAA
2401 CTTCGAGGAC GGCGGTCTGG TGACCGTGAC CCAGGACTCC TCCCTGCAGG ACGGCACGCT
2461 GATCTACAAG GTGAAGATGC GCGGCACCAA CTTCCCCCCC GACGGCCCCG TAATGCAGAA
2521 GAAGACCATG GGCTGGGAGG CCTCCACCGA GCGCCTGTAC CCCCGCGACG GCGTGCTGAA
2581 GGGCGAGATC CACCAGGCCC TGAAGCTGAA GGACGGCGGC CACTACCTGG TGGAGTTCAA
2641 GACCATCTAC ATGGCCAAGA AGCCCGTGCA ACTGCCCGGC TACTACTACG TGGACACCAA
2701 GCTGGACATC ACCTCCCACA ACGAGGACTA CACCATCGTG GAACAGTACG AGCGCTCCGA
2761 GGGCCGCCAC CACCTGTTCC TGGGGCATGG CACCGGCAGC ACCGGCAGCG GCAGCTCCGG
2821 CACCGCCTCC TCCGAGGACA ACAACATGGC CGTCATCAAA GAGTTCATGC GCTTCAAGGT
2881 GCGCATGGAG GGCTCCATGA ACGGCCACGA GTTCGAGATC GAGGGCGAGG GCGAGGGCCG
2941 CCCCTACGAG GGCACCCAGA CCGCCAAGCT GAAGGTGACC AAGGGCGGCC CCCTGCCCTT
3001 CGCCTGGGAC ATCCTGTCCC CCCAGTTCAT GTACGGCTCC AAGGCGTACG TGAAGCACCC
3061 CGCCGACATC CCCGATTACA AGAAGCTGTC CTTCCCCGAG GGCTTCAAGT GGGAGCGCGT
3121 GATGAACTTC GAGGACGGCG GTCTGGTGAC CGTGACCCAG GACTCCTCCC TGCAGGACGG
3181 CACGCTGATC TACAAGGTGA AGATGCGCGG CACCAACTTC CCCCCCGACG GCCCCGTAAT
3241 GCAGAAGAAG ACCATGGGCT GGGAGGCCTC CACCGAGCGC CTGTACCCCC GCGACGGCGT
3301 GCTGAAGGGC GAGATCCACC AGGCCCTGAA GCTGAAGGAC GGCGGCCACT ACCTGGTGGA
3361 GTTCAAGACC ATCTACATGG CCAAGAAGCC CGTGCAACTG CCCGGCTACT ACTACGTGGA
3421 CACCAAGCTG GACATCACCT CCCACAACGA GGACTACACC ATCGTGGAAC AGTACGAGCG
3481 CTCCGAGGGC CGCCACCACC TGTTCCTGGG GCATGGCACC GGCAGCACCG GCAGCGGCAG
3541 CTCCGGCACC GCCTCCTCCG AGGACAACAA CATGGCCGTC ATCAAAGAGT TCATGCGCTT
3601 CAAGGTGCGC ATGGAGGGCT CCATGAACGG CCACGAGTTC GAGATCGAGG GCGAGGGCGA
3661 GGGCCGCCAC CACCTGTTCC TGTACGGCAT GGACGAGCTG TACAAGTAAc ttCTAGACAT
3721 AATCAGCCAT ACCACATTTG TAGAGGTTTT ACTTGCTTTA AAAAACCTCC CACACCTCCC
3781 CCTGAACCTG AAACATAAAA TGAATGCAAT TGTTGTTGTT AACTTGTTTA TTGCAGCTTA
3841 TAATGGTTAC AAATAAAGCA ATAGCATCAC AAATTTCACA AATAAAGCAT TTTTCTTCAC
3901 TGCATTCTAG TTGTGGTTTG TCCAAACTCA TCAATGTATC tcgaCGATGT AGGTCACaGT
3961 CTCGAAGCCG CGGTGCGGGT GCCAGGGCGT GCCCTTGGGC TCCCCGGGCG CGTACTCCAC
4021 CTCACCCATC TGGTCCATCA TGATGAACGG GTCGAGGTGG CGGTAGTTGA TCCCGGCGAA
4081 CGCGCGGCGC ACCGGGAAGC CCTCGCCCTC GAAACCGCTG GGCGCGGTGG TCACGGTGAG
4141 CACGGGACGT GCGACGGCGT CGGCGGGTGC GGATACGCGG GGCAGCGTCA GCGGGTTCTC
4201 GACGGTCACG GCGGGCAatt CCTGCAGACT TCCGGTATCT CGCGTTTGTT TGATCGCACG
4261 GTTCCCACAA TGGTTAATTC GAGCTCGCCC GGGGATCTAA TTCAATTAGA GACTAATTCA
4321 ATTAGAGCTA ATTCAATTAG GATCCAAGCT TATCGATTTC GAACCCTCGA CCGCCGGAGT
4381 ATAAATAGAG GCGCTTCGTC TACGGAGCGA CAATTCAATT CAAACAAGCA AAGTGAACAC
4441 GTCGCTAAGC GAAAGCTAAG CAAATAAACA AGCGCAGCTG AACAAGCTAA ACAATCGGGG
4501 TACCGCTAGA GTCGACGGTA CCGCGGGCCC GGGATCCACC GGTCGCCACc atggtgagca
4561 agggcgagga gctgttcacc ggggtggtgc ccatcctggt cgagctggac ggcgacgtaa
4621 acggccacaa gttcagcgtg tccggcgagg gcgagggcga tgccacctac GGCAAGCTGA
4681 CCCTGAAGTT Catctgcacc accggcaagc tgcccgtgcc ctggcccacc ctcgtgacca
4741 ccctgtcctg gggcgtgcag tgcttcgccc gctaccccga ccacatgaag cagcacgact
4801 tcttcaagtc cgccatgccc gaaggctacg tccaggagcg caccatcttc ttcaaggacg
4861 acggcaacta caagacccgc gccgaggtga agttcgaggg cgacaccctg gtgaaccgca
4921 tcgagctgaa gggcatcgac ttcaaggagg acggcaacat cctggggcac aagctggagt
4981 acaactacTt Tagcgacaac gtctatatca ccgccgacaa gcagaagaac ggcatcaagg
5041 ccaacttcaa gatccgccac aacatcgagg acggcggcgt gcagctcgcc gaccactacc
5101 agcagaacac ccccatcggc gacggccccg tgctgctgcc cgacaaccac tacctgagca
5161 cccagtccaa gctgagcaaa gaccccaacg agaagcgcga tcacatggtc ctgctggagt
5221 tcgtgaccgc cgccgggatc actctcggca tggacgagct gtacaagtcc ggaTGAtaga
5281 tctgacgggt gatcaAATCA GCCATACCAC ATTTGTAGAG GTTTTACTTG CTTTAAAAAA
5341 CCTCCCACAC CTCCCCCTGA ACCTGAAACA TAAAATGAAT GCAATTGTTG TTGTTAACTT
5401 GTTTATTGCA GCTTATAATG GTTACAAATA AAGCAATAGC ATCACAAATT TCACAAATAA
5461 AGCATTTTTT TCACTGCATT CTAGTTGTGG TTTGTCCAAA CTCATCAATG TATCTTAAAG
5521 CTTATCGATA CGCGTACGGC GCGCCTAGAG CGGCCGCCAC CGCGGTGGAG CTCGAGTACC
5581 CAGCTTTCTT GTACAAAGTT GGCATTATAA GAAAGCATTG CTTATCAATT TGTTGCAACG
5641 AACAGGTCAC TATCAGTCAA AATAAAATCA TTATTTGCCA TCCAGCTGCA GGGCGGCCGC
5701 GATATCCCCT ATAGTGAGTC GTATTACATG GTCATAGCTG TTTCCTGGCA GCTCTGGCCC
5761 GTGTCTCAAA ATCTCTGATG TTACATTGCA CAAGATAAAA ATATATCATC ATGAACAATA
5821 AAACTGTCTG CTTACATAAA CAGTAATACA AGGGGTGTTA TGAGCCATAT TCAACGGGAA
5881 ACGTCGAGGC CGCGATTAAA TTCCAACATG GATGCTGATT TATATGGGTA TAAATGGGCT
5941 CGCGATAATG TCGGGCAATC AGGTGCGACA ATCTATCGCT TGTATGGGAA GCCCGATGCG
6001 CCAGAGTTGT TTCTGAAACA TGGCAAAGGT AGCGTTGCCA ATGATGTTAC AGATGAGATG
6061 GTCAGACTAA ACTGGCTGAC GGAATTTATG CCTCTTCCGA CCATCAAGCA TTTTATCCGT
6121 ACTCCTGATG ATGCATGGTT ACTCACCACT GCGATCCCCG GAAAAACAGC ATTCCAGGTA
6181 TTAGAAGAAT ATCCTGATTC AGGTGAAAAT ATTGTTGATG CGCTGGCAGT GTTCCTGCGC
6241 CGGTTGCATT CGATTCCTGT TTGTAATTGT CCTTTTAACA GCGATCGCGT ATTTCGTCTC
6301 GCTCAGGCGC AATCACGAAT GAATAACGGT TTGGTTGATG CGAGTGATTT TGATGACGAG
6361 CGTAATGGCT GGCCTGTTGA ACAAGTCTGG AAAGAAATGC ATAAACTTTT GCCATTCTCA
6421 CCGGATTCAG TCGTCACTCA TGGTGATTTC TCACTTGATA ACCTTATTTT TGACGAGGGG
6481 AAATTAATAG GTTGTATTGA TGTTGGACGA GTCGGAATCG CAGACCGATA CCAGGATCTT
```

```
     6541 GCCATCCTAT GGAACTGCCT CGGTGAGTTT TCTCCTTCAT TACAGAAACG GCTTTTTCAA
     6601 AAATATGGTA TTGATAATCC TGATATGAAT AAATTGCAGT TTCATTTGAT GCTCGATGAG
     6661 TTTTTCTAAT CAGAATTGGT TAATTGGTTG TAACACTGGC AGAGCATTAC GCTGACTTGA
     6721 CGGGACGGCG CAAGCTCATG ACCAAAATCC CTTAACGTGA GTTACGCGTC GTTCCACTGA
     6781 GCGTCAGACC CCGTAGAAAA GATCAAAGGA TCTTCTTGAG ATCCTTTTTT TCTGCGCGTA
     6841 ATCTGCTGCT TGCAAACAAA AAAACCACCG CTACCAGCGG TGGTTTGTTT GCCGGATCAA
     6901 GAGCTACCAA CTCTTTTTCC GAAGGTAACT GGCTTCAGCA GAGCGCAGAT ACCAAATACT
     6961 GTTCTTCTAG TGTAGCCGTA GTTAGGCCAC CACTTCAAGA ACTCTGTAGC ACCGCCTACA
     7021 TACCTCGCTC TGCTAATCCT GTTACCAGTG GCTGCTGCCA GTGGCGATAA GTCGTGTCTT
     7081 ACCGGGTTGG ACTCAAGACG ATAGTTACCG GATAAGGCGC AGCGGTCGGG CTGAACGGGG
     7141 GGTTCGTGCA CACAGCCCAG CTTGGAGCGA ACGACCTACA CCGAACTGAG ATACCTACAG
     7201 CGTGAGCTAT GAGAAAGCGC CACGCTTCCC GAAGGGAGAA AGGCGGACAG GTATCCGGTA
     7261 AGCGGCAGGG TCGGAACAGG AGAGCGCACG AGGGAGCTTC CAGGGGGAAA CGCCTGGTAT
     7321 CTTTATAGTC CTGTCGGGTT TCGCCACCTC TGACTTGAGC GTCGATTTTT GTGATGCTCG
     7381 TCAGGGGGGC GGAGCCTATG GAAAAACGCC AGCAACGCGG CCTTTTTACG GTTCCTGGCC
     7441 TTTTGCTGGC CTTTTGCTCA CATGTT
//
```

pAttbRfB2-actGFP: Plasmid used to generate *act5c*-GFP line

```
LOCUS       attBRfB2act_GFP        8432 bp    DNA     circular
FEATURES             Location/Qualifiers
     misc_feature    complement(7791..7811)
                     /note="T7"
     misc_feature    complement(2216..2500)
                     /note="attB"
     misc_feature    2173..2192
                     /note="T3"
     misc_feature    2138..2156
                     /note="M13R"
     misc_feature    6180..6379
                     /note="sv40 terminator"
     misc_feature    6500..6549
                     /note="3xP3 binding sites"
     misc_feature    6759..7684
                     /note="DsRed"
     misc_feature    6589..6596
                     /note="TATA"
     misc_feature    7747..7766
                     /note="attB2"
     misc_feature    2516..2537
                     /note="attB1"
     misc_feature    complement(5304..6083)
                     /note="GFP"
     misc_feature    2613..5181
                     /note="actin5C promoter"
     misc_feature    complement(5236..5257)
                     /note="attB4"
     misc_feature    6111..6132
                     /note="attB3"
     source          1..8432
                     /dnas_title="attBRfB2act_GFP_Sv40dsRED"
ORIGIN
        1 GTGGCACTTT TCGGGGAAAT GTGCGCGGAA CCCCTATTTG TTTATTTTTC TAAATACATT
       61 CAAATATGTA TCCGCTCATG AGACAATAAC CCTGATAAAT GCTTCAATAA TATTGAAAAA
      121 GGAAGAGTAT GAGTATTCAA CATTTCCGTG TCGCCCTTAT TCCCTTTTTT GCGGCATTTT
      181 GCCTTCCTGT TTTTGCTCAC CCAGAAACGC TGGTGAAAGT AAAAGATGCT GAAGATCAGT
      241 TGGGTGCACG AGTGGGTTAC ATCGAACTGG ATCTCAACAG CGGTAAGATC CTTGAGAGTT
      301 TTCGCCCCGA AGAACGTTTT CCAATGATGA GCACTTTTAA AGTTCTGCTA TGTGGCGCGG
      361 TATTATCCCG TATTGACGCC GGGCAAGAGC AACTCGGTCG CCGCATACAC TATTCTCAGA
      421 ATGACTTGGT TGAGTACTCA CCAGTCACAG AAAAGCATCT TACGGATGGC ATGACAGTAA
      481 GAGAATTATG CAGTGCTGCC ATAACCATGA GTGATAACAC TGCGGCCAAC TTACTTCTGA
      541 CAACGATCGG AGGACCGAAG GAGCTAACCG CTTTTTTGCA CAACATGGGG GNATCATGTA
      601 ACTCGCCTTN GATCGTTGGG AACCGGAGCT GAATGAAGCC ATACCAAACG ACGAGCGTGA
      661 CACCACGATG CCTGTAGCAA TGGCAACAAC GTTGCGCAAA CTATTAACTG GCGAACTACT
      721 TACTCTAGCT TCCCGGCAAC AATTAATAGA CTGGATGGAG GCGGATAAAG TTGCAGGACC
      781 ACTTCTGCGC TCGGCCCTTC CGGCTGGCTG GTTTATTGCT GATAAATCTG GAGCCGGTGA
      841 GCGTGGGTCT CGCGGTATCA TTGCAGCACT GGGGCCAGAT GGTAAGCCCT CCCGTATCGT
      901 AGTTATCTAC ACGACGGGGA GTCAGGCAAC TATGGATGAA CGAAATAGAC AGATCGCTGA
      961 GATAGGTGCC TCACTGATTA AGCATTGGTA ACTGTCAGAC CAAGTTTACT CATATATACT
     1021 TTAGATTGAT TTAAAACTTC ATTTTTAATT TAAAAGGATC TAGGTGAAGA TCCTTTTTGA
     1081 TAATCTCATG ACCAAAATCC CTTAACGTGA GTTTTCGTTC CACTGAGCGT CAGACCCCGT
     1141 AGAAAAGATC AAAGGATCTT CTTGAGATCC TTTTTTTCTG CGCGTAATCT GCTGCTTGCA
     1201 AACAAAAAAA CCACCGCTAC CAGCGGTGGT TTGTTTGCCG GATCAAGAGC TACCAACTCT
     1261 TTTTCCGAAG GTAACTGGCT TCAGCAGAGC GCAGATACCA AATACTGTCC TTCTAGTGTA
     1321 GCCGTAGTTA GGCCACCACT TCAAGAACTC TGTAGCACCG CCTACATACC TCGCTCTGCT
     1381 AATCCTGTTA CCAGTGGCTG CTGCCAGTGG CGATAAGTCG TGTCTTACCG GGTTGGACTC
     1441 AAGACGATAG TTACCGGATA AGGCGCAGCG GTCGGGCTGA ACGGGGGGTT CGTGCACACA
     1501 GCCCAGCTTG GAGCGAACGA CCTACACCGA ACTGAGATAC CTACAGCGTG AGCTATGAGA
```

```
1561 AAGCGCCACG CTTCCCGAAG GGAGAAAGGC GGACAGGTAT CCGGTAAGCG GCAGGGTCGG
1621 AACAGGAGAG CGCACGAGGG AGCTTCCAGG GGGAAACGCC TGGTATCTTT ATAGTCCTGT
1681 CGGGTTTCGC CACCTCTGAC TTGAGCGTCG ATTTTTGTGA TGCTCGTCAG GGGGGCGGAG
1741 CCTATGGAAA AACGCCAGCA ACGCGGCCTT TTTACGGTTC CTGGCCTTTT GCTGGCCTTT
1801 TGCTCACATG TTCTTTCCTG CGTTATCCCC TGATTCTGTG GATAACCGTA TTACCGCCTT
1861 TGAGTGAGCT GATACCGCTC GCCGCAGCCG AACGACCGAG CGCAGCGAGT CAGTGAGCGA
1921 GGAAGCGGAA GAGCGCCCAA TACGCAAACC GCCTCTCCCC GCGCGTTGGC CGATTCATTA
1981 ATGCAGCTGG CACGACAGGT TTCCCGACTG GAAAGCGGGC AGTGAGCGCA ACGCAATTAA
2041 TGTGAGTTAG CTCACTCATT AGGCACCCCA GGCTTTACAC TTTATGCTTC CGGCTCGTAT
2101 GTTGTGTGGA ATTGTGAGCG GATAACAATT TCACACAGGA AACAGCTATG ACCATGATTA
2161 CGCCAAGCTC GAAATTAACC CTCACTAAAG GGAACAAAAG CTGGCTAGAA CTAGTGTCGA
2221 CATGCCCGCC GTGACCGTCG AGAACCCGCT GACGCTGCCC CGCGTATCCG CACCCGCCGA
2281 CGCCGTCGCA CGTCCCGTGC TCACCGTGAC CACCGCGCCC AGCGGTTTCG AGGGCGAGGG
2341 CTTCCCGGTG CGCCGCGCGT TCGCCGGGAT CAACTACCGC CACCTCGACC CGTTCATCAT
2401 GATGGACCAG ATGGGTGAGG TGGAGTACGC GCCCGGGGAG CCCAAGGGCA CGCCCTGGCA
2461 CCCGCACCGC GGCTTCGAGA CCGTGACCTA CATCGTCGAC ACTAGTGgat catcaacaag
2521 tttGTACAAA AAAGCAGGCT GGTACCGGGC CCCCCGCTAG CGTCGACGGT ATCGATAAGC
2581 TTGATATCGA ATTCCTGCAG CCCGCGGCCG CAGCATGCAA TTCTATATTC TAAAAACACA
2641 AATGATACTT CTAAAAAAAA ATCATGAATG GCATCAACTC TGAATCAAAT CTTTGCAGAT
2701 GCACCTACTT CTCATTTCCA CTGTCACATC ATTTTTCCAG ATCTCGCTGC CTGTTATGTG
2761 GCCCACAAAC CAAGACACGT TTTATGGCCA TTAAAGCTGG CTGATCGTCG CCAAACACCA
2821 AATACATAAT GAATATGTAC ACATTCGAGA AAGAAGCGAT CAAAGAAGCG TCTTCGGGCG
2881 GAGTAGGAGA ATGCGGAGGA GAAGGAGAAC GAGCTGATCT AGTATCTCTC CACAATCCAA
2941 TGCCAACTGA CCAACTGGCC ATATTCGGAG CAATTTGAAG CCAATTTCCA TCGCCTGGCG
3001 ATCGCTCCAT TCTTGGCTAT ATGTTTTTCA CCGTTACCCG GGGCCATTTT CAAAGACTCG
3061 TCGGCAAGAT AAGATTGTGT CACTCGCTGT CTCTCTTCAT TTGTCGAAGA ATGCTGAGGA
3121 ATTTCGCGAT GACGTCGGCG AGTATTTTGA AGAATGAGAA TAATTTGTAT TTATACGAAA
3181 ATCAGTTAGT GGAATTTTCT ACAAAAACAT GTTATCTATA GATAATTTTG TTGCAAAATA
3241 TGTTGACTAT GACAAAGATT GTATGTATAT ACCTTTAATG TATTCTCATT TTCTTATGTA
3301 TTTATAATGG CAATGATGAT ACTGATGATA TTTTAAGATG ATGCCAGACC AAAAGGCTTG
3361 AATTTCTGCG TCTTTTGCCG AACGCAGTGC ATGTGCAATT GTTGTTTTTT GGAATATTCA
3421 ATTTTCGGAC TGTCCGCTTT GATTTCAGTT TCTTGGCTTA TTCAAAAAGC AAAGTAAAGC
3481 CAAAAAAGCG AGATGGCAAT ACCAAATGCG GCAAAACGGT AGTGGAAGGA AAGGGGTGCG
3541 GGGCAGCGGA AGGAAGGGTG GGGCGGGGCG TGGCGGGGTC TGTGGCTGGG CGCGACGTCA
3601 CCGACGTTGG AGCCACTCCT TTGACCATGT GTGCGTGTGT GTATTATTCG TGTCTCGCCA
3661 CTCGCCGGTT GTTTTTTTCT TTTTATGCTG CGCTCTCTCT AGCGCCATCT CGCTTACGCA
3721 TGCTCAACGC ACCGCATGTT GCCGTTTCCT TTTATGCGTC ATTTTGGCTC GAAATAGGCA
3781 ATTATTTAAA CAAAGATTAG TCAACGAAAA CGCTAAAATA AATAAGTCTA CAATATGGTT
3841 ACTTATTGCC ATGTGTGTGC AGCCAACGAT AGCAACAAAA GCAACAACAC AGGTGGCTTT
3901 CCCTCTTTCA CTTTTTGTTT GCAAGCCGCG TGCGAGCAAG ACGGCACGAC CGGCAAACGC
3961 AATTACGCTG ACAAAGAGCA GACGAAGTTT TGGCGAAAAA CATCAAGGCG CCTGATACGA
4021 ATGCATTTGC AATAACAATT GCGATATTTA ATATTGTTTA TGAAGCTGTT TGACTTCAAA
4081 ACACACAAAA AAAAAAATAA AACAAATTAT TTGAAAGAGA ATTAGGAATC GGACGCTTAT
4141 CGTTAGGGTA ACAACAAGAA ATGCTTACTG AGTCACAGCC TCTGGAAAAC TGCCGCAAGC
4201 CAGAGAGAGA GAGAAAAAGA GGGAGAGCAG CTTAGACCGC ATGTGCTTGT GTGTGAGGCG
4261 TCTCTCTCTT CGTCTCTGTT GCGCAAACGC ATAGACTGCA CTGAGAAAAT CGATTACCTA
4321 TTTTTTATGA ATGAATATTT GCACTATTAC TATTCAAAAC TATTAAGATA GCAATCACAT
4381 TCAATAGCCA AATACTATAC CACCTGAGCG ATGCAACGAA ATGATCAATT TGAGCAAAAA
4441 TGCTGCATAT TTAGGACGGC ATCATTATAG AAATGCTTCT TGCTGTGTAC TTTTCTCTCG
4501 TCTGGCAGCT GTTTCGCCGT TATTGTTAAA ACCGGCTTAA GTTAGGTGTG TTTTCTACGA
4561 CTAGTGAATG CCCTACTAGA AGATGTGTGT TGCACAAAAT GTCCCTGGAA TAACCAATTT
4621 GAAGTGCAGA TAGCAGTAAA CGTAAGCTAA TATGAATATT ATTTAACTGT AATGTTTTAA
4681 TATCGCTGGA CATTACTAAT AAACCCACTA TAAACACATG TACATATGTA TGTTTTGGCA
4741 TACAATGAGT AGTTGGGGAA AAAATGTGTA AAAGCACCGT GACCATCACA GCATAAAGAT
4801 AACCAGCTGA AGTATCGAAT ATGAGTAACC CCCAAATTGA ATCACATGCC GCAACTGATA
4861 GGACCCATGG AAGTACACTC TTCATGGCGA TATACAAGAC ACACACAAGC ACGAACACCC
4921 AGTTGCGGAG GAAATTCTCC GTAAATGAAA ACCCAATCGG CGAACAATTC ATACCCATAT
4981 ATGGTAAAAG TTTTGAACGC GACTTGAGAG CGGAGAGCAT TGCGGCTGAT AAGGTTTTAG
5041 CGCTAAGCGG GCTTTATAAA ACGGGCTGCG GGACCAGTTT TCATATCACT ACCGTTTGAG
5101 TTCTTGTGCT GTGTGGATAC TCCTCCCGAC ACAAAGCCGC TCCATCAGCC AGCAGTCGTC
5161 TAATCCAGAG ACCCCGGATC CCCGGGCTGC AGGAATTCGA TATCAAGCTT ATCGATACCG
5221 TCGACCTCGA GCACCCAACT TTTCTATACA AAGTTGGTAC CGGGCCCCCC GCTAGCGTCG
5281 ACGGTATCGA TAAGCTTGAT ATCGAATTCG TTAACAGATC CACCGGTCGC CACCATGGTG
5341 AGCAAGGGCG AGGAGCTGTT CACCGGGGTG GTGCCCATCC TGGTCGAGCT GGACGGCGAC
5401 GTAAACGGCC ACAAGTTCAG CGTGTCCGGC GAGGGCGAGG GCGATGCCAC CTACGGCAAG
5461 CTGACCCTGA AGTTCATCTG CACCACCGGC AAGCTGCCCG TGCCCTGGCC CACCCTCGTG
5521 ACCACCCTGA CCTACGGCGT GCAGTGCTTC AGCCGCTACC CCGACCACAT GAAGCAGCAC
5581 GACTTCTTCA AGTCCGCCAT GCCCGAAGGC TACGTCCAGG AGCGCACCAT CTTCTTCAAG
5641 GACGACGGCA ACTACAAGAC CCGCGCCGAG GTGAAGTTCG AGGGCGACAC CCTGGTGAAC
5701 CGCATCGAGC TGAAGGGCAT CGACTTCAAG GAGGACGGCA ACATCCTGGG GCACAAGCTG
5761 GAGTACAACT ACAACAGCCA CAACGTCTAT ATCATGGCCG acAAGCAGAA GAACGGCATC
5821 AAGGTGAACT TCAAGATCCG CCACAACATC GAGGaCGGCA GCGTGCAGCT CGCCGACCAC
5881 TACCAGCAGA ACACCCCCAT CGGCGacGGC CCCGTGCTGC TGCCCGaCAA CCACTACCTG
5941 AGCACCCAGT CCGCCCTGAG CAAagaCCCC AACGAgaagC GCGATCACAT GGTCCTGCTG
6001 gaGTTCGTGA CCGCCGCCGG gatCacTCTC GGCATGGacG AgcTGTacaA GTaAAgCgGC
6061 CgcGGcTcGA GGGtACCTct AGaGCGGCCG CCACCGCGGT GGAGCTCGAG ACAACTTTGT
6121 ATAATAAAGT TGGTACCGGG CCCCCCGCTA GCGTCGACGG TATCGATAAG CTTGATATCG
6181 AATTCTCTAG ATCATAATCA GCCATACCAC ATTTGTAGAG GTTTTACTTG CTTTAAAAAA
6241 CCTCCCACAC CTCCCCCTGA ACCTGAAACA TAAAATGAAT GCAATTGTTG TTGTTAACTT
6301 GTTTATTGCA GCTTATAATG GTTACAAATA AAGCAATAGC ATCACAAATT TCACAAATAA
6361 AGCATTTTTC TTCACTGCAT TCTAGTTGTG GTTTGTCCAA ACTCATCAAT GTATCAAGGG
```

```
     6421 CGAATTCCTG CAGCCCACTT CCGGTATCTC GCGTTTGTTT GATCGCACGG TTCCCACAAT
     6481 GGTTAATTCG AGCTCGCCCG GGGATCTAAT TCAATTAGAG ACTAATTCAA TTAGAGCTAA
     6541 TTCAATTAGG ATCCAAGCTT ATCGATTTCG AACCCTCGAC CGCCGGAGTA TAAATAGAGG
     6601 CGCTTCGTCT ACGGAGCGAC AATTCAATTC AAACAAGCAA AGTGAACACG TCGCTAAGCG
     6661 AAAGCTAAGC AAATAAACAA GCGCAGCTGA ACAAGCTAAA CAATCGGGGT ACCGCTAGAG
     6721 TCGACGGTAC CGCGGGCCCG GGATCCACCG GTCGCCACCA TGGTGCGCTC CTCCAAGAAC
     6781 GTCATCAAGG AGTTCATGCG CTTCAAGGTG CGCATGGAGG GCACCGTGAA CGGCCACGAG
     6841 TTCGAGATCG AGGGCGAGGG CGAGGGCCGC CCCTACGAGG GCCACAACAC CGTGAAGCTG
     6901 AAGGTGACCA AGGGCGGCCC CCTGCCCTTC GCCTGGGACA TCCTGTCCCC CCAGTTCCAG
     6961 TACGGCTCCA AGGTGTACGT GAAGCACCCC GCCGACATCC CCGACTACAA GAAGCTGTCC
     7021 TTCCCCGAGG GCTTCAAGTG GGAGCGCGTG ATGAACTTCG AGGACGGCGG CGTGGTGACC
     7081 GTGACCCAGG ACTCCTCCCT GCAGGACGGC TGCTTCATCT ACAAGGTGAA GTTCATCGGC
     7141 GTGAACTTCC CCTCCGACGG CCCCGTAATG CAGAAGAAGA CCATGGGCTG GGAGGCCTCC
     7201 ACCGAGCGCC TGTACCCCCG CGACGGCGTG CTGAAGGGCG AGATCCACAA GGCCCTGAAG
     7261 CTGAAGGACG GCGGCCACTA CCTGGTGGAG TTCAAGTCCA TCTACATGGC CAAGAAGCCC
     7321 GTGCAGCTGC CCGGCTACTA CTACGTGGAC TCCAAGCTGG ACATCACCTC CCACAACGAG
     7381 GACTACACCA TCGTGGACCA GTACGAGCGC ACCGAGGGCC GCCACCACCT GTTCCTGTAG
     7441 CGGCCGCGAC TCTAGATCAT AATCAGCCAT ACCACATTTG TAGAGGTTTT ACTTGCTTTA
     7501 AAAAACCTCC CACACCTCCC CCTGAACCTG AAACATAAAA TGAATGCAAT TGTTGTTGTT
     7561 AACTTGTTTA TTGCAGCTTA TAATGGTTAC AAATAAAGCA ATAGCATCAC AAATTTCACA
     7621 AATAAAGCAT TTTTCTTCAC TGCATTCTAG TTGTGGTTTG TCCAAACTCA TCAATGTATC
     7681 TTAAAGCTTA TCGATACGCG TACGGCGCGC CTAGAGCGGC CGCCACCGCG GTGGAGCTCG
     7741 AGTACCCAGC TTTcttgtac aaagtggttg atCGGTACGT ACCCAATTCG CCCTATAGTG
     7801 AGTCGTATTA CAATTCACTG GCCGTCGTTT TACAACGTCG TGACTGGGAA AACCCTGGCG
     7861 TTACCCAACT TAATCGCCTT GCAGCACATC CCCCTTTCGC CAGCTGGCGT AATAGCGAAG
     7921 AGGCCCGCAC CGATCGCCCT TCCCAACAGT TGCGCAGCCT GAATGGCGAA TGGAAATTGT
     7981 AAGCGTTAAT ATTTTGTTAA AATTCGCGTT AAATTTTTGT TAAATCAGCT CATTTTTTAA
     8041 CCAATAGGCC GAAATCGGCA AAATCCCTTA TAAATCAAAA GAATAGACCG AGATAGGGTT
     8101 GAGTGTTGTT CCAGTTTGGA ACAAGAGTCC ACTATTAAAG AACGTGGACT CCAACGTCAA
     8161 AGGGCGAAAA ACCGTCTATC AGGGCGATGG CCCACTACGT GAACCATCAC CCTAATCAAG
     8221 TTTTTTGGGG TCGAGGTGCC GTAAAGCACT AAATCGGAAC CCTAAAGGGA GCCCCCGATT
     8281 TAGAGCTTGA CGGGGAAAGC CGGCGAACGT GGCGAGAAAG GAAGGGAAGA AAGCGAAAGG
     8341 AGCGGGCGCT AGGGCGCTGG CAAGTGTAGC GGTCACGCTG CGCGTAACCA CCACACCCGC
     8401 CGCGCTTAAT GCGCCGCTAC AGGGCGCGTC AG
//
```

pAttbRfB2-G12GFP: Plasmid used to generate *G12*-GFP line

```
LOCUS       attBrf2G12_GFP_S        7012 bp    DNA     circular
FEATURES             Location/Qualifiers
     misc_feature    complement(6371..6391)
                     /note="T7"
     misc_feature    complement(2216..2500)
                     /note="attB"
     misc_feature    2173..2192
                     /note="T3"
     misc_feature    2138..2156
                     /note="M13R"
     gene            4760..4959
                     /note="sv40 terminator"
     misc_feature    5080..5129
                     /note="3xP3 binding sites"
     misc_feature    4691..4712
                     /note="attB3"
     misc_feature    5339..6264
                     /note="DsRed"
     misc_feature    5169..5176
                     /note="TATA"
     misc_feature    6327..6346
                     /note="attB2"
     misc_feature    2516..2537
                     /note="attB1"
     misc_feature    2541..3811
                     /note="G12 promoter"
     misc_feature    3723..3805
                     /note="G12 Exon1"
     misc_feature    complement(3884..4663)
                     /note="GFP"
     misc_feature    complement(3816..3837)
                     /note="attB4"
     source          1..7012
                     /dnas_title="attBrf2G12_GFP_Sv40dsRED"
ORIGIN
        1 GTGGCACTTT TCGGGGAAAT GTGCGCGGAA CCCCTATTTG TTTATTTTTC TAAATACATT
       61 CAAATATGTA TCCGCTCATG AGACAATAAC CCTGATAAAT GCTTCAATAA TATTGAAAAA
      121 GGAAGAGTAT GAGTATTCAA CATTTCCGTG TCGCCCTTAT TCCCTTTTTT GCGGCATTTT
      181 GCCTTCCTGT TTTTGCTCAC CCAGAAACGC TGGTGAAAGT AAAAGATGCT GAAGATCAGT
      241 TGGGTGCACG AGTGGGTTAC ATCGAACTGG ATCTCAACAG CGGTAAGATC CTTGAGAGTT
      301 TTCGCCCCGA AGAACGTTTT CCAATGATGA GCACTTTTAA AGTTCTGCTA TGTGGCGCGG
```

361 TATTATCCCG TATTGACGCC GGGCAAGAGC AACTCGGTCG CCGCATACAC TATTCTCAGA
421 ATGACTTGGT TGAGTACTCA CCAGTCACAG AAAAGCATCT TACGGATGGC ATGACAGTAA
481 GAGAATTATG CAGTGCTGCC ATAACCATGA GTGATAACAC TGCGGCCAAC TTACTTCTGA
541 CAACGATCGG AGGACCGAAG GAGCTAACCG CTTTTTTGCA CAACATGGGG GNATCATGTA
601 ACTCGCCTTN GATCGTTGGG AACCGGAGCT GAATGAAGCC ATACCAAACG ACGAGCGTGA
661 CACCACGATG CCTGTAGCAA TGGCAACAAC GTTGCGCAAA CTATTAACTG GCGAACTACT
721 TACTCTAGCT TCCCGGCAAC AATTAATAGA CTGGATGGAG GCGGATAAAG TTGCAGGACC
781 ACTTCTGCGC TCGGCCCTTC CGGCTGGCTG GTTTATTGCT GATAAATCTG GAGCCGGTGA
841 GCGTGGGTCT CGCGGTATCA TTGCAGCACT GGGGCCAGAT GGTAAGCCCT CCCGTATCGT
901 AGTTATCTAC ACGACGGGGA GTCAGGCAAC TATGGATGAA CGAAATAGAC AGATCGCTGA
961 GATAGGTGCC TCACTGATTA AGCATTGGTA ACTGTCAGAC CAAGTTTACT CATATATACT
1021 TTAGATTGAT TTAAAACTTC ATTTTTAATT TAAAAGGATC TAGGTGAAGA TCCTTTTTGA
1081 TAATCTCATG ACCAAAATCC CTTAACGTGA GTTTTCGTTC CACTGAGCGT CAGACCCCGT
1141 AGAAAAGATC AAAGGATCTT CTTGAGATCC TTTTTTTCTG CGCGTAATCT GCTGCTTGCA
1201 AACAAAAAAA CCACCGCTAC CAGCGGTGGT TTGTTTGCCG GATCAAGAGC TACCAACTCT
1261 TTTTCCGAAG GTAACTGGCT TCAGCAGAGC GCAGATACCA AATACTGTCC TTCTAGTGTA
1321 GCCGTAGTTA GGCCACCACT TCAAGAACTC TGTAGCACCG CCTACATACC TCGCTCTGCT
1381 AATCCTGTTA CCAGTGGCTG CTGCCAGTGG CGATAAGTCG TGTCTTACCG GGTTGGACTC
1441 AAGACGATAG TTACCGGATA AGGCGCAGCG GTCGGGCTGA ACGGGGGGTT CGTGCACACA
1501 GCCCAGCTTG GAGCGAACGA CCTACACCGA ACTGAGATAC CTACAGCGTG AGCTATGAGA
1561 AAGCGCCACG CTTCCCGAAG GGAGAAAGGC GGACAGGTAT CCGGTAAGCG GCAGGGTCGG
1621 AACAGGAGAG CGCACGAGGG AGCTTCCAGG GGGAAACGCC TGGTATCTTT ATAGTCCTGT
1681 CGGGTTTCGC CACCTCTGAC TTGAGCGTCG ATTTTTGTGA TGCTCGTCAG GGGGGCGGAG
1741 CCTATGGAAA AACGCCAGCA ACGCGGCCTT TTTACGGTTC CTGGCCTTTT GCTGGCCTTT
1801 TGCTCACATG TTCTTTCCTG CGTTATCCCC TGATTCTGTG GATAACCGTA TTACCGCCTT
1861 TGAGTGAGCT GATACCGCTC GCCGCAGCCG AACGACCGAG CGCAGCGAGT CAGTGAGCGA
1921 GGAAGCGGAA GAGCGCCCAA TACGCAAACC GCCTCTCCCC GCGCGTTGGC CGATTCATTA
1981 ATGCAGCTGG CACGACAGGT TTCCCGACTG GAAAGCGGGC AGTGAGCGCA ACGCAATTAA
2041 TGTGAGTTAG CTCACTCATT AGGCACCCCA GGCTTTACAC TTTATGCTTC CGGCTCGTAT
2101 GTTGTGTGGA ATTGTGAGCG GATAACAATT TCACACAGGA AACAGCTATG ACCATGATTA
2161 CGCCAAGCTC GAAATTAACC CTCACTAAAG GGAACAAAAG CTGGCTAGAA CTAGTGTCGA
2221 CATGCCCGCC GTGACCGTCG AGAACCCGCT GACGCTGCCC CGCGTATCCG CACCCGCCGA
2281 CGCCGTCGCA CGTCCCGTGC TCACCGTGAC CACCGCGCCC AGCGGTTTCG AGGGCGAGGG
2341 CTTCCCGGTG CGCCGCGCGT TCGCCGGGAT CAACTACCGC CACCTCGACC CGTTCATCAT
2401 GATGGACCAG ATGGGTGAGG TGGAGTACGC GCCCGGGGAG CCCAAGGGCA CGCCCTGGCA
2461 CCCGCACCGC GGCTTCGAGA CCGTGACCTA CATCGTCGAC ACTAGTGgat catcaacaag
2521 tttGTACAAA AAAGCAGGCT ggtaccCACA ATACCGGCCC TGAATCTTTA AAAGACCAGG
2581 TAATAATCCT AAACTTAAAC TAGTTTAGCG ATCCAGCAAC TAATTAGGTC CTAGAACTCA
2641 ATCCATTCTA GTCTTGGAAC TCTATCCTAC TCACGTACCA GCCCTGAAAA TGATTAAGAA
2701 ACTGAGCCCA GGTCAGTCCT GGAACCGACC ATGAAACGAT ACCATTCTTT GAAATCCCGG
2761 AAGTCCCAGA AGTACCCATA CCAGTCCTGG AACAGATACT GTACCCATCC TAGGAACTTG
2821 TCCAAATTTG ATAATATTCC CGACACTGAT AGTAAACTCA TATTGGTCTA CGAAATGATC
2881 CCTAAGCCAG CCATACCGAT TCAGGAACTT GTACGGAATC CTCACCGGTT CTCAAACTAA
2941 TTCCTTATCC ATATAGATCC TGGAATTGAT CCATAACCTA TACTGGTCCT GGATTTTTCC
3001 CCATGTCAAA CTGATCCTAG AACTCATTCC TACTTTATTC CAATTCATGG AAAACTGTAC
3061 CATAGACCAA TCTCAGTGAA AGAACGAATC GTGAACCTGT CCCGAAACAA GAAGCCGATA
3121 CCAAACCTCC TGCTCTAATC CGGCAACTGA TCTCCCTACT GGTGAATCAA CCATTCCCGA
3181 CGGTACTTTA GAGGAAAAAC TCAGCGTAGA AATTTAGCTA CGACCAAGTA GCCAGCCACT
3241 AGACTGAAGA TCTCCATCCA GAAACTAAAT CATGTGGAAA TCATTCCATT GCAACGAACT
3301 GTGTCCAAGG TCCAGGTTGG CCTTATCGGT TGCCTTAGAA ACATCTGTTC GCAGAAGAAG
3361 TACTGGATTG AACCGTAAGT WGAGAGTTCC AAGGGAAKAA GTTCCCATCC AAACTAAAAG
3421 GGCGTGATCA GTAATCAATT AAGCACACAA ATTCCATGCT AAATCATCAT TATCGCTAAT
3481 CGGATTATGT TATTATAATA AGTATGGACC ACTTGACCAG TGATGGGTAG TGGTCGGTGG
3541 ACTTCTTGAG AGTGAGTGTG GGACAGACGG GCTGAAAGTC CAGTACCTGC CAACAGATGT
3601 ACACTCGAAA GGCACAGATA ACAGCACTCC ACTCCTTSGA CAGCTGGTTA ATCGACTGTT
3661 GACCGCTCAA GATACTGACA GCAGYGGACG TATAAAAGCT ACTGGCTGGT TGGTAGAGAA
3721 ATCATTCCCA AGCCTACTAC AGCTCAGTAC AGCAGTACCA CAGCATCGRA ACCAGTGCAA
3781 AAGCAACCCA ATCAAATCTA GCATCctcga gCACCCAACT TTTCTATACA AAGTTGGTAC
3841 CGGGCCCCCC GCTAGCGTCG ACGGTATCGA TAAGCTTGAT ATCGAATTCG TTAACAGATC
3901 CACCGGTCGC CACCATGGTG AGCAAGGGCG AGGAGCTGTT CACCGGGGTG GTGCCCATCC
3961 TGGTCGAGCT GGACGGCGAC GTAAACGGCC ACAAGTTCAG CGTGTCCGGC GAGGGCGAGG
4021 GCGATGCCAC CTACGGCAAG CTGACCCTGA AGTTCATCTG CACCACCGGC AAGCTGCCCG
4081 TGCCCTGGCC CACCCTCGTG ACCACCCTGA CCTACGGCGT GCAGTGCTTC AGCCGCTACC
4141 CCGACCACAT GAAGCAGCAC GACTTCTTCA AGTCCGCCAT GCCCGAAGGC TACGTCCAGG
4201 AGCGCACCAT CTTCTTCAAG GACGACGGCA ACTACAAGAC CCGCGCCGAG GTGAAGTTCG
4261 AGGGCGACAC CCTGGTGAAC CGCATCGAGC TGAAGGGCAT CGACTTCAAG GAGGACGGCA
4321 ACATCCTGGG GCACAAGCTG GAGTACAACT ACAACAGCCA CAACGTCTAT ATCATGGCCG
4381 acAAGCAGAA GAACGGCATC AAGGTGAACT TCAAGATCCG CCACAACATC GAGGaCGGCA
4441 GCGTGCAGCT CGCCGACCAC TACCAGCAGA ACACCCCCAT CGGCGacGGC CCCGTGCTGC
4501 TGCCCGaCAA CCACTACCTG AGCACCCAGT CCGCCCTGAG CAAagaCCCC AACGAgaagC
4561 GCGATCACAT GGTCCTGCTG gaGTTCGTGA CCGCCGCCGG gatCacTCTC GGCATGGacG
4621 AgcTGTacaA GTaAAgCgGC CgcGGcTcGA GGGtACCTct AGaGCGGCCG CCACCGCGGT
4681 GGAGCTCGAG ACAACTTTGT ATAATAAAGT TGGTACCGGG CCCCCCGCTA GCGTCGACGG
4741 TATCGATAAG CTTGATATCG AATTCTCTAG ATCATAATCA GCCATACCAC ATTTGTAGAG
4801 GTTTTACTTG CTTTAAAAAA CCTCCCACAC CTCCCCCTGA ACCTGAAACA TAAAATGAAT
4861 GCAATTGTTG TTGTTAACTT GTTTATTGCA GCTTATAATG GTTACAAATA AAGCAATAGC
4921 ATCACAAATT TCACAAATAA AGCATTTTTC TTCACTGCAT TCTAGTTGTG GTTTGTCCAA
4981 ACTCATCAAT GTATCAAGGG CGAATTCCTG CAGCCCACTT CCGGTATCTC GCGTTTGTTT
5041 GATCGCACGG TTCCCACAAT GGTTAATTCG AGCTCGCCCG GGGATCTAAT TCAATTAGAG
5101 ACTAATTCAA TTAGAGCTAA TTCAATTAGG ATCCAAGCTT ATCGATTTCG AACCCTCGAC
5161 CGCCGGAGTA TAAATAGAGG CGCTTCGTCT ACGGAGCGAC AATTCAATTC AAACAAGCAA

```
     5221 AGTGAACACG TCGCTAAGCG AAAGCTAAGC AAATAAACAA GCGCAGCTGA ACAAGCTAAA
     5281 CAATCGGGGT ACCGCTAGAG TCGACGGTAC CGCGGGCCCG GGATCCACCG GTCGCCACCA
     5341 TGGTGCGCTC CTCCAAGAAC GTCATCAAGG AGTTCATGCG CTTCAAGGTG CGCATGGAGG
     5401 GCACCGTGAA CGGCCACGAG TTCGAGATCG AGGGCGAGGG CGAGGGCCGC CCCTACGAGG
     5461 GCCACAACAC CGTGAAGCTG AAGGTGACCA AGGGCGGCCC CCTGCCCTTC GCCTGGGACA
     5521 TCCTGTCCCC CCAGTTCCAG TACGGCTCCA AGGTGTACGT GAAGCACCCC GCCGACATCC
     5581 CCGACTACAA GAAGCTGTCC TTCCCCGAGG GCTTCAAGTG GGAGCGCGTG ATGAACTTCG
     5641 AGGACGGCGG CGTGGTGACC GTGACCCAGG ACTCCTCCCT GCAGGACGGC TGCTTCATCT
     5701 ACAAGGTGAA GTTCATCGGC GTGAACTTCC CCTCCGACGG CCCCGTAATG CAGAAGAAGA
     5761 CCATGGGCTG GGAGGCCTCC ACCGAGCGCC TGTACCCCCG CGACGGCGTG CTGAAGGGCG
     5821 AGATCCACAA GGCCCTGAAG CTGAAGGACG GCGGCCACTA CCTGGTGGAG TTCAAGTCCA
     5881 TCTACATGGC CAAGAAGCCC GTGCAGCTGC CCGGCTACTA CTACGTGGAC TCCAAGCTGG
     5941 ACATCACCTC CCACAACGAG GACTACACCA TCGTGGACCA GTACGAGCGC ACCGAGGGCC
     6001 GCCACCACCT GTTCCTGTAG CGGCCGCGAC TCTAGATCAT AATCAGCCAT ACCACATTTG
     6061 TAGAGGTTTT ACTTGCTTTA AAAAACCTCC CACACCTCCC CCTGAACCTG AAACATAAAA
     6121 TGAATGCAAT TGTTGTTGTT AACTTGTTTA TTGCAGCTTA TAATGGTTAC AAATAAAGCA
     6181 ATAGCATCAC AAATTTCACA AATAAAGCAT TTTTTCACT TGCATTCTAG TTGTGGTTTG
     6241 TCCAAACTCA TCAATGTATC TTAAAGCTTA TCGATACGCG TACGGCGCGC CTAGAGCGGC
     6301 CGCCACCGCG GTGGAGCTCG AGTACCCAGC TTTcttgtac aaagtggttg atCGGTACGT
     6361 ACCCAATTCG CCCTATAGTG AGTCGTATTA CAATTCACTG GCCGTCGTTT TACAACGTCG
     6421 TGACTGGGAA AACCCTGGCG TTACCCAACT TAATCGCCTT GCAGCACATC CCCCTTTCGC
     6481 CAGCTGGCGT AATAGCGAAG AGGCCCGCAC CGATCGCCCT TCCCAACAGT TGCGCAGCCT
     6541 GAATGGCGAA TGGAAATTGT AAGCGTTAAT ATTTTGTTAA AATTCGCGTT AAATTTTTGT
     6601 TAAATCAGCT CATTTTTTAA CCAATAGGCC GAAATCGGCA AAATCCCTTA TAAATCAAAA
     6661 GAATAGACCG AGATAGGGTT GAGTGTTGTT CCAGTTTGGA ACAAGAGTCC ACTATTAAAG
     6721 AACGTGGACT CCAACGTCAA AGGGCGAAAA ACCGTCTATC AGGGCGATGG CCCACTACGT
     6781 GAACCATCAC CCTAATCAAG TTTTTTGGGG TCGAGGTGCC GTAAAGCACT AAATCGGAAC
     6841 CCTAAAGGGA GCCCCCGATT TAGAGCTTGA CGGGGAAAGC CGGCGAACGT GGCGAGAAAG
     6901 GAAGGGAAGA AAGCGAAAGG AGCGGGCGCT AGGGCGCTGG CAAGTGTAGC GGTCACGCTG
     6961 CGCGTAACCA CCACACCCGC CGCGCTTAAT GCGCCGCTAC AGGGCGCGTC AG
//
```

pAttbRfB2-vas2RFP: Plasmid used to generate *vasa*-tdTomato line

```
LOCUS       pattB-vas2Tomato        8675 bp    DNA     circular
FEATURES             Location/Qualifiers
     misc_feature    complement(8034..8054)
                     /note="T7"
     misc_feature    complement(2216..2500)
                     /note="attB"
     misc_feature    2173..2192
                     /note="T3"
     misc_feature    2138..2156
                     /note="M13R"
     misc_feature    2575..4865
                     /dnas_title="5'vasa 5' region"
                     /vntifkey="21"
                     /label=5'vasa 5' region
     misc_feature    4931..6359
                     /note="tdTomato"
     misc_feature    6494..6693
                     /note="sv40 terminator"
     gene            7002..7718
                     /note="EYFP (Clontech)"
     misc_feature    6831..6838
                     /note="TATA"
     misc_feature    7725..7979
                     /note="SV40 term sequences"
     source          1..8675
                     /dnas_title="pattB-vas2Tomato-YFP"
ORIGIN
        1 GTGGCACTTT TCGGGGAAAT GTGCGCGGAA CCCCTATTTG TTTATTTTTC TAAATACATT
       61 CAAATATGTA TCCGCTCATG AGACAATAAC CCTGATAAAT GCTTCAATAA TATTGAAAAA
      121 GGAAGAGTAT GAGTATTCAA CATTTCCGTG TCGCCCTTAT TCCCTTTTTT GCGGCATTTT
      181 GCCTTCCTGT TTTTGCTCAC CCAGAAACGC TGGTGAAAGT AAAAGATGCT GAAGATCAGT
      241 TGGGTGCACG AGTGGGTTAC ATCGAACTGG ATCTCAACAG CGGTAAGATC CTTGAGAGTT
      301 TTCGCCCCGA AGAACGTTTT CCAATGATGA GCACTTTTAA AGTTCTGCTA TGTGGCGCGG
      361 TATTATCCCG TATTGACGCC GGGCAAGAGC AACTCGGTCG CCGCATACAC TATTCTCAGA
      421 ATGACTTGGT TGAGTACTCA CCAGTCACAG AAAAGCATCT TACGGATGGC ATGACAGTAA
      481 GAGAATTATG CAGTGCTGCC ATAACCATGA GTGATAACAC TGCGGCCAAC TTACTTCTGA
      541 CAACGATCGG AGGACCGAAG GAGCTAACCG CTTTTTTGCA CAACATGGGG GNATCATGTA
      601 ACTCGCCTTN GATCGTTGGG AACCGGAGCT GAATGAAGCC ATACCAAACG ACGAGCGTGA
      661 CACCACGATG CCTGTAGCAA TGGCAACAAC GTTGCGCAAA CTATTAACTG GCGAACTACT
      721 TACTCTAGCT TCCCGGCAAC AATTAATAGA CTGGATGGAG GCGGATAAAG TTGCAGGACC
      781 ACTTCTGCGC TCGGCCCTTC CGGCTGGCTG GTTTATTGCT GATAAATCTG GAGCCGGTGA
      841 GCGTGGGTCT CGCGGTATCA TTGCAGCACT GGGGCCAGAT GGTAAGCCCT CCCGTATCGT
      901 AGTTATCTAC ACGACGGGGA GTCAGGCAAC TATGGATGAA CGAAATAGAC AGATCGCTGA
      961 GATAGGTGCC TCACTGATTA AGCATTGGTA ACTGTCAGAC CAAGTTTACT CATATATACT
```

```
1021 TTAGATTGAT TTAAAACTTC ATTTTTAATT TAAAAGGATC TAGGTGAAGA TCCTTTTTGA
1081 TAATCTCATG ACCAAAATCC CTTAACGTGA GTTTTCGTTC CACTGAGCGT CAGACCCCGT
1141 AGAAAAGATC AAAGGATCTT CTTGAGATCC TTTTTTTCTG CGCGTAATCT GCTGCTTGCA
1201 AACAAAAAAA CCACCGCTAC CAGCGGTGGT TTGTTTGCCG GATCAAGAGC TACCAACTCT
1261 TTTTCCGAAG GTAACTGGCT TCAGCAGAGC GCAGATACCA AATACTGTCC TTCTAGTGTA
1321 GCCGTAGTTA GGCCACCACT TCAAGAACTC TGTAGCACCG CCTACATACC TCGCTCTGCT
1381 AATCCTGTTA CCAGTGGCTG CTGCCAGTGG CGATAAGTCG TGTCTTACCG GGTTGGACTC
1441 AAGACGATAG TTACCGGATA AGGCGCAGCG GTCGGGCTGA ACGGGGGGTT CGTGCACACA
1501 GCCCAGCTTG GAGCGAACGA CCTACACCGA ACTGAGATAC CTACAGCGTG AGCTATGAGA
1561 AAGCGCCACG CTTCCCGAAG GGAGAAAGGC GGACAGGTAT CCGGTAAGCG GCAGGGTCGG
1621 AACAGGAGAG CGCACGAGGG AGCTTCCAGG GGGAAACGCC TGGTATCTTT ATAGTCCTGT
1681 CGGGTTTCGC CACCTCTGAC TTGAGCGTCG ATTTTTGTGA TGCTCGTCAG GGGGGCGGAG
1741 CCTATGGAAA AACGCCAGCA ACGCGGCCTT TTTACGGTTC CTGGCCTTTT GCTGGCCTTT
1801 TGCTCACATG TTCTTTCCTG CGTTATCCCC TGATTCTGTG GATAACCGTA TTACCGCCTT
1861 TGAGTGAGCT GATACCGCTC GCCGCAGCCG AACGACCGAG CGCAGCGAGT CAGTGAGCGA
1921 GGAAGCGGAA GAGCGCCCAA TACGCAAACC GCCTCTCCCC GCGCGTTGGC CGATTCATTA
1981 ATGCAGCTGG CACGACAGGT TTCCCGACTG GAAAGCGGGC AGTGAGCGCA ACGCAATTAA
2041 TGTGAGTTAG CTCACTCATT AGGCACCCCA GGCTTTACAC TTTATGCTTC CGGCTCGTAT
2101 GTTGTGTGGA ATTGTGAGCG GATAACAATT TCACACAGGA AACAGCTATG ACCATGATTA
2161 CGCCAAGCTC GAAATTAACC CTCACTAAAG GGAACAAAAG CTGGCTAGAA CTAGTGTCGA
2221 CATGCCCGCC GTGACCGTCG AGAACCCGCT GACGCTGCCC CGCGTATCCG CACCCGCCGA
2281 CGCCGTCGCA CGTCCCGTGC TCACCGTGAC CACCGCGCCC AGCGGTTTCG AGGGCGAGGG
2341 CTTCCCGGTG CGCCGCGCGT TCGCCGGGAT CAACTACCGC CACCTCGACC CGTTCATCAT
2401 GATGGACCAG ATGGGTGAGG TGGAGTACGC GCCCGGGGAG CCCAAGGGCA CGCCCTGGCA
2461 CCCGCACCGC GGCTTCGAGA CCGTGACCTA CATCGTCGAC ACTAGTGgat catcaacaag
2521 TTTGTACAAA AAAGCAGGCT GGTACCGGGC CCCCCGCTAG CGTCGACGGT ATcgatgtag
2581 aacgcgagca aattcttttc cttccatgac agcagcagct acagtgggaa gccgaacgtc
2641 agacgtgttt gacatgccga actgggcggg aaaattacag cgtgcgcttt gttttcaagc
2701 aaatcacaac tcgctgcaaa caaaaccgtt gagaaattga ttgttttata atttgtattg
2761 tattttattt gttataataa actaaaaaga catacttttt gcatatttta tacataaaaa
2821 catacatgca gcattataaa acacatataa accctccctg tagagtcccg tatcgaaatc
2881 ttccatccta gttgcacagt acgacggacg agtaggccgt gtccgtgcaa attccagctt
2941 ttagcagtct tttgctcgga gcactcgcgg cgagtcggag gtttctgctg aggtgcttag
3001 cgctaaatta gccaattgct tttgcaagtg aaataaccag ccgaatagta cttcaaaact
3061 caggtaagtg aactagtttt atagaacaaa tgtttgtttg ttagaagtta gtgaagtgtt
3121 tgtgaaaaaa atctctcatt tcggcaaaac taacgtaact gatttcaaat tgaattattg
3181 ttttgtgatg ttatattatt tcatccagtt gattagtatt ttcttagtta tgttcaaaat
3241 acagttaaat taaatttcat ttcatttact cataaaataa tctcttggct tatttaattt
3301 ttctcgaatt cgcttgtatt gttcagtagc acgcgccatt cgccctttgt ttcattttgt
3361 acctgctccc actaacacac tggcagtgcg aaacaaaagc cttcgcacgc gttgctggta
3421 ttagagtgtg tgcgtgtgtg tgttgagcgc tctgtcaaaa tcggctgttg ccgccggtac
3481 cgaaattgcc tgttcgcacg ctgttcgtaa acattccgtg gtgtgtatcg tgtgttgtgc
3541 atgttgcgcg cctccccccct tttgatagca ggctgccgtg gctgccgtgg tgtgtggcgc
3601 agttgagttt ttggattaat tttctaagga aatggcacga gaagagcggt ggcagtgtgt
3661 tggtttgctc tgtcccttcc tttctgtgtg aagtgttctt acagcacagc acgtatccac
3721 caccgcacac agagcaggca aggaagtgga agtgaacaag tgtgctgcgc atgcatgtgt
3781 gtgggggggca ttttagctga gatcgtcgtt atttgagaag cggtataggg gccagtcggt
3841 gtcgacgtac ggaagcggtt tagttttaat ccaagcgtat cccgtcgtgg agtggttgtg
3901 tggctctgtg tgctctcata tcagttccag agtgaggtta gtagaatcac agtccttggc
3961 ctttttcgtt acaagatatc cagaaggatg gcgttatttc cacagcttac catggtgctc
4021 ttgtttgctc gaatcagggg agaaaaacag tttcgtgttt catgaaccgc agttggcact
4081 ggagcggatt caaaagtctt cgatatgcaa tagataagag agtcgttggg gcatagttgg
4141 gaagcctttc cgagatgtgg agtttccgag aggagaaatg gtgctttcgt gcacgttccg
4201 ggacagcggg ccccgcgaag agcatctcgt tgtcgttcat ccggcaataa ttgatgcgaa
4261 aagcgcgcgc gccactggct tagcgcagtg tacacagtga tattcaccta cacacacaga
4321 ggcacacgcc ttcacacgcg cgcgtgcttc aaaggctact tcggtggcgg tgtgtgaggt
4381 cgcttgcaat ggacaatgaa aatttcgctg gaaaatacca tcgtctcttt aggttgcaat
4441 gggtgcgggt agagcggtgg tcgtcgatat tggtggtgta gtgtgtgtgt gtgtgtgtgt
4501 gtgtgtgtgt gtgtgtgtgt gtgtgtgtgt gtgtgtgtgt gtgtgtgtgt gtgtgtgtgt
4561 gtgtgtgtgt gtgtgtgcaa cggcaattat tttttgtaat atttcgacca tctttctttc
4621 tctctctcca cgtgctgctg ctgttgctgc tgctgctgca ttgcatgttc cactattcct
4681 ctcggtttgt gcctgcggac gccattgcta gtcgaaagag agtcgccgtt agtcgcgctt
4741 cgagcaacgg acacgttttt tggttgaaac caacagcttt tttcatcttc gggagacaca
4801 cagatctcga atcgtacatt cccataagga gaattgtcat cttccggtga ataaagaaag
4861 gaaacggatc TCGAGCACCC AACTTTTCTA TACAAAGTTG GTACCGGGCC CCCCGCTAGC
4921 GTCGACAACC ATGGTGAGCA AGGGCGAGGA GGTCATCAAA GAGTTCATGC GCTTCAAGGT
4981 GCGCATGGAG GGCTCCATGA ACGGCCACGA GTTCGAGATC GAGGGCGAGG GCGAGGGCCG
5041 CCCCTACGAG GGCACCCAGA CCGCCAAGCT GAAGGTGACC AAGGGCGGCC CCCTGCCCTT
5101 CGCCTGGGAC ATCCTGTCCC CCCAGTTCAT GTACGGCTCC AAGGCGTACG TGAAGCACCC
5161 CGCCGACATC CCCGATTACA AGAAGCTGTC CTTCCCCGAG GGCTTCAAGT GGGAGCGCGT
5221 GATGAACTTC GAGGACGGCG GTCTGGTGAC CGTGACCCAG GACTCCTCCC TGCAGGACGG
5281 CACGCTGATC TACAAGGTGA AGATGCGCGG CACCAACTTC CCCCCCGACG GCCCCGTAAT
5341 GCAGAAGAAG ACCATGGGCT GGGAGGCCTC CACCGAGCGC CTGTACCCCC GCGACGGCGT
5401 GCTGAAGGGC GAGATCCACC AGGCCCTGAA GCTGAAGGAC GGCGGCCACT ACCTGGTGGA
5461 GTTCAAGACC ATCTACATGG CCAAGAAGCC CGTGCAACTG CCCGGCTACT ACTACGTGGA
5521 CACCAAGCTG GACATCACCT CCCACAACGA GGACTACACC ATCGTGGAAC AGTACGAGCG
5581 CTCCGAGGGC CGCCACCACC TGTTCCTGGG GCATGGCACC GGCAGCACCG GCAGCGGCAG
5641 CTCCGGCACC GCCTCCTCCG AGGACAACAA CATGGCCGTC ATCAAAGAGT TCATGCGCTT
5701 CAAGGTGCGC ATGGAGGGCT CCATGAACGG CCACGAGTTC GAGATCGAGG GCGAGGGCGA
5761 GGGCCGCCCC TACGAGGGCA CCCAGACCGC CAAGCTGAAG GTGACCAAGG GCGGCCCCCT
5821 GCCCTTCGCC TGGGACATCC TGTCCCCCCA GTTCATGTAC GGCTCCAAGG CGTACGTGAA
```

```
5881 GCACCCCGCC GACATCCCCG ATTACAAGAA GCTGTCCTTC CCCGAGGGCT TCAAGTGGGA
5941 GCGCGTGATG AACTTCGAGG ACGGCGGTCT GGTGACCGTG ACCCAGGACT CCTCCCTGCA
6001 GGACGGCACG CTGATCTACA AGGTGAAGAT GCGCGGCACC AACTTCCCCC CCGACGGCCC
6061 CGTAATGCAG AAGAAGACCA TGGGCTGGGA GGCCTCCACC GAGCGCCTGT ACCCCCGCGA
6121 CGGCGTGCTG AAGGGCGAGA TCCACCAGGC CCTGAAGCTG AAGGACGGCG GCCACTACCT
6181 GGTGGAGTTC AAGACCATCT ACATGGCCAA GAAGCCCGTG CAACTGCCCG GCTACTACTA
6241 CGTGGACACC AAGCTGGACA TCACCTCCCA CAACGAGGAC TACACCATCG TGGAACAGTA
6301 CGAGCGCTCC GAGGGCCGCC ACCACCTGTT CCTGTACGGC ATGGACGAGC TGTACAAGTA
6361 AGAATTCCTG CAGCCCGGGG GATCCACTAG TTCTAGAGCG GCCGCCACCG CGGTGGAGCT
6421 CGAGACAACT TTGTATAATA AAGTTGGTAC CGGGCCCCCC GCTAGCGTCG ACGGTATCGA
6481 TAAGCTTGAT ATCGAATTCT CTAGATCATA ATCAGCCATA CCACATTTGT AGAGGTTTTA
6541 CTTGCTTTAA AAAACCTCCC ACACCTCCCC CTGAACCTGA AACATAAAAT GAATGCAATT
6601 GTTGTTGTTA ACTTGTTTAT TGCAGCTTAT AATGGTTACA AATAAAGCAA TAGCATCACA
6661 AATTTCACAA ATAAAGCATT TTTCTTCACT GCATTCTAGT TGTGGTTTGT CCAAACTCAT
6721 CAATGTATCA AGGGCGAATT CGGGGATCTA ATTCAATTAG AGACTAATTC AATTAGAGCT
6781 AATTCAATTA GGATCCAAGC TTATCGATTT CGAACCCTCG ACCGCCGGAG TATAAATAGA
6841 GGCGCTTCGT CTACGGAGCG ACAATTCAAT TCAAACAAGC AAAGTGAACA CGTCGCTAAG
6901 CGAAAGCTAA GCAAATAAAC AAGCGCAGCT GAACAAGCTA AACAATCGGG GTACCGCTAG
6961 AGTCGACGGT ACCGCGGGCC CGGGATCCAC CGGTCGCCAC CATGGTGAGC AAGGGCGAGG
7021 AGCTGTTCAC CGGGGTGGTG CCCATCCTGG TCGAGCTGGA CGGCGACGTA AACGGCCACA
7081 AGTTCAGCGT GTCCGGCGAG GGCGAGGGCG ATGCCACCTA CGGCAAGCTG ACCCTGAAGT
7141 TCATCTGCAC CACCGGCAAG CTGCCCGTGC CCTGGCCCAC CCTCGTGACC ACCTTCGGCT
7201 ACGGCCTGCA GTGCTTCGCC CGCTACCCCG ACCACATGAA GCAGCACGAC TTCTTCAAGT
7261 CCGCCATGCC CGAAGGCTAC GTCCAGGAGC GCACCATCTT CTTCAAGGAC GACGGCAACT
7321 ACAAGACCCG CGCCGAGGTG AAGTTCGAGG GCGACACCCT GGTGAACCGC ATCGAGCTGA
7381 AGGGCATCGA CTTCAAGGAG GACGGCAACA TCCTGGGGCA CAAGCTGGAG TACAACTACA
7441 ACAGCCACAA CGTCTATATC ATGGCCGACA AGCAGAAGAA CGGCATCAAG GTGAACTTCA
7501 AGATCCGCCA CAACATCGAG GACGGCAGCG TGCAGCTCGC CGACCACTAC CAGCAGAACA
7561 CCCCCATCGG CGACGGCCCC GTGCTGCTGC CCGACAACCA CTACCTGAGC TACCAGTCCG
7621 CCCTGAGCAA AGACCCCAAC GAGAAGCGCG ATCACATGGT CCTGCTGGAG TTCGTGACCG
7681 CCGCCGGGAT CACTCTCGGC ATGGACGAGC TGTACAAGTA AAGCGGCCGC GACTCTAGAT
7741 CATAATCAGC CATACCACAT TTGTAGAGGT TTTACTTGCT TTAAAAAACC TCCCACACCT
7801 CCCCCTGAAC CTGAAACATA AAATGAATGC AATTGTTGTT GTTAACTTGT TTATTGCAGC
7861 TTATAATGGT TACAAATAAA GCAATAGCAT CACAAATTTC ACAAATAAAG CATTTTTTTC
7921 ACTGCATTCT AGTTGTGGTT TGTCCAAACT CATCAATGTA TCTTAAAGCT TATCGATACc
7981 tcgagTACCC AGCTTTcttg tacaaagtgg ttgatCGGTA CGTACCCAAT TCGCCCTATA
8041 GTGAGTCGTA TTACAATTCA CTGGCCGTCG TTTTACAACG TCGTGACTGG GAAAACCCTG
8101 GCGTTACCCA ACTTAATCGC CTTGCAGCAC ATCCCCCTTT CGCCAGCTGG CGTAATAGCG
8161 AAGAGGCCCG CACCGATCGC CCTTCCCAAC AGTTGCGCAG CCTGAATGGC GAATGGAAAT
8221 TGTAAGCGTT AATATTTTGT TAAAATTCGC GTTAAATTTT TGTTAAATCA GCTCATTTTT
8281 TAACCAATAG GCCGAAATCG GCAAAATCCC TTATAAATCA AAAGAATAGA CCGAGATAGG
8341 GTTGAGTGTT GTTCCAGTTT GGAACAAGAG TCCACTATTA AAGAACGTGG ACTCCAACGT
8401 CAAAGGGCGA AAAACCGTCT ATCAGGGCGA TGGCCCACTA CGTGAACCAT CACCCTAATC
8461 AAGTTTTTTG GGGTCGAGGT GCCGTAAAGC ACTAAATCGG AACCCTAAAG GGAGCCCCCG
8521 ATTTAGAGCT TGACGGGGAA AGCCGGCGAA CGTGGCGAGA AAGGAAGGGA AGAAAGCGAA
8581 AGGAGCGGGC GCTAGGGCGC TGGCAAGTGT AGCGGTCACG CTGCGCGTAA CCACCACACC
8641 CGCCGCGCTT AATGCGCCGC TACAGGGCGC GTCAG
//
```

File S4

perl script analyzing codon usage in an input sequence to evaluate how efficiently it will be expressed in *Anopheles gambiae*. The input codon usage table derived from highly-expressed *A. gambiae* genes is appended below.

```
#!/usr/bin/perl -w


use strict;


##################################################################
########

#Assessment of the codon usage status of a coding sequence

#Input : codon usage table - the threshold frequency - the
heterologous coding sequence from ATG to STOP

#

#Output : results.TXT

#Vittu Ana•Øs 28-02-2013

##################################################################
########



if ($#ARGV != 2){

     print " Syntaxe : codon_usage.pl
input_table_codon_usage.tab    coding_sequence.fa
threshold_frequency_in_%   \n" ;

     exit(-1) ;

}


###################################

##             Codon usage             ##

###################################
```

```
my %codonUsageTab = ();
my ($codon_tab, $codon_usage);
my $threshold = $ARGV[2];

if ($threshold < 1 || $threshold > 100)
{
     print "The frequency threshold must be between 1% and
100%\nPlease, enter a new value\n";
     $threshold = <STDIN>;
}
if ($threshold >= 1 && $threshold <= 100)
{
     $threshold = $threshold / 100;
}

open(TAB, $ARGV[0]) || die ($ARGV[0]."can't be open! Exit\n");
while(<TAB>)
{
     if ($_ =~ /^[DEFGAC\*LMNHIKTWVQPSRY]/)
     {
          next;
     }else
     {
          chomp;
          ($codon_tab, $codon_usage) = split(" ", $_);
          if($codon_usage <= $threshold)
          {
               chop($codon_tab);
               $codonUsageTab{$codon_tab} = $codon_usage;
          }
```

```
	}
}
close(TAB);


###################################
##             Sequence            ##
###################################

my $sequence = "";
my $name_seq;

open(SEQ, $ARGV[1]) || die ($ARGV[1]."can't be open! Exit\n");
while(<SEQ>)
{
	if ($_ =~ /^[atcg]/)
	{
		chomp;
		$sequence .= $_;
	}
	elsif ($_ =~ /^[ATCG]/)
	{
		chomp;
		$sequence .= lc($_);
	}
	else
	{
		$name_seq = $_;
	}
}
```

```
close SEQ;



####################################
##             Count                   ##
####################################

my @seq = ();
my $nb = 0;
my $codon = "";
my %count = ();
my $m;

@seq = split (/ */, $sequence);
for ($m = 0; $m <= $#seq; $m++) {
     $codon .= $seq[$m];
     $nb += 1;
     if ($nb == 3)
     {
          if (exists $count{$codon})
          {
                    $count{$codon} += 1;
          }else
          {
               $count{$codon} = 1;
          }
          $nb = 0;
          $codon = "";
     }
```

```
}

###################################
##             Frequency           ##
###################################

my %freqcodon = ();
my $nbcodon;
my $codonFreq;

$nbcodon = length($sequence) / 3 ;
foreach my $codoncount (keys(%count))
{
     $codonFreq = $count{$codoncount} / $nbcodon;
     $freqcodon{$codoncount} = $codonFreq;
}

###################################
##             Penalty                  ##
###################################

my $penalty;
my $allpenalties = 0;
my %penalties = ();
my $proteinLength;
my $densityOfProblems;

foreach my $codoncount (keys(%count))
{
     foreach my $codontab (keys(%codonUsageTab))
```

```
	{
		if ($codoncount eq $codontab)
		{
			$penalty = $count{$codoncount} / 
$codonUsageTab{$codontab};
			$penalties{$codoncount} = $penalty;
			$allpenalties += $penalty;
		}
	}
}

$proteinLength = length($sequence) / 3 ;
$densityOfProblems = $allpenalties / $proteinLength;
$allpenalties = $allpenalties / 100;
$densityOfProblems = $densityOfProblems * 10;



###################################
##            Output                  ##
###################################

$name_seq =~ s/^.//;
chomp($name_seq);

open(OUT, ">".$name_seq."_codus_results.txt") || die 
($name_seq."_codus_results.txt can't be open! Exit\n");

print OUT "Protein length :\t$proteinLength\n";
print OUT "Sum of all penalties :\t";
printf OUT "%0.2f", $allpenalties;
```

```
print OUT "\nDensity of problems :\t";
printf OUT "%0.2f", $densityOfProblems;


print OUT "\nCodon\tFreq in model\tCount\tFreq in
seq\tPenalty\n";
foreach my $codonpenalty (keys(%penalties))
{
     print OUT "$codonpenalty\t$codonUsageTab{$codonpenalty}\t";
     print OUT "$count{$codonpenalty}\t";
     printf OUT "%0.2f", $freqcodon{$codonpenalty};
     print OUT "\t";
     printf OUT "%0.2f", $penalties{$codonpenalty};
     print OUT "\n";
}

close OUT;
```

Codon usage table:

```
D
gat: 0.36
gac: 0.64
E
gaa: 0.23
gag: 0.77
F
ttt: 0.16
```

ttc: 0.84

G

ggt: 0.27

ggc: 0.45

gga: 0.22

ggg: 0.07

A

gct: 0.19

gcc: 0.45

gca: 0.09

gcg: 0.28

C

tgt: 0.33

tgc: 0.67

*

taa: 0.57

tag: 0.29

tga: 0.14

L

tta: 0.01

ttg: 0.06

ctt: 0.07

ctc: 0.17

cta: 0.03

ctg: 0.66

M

atg: 1.00

N

aat: 0.14

aac: 0.86

H

cat: 0.21

cac: 0.79

I

att: 0.18

atc: 0.79

ata: 0.04

K

aaa: 0.08

aag: 0.92

T

act: 0.06

acc: 0.48

aca: 0.05

acg: 0.42

W

tgg: 1.00

V

gtt: 0.11

gtc: 0.35

gta: 0.04

gtg: 0.51

Q

caa: 0.09

cag: 0.91

P

cct: 0.05

ccc: 0.26

cca: 0.13

ccg: 0.56

S

tct: 0.08

tcc: 0.17

tca: 0.01

tcg: 0.40

agt: 0.05

agc: 0.29

R

cgt: 0.30

cgc: 0.51

cga: 0.05

cgg: 0.10

aga: 0.02

agg: 0.02

Y

tat: 0.11

tac: 0.89

File S5

Annotated DNA sequence of the four plasmids used to generate transgenic *A. gambiae* lines expressing codon-optimized CSP (with and without Fasciclin gpi anchor, with Lipophorin (Lp) or Vitellogenin (Vg) promoter)

pAttBRfB1CFP-Vg-OptCSPΔgpi:

```
LOCUS       pattB-VgP-OptCSP        7510 bp    DNA     circular
FEATURES             Location/Qualifiers
     misc_feature    complement(6869..6889)
                     /note="T7"
     misc_feature    complement(2216..2500)
                     /note="attB"
     misc_feature    2173..2192
                     /note="T3"
     misc_feature    2138..2156
                     /note="M13R"
     misc_feature    2516..2538
                     /note="attB1"
     misc_feature    4311..4385
                     /note="lipophorin signal peptide"
     misc_feature    4303..4310
                     /note="Vg Kozak"
     misc_feature    complement(4280..4299)
                     /note="attB4"
     misc_feature    5247..5476
                     /note="SV40 term"
     misc_feature    5539..5744
                     /note="3xP3-hsp70"
     misc_feature    5481..5502
                     /note="attB3"
     misc_feature    6534..6755
                     /note="SV40 term"
     misc_feature    complement(6824..6845)
                     /note="attB2"
     misc_feature    2564..4265
                     /note="Vitellogenin promoter"
     gene            5797..6513
                     /note="ECFP (Clontech)"
ORIGIN
        1 GTGGCACTTT TCGGGGAAAT GTGCGCGGAA CCCCTATTTG TTTATTTTTC TAAATACATT
       61 CAAATATGTA TCCGCTCATG AGACAATAAC CCTGATAAAT GCTTCAATAA TATTGAAAAA
      121 GGAAGAGTAT GAGTATTCAA CATTTCCGTG TCGCCCTTAT TCCCTTTTTT GCGGCATTTT
      181 GCCTTCCTGT TTTTGCTCAC CCAGAAACGC TGGTGAAAGT AAAAGATGCT GAAGATCAGT
      241 TGGGTGCACG AGTGGGTTAC ATCGAACTGG ATCTCAACAG CGGTAAGATC CTTGAGAGTT
      301 TTCGCCCCGA AGAACGTTTT CCAATGATGA GCACTTTTAA AGTTCTGCTA TGTGGCGCGG
      361 TATTATCCCG TATTGACGCC GGGCAAGAGC AACTCGGTCG CCGCATACAC TATTCTCAGA
      421 ATGACTTGGT TGAGTACTCA CCAGTCACAG AAAAGCATCT TACGGATGGC ATGACAGTAA
      481 GAGAATTATG CAGTGCTGCC ATAACCATGA GTGATAACAC TGCGGCCAAC TTACTTCTGA
      541 CAACGATCGG AGGACCGAAG GAGCTAACCG CTTTTTTGCA CAACATGGGG GNATCATGTA
      601 ACTCGCCTTN GATCGTTGGG AACCGGAGCT GAATGAAGCC ATACCAAACG ACGAGCGTGA
      661 CACCACGATG CCTGTAGCAA TGGCAACAAC GTTGCGCAAA CTATTAACTG GCGAACTACT
      721 TACTCTAGCT TCCCGGCAAC AATTAATAGA CTGGATGGAG GCGGATAAAG TTGCAGGACC
      781 ACTTCTGCGC TCGGCCCTTC CGGCTGGCTG GTTTATTGCT GATAAATCTG GAGCCGGTGA
      841 GCGTGGGTCT CGCGGTATCA TTGCAGCACT GGGGCCAGAT GGTAAGCCCT CCCGTATCGT
      901 AGTTATCTAC ACGACGGGGA GTCAGGCAAC TATGGATGAA CGAAATAGAC AGATCGCTGA
      961 GATAGGTGCC TCACTGATTA AGCATTGGTA ACTGTCAGAC CAAGTTTACT CATATATACT
     1021 TTAGATTGAT TTAAAACTTC ATTTTTAATT TAAAAGGATC TAGGTGAAGA TCCTTTTTGA
     1081 TAATCTCATG ACCAAAATCC CTTAACGTGA GTTTTCGTTC CACTGAGCGT CAGACCCCGT
     1141 AGAAAAGATC AAAGGATCTT CTTGAGATCC TTTTTTTCTG CGCGTAATCT GCTGCTTGCA
     1201 AACAAAAAAA CCACCGCTAC CAGCGGTGGT TTGTTTGCCG GATCAAGAGC TACCAACTCT
     1261 TTTTCCGAAG GTAACTGGCT TCAGCAGAGC GCAGATACCA AATACTGTCC TTCTAGTGTA
     1321 GCCGTAGTTA GGCCACCACT TCAAGAACTC TGTAGCACCG CCTACATACC TCGCTCTGCT
     1381 AATCCTGTTA CCAGTGGCTG CTGCCAGTGG CGATAAGTCG TGTCTTACCG GGTTGGACTC
     1441 AAGACGATAG TTACCGGATA AGGCGCAGCG GTCGGGCTGA ACGGGGGGTT CGTGCACACA
     1501 GCCCAGCTTG GAGCGAACGA CCTACACCGA ACTGAGATAC CTACAGCGTG AGCTATGAGA
     1561 AAGCGCCACG CTTCCCGAAG GGAGAAAGGC GGACAGGTAT CCGGTAAGCG GCAGGGTCGG
     1621 AACAGGAGAG CGCACGAGGG AGCTTCCAGG GGGAAACGCC TGGTATCTTT ATAGTCCTGT
     1681 CGGGTTTCGC CACCTCTGAC TTGAGCGTCG ATTTTTGTGA TGCTCGTCAG GGGGGCGGAG
     1741 CCTATGGAAA AACGCCAGCA ACGCGGCCTT TTTACGGTTC CTGGCCTTTT GCTGGCCTTT
     1801 TGCTCACATG TTCTTTCCTG CGTTATCCCC TGATTCTGTG GATAACCGTA TTACCGCCTT
     1861 TGAGTGAGCT GATACCGCTC GCCGCAGCCG AACGACCGAG CGCAGCGAGT CAGTGAGCGA
     1921 GGAAGCGGAA GAGCGCCCAA TACGCAAACC GCCTCTCCCC GCGCGTTGGC CGATTCATTA
     1981 ATGCAGCTGG CACGACAGGT TTCCCGACTG GAAAGCGGGC AGTGAGCGCA ACGCAATTAA
```

2041 TGTGAGTTAG CTCACTCATT AGGCACCCCA GGCTTTACAC TTTATGCTTC CGGCTCGTAT
2101 GTTGTGTGGA ATTGTGAGCG GATAACAATT TCACACAGGA AACAGCTATG ACCATGATTA
2161 CGCCAAGCTC GAAATTAACC CTCACTAAAG GGAACAAAAG CTGGCTAGAA CTAGTGTCGA
2221 CATGCCCGCC GTGACCGTCG AGAACCCGCT GACGCTGCCC CGCGTATCCG CACCCGCCGA
2281 CGCCGTCGCA CGTCCCGTGC TCACCGTGAC CACCGCGCCC AGCGGTTTCG AGGGCGAGGG
2341 CTTCCCGGTG CGCCGCGCGT TCGCCGGGAT CAACTACCGC CACCTCGACC CGTTCATCAT
2401 GATGGACCAG ATGGGTGAGG TGGAGTACGC GCCCGGGGAG CCCAAGGGCA CGCCCTGGCA
2461 CCCGCACCGC GGCTTCGAGA CCGTGACCTA CATCGTCGAC ACTAGTGgat catcaaacaa
2521 gttGTACAA AAAAGCAGGC TGGTACCGGG CCCCCCGCTA GCGTCGAGTT CAACTCGACC
2581 ATAATAATTG ATCCGTCAAT CCATATTGGT CCGCAATAAT GAAAGTTGCA AGAGTACGAC
2641 GGTATGAAAA GAGGTTCAGT AAGTTGTAAA CTAATAGTTT CTTCCCAACG TTCAAATGCT
2701 GGCAAATCTT TTCGCGGGCC GCACTTCGTC GATCGCTAGT CTTAATGATA ATTTCTGAGA
2761 AAAAGGTGCT ACTGCATCTA CTATATTCTA CTGGATATAA ATGAAATAAC AACGTGAGAC
2821 TCACCTACAA CATGTAATTT ATTGATGGTT TAGTTTAACC AACCTATGAA ATAATTTGAT
2881 ATAGAAATTT GTAGTCGTTT TTCTATGAAG TAAAATTCTA AAATCAAACA TTAAACTGTT
2941 TTGTAGTACC CGGACTCATG GTATGGCTTC TATTAGCCGT AAACAAAGAT TTACAATTGA
3001 CTAAGGTTAG GTCCGACACT GTAGGAGCCA GCGCGTCCTT TCAATACATC AACGGACCAT
3061 CTCGTGTTGT TAAATACTTA TTATTATTAT GGTTTGCTAA TTGATATGTT CCAAGACCGA
3121 TTTGGATTTC GAAATAAGTA TTCTCTGATT CATTTTGGGA GCCGGTCTCG TGATACAGTC
3181 GTCAGCCCGT ACGACTTAAC TACATTCCTCG TCATGGGTTC AAGCCCCAGA TGGACCGTGC
3241 CGCCATACGT AGAGTCAGTC CTATCCTGTT ATGGGGGGTA ATACATAAGA CACTGAAAGC
3301 CAACCCCACA AGTGGTACAG ACAAGCCTTG aCCGACAATT GTTGTTGAGC CAAACAGAAG
3361 AAGAATCCAT TTCGGGAAAT GATTTTATCA TTCAATCAAA CCAGTCAATC ATAAACATCA
3421 TAGTTTTAAA TACTCAAAAC TAGTTGAGAT CTTTAAAACA CATTATTTTA GTTTAATTAA
3481 ATGATCTGTT AGCTAGAAGG TAGATACGAT ATTTAGACA TTTCGTAATA GATCGCAAAT
3541 CTCTATTATG TTGGTAATTC ACTTCGTAAA ACTCTTAGGC AAAACTCTTA TTAGTAAACA
3601 AAATACTAAT CAAACACTGA TAAACTAACG CGATTTATAC ATTGGACAAA GAAGAGGCTG
3661 ATTTTAAAAA TACTCGCTTT AAAATTTGCT TCATTCATCA ATGTATTGTA AAGCACATAA
3721 AGAACACAAT CATTGACTGA AAACAATTCC ACGTCTCAGC CAACTTCCAG GATCAATGAA
3781 ATAGCAAGTT CCAAGTTCCA TTTCATTGAT TATGGTAACT ACTGATTATT TTCAATAACA
3841 AATACTTCGA AGACTGCACA ATTCAAAAGT ATGCCAGAAA GAAAGGATTA CTATCAATTG
3901 TGGGTTAATC AAACTAAGAC AGGTGGCAAA AATGGAACCA TTGATTAAGG CAGCCACTGA
3961 CCGATTTCAT TTAAAAAACA CACTCTTGGA AGTTTCCACA CAATCTCACC TTTTGCCAAT
4021 TTTAGCAAAG ACGTTGTGCT GCACTGATAA GAATCGAACT GTAAACATGT GGGCAGTAAA
4081 AATTATTTCA TCGTTCAACA CGGCGGTCAT TACACTATTC GAAGCAGCTG AAAAGATTTG
4141 ATGATAGCAG GACCGTGAGA TCAGCAAATT TGAGGTATAA AAGATGATCC TGCGACCACC
4201 AGAAGGCACA TTCGAGCTTT GGAGTGCATT CAAAGCATCC GGGCAACTGC GAACAACCGA
4261 ACCatCGATC TCGAGCACCC AACTTTTCTA TACAAAGTTg gtACCGAACC ATGTGGGTGC
4321 TGGGTGGCCG TCGGCTCCTG TGGAGCTTCC TGGTGAGCCT GGTGTTGATC CAAAGCGTGT
4381 CCGCCAAGAT CATCCAGGCG CAGCGCAACT TGAATGAGCT GTGTTATAAT GAAGGAAACG
4441 ATAACAAATT GTACCATGTG TTGAACAGCA AGAATGGTAA GATCTATAAT AGAAACACGG
4501 TCAACCGACT CTTGGCTGAT GCTCCGGAAG GAAAGAAAAA CGAGAAGAAA AATGAGAAGA
4561 TCGAGCGGAA TAACAAATTG AAGCAGCCTC CTCCGCCGCC CAACCCCAAC GATCCACCTC
4621 CACCAAACCC AAACGACCCG CCACCTCCCA ATCCAAATGA TCCGCCGCCG CCGAACCCGA
4681 ACGACCCTGC GCCTCCTAAC GCAAACGATC CGGCCCCACC GAATGCCAAC GATCCAGCCC
4741 CGCCGAACGC GAACGACCCT GCACCGCCTA ATGCCAACGA TCCGGCTCCG CCGAATGCAA
4801 ATGATCCAGC TCCACCGAAT GCAAATGACC CCGCGCCGCC TAACGCGAAT GATCCGCCTC
4861 CGCCGAATCC AAACGACCCG GCACCTCCGC AGGGAAACAA CAACCCGCAG CCGCAACCGC
4921 GTCCGCAGCC ACAACCCCAG CCCCAACCGC AGCCGCAGCC GCAGCCACAA CCTCAGCCGC
4981 GTCCGCAGCC GCAGCCTCAG CCGGGAGGAA ACAACAACAA CAAGAATAAT AACAATGATG
5041 ACTCCTACAT TCCTAGCGCA GAAAAGATCC TGGAGTTTGT GAAGCAAATC CGCGACTCGA
5101 TCACTGAGGA GTGGAGCCAG TGTAACGTGA CGTGCGGTTC GGGCATCCGC GTGCGCAAGC
5161 GTAAAGGCTC GAATAAGAAG GCTGAGGACC TGACGCTGGA AGATATCGAC ACGGAAATTT
5221 GCAAGATGGA CAAATGCGAA TTCtaaTCTA GACATAATCA GCCATACCAC ATTTGTAGAG
5281 GTTTTACTTG CTTTAAAAAA CCTCCCACAC CTCCCCCTGA ACCTGAAACA TAAAATGAAT
5341 GCAATTGTTG TTGTTAACTT GTTTATTGCA GCTTATAATG GTTACAAATA AAGCAATAGC
5401 ATCACAAATT TCACAAATAA AGCATTTTTC TTCACTGCAT TCTAGTTGTG GTTTGTCCAA
5461 ACTCATCAAT GTATCTcgag ACAACTTTGT ATAATAAAGT TGGTACCGGG CCCCCCGCTA
5521 GCGtcgaGTA CCGGGCCCGG ATCTAATTCA ATTAGAGACT AATTCAATTA GAGCTAATTC
5581 AATTAGGATC CAAGCTTATC GATTTCGAAC CCTCGACCGC CGGAGTATAA ATAGAGGCGC
5641 TTCGTCTACG GAGCGACAAT TCAATTCAAA CAAGCAAAGT GAACACGTCG CTAAGCGAAA
5701 GCTAAGCAAA TAAACAAGCG CAGCTGAACA AGCTAAACAA TCGGGGTACC GCTAGAGTCG
5761 ACGGTACCGC GGGCCCGGGA TCCACCGGTC GCCACCATGG TGAGCAAGGG CGAGGAGCTG
5821 TTCACCGGGG TGGTGCCCAT CCTGGTCGAG CTGGACGGCG ACGTAAACGG CCACAgGTTC
5881 AGCGTGTCCG GCGAGGGCGA GGGCGATGCC ACCTACGGCA AGCTGACCCT GAAGTTCATC
5941 TGCACCACCG GCAAGCTGCC CGTGCCCTGG CCCACCCTCG TGACCACCCT GACCTGGGGC
6001 GTGCAGTGCT TCAGCCGCTA CCCCGACCAC ATGAAGCAGC ACGACTTCTT CAAGTCCGCC
6061 ATGCCCGAAG GCTACGTCCA GGAGCGtACC ATCTTCTTCA AGGACGACGG CAACTACAAG
6121 ACCCGCGCCG AGGTGAAGTT CGAGGGCGAC ACCCTGGTGA ACCGCATCGA GCTGAAGGGC
6181 ATCGACTTCA AGGAGGACGG CAACATCCTG GGGCACAAGC TGGAGTACAA CTACATCAGC
6241 CACAACGTCT ATATCACCGC CGACAAGCAG AAGAACGGCA TCAAGGCCcA CTTCAAGATC
6301 CGCCACAACA TCGAGGACGG CAGCGTGCAG CTCGCCGACC ACTACCAGCA GAACACCCCC
6361 ATCGGCGACG GCCCCGTGCT GCTGCCCGAC AACCACTACC TGAGCACCCA GTCCGCCCTG
6421 AGCAAAGACC CCAACGAGAA GCGCGATCAC ATGGTCCTGC TGGAGTTCGT GACCGCCGCC
6481 GGGATCACTC TCGGCATGGA CGAGCTGTAC AAGTAAAGCG GCCGCGACTC TAGATCATAA
6541 TCAGCCATAC CACATTTGTA GAGGTTTTAC TTGCTTTAAA AAACCTCCCA CACCTCCCCC
6601 TGAACCTGAA ACATAAAATG AATGCAATTG TTGTTGTTAA CTTGTTTATT GCAGCTTATA
6661 ATGGTTACAA ATAAAGCAAT AGCATCACAA ATTTCACAAA TAAAGCATTT TTTTCACTGC
6721 ATTCTAGTTG TGGTTTGTCC AAACTCATCA ATGTATCTTA AAGCTTATCG ATACGCGTAC
6781 GGCGCGCCTA GAGCGGCCGC CACCGCGGTG GAGCTCGAGT ACCCAGCTTT cttgtacaaa
6841 gtggtttgat CGGTACGTAC CCAATTCGCC CTATAGTGAG TCGTATTACA ATTCACTGGC

```
     6901 CGTCGTTTTA CAACGTCGTG ACTGGGAAAA CCCTGGCGTT ACCCAACTTA ATCGCCTTGC
     6961 AGCACATCCC CCTTTCGCCA GCTGGCGTAA TAGCGAAGAG GCCCGCACCG ATCGCCCTTC
     7021 CCAACAGTTG CGCAGCCTGA ATGGCGAATG GAAATTGTAA GCGTTAATAT TTTGTTAAAA
     7081 TTCGCGTTAA ATTTTTGTTA AATCAGCTCA TTTTTTAACC AATAGGCCGA AATCGGCAAA
     7141 ATCCCTTATA AATCAAAAGA ATAGACCGAG ATAGGGTTGA GTGTTGTTCC AGTTTGGAAC
     7201 AAGAGTCCAC TATTAAAGAA CGTGGACTCC AACGTCAAAG GGCGAAAAAC CGTCTATCAG
     7261 GGCGATGGCC CACTACGTGA ACCATCACCC TAATCAAGTT TTTTGGGGTC GAGGTGCCGT
     7321 AAAGCACTAA ATCGGAACCC TAAAGGGAGC CCCCGATTTA GAGCTTGACG GGGAAAGCCG
     7381 GCGAACGTGG CGAGAAAGGA AGGGAAGAAA GCGAAAGGAG CGGGCGCTAG GGCGCTGGCA
     7441 AGTGTAGCGG TCACGCTGCG CGTAACCACC ACACCCGCCG CGCTTAATGC GCCGCTACAG
     7501 GGCGCGTCAG
//
```

pAttBRfB1GFP-Lp-OptCSP:

```
LOCUS       pattB-LpP-OptCSP        7496 bp    DNA     linear       18-NOV-2014
FEATURES             Location/Qualifiers
     misc_feature    complement(6855..6875)
                     /note="T7"
     misc_feature    complement(2216..2500)
                     /note="attB"
     misc_feature    2173..2192
                     /note="T3"
     misc_feature    2138..2156
                     /note="M13R"
     promoter        2525..4220
                     /source="Lp promoter"
     misc_feature    2516..2538
                     /note="attB1"
     misc_feature    4245..4319
                     /note="lipophorin signal peptide"
     misc_feature    4237..4244
                     /note="Vg Kozak"
     clone           4221..5533
                     /source="pENTR R4-optCSP-R3"
                     /type="Entry Clone"
     misc_feature    complement(4214..4233)
                     /note="attB4"
     misc_feature    5175..5279
                     /note="AgFas1 gpi anchoring signal"
     misc_feature    5202..5204
                     /note="gpi-modified serine"
     misc_feature    5292..5521
                     /note="SV40 term"
     misc_feature    5584..5789
                     /note="3xP3-hsp70"
     misc_feature    5809..6528
                     /note="GFP"
     misc_feature    5526..5547
                     /note="attB3"
     misc_feature    6546..6767
                     /note="SV40 term"
     misc_feature    complement(6810..6831)
                     /note="attB2"
ORIGIN
        1 GTGGCACTTT TCGGGGAAAT GTGCGCGGAA CCCCTATTTG TTTATTTTTC TAAATACATT
       61 CAAATATGTA TCCGCTCATG AGACAATAAC CCTGATAAAT GCTTCAATAA TATTGAAAAA
      121 GGAAGAGTAT GAGTATTCAA CATTTCCGTG TCGCCCTTAT TCCCTTTTTT GCGGCATTTT
      181 GCCTTCCTGT TTTTGCTCAC CCAGAAACGC TGGTGAAAGT AAAAGATGCT GAAGATCAGT
      241 TGGGTGCACG AGTGGGTTAC ATCGAACTGG ATCTCAACAG CGGTAAGATC CTTGAGAGTT
      301 TTCGCCCCGA AGAACGTTTT CCAATGATGA GCACTTTTAA AGTTCTGCTA TGTGGCGCGG
      361 TATTATCCCG TATTGACGCC GGGCAAGAGC AACTCGGTCG CCGCATACAC TATTCTCAGA
      421 ATGACTTGGT TGAGTACTCA CCAGTCACAG AAAAGCATCT TACGGATGGC ATGACAGTAA
      481 GAGAATTATG CAGTGCTGCC ATAACCATGA GTGATAACAC TGCGGCCAAC TTACTTCTGA
      541 CAACGATCGG AGGACCGAAG GAGCTAACCG CTTTTTTGCA CAACATGGGG GNATCATGTA
      601 ACTCGCCTTN GATCGTTGGG AACCGGAGCT GAATGAAGCC ATACCAAACG ACGAGCGTGA
      661 CACCACGATG CCTGTAGCAA TGGCAACAAC GTTGCGCAAA CTATTAACTG GCGAACTACT
      721 TACTCTAGCT TCCCGGCAAC AATTAATAGA CTGGATGGAG GCGGATAAAG TTGCAGGACC
      781 ACTTCTGCGC TCGGCCCTTC CGGCTGGCTG GTTTATTGCT GATAAATCTG GAGCCGGTGA
      841 GCGTGGGTCT CGCGGTATCA TTGCAGCACT GGGGCCAGAT GGTAAGCCCT CCCGTATCGT
      901 AGTTATCTAC ACGACGGGGA GTCAGGCAAC TATGGATGAA CGAAATAGAC AGATCGCTGA
      961 GATAGGTGCC TCACTGATTA AGCATTGGTA ACTGTCAGAC CAAGTTTACT CATATATACT
     1021 TTAGATTGAT TTAAAACTTC ATTTTTAATT TAAAAGGATC TAGGTGAAGA TCCTTTTTGA
     1081 TAATCTCATG ACCAAAATCC CTTAACGTGA GTTTTCGTTC CACTGAGCGT CAGACCCCGT
     1141 AGAAAAGATC AAAGGATCTT CTTGAGATCC TTTTTTTCTG CGCGTAATCT GCTGCTTGCA
     1201 AACAAAAAAA CCACCGCTAC CAGCGGTGGT TTGTTTGCCG GATCAAGAGC TACCAACTCT
     1261 TTTTCCGAAG GTAACTGGCT TCAGCAGAGC GCAGATACCA AATACTGTCC TTCTAGTGTA
```

1321 GCCGTAGTTA GGCCACCACT TCAAGAACTC TGTAGCACCG CCTACATACC TCGCTCTGCT
1381 AATCCTGTTA CCAGTGGCTG CTGCCAGTGG CGATAAGTCG TGTCTTACCG GGTTGGACTC
1441 AAGACGATAG TTACCGGATA AGGCGCAGCG GTCGGGCTGA ACGGGGGGTT CGTGCACACA
1501 GCCCAGCTTG GAGCGAACGA CCTACACCGA ACTGAGATAC CTACAGCGTG AGCTATGAGA
1561 AAGCGCCACG CTTCCCGAAG GGAGAAAGGC GGACAGGTAT CCGGTAAGCG GCAGGGTCGG
1621 AACAGGAGAG CGCACGAGGG AGCTTCCAGG GGGAAACGCC TGGTATCTTT ATAGTCCTGT
1681 CGGGTTTCGC CACCTCTGAC TTGAGCGTCG ATTTTTGTGA TGCTCGTCAG GGGGGCGGAG
1741 CCTATGGAAA AACGCCAGCA ACGCGGCCTT TTTACGGTTC CTGGCCTTTT GCTGGCCTTT
1801 TGCTCACATG TTCTTTCCTG CGTTATCCCC TGATTCTGTG GATAACCGTA TTACCGCCTT
1861 TGAGTGAGCT GATACCGCTC GCCGCAGCCG AACGACCGAG CGCAGCGAGT CAGTGAGCGA
1921 GGAAGCGGAA GAGCGCCCAA TACGCAAACC GCCTCTCCCC GCGCGTTGGC CGATTCATTA
1981 ATGCAGCTGG CACGACAGGT TTCCCGACTG GAAAGCGGGC AGTGAGCGCA ACGCAATTAA
2041 TGTGAGTTAG CTCACTCATT AGGCACCCCA GGCTTTACAC TTTATGCTTC CGGCTCGTAT
2101 GTTGTGTGGA ATTGTGAGCG GATAACAATT TCACACAGGA AACAGCTATG ACCATGATTA
2161 CGCCAAGCTC GAAATTAACC CTCACTAAAG GGAACAAAAG CTGGCTAGAA CTAGTGTCGA
2221 CATGCCCGCC GTGACCGTCG AGAACCCGCT GACGCTGCCC CGCGTATCCG CACCCGCCGA
2281 CGCCGTCGCA CGTCCCGTGC TCACCGTGAC CACCGCGCCC AGCGGTTTCG AGGGCGAGGG
2341 CTTCCCGGTG CGCCGCGCGT TCGCCGGGAT CAACTACCGC CACCTCGACC CGTTCATCAT
2401 GATGGACCAG ATGGGTGAGG TGGAGTACGC GCCCGGGGAG CCCAAGGGCA CGCCCTGGCA
2461 CCCGCACCGC GGCTTCGAGA CCGTGACCTA CATCGTCGAC ACTAGTGgat catcaaacaa
2521 gtttGTACAA AAAAGCAGGC TGGTACCGGG CCCCCCGCTA GCGTCGACGG TATCGATAAg
2581 cTTGCGGGGA AGACACATTC GAGATACGCT AAGTGATTGA GCGATTACGA TCTAGCAAAA
2641 CATACGTTCA GCTGTGAGAA TAATCATCCA TCTTCCTGCA ATGAGCAGTT CATTCCCGAT
2701 TGAGGGATTT TATTCCCCGG GGGCCTTTTC AAACGGCTTA ATATAAGCAA TTAATAGTAT
2761 TTTTTCTTTC AGGTTAGTTT ACTGTAATGG TGTAATTGTC ATCTTACACC TCCGTCTGAT
2821 AAGAGATTAC GAAGCTCAGT ATGATGAAAT AAATAAGATA AATTTATTTA AAAAAGAACA
2881 ATTGCTATGA GAGTGAAATA CAACAGTGGC GTTCACAATA TTCGAAAAAC AATAAAATTA
2941 AAAAAAAAAC AAGAAAACAT TCACAAACAT ATCAATCTGC TTTCATCGAC ACCGAACTGC
3001 TAGCCTCCCC AGTCTAACCG CGGTGGGGAC GTTTAATTGC CTTTGTTCTC GCACCCGGTC
3061 AAACATACAC TTCGGACCTT GCTCCGAACC CCACTGTGAT CCCTAGCTCG TCATCATCAT
3121 TGCCGGCATC ATGCTAAGCG TGCATTATTT TCACAACTTA GCGTAATGCT AGCGTGCGCT
3181 AGCAACAAAC TCGCCCGCAG ACTCGTCACA GCACCGGTAC GATCGATCGT TTACCGTTCC
3241 CTTTCCCGAT CGGGTTGGCT GCGATATCCG TGTCCGGGTA GAAAACTTCC CCTTTTACAC
3301 ACACACACTC ACATACACAC AGAGCTGAAT AGCAACTTAC CTTATCTGTT CGTCATCGCT
3361 CGGGCGGATC TGGACGAATC TTCGCACCGA TAACCATGTG GATCTACGAC CTCCGCTTGG
3421 CTGTCTCTCT GCTCATGTGT ATGTCTGTGT GTGTGTGTAT GTGAGCTTCT TCCCTCAAAT
3481 CCCTCGATCT CGCTGTGSCA ACAATCAAAC GTGCAAGTGC AAACATTGCA CCCCATTGAT
3541 TATACACCCA ACACCAACCA ATTCCCCTTG CGGAGGCATC TCTGTGCTCG GCAGCATGTT
3601 TACCGCAGAT CTACAGAGAA CTTCAATTGA GGTCCTTTCC ACCCCCAGCC CTCAACCGGC
3661 AATCCGGCAG CCACTGGATC ATCAGCGAAA GAGAGAGAGA GCAGAGCAGA ACAGAGGTGA
3721 CCAACTGTGG TATCGCTTCC CGCGCGCCGG TGTGTTGGTG TCCATTTCGG TGATCGCGAT
3781 CCCGGCCGCT TCCAGCACCG TCCACCGATC AGTCACAAAA ACGCTCTCCA AACCCCTTAT
3841 CAGCACCGTT CGCTGATGTG AACCCCCGTT CAAACCCCAA ATGCAGTGTT TGTATTGCTG
3901 TGTGTATGTG TACGTGCGTG TGTGTGGAAA TTCTATAAAA GTAGGCACCC GTGGCCGGGA
3961 TCCGTTATTC CCGTCCTGAG GCCCGCCCGG GATCGCTGGT GACGACAGAC GAGCCGCTGT
4021 GTGACGTACG TAGTGCCCGA TCGGTAAAGA GTGAACCGTC TTCTCTGCAG TGTAGGAGAG
4081 AACGGTTTCA TCTTTTTCGC CCACACCCCC CCGGTTACAT TCCATGTTGA ACTGTAAGGT
4141 CTAGTGAACA TTTCCTGAGT GTGGAAAGTG TGGTTTAGTG CGTGAGAGTG CACGGACACG
4201 ACACTCGAGC ACCCAACTTT TCTATACAAA GTTggtACCG AACCATGTGG GTGCTGGGTG
4261 GCCGTCGGCT CCTGTGGAGC TTCCTGGTGA GCCTGGTGTT GATCCAAAGC GTGTCCGCCA
4321 AGATCATCCA GGCGCAGCGC AACTTGAATG AGCTGTGTTA TAATGAAGGA AACGATAACA
4381 AATTGTACCA TGTGTTGAAC AGCAAGAATG GTAAGATCTA TAATAGAAAC ACGGTCAACC
4441 GACTCTTGGC TGATGCTCCG GAAGGAAAGA AAAACGAGAA GAAAAATGAG AAGATCGAGC
4501 GGAATAACAA ATTGAAGCAG CCTCCTCCGC CGCCCAACCC CAACGATCCA CCTCCACCAA
4561 ACCCAAACGA CCCGCCACCT CCCAATCCAA ATGATCCGCC GCCGCCGAAC CCGAACGACC
4621 CTGCGCCTCC TAACGCAAAC GATCCGGCCC CACCGAATGC CAACGATCCA GCCCCGCCGA
4681 ACGCGAACGA CCCTGCACCG CCTAATGCCA ACGATCCGGC TCCGCCGAAT GCAAATGATC
4741 CAGCTCCACC GAATGCAAAT GACCCCGCGC CGCCTAACGC GAATGATCCG CCTCCGCCGA
4801 ATCCAAACGA CCCGGCACCT CCGCAGGGAA ACAACAACCC GCAGCCGCAA CCGCGTCCGC
4861 AGCCACAACC CCAGCCCCAA CCGCAGCCGC AGCCGCAGCC ACAACCTCAG CCGCGTCCGC
4921 AGCCGCAGCC TCAGCCGGGA GGAAACAACA ACAACAAGAA TAATAACAAT GATGACTCCT
4981 ACATTCCTAG CGCAGAAAAG ATCCTGGAGT TTGTGAAGCA AATCCGCGAC TCGATCACTG
5041 AGGAGTGGAG CCAGTGTAAC GTGACGTGCG GTTCGGGCAT CCGCGTGCGC AAGCGTAAAG
5101 GCTCGAATAA GAAGGCTGAG GACCTGACGC TGGAAGATAT CGACACGGAA ATTTGCAAGA
5161 TGGACAAATG CGAATTCGTT CACGAGTACG ACATGATTAC GTCGGACGGC CATCACCTGT
5221 ACGGTATCGA ACCGTTGACC CTGCTGCTGG TGACCGTTCT CACCGCACTG GGAGCAGTTt
5281 aaGAATTCta aTCTAGACAT AATCAGCCAT ACCACATTTG TAGAGGTTTT ACTTGCTTTA
5341 AAAAACCTCC CACACCTCCC CCTGAACCTG AAACATAAAA TGAATGCAAT TGTTGTTGTT
5401 AACTTGTTTA TTGCAGCTTA TAATGGTTAC AAATAAAGCA ATAGCATCAC AAATTTCACA
5461 AATAAAGCAT TTTTCTTCAC TGCATTCTAG TTGTGGTTTG TCCAAACTCA TCAATGTATC
5521 TcgagACAAC TTTGTATAAT AAAGTTGGTA CCGGGCCCCC CGCTAGCGtc gaGTACCGGG
5581 CCCGGATCTA ATTCAATTAG AGACTAATTC AATTAGAGCT AATTCAATTA GGATCCAAGC
5641 TTATCGATTT CGAACCCTCG ACCGCCGGAG TATAAATAGA GGCGCTTCGT CTACGGAGCG
5701 ACAATTCAAT TCAAACAAGC AAAGTGAACA CGTCGCTAAG CGAAAGCTAA GCAAATAAAC
5761 AAGCGCAGCT GAACAAGCTA AACAATCGGC TCGAGACCGG TCGCCACCAT GGTGAGCAAG
5821 GGCGAGGAGC TGTTCACCGG GGTGGTGCCC ATCCTGGTCG AGCTGGACGG CGACGTAAAC
5881 GGCCACAAGT TCAGCGTGTC CGGCGAGGGC GAGGGCGATG CCACCTACGG CAAGCTGACC
5941 CTGAAGTTCA TCTGCACCAC CGGCAAGCTG CCCGTGCCCT GGCCCACCCT CGTGACCACC
6001 CTGACCTACG GCGTGCAGTG CTTCAGCCGC TACCCCGACC ACATGAAGCA GCACGACTTC
6061 TTCAAGTCCG CCATGCCCGA AGGCTACGTC CAGGAGCGCA CCATCTTCTT CAAGGACGAC
6121 GGCAACTACA AGACCCGCGC CGAGGTGAAG TTCGAGGGCG ACACCCTGGT GAACCGCATC

```
     6181 GAGCTGAAGG GCATCGACTT CAAGGAGGAC GGCAACATCC TGGGGCACAA GCTGGAGTAC
     6241 AACTACAACA GCCACAACGT CTATATCATG GCCGACAAGC AGAAGAACGG CATCAAGGTG
     6301 AACTTCAAGA TCCGCCACAA CATCGAGGAC GGCAGCGTGC AGCTCGCCGA CCACTACCAG
     6361 CAGAACACCC CCATCGGCGA CGGCCCCGTG CTGCTGCCCG ACAACCACTA CCTGAGCACC
     6421 CAGTCCGCCC TGAGCAAAGA CCCCAACGAG AAGCGCGATC ACATGGTCCT GCTGGAGTTC
     6481 GTGACCGCCG CCGGGATCAC TCTCGGCATG GACGAGCTGT ACAAGTAAAG CGGCCGCGAC
     6541 TCTAGATCAT AATCAGCCAT ACCACATTTG TAGAGGTTTT ACTTGCTTTA AAAAACCTCC
     6601 CACACCTCCC CCTGAACCTG AAACATAAAA TGAATGCAAT TGTTGTTGTT AACTTGTTTA
     6661 TTGCAGCTTA TAATGGTTAC AAATAAAGCA ATAGCATCAC AAATTTCACA AATAAAGCAT
     6721 TTTTTTCACT GCATTCTAGT TGTGGTTTGT CCAAACTCAT CAATGTATCT TAGCTAGAGC
     6781 GGCCGCCACC GCGGTGGAGC TCGAGTACCC AGCTTTcttg tacaaagtgg tttgatCGGT
     6841 ACGTACCCAA TTCGCCCTAT AGTGAGTCGT ATTACAATTC ACTGGCCGTC GTTTTACAAC
     6901 GTCGTGACTG GGAAAACCCT GGCGTTACCC AACTTAATCG CCTTGCAGCA CATCCCCCTT
     6961 TCGCCAGCTG GCGTAATAGC GAAGAGGCCC GCACCGATCG CCCTTCCCAA CAGTTGCGCA
     7021 GCCTGAATGG CGAATGGAAA TTGTAAGCGT TAATATTTTG TTAAAATTCG CGTTAAATTT
     7081 TTGTTAAATC AGCTCATTTT TTAACCAATA GGCCGAAATC GGCAAAATCC CTTATAAATC
     7141 AAAAGAATAG ACCGAGATAG GGTTGAGTGT TGTTCCAGTT TGGAACAAGA GTCCACTATT
     7201 AAAGAACGTG GACTCCAACG TCAAAGGGCG AAAAACCGTC TATCAGGGCG ATGGCCCACT
     7261 ACGTGAACCA TCACCCTAAT CAAGTTTTTT GGGGTCGAGG TGCCGTAAAG CACTAAATCG
     7321 GAACCCTAAA GGGAGCCCCC GATTTAGAGC TTGACGGGGA AAGCCGGCGA ACGTGGCGAG
     7381 AAAGGAAGGG AAGAAAGCGA AAGGAGCGGG CGCTAGGGCG CTGGCAAGTG TAGCGGTCAC
     7441 GCTGCGCGTA ACCACCACAC CCGCCGCGCT TAATGCGCCG CTACAGGGCG CGTCAG
//
```

pAttBRfB1YFP-Vg-OptCSP:

```
LOCUS       pattB-VgP-OptCSP        7562 bp    DNA     circular
FEATURES             Location/Qualifiers
     misc_feature    complement(6921..6941)
                     /note="T7"
     misc_feature    complement(2216..2500)
                     /note="attB"
     misc_feature    2173..2192
                     /note="T3"
     misc_feature    2138..2156
                     /note="M13R"
     misc_feature    2516..2538
                     /note="attB1"
     misc_feature    4311..4385
                     /note="lipophorin signal peptide"
     misc_feature    4303..4310
                     /note="Vg Kozak"
     clone           4287..5599
                     /source="pENTR R4-optCSP-R3"
                     /type="Entry Clone"
     misc_feature    complement(4280..4299)
                     /note="attB4"
     misc_feature    5241..5345
                     /note="AgFas1 gpi anchoring signal"
     misc_feature    5268..5270
                     /note="gpi-modified serine"
     misc_feature    5358..5587
                     /note="SV40 term"
     misc_feature    5650..5855
                     /note="3xP3-hsp70"
     misc_feature    5592..5613
                     /note="attB3"
     misc_feature    6612..6833
                     /note="SV40 term"
     misc_feature    complement(6876..6897)
                     /note="attB2"
     misc_feature    2516..2524
                     /note="RfB"
     misc_feature    2564..4265
                     /note="Vitellogenin promoter"
     gene            5875..6591
                     /note="EYFP (Clontech)"

ORIGIN
        1 GTGGCACTTT TCGGGGAAAT GTGCGCGGAA CCCCTATTTG TTTATTTTTC TAAATACATT
       61 CAAATATGTA TCCGCTCATG AGACAATAAC CCTGATAAAT GCTTCAATAA TATTGAAAAA
      121 GGAAGAGTAT GAGTATTCAA CATTTCCGTG TCGCCCTTAT TCCCTTTTTT GCGGCATTTT
      181 GCCTTCCTGT TTTTGCTCAC CCAGAAACGC TGGTGAAAGT AAAAGATGCT GAAGATCAGT
      241 TGGGTGCACG AGTGGGTTAC ATCGAACTGG ATCTCAACAG CGGTAAGATC CTTGAGAGTT
      301 TTCGCCCCGA AGAACGTTTT CCAATGATGA GCACTTTTAA AGTTCTGCTA TGTGGCGCGG
      361 TATTATCCCG TATTGACGCC GGGCAAGAGC AACTCGGTCG CCGCATACAC TATTCTCAGA
      421 ATGACTTGGT TGAGTACTCA CCAGTCACAG AAAAGCATCT TACGGATGGC ATGACAGTAA
```

```
 481 GAGAATTATG CAGTGCTGCC ATAACCATGA GTGATAACAC TGCGGCCAAC TTACTTCTGA
 541 CAACGATCGG AGGACCGAAG GAGCTAACCG CTTTTTTGCA CAACATGGGG GNATCATGTA
 601 ACTCGCCTTN GATCGTTGGG AACCGGAGCT GAATGAAGCC ATACCAAACG ACGAGCGTGA
 661 CACCACGATG CCTGTAGCAA TGGCAACAAC GTTGCGCAAA CTATTAACTG GCGAACTACT
 721 TACTCTAGCT TCCCGGCAAC AATTAATAGA CTGGATGGAG GCGGATAAAG TTGCAGGACC
 781 ACTTCTGCGC TCGGCCCTTC CGGCTGGCTG GTTTATTGCT GATAAATCTG GAGCCGGTGA
 841 GCGTGGGTCT CGCGGTATCA TTGCAGCACT GGGGCCAGAT GGTAAGCCCT CCCGTATCGT
 901 AGTTATCTAC ACGACGGGGA GTCAGGCAAC TATGGATGAA CGAAATAGAC AGATCGCTGA
 961 GATAGGTGCC TCACTGATTA AGCATTGGTA ACTGTCAGAC CAAGTTTACT CATATATACT
1021 TTAGATTGAT TTAAAACTTC ATTTTTAATT TAAAAGGATC TAGGTGAAGA TCCTTTTTGA
1081 TAATCTCATG ACCAAAATCC CTTAACGTGA GTTTTCGTTC CACTGAGCGT CAGACCCCGT
1141 AGAAAAGATC AAAGGATCTT CTTGAGATCC TTTTTTTCTG CGCGTAATCT GCTGCTTGCA
1201 AACAAAAAAA CCACCGCTAC CAGCGGTGGT TTGTTTGCCG GATCAAGAGC TACCAACTCT
1261 TTTTCCGAAG GTAACTGGCT TCAGCAGAGC GCAGATACCA AATACTGTCC TTCTAGTGTA
1321 GCCGTAGTTA GGCCACCACT TCAAGAACTC TGTAGCACCG CCTACATACC TCGCTCTGCT
1381 AATCCTGTTA CCAGTGGCTG CTGCCAGTGG CGATAAGTCG TGTCTTACCG GGTTGGACTC
1441 AAGACGATAG TTACCGGATA AGGCGCAGCG GTCGGGCTGA ACGGGGGGTT CGTGCACACA
1501 GCCCAGCTTG GAGCGAACGA CCTACACCGA ACTGAGATAC CTACAGCGTG AGCTATGAGA
1561 AAGCGCCACG CTTCCCGAAG GGAGAAAGGC GGACAGGTAT CCGGTAAGCG GCAGGGTCGG
1621 AACAGGAGAG CGCACGAGGG AGCTTCCAGG GGGAAACGCC TGGTATCTTT ATAGTCCTGT
1681 CGGGTTTCGC CACCTCTGAC TTGAGCGTCG ATTTTTGTGA TGCTCGTCAG GGGGGCGGAG
1741 CCTATGGAAA AACGCCAGCA ACGCGGCCTT TTTACGGTTC CTGGCCTTTT GCTGGCCTTT
1801 TGCTCACATG TTCTTTCCTG CGTTATCCCC TGATTCTGTG GATAACCGTA TTACCGCCTT
1861 TGAGTGAGCT GATACCGCTC GCCGCAGCCG AACGACCGAG CGCAGCGAGT CAGTGAGCGA
1921 GGAAGCGGAA GAGCGCCCAA TACGCAAACC GCCTCTCCCC GCGCGTTGGC CGATTCATTA
1981 ATGCAGCTGG CACGACAGGT TTCCCGACTG GAAAGCGGGC AGTGAGCGCA ACGCAATTAA
2041 TGTGAGTTAG CTCACTCATT AGGCACCCCA GGCTTTACAC TTTATGCTTC CGGCTCGTAT
2101 GTTGTGTGGA ATTGTGAGCG GATAACAATT TCACACAGGA AACAGCTATG ACCATGATTA
2161 CGCCAAGCTC GAAATTAACC CTCACTAAAG GGAACAAAAG CTGGCTAGAA CTAGTGTCGA
2221 CATGCCCGCC GTGACCGTCG AGAACCCGCT GACGCTGCCC CGCGTATCCG CACCCGCCGA
2281 CGCCGTCGCA CGTCCCGTGC TCACCGTGAC CACCGCGCCC AGCGGTTTCG AGGGCGAGGG
2341 CTTCCCGGTG CGCCGCGCGT TCGCCGGGAT CAACTACCGC CACCTCGACC CGTTCATCAT
2401 GATGGACCAG ATGGGTGAGG TGGAGTACGC GCCCGGGGAG CCCAAGGGCA CGCCCTGGCA
2461 CCCGCACCGC GGCTTCGAGA CCGTGACCTA CATCGTCGAC ACTAGTGgat catcaaacaa
2521 gtttGTACAA AAAAGCAGGC TGGTACCGGG CCCCCCGCTA GCGTCGAGTT CAACTCGACC
2581 ATAATAATTG ATCCGTCAAT CCATATTGGT CCGCAATAAT GAAAGTTGCA AGAGTACGAC
2641 GGTATGAAAA GAGGTTCAGT AAGTTGTAAA CTAATAGTTT CTTCCCAACG TTCAAATGCT
2701 GGCAAATCTT TTCGCGGGCC GCACTTCGTC GATCGCTAGT CTTAATGATA ATTTCTGAGA
2761 AAAAGGTGCT ACTGCATCTA CTATATTCTA CTGGATATAA ATGAAATAAC AACGTGAGAC
2821 TCACCTACAA CATGTAATTT ATTGATGGTT TAGTTTAACC AACCTATGAA ATAATTTGAT
2881 ATAGAAATTT GTAGTCGTTT TTCTATGAAG TAAAATTCTA AAATCAAACA TTAAACTGTT
2941 TTGTAGTACC CGGACTCATG GTATGGCTTC TATTAGCCGT AAACAAAGAT TTACAATTGA
3001 CTAAGGTTAG GTCCGACACT GTAGGAGCCA GCGCGTCCTT TCAATACATC AACGGACCAT
3061 CTCGTGTTGT TAAATACTTA TTATTATTAT GGTTTGCTAA TTGATATGTT CCAAGACCGA
3121 TTTGGATTTC GAAATAAGTA TTCTCTGATT CATTTTGGGA GCCGGTCTCG TGATACAGTC
3181 GTCAGCCCGT ACGACTTAAC TACATTCTCG TCATGGGTTC AAGCCCCAGA TGGACCGTGC
3241 CGCCATACGT AGAGTCAGTC CTATCCTGTT ATGGGGGGTA ATACATAAGA CACTGAAAGC
3301 CAACCCCACA AGTGGTACAG ACAAGCCTTG aCCGACAATT GTTGTTGAGC CAAACAGAAG
3361 AAGAATCCAT TTCGGGAAAT GATTTTATCA TTCAATCAAA CCAGTCAATC ATAAACATCA
3421 TAGTTTTAAA TACTCAAAAC TAGTTGAGAT CTTTAAAACA CATTATTTTA GTTTAATTAA
3481 ATGATCTGTT AGCTAGAAGG TAGATACGAT ATTTTAGACA TTTCGTAATA GATCGCAAAT
3541 CTCTATTATG TTGGTAATTC ACTTCGTAAA ACTCTTAGGC AAAACTCTTA TTAGTAAACA
3601 AAATACTAAT CAAACACTGA TAAACTAACG CGATTTATAC ATTGGACAAA GAAGAGGCTG
3661 ATTTTAAAAA TACTCGCTTT AAAATTTGCT TCATTCATCA ATGTATTGTA AAGCACATAA
3721 AGAACACAAT CATTGACTGA AAACAATTCC ACGTCTCAGC CAACTTCCAG GATCAATGAA
3781 ATAGCAAGTT CCAAGTTCCA TTTCATTGAT TATGGTAACT ACTGATTATT TTCAATAACA
3841 AATACTTCGA AGACTGCACA ATTCAAAAGT ATGCCAGAAA GAAAGGATTA CTATCAATTG
3901 TGGGTTAATC AAACTAAGAC AGGTGGCAAA AATGGAACCA TTGATTAAGG CAGCCACTGA
3961 CCGATTTCAT TTAAAAAACA CACTCTTGGA AGTTTCCACA CAATCTCACC TTTTGCCAAT
4021 TTTAGCAAAG ACGTTGTGCT GCACTGATAA GAATCGAACT GTAAACATGT GGGCAGTAAA
4081 AATTATTTCA TCGTTCAACA CGGCGGTCAT TACACTATTC GAAGCAGCTG AAAAGATTTG
4141 ATGATAGCAG GACCGTGAGA TCAGCAAATT TGAGGTATAA AAGATGATCC TGCGACCACC
4201 AGAAGGCACA TTCGAGCTTT GGAGTGCATT CAAAGCATCC GGGCAACTGC GAACAACCGA
4261 ACCatCGATC TCGAGCACCC AACTTTTCTA TACAAAGTTg gtACCGAACC ATGTGGGTGC
4321 TGGGTGGCCG TCGGCTCCTG TGGAGCTTCC TGGTGAGCCT GGTGTTGATC CAAAGCGTGT
4381 CCGCCAAGAT CATCCAGGCG CAGCGCAACT TGAATGAGCT GTGTTATAAT GAAGGAAACG
4441 ATAACAAATT GTACCATGTG TTGAACAGCA AGAATGGTAA GATCTATAAT AGAAACACGG
4501 TCAACCGACT CTTGGCTGAT GCTCCGGAAG GAAAGAAAAA CGAGAAGAAA AATGAGAAGA
4561 TCGAGCGGAA TAACAAATTG AAGCAGCCTC CTCCGCCGCC CAACCCCAAC GATCCACCTC
4621 CACCAAACCC AAACGACCCG CCACCTCCCA ATCCAAATGA TCCGCCGCCG CCGAACCCGA
4681 ACGACCCTGC GCCTCCTAAC GCAAACGATC CGGCCCCACC GAATGCCAAC GATCCAGCCC
4741 CGCCGAACGC GAACGACCCT GCACCGCCTA ATGCCAACGA TCCGGCTCCG CCGAATGCAA
4801 ATGATCCAGC TCCACCGAAT GCAAATGACC CCGCGCCGCC TAACGCGAAT GATCCGCCTC
4861 CGCCGAATCC AAACGACCCG GCACCTCCGC AGGGAAACAA CAACCCGCAG CCGCAACCGC
4921 GTCCGCAGCC ACAACCCCAG CCCCAACCGC AGCCGCAGCC GCAGCCACAA CCTCAGCCGC
4981 GTCCGCAGCC GCAGCCTCAG CCGGGAGGAA ACAACAACAA CAAGAATAAT AACAATGATG
5041 ACTCCTACAT TCCTAGCGCA GAAAAGATCC TGGAGTTTGT GAAGCAAATC CGCGACTCGA
5101 TCACTGAGGA GTGGAGCCAG TGTAACGTGA CGTGCGGTTC GGGCATCCGC GTGCGCAAGC
5161 GTAAAGGCTC GAATAAGAAG GCTGAGGACC TGACGCTGGA AGATATCGAC ACGGAAATTT
5221 GCAAGATGGA CAAATGCGAA TTCGTTCACG AGTACGACAT GATTACGTCG GACGGCCATC
5281 ACCTGTACGG TATCGAACCG TTGACCCTGC TGCTGGTGAC CGTTCTCACC GCACTGGGAG
```

```
     5341 CAGTTtaaGA ATTCtaaTCT AGACATAATC AGCCATACCA CATTTGTAGA GGTTTTACTT
     5401 GCTTTAAAAA ACCTCCCACA CCTCCCCCTG AACCTGAAAC ATAAAATGAA TGCAATTGTT
     5461 GTTGTTAACT TGTTTATTGC AGCTTATAAT GGTTACAAAT AAAGCAATAG CATCACAAAT
     5521 TTCACAAATA AAGCATTTTT CTTCACTGCA TTCTAGTTGT GGTTTGTCCA AACTCATCAA
     5581 TGTATCTcga gACAACTTTG TATAATAAAG TTGGTACCGG GCCCCCCGCT AGCGtcgaGT
     5641 ACCGGGCCCG GATCTAATTC AATTAGAGAC TAATTCAATT AGAGCTAATT CAATTAGGAT
     5701 CCAAGCTTAT CGATTTCGAA CCCTCGACCG CCGGAGTATA AATAGAGGCG CTTCGTCTAC
     5761 GGAGCGACAA TTCAATTCAA ACAAGCAAAG TGAACACGTC GCTAAGCGAA AGCTAAGCAA
     5821 ATAAACAAGC GCAGCTGAAC AAGCTAAACA ATCGGCTCGA GACCGGTCGC CACCATGGTG
     5881 AGCAAGGGCG AGGAGCTGTT CACCGGGGTG GTGCCCATCC TGGTCGAGCT GGACGGCGAC
     5941 GTAAACGGCC ACAAGTTCAG CGTGTCCGGC GAGGGCGAGG GCGATGCCAC CTACGGCAAG
     6001 CTGACCCTGA AGTTCATCTG CACCACCGGC AAGCTGCCCG TGCCCTGGCC CACCCTCGTG
     6061 ACCACCTTCG GCTACGGCCT GCAGTGCTTC GCCCGCTACC CCGACCACAT GAAGCAGCAC
     6121 GACTTCTTCA AGTCCGCCAT GCCCGAAGGC TACGTCCAGG AGCGCACCAT CTTCTTCAAG
     6181 GACGACGGCA ACTACAAGAC CCGCGCCGAG GTGAAGTTCG AGGGCGACAC CCTGGTGAAC
     6241 CGCATCGAGC TGAAGGGCAT CGACTTCAAG GAGGACGGCA ACATCCTGGG GCACAAGCTG
     6301 GAGTACAACT ACAACAGCCA CAACGTCTAT ATCATGGCCG ACAAGCAGAA GAACGGCATC
     6361 AAGGTGAACT TCAAGATCCG CCACAACATC GAGGACGGCA GCGTGCAGCT CGCCGACCAC
     6421 TACCAGCAGA ACACCCCCAT CGGCGACGGC CCCGTGCTGC TGCCCGACAA CCACTACCTG
     6481 AGCTACCAGT CCGCCCTGAG CAAAGACCCC AACGAGAAGC GCGATCACAT GGTCCTGCTG
     6541 GAGTTCGTGA CCGCCGCCGG GATCACTCTC GGCATGGACG AGCTGTACAA GTAAAGCGGC
     6601 CGCGACTCTA GATCATAATC AGCCATACCA CATTTGTAGA GGTTTTACTT GCTTTAAAAA
     6661 ACCTCCCACA CCTCCCCCTG AACCTGAAAC ATAAAATGAA TGCAATTGTT GTTGTTAACT
     6721 TGTTTATTGC AGCTTATAAT GGTTACAAAT AAAGCAATAG CATCACAAAT TTCACAAATA
     6781 AAGCATTTTT TTCACTGCAT TCTAGTTGTG GTTTGTCCAA ACTCATCAAT GTATCTTAGC
     6841 TAGAGCGGCC GCCACCGCGG TGGAGCTCGA GTACCCAGCT TTcttgtaca aagtggtttg
     6901 atCGGTACGT ACCCAATTCG CCCTATAGTG AGTCGTATTA CAATTCACTG GCCGTCGTTT
     6961 TACAACGTCG TGACTGGGAA AACCCTGGCG TTACCCAACT TAATCGCCTT GCAGCACATC
     7021 CCCCTTTCGC CAGCTGGCGT AATAGCGAAG AGGCCCGCAC CGATCGCCCT TCCCAACAGT
     7081 TGCGCAGCCT GAATGGCGAA TGGAAATTGT AAGCGTTAAT ATTTTGTTAA AATTCGCGTT
     7141 AAATTTTTGT TAAATCAGCT CATTTTTTAA CCAATAGGCC GAAATCGGCA AAATCCCTTA
     7201 TAAATCAAAA GAATAGACCG AGATAGGGTT GAGTGTTGTT CCAGTTTGGA ACAAGAGTCC
     7261 ACTATTAAAG AACGTGGACT CCAACGTCAA AGGGCGAAAA ACCGTCTATC AGGGCGATGG
     7321 CCCACTACGT GAACCATCAC CCTAATCAAG TTTTTTGGGG TCGAGGTGCC GTAAAGCACT
     7381 AAATCGGAAC CCTAAAGGGA GCCCCCGATT TAGAGCTTGA CGGGGAAAGC CGGCGAACGT
     7441 GGCGAGAAAG GAAGGGAAGA AAGCGAAAGG AGCGGGCGCT AGGGCGCTGG CAAGTGTAGC
     7501 GGTCACGCTG CGCGTAACCA CCACACCCGC CGCGCTTAAT GCGCCGCTAC AGGGCGCGTC
     7561 AG
//
```

pAttBRfB1RFP-Lp-OptCSPΔgpi:

```
LOCUS       pattB-LpP-OptCSP        7370 bp    DNA     circular
FEATURES             Location/Qualifiers
     misc_feature    complement(6729..6749)
                     /note="T7"
     misc_feature    complement(2216..2500)
                     /note="attB"
     misc_feature    2173..2192
                     /note="T3"
     misc_feature    2138..2156
                     /note="M13R"
     clone           2525..4220
                     /source="pENTR L1-lpProm-L4 4"
                     /type="Entry Clone"
     misc_feature    2516..2538
                     /note="attB1"
     misc_feature    4245..4319
                     /note="lipophorin signal peptide"
     misc_feature    4237..4244
                     /note="Vg Kozak"
     clone           4221..5422
                     /source="pENTR R4-optCSP-R3"
                     /type="Entry Clone"
     misc_feature    complement(4214..4233)
                     /note="attB4"
     misc_feature    5181..5410
                     /note="SV40 term"
     misc_feature    5473..5678
                     /note="3xP3-hsp70"
     misc_feature    5415..5436
                     /note="attB3"
     misc_feature    6390..6615
                     /note="SV40 term"
     misc_feature    complement(6684..6705)
                     /note="attB2"
```

```
     misc_feature    5697..6379
                     /note="DsRed"
     misc_feature    6621..6646
                     /note="insertion"

ORIGIN
        1 GTGGCACTTT TCGGGGAAAT GTGCGCGGAA CCCCTATTTG TTTATTTTTC TAAATACATT
       61 CAAATATGTA TCCGCTCATG AGACAATAAC CCTGATAAAT GCTTCAATAA TATTGAAAAA
      121 GGAAGAGTAT GAGTATTCAA CATTTCCGTG TCGCCCTTAT TCCCTTTTTT GCGGCATTTT
      181 GCCTTCCTGT TTTTGCTCAC CCAGAAACGC TGGTGAAAGT AAAAGATGCT GAAGATCAGT
      241 TGGGTGCACG AGTGGGTTAC ATCGAACTGG ATCTCAACAG CGGTAAGATC CTTGAGAGTT
      301 TTCGCCCCGA AGAACGTTTT CCAATGATGA GCACTTTTAA AGTTCTGCTA TGTGGCGCGG
      361 TATTATCCCG TATTGACGCC GGGCAAGAGC AACTCGGTCG CCGCATACAC TATTCTCAGA
      421 ATGACTTGGT TGAGTACTCA CCAGTCACAG AAAAGCATCT TACGGATGGC ATGACAGTAA
      481 GAGAATTATG CAGTGCTGCC ATAACCATGA GTGATAACAC TGCGGCCAAC TTACTTCTGA
      541 CAACGATCGG AGGACCGAAG GAGCTAACCG CTTTTTTGCA CAACATGGGG GNATCATGTA
      601 ACTCGCCTTN GATCGTTGGG AACCGGAGCT GAATGAAGCC ATACCAAACG ACGAGCGTGA
      661 CACCACGATG CCTGTAGCAA TGGCAACAAC GTTGCGCAAA CTATTAACTG GCGAACTACT
      721 TACTCTAGCT TCCCGGCAAC AATTAATAGA CTGGATGGAG GCGGATAAAG TTGCAGGACC
      781 ACTTCTGCGC TCGGCCCTTC CGGCTGGCTG GTTTATTGCT GATAAATCTG GAGCCGGTGA
      841 GCGTGGGTCT CGCGGTATCA TTGCAGCACT GGGGCCAGAT GGTAAGCCCT CCCGTATCGT
      901 AGTTATCTAC ACGACGGGGA GTCAGGCAAC TATGGATGAA CGAAATAGAC AGATCGCTGA
      961 GATAGGTGCC TCACTGATTA AGCATTGGTA ACTGTCAGAC CAAGTTTACT CATATATACT
     1021 TTAGATTGAT TTAAAACTTC ATTTTTAATT TAAAAGGATC TAGGTGAAGA TCCTTTTTGA
     1081 TAATCTCATG ACCAAAATCC CTTAACGTGA GTTTTCGTTC CACTGAGCGT CAGACCCCGT
     1141 AGAAAAGATC AAAGGATCTT CTTGAGATCC TTTTTTTCTG CGCGTAATCT GCTGCTTGCA
     1201 AACAAAAAAA CCACCGCTAC CAGCGGTGGT TTGTTTGCCG GATCAAGAGC TACCAACTCT
     1261 TTTTCCGAAG GTAACTGGCT TCAGCAGAGC GCAGATACCA AATACTGTCC TTCTAGTGTA
     1321 GCCGTAGTTA GGCCACCACT TCAAGAACTC TGTAGCACCG CCTACATACC TCGCTCTGCT
     1381 AATCCTGTTA CCAGTGGCTG CTGCCAGTGG CGATAAGTCG TGTCTTACCG GGTTGGACTC
     1441 AAGACGATAG TTACCGGATA AGGCGCAGCG GTCGGGCTGA ACGGGGGGTT CGTGCACACA
     1501 GCCCAGCTTG GAGCGAACGA CCTACACCGA ACTGAGATAC CTACAGCGTG AGCTATGAGA
     1561 AAGCGCCACG CTTCCCGAAG GGAGAAAGGC GGACAGGTAT CCGGTAAGCG GCAGGGTCGG
     1621 AACAGGAGAG CGCACGAGGG AGCTTCCAGG GGGAAACGCC TGGTATCTTT ATAGTCCTGT
     1681 CGGGTTTCGC CACCTCTGAC TTGAGCGTCG ATTTTTGTGA TGCTCGTCAG GGGGGCGGAG
     1741 CCTATGGAAA AACGCCAGCA ACGCGGCCTT TTTACGGTTC CTGGCCTTTT GCTGGCCTTT
     1801 TGCTCACATG TTCTTTCCTG CGTTATCCCC TGATTCTGTG GATAACCGTA TTACCGCCTT
     1861 TGAGTGAGCT GATACCGCTC GCCGCAGCCG AACGACCGAG CGCAGCGAGT CAGTGAGCGA
     1921 GGAAGCGGAA GAGCGCCCAA TACGCAAACC GCCTCTCCCC GCGCGTTGGC CGATTCATTA
     1981 ATGCAGCTGG CACGACAGGT TTCCCGACTG GAAAGCGGGC AGTGAGCGCA ACGCAATTAA
     2041 TGTGAGTTAG CTCACTCATT AGGCACCCCA GGCTTTACAC TTTATGCTTC CGGCTCGTAT
     2101 GTTGTGTGGA ATTGTGAGCG GATAACAATT TCACACAGGA AACAGCTATG ACCATGATTA
     2161 CGCCAAGCTC GAAATTAACC CTCACTAAAG GGAACAAAAG CTGGCTAGAA CTAGTGTCGA
     2221 CATGCCCGCC GTGACCGTCG AGAACCCGCT GACGCTGCCC CGCGTATCCG CACCCGCCGA
     2281 CGCCGTCGCA CGTCCCGTGC TCACCGTGAC CACCGCGCCC AGCGGTTTCG AGGGCGAGGG
     2341 CTTCCCGGTG CGCCGCGCGT TCGCCGGGAT CAACTACCGC CACCTCGACC CGTTCATCAT
     2401 GATGGACCAG ATGGGTGAGG TGGAGTACGC GCCCGGGGAG CCCAAGGGCA CGCCCTGGCA
     2461 CCCGCACCGC GGCTTCGAGA CCGTGACCTA CATCGTCGAC ACTAGTGgat catcaaacaa
     2521 gtttGTACAA AAAAGCAGGC TGGTACCGGG CCCCCCGCTA GCGTCGACGG TATCGATAAg
     2581 cTTGCGGGGA AGACACATTC GAGATACGCT AAGTGATTGA GCGATTACGA TCTAGCAAAA
     2641 CATACGTTCA GCTGTGAGAA TAATCATCCA TCTTCCTGCA ATGAGCAGTT CATTCCCGAT
     2701 TGAGGGATTT TATTCCCCGG GGGCCTTTTC AAACGGCTTA ATATAAGCAA TTAATAGTAT
     2761 TTTTTCTTTC AGGTTAGTTT ACTGTAATGG TGTAATTGTC ATCTTACACC TCCGTCTGAT
     2821 AAGAGATTAC GAAGCTCAGT ATGATGAAAT AAATAAGATA AATTTATTTA AAAAAGAACA
     2881 ATTGCTATGA GAGTGAAATA CAACAGTGGC GTTCACAATA TTCGAAAAAC AATAAAATTA
     2941 AAAAAAAAAC AAGAAAACAT TCACAAACAT ATCAATCTGC TTTCATCGAC ACCGAACTGC
     3001 TAGCCTCCCC AGTCTAACCG CGGTGGGGAC GTTTAATTGC CTTTGTTCTC GCACCCGGTC
     3061 AAACATACAC TTCGGACCTT GCTCCGAACC CCACTGTGAT CCCTAGCTCG TCATCATCAT
     3121 TGCCGGCATC ATGCTAAGCG TGCATTATTT TCACAACTTA GCGTAATGCT AGCGTGCGCT
     3181 AGCAACAAAC TCGCCCGCAG ACTCGTCACA GCACCGGTAC GATCGATCGT TTACCGTTCC
     3241 CTTTCCCGAT CGGGTTGGCT GCGATATCCG TGTCCGGGTA GAAAACTTCC CCTTTTACAC
     3301 ACACACACTC ACATACACAC AGAGCTGAAT AGCAACTTAC CTTATCTGTT CGTCATCGCT
     3361 CGGGCGGATC TGGACGAATC TTCGCACCGA TAACCATGTG GATCTACGAC CTCCGCTTGG
     3421 CTGTCTCTCT GCTCATGTGT ATGTCTGTGT GTGTGTGTAT GTGAGCTTCT TCCCTCAAAT
     3481 CCCTCGATCT CGCTGTGSCA ACAATCAAAC GTGCAAGTGC AAACATTGCA CCCCATTGAT
     3541 TATACACCCA ACACCAACCA ATTCCCCTTG CGGAGGCATC TCTGTGCTCG GCAGCATGTT
     3601 TACCGCAGAT CTACAGAGAA CTTCAATTGA GGTCCTTTCC ACCCCCAGCC CTCAACCGGC
     3661 AATCCGGCAG CCACTGGATC ATCAGCGAAA GAGAGAGAGA GCAGAGCAGA ACAGAGGTGA
     3721 CCAACTGTGG TATCGCTTCC CGCGCGCCGG TGTGTTGGTG TCCATTTCGG TGATCGCGAT
     3781 CCCGGCCGCT TCCAGCACCG TCCACCGATC AGTCACAAAA ACGCTCTCCA AACCCCTTAT
     3841 CAGCACCGTT CGCTGATGTG AACCCCCGTT CAAACCCCAA ATGCAGTGTT TGTATTGCTG
     3901 TGTGTATGTG TACGTGCGTG TGTGTGGAAA TTCTATAAAA GTAGGCACCC GTGGCCGGGA
     3961 TCCGTTATTC CCGTCCTGAG GCCCGCCCGG GATCGCTGGT GACGACAGAC GAGCCGCTGT
     4021 GTGACGTACG TAGTGCCCGA TCGGTAAAGA GTGAACCGTC TTCTCTGCAG TGTAGGAGAG
     4081 AACGGTTTCA TCTTTTTCGC CCACACCCCC CCGGTTACAT TCCATGTTGA ACTGTAAGGT
     4141 CTAGTGAACA TTTCCTGAGT GTGGAAAGTG TGGTTTAGTG CGTGAGAGTG CACGGACACG
     4201 ACACTCGAGC ACCCAACTTT TCTATACAAA GTTggtACCG AACCATGTGG GTGCTGGGTG
     4261 GCCGTCGGCT CCTGTGGAGC TTCCTGGTGA GCCTGGTGTT GATCCAAAGC GTGTCCGCCA
     4321 AGATCATCCA GGCGCAGCGC AACTTGAATG AGCTGTGTTA TAATGAAGGA AACGATAACA
     4381 AATTGTACCA TGTGTTGAAC AGCAAGAATG GTAAGATCTA TAATAGAAAC ACGGTCAACC
     4441 GACTCTTGGC TGATGCTCCG GAAGGAAAGA AAAACGAGAA GAAAAATGAG AAGATCGAGC
```

```
     4501 GGAATAACAA ATTGAAGCAG CCTCCTCCGC CGCCCAACCC CAACGATCCA CCTCCACCAA
     4561 ACCCAAACGA CCCGCCACCT CCCAATCCAA ATGATCCGCC GCCGCCGAAC CCGAACGACC
     4621 CTGCGCCTCC TAACGCAAAC GATCCGGCCC CACCGAATGC CAACGATCCA GCCCCGCCGA
     4681 ACGCGAACGA CCCTGCACCG CCTAATGCCA ACGATCCGGC TCCGCCGAAT GCAAATGATC
     4741 CAGCTCCACC GAATGCAAAT GACCCCGCGC CGCCTAACGC GAATGATCCG CCTCCGCCGA
     4801 ATCCAAACGA CCCGGCACCT CCGCAGGGAA ACAACAACCC GCAGCCGCAA CCGCGTCCGC
     4861 AGCCACAACC CCAGCCCCAA CCGCAGCCGC AGCCGCAGCC ACAACCTCAG CCGCGTCCGC
     4921 AGCCGCAGCC TCAGCCGGGA GGAAACAACA ACAACAAGAA TAATAACAAT GATGACTCCT
     4981 ACATTCCTAG CGCAGAAAAG ATCCTGGAGT TTGTGAAGCA AATCCGCGAC TCGATCACTG
     5041 AGGAGTGGAG CCAGTGTAAC GTGACGTGCG GTTCGGGCAT CCGCGTGCGC AAGCGTAAAG
     5101 GCTCGAATAA GAAGGCTGAG GACCTGACGC TGGAAGATAT CGACACGGAA ATTTGCAAGA
     5161 TGGACAAATG CGAATTCtaa TCTAGACATA ATCAGCCATA CCACATTTGT AGAGGTTTTA
     5221 CTTGCTTTAA AAAACCTCCC ACACCTCCCC CTGAACCTGA AACATAAAAT GAATGCAATT
     5281 GTTGTTGTTA ACTTGTTTAT TGCAGCTTAT AATGGTTACA AATAAAGCAA TAGCATCACA
     5341 AATTTCACAA ATAAAGCATT TTTCTTCACT GCATTCTAGT TGTGGTTTGT CCAAACTCAT
     5401 CAATGTATCT cgagACAACT TTGTATAATA AAGTTGGTAC CGGGCCCCCC GCTAGCGtcg
     5461 aGTACCGGGC CCGGATCTAA TTCAATTAGA GACTAATTCA ATTAGAGCTA ATTCAATTAG
     5521 GATCCAAGCT TATCGATTTC GAACCCTCGA CCGCCGGAGT ATAAATAGAG GCGCTTCGTC
     5581 TACGGAGCGA CAATTCAATT CAAACAAGCA AAGTGAACAC GTCGCTAAGC GAAAGCTAAG
     5641 CAAATAAACA AGCGCAGCTG AACAAGCTAA ACAATCGGCT CGAGACCGGT CGCCACCATG
     5701 GTGCGCTCCT CCAAGAACGT CATCAAGGAG TTCATGCGCT TCAAGGTGCG CATGGAGGGC
     5761 ACCGTGAACG GCCACGAGTT CGAGATCGAG GGCGAGGGCG AGGGCCGCCC CTACGAGGGC
     5821 CACAACACCG TGAAGCTGAA GGTGACCAAG GGCGGCCCCC TGCCCTTCGC CTGGGACATC
     5881 CTGTCCCCCC AGTTCCAGTA CGGCTCCAAG GTGTACGTGA AGCACCCCGC CGACATCCCC
     5941 GACTACAAGA AGCTGTCCTT CCCCGAGGGC TTCAAGTGGG AGCGCGTGAT GAACTTCGAG
     6001 GACGGCGGCG TGGTGACCGT GACCCAGGAC TCCTCCCTGC AGGACGGCTG CTTCATCTAC
     6061 AAGGTGAAGT TCATCGGCGT GAACTTCCCC TCCGACGGCC CCGTAATGCA GAAGAAGACC
     6121 ATGGGCTGGG AGGCCTCCAC CGAGCGCCTG TACCCCCGCG ACGGCGTGCT GAAGGGCGAG
     6181 ATCCACAAGG CCCTGAAGCT GAAGGACGGC GGCCACTACC TGGTGGAGTT CAAGTCCATC
     6241 TACATGGCCA AGAAGCCCGT GCAGCTGCCC GGCTACTACT ACGTGGACTC CAAGCTGGAC
     6301 ATCACCTCCC ACAACGAGGA CTACACCATC GTGGAgCAGT ACGAGCGCAC CGAGGGCCGC
     6361 CACCACCTGT TCCTGTAGCG GCCGCGACTC TAGATCATAA TCAGCCATAC CACATTTGTA
     6421 GAGGTTTTAC TTGCTTTAAA AAACCTCCCA CACCTCCCCC TGAACCTGAA ACATAAAATG
     6481 AATGCAATTG TTGTTGTTAA CTTGTTTATT GCAGCTTATA ATGGTTACAA ATAAAGCAAT
     6541 AGCATCACAA ATTTCACAAA TAAAGCATTT TTTTCACTGC ATTCTAGTTG TGGTTTGTCC
     6601 AAACTCATCA ATGTATCTTA AAGCTTATCG ATACGCGTAC GGCGCGcCTA GAGCGGCCGC
     6661 CACCGCGGTG GAGCTCGAGT ACCCAGCTTT cttgtacaaa gtggtttgat CGGTACGTAC
     6721 CCAATTCGCC CTATAGTGAG TCGTATTACA ATTCACTGGC CGTCGTTTTA CAACGTCGTG
     6781 ACTGGGAAAA CCCTGGCGTT ACCCAACTTA ATCGCCTTGC AGCACATCCC CCTTTCGCCA
     6841 GCTGGCGTAA TAGCGAAGAG GCCCGCACCG ATCGCCCTTC CCAACAGTTG CGCAGCCTGA
     6901 ATGGCGAATG GAAATTGTAA GCGTTAATAT TTTGTTAAAA TTCGCGTTAA ATTTTTGTTA
     6961 AATCAGCTCA TTTTTTAACC AATAGGCCGA AATCGGCAAA ATCCCTTATA AATCAAAAGA
     7021 ATAGACCGAG ATAGGGTTGA GTGTTGTTCC AGTTTGGAAC AAGAGTCCAC TATTAAAGAA
     7081 CGTGGACTCC AACGTCAAAG GGCGAAAAAC CGTCTATCAG GGCGATGGCC CACTACGTGA
     7141 ACCATCACCC TAATCAAGTT TTTTGGGGTC GAGGTGCCGT AAAGCACTAA ATCGGAACCC
     7201 TAAAGGGAGC CCCCGATTTA GAGCTTGACG GGGAAAGCCG GCGAACGTGG CGAGAAAGGA
     7261 AGGGAAGAAA GCGAAAGGAG CGGGCGCTAG GGCGCTGGCA AGTGTAGCGG TCACGCTGCG
     7321 CGTAACCACC ACACCCGCCG CGCTTAATGC GCCGCTACAG GGCGCGTCAG
//
```

Table S1 Comparison of transgenesis efficiency using two promoters controlling *piggyBac* transposase expression. The data summarize many transgenesis experiments in which *piggyBac* constructs were micro-injected into *A. gambiae* embryos together with helper plasmid expressing transposase under control of either the Drosophila *hsp70* or the *A. gambiae vasa* (*Vas2*) promoter. Using the *Vas2* promoter, 3.4 x more transiently expressing survivor larvae gave transgenic progeny and 15 x more transgenic larvae could be recovered.

Transposase promoter in helper plasmid	*hsp70*	*Vas2*
Number of injected eggs	1421	950
Surviving larvae (%)	155 (10.9%)	137 (14.4%)
Transiently expressing larvae (%)	71 (45.8%)	79 (57.6%)
% transiently expressing larvae that gave rise to transgenics	5.6%	18.9%
Average number of transgenics per transient	1.75	27.1
