# Supplementary material for: Tools for Anopheles gambiae Transgenesis
Source: G3 (Bethesda). 2015 Apr 13;5(6):1151–63. doi: 10.1534/g3.115.016808 (PMC4478545; doi:10.1534/g3.115.016808)
Supplement: Supporting Information [file supp_g3.115.016808_FigureS1.pdf]

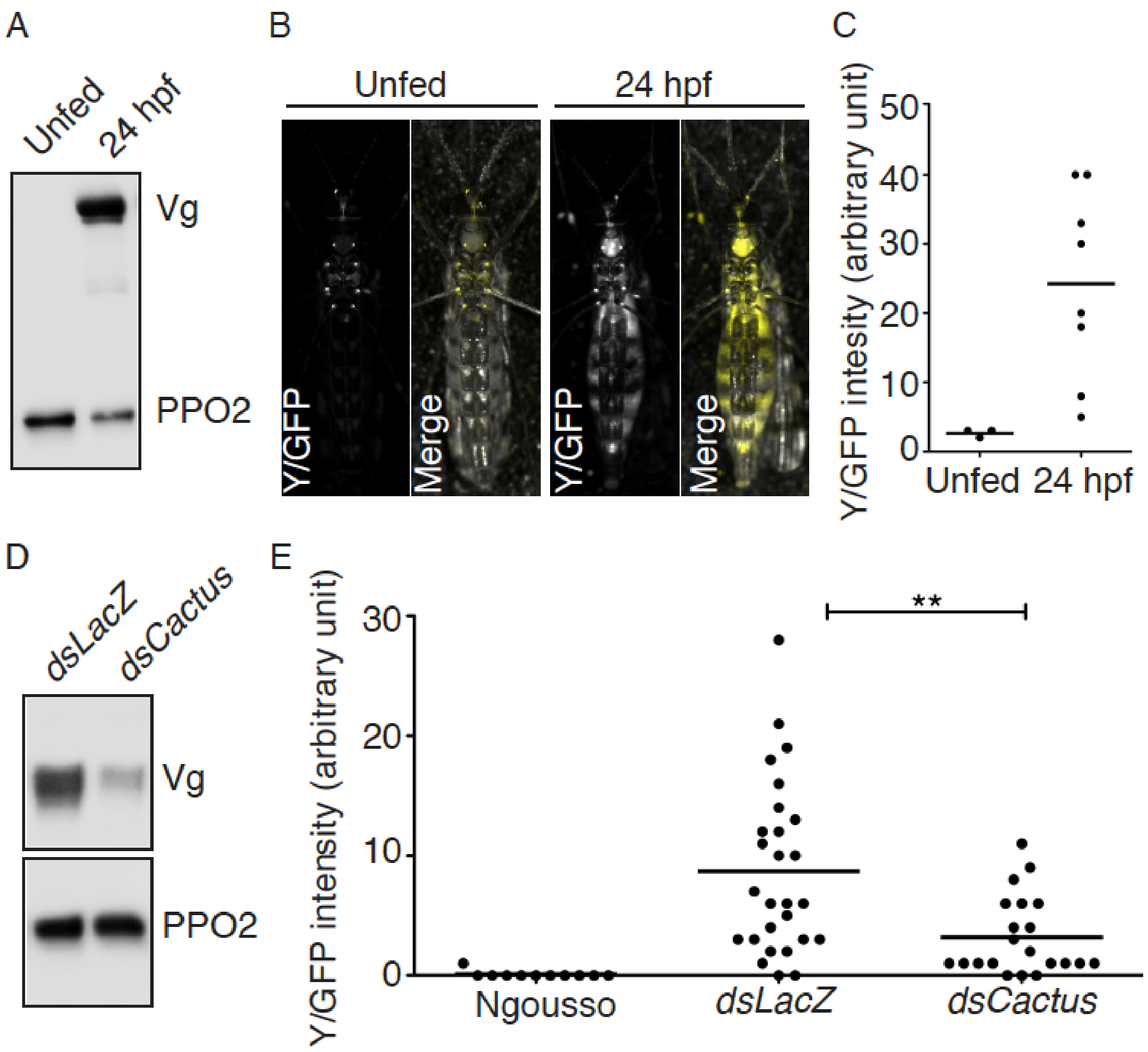

Figure S1 Expression of the *Vg::YFP* reporter in the FK line reproduces endogenous *Vg* expression. A: Expression of Vitellogenin (Vg) monitored by immunoblotting of mosquito hemolymph extracts from unfed and 24 h blood-fed FK mosquitoes. Anti-PPO2 antibodies were used as a loading control. B: Fluorescence analysis of FK mosquitoes reveals expression of the *Vg-YFP* reporter in the fat body 24 h post feeding while expression of the transgenesis marker *3xP3-GFP* in mosquito neurons is seen in both unfed and fed mosquitoes. C: Quantification of the YFP/GFP signal in the mosquito bodies expressed as mean of intensity. D: FK mosquitoes were injected with a control dsRNA (*dsLacZ*) or *dsCactus* and offered a blood meal infected with *P. berghei*. Immunoblotting was performed on the hemolymph extracted 52 h after infection using anti-Vitellogenin and anti-PPO2 antibodies. E: Quantification of YFP/GFP intensities from the same mosquitoes as in D 52 h after infection. Non-transgenic Ngousso mosquitoes were used as a control. **: p≤0.01 (1 way ANOVA). Pictures were acquired with a Leica AF6000E fluorescent stereomicroscope equipped with an M205 FA module. Images were analyzed with FIJI, by quantification of ROI in the delineated regions corresponding to the mosquito abdomen/thorax. Mean fluorescence intensity of the YFP/GFP signal was measured for each individual mosquito.
