# Supplementary material for: Tools for Anopheles gambiae Transgenesis
Source: G3 (Bethesda). 2015 Apr 13;5(6):1151–63. doi: 10.1534/g3.115.016808 (PMC4478545; doi:10.1534/g3.115.016808)
Supplement: Supporting Information [file supp_g3.115.016808_FigureS2.pdf]

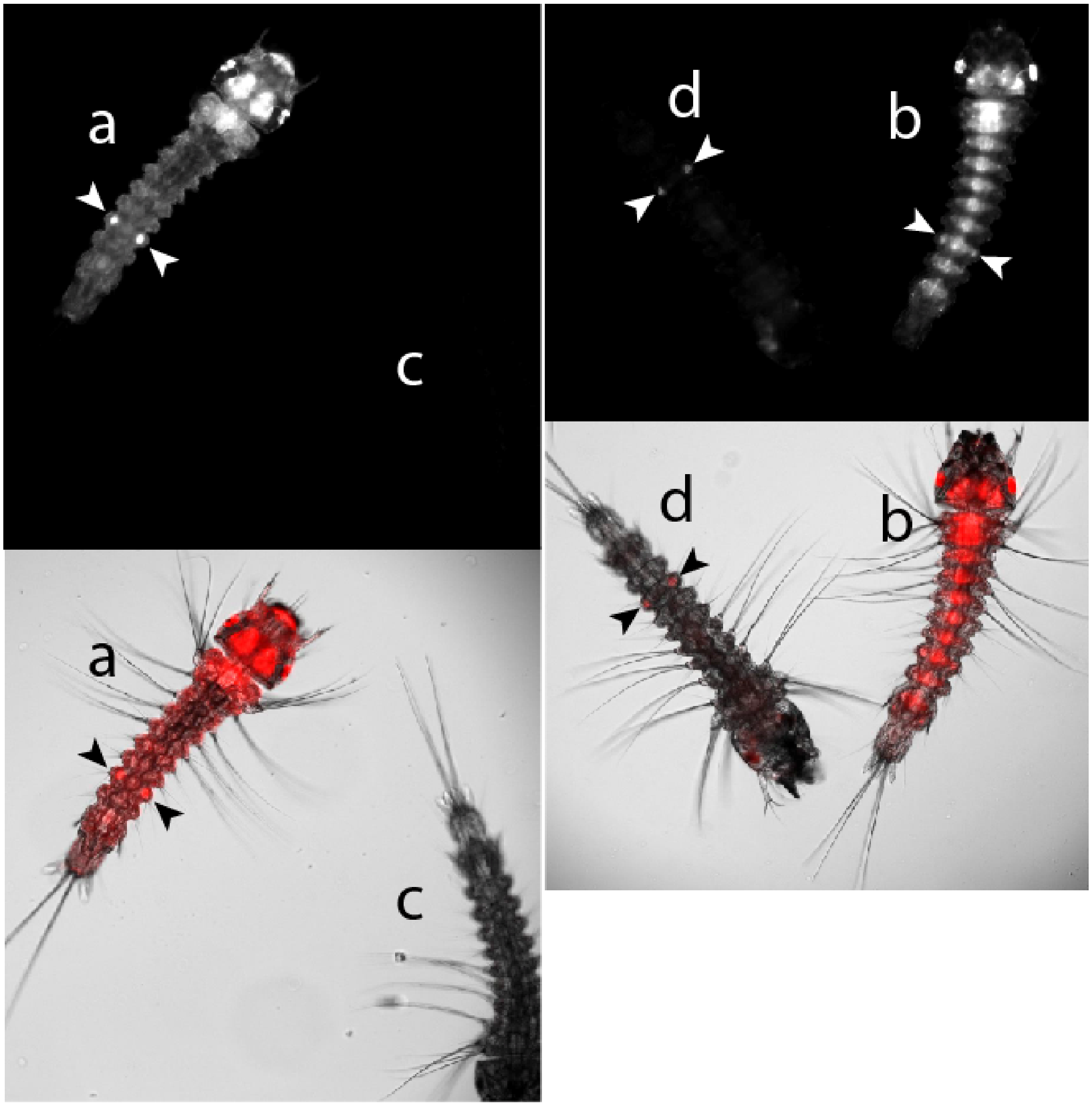

Figure S2 Neonate larvae from heterozygous *vas2-tdTomato* x wild type crosses, red channel (top panels) and merged red plus bright field channels (bottom panels). Larva (a), seen from the dorsal side, inherited the transgene and systemic red fluorescence from its mother. Larva (b) same, ventral side. Larva (c) had a transgenic heterozygous father but did not inherit the transgene. Larva (d) inherited the transgene from its father, expression is mainly restricted to the gonad and eyes. Expression in the eyes likely results from the nearby 3xP3 element.
