# Supplementary material for: Tools for Anopheles gambiae Transgenesis
Source: G3 (Bethesda). 2015 Apr 13;5(6):1151–63. doi: 10.1534/g3.115.016808 (PMC4478545; doi:10.1534/g3.115.016808)
Supplement: Supporting Information [file supp_g3.115.016808_FigureS3.pdf]

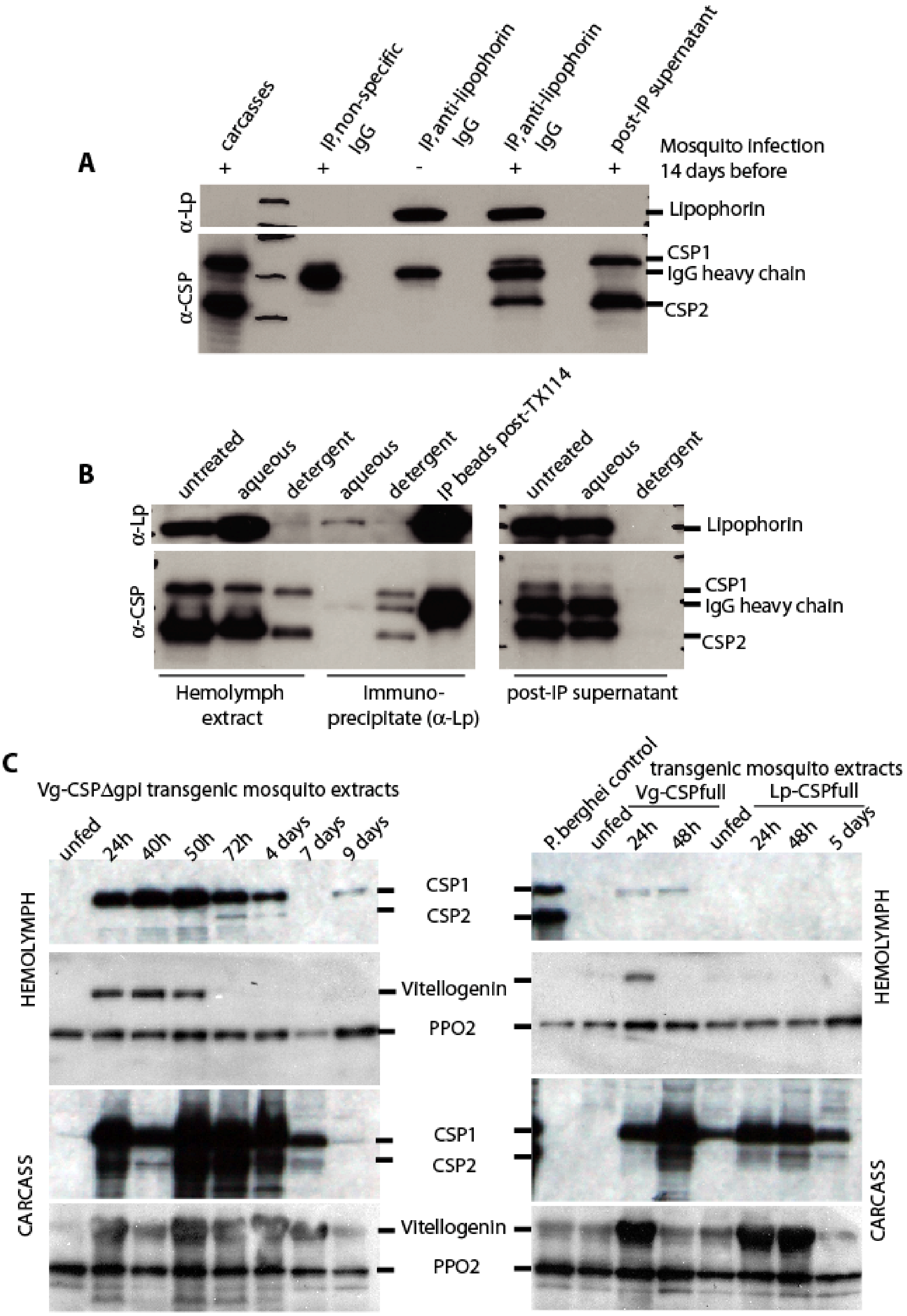
A
carcasses
IP, non-specific IgG
IP, anti-lipophorin IgG
IP, anti-lipophorin IgG
post-IP supernatant
Mosquito infection 14 days before
+
+
-
+
+
α-Lp
α-CSP
Lipophorin
CSP1
IgG heavy chain
CSP2
B
untreated
aqueous
detergent
aqueous
detergent
IP beads post-TX114
untreated
aqueous
detergent
α-Lp
α-CSP
Lipophorin
CSP1
IgG heavy chain
CSP2
Hemolymph extract
Immuno-precipitate (α-Lp)
post-IP supernatant
C
Vg-CSPΔgpi transgenic mosquito extracts
unfed
24h
40h
50h
72h
4 days
7 days
9 days
P. berghei control
transgenic mosquito extracts
Vg-CSPfull
Lp-CSPfull
unfed
24h
48h
unfed
24h
48h
5 days
HEMOLYMPH
CARCASS
CSP1
CSP2
Vitellogenin
PPO2
CSP1
CSP2
Vitellogenin
PPO2
HEMOLYMPH
CARCASS

Figure S3 ***Plasmodium berghei*** **CSP in mosquitoes. A: CSP associates with lipophorin particles in mosquito hemolymph.** Twenty infected females were anaesthetized and bled in 350 µl buffer. The extract was centrifuged and subjected to lipophorin immunoprecipitation (see Methods). Western blot with anti-lipophorin antibody (top panel) or CSP antibody (bottom panel), note that the secondary antibody reveals the mouse primary antibody used in the IP (IgG heavy chain) in addition to the CSP signal. Some released CSP associates with Lipophorin particles, while a large fraction remains in lipophorin-depleted supernatant. **B: CSP is released both without and with a GPI anchor and GPI-linked released CSP associates with lipophorin.** Triton X-114 protein fractionation (see Methods) of raw mosquito hemolymph extracts, immunoprecipitated lipophorin captured on sepharose beads and post-immunoprecipitation supernatant. CSP present in hemolymph extracts partitions in both aqueous (no GPI anchor) and detergent (GPI anchor) phases. CSP present on immunoprecipitated lipophorin exclusively partitions in the detergent phase (GPI anchored). Most lipophorin protein bound to antibodies and sepharose beads remained attached to the beads throughout the detergent fractionation process. No GPI-linked CSP remains in the lipophorin-depleted post-IP supernatant. **C: Codon-optimized *CSP* is expressed in transgenic mosquitoes**. Western blot was performed on mosquito hemolymph and whole body (carcass) extracts prepared at the indicated times after blood feeding with antibodies recognizing CSP, Vitellogenin (to monitor *Vg* promoter activity) and PPO2 (used as a loading control). Note that although both CSPΔGPI (left panel) and full-length CSP (right panel) expressions are highly induced by the blood meal (in addition to constitutive expression under the control of the *Lp* promoter, right panel) in tissues (carcasses), only CSPΔGPI is released to high levels in hemolymph. Also note that CSP appears to be cleaved inefficiently in transgenic mosquitoes compared to the *P. berghei* control, suggesting that mosquitoes lack the protease that usually cleaves CSP. Antibodies to Lipophorin, Vitellogenin and PPO2 are described in Rono Rono *et al.* (2010) and Fraiture *et al.* (2009).
