# Supplementary material for: Tools for Anopheles gambiae Transgenesis
Source: G3 (Bethesda). 2015 Apr 13;5(6):1151–63. doi: 10.1534/g3.115.016808 (PMC4478545; doi:10.1534/g3.115.016808)
Supplement: Supporting Information [file supp_g3.115.016808_FileS1.pdf]

File S1

Docking lines

DNA sequence of *Anopheles gambiae att*P docking lines X1, X13, X6 and XK. *att*P sites are embedded in a small *piggyBac* transposon; sequence is provided in its genomic context with flanking segments from the mosquito chromosome.

X1 line (2L: 1802562)

```
LOCUS       X1 locus       12061 bp    DNA     circular     20-Jan-2010
FEATURES             Location/Qualifiers
     misc_feature    1193..1328
                     /note="PiggyBac 5'region"
     misc_feature    798..1018
                     /note="attP'"
     misc_feature    504..661
                     /note="PiggyBac 3'region"
     misc_feature    754..787
                     /note="loxP"
     misc_feature    1..503
                     /note="A. gambiae chromosome 2L"
     misc_feature    1329..1745
                     /note="A. gambiae chromosome 2L"

ORIGIN
        1 ctcaataaca tgcccgtcat gagttcaagc ctgaaataga ccgtcccccc gcagcaaaga
       61 tttgacttat ctggctgctt cgtaattaat taagtctcca aagcctgtat aggccggcat
      121 gtccgcgtag ggcgttacgc caaatagaag aagagaagac acgtatgtca taccctcgta
      181 tttatgtata gataaatatt cttaccttcc agaaaaaagt ttgtgtcatc atctgattct
      241 accgtggata caaagcttta tttatttctc ttttacaact acaatagtat ttttaatgat
      301 tctttaaaat agtgtatgta gtttcagact catcgcaaaa aaaaataaat aaagtttaac
      361 tgcttccagg agaacttgaa gctaccaata atttataggg aagattggaa tccatccatt
      421 ggacgatgat attatttgct ttaagaagag atatattacg gcaaacatta gtgaagataa
      481 caccAttttg tattttgttt taaccctaga aagataatca tattgtgacg tacgttaaag
      541 ataatcatgc gtaaaattga cgcatgtgtt ttatcggtct gtatatcgag gtttatttat
      601 taatttgaat agatattaag ttttattata tttacactta catactaata ataaattcaa
      661 caaacaattt atttatgttt atttatttat taaaaaaaac aaaaactcaa aatttcttct
      721 ataaagtaac aaaactttta tcgaattgag ctcataactt cgtataatgt atgctatacg
      781 aagttatggt acctgcagta ctgacggaca caccgaagcc ccggcggcaa ccctcagcgg
      841 atgccccggg gcttcacgtt ttcccaggtc agaagcggtt ttcgggagta gtgccccaac
      901 tggggtaacc tttgagttct ctcagttggg ggcgtagggt cgccgacatg acacaagggg
      961 ttgtgaccgg ggtggacacg tacgcgggtg cttacgaccg tcagtcgcgc gagcgcgatc
     1021 taatctataa caagaaaata tatatataat aagttatcac gtaagtagaa catgaaataa
     1081 caatataatt atcgtatgag ttaaatctta aaagtcacgt aaaagataat catgcgtcat
     1141 tttgactcac gcggtcgtta tagttcaaaa tcagtgacac ttaccgcatt gacaagcacg
     1201 cctcacggga gctccaagcg gcgactgaga tgtcctaaat gcacagcgac ggattcgcgc
     1261 tatttagaaa gagagagcaa tatttcaaga atgcatgcgt caattttacg cagactatct
     1321 ttctagggtt aagtAtcatg aatatgtggt aatGaTatta atCgaatgag cattAaggga
     1381 ccaaccataa attacgtaac aatatcagtg ggccaacgat attttagacc gaacaaatag
     1441 ataggagagt tctagactgt gTatcaaaac atgaacataa ttAaaaaaaa cttatttaat
     1501 ttctgttgga aaaCactttg gttaatcaag atagttttga aagtttattt caaaagtcta
     1561 agctatcacg atagttgtgc aattactttg tctctaaaag caacagtgtg aatgggttgc
     1621 agttgttctt gtttggatga gttattttca cctccttcgg cttatttctt tcttattttt
     1681 agttattgga tttacatatg tcgagggagc tggcgtttta aaggcgaaag aattgtaccg
     1741 gaatt
//
```

X13 line (2L: 2798380)

```
LOCUS       X13 locus       12772 bp    DNA     circular     20-Jan-2010
FEATURES             Location/Qualifiers
     misc_feature    1099..1319
                     /note= "attP'"
     misc_feature    805..962
                     /note="PiggyBac 3'region"
     misc_feature    1055..1088
                     /note="loxP"
     misc_feature    1494..1629
                     /note="PiggyBac 5' region"
     misc_feature    1630..2459
                     /note="A. gambiae chromosome 2L"
     misc_feature    1..804
                     /note="A. gambiae chromosome 2L"

ORIGIN
        1 gggtggacaa gtcaacgctt attggtatgc agtggtttcg ctcgttggtc aaacctcggt
       61 ttgacctcta cggaaagggc ggaaaggagg aaaagagcga aagcaacagg catgttgatg
      121 caccacacac gcactgacgc aagatgccac agggatggga aacttaactg gccaatattg
      181 acaattttcc gcttacgtgt gtaaacaatg agcgccgaga gtatattgat tttctatcga
      241 ttgtgcatcc ctttaacctg cacggactga ctttaaacga gtgggtgttt atcgaagacg
      301 agtgcgtaac gagtgtgaag gcagcatgct ggaagcaaag cgacgttctt ctaactatcc
      361 tcatcacatc aagcaatccg acacatctaa acggaaacat tttatggtgg gctctgtcat
      421 cgtgcatcgc cgctcgccaa atgtctatat tgatggctat aaacacataa agcaatcgtg
      481 gccgcttgcc ctcaccgggg ctccactctc agctctcgaa gcttttaccc ggcgcacaga
      541 cgaatggcgt atttatgcga ccaacgaatg gcccaaccct tggttggctg gtgttttatt
      601 ttactttttt ttgcgacgct tggcttcctg gatgcgagcg gagccgtaaa agtggtcgaa
      661 aatgaaaata caccccggaa atgccggagt ccacggagtc acgaaatcaa tttccaccag
      721 ctgaaggatg cttgggttcc tttttatgt gtttgcctgc agtcgttgcc ggtatttgac
      781 gcattgcccc tgggaggttt ttaaccctag aaagataatc atattgtgac gtacgttaaa
      841 gataatcatg cgtaaaattg acgcatgtgt tttatcggtc tgtatatcga ggtttattta
      901 ttaatttgaa tagatattaa gttttattat atttacactt acatactaat aataaattca
      961 acaaacaatt tatttatgtt tatttattta ttaaaaaaaa caaaaactca aaatttcttc
     1021 tataaagtaa caaaactttt atcgaattga gctcataact tcgtataatg tatgctatac
     1081 gaagttatgg tacctgcagt actgacggac acaccgaagc cccggcggca accctcagcg
     1141 gatgccccgg ggcttcacgt tttcccaggt cagaagcggt tttcgggagt agtgccccaa
     1201 ctggggtaac ctttgagttc tctcagttgg gggcgtaggg tcgccgacat gacacaaggg
     1261 gttgtgaccg gggtggacac gtacgcgggt gcttacgacc gtcagtcgcg cgagcgcgat
     1321 ctaatctata acaagaaaat atatatataa taagttatca cgtaagtaga acatgaaata
     1381 acaatataat tatcgtatga gttaaatctt aaaagtcacg taaaagataa tcatgcgtca
     1441 ttttgactca cgcggtcgtt atagttcaaa atcagtgaca cttaccgcat tgacaagcac
     1501 gcctcacggg agctccaagc ggcgactgag atgtcctaaa tgcacagcga cggattcgcg
     1561 ctatttagaa agagagagca atatttcaag aatgcatgcg tcaattttac gcagactatc
     1621 tttctagggt taagggggac tgggtggaag catttgtttt tgctcgcttc ttttctctcg
     1681 ttCTGTCTCT ATCTatctct gctttgtgcg tagggcgtag ggatgtatga aaaagcttaT
     1741 tgcgtgtgat ggagttacac aagtacgtac atattcacaa cgacctggag tggggttgat
     1801 aattttagat ttcgaggatg gttttgaagg ctttataatc cccatgagtg gggatgttct
     1861 gggcatgatg ttgtaattat tttattaagg gtttccgact gacatgtgtg ttaacagcga
     1921 gtgtggcggt gttttgtgcc aggcgcttaa atgaaatgca ggaaatattt tcaattggcg
     1981 aaacatccta tgctgctttt tattctgtag tatgaagaca attaaaattt taattcaatt
     2041 tcaattgcat gccttaatgc gctccaaaca atgaacggag ggcctcttta tttacttttg
     2101 tagaataatt actttaattt tgatttgcag ctactaacgc ttatgggcct tgttttgtac
     2161 atagtacatt ctacatttga atcacaaaga atactattat aatcattctt gtacataaaa
     2221 actgtgttaa aaaagaggtg tcaaagcgtc tttcctaatc acacatgaac tatcatgtat
     2281 taaagagttc atactttttt taaataattg aaacttattg cagttgattt tatcagtatt
     2341 gtttttgat ataacagata agctcttgac ttaatctaca tattaaagaa cttatcatca
     2401 tttttctgac ttacctgaat gactatgact aatgtcactt gacaggtgaa agtggatgg
//
```

X6 line (3R: 53037011)

```
LOCUS       X6 locus                2127 bp    DNA     linear       12-MAY-2009
FEATURES             Location/Qualifiers
     misc_feature    691..724
                     /note="loxP"
     misc_feature    735..955
                     /note="attP'"
     misc_feature    complement(1130..1265)
                     /note="PiggyBac 5' region"
     misc_feature    complement(441..598)
                     /note="PiggyBac 3'region"
     misc_feature    1..440
                     /note="A. gambiae chromosome 3R"
     misc_feature    1266..2127
                     /note="A. gambiae chromosome 3R"
     source          1..2127
                     /dnas_title="X6"
ORIGIN
        1 GCAAAACGGA AGGAGGAAAT GTAACAAAGA ATAGGTTTCA ACTAGGCATG GGCAAAAGAA
       61 TTCTTTTCAC CGATCGCGAG CGTACTAGTT CGCTCAGTAA AAAGAACCGA ACGCGAACGG
      121 CGATTCTATC GTTAATTTTT TAATCAATAT GACCCAGCCA TATCCCTAGC AGGATTTTCT
      181 ATTAACAAAT TATTTATTCG TTATTTAGCC TGCATTAATA ATTTGCATTA CCCATGATTG
      241 TGCATAGTAA AGTTGCAGAA GTGTATACAA CCACGCGGTT GAACCAAAAG CAAACAAGAA
      301 ATTTAACTAA CAAACCGCGT TGTCTTATTT CCAATTTTAC AATATATTTA CATACTAGTT
      361 GATATCATAG AGTGTGCCTG GCAAAATGTA TCATTCATTT AGTTGTGTTT GAAAATTATT
      421 TCATAGTTCA TTATGtTTAA CCCTAGAAAG ATAATCATAT TGTGACGTAC GTTAAAGATA
      481 ATCATGCGTA AAATTGACGC ATGTGTTTTA TCGGTCTGTA TATCGAGGTT TATTTATTAA
      541 TTTGAATAGA TATTAAGTTT TATTATATTT ACACTTACAT ACTAATAATA AATTCAACAA
      601 ACAATTTATT TATGTTTATT TATTTATTAA AAAAAACAAA AACTCAAAAT TTCTTCTATA
      661 AAGTAACAAA ACTTTTATCG AATTGAGCTC ataacttcgt ataatgtatg ctatacgaag
      721 ttatGGTACC TGCAGTACTG ACGGACACAC CGAAGCCCCG GCGGCAACCC TCAGCGGATG
      781 CCCCGGGGCT TCACGTTTTC CCAGGTCAGA AGCGGTTTTC GGGAGTAGTG CCCCAACTGG
      841 GGTAACCTTT GAGTTCTCTC AGTTGGGGGC GTAGGGTCGC CGACATGACA CAAGGGGTTG
      901 TGACCGGGGT GGACACGTAC GCGGGTGCTT ACGACCGTCA GTCGCGCGAG CGCGATCTAA
      961 TCTATAACAA GAAAATATAT ATATAATAAG TTATCACGTA AGTAGAACAT GAAATAACAA
     1021 TATAATTATC GTATGAGTTA AATCTTAAAA GTCACGTAAA AGATAATCAT GCGTCATTTT
     1081 GACTCACGCG GTCGTTATAG TTCAAAATCA GTGACACTTA CCGCATTGAC AAGCACGCCT
     1141 CACGGGAGCT CCAAGCGGCG ACTGAGATGT CCTAAATGCA CAGCGACGGA TTCGCGCTAT
     1201 TTAGAAAGAG AGAGCAATAT TTCAAGAATG CATGCGTCAA TTTTACGCAG ACTATCTTTC
     1261 TAGGGTTAAC AATTATTAAC TCTTcACAAA ATTTAAAAAA AAAGCTCAAC TATACCTTGA
     1321 CTGTCTTACT AAGAGAAAAA ATCAAAAGAA TATATTATGA ATTGTGTTGT AAGAAAACTA
     1381 ATTCGTGTAG TTTTTTAGGT AGTAATCTTG TTCGTTTTTC TGAAAATATC TAGCCTGTTT
     1441 TGGAGAAAAC TCTCTCAAGA GGGTTTTCCA TTGCTAAAAT GTACAATGTT GGATATTTCA
     1501 TACGATTTTC TTGCCACCAT GATAGAGGAT CTCGTTCCAA TGCAATTGGA TTTACCTTAA
     1561 GGTACAAATT AATTTCGGCT TCCGCAAGTT CCCTAAGAGT ATAAGATGTT TCCAATTCAG
     1621 AATGACAGAC TATGTCGCCA TATAATAAGT TTGCATCAGT ATTAGATTTG AAAGCATCCT
     1681 CTCTTACATT ACATCCTCTT ATATTTGTTT GCTCTCCTGA AGGGGAAGCA TATCCGTTAG
     1741 TATCAAATCA TGTGTTTTTT TTAGTTTATC GGGTTCATTC TGAAACCCCG CTTTCATCAC
     1801 ACGTGGATCT AGGAACATTG CTTTCCAAAT TTGATGGTTA TCTCTATACA ATTTAAGTTT
     1861 TTCCTCTGTG TTTTCAACTA GTAAAGATAT CAAAGTCTTT ACTTCTGGGA CTAATTCGTT
     1921 GTTCAATAGC AAAGTTGAAG TTTTACGCAA TAATACATTA GCTAACGTGT GAAAGGGTCG
     1981 TATGTTGTTC CGCCGAAACT AATTTAGTTT CTGATGCAAA ATATTTGAGT GCCTCCAATC
     2041 ATTGCTCGAT AATTACCCAA TCAGTGGGTG ACAAGTTGGT TTTCATTTTT AAGCTATCTA
     2101 CGCACTAAAG AATGGGTATT TTATTTT
//
```

XK line (X: 22463468)

```
LOCUS       XK                       1666 bp    DNA     linear
```

```
FEATURES             Location/Qualifiers
     misc_feature    1248..1383
                     /note="PiggyBac 5'TR"
     misc_feature    853..1073
                     /note="attP'"
     misc_feature    809..842
                     /note="loxP"
     misc_feature    1..558
                     /note="A. gambiae X chromosome"
     misc_feature    559..804
                     /note="PiggyBac 3' region"
     misc_feature    1384..1666
                     /note="A. gambiae X chromosome"
     source          1..1666
                     /dnas_title="XK"
ORIGIN
        1 TATCTCGAAT TTCTGCGTAG GTCGAGCCTC TTAATTTTTA GCCAAAATAT CACTAATTTC
       61 GTTTAATTGT GCAAGGGGGT GGTTTTAGCC ACGTAATCTA TTATTTGATA TGAGTCTGAT
      121 TAAATTTAAC AGTTTTAATC CTGTTGATTT GGTTTTAAAC ATTCGATCAA AAAGGTAATT
      181 ATTTTGCATT TGACAAAAAT TGATGAGTCA AATCAGTACA ATTTGCTCAA AGAACTGTCA
      241 TATTTAGAAT CTCCTATTCC GCGTATTAGA GGTACCGTGT ATCTCAGGGA CTTCCTGTAC
      301 TTTTGTATCT TGTTGTTTGT TACACGGAAT GATTTTTCCT GAGTGCACCA TGAATTATGC
      361 TGCTACTCGC ACGACGCTGA TGATGCACCT CACGAATAGA TACAAGAGAT TTCGGGAGTT
      421 TTCTTAATTT TGGGACACTT TCTTGATTGT GTTTTACGAG TAGTGAGAAC TTTATGTGAT
      481 AAGAAGTGAA GTCTAAGTGT TGAAAAACGC GGTGGATTTT TTAGAGAAAA CACTTTATTT
      541 ACTGTGGTAT GAGGTTAAcc ctagaaagat aatcatattg tgacgtacgt taaagataat
      601 catgcgtaaa attgacgcat gtgttttatc ggtctgtata tcgaggttta tttattaatt
      661 tgaatagata ttaagtttta ttatatttac acttacatac taataataaa ttcaacaaac
      721 aatttattta tgtttattta tttattaaaa aaaacaaaaa ctcaaaattt cttctataaa
      781 gtaacaaaac ttttatcgaa ttgagctcat aacttcgtat aatgtatgct atacgaagtt
      841 atggtacctg cagtactgac ggacacaccg aagccccggc ggcaaccctc agcggatgcc
      901 ccggggcttc acgttttccc aggtcagaag cggttttcgg gagtagtgcc ccaactgggg
      961 taacctttga gttctctcag ttgggggcgt agggtcgccg acatgacaca aggggttgtg
     1021 accggggtgg acacgtacgc gggtgcttac gaccgtcagt cgcgcgagcg cgatctaatc
     1081 tataacaaga aaatatatat ataataagtt atcacgtaag tagaacatga aataacaata
     1141 taattatcgt atgagttaaa tcttaaaagt cacgtaaaag ataatcatgc gtcattttga
     1201 ctcacgcggt cgttatagtt caaaatcagt gacacttacc gcattgacaa gcacgcctca
     1261 cgggagctcc aagcggcgac tgagatgtcc taaatgcaca gcgacggatt cgcgctattt
     1321 agaaagagag agcaatattt caagaatgca tgcgtcaatt ttacgcagac tatctttcta
     1381 gggTTAAATC CAAAAATTAG TCCACTTGTA CCGATTGTCG AATTTCAACT AACTGGTTAG
     1441 GAGCGGACCA ACCCTTCTCC TTATCTCCTA ATTTACCGTT TTTATCCTCC CTCTCACTCT
     1501 CTTCTCTAGC GTTGTCACCT GACAGACTGT TGGGCACTCA CGAGTTTCCA ACACTAAGAA
     1561 TAAGAAGTCT ACGGCACCCT TTTTGAAACG GCTTTTATAT ATTTACTCCA GCGTAGAAAC
     1621 ACACGCTCTT GGATTCTTTT TCACCCATAC CACTCCCATT CCCGGG
//
```
