# Supplementary material for: Tools for Anopheles gambiae Transgenesis
Source: G3 (Bethesda). 2015 Apr 13;5(6):1151–63. doi: 10.1534/g3.115.016808 (PMC4478545; doi:10.1534/g3.115.016808)
Supplement: Supporting Information [file supp_g3.115.016808_FileS2.pdf]

File S2

DNA sequence of transgenesis vectors

Note: the pDSAx vector series carries kanamycin resistance.

<u>pDSAG (GFP):</u>

```
LOCUS       pDSAG                  5143 bp    DNA     circular
FEATURES             Location/Qualifiers
     misc_feature    complement(3258..3353)
                     /note="attL2"
     misc_feature    complement(3404..3422)
                     /note="M13R"
     misc_feature    1972..2021
                     /note="3xP3 element"
     misc_feature    2061..2068
                     /note="TATA"
                     /note="attR4"
     misc_feature    271..286
                     /note="M13F"
     misc_feature    600..1174
                     /note="LacZ"
     misc_feature    479..599
                     /note="lac promoter"
     misc_feature    590..608
                     /note="M13R"
     misc_feature    625..644
                     /note="T3"
     misc_feature    677..696
                     /note="SK primer"
     misc_feature    complement(731..747)
                     /note="KS primer"
     misc_feature    complement(800..816)
                     /note="M13F"
     misc_feature    complement(771..790)
                     /note="T7"
     misc_feature    1395..1621
                     /note="SV40 term"
     misc_feature    1622..1898
                     /note="attB"
     misc_feature    2965..3194
                     /note="SV40 term"
     misc_feature    2232..2951
                     /note="GFP"
     source          1..5143
                     /dnas_title="pDSAG"
ORIGIN
        1 CTTTCCTGCG TTATCCCCTG ATTCTGTGGA TAACCGTATT ACCGCCTTTG AGTGAGCTGA
       61 TACCGCTCGC CGCAGCCGAA CGACCGAGCG CAGCGAGTCA GTGAGCGAGG AAGCGGAAGA
      121 GCGCCCAATA CGCAAACCGC CTCTCCCCGC GCGTTGGCCG ATTCATTAAT GCAGCTGGCA
      181 CGACAGGTTT CCCGACTGGA AAGCGGGCAG TGAGCGCAAC GCAATTAATA CGCGTACCGC
      241 TAGCATGGAT GTTTTCCCAG TCACGACGTT GTAAAACGAC GGCCAGTCTT AAGCTCGGGC
      301 CCCTACAGGT CACTAATACC ATCTAAGTAG TTGATTCATA GTGACTGGAT ATGTTGTGTT
      361 TTACAGTATT ATGTAGTCTG TTTTTTATGC AAAATCTAAT TTAATATATT GATATTTATA
      421 TCATTTTACG TTTCTCGTTC AACTTTTCTA TACAAAGTTg gtACcggatc cagagaccCG
      481 CAACGCAATT AATGTGAGTT AGCTCACTCA TTAGGCACCC CAGGCTTTAC ACTTTATGCT
      541 TCCGGCTCGT ATGTTGTGTG GAATTGTGAG CGGATAACAA TTTCACACAG GAAACAGCTA
      601 TGACCATGAT TACGCCAAGC GCGCAATTAA CCCTCACTAA AGGGAACAAA AGCTGGAGCT
      661 CCACCGCGGT GGCGGCCGCT CTAGAACTAG TGGATCCCCC GGGCTGCAGG AATTCGATAT
      721 CAAGCTTATC GATACCGTCG ACCTCGAGGG GGGGCCCGGT ACCCAATTCG CCCTATAGTG
      781 AGTCGTATTA CGCGCGCTCA CTGGCCGTCG TTTTACAACG TCGTGACTGG GAAAACCCTG
```

```
 841 GCGTTACCCA ACTTAATCGC CTTGCAGCAC ATCCCCCTTT CGCCAGCTGG CGTAATAGCG
 901 AAGAGGCCCG CACCGATCGC CCTTCCCAAC AGTTGCGCAG CCTGAATGGC GAATGGGACG
 961 CGCCCTGTAG CGGCGCATTA AGCGCGGCGG GTGTGGTGGT TACGCGCAGC GTGACCGCTA
1021 CACTTGCCAG CGCCCTAGCG CCCGCTCCTT TCGCTTTCTT CCCTTCCTTT CTCGCCACGT
1081 TCGCCGGCTT TCCCCGTCAA GCTCTAAATC GGGGGCTCCC TTTAGGGTTC CGATTTAGTG
1141 CTTTACGGCA CCTCGACCCC AAAAAACTTG ATTAGGGTGA TGGTTCACGT AGTGGGCCAT
1201 CGCCCTGATA GACGGTTTTT CGCCCTTTGA CGTTGGAGTC CACGTTCTTT AATAGTGGAC
1261 TCTTGTTCCA AACTGGAACA ACACTCAACC CTATCTCGGT CTATTCTTTT GATTTATAAG
1321 GGATTTTGCC GATTTCGGCC TATTGGTTAA AAAATGAGCT GATTTAACAA AAATTTAACG
1381 CGggtctccg cttCTAGACA TAATCAGCCA TACCACATTT GTAGAGGTTT TACTTGCTTT
1441 AAAAAACCTC CCACACCTCC CCCTGAACCT GAAACATAAA ATGAATGCAA TTGTTGTTGT
1501 TAACTTGTTT ATTGCAGCTT ATAATGGTTA CAAATAAAGC AATAGCATCA CAAATTTCAC
1561 AAATAAAGCA TTTTTTTCA CTGCATTCTA GTTGTGGTTT GTCCAAACTC ATCAATGTAT
1621 CtcgaCGATG TAGGTCACaG TCTCGAAGCC GCGGTGCGGG TGCCAGGGCG TGCCCTTGGG
1681 CTCCCCGGGC GCGTACTCCA CCTCACCCAT CTGGTCCATC ATGATGAACG GGTCGAGGTG
1741 GCGGTAGTTG ATCCCGGCGA ACGCGCGGCG CACCGGGAAG CCCTCGCCCT CGAAACCGCT
1801 GGGCGCGGTG GTCACGGTGA GCACGGGACG TGCGACGGCG TCGGCGGGTG CGGATACGCG
1861 GGGCAGCGTC AGCGGGTTCT CGACGGTCAC GGCGGGCAat tCCTGCAGAC TTCCGGTATC
1921 TCGCGTTTGT TTGATCGCAC GGTTCCCACA ATGGTTAATT CGAGCTCGCC CGGGGATCTA
1981 ATTCAATTAG AGACTAATTC AATTAGAGCT AATTCAATTA GGATCCAAGC TTATCGATTT
2041 CGAACCCTCG ACCGCCGGAG TATAAATAGA GGCGCTTCGT CTACGGAGCG ACAATTCAAT
2101 TCAAACAAGC AAAGTGAACA CGTCGCTAAG CGAAAGCTAA GCAAATAAAC AAGCGCAGCT
2161 GAACAAGCTA AACAATCGGG GTACCGCTAG AGTCGACGGT ACCGCGGGCC CGGGATCCAC
2221 CGGTCGCCAC CATGGTGAGC AAGGGCGAGG AGCTGTTCAC CGGGGTGGTG CCCATCCTGG
2281 TCGAGCTGGA CGGCGACGTA AACGGCCACA AGTTCAGCGT GTCCGGCGAG GGCGAGGGCG
2341 ATGCCACCTA CGGCAAGCTG ACCCTGAAGT TCATCTGCAC CACCGGCAAG CTGCCCGTGC
2401 CCTGGCCCAC CCTCGTGACC ACCCTGACCT ACGGCGTGCA GTGCTTCAGC CGCTACCCCG
2461 ACCACATGAA GCAGCACGAC TTCTTCAAGT CCGCCATGCC CGAAGGCTAC GTCCAGGAGC
2521 GCACCATCTT CTTCAAGGAC GACGGCAACT ACAAGACCCG CGCCGAGGTG AAGTTCGAGG
2581 GCGACACCCT GGTGAACCGC ATCGAGCTGA AGGGCATCGA CTTCAAGGAG GACGGCAACA
2641 TCCTGGGGCA CAAGCTGGAG TACAACTACA ACAGCCACAA CGTCTATATC ATGGCCGACA
2701 AGCAGAAGAA CGGCATCAAG GTGAACTTCA AGATCCGCCA CAACATCGAG GACGGCAGCG
2761 TGCAGCTCGC CGACCACTAC CAGCAGAACA CCCCCATCGG CGACGGCCCC GTGCTGCTGC
2821 CCGACAACCA CTACCTGAGC ACCCAGTCCG CCCTGAGCAA AGACCCCAAC GAGAAGCGCG
2881 ATCACATGGT CCTGCTGGAG TTCGTGACCG CCGCCGGGAT CACTCTCGGC ATGGACGAGC
2941 TGTACAAGTA AAGCGGCCGC GACTctagat caAATCAGCC ATACCACATT TGTAGAGGTT
3001 TTACTTGCTT TAAAAAACCT CCCACACCTC CCCCTGAACC TGAAACATAA AATGAATGCA
3061 ATTGTTGTTG TTAACTTGTT TATTGCAGCT TATAATGGTT ACAAATAAAG CAATAGCATC
3121 ACAAATTTCA CAAATAAAGC ATTTTTTTCA CTGCATTCTA GTTGTGGTTT GTCCAAACTC
3181 ATCAATGTAT CTTAAAGCTT ATCGATACGC GTACGGCGCG CCTAGAGCGG CCGCCACCGC
3241 GGTGGAGCTC GAGTACCCAG CTTTCTTGTA CAAAGTTGGC ATTATAAGAA AGCATTGCTT
3301 ATCAATTTGT TGCAACGAAC AGGTCACTAT CAGTCAAAAT AAAATCATTA TTTGCCATCC
3361 AGCTGCAGGG CGGCCGCGAT ATCCCCTATA GTGAGTCGTA TTACATGGTC ATAGCTGTTT
3421 CCTGGCAGCT CTGGCCCGTG TCTCAAAATC TCTGATGTTA CATTGCACAA GATAAAAATA
3481 TATCATCATG AACAATAAAA CTGTCTGCTT ACATAAACAG TAATACAAGG GGTGTTATGA
3541 GCCATATTCA ACGGGAAACG TCGAGGCCGC GATTAAATTC CAACATGGAT GCTGATTTAT
3601 ATGGGTATAA ATGGGCTCGC GATAATGTCG GGCAATCAGG TGCGACAATC TATCGCTTGT
3661 ATGGGAAGCC CGATGCGCCA GAGTTGTTTC TGAAACATGG CAAAGGTAGC GTTGCCAATG
3721 ATGTTACAGA TGAGATGGTC AGACTAAACT GGCTGACGGA ATTTATGCCT CTTCCGACCA
3781 TCAAGCATTT TATCCGTACT CCTGATGATG CATGGTTACT CACCACTGCG ATCCCCGGAA
3841 AAACAGCATT CCAGGTATTA GAAGAATATC CTGATTCAGG TGAAAATATT GTTGATGCGC
3901 TGGCAGTGTT CCTGCGCCGG TTGCATTCGA TTCCTGTTTG TAATTGTCCT TTTAACAGCG
3961 ATCGCGTATT TCGTCTCGCT CAGGCGCAAT CACGAATGAA TAACGGTTTG GTTGATGCGA
4021 GTGATTTTGA TGACGAGCGT AATGGCTGGC CTGTTGAACA AGTCTGGAAA GAAATGCATA
4081 AACTTTTGCC ATTCTCACCG GATTCAGTCG TCACTCATGG TGATTTCTCA CTTGATAACC
4141 TTATTTTTGA CGAGGGGAAA TTAATAGGTT GTATTGATGT TGGACGAGTC GGAATCGCAG
4201 ACCGATACCA GGATCTTGCC ATCCTATGGA ACTGCCTCGG TGAGTTTTCT CCTTCATTAC
4261 AGAAACGGCT TTTTCAAAAA TATGGTATTG ATAATCCTGA TATGAATAAA TTGCAGTTTC
4321 ATTTGATGCT CGATGAGTTT TTCTAATCAG AATTGGTTAA TTGGTTGTAA CACTGGCAGA
4381 GCATTACGCT GACTTGACGG GACGGCGCAA GCTCATGACC AAAATCCCTT AACGTGAGTT
4441 ACGCGTCGTT CCACTGAGCG TCAGACCCCG TAGAAAAGAT CAAAGGATCT TCTTGAGATC
4501 CTTTTTTTCT GCGCGTAATC TGCTGCTTGC AAACAAAAAA ACCACCGCTA CCAGCGGTGG
4561 TTTGTTTGCC GGATCAAGAG CTACCAACTC TTTTTCCGAA GGTAACTGGC TTCAGCAGAG
4621 CGCAGATACC AAATACTGTT CTTCTAGTGT AGCCGTAGTT AGGCCACCAC TTCAAGAACT
4681 CTGTAGCACC GCCTACATAC CTCGCTCTGC TAATCCTGTT ACCAGTGGCT GCTGCCAGTG
```

```
     4741 GCGATAAGTC GTGTCTTACC GGGTTGGACT CAAGACGATA GTTACCGGAT AAGGCGCAGC
     4801 GGTCGGGCTG AACGGGGGGT TCGTGCACAC AGCCCAGCTT GGAGCGAACG ACCTACACCG
     4861 AACTGAGATA CCTACAGCGT GAGCTATGAG AAAGCGCCAC GCTTCCCGAA GGGAGAAAGG
     4921 CGGACAGGTA TCCGGTAAGC GGCAGGGTCG GAACAGGAGA GCGCACGAGG GAGCTTCCAG
     4981 GGGGAAACGC CTGGTATCTT TATAGTCCTG TCGGGTTTCG CCACCTCTGA CTTGAGCGTC
     5041 GATTTTTGTG ATGCTCGTCA GGGGGGCGGA GCCTATGGAA AAACGCCAGC AACGCGGCCT
     5101 TTTTACGGTT CCTGGCCTTT TGCTGGCCTT TTGCTCACAT GTT
//
```

pDSAT (mTurquoise2):

```
LOCUS       pDSAT      5147 bp    DNA     circular
FEATURES             Location/Qualifiers

     misc_feature    complement(3408..3426)
                     /note="M13R"
     misc_feature    1972..2021
                     /note="3x Pax6 binding sites"
     misc_feature    2061..2068
                     /note="TATA"
     misc_feature    271..286
                     /note="M13F"
     misc_feature    600..1174
                     /note="LacZ"
     misc_feature    479..599
                     /note="lac promoter"
     misc_feature    590..608
                     /note="M13R"
     misc_feature    625..644
                     /note="T3"
     misc_feature    677..696
                     /note="SK primer"
     misc_feature    complement(731..747)
                     /note="KS primer"
     misc_feature    complement(800..816)
                     /note="M13F"
     misc_feature    complement(771..790)
                     /note="T7"
     misc_feature    1395..1621
                     /note="SV40 term"
     misc_feature    1622..1898
                     /note="attB"
     misc_feature    2977..3198
                     /note="SV40 term"
     misc_feature    2232..2948
                     /note="mTurquoise2"
     misc_feature    complement(3452..3470)
                     /note="DONR-RP"

ORIGIN
        1 CTTTCCTGCG TTATCCCCTG ATTCTGTGGA TAACCGTATT ACCGCCTTTG AGTGAGCTGA
       61 TACCGCTCGC CGCAGCCGAA CGACCGAGCG CAGCGAGTCA GTGAGCGAGG AAGCGGAAGA
      121 GCGCCCAATA CGCAAACCGC CTCTCCCCGC GCGTTGGCCG ATTCATTAAT GCAGCTGGCA
      181 CGACAGGTTT CCCGACTGGA AAGCGGGCAG TGAGCGCAAC GCAATTAATA CGCGTACCGC
      241 TAGCATGGAT GTTTTCCCAG TCACGACGTT GTAAAACGAC GGCCAGTCTT AAGCTCGGGC
      301 CCCTACAGGT CACTAATACC ATCTAAGTAG TTGATTCATA GTGACTGGAT ATGTTGTGTT
      361 TTACAGTATT ATGTAGTCTG TTTTTTATGC AAAATCTAAT TTAATATATT GATATTTATA
      421 TCATTTTACG TTTCTCGTTC AACTTTTCTA TACAAAGTTg gtACcggatc cagagaccCG
      481 CAACGCAATT AATGTGAGTT AGCTCACTCA TTAGGCACCC CAGGCTTTAC ACTTTATGCT
      541 TCCGGCTCGT ATGTTGTGTG GAATTGTGAG CGGATAACAA TTTCACACAG GAAACAGCTA
      601 TGACCATGAT TACGCCAAGC GCGCAATTAA CCCTCACTAA AGGGAACAAA AGCTGGAGCT
```

```
 661 CCACCGCGGT GGCGGCCGCT CTAGAACTAG TGGATCCCCC GGGCTGCAGG AATTCGATAT
 721 CAAGCTTATC GATACCGTCG ACCTCGAGGG GGGGCCCGGT ACCCAATTCG CCCTATAGTG
 781 AGTCGTATTA CGCGCGCTCA CTGGCCGTCG TTTTACAACG TCGTGACTGG GAAAACCCTG
 841 GCGTTACCCA ACTTAATCGC CTTGCAGCAC ATCCCCCTTT CGCCAGCTGG CGTAATAGCG
 901 AAGAGGCCCG CACCGATCGC CCTTCCCAAC AGTTGCGCAG CCTGAATGGC GAATGGACG
 961 CGCCCTGTAG CGGCGCATTA AGCGCGGCGG GTGTGGTGGT TACGCGCAGC GTGACCGCTA
1021 CACTTGCCAG CGCCCTAGCG CCCGCTCCTT TCGCTTTCTT CCCTTCCTTT CTCGCCACGT
1081 TCGCCGGCTT TCCCCGTCAA GCTCTAAATC GGGGGCTCCC TTTAGGGTTC CGATTTAGTG
1141 CTTTACGGCA CCTCGACCCC AAAAAACTTG ATTAGGGTGA TGGTTCACGT AGTGGGCCAT
1201 CGCCCTGATA GACGGTTTTT CGCCCTTTGA CGTTGGAGTC CACGTTCTTT AATAGTGGAC
1261 TCTTGTTCCA AACTGGAACA ACACTCAACC CTATCTCGGT CTATTCTTTT GATTTATAAG
1321 GGATTTTGCC GATTTCGGCC TATTGGTTAA AAAATGAGCT GATTTAACAA AAATTTAACG
1381 CGggtctccg cttCTAGACA TAATCAGCCA TACCACATTT GTAGAGGTTT TACTTGCTTT
1441 AAAAAACCTC CCACACCTCC CCCTGAACCT GAAACATAAA ATGAATGCAA TTGTTGTTGT
1501 TAACTTGTTT ATTGCAGCTT ATAATGGTTA CAAATAAAGC AATAGCATCA CAAATTTCAC
1561 AAATAAAGCA TTTTTTTCA CTGCATTCTA GTTGTGGTTT GTCCAAACTC ATCAATGTAT
1621 CtcgaCGATG TAGGTCACaG TCTCGAAGCC GCGGTGCGGG TGCCAGGGCG TGCCCTTGGG
1681 CTCCCCGGGC GCGTACTCCA CCTCACCCAT CTGGTCCATC ATGATGAACG GGTCGAGGTG
1741 GCGGTAGTTG ATCCCGGCGA ACGCGCGGCG CACCGGGAAG CCCTCGCCCT CGAAACCGCT
1801 GGGCGCGGTG GTCACGGTGA GCACGGGACG TGCGACGGCG TCGGCGGGTG CGGATACGCG
1861 GGGCAGCGTC AGCGGGTTCT CGACGGTCAC GGCGGGCAat tCCTGCAGAC TTCCGGTATC
1921 TCGCGTTTGT TTGATCGCAC GGTTCCCACA ATGGTTAATT CGAGCTCGCC CGGGGATCTA
1981 ATTCAATTAG AGACTAATTC AATTAGAGCT AATTCAATTA GGATCCAAGC TTATCGATTT
2041 CGAACCCTCG ACCGCCGGAG TATAAATAGA GGCGCTTCGT CTACGGAGCG ACAATTCAAT
2101 TCAAACAAGC AAAGTGAACA CGTCGCTAAG CGAAAGCTAA GCAAATAAAC AAGCGCAGCT
2161 GAACAAGCTA AACAATCGGG GTACCGCTAG AGTCGACGGT ACCGCGGGCC CGGGATCCAC
2221 CGGTCGCCAC catggtgagc aagggcgagg agctgttcac cggggtggtg cccatcctgg
2281 tcgagctgga cggcgacgta aacggccaca agttcagcgt gtccggcgag ggcgagggcg
2341 atgccaccta cGGCAAGCTG ACCCTGAAGT TCatctgcac caccggcaag ctgcccgtgc
2401 cctggcccac cctcgtgacc accctgtcct ggggcgtgca gtgcttcgcc cgctaccccg
2461 accacatgaa gcagcacgac ttcttcaagt ccgccatgcc cgaaggctac gtccaggagc
2521 gcaccatctt cttcaaggac gacggcaact acaagacccg cgccgaggtg aagttcgagg
2581 gcgacaccct ggtgaaccgc atcgagctga agggcatcga cttcaaggag gacggcaaca
2641 tcctggggca caagctggag tacaactacT tTagcgacaa cgtctatatc accgccgaca
2701 agcagaagaa cggcatcaag gccaacttca agatccgcca caacatcgag gacggcggcg
2761 tgcagctcgc cgaccactac cagcagaaca cccccatcgg cgacggcccc gtgctgctgc
2821 ccgacaacca ctacctgagc acccagtcca agctgagcaa agaccccaac gagaagcgcg
2881 atcacatggt cctgctggag ttcgtgaccg ccgccgggat cactctcggc atggacgagc
2941 tgtacaagtc cggaTGAtag atctgacggg tgatcaAATC AGCCATACCA CATTTGTAGA
3001 GGTTTTACTT GCTTTAAAAA ACCTCCCACA CCTCCCCCTG AACCTGAAAC ATAAAATGAA
3061 TGCAATTGTT GTTGTTAACT TGTTTATTGC AGCTTATAAT GGTTACAAAT AAAGCAATAG
3121 CATCACAAAT TTCACAAATA AAGCATTTTT TTCACTGCAT TCTAGTTGTG GTTTGTCCAA
3181 ACTCATCAAT GTATCTTAAA GCTTATCGAT ACGCGTACGG CGCGCCTAGA GCGGCCGCCA
3241 CCGCGGTGGA GCTCGAGTAC CCAGCTTTCT TGTACAAAGT TGGCATTATA AGAAAGCATT
3301 GCTTATCAAT TTGTTGCAAC GAACAGGTCA CTATCAGTCA AAATAAAATC ATTATTTGCC
3361 ATCCAGCTGC AGGGCGGCCG CGATATCCCC TATAGTGAGT CGTATTACAT GGTCATAGCT
3421 GTTTCCTGGC AGCTCTGGCC CGTGTCTCAA AATCTCTGAT GTTACATTGC ACAAGATAAA
3481 AATATATCAT CATGAACAAT AAAACTGTCT GCTTACATAA ACAGTAATAC AAGGGGTGTT
3541 ATGAGCCATA TTCAACGGGA AACGTCGAGG CCGCGATTAA ATTCCAACAT GGATGCTGAT
3601 TTATATGGGT ATAAATGGGC TCGCGATAAT GTCGGGCAAT CAGGTGCGAC AATCTATCGC
3661 TTGTATGGGA AGCCCGATGC GCCAGAGTTG TTTCTGAAAC ATGGCAAAGG TAGCGTTGCC
3721 AATGATGTTA CAGATGAGAT GGTCAGACTA AACTGGCTGA CGGAATTTAT GCCTCTTCCG
3781 ACCATCAAGC ATTTTATCCG TACTCCTGAT GATGCATGGT TACTCACCAC TGCGATCCCC
3841 GGAAAAACAG CATTCCAGGT ATTAGAAGAA TATCCTGATT CAGGTGAAAA TATTGTTGAT
3901 GCGCTGGCAG TGTTCCTGCG CCGGTTGCAT TCGATTCCTG TTTGTAATTG TCCTTTTAAC
3961 AGCGATCGCG TATTTCGTCT CGCTCAGGCG CAATCACGAA TGAATAACGG TTTGGTTGAT
4021 GCGAGTGATT TTGATGACGA GCGTAATGGC TGGCCTGTTG AACAAGTCTG GAAAGAAATG
4081 CATAAACTTT TGCCATTCTC ACCGGATTCA GTCGTCACTC ATGGTGATTT CTCACTTGAT
4141 AACCTTATTT TTGACGAGGG GAAATTAATA GGTTGTATTG ATGTTGGACG AGTCGGAATC
4201 GCAGACCGAT ACCAGGATCT TGCCATCCTA TGGAACTGCC TCGGTGAGTT TTCTCCTTCA
4261 TTACAGAAAC GGCTTTTTCA AAAATATGGT ATTGATAATC CTGATATGAA TAAATTGCAG
4321 TTTCATTTGA TGCTCGATGA GTTTTTCTAA TCAGAATTGG TTAATTGGTT GTAACACTGG
4381 CAGAGCATTA CGCTGACTTG ACGGGACGGC GCAAGCTCAT GACCAAAATC CCTTAACGTG
4441 AGTTACGCGT CGTTCCACTG AGCGTCAGAC CCCGTAGAAA AGATCAAAGG ATCTTCTTGA
4501 GATCCTTTTT TTCTGCGCGT AATCTGCTGC TTGCAAACAA AAAAACCACC GCTACCAGCG
```

```
       4561 GTGGTTTGTT TGCCGGATCA AGAGCTACCA ACTCTTTTTC CGAAGGTAAC TGGCTTCAGC
       4621 AGAGCGCAGA TACCAAATAC TGTTCTTCTA GTGTAGCCGT AGTTAGGCCA CCACTTCAAG
       4681 AACTCTGTAG CACCGCCTAC ATACCTCGCT CTGCTAATCC TGTTACCAGT GGCTGCTGCC
       4741 AGTGGCGATA AGTCGTGTCT TACCGGGTTG GACTCAAGAC GATAGTTACC GGATAAGGCG
       4801 CAGCGGTCGG GCTGAACGGG GGGTTCGTGC ACACAGCCCA GCTTGGAGCG AACGACCTAC
       4861 ACCGAACTGA GATACCTACA GCGTGAGCTA TGAGAAAGCG CCACGCTTCC CGAAGGGAGA
       4921 AAGGCGGACA GGTATCCGGT AAGCGGCAGG GTCGGAACAG GAGAGCGCAC GAGGGAGCTT
       4981 CCAGGGGGAA ACGCCTGGTA TCTTTATAGT CCTGTCGGGT TTCGCCACCT CTGACTTGAG
       5041 CGTCGATTTT TGTGATGCTC GTCAGGGGGG CGGAGCCTAT GGAAAAACGC CAGCAACGCG
       5101 GCCTTTTTAC GGTTCCTGGC CTTTTGCTGG CCTTTTGCTC ACATGTT
//
```

pDSAY (YFP):

```
LOCUS       pDSAY       5145 bp    DNA     circular
FEATURES             Location/Qualifiers
     misc_feature    complement(3406..3424)
                     /note="M13R"
     misc_feature    271..286
                     /note="M13F"
     misc_feature    600..1174
                     /note="LacZ"
     misc_feature    479..599
                     /note="lac promoter"
     misc_feature    590..608
                     /note="M13R"
     misc_feature    625..644
                     /note="T3"
     misc_feature    677..696
                     /note="SK primer"
     misc_feature    complement(731..747)
                     /note="KS primer"
     misc_feature    complement(800..816)
                     /note="M13F"
     misc_feature    complement(771..790)
                     /note="T7"
     misc_feature    1395..1621
                     /note="SV40 term"
     misc_feature    1622..1898
                     /note="attB"
     misc_feature    1972..2021
                     /note="3x Pax6 binding sites"
     misc_feature    2061..2068
                     /note="TATA"
     gene            2232..2948
                     /note="EYFP (Clontech)"
     misc_feature    2955..3198
                     /note="SV40 term sequences"

ORIGIN
          1 CTTTCCTGCG TTATCCCCTG ATTCTGTGGA TAACCGTATT ACCGCCTTTG AGTGAGCTGA
         61 TACCGCTCGC CGCAGCCGAA CGACCGAGCG CAGCGAGTCA GTGAGCGAGG AAGCGGAAGA
        121 GCGCCCAATA CGCAAACCGC CTCTCCCCGC GCGTTGGCCG ATTCATTAAT GCAGCTGGCA
        181 CGACAGGTTT CCCGACTGGA AAGCGGGCAG TGAGCGCAAC GCAATTAATA CGCGTACCGC
        241 TAGCATGGAT GTTTTCCCAG TCACGACGTT GTAAAACGAC GGCCAGTCTT AAGCTCGGGC
        301 CCCTACAGGT CACTAATACC ATCTAAGTAG TTGATTCATA GTGACTGGAT ATGTTGTGTT
        361 TTACAGTATT ATGTAGTCTG TTTTTTATGC AAAATCTAAT TTAATATATT GATATTTATA
        421 TCATTTTACG TTTCTCGTTC AACTTTTCTA TACAAAGTTg gtACcggatc cagagaccCG
        481 CAACGCAATT AATGTGAGTT AGCTCACTCA TTAGGCACCC CAGGCTTTAC ACTTTATGCT
        541 TCCGGCTCGT ATGTTGTGTG GAATTGTGAG CGGATAACAA TTTCACACAG GAAACAGCTA
        601 TGACCATGAT TACGCCAAGC GCGCAATTAA CCCTCACTAA AGGGAACAAA AGCTGGAGCT
        661 CCACCGCGGT GGCGGCCGCT CTAGAACTAG TGGATCCCCC GGGCTGCAGG AATTCGATAT
        721 CAAGCTTATC GATACCGTCG ACCTCGAGGG GGGGCCCGGT ACCCAATTCG CCCTATAGTG
```

```
 781 AGTCGTATTA CGCGCGCTCA CTGGCCGTCG TTTTACAACG TCGTGACTGG GAAAACCCTG
 841 GCGTTACCCA ACTTAATCGC CTTGCAGCAC ATCCCCCTTT CGCCAGCTGG CGTAATAGCG
 901 AAGAGGCCCG CACCGATCGC CCTTCCCAAC AGTTGCGCAG CCTGAATGGC GAATGGGACG
 961 CGCCCTGTAG CGGCGCATTA AGCGCGGCGG GTGTGGTGGT TACGCGCAGC GTGACCGCTA
1021 CACTTGCCAG CGCCCTAGCG CCCGCTCCTT TCGCTTTCTT CCCTTCCTTT CTCGCCACGT
1081 TCGCCGGCTT TCCCCGTCAA GCTCTAAATC GGGGGCTCCC TTTAGGGTTC CGATTTAGTG
1141 CTTTACGGCA CCTCGACCCC AAAAAACTTG ATTAGGGTGA TGGTTCACGT AGTGGGCCAT
1201 CGCCCTGATA GACGGTTTTT CGCCCTTTGA CGTTGGAGTC CACGTTCTTT AATAGTGGAC
1261 TCTTGTTCCA AACTGGAACA ACACTCAACC CTATCTCGGT CTATTCTTTT GATTTATAAG
1321 GGATTTTGCC GATTTCGGCC TATTGGTTAA AAAATGAGCT GATTTAACAA AAATTTAACG
1381 CGggtctccg cttCTAGACA TAATCAGCCA TACCACATTT GTAGAGGTTT TACTTGCTTT
1441 AAAAAACCTC CCACACCTCC CCCTGAACCT GAAACATAAA ATGAATGCAA TTGTTGTTGT
1501 TAACTTGTTT ATTGCAGCTT ATAATGGTTA CAAATAAAGC AATAGCATCA CAAATTTCAC
1561 AAATAAAGCA TTTTTTTCA CTGCATTCTA GTTGTGGTTT GTCCAAACTC ATCAATGTAT
1621 CtcgaCGATG TAGGTCACaG TCTCGAAGCC GCGGTGCGGG TGCCAGGGCG TGCCCTTGGG
1681 CTCCCCGGGC GCGTACTCCA CCTCACCCAT CTGGTCCATC ATGATGAACG GGTCGAGGTG
1741 GCGGTAGTTG ATCCCGGCGA ACGCGCGGCG CACCGGGAAG CCCTCGCCCT CGAAACCGCT
1801 GGGCGCGGTG GTCACGGTGA GCACGGGACG TGCGACGGCG TCGGCGGGTG CGGATACGCG
1861 GGGCAGCGTC AGCGGGTTCT CGACGGTCAC GGCGGGCAAT TCCTGCAGAC TTCCGGTATC
1921 TCGCGTTTGT TTGATCGCAC GGTTCCCACA ATGGTTAATT CGAGCTCGCC CGGGGATCTA
1981 ATTCAATTAG AGACTAATTC AATTAGAGCT AATTCAATTA GGATCCAAGC TTATCGATTT
2041 CGAACCCTCG ACCGCCGGAG TATAAATAGA GGCGCTTCGT CTACGGAGCG ACAATTCAAT
2101 TCAAACAAGC AAAGTGAACA CGTCGCTAAG CGAAAGCTAA GCAAATAAAC AAGCGCAGCT
2161 GAACAAGCTA AACAATCGGG GTACCGCTAG AGTCGACGGT ACCGCGGGCC CGGGATCCAC
2221 CGGTCGCCAC CATGGTGAGC AAGGGCGAGG AGCTGTTCAC CGGGGTGGTG CCCATCCTGG
2281 TCGAGCTGGA CGGCGACGTA AACGGCCACA AGTTCAGCGT GTCCGGCGAG GGCGAGGGCG
2341 ATGCCACCTA CGGCAAGCTG ACCCTGAAGT TCATCTGCAC CACCGGCAAG CTGCCCGTGC
2401 CCTGGCCCAC CCTCGTGACC ACCTTCGGCT ACGGCCTGCA GTGCTTCGCC CGCTACCCCG
2461 ACCACATGAA GCAGCACGAC TTCTTCAAGT CCGCCATGCC CGAAGGCTAC GTCCAGGAGC
2521 GCACCATCTT CTTCAAGGAC GACGGCAACT ACAAGACCCG CGCCGAGGTG AAGTTCGAGG
2581 GCGACACCCT GGTGAACCGC ATCGAGCTGA AGGGCATCGA CTTCAAGGAG GACGGCAACA
2641 TCCTGGGGCA CAAGCTGGAG TACAACTACA ACAGCCACAA CGTCTATATC ATGGCCGACA
2701 AGCAGAAGAA CGGCATCAAG GTGAACTTCA AGATCCGCCA CAACATCGAG GACGGCAGCG
2761 TGCAGCTCGC CGACCACTAC CAGCAGAACA CCCCCATCGG CGACGGCCCC GTGCTGCTGC
2821 CCGACAACCA CTACCTGAGC TACCAGTCCG CCCTGAGCAA AGACCCCAAC GAGAAGCGCG
2881 ATCACATGGT CCTGCTGGAG TTCGTGACCG CCGCCGGGAT CACTCTCGGC ATGGACGAGC
2941 TGTACAAGTA AAGCGGCCGC GACTCTAGAT CATAATCAGC CATACCACAT TTGTAGAGGT
3001 TTTACTTGCT TTAAAAAACC TCCCACACCT CCCCCTGAAC CTGAAACATA AAATGAATGC
3061 AATTGTTGTT GTTAACTTGT TTATTGCAGC TTATAATGGT TACAAATAAA GCAATAGCAT
3121 CACAAATTTC ACAAATAAAG CATTTTTTTCTT CACTGCATTC TAGTTGTGGT TTGTCCAAAC
3181 TCATCAATGT ATCTTAAAGC TTATCGATAC GCGTACGGCG CGCCTAGAGC GGCCGCCACC
3241 GCGGTGGAGC TCGAGTACCC AGCTTTCTTG TACAAAGTTG GCATTATAAG AAAGCATTGC
3301 TTATCAATTT GTTGCAACGA ACAGGTCACT ATCAGTCAAA ATAAAATCAT TATTTGCCAT
3361 CCAGCTGCAG GGCGGCCGCG ATATCCCCTA TAGTGAGTCG TATTACATGG TCATAGCTGT
3421 TTCCTGGCAG CTCTGGCCCG TGTCTCAAAA TCTCTGATGT TACATTGCAC AAGATAAAAA
3481 TATATCATCA TGAACAATAA AACTGTCTGC TTACATAAAC AGTAATACAA GGGGTGTTAT
3541 GAGCCATATT CAACGGGAAA CGTCGAGGCC GCGATTAAAT TCCAACATGG ATGCTGATTT
3601 ATATGGGTAT AAATGGGCTC GCGATAATGT CGGGCAATCA GGTGCGACAA TCTATCGCTT
3661 GTATGGGAAG CCCGATGCGC CAGAGTTGTT TCTGAAACAT GGCAAAGGTA GCGTTGCCAA
3721 TGATGTTACA GATGAGATGG TCAGACTAAA CTGGCTGACG GAATTTATGC CTCTTCCGAC
3781 CATCAAGCAT TTTATCCGTA CTCCTGATGA TGCATGGTTA CTCACCACTG CGATCCCCGG
3841 AAAAACAGCA TTCCAGGTAT TAGAAGAATA TCCTGATTCA GGTGAAAATA TTGTTGATGC
3901 GCTGGCAGTG TTCCTGCGCC GGTTGCATTC GATTCCTGTT TGTAATTGTC CTTTTAACAG
3961 CGATCGCGTA TTTCGTCTCG CTCAGGCGCA ATCACGAATG AATAACGGTT TGGTTGATGC
4021 GAGTGATTTT GATGACGAGC GTAATGGCTG GCCTGTTGAA CAAGTCTGGA AAGAAATGCA
4081 TAAACTTTTG CCATTCTCAC CGGATTCAGT CGTCACTCAT GGTGATTTCT CACTTGATAA
4141 CCTTATTTTT GACGAGGGGA AATTAATAGG TTGTATTGAT GTTGGACGAG TCGGAATCGC
4201 AGACCGATAC CAGGATCTTG CCATCCTATG GAACTGCCTC GGTGAGTTTT CTCCTTCATT
4261 ACAGAAACGG CTTTTTCAAA AATATGGTAT TGATAATCCT GATATGAATA AATTGCAGTT
4321 TCATTTGATG CTCGATGAGT TTTTCTAATC AGAATTGGTT AATTGGTTGT AACACTGGCA
4381 GAGCATTACG CTGACTTGAC GGGACGGCGC AAGCTCATGA CCAAAATCCC TTAACGTGAG
4441 TTACGCGTCG TTCCACTGAG CGTCAGACCC CGTAGAAAAG ATCAAAGGAT CTTCTTGAGA
4501 TCCTTTTTTT CTGCGCGTAA TCTGCTGCTT GCAAACAAAA AAACCACCGC TACCAGCGGT
4561 GGTTTGTTTG CCGGATCAAG AGCTACCAAC TCTTTTTCCG AAGGTAACTG GCTTCAGCAG
4621 AGCGCAGATA CCAAATACTG TTCTTCTAGT GTAGCCGTAG TTAGGCCACC ACTTCAAGAA
```

```
        4681 CTCTGTAGCA CCGCCTACAT ACCTCGCTCT GCTAATCCTG TTACCAGTGG CTGCTGCCAG
        4741 TGGCGATAAG TCGTGTCTTA CCGGGTTGGA CTCAAGACGA TAGTTACCGG ATAAGGCGCA
        4801 GCGGTCGGGC TGAACGGGGG GTTCGTGCAC ACAGCCCAGC TTGGAGCGAA CGACCTACAC
        4861 CGAACTGAGA TACCTACAGC GTGAGCTATG AGAAAGCGCC ACGCTTCCCG AAGGGAGAAA
        4921 GGCGGACAGG TATCCGGTAA GCGGCAGGGT CGGAACAGGA GAGCGCACGA GGGAGCTTCC
        4981 AGGGGGAAAC GCCTGGTATC TTTATAGTCC TGTCGGGTTT CGCCACCTCT GACTTGAGCG
        5041 TCGATTTTTG TGATGCTCGT CAGGGGGGCG GAGCCTATGG AAAAACGCCA GCAACGCGGC
        5101 CTTTTTACGG TTCCTGGCCT TTTGCTGGCC TTTTGCTCAC ATGTT
//
```

pDSAR (DsRed2):

```
LOCUS       pDSAR       5103 bp    DNA     linear       17-JAN-2013
FEATURES                     Location/Qualifiers
     misc_feature            complement(3364..3382)
                             /note="M13R"
     misc_feature            2231..2912
                             /note="DsRed"
     misc_feature            1972..2021
                             /note="3x Pax6 binding sites"
     misc_feature            2061..2068
                             /note="TATA"
     misc_feature            271..286
                             /note="M13F"
     misc_feature            600..1174
                             /note="LacZ"
     misc_feature            479..599
                             /note="lac promoter"
     misc_feature            590..608
                             /note="M13R"
     misc_feature            625..644
                             /note="T3"
     misc_feature            677..696
                             /note="SK primer"
     misc_feature            complement(731..747)
                             /note="KS primer"
     misc_feature            complement(800..816)
                             /note="M13F"
     misc_feature            complement(771..790)
                             /note="T7"
     misc_feature            1395..1621
                             /note="SV40 term"
     misc_feature            1622..1898
                             /note="attB"
     misc_feature            2914..3154
                             /note="SV40 terminator"
ORIGIN
           1 CTTTCCTGCG TTATCCCCTG ATTCTGTGGA TAACCGTATT ACCGCCTTTG AGTGAGCTGA
          61 TACCGCTCGC CGCAGCCGAA CGACCGAGCG CAGCGAGTCA GTGAGCGAGG AAGCGGAAGA
         121 GCGCCCAATA CGCAAACCGC CTCTCCCCGC GCGTTGGCCG ATTCATTAAT GCAGCTGGCA
         181 CGACAGGTTT CCCGACTGGA AAGCGGGCAG TGAGCGCAAC GCAATTAATA CGCGTACCGC
         241 TAGCATGGAT GTTTTCCCAG TCACGACGTT GTAAAACGAC GGCCAGTCTT AAGCTCGGGC
         301 CCCTACAGGT CACTAATACC ATCTAAGTAG TTGATTCATA GTGACTGGAT ATGTTGTGTT
         361 TTACAGTATT ATGTAGTCTG TTTTTTATGC AAAATCTAAT TTAATATATT GATATTTATA
         421 TCATTTTACG TTTCTCGTTC AACTTTTCTA TACAAAGTTg gtACcggatc cagagacCG
         481 CAACGCAATT AATGTGAGTT AGCTCACTCA TTAGGCACCC CAGGCTTTAC ACTTTATGCT
         541 TCCGGCTCGT ATGTTGTGTG GAATTGTGAG CGGATAACAA TTTCACACAG GAAACAGCTA
         601 TGACCATGAT TACGCCAAGC GCGCAATTAA CCCTCACTAA AGGGAACAAA AGCTGGAGCT
         661 CCACCGCGGT GGCGGCCGCT CTAGAACTAG TGGATCCCCC GGGCTGCAGG AATTCGATAT
         721 CAAGCTTATC GATACCGTCG ACCTCGAGGG GGGGCCCGGT ACCCAATTCG CCCTATAGTG
         781 AGTCGTATTA CGCGCGCTCA CTGGCCGTCG TTTTACAACG TCGTGACTGG GAAAACCCTG
         841 GCGTTACCCA ACTTAATCGC CTTGCAGCAC ATCCCCCTTT CGCCAGCTGG CGTAATAGCG
```

```
 901 AAGAGGCCCG CACCGATCGC CCTTCCCAAC AGTTGCGCAG CCTGAATGGC GAATGGGACG
 961 CGCCCTGTAG CGGCGCATTA AGCGCGGCGG GTGTGGTGGT TACGCGCAGC GTGACCGCTA
1021 CACTTGCCAG CGCCCTAGCG CCCGCTCCTT TCGCTTTCTT CCCTTCCTTT CTCGCCACGT
1081 TCGCCGGCTT TCCCCGTCAA GCTCTAAATC GGGGGCTCCC TTTAGGGTTC CGATTTAGTG
1141 CTTTACGGCA CCTCGACCCC AAAAAACTTG ATTAGGGTGA TGGTTCACGT AGTGGGCCAT
1201 CGCCCTGATA GACGGTTTTT CGCCCTTTGA CGTTGGAGTC CACGTTCTTT AATAGTGGAC
1261 TCTTGTTCCA AACTGGAACA ACACTCAACC CTATCTCGGT CTATTCTTTT GATTTATAAG
1321 GGATTTTGCC GATTTCGGCC TATTGGTTAA AAAATGAGCT GATTTAACAA AAATTTAACG
1381 CGggtctccg cttCTAGACA TAATCAGCCA TACCACATTT GTAGAGGTTT TACTTGCTTT
1441 AAAAAACCTC CCACACCTCC CCCTGAACCT GAAACATAAA ATGAATGCAA TTGTTGTTGT
1501 TAACTTGTTT ATTGCAGCTT ATAATGGTTA CAAATAAAGC AATAGCATCA CAAATTTCAC
1561 AAATAAAGCA TTTTTTTCA CTGCATTCTA GTTGTGGTTT GTCCAAACTC ATCAATGTAT
1621 CtcgaCGATG TAGGTCACaG TCTCGAAGCC GCGGTGCGGG TGCCAGGGCG TGCCCTTGGG
1681 CTCCCCGGGC GCGTACTCCA CCTCACCCAT CTGGTCCATC ATGATGAACG GGTCGAGGTG
1741 GCGGTAGTTG ATCCCGGCGA ACGCGCGGCG CACCGGGAAG CCCTCGCCCT CGAAACCGCT
1801 GGGCGCGGTC GTCACGGTGA GCACGGGACG TGCGACGGCG TCGGCGGGTG CGGATACGCG
1861 GGGCAGCGTC AGCGGGTTCT CGACGGTCAC GGCGGGCAat tCCTGCAGAC TTCCGGTATC
1921 TCGCGTTTGT TTGATCGCAC GGTTCCCACA ATGGTTAATT CGAGCTCGCC CGGGGATCTA
1981 ATTCAATTAG AGACTAATTC AATTAGAGCT AATTCAATTA GGATCCAAGC TTATCGATTT
2041 CGAACCCTCG ACCGCCGGAG TATAAATAGA GGCGCTTCGT CTACGGAGCG ACAATTCAAT
2101 TCAAACAAGC AAAGTGAACA CGTCGCTAAG CGAAAGCTAA GCAAATAAAC AAGCGCAGCT
2161 GAACAAGCTA AACAATCGGG GTACCGCTAG AGTCGACGGT ACCGCGGGCC CGGGATCCAC
2221 CGGTCGCCAC CATGGTGCGC TCCTCCAAGA ACGTCATCAA GGAGTTCATG CGCTTCAAGG
2281 TGCGCATGGA GGGCACCGTG AACGGCCACG AGTTCGAGAT CGAGGGCGAG GGCGAGGGCC
2341 GCCCCTACGA GGGCCACAAC ACCGTGAAGC TGAAGGTGAC CAAGGGCGGC CCCCTGCCCT
2401 TCGCCTGGGA CATCCTGTCC CCCCAGTTCC AGTACGGCTC CAAGGTGTAC GTGAAGCACC
2461 CCGCCGACAT CCCCGACTAC AAGAAGCTGT CCTTCCCCGA GGGCTTCAAG TGGGAGCGCG
2521 TGATGAACTT CGAGGACGGC GGCGTGGTGA CCGTGACCCA GGACTCCTCC CTGCAGGACG
2581 GCTGCTTCAT CTACAAGGTG AAGTTCATCG GCGTGAACTT CCCCTCCGAC GGCCCCGTAA
2641 TGCAGAAGAA GACCATGGGC TGGGAGGCCT CCACCGAGCG CCTGTACCCC CGCGACGGCG
2701 TGCTGAAGGG CGAGATCCAC AAGGCCCTGA AGCTGAAGGA CGGCGGCCAC TACCTGGTGG
2761 AGTTCAAGTC CATCTACATG GCCAAGAAGC CCGTGCAGCT GCCCGGCTAC TACTACGTGG
2821 ACTCCAAGCT GGACATCACC TCCCACAACG AGGACTACAC CATCGTGGAg CAGTACGAGC
2881 GCACCGAGGG CCGCCACCAC CTGTTCCTGT AGCGGCCGCG ACTCTAGATC ATAATCAGCC
2941 ATACCACATT TGTAGAGGTT TTACTTGCTT TAAAAAACCT CCCACACCTC CCCCTGAACC
3001 TGAAACATAA AATGAATGCA ATTGTTGTTG TTAACTTGTT TATTGCAGCT TATAATGGTT
3061 ACAAATAAAG CAATAGCATC ACAAATTTCA CAAATAAAGC ATTTTTTTCA CTGCATTCTA
3121 GTTGTGGTTT GTCCAAACTC ATCAATGTAT CTTAAAGCTT ATCGATACGC GTACGGCGCG
3181 CCTAGAGCGG CCGCCACCGC GGTGGAGCTC GAGTACCCAG CTTTCTTGTA CAAAGTTGGC
3241 ATTATAAGAA AGCATTGCTT ATCAATTTGT TGCAACGAAC AGGTCACTAT CAGTCAAAAT
3301 AAAATCATTA TTTGCCATCC AGCTGCAGGG CGGCCGCGAT ATCCCCTATA GTGAGTCGTA
3361 TTACATGGTC ATAGCTGTTT CCTGGCAGCT CTGGCCCGTG TCTCAAAATC TCTGATGTTA
3421 CATTGCACAA GATAAAAATA TATCATCATG AACAATAAAA CTGTCTGCTT ACATAAACAG
3481 TAATACAAGG GGTGTTATGA GCCATATTCA ACGGGAAACG TCGAGGCCGC GATTAAATTC
3541 CAACATGGAT GCTGATTTAT ATGGGTATAA ATGGGCTCGC GATAATGTCG GGCAATCAGG
3601 TGCGACAATC TATCGCTTGT ATGGGAAGCC CGATGCGCCA GAGTTGTTTC TGAAACATGG
3661 CAAAGGTAGC GTTGCCAATG ATGTTACAGA TGAGATGGTC AGACTAAACT GGCTGACGGA
3721 ATTTATGCCT CTTCCGACCA TCAAGCATTT TATCCGTACT CCTGATGATG CATGGTTACT
3781 CACCACTGCG ATCCCCGGAA AAACAGCATT CCAGGTATTA GAAGAATATC CTGATTCAGG
3841 TGAAAATATT GTTGATGCGC TGGCAGTGTT CCTGCGCCGG TTGCATTCGA TTCCTGTTTG
3901 TAATTGTCCT TTTAACAGCG ATCGCGTATT TCGTCTCGCT CAGGCGCAAT CACGAATGAA
3961 TAACGGTTTG GTTGATGCGA GTGATTTTGA TGACGAGCGT AATGGCTGGC CTGTTGAACA
4021 AGTCTGGAAA GAAATGCATA AACTTTTGCC ATTCTCACCG GATTCAGTCG TCACTCATGG
4081 TGATTTCTCA CTTGATAACC TTATTTTTGA CGAGGGGAAA TTAATAGGTT GTATTGATGT
4141 TGGACGAGTC GGAATCGCAG ACCGATACCA GGATCTTGCC ATCCTATGGA ACTGCCTCGG
4201 TGAGTTTTCT CCTTCATTAC AGAAACGGCT TTTTCAAAAA TATGGTATTG ATAATCCTGA
4261 TATGAATAAA TTGCAGTTTC ATTTGATGCT CGATGAGTTT TTCTAATCAG AATTGGTTAA
4321 TTGGTTGTAA CACTGGCAGA GCATTACGCT GACTTGACGG GACGGCGCAA GCTCATGACC
4381 AAAATCCCTT AACGTGAGTT ACGCGTCGTT CCACTGAGCG TCAGACCCCG TAGAAAAGAT
4441 CAAAGGATCT TCTTGAGATC CTTTTTTTCT GCGCGTAATC TGCTGCTTGC AAACAAAAAA
4501 ACCACCGCTA CCAGCGGTGG TTTGTTTGCC GGATCAAGAG CTACCAACTC TTTTTCCGAA
4561 GGTAACTGGC TTCAGCAGAG CGCAGATACC AAATACTGTT CTTCTAGTGT AGCCGTAGTT
4621 AGGCCACCAC TTCAAGAACT CTGTAGCACC GCCTACATAC CTCGCTCTGC TAATCCTGTT
4681 ACCAGTGGCT GCTGCCAGTG GCGATAAGTC GTGTCTTACC GGGTTGGACT CAAGACGATA
4741 GTTACCGGAT AAGGCGCAGC GGTCGGGCTG AACGGGGGGT TCGTGCACAC AGCCCAGCTT
```

```
        4801 GGAGCGAACG ACCTACACCG AACTGAGATA CCTACAGCGT GAGCTATGAG AAAGCGCCAC
        4861 GCTTCCCGAA GGGAGAAAGG CGGACAGGTA TCCGGTAAGC GGCAGGGTCG GAACAGGAGA
        4921 GCGCACGAGG GAGCTTCCAG GGGGAAACGC CTGGTATCTT TATAGTCCTG TCGGGTTTCG
        4981 CCACCTCTGA CTTGAGCGTC GATTTTTGTG ATGCTCGTCA GGGGGGCGGA GCCTATGGAA
        5041 AAACGCCAGC AACGCGGCCT TTTTACGGTT CCTGGCCTTT TGCTGGCCTT TTGCTCACAT
        5101 GTT
//
```

<u>pDSAP (puromycin resistance):</u>

```
LOCUS       pDSAP                   5456 bp    DNA     circular
FEATURES             Location/Qualifiers
     misc_feature    complement(3717..3735)
                     /note="M13R"
     misc_feature    1972..2021
                     /note="3x Pax6 binding sites"
     misc_feature    271..286
                     /note="M13F"
     misc_feature    600..1174
                     /note="LacZ"
     misc_feature    479..599
                     /note="lac promoter"
     misc_feature    590..608
                     /note="M13R"
     misc_feature    625..644
                     /note="T3"
     misc_feature    677..696
                     /note="SK primer"
     misc_feature    complement(731..747)
                     /note="KS primer"
     misc_feature    complement(800..816)
                     /note="M13F"
     misc_feature    complement(771..790)
                     /note="T7"
     misc_feature    1395..1621
                     /note="SV40 terminator"
     misc_feature    1622..1898
                     /note="attB"
     misc_feature    2114..2762
                     /note="OpIE2 promoter"
     misc_feature    2763..3364
                     /note="puromycin resistance ORF"
     misc_feature    3364..3523
                     /note="SV40 terminator"
     misc_feature    complement(3761..3779)
                     /note="pDONR-RP"

ORIGIN
           1 CTTTCCTGCG TTATCCCCTG ATTCTGTGGA TAACCGTATT ACCGCCTTTG AGTGAGCTGA
          61 TACCGCTCGC CGCAGCCGAA CGACCGAGCG CAGCGAGTCA GTGAGCGAGG AAGCGGAAGA
         121 GCGCCCAATA CGCAAACCGC CTCTCCCCGC GCGTTGGCCG ATTCATTAAT GCAGCTGGCA
         181 CGACAGGTTT CCCGACTGGA AAGCGGGCAG TGAGCGCAAC GCAATTAATA CGCGTACCGC
         241 TAGCATGGAT GTTTTCCCAG TCACGACGTT GTAAAACGAC GGCCAGTCTT AAGCTCGGGC
         301 CCCTACAGGT CACTAATACC ATCTAAGTAG TTGATTCATA GTGACTGGAT ATGTTGTGTT
         361 TTACAGTATT ATGTAGTCTG TTTTTTATGC AAAATCTAAT TTAATATATT GATATTTATA
         421 TCATTTTACG TTTCTCGTTC AACTTTTCTA TACAAAGTTg gtACcggatc cagagaccCG
         481 CAACGCAATT AATGTGAGTT AGCTCACTCA TTAGGCACCC CAGGCTTTAC ACTTTATGCT
         541 TCCGGCTCGT ATGTTGTGTG GAATTGTGAG CGGATAACAA TTTCACACAG GAAACAGCTA
         601 TGACCATGAT TACGCCAAGC GCGCAATTAA CCCTCACTAA AGGGAACAAA AGCTGGAGCT
         661 CCACCGCGGT GGCGGCCGCT CTAGAACTAG TGGATCCCCC GGGCTGCAGG AATTCGATAT
         721 CAAGCTTATC GATACCGTCG ACCTCGAGGG GGGGCCCGGT ACCCAATTCG CCCTATAGTG
         781 AGTCGTATTA CGCGCGCTCA CTGGCCGTCG TTTTACAACG TCGTGACTGG GAAAACCCTG
```

```
 841 GCGTTACCCA ACTTAATCGC CTTGCAGCAC ATCCCCCTTT CGCCAGCTGG CGTAATAGCG
 901 AAGAGGCCCG CACCGATCGC CCTTCCCAAC AGTTGCGCAG CCTGAATGGC GAATGGACG
 961 CGCCCTGTAG CGGCGCATTA AGCGCGGCGG GTGTGGTGGT TACGCGCAGC GTGACCGCTA
1021 CACTTGCCAG CGCCCTAGCG CCCGCTCCTT TCGCTTTCTT CCCTTCCTTT CTCGCCACGT
1081 TCGCCGGCTT TCCCCGTCAA GCTCTAAATC GGGGGCTCCC TTTAGGGTTC CGATTTAGTG
1141 CTTTACGGCA CCTCGACCCC AAAAAACTTG ATTAGGGTGA TGGTTCACGT AGTGGGCCAT
1201 CGCCCTGATA GACGGTTTTT CGCCCTTTGA CGTTGGAGTC CACGTTCTTT AATAGTGGAC
1261 TCTTGTTCCA AACTGGAACA ACACTCAACC CTATCTCGGT CTATTCTTTT GATTTATAAG
1321 GGATTTTGCC GATTTCGGCC TATTGGTTAA AAAATGAGCT GATTTAACAA AAATTTAACG
1381 CGggtctccg cttCTAGACA TAATCAGCCA TACCACATTT GTAGAGGTTT TACTTGCTTT
1441 AAAAAACCTC CCACACCTCC CCCTGAACCT GAAACATAAA ATGAATGCAA TTGTTGTTGT
1501 TAACTTGTTT ATTGCAGCTT ATAATGGTTA CAAATAAAGC AATAGCATCA CAAATTTCAC
1561 AAATAAAGCA TTTTTTTCA CTGCATTCTA GTTGTGGTTT GTCCAAACTC ATCAATGTAT
1621 CtcgaCGATG TAGGTCACaG TCTCGAAGCC GCGGTGCGGG TGCCAGGGCG TGCCCTTGGG
1681 CTCCCCGGGC GCGTACTCCA CCTCACCCAT CTGGTCCATC ATGATGAACG GGTCGAGGTG
1741 GCGGTAGTTG ATCCCGGCGA ACGCGCGGCG CACCGGGAAG CCCTCGCCCT CGAAACCGCT
1801 GGGCGCGGTG GTCACGGTGA GCACGGGACG TGCGACGGCG TCGGCGGGTG CGGATACGCG
1861 GGGCAGCGTC AGCGGGTTCT CGACGGTCAC GGCGGGCAat tCCTGCAGAC TTCCGGTATC
1921 TCGCGTTTGT TTGATCGCAC GGTTCCCACA ATGGTTAATT CGAGCTCGCC CGGGGATCTA
1981 ATTCAATTAG AGACTAATTC AATTAGAGCT AATTCAATTA GGATCCAAGC TTATCGATTT
2041 CGATAAGCTT GATATCGAAT TCCTGCAGCC CGGGGGATCC Actagtggat cccccgggct
2101 gcaggaattc gatCATGATG ATAAACAATG TATGGTGCTA ATGTTGCTTC AACAACAATT
2161 CTGTTGAACT GTGTTTTCAT GTTTGCCAAC AAGCACCTTT ATACTCGGTG GCCTCCCCAC
2221 CACCAACTTT TTTGCACTGC AAAAAAACAC GCTTTTGCAC GCGGGCCCAT ACATAGTACA
2281 AACTCTACGT TTCGTAGACT ATTTTACATA AATAGTCTAC ACCGTTGTAT ACGCTCCAAA
2341 TACACTACCA CACATTGAAC CTTTTTGCAG TGCAAAAAAG TACGTGTCGG CAGTCACGTA
2401 GGCCGGCCTT ATCGGGTCGC GTCCTGTCAC GTACGAATCA CATTATCGGA CCGGACGAGT
2461 GTTGTCTTAT CGTGACAGGA CGCCAGCTTC CTGTGTTGCT AACCGCAGCC GGACGCAACT
2521 CCTTATCGGA ACAGGACGCG CCTCCATATC AGCCGCGCGT TATCTCATGC GCGTGACCGG
2581 ACACGAGGCG CCCGTCCCGC TTATCGCGCC TATAAATACA GCCCGCAACG ATCTGGTAAA
2641 CACAGTTGAA CAGCATCTGT TCGAATTAAT TCgGGATCTg ccgggCTGCA GCACGTGTTG
2701 ACAATTAATC ATCGGCATAG TATATCGGCA TAGTATAATA CGACTCACTA TAGGAGGGCC
2761 ACcATGaccg agtacaagcc cacggtgcgc ctcgccaccc gcgacgacgt ccccAgggcc
2821 gtacgcaccc tcgccgccgc gttcgccgac taccccgcca cgcgccacac cgtcgaTccg
2881 gaccgccaca tcgagcgggt caccgagctg caagaactct tcctcacgcg cgtcgggctc
2941 gacatcggca aggtgtgggt cgcggacgac ggcgccgcgg tggcggtctg gaccacgccg
3001 gagagcgtcg aagcgggggc ggtgttcgcc gagatcggcc cgcgcatggc cgagttgagc
3061 ggttcccggc tggccgcgca gcaacagatg gaaggcctcc tggcgccgca ccggcccaag
3121 gagcccgcgt ggttcctggc caccgtcggc gtctcgcccg accaccaggg caagggtctg
3181 ggcagcgccg tcgtgctccc cggagtggag gcggccgagc gcgccggggt gcccgccttc
3241 ctggagacct ccgcgccccg caacctcccc ttctacgagc ggctcggctt caccgtcacc
3301 gccgacgtcg agGtgcccga aggaccgcgc acctggtgca tgacccgcaa gcccggtgcc
3361 tgaCTCGACC TCGAAACTTG TTTATTGCAG CTTATAATGG TTACAAATAA AGCAATAGCA
3421 TCACAAATTT CACAAATAAA GCATTTTTTT CACTGCATTC TAGTTGTGGT TTGTCCAAAC
3481 TCATCAATGT ATCTTATCAT GTCTggatca tgggggggc ccgGTACGGC GCGCCTAGAG
3541 CGGCCGCCAC CGCGGTGGAG CTCGAGTACC CAGCTTTCTT GTACAAAGTT GGCATTATAA
3601 GAAAGCATTG CTTATCAATT TGTTGCAACG AACAGGTCAC TATCAGTCAA AATAAAATCA
3661 TTATTTGCCA TCCAGCTGCA GGGCGGCCGC GATATCCCCT ATAGTGAGTC GTATTACATG
3721 GTCATAGCTG TTTCCTGGCA GCTCTGGCCC GTGTCTCAAA ATCTCTGATG TTACATTGCA
3781 CAAGATAAAA ATATATCATC ATGAACAATA AAACTGTCTG CTTACATAAA CAGTAATACA
3841 AGGGGTGTTA TGAGCCATAT TCAACGGGAA ACGTCGAGGC CGCGATTAAA TTCCAACATG
3901 GATGCTGATT TATATGGGTA TAAATGGGCT CGCGATAATG TCGGGCAATC AGGTGCGACA
3961 ATCTATCGCT TGTATGGGAA GCCCGATGCG CCAGAGTTGT TTCTGAAACA TGGCAAAGGT
4021 AGCGTTGCCA ATGATGTTAC AGATGAGATG GTCAGACTAA ACTGGCTGAC GGAATTTATG
4081 CCTCTTCCGA CCATCAAGCA TTTTATCCGT ACTCCTGATG ATGCATGGTT ACTCACCACT
4141 GCGATCCCCG GAAAAACAGC ATTCCAGGTA TTAGAAGAAT ATCCTGATTC AGGTGAAAAT
4201 ATTGTTGATG CGCTGGCAGT GTTCCTGCGC CGGTTGCATT CGATTCCTGT TTGTAATTGT
4261 CCTTTTAACA GCGATCGCGT ATTTCGTCTC GCTCAGGCGC AATCACGAAT GAATAACGGT
4321 TTGGTTGATG CGAGTGATTT TGATGACGAG CGTAATGGCT GGCCTGTTGA ACAAGTCTGG
4381 AAAGAAATGC ATAAACTTTT GCCATTCTCA CCGGATTCAG TCGTCACTCA TGGTGATTTC
4441 TCACTTGATA ACCTTATTTT TGACGAGGGG AAATTAATAG GTTGTATTGA TGTTGGACGA
4501 GTCGGAATCG CAGACCGATA CCAGGATCTT GCCATCCTAT GGAACTGCCT CGGTGAGTTT
4561 TCTCCTTCAT TACAGAAACG GCTTTTTCAA AAATATGGTA TTGATAATCC TGATATGAAT
4621 AAATTGCAGT TTCATTTGAT GCTCGATGAG TTTTTCTAAT CAGAATTGGT TAATTGGTTG
4681 TAACACTGGC AGAGCATTAC GCTGACTTGA CGGGACGGCG CAAGCTCATG ACCAAAATCC
```

```
        4741 CTTAACGTGA GTTACGCGTC GTTCCACTGA GCGTCAGACC CCGTAGAAAA GATCAAAGGA
        4801 TCTTCTTGAG ATCCTTTTTT TCTGCGCGTA ATCTGCTGCT TGCAAACAAA AAAACCACCG
        4861 CTACCAGCGG TGGTTTGTTT GCCGGATCAA GAGCTACCAA CTCTTTTTCC GAAGGTAACT
        4921 GGCTTCAGCA GAGCGCAGAT ACCAAATACT GTTCTTCTAG TGTAGCCGTA GTTAGGCCAC
        4981 CACTTCAAGA ACTCTGTAGC ACCGCCTACA TACCTCGCTC TGCTAATCCT GTTACCAGTG
        5041 GCTGCTGCCA GTGGCGATAA GTCGTGTCTT ACCGGGTTGG ACTCAAGACG ATAGTTACCG
        5101 GATAAGGCGC AGCGGTCGGG CTGAACGGGG GGTTCGTGCA CACAGCCCAG CTTGGAGCGA
        5161 ACGACCTACA CCGAACTGAG ATACCTACAG CGTGAGCTAT GAGAAAGCGC CACGCTTCCC
        5221 GAAGGGAGAA AGGCGGACAG GTATCCGGTA AGCGGCAGGG TCGGAACAGG AGAGCGCACG
        5281 AGGGAGCTTC CAGGGGGAAA CGCCTGGTAT CTTTATAGTC CTGTCGGGTT TCGCCACCTC
        5341 TGACTTGAGC GTCGATTTTT GTGATGCTCG TCAGGGGGGC GGAGCCTATG GAAAAACGCC
        5401 AGCAACGCGG CCTTTTTACG GTTCCTGGCC TTTTGCTGGC CTTTTGCTCA CATGTT
//
```

pDSAYN (YFPnls):

```
LOCUS       pDSAYnls                5187 bp    DNA     circular
FEATURES             Location/Qualifiers
     misc_feature    complement(3448..3466)
                     /note="M13R"
     misc_feature    1972..2021
                     /note="3x Pax6 binding sites"
     misc_feature    2061..2068
                     /note="TATA"
     misc_feature    271..286
                     /note="M13F"
     misc_feature    600..1174
                     /note="LacZ"
     misc_feature    479..599
                     /note="lac promoter"
     misc_feature    590..608
                     /note="M13R"
     misc_feature    625..644
                     /note="T3"
     misc_feature    677..696
                     /note="SK primer"
     misc_feature    complement(731..747)
                     /note="KS primer"
     misc_feature    complement(800..816)
                     /note="M13F"
     misc_feature    complement(771..790)
                     /note="T7"
     misc_feature    1395..1621
                     /note="SV40 terminator"
     misc_feature    1622..1898
                     /note="attB"
     CDS             2232..2951
                     /dnas_title="YFP"
                     /vntifkey="4"
                     /label=YFP
     3'UTR           3007..3234
                     /dnas_title="SV40 3'UTR"
                     /vntifkey="50"
                     /label=SV40 3'UTR
     sig_peptide     2949..2987
                     /dnas_title="NLS"
                     /vntifkey="94"
                     /label=NLS
     source          1..5187
                     /dnas_title="pDSAYnls"

ORIGIN
```

```
   1 CTTTCCTGCG TTATCCCCTG ATTCTGTGGA TAACCGTATT ACCGCCTTTG AGTGAGCTGA
  61 TACCGCTCGC CGCAGCCGAA CGACCGAGCG CAGCGAGTCA GTGAGCGAGG AAGCGGAAGA
 121 GCGCCCAATA CGCAAACCGC CTCTCCCCGC GCGTTGGCCG ATTCATTAAT GCAGCTGGCA
 181 CGACAGGTTT CCCGACTGGA AAGCGGGCAG TGAGCGCAAC GCAATTAATA CGCGTACCGC
 241 TAGCATGGAT GTTTTCCCAG TCACGACGTT GTAAAACGAC GGCCAGTCTT AAGCTCGGGC
 301 CCCTACAGGT CACTAATACC ATCTAAGTAG TTGATTCATA GTGACTGGAT ATGTTGTGTT
 361 TTACAGTATT ATGTAGTCTG TTTTTTATGC AAAATCTAAT TTAATATATT GATATTTATA
 421 TCATTTTACG TTTCTCGTTC AACTTTTCTA TACAAAGTTg gtACcggatc cagagaccCG
 481 CAACGCAATT AATGTGAGTT AGCTCACTCA TTAGGCACCC CAGGCTTTAC ACTTTATGCT
 541 TCCGGCTCGT ATGTTGTGTG GAATTGTGAG CGGATAACAA TTTCACACAG GAAACAGCTA
 601 TGACCATGAT TACGCCAAGC GCGCAATTAA CCCTCACTAA AGGGAACAAA AGCTGGAGCT
 661 CCACCGCGGT GGCGGCCGCT CTAGAACTAG TGGATCCCCC GGGCTGCAGG AATTCGATAT
 721 CAAGCTTATC GATACCGTCG ACCTCGAGGG GGGGCCCGGT ACCCAATTCG CCCTATAGTG
 781 AGTCGTATTA CGCGCGCTCA CTGGCCGTCG TTTTACAACG TCGTGACTGG GAAAACCCTG
 841 GCGTTACCCA ACTTAATCGC CTTGCAGCAC ATCCCCCTTT CGCCAGCTGG CGTAATAGCG
 901 AAGAGGCCCG CACCGATCGC CCTTCCCAAC AGTTGCGCAG CCTGAATGGC GAATGGACG
 961 CGCCCTGTAG CGGCGCATTA AGCGCGGCGG GTGTGGTGGT TACGCGCAGC GTGACCGCTA
1021 CACTTGCCAG CGCCCTAGCG CCCGCTCCTT TCGCTTTCTT CCCTTCCTTT CTCGCCACGT
1081 TCGCCGGCTT TCCCCGTCAA GCTCTAAATC GGGGGCTCCC TTTAGGGTTC CGATTTAGTG
1141 CTTTACGGCA CCTCGACCCC AAAAAACTTG ATTAGGGTGA TGGTTCACGT AGTGGGCCAT
1201 CGCCCTGATA GACGGTTTTT CGCCCTTTGA CGTTGGAGTC CACGTTCTTT AATAGTGGAC
1261 TCTTGTTCCA AACTGGAACA ACACTCAACC CTATCTCGGT CTATTCTTTT GATTTATAAG
1321 GGATTTTGCC GATTTCGGCC TATTGGTTAA AAAATGAGCT GATTTAACAA AAATTTAACG
1381 CGggtctccg cttCTAGACA TAATCAGCCA TACCACATTT GTAGAGGTTT TACTTGCTTT
1441 AAAAAACCTC CCACACCTCC CCCTGAACCT GAAACATAAA ATGAATGCAA TTGTTGTTGT
1501 TAACTTGTTT ATTGCAGCTT ATAATGGTTA CAAATAAAGC AATAGCATCA CAAATTTCAC
1561 AAATAAAGCA TTTTTCTTCA CTGCATTCTA GTTGTGGTTT GTCCAAACTC ATCAATGTAT
1621 CtcgaCGATG TAGGTCACaG TCTCGAAGCC GCGGTGCGGG TGCCAGGGCG TGCCCTTGGG
1681 CTCCCCGGGC GCGTACTCCA CCTCACCCAT CTGGTCCATC ATGATGAACG GGTCGAGGTG
1741 GCGGTAGTTG ATCCCGGCGA ACGCGCGGCG CACCGGGAAG CCCTCGCCCT CGAAACCGCT
1801 GGGCGCGGTG GTCACGGTGA GCACGGGACG TGCGACGGCG TCGGCGGGTG CGGATACGCG
1861 GGGCAGCGTC AGCGGGTTCT CGACGGTCAC GGCGGGCAat tCCTGCAGAC TTCCGGTATC
1921 TCGCGTTTGT TTGATCGCAC GGTTCCCACA ATGGTTAATT CGAGCTCGCC CGGGGATCTA
1981 ATTCAATTAG AGACTAATTC AATTAGAGCT AATTCAATTA GGATCCAAGC TTATCGATTT
2041 CGAACCCTCG ACCGCCGGAG TATAAATAGA GGCGCTTCGT CTACGGAGCG ACAATTCAAT
2101 TCAAACAAGC AAAGTGAACA CGTCGCTAAG CGAAAGCTAA GCAAATAAAC AAGCGCAGCT
2161 GAACAAGCTA AACAATCGGG GTACCGCTAG AGTCGACGGT ACCGCGGGCC CGGGATCCAc
2221 cggtcgccac catggtgagc aagggcgagg agctgttcac cggggtggtg cccatcctgg
2281 tcgagctgga cggcgacgta aacggccaca agttcagcgt gtccggcgag ggcgagggcg
2341 atgccaccta cggcaagctg accctgaagt tcatctgcac caccggcaag ctgcccgtgc
2401 cctggcccac cctcgtgacc accttcggct acggcctgca gtgcttcgcc cgctaccccg
2461 accacatgaa gcagcacgac ttcttcaagt ccgccatgcc cgaaggctac gtccaggagc
2521 gcaccatctt cttcaaggac gacggcaact acaagacccg cgccgaggtg aagttcgagg
2581 gcgacaccct ggtgaaccgc atcgagctga agggcatcga cttcaaggag gacggcaaca
2641 tcctggggca caagctggag tacaactaca acagccacaa cgtctatatc atggccgaca
2701 agcagaagaa cggcatcaag gtgaacttca agatccgcca caacatcgag gacggcagcg
2761 tgcagctcgc cgaccactac cagcagaaca cccccatcgg cgacggcccc gtgctgctgc
2821 ccgacaacca ctacctgagc taccagtccg ccctgagcaa agaccccaac gagaagcgcg
2881 atcacatggt cctgctggag ttcgtgaccg ccgccgggat cactctcggc atggacgagc
2941 tgtacaagag atctcgaccc aagaaaaagc ggaaggtgga ggacccgtaa agcggccgcg
3001 actctagatc ataatcagcc ataccacatt tgtagaggtt ttacttgctt taaaaaacct
3061 cccacacctc cccctgaacc tgaaacataa aatgaatgca attgttgttg ttaacttgtt
3121 tattgcagct tataatggtt acaaataaag caatagcatc acaaatttca caaataaagc
3181 atttttttca ctgcattcta gttgtggttt gtccaaactc atcaatgtat cttaaagctt
3241 atcgatacgc GTACGtacgG CGCGCCTAGA GCGGCCGCCA CCGCGGTGGA GCTCGAGTAC
3301 CCAGCTTTCT TGTACAAAGT TGGCATTATA AGAAAGCATT GCTTATCAAT TTGTTGCAAC
3361 GAACAGGTCA CTATCAGTCA AAATAAAATC ATTATTTGCC ATCCAGCTGC AGGGCGGCCG
3421 CGATATCCCC TATAGTGAGT CGTATTACAT GGTCATAGCT GTTTCCTGGC AGCTCTGGCC
3481 CGTGTCTCAA AATCTCTGAT GTTACATTGC ACAAGATAAA AATATATCAT CATGAACAAT
3541 AAAACTGTCT GCTTACATAA ACAGTAATAC AAGGGGTGTT ATGAGCCATA TTCAACGGGA
3601 AACGTCGAGG CCGCGATTAA ATTCCAACAT GGATGCTGAT TTATATGGGT ATAAATGGGC
3661 TCGCGATAAT GTCGGGCAAT CAGGTGCGAC AATCTATCGC TTGTATGGGA AGCCCGATGC
3721 GCCAGAGTTG TTTCTGAAAC ATGGCAAAGG TAGCGTTGCC AATGATGTTA CAGATGAGAT
3781 GGTCAGACTA AACTGGCTGA CGGAATTTAT GCCTCTTCCG ACCATCAAGC ATTTTATCCG
3841 TACTCCTGAT GATGCATGGT TACTCACCAC TGCGATCCCC GGAAAAACAG CATTCCAGGT
```

```
     3901 ATTAGAAGAA TATCCTGATT CAGGTGAAAA TATTGTTGAT GCGCTGGCAG TGTTCCTGCG
     3961 CCGGTTGCAT TCGATTCCTG TTTGTAATTG TCCTTTTAAC AGCGATCGCG TATTTCGTCT
     4021 CGCTCAGGCG CAATCACGAA TGAATAACGG TTTGGTTGAT GCGAGTGATT TTGATGACGA
     4081 GCGTAATGGC TGGCCTGTTG AACAAGTCTG GAAAGAAATG CATAAACTTT TGCCATTCTC
     4141 ACCGGATTCA GTCGTCACTC ATGGTGATTT CTCACTTGAT AACCTTATTT TTGACGAGGG
     4201 GAAATTAATA GGTTGTATTG ATGTTGGACG AGTCGGAATC GCAGACCGAT ACCAGGATCT
     4261 TGCCATCCTA TGGAACTGCC TCGGTGAGTT TTCTCCTTCA TTACAGAAAC GGCTTTTTCA
     4321 AAAATATGGT ATTGATAATC CTGATATGAA TAAATTGCAG TTTCATTTGA TGCTCGATGA
     4381 GTTTTTCTAA TCAGAATTGG TTAATTGGTT GTAACACTGG CAGAGCATTA CGCTGACTTG
     4441 ACGGGACGGC GCAAGCTCAT GACCAAAATC CCTTAACGTG AGTTACGCGT CGTTCCACTG
     4501 AGCGTCAGAC CCCGTAGAAA AGATCAAAGG ATCTTCTTGA GATCCTTTTT TTCTGCGCGT
     4561 AATCTGCTGC TTGCAAACAA AAAAACCACC GCTACCAGCG GTGGTTTGTT TGCCGGATCA
     4621 AGAGCTACCA ACTCTTTTTC CGAAGGTAAC TGGCTTCAGC AGAGCGCAGA TACCAAATAC
     4681 TGTTCTTCTA GTGTAGCCGT AGTTAGGCCA CCACTTCAAG AACTCTGTAG CACCGCCTAC
     4741 ATACCTCGCT CTGCTAATCC TGTTACCAGT GGCTGCTGCC AGTGGCGATA AGTCGTGTCT
     4801 TACCGGGTTG GACTCAAGAC GATAGTTACC GGATAAGGCG CAGCGGTCGG GCTGAACGGG
     4861 GGGTTCGTGC ACACAGCCCA GCTTGGAGCG AACGACCTAC ACCGAACTGA GATACCTACA
     4921 GCGTGAGCTA TGAGAAAGCG CCACGCTTCC CGAAGGGAGA AAGGCGGACA GGTATCCGGT
     4981 AAGCGGCAGG GTCGGAACAG GAGAGCGCAC GAGGGAGCTT CCAGGGGGAA ACGCCTGGTA
     5041 TCTTTATAGT CCTGTCGGGT TTCGCCACCT CTGACTTGAG CGTCGATTTT TGTGATGCTC
     5101 GTCAGGGGGG CGGAGCCTAT GGAAAAACGC CAGCAACGCG GCCTTTTTAC GGTTCCTGGC
     5161 CTTTTGCTGG CCTTTTGCTC ACATGTT
//
```

pDSARN (DsRed2NLS):

```
LOCUS       pDSARed2nls             5247 bp    DNA     circular
FEATURES             Location/Qualifiers
     misc_feature    complement(3508..3526)
                     /note="M13R"
     misc_feature    1972..2021
                     /note="3x Pax6 binding sites"
     misc_feature    2061..2068
                     /note="TATA"
     misc_feature    271..286
                     /note="M13F"
     misc_feature    600..1174
                     /note="LacZ"
     misc_feature    479..599
                     /note="lac promoter"
     misc_feature    590..608
                     /note="M13R"
     misc_feature    625..644
                     /note="T3"
     misc_feature    677..696
                     /note="SK primer"
     misc_feature    complement(731..747)
                     /note="KS primer"
     misc_feature    complement(800..816)
                     /note="M13F"
     misc_feature    complement(771..790)
                     /note="T7"
     misc_feature    1395..1621
                     /note="SV40 terminator"
     misc_feature    1622..1898
                     /note="attB"
     polyA_signal    2961..3208
                     /dnas_title="SV40"
                     /vntifkey="25"
                     /label=SV40
     misc_feature    complement(3259..3292)
                     /dnas_title="lox66"
```

```
                     /vntifkey="21"
                     /label=lox66
     CDS             2220..2953
                     /dnas_title="DsRed2nls"
                     /vntifkey="4"
                     /label=DsRed2nls

ORIGIN
        1 CTTTCCTGCG TTATCCCCTG ATTCTGTGGA TAACCGTATT ACCGCCTTTG AGTGAGCTGA
       61 TACCGCTCGC CGCAGCCGAA CGACCGAGCG CAGCGAGTCA GTGAGCGAGG AAGCGGAAGA
      121 GCGCCCAATA CGCAAACCGC CTCTCCCCGC GCGTTGGCCG ATTCATTAAT GCAGCTGGCA
      181 CGACAGGTTT CCCGACTGGA AAGCGGGCAG TGAGCGCAAC GCAATTAATA CGCGTACCGC
      241 TAGCATGGAT GTTTTCCCAG TCACGACGTT GTAAAACGAC GGCCAGTCTT AAGCTCGGGC
      301 CCCTACAGGT CACTAATACC ATCTAAGTAG TTGATTCATA GTGACTGGAT ATGTTGTGTT
      361 TTACAGTATT ATGTAGTCTG TTTTTTATGC AAAATCTAAT TTAATATATT GATATTTATA
      421 TCATTTTACG TTTCTCGTTC AACTTTTCTA TACAAAGTTg gtACcggatc cagagaccCG
      481 CAACGCAATT AATGTGAGTT AGCTCACTCA TTAGGCACCC CAGGCTTTAC ACTTTATGCT
      541 TCCGGCTCGT ATGTTGTGTG GAATTGTGAG CGGATAACAA TTTCACACAG GAAACAGCTA
      601 TGACCATGAT TACGCCAAGC GCGCAATTAA CCCTCACTAA AGGGAACAAA AGCTGGAGCT
      661 CCACCGCGGT GGCGGCCGCT CTAGAACTAG TGGATCCCCC GGGCTGCAGG AATTCGATAT
      721 CAAGCTTATC GATACCGTCG ACCTCGAGGG GGGGCCCGGT ACCCAATTCG CCCTATAGTG
      781 AGTCGTATTA CGCGCGCTCA CTGGCCGTCG TTTTACAACG TCGTGACTGG GAAAACCCTG
      841 GCGTTACCCA ACTTAATCGC CTTGCAGCAC ATCCCCCTTT CGCCAGCTGG CGTAATAGCG
      901 AAGAGGCCCG CACCGATCGC CCTTCCCAAC AGTTGCGCAG CCTGAATGGC GAATGGGACG
      961 CGCCCTGTAG CGGCGCATTA AGCGCGGCGG GTGTGGTGGT TACGCGCAGC GTGACCGCTA
     1021 CACTTGCCAG CGCCCTAGCG CCCGCTCCTT TCGCTTTCTT CCCTTCCTTT CTCGCCACGT
     1081 TCGCCGGCTT TCCCCGTCAA GCTCTAAATC GGGGGCTCCC TTTAGGGTTC CGATTTAGTG
     1141 CTTTACGGCA CCTCGACCCC AAAAAACTTG ATTAGGGTGA TGGTTCACGT AGTGGGCCAT
     1201 CGCCCTGATA GACGGTTTTT CGCCCTTTGA CGTTGGAGTC CACGTTCTTT AATAGTGGAC
     1261 TCTTGTTCCA AACTGGAACA ACACTCAACC CTATCTCGGT CTATTCTTTT GATTTATAAG
     1321 GGATTTTGCC GATTTCGGCC TATTGGTTAA AAAATGAGCT GATTTAACAA AAATTTAACG
     1381 CGggtctccg cttCTAGACA TAATCAGCCA TACCACATTT GTAGAGGTTT TACTTGCTTT
     1441 AAAAAACCTC CCACACCTCC CCCTGAACCT GAAACATAAA ATGAATGCAA TTGTTGTTGT
     1501 TAACTTGTTT ATTGCAGCTT ATAATGGTTA CAAATAAAGC AATAGCATCA CAAATTTCAC
     1561 AAATAAAGCA TTTTTTCTTCA CTGCATTCTA GTTGTGGTTT GTCCAAACTC ATCAATGTAT
     1621 CtcgaCGATG TAGGTCACaG TCTCGAAGCC GCGGTGCGGG TGCCAGGGCG TGCCCTTGGG
     1681 CTCCCCGGGC GCGTACTCCA CCTCACCCAT CTGGTCCATC ATGATGAACG GGTCGAGGTG
     1741 GCGGTAGTTG ATCCCGGCGA ACGCGCGGCG CACCGGGAAG CCCTCGCCCT CGAAACCGCT
     1801 GGGCGCGGTG GTCACGGTGA GCACGGGACG TGCGACGGCG TCGGCGGGTG CGGATACGCG
     1861 GGGCAGCGTC AGCGGGTTCT CGACGGTCAC GGCGGGCAat tCCTGCAGAC TTCCGGTATC
     1921 TCGCGTTTGT TTGATCGCAC GGTTCCCACA ATGGTTAATT CGAGCTCGCC CGGGGATCTA
     1981 ATTCAATTAG AGACTAATTC AATTAGAGCT AATTCAATTA GGATCCAAGC TTATCGATTT
     2041 CGAACCCTCG ACCGCCGGAG TATAAATAGA GGCGCTTCGT CTACGGAGCG ACAATTCAAT
     2101 TCAAACAAGC AAAGTGAACA CGTCGCTAAG CGAAAGCTAA GCAAATAAAC AAGCGCAGCT
     2161 GAACAAGCTA AACAATCGGG GTACCGCTAG AGTCGACGGT ACCGCGGGCC CGGGATCCAc
     2221 cggtcgccac catggcctcc tccgagaacg tcatcaccga gttcatgcgc ttcaaggtgc
     2281 gcatggaggg caccgtgaac ggccacgagt tcgagatcga gggcgagggc gagggccgcc
     2341 cctacgaggg ccacaacacc gtgaagctga aggtgaccaa gggcggcccc ctgcccttcg
     2401 cctgggacat cctgtccccc cagttccagt acggctccaa ggtgtacgtg aagcaccccg
     2461 ccgacatccc cgactacaag aagctgtcct tccccgaggg cttcaagtgg gagcgcgtga
     2521 tgaacttcga ggacggcggc gtggcgaccg tgacccagga ctcctccctg caggacggct
     2581 gcttcatcta caaggtgaag ttcatcggcg tgaacttccc ctccgacggc cccgtgatgc
     2641 agaagaagac catgggctgg gaggcctcca ccgagcgcct gtaccccgc gacggcgtgc
     2701 tgaagggcga gacccacaag gccctgaagc tgaaggacgg cggccactac ctggtggagt
     2761 tcaagtccat ctacatggcc aagaagcccg tgcagctgcc cggctactac tacgtggacg
     2821 ccaagctgga catcacctcc cacaacgagg actacaccat cgtggagcag tacgagcgca
     2881 ccgagggccg ccaccacctg ttcctgagat ctcgacccaa gaaaaagcgg aaggtggagg
     2941 acccgtaaga tccaccggat ctagataact gatcataatc agccatacca catttgtaga
     3001 ggttttactt gctttaaaaa acctcccaca cctcccccctg aacctgaaac ataaaatgaa
     3061 tgcaattgtt gttgttaact tgtttattgc agcttataat ggttacaaat aaagcaatag
     3121 catcacaaat ttcacaaata aagcattttt ttcactgcat tctagttgtg gtttgtccaa
     3181 actcatcaat gtatcttatc atgtctggat cccgtttgac ggtatcgata agcttgatgg
     3241 ggatccggaa cccttaatta ccgttcgtat aatgtatgct atacgaagtt attaggtccc
     3301 tcgacctgca gcccGTACGG CGCGCCTAGA GCGGCCGCCA CCGCGGTGGA GCTCGAGTAC
     3361 CCAGCTTTCT TGTACAAAGT TGGCATTATA AGAAAGCATT GCTTATCAAT TTGTTGCAAC
```

```
     3421 GAACAGGTCA CTATCAGTCA AAATAAAATC ATTATTTGCC ATCCAGCTGC AGGGCGGCCG
     3481 CGATATCCCC TATAGTGAGT CGTATTACAT GGTCATAGCT GTTTCCTGGC AGCTCTGGCC
     3541 CGTGTCTCAA AATCTCTGAT GTTACATTGC ACAAGATAAA AATATATCAT CATGAACAAT
     3601 AAAACTGTCT GCTTACATAA ACAGTAATAC AAGGGGTGTT ATGAGCCATA TTCAACGGGA
     3661 AACGTCGAGG CCGCGATTAA ATTCCAACAT GGATGCTGAT TTATATGGGT ATAAATGGGC
     3721 TCGCGATAAT GTCGGGCAAT CAGGTGCGAC AATCTATCGC TTGTATGGGA AGCCCGATGC
     3781 GCCAGAGTTG TTTCTGAAAC ATGGCAAAGG TAGCGTTGCC AATGATGTTA CAGATGAGAT
     3841 GGTCAGACTA AACTGGCTGA CGGAATTTAT GCCTCTTCCG ACCATCAAGC ATTTTATCCG
     3901 TACTCCTGAT GATGCATGGT TACTCACCAC TGCGATCCCC GGAAAAACAG CATTCCAGGT
     3961 ATTAGAAGAA TATCCTGATT CAGGTGAAAA TATTGTTGAT GCGCTGGCAG TGTTCCTGCG
     4021 CCGGTTGCAT TCGATTCCTG TTTGTAATTG TCCTTTTAAC AGCGATCGCG TATTTCGTCT
     4081 CGCTCAGGCG CAATCACGAA TGAATAACGG TTTGGTTGAT GCGAGTGATT TTGATGACGA
     4141 GCGTAATGGC TGGCCTGTTG AACAAGTCTG GAAAGAAATG CATAAACTTT TGCCATTCTC
     4201 ACCGGATTCA GTCGTCACTC ATGGTGATTT CTCACTTGAT AACCTTATTT TTGACGAGGG
     4261 GAAATTAATA GGTTGTATTG ATGTTGGACG AGTCGGAATC GCAGACCGAT ACCAGGATCT
     4321 TGCCATCCTA TGGAACTGCC TCGGTGAGTT TTCTCCTTCA TTACAGAAAC GGCTTTTTCA
     4381 AAAATATGGT ATTGATAATC CTGATATGAA TAAATTGCAG TTTCATTTGA TGCTCGATGA
     4441 GTTTTTCTAA TCAGAATTGG TTAATTGGTT GTAACACTGG CAGAGCATTA CGCTGACTTG
     4501 ACGGGACGGC GCAAGCTCAT GACCAAAATC CCTTAACGTG AGTTACGCGT CGTTCCACTG
     4561 AGCGTCAGAC CCCGTAGAAA AGATCAAAGG ATCTTCTTGA GATCCTTTTT TTCTGCGCGT
     4621 AATCTGCTGC TTGCAAACAA AAAAACCACC GCTACCAGCG GTGGTTTGTT TGCCGGATCA
     4681 AGAGCTACCA ACTCTTTTTC CGAAGGTAAC TGGCTTCAGC AGAGCGCAGA TACCAAATAC
     4741 TGTTCTTCTA GTGTAGCCGT AGTTAGGCCA CCACTTCAAG AACTCTGTAG CACCGCCTAC
     4801 ATACCTCGCT CTGCTAATCC TGTTACCAGT GGCTGCTGCC AGTGGCGATA AGTCGTGTCT
     4861 TACCGGGTTG GACTCAAGAC GATAGTTACC GGATAAGGCG CAGCGGTCGG GCTGAACGGG
     4921 GGGTTCGTGC ACACAGCCCA GCTTGGAGCG AACGACCTAC ACCGAACTGA GATACCTACA
     4981 GCGTGAGCTA TGAGAAAGCG CCACGCTTCC CGAAGGGAGA AAGGCGGACA GGTATCCGGT
     5041 AAGCGGCAGG GTCGGAACAG GAGAGCGCAC GAGGGAGCTT CCAGGGGGAA ACGCCTGGTA
     5101 TCTTTATAGT CCTGTCGGGT TTCGCCACCT CTGACTTGAG CGTCGATTTT TGTGATGCTC
     5161 GTCAGGGGGG CGGAGCCTAT GGAAAAACGC CAGCAACGCG GCCTTTTTAC GGTTCCTGGC
     5221 CTTTTGCTGG CCTTTTGCTC ACATGTT
//
```

pattB-RfB2 (ampicillin resistant attB site-containing vector for docking site-based transgenesis, compatible with Gateway and Multisite Gateway cloning. Transform a *ccdB* resistant *E. coli* strain!)

```
LOCUS       pattB-RfB2             4884 bp    DNA     circular
FEATURES             Location/Qualifiers
     misc_feature    2385..2669
                     /note="attB"
     misc_feature    complement(2693..2712)
                     /note="T3"
     misc_feature    complement(2729..2747)
                     /note="M13R"
     misc_feature    622..642
                     /note="T7"
     misc_feature    complement(661..2373)
                     /note="RfB Gateway cassette"
     misc_feature    666..789
                     /note="attR2"
     misc_feature    complement(2245..2369)
                     /note="attR1"
     misc_feature    600..616
                     /note="M13F"
     source          1..4884
                     /dnas_title="pattB-RfB2"

ORIGIN
        1 CTAAATTGTA AGCGTTAATA TTTTGTTAAA ATTCGCGTTA AATTTTTGTT AAATCAGCTC
       61 ATTTTTTAAC CAATAGGCCG AAATCGGCAA AATCCCTTAT AAATCAAAAG AATAGACCGA
```

```
 121 GATAGGGTTG AGTGTTGTTC CAGTTTGGAA CAAGAGTCCA CTATTAAAGA ACGTGGACTC
 181 CAACGTCAAA GGGCGAAAAA CCGTCTATCA GGGCGATGGC CCACTACGTG AACCATCACC
 241 CTAATCAAGT TTTTTGGGGT CGAGGTGCCG TAAAGCACTA AATCGGAACC CTAAAGGGAG
 301 CCCCCGATTT AGAGCTTGAC GGGGAAAGCC GGCGAACGTG GCGAGAAAGG AAGGGAAGAA
 361 AGCGAAAGGA GCGGGCGCTA GGGCGCTGGC AAGTGTAGCG GTCACGCTGC GCGTAACCAC
 421 CACACCCGCC GCGCTTAATG CGCCGCTACA GGGCGCGTCC CATTCGCCAT TCAGGCTGCG
 481 CAACTGTTGG GAAGGGCGAT CGGTGCGGGC CTCTTCGCTA TTACGCCAGC TGGCGAAAGG
 541 GGGATGTGCT GCAAGGCGAT TAAGTTGGGT AACGCCAGGG TTTTCCCAGT CACGACGTTG
 601 TAAAACGACG GCCAGTGAAT TGTAATACGA CTCACTATAG GGCGAATTGG GTACGTACCG
 661 atcaaccact ttgtacaaga aagctgaacg agaaacgtaa aatgatataa atatcaatat
 721 attaaattag attttgcata aaaaacagac tacataatac tgtaaaacac aacatatcca
 781 gtcactatgg tcgacctgca gactggctgt gtataaggga gcctgacatt tatattcccc
 841 agaacatcag gttaatggcg tttttgatgt cattttcgcg gtggctgaga tcagccactt
 901 cttccccgat aacggagacc ggcacactgg ccatatcggt ggtcatcatg cgccagcttt
 961 catccccgat atgcaccacc gggtaaagtt cacgggagac tttatctgac agcagacgtg
1021 cactggccag ggggatcacc atccgtcgcc cgggcgtgtc aataatatca ctctgtacat
1081 ccacaaacag acgataacgg ctctctcttt tataggtgta aaccttaaac tgcatttcac
1141 cagcccctgt tctcgtcagc aaaagagccg ttcatttcaa taaaccgggc gacctcagcc
1201 atcccttcct gattttccgc tttccagcgt tcggcacgca gacgacgggc ttcattctgc
1261 atggttgtgc ttaccagacc ggagatattg acatcatata tgccttgagc aactgatagc
1321 tgtcgctgtc aactgtcact gtaatacgct gcttcatagc atacctcttt ttgacatact
1381 tcgggtatac atatcagtat atattcttat accgcaaaaa tcagcgcgca aatacgcata
1441 ctgttatctg gcttttagta agccggatcc agatctttac gccccgccct gccactcatc
1501 gcagtactgt tgtaattcat taagcattct gccgacatgg aagccatcac aaacggcatg
1561 atgaacctga atcgccagcg gcatcagcac cttgtcgcct tgcgtataat atttgcccat
1621 ggtgaaaacg gggcgaaga agttgtccat attggccacg tttaaatcaa aactggtgaa
1681 actcacccag ggattggctg agacgaaaaa catattctca ataaaccctt tagggaaata
1741 ggccaggttt tcaccgtaac acgccacatc ttgcgaatat atgtgtagaa actgccggaa
1801 atcgtcgtgg tattcactcc agagcgatga aaacgtttca gtttgctcat ggaaaacggt
1861 gtaacaaggg tgaacactat cccatatcac cagctcaccg tctttcattg ccatacggaa
1921 ttccggatga gcattcatca ggcgggcaag aatgtgaata aaggccggat aaaacttgtg
1981 cttatttttc tttacggtct ttaaaaaggc cgtaatatcc agctgaacgg tctggttata
2041 ggtacattga gcaactgact gaaatgcctc aaaatgttct ttacgatgcc attgggatat
2101 atcaacggtg gtatatccag tgattttttt ctccatttta gcttccttag ctcctgaaaa
2161 tctcgacgga tcctaactca aaatccacac attatacgag ccggaagcat aaagtgtaaa
2221 gcctggggtg cctaatgcgg ccgccatagt gactggatat gttgtgtttt acagtattat
2281 gtagtctgtt ttttatgcaa aatctaattt aatatattga tatttatatc attttacgtt
2341 tctcgttcag ctttttgta caaacttgtt gatgatcCAC TAGTGTCGAC GATGTAGGTC
2401 ACGGTCTCGA AGCCGCGGTG CGGGTGCCAG GGCGTGCCCT TGGGCTCCCC GGGCGCGTAC
2461 TCCACCTCAC CCATCTGGTC CATCATGATG AACGGGTCGA GGTGGCGGTA GTTGATCCCG
2521 GCGAACGCGC GGCGCACCGG GAAGCCCTCG CCCTCGAAAC CGCTGGGCGC GGTGGTCACG
2581 GTGAGCACGG GACGTGCGAC GGCGTCGGCG GGTGCGGATA CGCGGGGCAG CGTCAGCGGG
2641 TTCTCGACGG TCACGGCGGG CATGTCGACA CTAGTTCTAG CCAGCTTTTG TTCCCTTTAG
2701 TGAGGGTTAA TTTCGAGCTT GGCGTAATCA TGGTCATAGC TGTTTCCTGT GTGAAATTGT
2761 TATCCGCTCA CAATTCCACA CAACATACGA GCCGGAAGCA TAAAGTGTAA AGCCTGGGGT
2821 GCCTAATGAG TGAGCTAACT CACATTAATT GCGTTGCGCT CACTGCCCGC TTTCCAGTCG
2881 GGAAACCTGT CGTGCCAGCT GCATTAATGA ATCGGCCAAC GCGCGGGGAG AGGCGGTTTG
2941 CGTATTGGGC GCTCTTCCGC TTCCTCGCTC ACTGACTCGC TGCGCTCGGT CGTTCGGCTG
3001 CGGCGAGCGG TATCAGCTCA CTCAAAGGCG GTAATACGGT TATCCACAGA ATCAGGGGAT
3061 AACGCAGGAA AGAACATGTG AGCAAAAGGC CAGCAAAAGG CCAGGAACCG TAAAAAGGCC
3121 GCGTTGCTGG CGTTTTTCCA TAGGCTCCGC CCCCCTGACG AGCATCACAA AAATCGACGC
3181 TCAAGTCAGA GGTGGCGAAA CCCGACAGGA CTATAAAGAT ACCAGGCGTT TCCCCCTGGA
3241 AGCTCCCTCG TGCGCTCTCC TGTTCCGACC CTGCCGCTTA CCGGATACCT GTCCGCCTTT
3301 CTCCCTTCGG GAAGCGTGGC GCTTTCTCAT AGCTCACGCT GTAGGTATCT CAGTTCGGTG
3361 TAGGTCGTTC GCTCCAAGCT GGGCTGTGTG CACGAACCCC CCGTTCAGCC CGACCGCTGC
3421 GCCTTATCCG GTAACTATCG TCTTGAGTCC AACCCGGTAA GACACGACTT ATCGCCACTG
3481 GCAGCAGCCA CTGGTAACAG GATTAGCAGA GCGAGGTATG TAGGCGGTGC TACAGAGTTC
3541 TTGAAGTGGT GGCCTAACTA CGGCTACACT AGAAGGACAG TATTTGGTAT CTGCGCTCTG
3601 CTGAAGCCAG TTACCTTCGG AAAAAGAGTT GGTAGCTCTT GATCCGGCAA ACAAACCACC
3661 GCTGGTAGCG GTGGTTTTTT TGTTTGCAAG CAGCAGATTA CGCGCAGAAA AAAAGGATCT
3721 CAAGAAGATC CTTTGATCTT TTCTACGGGG TCTGACGCTC AGTGGAACGA AAACTCACGT
3781 TAAGGGATTT TGGTCATGAG ATTATCAAAA AGGATCTTCA CCTAGATCCT TTTAAATTAA
3841 AAATGAAGTT TTAAATCAAT CTAAAGTATA TATGAGTAAA CTTGGTCTGA CAGTTACCAA
3901 TGCTTAATCA GTGAGGCACC TATCTCAGCG ATCTGTCTAT TTCGTTCATC CATAGTTGCC
3961 TGACTCCCCG TCGTGTAGAT AACTACGATA CGGGAGGGCT TACCATCTGG CCCCAGTGCT
```

```
     4021 GCAATGATAC CGCGAGACCC ACGCTCACCG GCTCCAGATT TATCAGCAAT AAACCAGCCA
     4081 GCCGGAAGGG CCGAGCGCAG AAGTGGTCCT GCAACTTTAT CCGCCTCCAT CCAGTCTATT
     4141 AATTGTTGCC GGGAAGCTAG AGTAAGTAGT TCGCCAGTTA ATAGTTTGCG CAACGTTGTT
     4201 GCCATTGCTA CAGGCATCGT GGTGTCACGC TCGTCGTTTG GTATGGCTTC ATTCAGCTCC
     4261 GGTTCCCAAC GATCNAAGGC GAGTTACATG ATNCCCCCAT GTTGTGCAAA AAAGCGGTTA
     4321 GCTCCTTCGG TCCTCCGATC GTTGTCAGAA GTAAGTTGGC CGCAGTGTTA TCACTCATGG
     4381 TTATGGCAGC ACTGCATAAT TCTCTTACTG TCATGCCATC CGTAAGATGC TTTTCTGTGA
     4441 CTGGTGAGTA CTCAACCAAG TCATTCTGAG AATAGTGTAT GCGGCGACCG AGTTGCTCTT
     4501 GCCCGGCGTC AATACGGGAT AATACCGCGC CACATAGCAG AACTTTAAAA GTGCTCATCA
     4561 TTGGAAAACG TTCTTCGGGG CGAAAACTCT CAAGGATCTT ACCGCTGTTG AGATCCAGTT
     4621 CGATGTAACC CACTCGTGCA CCCAACTGAT CTTCAGCATC TTTTACTTTC ACCAGCGTTT
     4681 CTGGGTGAGC AAAAACAGGA AGGCAAAATG CCGCAAAAAA GGGAATAAGG GCGACACGGA
     4741 AATGTTGAAT ACTCATACTC TTCCTTTTTC AATATTATTG AAGCATTTAT CAGGGTTATT
     4801 GTCTCATGAG CGGATACATA TTTGAATGTA TTTAGAAAAA TAAACAAATA GGGGTTCCGC
     4861 GCACATTTCC CCGAAAAGTG CCAC

//
```

<u>pXLtTloxattPGat2 (ampicillin resistant *piggyBac* transgenesis vector with transposase in backbone and Gateway cloning cassette. Ampicillin resistant):</u>

```
LOCUS       pXLBacIItTloxatt        8692 bp    DNA     circular
FEATURES             Location/Qualifiers
     misc_feature    3718..3751
                     /note="loxP"
     misc_feature    5522..5742
                     /note="attP'"
     misc_feature    complement(6539..6557)
                     /note="M13R"
     misc_feature    3322..3338
                     /note="M13F"
     misc_feature    complement(339..2405)
                     /note="transposase"
     misc_feature    complement(2462..3049)
                     /note="hsp70 promoter"
     misc_feature    complement(2407..2462)
                     /note="rrnB terminator"
     misc_feature    5478..5511
                     /note="loxP"
     misc_feature    complement(3933..4238)
                     /note="ccdB ORF"
     misc_feature    3768..3892
                     /note="attR2"
     misc_feature    complement(4580..5239)
                     /note="chloramphenicol resistance ORF"
     misc_feature    complement(5348..5472)
                     /note="attR1"
     misc_feature    complement(3468..3625)
                     /note="piggyBac 3'region"
     misc_feature    complement(3768..5472)
                     /note="Gateway cassette"
     misc_feature    5917..6053
                     /note="piggyBac 5' region"
     source          1..8692
                     /dnas_title="pXLBacIItTloxatt"
ORIGIN
        1 CTAAATTGTA AGCGTTAATA TTTTGTTAAA ATTCGCGTTA AATTTTTGTT AAATCAGCTC
       61 ATTTTTTAAC CAATAGGCCG AAATCGGCAA AATCCCTTAT AAATCAAAAG AATAGACCGA
      121 GATAGGGTTG AGTGTTGTTC CAGTTTGGAA CAAGAGTCCA CTATTAAAGA ACGTGGACTC
      181 CAACGTCAAA GGGCGAAAAA CCGTCTATCA GGGCGATGGC CCACTACGTG AACCATCACC
      241 CTAATCAAGT TTTTTGGGGT CGAGGTGCCG TAAAGCACTA AATCGGAACC CTAAAGGGAG
      301 CCCCCGATTT AGAGCTTGAC GGGGAAAGCC TCGACGGATC CAAATTCAAC AAACAATTTA
      361 TGTTTATTTA TTTATTAAAA AAAAACAAAA ACTCAAAATT TCTTCTATAA AGTAACAAAA
```

421 CTTTTAAACA TTCTCTCCTT TACAAAAATA AACTTATTTT GTACTTTAAA AACAGTCATG
481 TTGTATTATA AAATAAGTAA TTAGTTTAAC TTATACATAA TAGAAACAAA TTATACTTAT
541 TAGTCAGTCA GAAACAACTT TGGCACATAT CAATATTATG CTCTCGACAA ATAACTTTTT
601 TGCATTTTTT GCACGATGCA TTTGCCTTTC GCCTTATTTT AGAGGGGCAG TAAGTACAGT
661 AAGTACGTTT TTTCATTACT GGCTCTTCAG TACTGTCATC TGATGTACCA GGCACTTCAT
721 TTGGCAAAAT ATTAGAGATA TTATCGCGCA AATATCTCTT CAAAGTAGGA GCTTCTAAAC
781 GCTTACGCAT AAACGATGAC GTCAGGCTCA TGTAAAGGTT TCTCATAAAT TTTTTGCGAC
841 TTTGAACCTT TTCTCCCTTG CTACTGACAT TATGGCTGTA TATAATAAAA GAATTTATGC
901 AGGCAATGTT TATCATTCCG TACAATAATG CCATAGGCCA CCTATTCGTC TTCCTACTGC
961 AGGTCATCAC AGAACACATT TGGTCTAGCG TGTCCACTCC GCCTTTAGTT TGATTATAAT
1021 ACATAACCAT TTGCGGTTTA CCGGTACTTT CGTTGATAGA AGCATCCTCA TCACAAGATG
1081 ATAATAAGTA TACCATCTTA GCTGGCTTCG GTTTATATGA GACGAGAGTA AGGGGTCCGT
1141 CAAAACAAAA CATCGATGTT CCCACTGGCC TGGAGCGACT GTTTTTCAGT ACTTCCGGTA
1201 TCTCGCGTTT GTTTGATCGC ACGGTTCCCA CAATGGTTAA CTTATACGGT TCTTGTAGTA
1261 AGTTTTTTGC CAAAGGGATT GAGGTGAACC AATTGTCACA CGTAATATTA CGACAACTAC
1321 CGTGCACAGG CTTTGATAAC TCCTTCACGT AGTATTCACC GAGTGGTACT CCGTTGGTCT
1381 GTGTTCCTCT TCCCAAATAA GGCATTCCAT TTATCATATA CTTCGTACCA CTGTCACACA
1441 TCATGAGGAT TTTTATTCCA TACTTACTTG GCTTGTTTGG GATATACATC CTAAACGGAC
1501 ACCGTCCTCT AAAACCAAGT AACTGTTCAT CTATGGTCAA ATGAGCCCCT GGAGTGTAAT
1561 TTTGTATGCA CTGATGGATA AAGAGATCCC ATATTTTTCT AACAGGAGTA AATACATCGT
1621 TTTCTCGAAG TGTGGGCCGT ATACTTTTGT CATCCATTCT AAGACATCGT ATCAAAAAAT
1681 CAAAACGATC ACGACTCATT ACAGAGACGT ACACCATTGA CAAAGATCGA TCAAAGAGGT
1741 CATCTGTGGA CATGTGGTTA TCTTTTCTCA CTGCTGTCAT TACCAGAATA CCAAAGAAAG
1801 CATAGATTTC ATCTTCATTC GTGTCACGAA ATGTAGCACC TGTCATAGAT TCCCGACGTT
1861 TCAATGATAT CTCAGCATTT GTCCATTTTA CAATTTCCGA AATTATCTCA TCAGTAAAAA
1921 ATAGTTTGAA GCATAAAAGT GGGTCATATA TATTGCGGCA CATACGCGTC GGACCTCTTT
1981 GAGATCTGAC AATGTTCAGT GCAGAGACTC GGCTACGCCT CGTGGACTTT GAAGTTGACC
2041 AACAATGTTT ATTCTTACCT CTAATAGTCC TCTGTGGCAA GGTCAAGATT CTGTTAGAAG
2101 CCAATGAAGA ACCTGGTTGT TCAATAACAT TTTGTTCGTC TAATATTTCA CTACCGCTTG
2161 ACGTTGGCTG CACTTCATGT ACCTCATCTA TAAACGCTTC TTCTGTATCG CTCTGGACGT
2221 CATCTTCACT TACGTGATCT GATATTTCAC TGTCAGAATC CTCACCAACA AGCTCGTCAT
2281 CGCTTTGCAG AAGAGCAGAG AGGATATGCT CATCGTCTAA AGAACTACCC ATTTTATTAT
2341 ATAGGATCCC CGACACCAGA CCAACTGGTA ATGGTAGCGA CCGGCGCTCA GCTGGAATTA
2401 GGCCTTCTAG acagataaaa cgaaaggccc agtctttcga ctgagccttt cgttttattt
2461 gAAATTCCCA ATTCCCTATT CAGAGTTCTC TTCTTGTATT CAATAATTAC TTCTTGGCAG
2521 ATTTCAGTAG TTGCAGTTGA TTTACTTGGT TGCTGGTTAC TTTTAATTGA TTCACTTTAA
2581 CTTGCACTTT ACTGCAGATT GTTTAGCTTG TTCAGCTGCG CTTGTTTATT TGCTTAGCTT
2641 TCGCTTAGCG ACGTGTTCAC TTTGCTTGTT TGAATTGAAT TGTCGCTCCG TAGACGAAGC
2701 GCCTCTATTT ATACTCCGGC GCTCTTTTCG CGAACATTCG AGGCGCGCTC TCTCGAACCA
2761 ACGAGAGCAG TATGCCGTTT ACTGTGTGAC AGAGTGAGAG AGCATTAGTG CAGAGAGGGA
2821 GAGACCCAAA AAGAAAAGAG AGAATAACGA ATAACGGCCA GAGAAATTTC TCGAGTTTTC
2881 TTTCTGCCAA ACAAATGACC TACCACAATA ACCAGTTTGT TTTGGGATTC TAGGGGGATC
2941 GGGGATCAAT TCTAGTATGT ATGTAAGTTA ATAAAACCCA TTTTTGCGGA AAGTAGATAA
3001 AAAAAACATT TTTTTTTTTT ACTGCACTGG ATATCATTGA ACTTATCTGA TCGGCGAACG
3061 TGGCGAGAAA GGAAGGGAAG AAAGCGAAAG GAGCGGGCGC TAGGGCGCTG GCAAGTGTAG
3121 CGGTCACGCT GCGCGTAACC ACCACACCCG CCGCGCTTAA TGCGCCGCTA CAGGGCGCGT
3181 CCCATTCGCC ATTCAGGCTG CGCAACTGTT GGGAAGGGCG ATCGGTGCGG GCCTCTTCGC
3241 TATTACGCCA GCTGGCGAAA GGGGGATGTG CTGCAAGGCG ATTAAGTTGG GTAACGCCAG
3301 GGTTTTCCCA GTCACGACGT TGTAAAACGA CGGCCAGTGA GCGCGCCTCG TTCATTCACG
3361 TTTTTGAACC CGTGGAGGAC GGGCAGACTC GCGGTGCAAA TGTGTTTTAC AGCGTGATGG
3421 AGCAGATGAA GATGCTCGAC ACGCTGCAGA ACACGCAGCT AGATTAACCC TAGAAAGATA
3481 ATCATATTGT GACGTACGTT AAAGATAATC ATGCGTAAAA TTGACGCATG TGTTTTATCG
3541 GTCTGTATAT CGAGGTTTAT TTATTAATTT GAATAGATAT TAAGTTTTAT TATATTTACA
3601 CTTACATACT AATAATAAAT TCAACAAACA ATTTATTTAT GTTTATTTAT TTATTAAAAA
3661 AAACAAAAAC TCAAAATTTC TTCTATAAAG TAACAAAACT TTTATCGAAT TGAGCTCata
3721 acttcgtata atgtatgcta tacgaagtta tGTCGACGAA TTACATCACC ACTTTGTACA
3781 AGAAAGCTGA ACGAGAAACG TAAAATGATA TAAATATCAA TATATTAAAT TAGATTTTGC
3841 ATAAAAAACA GACTACATAA TACTGTAAAA CACAACATAT CCAGTCACTA TGGTCGACCT
3901 GCAGACTGGC TGTGTATAAG GGAGCCTGAC ATTTATATTC CCCAGAACAT CAGGTTAATG
3961 GCGTTTTTGA TGTCATTTTC GCGGTGGCTG AGATCAGCCA CTTCTTCCCC GATAACGGAG
4021 ACCGGCACAC TGGCCATATC GGTGGTCATC ATGCGCCAGC TTTCATCCCC GATATGCACC
4081 ACCGGGTAAA GTTCACGGGA GACTTTATCT GACAGCAGAC GTGCACTGGC CAGGGGGATC
4141 ACCATCCGTC GCCCGGGCGT GTCAATAATA TCACTCTGTA CATCCACAAA CAGACGATAA
4201 CGGCTCTCTC TTTTATAGGT GTAAACCTTA AACTGCATTT CACCAGCCCC TGTTCTCGTC
4261 AGCAAAAGAG CCGTTCATTT CAATAAACCG GGCGACCTCA GCCATCCCTT CCTGATTTTC

```
4321 CGCTTTCCAG CGTTCGGCAC GCAGACGACG GGCTTCATTC TGCATGGTTG TGCTTACCAG
4381 ACCGGAGATA TTGACATCAT ATATGCCTTG AGCAACTGAT AGCTGTCGCT GTCAACTGTC
4441 ACTGTAATAC GCTGCTTCAT AGCATACCTC TTTTTGACAT ACTTCGGGTA TACATATCAG
4501 TATATATTCT TATACCGCAA AAATCAGCGC GCAAATACGC ATACTGTTAT CTGGCTTTTA
4561 GTAAGCCGGA TCCACGCGTT TACGCCCCGC CCTGCCACTC ATCGCAGTAC TGTTGTAATT
4621 CATTAAGCAT TCTGCCGACA TGGAAGCCAT CACAAACGGC ATGATGAACC TGAATCGCCA
4681 GCGGCATCAG CACCTTGTCG CCTTGCGTAT AATATTTGCC CATGGTGAAA ACGGGGGCGA
4741 AGAAGTTGTC CATATTGGCC ACGTTTAAAT CAAAACTGGT GAAACTCACC CAGGGATTGG
4801 CTGAGACGAA AAACATATTC TCAATAAACC CTTTAGGGAA ATAGGCCAGG TTTTCACCGT
4861 AACACGCCAC ATCTTGCGAA TATATGTGTA GAAACTGCCG GAAATCGTCG TGGTATTCAC
4921 TCCAGAGCGA TGAAAACGTT TCAGTTTGCT CATGGAAAAC GGTGTAACAA GGGTGAACAC
4981 TATCCCATAT CACCAGCTCA CCGTCTTTCA TTGCCATACG GAATTCCGGA TGAGCATTCA
5041 TCAGGCGGGC AAGAATGTGA ATAAAGGCCG GATAAAACTT GTGCTTATTT TTCTTTACGG
5101 TCTTTAAAAA GGCCGTAATA TCCAGCTGAA CGGTCTGGTT ATAGGTACAT TGAGCAACTG
5161 ACTGAAATGC CTCAAAATGT TCTTTACGAT GCCATTGGGA TATATCAACG GTGGTATATC
5221 CAGTGATTTT TTTCTCCATT TTAGCTTCCT TAGCTCCTGA AAATCTCGAC GGATCCTAAC
5281 TCAAAATCCA CACATTATAC GAGCCGGAAG CATAAAGTGT AAAGCCTGGG GTGCCTAATG
5341 CGGCCGCCAT AGTGACTGGA TATGTTGTGT TTTACAGTAT TATGTAGTCT GTTTTTTATG
5401 CAAAATCTAA TTTAATATAT TGATATTTAT ATCATTTTAC GTTTCTCGTT CAGCTTTTTT
5461 GTACAAACTT GTGATGTata acttcgtata atgtatgcta tacgaagtta tGGTACCTGC
5521 AGTACTGACG GACACACCGA AGCCCCGGCG GCAACCCTCA GCGGATGCCC CGGGGCTTCA
5581 CGTTTTCCCA GGTCAGAAGC GGTTTTCGGG AGTAGTGCCC CAACTGGGGT AACCTTTGAG
5641 TTCTCTCAGT TGGGGGCGTA GGGTCGCCGA CATGACACAA GGGGTTGTGA CCGGGGTGGA
5701 CACGTACGCG GGTGCTTACG ACCGTCAGTC GCGCGAGCGC GATCTAATCT ATAACAAGAA
5761 AATATATATA TAATAAGTTA TCACGTAAGT AGAACATGAA ATAACAATAT AATTATCGTA
5821 TGAGTTAAAT CTTAAAAGTC ACGTAAAAGA TAATCATGCG TCATTTTGAC TCACGCGGTC
5881 GTTATAGTTC AAAATCAGTG ACACTTACCG CATTGACAAG CACGCCTCAC GGGAGCTCCA
5941 AGCGGCGACT GAGATGTCCT AAATGCACAG CGACGGATTC GCGCTATTTA GAAAGAGAGA
6001 GCAATATTTC AAGAATGCAT GCGTCAATTT TACGCAGACT ATCTTTCTAG GGTTAATCTA
6061 GCTGCATCAG GATCATATCG TCGGGTCTTT TTTCCGGCTC AGTCATCGCC CAAGCTGGCG
6121 CTATCTGGGC ATCGGGGAGG AAGAAGCCCG TGCCTTTTCC CGCGAGGTTG AAGCGGCATG
6181 GAAAGAGTTT GCCGAGGATG ACTGCTGCTG CATTGACGTT GAGCGAAAAC GCACGTTTAC
6241 CATGATGATT CGGGAAGGTG TGGCCATGCA CGCCTTTAAC GGTGAACTGT TCGTTCAGGC
6301 CACCTGGGAT ACCAGTTCGT CGCGGCTTTT CCGGACACAG TTCCGGATGG TCAGCCCGAA
6361 GCGCATCAGC AACCCGAACA ATACCGGCGA CAGCCGGAAC TGCCGTGCCG GTGTGCAGAT
6421 TAATGACAGC GGTGCGGCGC TGGGATATTA CGTCAGCGAG GACGGGTATC CTGGCTGGAT
6481 GCCGCAGAAA TGGACATGGA TACCCCGTGA GTTACCCGGC GGGCGCGCTT GGCGTAATCA
6541 TGGTCATAGC TGTTTCCTGT GTGAAATTGT TATCCGCTCA CAATTCCACA CAACATACGA
6601 GCCGGAAGCA TAAAGTGTAA AGCCTGGGGT GCCTAATGAG TGAGCTAACT CACATTAATT
6661 GCGTTGCGCT CACTGCCCGC TTTCCAGTCG GGAAACCTGT CGTGCCAGCT GCATTAATGA
6721 ATCGGCCAAC GCGCGGGGAG AGGCGGTTTG CGTATTGGGC GCTCTTCCGC TTCCTCGCTC
6781 ACTGACTCGC TGCGCTCGGT CGTTCGGCTG CGGCGAGCGG TATCAGCTCA CTCAAAGGCG
6841 GTAATACGGT TATCCACAGA ATCAGGGGAT AACGCAGGAA AGAACATGTG AGCAAAAGGC
6901 CAGCAAAAGG CCAGGAACCG TAAAAAGGCC GCGTTGCTGG CGTTTTTCCA TAGGCTCCGC
6961 CCCCCTGACG AGCATCACAA AAATCGACGC TCAAGTCAGA GGTGGCGAAA CCCGACAGGA
7021 CTATAAAGAT ACCAGGCGTT TCCCCCTGGA AGCTCCCTCG TGCGCTCTCC TGTTCCGACC
7081 CTGCCGCTTA CCGGATACCT GTCCGCCTTT CTCCCTTCGG GAAGCGTGGC GCTTTCTCAT
7141 AGCTCACGCT GTAGGTATCT CAGTTCGGTG TAGGTCGTTC GCTCCAAGCT GGGCTGTGTG
7201 CACGAACCCC CCGTTCAGCC CGACCGCTGC GCCTTATCCG GTAACTATCG TCTTGAGTCC
7261 AACCCGGTAA GACACGACTT ATCGCCACTG GCAGCAGCCA CTGGTAACAG GATTAGCAGA
7321 GCGAGGTATG TAGGCGGTGC TACAGAGTTC TTGAAGTGGT GGCCTAACTA CGGCTACACT
7381 AGAAGGACAG TATTTGGTAT CTGCGCTCTG CTGAAGCCAG TTACCTTCGG AAAAAGAGTT
7441 GGTAGCTCTT GATCCGGCAA ACAAACCACC GCTGGTAGCG GTGGTTTTTT TGTTTGCAAG
7501 CAGCAGATTA CGCGCAGAAA AAAAGGATCT CAAGAAGATC CTTTGATCTT TTCTACGGGG
7561 TCTGACGCTC AGTGGAACGA AAACTCACGT TAAGGGATTT TGGTCATGAG ATTATCAAAA
7621 AGGATCTTCA CCTAGATCCT TTTAAATTAA AAATGAAGTT TTAAATCAAT CTAAAGTATA
7681 TATGAGTAAA CTTGGTCTGA CAGTTACCAA TGCTTAATCA GTGAGGCACC TATCTCAGCG
7741 ATCTGTCTAT TTCGTTCATC CATAGTTGCC TGACTCCCCG TCGTGTAGAT AACTACGATA
7801 CGGGAGGGCT TACCATCTGG CCCCAGTGCT GCAATGATAC CGCGAGACCC ACGCTCACCG
7861 GCTCCAGATT TATCAGCAAT AAACCAGCCA GCCGGAAGGG CCGAGCGCAG AAGTGGTCCT
7921 GCAACTTTAT CCGCCTCCAT CCAGTCTATT AATTGTTGCC GGGAAGCTAG AGTAAGTAGT
7981 TCGCCAGTTA ATAGTTTGCG CAACGTTGTT GCCATTGCTA CAGGCATCGT GGTGTCACGC
8041 TCGTCGTTTG GTATGGCTTC ATTCAGCTCC GGTTCCCAAC GATCAAGGCG AGTTACATGA
8101 TCCCCCATGT TGTGCAAAAA AGCGGTTAGC TCCTTCGGTC CTCCGATCGT TGTCAGAAGT
8161 AAGTTGGCCG CAGTGTTATC ACTCATGGTT ATGGCAGCAC TGCATAATTC TCTTACTGTC
```

```
     8221 ATGCCATCCG TAAGATGCTT TTCTGTGACT GGTGAGTACT CAACCAAGTC ATTCTGAGAA
     8281 TAGTGTATGC GGCGACCGAG TTGCTCTTGC CCGGCGTCAA TACGGGATAA TACCGCGCCA
     8341 CATAGCAGAA CTTTAAAAGT GCTCATCATT GGAAAACGTT CTTCGGGGCG AAAACTCTCA
     8401 AGGATCTTAC CGCTGTTGAG ATCCAGTTCG ATGTAACCCA CTCGTGCACC CAACTGATCT
     8461 TCAGCATCTT TTACTTTCAC CAGCGTTTCT GGGTGAGCAA AAACAGGAAG GCAAAATGCC
     8521 GCAAAAAAGG GAATAAGGGC GACACGGAAA TGTTGAATAC TCATACTCTT CCTTTTTCAA
     8581 TATTATTGAA GCATTTATCA GGGTTATTGT CTCATGAGCG GATACATATT TGAATGTATT
     8641 TAGAAAAATA AACAAATAGG GGTTCCGCGC ACATTTCCCC GAAAAGTGCC AC
//
```

<u>pENTR R4-vas2-integrase-R3 (helper plasmid expressing phage ΦC31 integrase under the control of the vasa promoter). Kanamycin resistant</u>

```
LOCUS       pENTRR4-Vas2-INT-R3        6847 bp    DNA     circular
FEATURES             Location/Qualifiers
     misc_feature    complement(603..727)
                     /dnas_title="attR4"
                     /vntifkey=21
                     /label=attR4
                     /note="attR4"
     misc_feature    6052..6176
                     /dnas_title="attR3"
                     /vntifkey=21
                     /label=attR3
                     /note="attR3"
     misc_feature    complement(6295..6313)
                     /dnas_title="pDONR-RP"
                     /vntifkey=21
                     /label=pDONR-RP
                     /note="pDONR-RP"
     misc_feature    537..552
                     /dnas_title="M13F"
                     /vntifkey=21
                     /label=M13F
                     /note="M13F"
     misc_feature    760..3050
                     /dnas_title="Vasa promoter"
                     /vntifkey=21
                     /label=Vasa promoter
     misc_feature    5046..6046
                     /dnas_title="3'UTR_Vasa"
                     /vntifkey=21
                     /label=3'UTR_Vasa
     misc_feature    3213..5030
                     /note="integrase"
     misc_feature    3157..3181
                     /note="HA"
     misc_feature    3121..3134
                     /note="NLS"
     misc_feature    complement(6251..6269)
                     /note="M13R"

BASE COUNT     1986 a   1866 c   2060 g   2078 t
ORIGIN
        1 ctttcctgcg ttatcccctg attctgtgga taaccgtatt accgcctttg agtgagctga
       61 taccgctcgc cgcagccgaa cgaccgagcg cagcgagtca gtgagcgagg aagcggaaga
      121 gcgcccaata cgcaaaccgc ctctccccgc gcgttggccg attcattaat gcagctggca
      181 cgacaggttt cccgactgga aagcgggcag tgagcgcaac gcaattaata cgcgtaccgc
      241 tagccaggaa gagtttgtag aaacgcaaaa aggccatccg tcaggatggc cttctgctta
      301 gtttgatgcc tggcagttta tggcgggcgt cctgcccgcc accctccggg ccgttgcttc
      361 acaacgttca aatccgctcc cggcggattt gtcctactca ggagagcgtt caccgacaaa
```

```
 421 caacagataa aacgaaaggc ccagtcttcc gactgagcct ttcgttttat ttgatgcctg
 481 gcagttccct actctcgcgt taacgctagc atggatgttt tcccagtcac gacgttgtaa
 541 aacgacggcc agtcttaagc tcgggcccct acaggtcact aataccatct aagtagttga
 601 ttcatagtga ctggatatgt tgtgttttac agtattatgt agtctgtttt ttatgcaaaa
 661 tctaatttaa tatattgata tttatatcat tttacgtttc tcgttcaact tttctataca
 721 aagttggtac cgggcccccc gctagcgtcg acggtatcga tgtagaacgc gagcaaattc
 781 ttttccttcc atgacagcag cagctacagt gggaagccga acgtcagacg tgtttgacat
 841 gccgaactgg gcgggaaaat tacagcgtgc gctttgtttt caagcaaatc acaactcgct
 901 gcaaacaaaa ccgttgagaa attgattgtt ttataatttg tattgtattt tatttgttat
 961 aataaactaa aaagacatac tttttgcata ttttatacat aaaaacatac atgcagcatt
1021 ataaaacaca tataaaccct ccctgtagag tcccgtatcg aaatcttcca tcctagttgc
1081 acagtacgac ggacgagtag gccgtgtccg tgcaaattcc agcttttagc agtcttttgc
1141 tcggagcact cgcggcgagt cggaggtttc tgctgaggtg cttagcgcta aattagccaa
1201 ttgcttttgc aagtgaaata accagccgaa tagtacttca aaactcaggt aagtgaacta
1261 gttttataga acaaatgttt gtttgttaga agttagtgaa gtgtttgtga aaaaaatctc
1321 tcatttcggc aaaactaacg taactgattt caaattgaat tattgttttg tgatgttata
1381 ttatttcatc cagttgatta gtattttctt agttatgttc aaaatacagt taaattaaat
1441 ttcatttcat ttactcataa aataatctct tggcttattt aatttttctc gaattcgctt
1501 gtattgttca gtagcacgcg ccattcgccc tttgtttcat tttgtacctg ctcccactaa
1561 cacactggca gtgcgaaaca aaagccttcg cacgcgttgc tggtattaga gtgtgtgcgt
1621 gtgtgtgttg agcgctctgt caaaatcggc tgttgccgcc ggtaccgaaa ttgcctgttc
1681 gcacgctgtt cgtaaacatt ccgtggtgtg tatcgtgtgt tgtgcatgtt gcgcgcctcc
1741 ccccttttga tagcaggctg ccgtggctgc cgtggtgtgt ggcgcagttg agtttttgga
1801 ttaattttct aaggaaatgg cacgagaaga gcggtggcag tgtgttggtt tgctctgtcc
1861 cttcctttct gtgtgaagtg ttcttacagc acagcacgta tccaccaccg cacacagagc
1921 aggcaaggaa gtggaagtga acaagtgtgc tgcgcatgca tgtgtgtggg gggcatttta
1981 gctgagatcg tcgttatttg agaagcggta taggggccag tcggtgtcga cgtacggaag
2041 cggtttagtt ttaatccaag cgtatcccgt cgtggagtgg ttgtgtggct ctgtgtgctc
2101 tcatatcagt tccagagtga ggttagtaga atcacagtcc ttggcctttt tcgttacaag
2161 atatccagaa ggatggcgtt atttccacag cttaccatgg tgctcttgtt tgctcgaatc
2221 aggggagaaa aacagtttcg tgtttcatga accgcagttg gcactggagc ggattcaaaa
2281 gtcttcgata tgcaatagat aagagagtcg ttggggcata gttgggaagc ctttccgaga
2341 tgtggagttt ccgagaggag aaatggtgct ttcgtgcacg ttccgggaca gcgggccccg
2401 cgaagagcat ctcgttgtcg ttcatccggc aataattgat gcgaaaagcg cgcgcgccac
2461 tggcttagcg cagtgtacac agtgatattc acctacacac acagaggcac acgccttcac
2521 acgcgcgcgt gcttcaaagg ctacttcggt ggcggtgtgt gaggtcgctt gcaatggaca
2581 atgaaaattt cgctggaaaa taccatcgtc tctttaggtt gcaatgggtg cgggtagagc
2641 ggtggtcgtc gatattggtg gtgtagtgtg tgtgtgtgtg tgtgtgtgtg tgtgtgtgtg
2701 tgtgtgtgtg tgtgtgtgtg tgtgtgtgtg tgtgtgtgtg tgtgtgtgtg tgtgtgtgtg
2761 tgcaacggca attatttttt gtaatatttc gaccatcttt ctttctctct ctccacgtgc
2821 tgctgctgtt gctgctgctg ctgcattgca tgttccacta ttcctctcgg tttgtgcctg
2881 cggacgccat tgctagtcga aagagagtcg ccgttagtcg cgcttcgagc aacggacacg
2941 tttttttggtt gaaaccaaca gctttttttca tcttcgggag acacacagat ctcgaatcgt
3001 acattcccat aaggagaatt gtcatcttcc ggtgaataaa gaaaggaaac ggatccggaa
3061 acatgggatc atcatcagac gacgaagcta cggccgatgc gcagcatgca gcaccaccaa
3121 aaaaaaaacg aaaagtagaa gacccacgat ttatgtaccc atacgatgtt cctgactatg
3181 cgggtatgaa aaacatcaaa aaaaaccagg tTATGGACAC GTACGCGGGT GCTTACGACC
3241 GTCAGTCGCG CGAGCGCGAA AATTCGAGCG CAGCAAGCCC AGCGACACAG CGTAGCGCCA
3301 ACGAAGACAA GGCGGCCGAC CTTCAGCGCG AAGTCGAGCG CGACGGGGGC CGGTTCAGGT
3361 TCGTCGGGCA TTTCAGCGAA GCGCCGGGCA CGTCGGCGTT CGGGACGGCG GAGCGCCCGG
3421 AGTTCGAACG CATCCTGAAC GAATGCCGCG CCGGGCGGCT CAACATGATC ATTGTCTATG
3481 ACGTGTCGCG CTTCTCGCGC CTGAAGGTCA TGGACGCGAT TCCGATTGTC TCGGAATTGC
3541 TCGCCCTGGG CGTGACGATT GTTTCCACTC AGGAAGGCGT CTTCCGGCAG GGAAACGTCA
3601 TGGACCTGAT TCACCTGATT ATGCGGCTCG ACGCGTCGCA CAAAGAATCT TCGCTGAAGT
3661 CGGCGAAGAT TCTCGACACG AAGAACCTTC AGCGCGAATT GGGCGGGTAC GTCGGCGGGA
3721 AGGCGCCTTA CGGCTTCGAG CTTGTTTCGG AGACGAAGGA GATCACGCGC AACGGCCGAA
3781 TGGTCAATGT CGTCATCAAC AAGCTTGCGC ACTCGACCAC TCCCCTTACC GGACCCTTCG
3841 AGTTCGAGCC CGACGTAATC CGGTGGTGGT GGCGTGAGAT CAAGACGCAC AAACACCTTC
3901 CCTTCAAGCC GGGCAGTCAA GCCGCCATTC ACCCGGGCAG CATCACGGGG CTTTGTAAGC
3961 GCATGGACGC TGACGCCGTG CCGACCCGGG GCGAGACGAT TGGGAAGAAG ACCGCTTCAA
4021 GCGCCTGGGA CCCGGCAACC GTTATGCGAA TCCTTCGGGA CCCGCGTATT GCGGGCTTCG
4081 CCGCTGAGGT GATCTACAAG AAGAAGCCGG ACGGCACGCC GACCACGAAG ATTGAGGGTT
4141 ACCGCATTCA GCGCGACCCG ATCACGCTCC GGCCGGTCGA GCTTGATTGC GGACCGATCA
4201 TCGAGCCCGC TGAGTGGTAT GAGCTTCAGG CGTGGTTGGA CGGCAGGGGG CGCGGCAAGG
4261 GGCTTTCCCG GGGGCAAGCC ATTCTGTCCG CCATGGACAA GCTGTACTGC GAGTGTGGCG
```

```
4321 CCGTCATGAC TTCGAAGCGC GGGGAAGAAT CGATCAAGGA CTCTTACCGC TGCCGTCGCC
4381 GGAAGGTGGT CGACCCGTCC GCACCTGGGC AGCACGAAGG CACGTGCAAC GTCAGCATGG
4441 CGGCACTCGA CAAGTTCGTT GCGGAACGCA TCTTCAACAA GATCAGGCAC GCCGAAGGCG
4501 ACGAAGAGAC GTTGGCGCTT CTGTGGGAAG CCGCCCGACG CTTCGGCAAG CTCACTGAGG
4561 CGCCTGAGAA GAGCGGCGAA CGGGCGAACC TTGTTGCGGA GCGCGCCGAC GCCCTGAACG
4621 CCCTTGAAGA GCTGTACGAA GACCGCGCGG CAGGCGCGTA CGACGGACCC GTTGGCAGGA
4681 AGCACTTCCG GAAGCAACAG GCAGCGCTGA CGCTCCGGCA GCAAGGGGCG GAAGAGCGGC
4741 TTGCCGAACT TGAAGCCGCC GAAGCCCCGA AGCTTCCCCT TGACCAATGG TTCCCCGAAG
4801 ACGCCGACGC TGACCCGACC GGCCCTAAGT CGTGGTGGGG GCGCGCGTCA GTAGACGACA
4861 AGCGCGTGTT CGTCGGGCTC TTCGTAGACA AGATCGTTGT CACGAAGTCG ACTACGGGCA
4921 GGGGGCAGGG AACGCCCATC GAGAAGCGCG CTTCGATCAC GTGGGCGAAG CCGCCGACCG
4981 ACGACGACGA AGACGACGCC CAGGACGGCA CGGAAGACGT AGCGGCGTAG tgtacCCCcc
5041 tcgagcttgg ggtggggttg ttatgtgttg cgaacgagag tggatctctc tcgacatttc
5101 cttatttttt ttttctattg ttaacactta caacgaaact tcggaagaga agtttcctca
5161 ttcgaaacga ggagtcaaac tcctttcctt gcttttgtga catgcatgat tattctttca
5221 tttactgacg taacgatgta aaacacacag aagaagcatg acacacagca aagaattgtt
5281 cgttaataaa acgttgatga aaactttgaa aacataagaa cttgcacttt tattctataa
5341 ttcgtgaaag cttgcaccga ttgtctttca ttattcaatg taatgtactg aaaggtgatt
5401 tttcgcactt gtatactcta gaactgaagt attctaacaa tacgtcacct ttaggtccat
5461 tccaggataa aatacacaag tgaaggtagt tgtacaaagt acttagacta acccaggttt
5521 caaaaaagat aacgacgaat tgagcgtaac tgcacaaagc ccgttcattg tcaacatatc
5581 gttcaggggt cttcaaactt tattatcagc ccgcgggccg caacaacgtt gcttagagca
5641 ccatcttgat gtatccttta tctagtggct tatcgtttat gtctcgtttg gctaagaaaa
5701 tactaataac atattcaatt tacggagcgg taccatggta caaacgtcat actgctagac
5761 ttgacaacat gcccatcgag ggttcttacc tcaaatggac cgatcccttg taactggcgg
5821 cgtaaactca gtaaattcca aaagccagta taggccacca tgaccgtcta actattccaa
5881 aaaggagaat atttgaaatt tctatatcta tagtattatt actatatcta tatgtttctt
5941 tagtacaata ttagagcttg attattttcc tgaaaattgt aaaatatgca tgttagatgg
6001 ttgatacatc gttttaaaaa aaactgctgt taatggccac attttctcga gacaactttg
6061 tataataaag ttgaacgaga aacgtaaaat gatataaata tcaatatatt aaattagatt
6121 ttgcataaaa aacagactac ataatactgt aaaacacaac atatccagtc actatgaatc
6181 aactacttag atggtattag tgacctgtac tgcagggcgg ccgcgatatc ccctatagtg
6241 agtcgtatta catggtcata gctgtttcct ggcagctctg gcccgtgtct caaaatctct
6301 gatgttacat tgcacaagat aaaaatatat catcatgaac aataaaactg tctgcttaca
6361 taaacagtaa tacaaggggt gttatgagcc atattcaacg ggaaacgtcg aggccgcgat
6421 taaattccaa catggatgct gatttatatg ggtataaatg ggctcgcgat aatgtcgggc
6481 aatcaggtgc gacaatctat cgcttgtatg ggaagcccga tgcgccagag ttgtttctga
6541 aacatggcaa aggtagcgtt gccaatgatg ttacagatga gatggtcaga ctaaactggc
6601 tgacggaatt tatgcctctt ccgaccatca agcattttat ccgtactcct gatgatgcat
6661 ggttactcac cactgcgatc cccggaaaaa cagcattcca ggtattagaa gaatatcctg
6721 attcaggtga aaatattgtt gatgcgctgg cagtgttcct gcgccggttg cattcgattc
6781 ctgtttgtaa ttgtcctttt aacagcgatc gcgtatttcg tctcgctcag gcgcaatcac
6841 gaatgaataa cggtttggtt gatgcgagtg attttgatga cgagcgtaat ggctggcctg
6901 ttgaacaagt ctggaaagaa atgcataaac ttttgccatt ctcaccggat tcagtcgtca
6961 ctcatggtga tttctcactt gataacctta tttttgacga ggggaaatta ataggttgta
7021 ttgatgttgg acgagtcgga atcgcagacc gataccagga tcttgccatc ctatggaact
7081 gcctcggtga gttttctcct tcattacaga aacggctttt tcaaaaatat ggtattgata
7141 atcctgatat gaataaattg cagtttcatt tgatgctcga tgagtttttc taatcagaat
7201 tggttaattg gttgtaacac tggcagagca ttacgctgac ttgacgggac ggcgcaagct
7261 catgaccaaa atcccttaac gtgagttacg cgtcgttcca ctgagcgtca gaccccgtag
7321 aaaagatcaa aggatcttct tgagatcctt tttttctgcg cgtaatctgc tgcttgcaaa
7381 caaaaaaacc accgctacca gcggtggttt gtttgccgga tcaagagcta ccaactcttt
7441 ttccgaaggt aactggcttc agcagagcgc agataccaaa tactgttctt ctagtgtagc
7501 cgtagttagg ccaccacttc aagaactctg tagcaccgcc tacatacctc gctctgctaa
7561 tcctgttacc agtggctgct gccagtggcg ataagtcgtg tcttaccggg ttggactcaa
7621 gacgatagtt accggataag gcgcagcggt cgggctgaac ggggggttcg tgcacacagc
7681 ccagcttgga gcgaacgacc tacaccgaac tgagatacct acagcgtgag ctatgagaaa
7741 gcgccacgct tcccgaaggg agaaaggcgg acaggtatcc ggtaagcggc agggtcggaa
7801 caggagagcg cacgagggag cttccagggg gaaacgcctg gtatctttat agtcctgtcg
7861 ggtttcgcca cctctgactt gagcgtcgat ttttgtgatg ctcgtcaggg gggcggagcc
7921 tatggaaaaa cgccagcaac gcggcctttt tacggttcct ggccttttgc tggccttttg
7981 ctcacatgtt
//
```

pENTR R4-vas2-Transposase-R3 (helper plasmid expressing *piggyBac* transposase under the control of the vasa promoter). Kanamycin resistant

```
LOCUS       pENTRR4-Vas2-Transp-R3        6847 bp    DNA     circular
FEATURES             Location/Qualifiers
     misc_feature    complement(603..727)
                     /dnas_title="attR4"
                     /vntifkey="21"
                     /label=attR4
                     /note="attR4"
     misc_feature    5945..6069
                     /dnas_title="attR3"
                     /vntifkey="21"
                     /label=attR3
                     /note="attR3"
     misc_feature    complement(6188..6206)
                     /dnas_title="pDONR-RP"
                     /vntifkey="21"
                     /label=pDONR-RP
                     /note="pDONR-RP"
     misc_feature    537..552
                     /dnas_title="M13F"
                     /vntifkey="21"
                     /label=M13F
                     /note="M13F"
     misc_feature    760..3050
                     /dnas_title="Vasa promoter"
                     /vntifkey="21"
                     /label=Vasa promoter
     misc_feature    4939..5939
                     /dnas_title="3'UTR_Vasa"
                     /vntifkey="21"
                     /label=3'UTR_Vasa
     misc_feature    3068..4852
                     /note="transposase ORF"
     misc_feature    complement(6144..6162)
                     /note="M13R"
     misc_feature    complement(6124..6143)
                     /note="T7"

ORIGIN
        1 ctttcctgcg ttatcccctg attctgtgga taaccgtatt accgcctttg agtgagctga
       61 taccgctcgc cgcagccgaa cgaccgagcg cagcgagtca gtgagcgagg aagcggaaga
      121 gcgcccaata cgcaaaccgc ctctccccgc gcgttggccg attcattaat gcagctggca
      181 cgacaggttt cccgactgga aagcgggcag tgagcgcaac gcaattaata cgcgtaccgc
      241 tagccaggaa gagtttgtag aaacgcaaaa aggccatccg tcaggatggc cttctgctta
      301 gtttgatgcc tggcagttta tggcgggcgt cctgcccgcc accctccggg ccgttgcttc
      361 acaacgttca aatccgctcc cggcggattt gtcctactca ggagagcgtt caccgacaaa
      421 caacagataa aacgaaaggc ccagtcttcc gactgagcct ttcgttttat ttgatgcctg
      481 gcagttccct actctcgcgt taacgctagc atggatgttt tcccagtcac gacgttgtaa
      541 aacgacggcc agtcttaagc tcgggcccct acaggtcact aataccatct aagtagttga
      601 ttcatagtga ctggatatgt tgtgttttac agtattatgt agtctgtttt ttatgcaaaa
      661 tctaatttaa tatattgata tttatatcat tttacgtttc tcgttcaact tttctataca
      721 aagttggtac cgggcccccc gctagcgtcg acggtatcga tgtagaacgc gagcaaattc
      781 ttttccttcc atgacagcag cagctacagt gggaagccga acgtcagacg tgtttgacat
      841 gccgaactgg gcgggaaaat tacagcgtgc gctttgtttt caagcaaatc acaactcgct
      901 gcaaacaaaa ccgttgagaa attgattgtt ttataatttg tattgtattt tatttgttat
      961 aataaactaa aaagacatac tttttgcata ttttatacat aaaaacatac atgcagcatt
     1021 ataaaacaca tataaaccct ccctgtagag tcccgtatcg aaatcttcca tcctagttgc
     1081 acagtacgac ggacgagtag gccgtgtccg tgcaaattcc agcttttagc agtcttttgc
     1141 tcggagcact cgcggcgagt cggaggtttc tgctgaggtg cttagcgcta aattagccaa
     1201 ttgcttttgc aagtgaaata accagccgaa tagtacttca aaactcaggt aagtgaacta
     1261 gttttataga acaaatgttt gtttgttaga agttagtgaa gtgtttgtga aaaaaatctc
     1321 tcatttcggc aaaactaacg taactgattt caaattgaat tattgttttg tgatgttata
```

1381 ttatttcatc cagttgatta gtattttctt agttatgttc aaaatacagt taaattaaat
1441 ttcatttcat ttactcataa aataatctct tggcttattt aatttttctc gaattcgctt
1501 gtattgttca gtagcacgcg ccattcgccc tttgtttcat tttgtacctg ctcccactaa
1561 cacactggca gtgcgaaaca aaagccttcg cacgcgttgc tggtattaga gtgtgtgcgt
1621 gtgtgtgttg agcgctctgt caaaatcggc tgttgccgcc ggtaccgaaa ttgcctgttc
1681 gcacgctgtt cgtaaacatt ccgtggtgtg tatcgtgtgt tgtgcatgtt gcgcgcctcc
1741 cccctttga tagcaggctg ccgtggctgc cgtggtgtgt ggcgcagttg agtttttgga
1801 ttaattttct aaggaaatgg cacgagaaga gcggtggcag tgtgttggtt tgctctgtcc
1861 cttcctttct gtgtgaagtg ttcttacagc acagcacgta tccaccaccg cacacagagc
1921 aggcaaggaa gtggaagtga acaagtgtgc tgcgcatgca tgtgtgtggg gggcatttta
1981 gctgagatcg tcgttatttg agaagcggta taggggccag tcggtgtcga cgtacggaag
2041 cggtttagtt ttaatccaag cgtatcccgt cgtggagtgg ttgtgtggct ctgtgtgctc
2101 tcatatcagt tccagagtga ggttagtaga atcacagtcc ttggcctttt tcgttacaag
2161 atatccagaa ggatggcgtt atttccacag cttaccatgg tgctcttgtt tgctcgaatc
2221 aggggagaaa aacagtttcg tgtttcatga accgcagttg gcactggagc ggattcaaaa
2281 gtcttcgata tgcaatagat aagagagtcg ttggggcata gttgggaagc ctttccgaga
2341 tgtggagttt ccgagaggag aaatggtgct ttcgtgcacg ttccgggaca gcgggccccg
2401 cgaagagcat ctcgttgtcg ttcatccggc aataattgat gcgaaaagcg cgcgcgccac
2461 tggcttagcg cagtgtacac agtgatattc acctacacac acagaggcac acgccttcac
2521 acgcgcgcgt gcttcaaagg ctacttcggt ggcggtgtgt gaggtcgctt gcaatggaca
2581 atgaaaattt cgctggaaaa taccatcgtc tctttaggtt gcaatgggtg cgggtagagc
2641 ggtggtcgtc gatattggtg gtgtagtgtg tgtgtgtgtg tgtgtgtgtg tgtgtgtgtg
2701 tgtgtgtgtg tgtgtgtgtg tgtgtgtgtg tgtgtgtgtg tgtgtgtgtg tgtgtgtgtg
2761 tgcaacggca attatttttt gtaatatttc gaccatcttt ctttctctct ctccacgtgc
2821 tgctgctgtt gctgctgctg ctgcattgca tgttccacta ttcctctcgg tttgtgcctg
2881 cggacgccat tgctagtcga aagagagtcg ccgttagtcg cgcttcgagc aacggacacg
2941 tttttggtt gaaaccaaca gctttttca tcttcgggag acacacagat ctcgaatcgt
3001 acattcccat aaggagaatt gtcatcttcc ggtgaataaa gaaaggaaac gGATCCTATA
3061 TAATAAAATG GGTAGTTCTT TAGACGATGA GCATATCCTC TCTGCTCTTC TGCAAAGCGA
3121 TGACGAGCTT GTTGGTGAGG ATTCTGACAG TGAAATATCA GATCACGTAA GTGAAGATGA
3181 CGTCCAGAGC GATACAGAAG AAGCGTTTAT AGATGAGGTA CATGAAGTGC AGCCAACGTC
3241 AAGCGGTAGT GAAATATTAG ACGAACAAAA TGTTATTGAA CAACCAGGTT CTTCATTGGC
3301 TTCTAACAGA ATCTTGACCT TGCCACAGAG GACTATTAGA GGTAAGAATA AACATTGTTG
3361 GTCAACTTCA AAGTCCACGA GGCGTAGCCG AGTCTCTGCA CTGAACATTG TCAGATCTCA
3421 AAGAGGTCCG ACGCGTATGT GCCGCAATAT ATATGACCCA CTTTTATGCT TCAAACTATT
3481 TTTTACTGAT GAGATAATTT CGGAAATTGT AAAATGGACA AATGCTGAGA TATCATTGAA
3541 ACGTCGGGAA TCTATGACAG GTGCTACATT TCGTGACACG AATGAAGATG AAATCTATGC
3601 TTTCTTTGGT ATTCTGGTAA TGACAGCAGT GAGAAAAGAT AACCACATGT CCACAGATGA
3661 CCTCTTTGAT CGATCTTTGT CAATGGTGTA CGTCTCTGTA ATGAGTCGTG ATCGTTTTGA
3721 TTTTTTGATA CGATGTCTTA GAATGGATGA CAAAAGTATA CGGCCCACAC TTCGAGAAAA
3781 CGATGTATTT ACTCCTGTTA GAAAAATATG GGATCTCTTT ATCCATCAGT GCATACAAAA
3841 TTACACTCCA GGGGCTCATT TGACCATAGA TGAACAGTTA CTTGGTTTTA GAGGACGGTG
3901 TCCGTTTAGG ATGTATATCC CAAACAAGCC AAGTAAGTAT GGAATAAAAA TCCTCATGAT
3961 GTGTGACAGT GGTACGAAGT ATATGATAAA TGGAATGCCT TATTTGGGAA GAGGAACACA
4021 GACCAACGGA GTACCACTCG GTGAATACTA CGTGAAGGAG TTATCAAAGC CTGTGCACGG
4081 TAGTTGTCGT AATATTACGT GTGACAATTG GTTCACCTCA ATCCCTTTaG CAAAAAACTT
4141 ACTACAAGAA CCGTATAAGT TAACCATTGT GGGAACCGTG CGATCAAACA AACGCGAGAT
4201 ACCGGAAGTA CTGAAAAACA GTCGCTCCAG GCCAGTGGGA ACATCGATGT TTTGTTTTGA
4261 CGGACCCCTT ACTCTCGTCT CATATAAACC GAAGCCAGCT AAGATGGTAT ACTTATTATC
4321 ATCTTGTGAT GAGGATGCTT CTATCAACGA AAGTACCGGT AAACCGCAAA TGGTTATGTA
4381 TTATAATCAA ACTAAAGGCG GAGTGGACAC GCTAGACCAA ATGTGTTCTG TGATGACCTG
4441 CAGTAGGAAG ACGAATAGGT GGCCTATGGC ATTATTGTAC GGAATGATAA ACATTGCCTG
4501 CATAAATTCT TTTATTATAT ACAGCCATAA TGTCAGTAGC AAGGGAGAAA AGGTTCAAAG
4561 TCGCAAAAAA TTTATGAGAA ACCTTTACAT GAGCCTGACG TCATCGTTTA TGCGTAAGCG
4621 TTTAGAAGCT CCTACTTTGA AGAGATATTT GCGCGATAAT ATCTCTAATA TTTTGCCAAA
4681 TGAAGTGCCT GGTACATCAG ATGACAGTAC TGAAGAGCCA GTAATGAAAA AACGTACTTA
4741 CTGTACTTAC TGCCCCTCTA AAATAAGGCG AAAGGCAAAT GCATCGTGCA AAAAATGCAA
4801 AAAAGTTATT TGTCGAGAGC ATAATATTGA TATGTGCCAA AGTTGTTTCT GACTGACTAA
4861 TAAGTATAAT TTGTTTCTAT TATGTATAAG TTAAgCTAAT TACTTATTTT ATAATACAAC
4921 ATGACTGTTT TTctcgagct tggggtgggg ttgttatgtg ttgcgaacga gagtggatct
4981 ctctcgacat ttccttattt tttttttcta ttgttaacac ttacaacgaa acttcggaag
5041 agaagtttcc tcattcgaaa cgaggagtca aactcctttc cttgcttttg tgacatgcat
5101 gattattctt tcatttactg acgtaacgat gtaaaacaca cagaagaagc atgacacaca
5161 gcaaagaatt gttcgttaat aaaacgttga tgaaaacttt gaaaacataa gaacttgcac
5221 ttttattcta taattcgtga aagcttgcac cgattgtctt tcattattca atgtaatgta

```
        5281 ctgaaaggtg atttttcgca cttgtatact ctagaactga agtattctaa caatacgtca
        5341 cctttaggtc cattccagga taaaatacac aagtgaaggt agttgtacaa agtacttaga
        5401 ctaacccagg tttcaaaaaa gataacgacg aattgagcgt aactgcacaa agcccgttca
        5461 ttgtcaacat atcgttcagg ggtcttcaaa ctttattatc agcccgcggg ccgcaacaac
        5521 gttgcttaga gcaccatctt gatgtatcct ttatctagtg gcttatcgtt tatgtctcgt
        5581 ttggctaaga aaatactaat aacatattca atttacggag cggtaccatg gtacaaacgt
        5641 catactgcta gacttgacaa catgcccatc gagggttctt acctcaaatg gaccgatccc
        5701 ttgtaactgg cggcgtaaac tcagtaaatt ccaaaagcca gtataggcca ccatgaccgt
        5761 ctaactattc caaaaaggag aatatttgaa atttctatat ctatagtatt attactatat
        5821 ctatatgttt ctttagtaca atattagagc ttgattattt tcctgaaaat tgtaaaatat
        5881 gcatgttaga tggttgatac atcgttttaa aaaaaactgc tgttaatggc cacattttct
        5941 cgagacaact ttgtataata aagttgaacg agaaacgtaa aatgatataa atatcaatat
        6001 attaaattag attttgcata aaaaacagac tacataatac tgtaaaacac aacatatcca
        6061 gtcactatga atcaactact tagatggtat tagtgacctg tactgcaggg cggccgcgat
        6121 atcccctata gtgagtcgta ttacatggtc atagctgttt cctggcagct ctggcccgtg
        6181 tctcaaaatc tctgatgtta cattgcacaa gataaaaata tatcatcatg aacaataaaa
        6241 ctgtctgctt acataaacag taatacaagg ggtgttatga gccatattca acgggaaacg
        6301 tcgaggccgc gattaaattc caacatggat gctgatttat atgggtataa atgggctcgc
        6361 gataatgtcg ggcaatcagg tgcgacaatc tatcgcttgt atgggaagcc cgatgcgcca
        6421 gagttgtttc tgaaacatgg caaaggtagc gttgccaatg atgttacaga tgagatggtc
        6481 agactaaact ggctgacgga atttatgcct cttccgacca tcaagcattt tatccgtact
        6541 cctgatgatg catggttact caccactgcg atccccggaa aaacagcatt ccaggtatta
        6601 gaagaatatc ctgattcagg tgaaaatatt gttgatgcgc tggcagtgtt cctgcgccgg
        6661 ttgcattcga ttcctgtttg taattgtcct tttaacagcg atcgcgtatt tcgtctcgct
        6721 caggcgcaat cacgaatgaa taacggtttg gttgatgcga gtgattttga tgacgagcgt
        6781 aatggctggc ctgttgaaca agtctggaaa gaaatgcata aacttttgcc attctcaccg
        6841 gattcagtcg tcactcatgg tgatttctca cttgataacc ttatttttga cgaggggaaa
        6901 ttaataggtt gtattgatgt tggacgagtc ggaatcgcag accgatacca ggatcttgcc
        6961 atcctatgga actgcctcgg tgagttttct ccttcattac agaaacggct ttttcaaaaa
        7021 tatggtattg ataatcctga tatgaataaa ttgcagtttc atttgatgct cgatgagttt
        7081 ttctaatcag aattggttaa ttggttgtaa cactggcaga gcattacgct gacttgacgg
        7141 gacggcgcaa gctcatgacc aaaatccctt aacgtgagtt acgcgtcgtt ccactgagcg
        7201 tcagaccccg tagaaaagat caaaggatct tcttgagatc ctttttttct gcgcgtaatc
        7261 tgctgcttgc aaacaaaaaa accaccgcta ccagcggtgg tttgtttgcc ggatcaagag
        7321 ctaccaactc tttttccgaa ggtaactggc ttcagcagag cgcagatacc aaatactgtt
        7381 cttctagtgt agccgtagtt aggccaccac ttcaagaact ctgtagcacc gcctacatac
        7441 ctcgctctgc taatcctgtt accagtggct gctgccagtg gcgataagtc gtgtcttacc
        7501 gggttggact caagacgata gttaccggat aaggcgcagc ggtcgggctg aacggggggt
        7561 tcgtgcacac agcccagctt ggagcgaacg acctacaccg aactgagata cctacagcgt
        7621 gagctatgag aaagcgccac gcttcccgaa gggagaaagg cggacaggta tccggtaagc
        7681 ggcagggtcg gaacaggaga gcgcacgagg gagcttccag ggggaaacgc ctggtatctt
        7741 tatagtcctg tcgggtttcg ccacctctga cttgagcgtc gatttttgtg atgctcgtca
        7801 gggggcgga gcctatggaa aaacgccagc aacgcggcct ttttacggtt cctggccttt
        7861 tgctggcctt ttgctcacat gtt
//
```

<u>pENTR L1-vas2-Cre-L4 (helper plasmid expressing *Cre recombinase* under the control of the vasa promoter). Kanamycin resistant</u>

```
LOCUS       pENTR L1-vasaCre-L4                7138 bp    DNA     circular
FEATURES             Location/Qualifiers
     misc_feature    complement(5443..5461)
                     /note="DONR-RP"
     misc_feature    570..665
                     /note="attL1"
     misc_feature    complement(5253..5348)
                     /note="attL4"
     misc_feature    537..553
                     /note="M13F"
     misc_feature    complement(5399..5417)
```

```
                     /note="M13R"
     misc_feature    703..2991
                     /dnas_title="Vasa promoter"
                     /vntifkey="21"
                     /label=vasa promoter
     misc_feature    4263..5241
                     /dnas_title="3'UTR_Vasa"
                     /vntifkey="21"
                     /label=3'UTR_Vasa
     misc_feature    complement(3061..3074)
                     /note="NLS"
     misc_feature    3098..3123
                     /note="HA epitope"
     misc_feature    3166..4248
                     /note="Cre ORF"

ORIGIN
        1 CTTTCCTGCG TTATCCCCTG ATTCTGTGGA TAACCGTATT ACCGCCTTTG AGTGAGCTGA
       61 TACCGCTCGC CGCAGCCGAA CGACCGAGCG CAGCGAGTCA GTGAGCGAGG AAGCGGAAGA
      121 GCGCCCAATA CGCAAACCGC CTCTCCCCGC GCGTTGGCCG ATTCATTAAT GCAGCTGGCA
      181 CGACAGGTTT CCCGACTGGA AAGCGGGCAG TGAGCGCAAC GCAATTAATA CGCGTACCGC
      241 TAGCCAGGAA GAGTTTGTAG AAACGCAAAA AGGCCATCCG TCAGGATGGC CTTCTGCTTA
      301 GTTTGATGCC TGGCAGTTTA TGGCGGGCGT CCTGCCCGCC ACCCTCCGGG CCGTTGCTTC
      361 ACAACGTTCA AATCCGCTCC CGGCGGATTT GTCCTACTCA GGAGAGCGTT CACCGACAAA
      421 CAACAGATAA AACGAAAGGC CCAGTCTTCC GACTGAGCCT TTCGTTTTAT TTGATGCCTG
      481 GCAGTTCCCT ACTCTCGCGT TAACGCTAGC ATGGATGTTT TCCCAGTCAC GACGTTGTAA
      541 AACGACGGCC AGTCTTAAGC TCGGGCCCCA AATAATGATT TTATTTTGAC TGATAGTGAC
      601 CTGTTCGTTG CAACAAATTG ATGAGCAATG CTTTTTTATA ATGCCAACTT TGTACAAAAA
      661 AGCAGGCTGG TACCGGGCCC CCCGCTAGCG TCGACGGTAT cgatgtagaa cgcgagcaaa
      721 ttcttttcct tccatgacag cagcagctac agtgggaagc cgaacgtcag acgtgtttga
      781 catgccgaac tgggcgggaa aattacagcg tgcgctttgt tttcaagcaa atcacaactc
      841 gctgcaaaca aaaccgttga gaaattgatt gttttataat ttgtattgta ttttatttgt
      901 tataataaac taaaaagaca tactttttgc atattttata cataaaaaca tacatgcagc
      961 attataaaac acatataaac cctccctgta gagtcccgta tcgaaatctt ccatcctagt
     1021 tgcacagtac gacggacgag taggccgtgt ccgtgcaaat tccagctttt agcagtcttt
     1081 tgctcggagc actcgcggcg agtcggaggt ttctgctgag gtgcttagcg ctaaattagc
     1141 caattgcttt tgcaagtgaa ataaccagcc gaatagtact tcaaaactca ggtaagtgaa
     1201 ctagttttat agaacaaatg tttgtttgtt agaagttagt gaagtgtttg tgaaaaaaat
     1261 ctctcatttc ggcaaaacta acgtaactga tttcaaattg aattattgtt ttgtgatgtt
     1321 atattatttc atccagttga ttagtatttt cttagttatg ttcaaaatac agttaaatta
     1381 aatttcattt catttactca taaaataatc tcttggctta tttaattttt ctcgaattcg
     1441 cttgtattgt tcagtagcac gcgccattcg ccctttgttt cattttgtac ctgctcccac
     1501 taacacactg gcagtgcgaa acaaaagcct tcgcacgcgt tgctggtatt agagtgtgtg
     1561 cgtgtgtgtg ttgagcgctc tgtcaaaatc ggctgttgcc gccggtaccg aaattgcctg
     1621 ttcgcacgct gttcgtaaac attccgtggt gtgtatcgtg tgttgtgcat gttgcgcgcc
     1681 tcccccttt tgatagcagg ctgccgtggc tgccgtggtg tgtggcgcag ttgagttttt
     1741 ggattaattt tctaaggaaa tggcacgaga agagcggtgg cagtgtgttg gtttgctctg
     1801 tcccttcctt tctgtgtgaa gtgttcttac agcacagcac gtatccacca ccgcacacag
     1861 agcaggcaag gaagtggaag tgaacaagtg tgctgcgcat gcatgtgtgt gggggcatt
     1921 ttagctgaga tcgtcgttat ttgagaagcg gtataggggc cagtcggtgt cgacgtacgg
     1981 aagcggttta gttttaatcc aagcgtatcc cgtcgtggag tggttgtgtg gctctgtgtg
     2041 ctctcatatc agttccagag tgaggttagt agaatcacag tccttggcct ttttcgttac
     2101 aagatatcca gaaggatggc gttatttcca cagcttacca tggtgctctt gtttgctcga
     2161 atcaggggag aaaaacagtt tcgtgtttca tgaaccgcag ttggcactgg agcggattca
     2221 aaagtcttcg atatgcaata gataagagag tcgttggggc atagttggga agcctttccg
     2281 agatgtggag tttccgagag gagaaatggt gctttcgtgc acgttccggg acagcgggcc
     2341 ccgcgaagag catctcgttg tcgttcatcc ggcaataatt gatgcgaaaa gcgcgcgcgc
     2401 cactggctta gcgcagtgta cacagtgata ttcacctaca cacacagagg cacacgcctt
     2461 cacacgcgcg cgtgcttcaa aggctacttc ggtggcggtg tgtgaggtcg cttgcaatgg
     2521 acaatgaaaa tttcgctgga aaataccatc gtctctttag gttgcaatgg gtgcgggtag
     2581 agcggtggtc gtcgatattg gtggtgtagt gtgtgtgtgt gtgtgtgtgt gtgtgtgtgt
     2641 gtgtgtgtgt gtgtgtgtgt gtgtgtgtgt gtgtgtgtgt gtgtgtgtgt gtgtgtgtgt
     2701 gtgcaacggc aattattttt tgtaatattt cgaccatctt tctttctctc tctccacgtg
     2761 ctgctgctgt tgctgctgct gctgcattgc atgttccact attcctctcg gtttgtgcct
     2821 gcggacgcca ttgctagtcg aaagagagtc gccgttagtc gcgcttcgag caacggacac
```

2881 gttttttggt tgaaaccaac agcttttttc atcttcggga gacacacaga tctcgaatcg
2941 tacattccca taaggagaat tgtcatcttc cggtgaataa agaaaggaaa cggatccgga
3001 aacatgggat catcatcaga cgacgaagct acggccgatg cgcagcatgc agcaccacca
3061 aaaaaaaaac gaaaagtaga agacccacga tttatgtacc catacgatgt tcctgactat
3121 gcgggtatga aaaacatcaa aaaaaaccag gGAATTCGCC CTTTCATGAG CGGCCCTCCA
3181 AAAAAGAAGA GAAAGGTAGA AGACCCGGGC GGCCGCATGT CCAATTTACT GACCGTACAC
3241 CAAAATTTGC CTGCATTACC GGTCGATGCA ACGAGTGATG AGGTTCGCAA GAACCTGATG
3301 GACATGTTCA GGGATCGCCA GGCGTTTTCT GAGCATACCT GGAAAATGCT TCTGTCCGTT
3361 TGCCGGTCGT GGGCGGCATG GTGCAAGTTG AATAACCGGA AATGGTTTCC CGCAGAACCT
3421 GAAGATGTTC GCGATTATCT TCTATATCTT CAGGCGCGCG GTCTGGCAGT AAAAACTATC
3481 CAGCAACATT TGGGCCAGCT AAACATGCTT CATCGTCGGT CCGGGCTGCC ACGACCAAGT
3541 GACAGCAATG CTGTTTCACT GGTTATGCGG CGGATCCGAA AAGAAAACGT TGATGCCGGT
3601 GAACGTGCAA AACAGGCTCT AGCGTTCGAA CGCACTGATT TCGACCAGGT TCGTTCACTC
3661 ATGGAAAATA GCGATCGCTG CCAGGATATA CGTAATCTGG CATTTCTGGG GATTGCTTAT
3721 AACACCCTGT TACGTATAGC CGAAATTGCC AGGATCAGGG TTAAAGATAT CTCACGTACT
3781 GACGGTGGGA GAATGTTAAT CCATATTGGC AGAACGAAAA CGCTGGTTAG CACCGCAGGT
3841 GTAGAGAAGG CACTTAGCCT GGGGGTAACT AAACTGGTCG AGCGATGGAT TTCCGTCTCT
3901 GGTGTAGCTG ATGATCCGAA TAACTACCTG TTTTGCCGGG TCAGAAAAAA TGGTGTTGCC
3961 GCGCCATCTG CCACCAGCCA GCTATCAACT CGCGCCCTGG AAGGGATTTT TGAAGCAACT
4021 CATCGATTGA TTTACGGCGC TAAGGATGAC TCTGGTCAGA GATACCTGGC CTGGTCTGGA
4081 CACAGTGCCC GTGTCGGAGC CGCGCGAGAT ATGGCCCGCG CTGGAGTTTC AATACCGGAG
4141 ATCATGCAAG CTGGTGGCTG GACCAATGTA AATATTGTCA TGAACTATAT CCGTAACCTG
4201 GATAGTGAAA CAGGGGCAAT GGTGCGCCTG CTGGAAGATG GCGATTAGCC ATgtacCTCg
4261 agcttggggt ggggttgtta tgtgttgcga acgagagtgg atctctctcg acatttcctt
4321 atttttttttt tctattgtta acacttacaa cgaaacttcg gaagagaagt ttcctcattc
4381 gaaacgagga gtcaaactcc tttccttgct tttgtgacat gcatgattat tctttcattt
4441 actgacgtaa cgatgtaaaa cacacagaag aagcatgaca cacagcaaag aattgttcgt
4501 taataaaacg ttgatgaaaa ctttgaaaac ataagaactt gcacttttat tctataattc
4561 gtgaaagctt gcaccgattg tctttcatta ttcaatgtaa tgtactgaaa ggtgattttt
4621 cgcacttgta tactctagaa ctgaagtatt ctaacaatac gtcaccttta ggtccattcc
4681 aggataaaat acacaagtga aggtagttgt acaaagtact tagactaacc caggtttcaa
4741 aaaagataac gacgaattga gcgtaactgc acaaagcccg ttcattgtca acatatcgtt
4801 caggggtctt caaactttat tatcagcccg cgggccgctt agagcaccat cttgatgtat
4861 cctttatcta gtggcttatc gtttatgtct cgtttggcta agaaaatact aataacatat
4921 tcaatttacg gagcggtacc atggtacaaa cgtcatactg ctagacttga caacatgccc
4981 atcgagggtt cttacctcaa atggaccgat cccttgtaac tggcggcgta aactcagtaa
5041 attccaaaag ccagtatagg ccaccatgac cgtctaacta ttccaaaaag gagaatattt
5101 gaaatttcta tatctatagt attattacta tatctatatg tttctttagt acaatattag
5161 agcttgatta ttttcctgaa aattgtaaaa tatgcatgtt agatggttga tacatcgttt
5221 taaaaaaaac tgctgttaat ggCTCGAGCA CCCAACTTTT CTATACAAAG TTGGCATTAT
5281 AAGAAAGCAT TGCTTATCAA TTTGTTGCAA CGAACAGGTC ACTATCAGTC AAAATAAAAT
5341 CATTATTTGC CATCCAGCTG CAGGGCGGCC GCGATATCCC CTATAGTGAG TCGTATTACA
5401 TGGTCATAGC TGTTTCCTGG CAGCTCTGGC CCGTGTCTCA AAATCTCTGA TGTTACATTG
5461 CACAAGATAA AAATATATCA TCATGAACAA TAAAACTGTC TGCTTACATA AACAGTAATA
5521 CAAGGGGTGT TATGAGCCAT ATTCAACGGG AAACGTCGAG GCCGCGATTA AATTCCAACA
5581 TGGATGCTGA TTTATATGGG TATAAATGGG CTCGCGATAA TGTCGGGCAA TCAGGTGCGA
5641 CAATCTATCG CTTGTATGGG AAGCCCGATG CGCCAGAGTT GTTTCTGAAA CATGGCAAAG
5701 GTAGCGTTGC CAATGATGTT ACAGATGAGA TGGTCAGACT AAACTGGCTG ACGGAATTTA
5761 TGCCTCTTCC GACCATCAAG CATTTTATCC GTACTCCTGA TGATGCATGG TTACTCACCA
5821 CTGCGATCCC CGGAAAAACA GCATTCCAGG TATTAGAAGA ATATCCTGAT TCAGGTGAAA
5881 ATATTGTTGA TGCGCTGGCA GTGTTCCTGC GCCGGTTGCA TTCGATTCCT GTTTGTAATT
5941 GTCCTTTTAA CAGCGATCGC GTATTTCGTC TCGCTCAGGC GCAATCACGA ATGAATAACG
6001 GTTTGGTTGA TGCGAGTGAT TTTGATGACG AGCGTAATGG CTGGCCTGTT GAACAAGTCT
6061 GGAAAGAAAT GCATAAACTT TTGCCATTCT CACCGGATTC AGTCGTCACT CATGGTGATT
6121 TCTCACTTGA TAACCTTATT TTTGACGAGG GGAAATTAAT AGGTTGTATT GATGTTGGAC
6181 GAGTCGGAAT CGCAGACCGA TACCAGGATC TTGCCATCCT ATGGAACTGC CTCGGTGAGT
6241 TTTCTCCTTC ATTACAGAAA CGGCTTTTTC AAAAATATGG TATTGATAAT CCTGATATGA
6301 ATAAATTGCA GTTTCATTTG ATGCTCGATG AGTTTTTCTA ATCAGAATTG GTTAATTGGT
6361 TGTAACACTG GCAGAGCATT ACGCTGACTT GACGGGACGG CGCAAGCTCA TGACCAAAAT
6421 CCCTTAACGT GAGTTACGCG TCGTTCCACT GAGCGTCAGA CCCCGTAGAA AAGATCAAAG
6481 GATCTTCTTG AGATCCTTTT TTTCTGCGCG TAATCTGCTG CTTGCAAACA AAAAAACCAC
6541 CGCTACCAGC GGTGGTTTGT TTGCCGGATC AAGAGCTACC AACTCTTTTT CCGAAGGTAA
6601 CTGGCTTCAG CAGAGCGCAG ATACCAAATA CTGTTCTTCT AGTGTAGCCG TAGTTAGGCC
6661 ACCACTTCAA GAACTCTGTA GCACCGCCTA CATACCTCGC TCTGCTAATC CTGTTACCAG
6721 TGGCTGCTGC CAGTGGCGAT AAGTCGTGTC TTACCGGGTT GGACTCAAGA CGATAGTTAC

```
     6781 CGGATAAGGC GCAGCGGTCG GGCTGAACGG GGGGTTCGTG CACACAGCCC AGCTTGGAGC
     6841 GAACGACCTA CACCGAACTG AGATACCTAC AGCGTGAGCT ATGAGAAAGC GCCACGCTTC
     6901 CCGAAGGGAG AAAGGCGGAC AGGTATCCGG TAAGCGGCAG GGTCGGAACA GGAGAGCGCA
     6961 CGAGGGAGCT TCCAGGGGGA AACGCCTGGT ATCTTTATAG TCCTGTCGGG TTTCGCCACC
     7021 TCTGACTTGA GCGTCGATTT TTGTGATGCT CGTCAGGGGG GCGGAGCCTA TGGAAAAACG
     7081 CCAGCAACGC GGCCTTTTTA CGGTTCCTGG CCTTTTGCTG GCCTTTTGCT CACATGTT
//
```

pDSAR-Cre-F2A-IsceI (transgenesis plasmid that produced the C2S transgenic line, expressing Cre and I-SceI)

```
LOCUS       pDSAR-Cre-F2A-Is        9463 bp    DNA     circular
FEATURES             Location/Qualifiers
     misc_feature    complement(7724..7742)
                     /note="M13R"
     misc_feature    6591..7272
                     /note="DsRed"
     misc_feature    6332..6381
                     /note="3xP3 element"
     misc_feature    6421..6428
                     /note="TATA"
     misc_feature    5755..5981
                     /note="SV40 term"
     misc_feature    5982..6258
                     /note="attB"
     misc_feature    7274..7514
                     /note="SV40 term"
     misc_feature    complement(286..1275)
                     /dnas_title="3'UTR_Vasa"
                     /vntifkey="21"
                     /label=3'UTR_Vasa
     misc_feature    complement(1285..2111)
                     /dnas_title="I-SceI CDS"
                     /vntifkey="21"
                     /label=I-SceI CDS
     misc_feature    complement(2197..3276)
                     /note="Cre ORF"
     misc_feature    complement(2044..2057)
                     /note="NLS"
     misc_feature    complement(1996..2022)
                     /note="HA"
     misc_feature    complement(2186..2195)
                     /note="furin site"
     misc_feature    complement(2116..2178)
                     /note="2A peptide"
     misc_feature    complement(3451..5739)
                     /dnas_title="5'Vas_Int"
                     /vntifkey="21"
                     /label=5'Vas_Int
     misc_feature    3368..3381
                     /note="NLS"
     misc_feature    complement(3319..3344)
                     /note="HA epitope"
     source          1..9463
                     /dnas_title="pDSAR-Cre-F2A-Isce-2"

ORIGIN
        1 CTTTCCTGCG TTATCCCCTG ATTCTGTGGA TAACCGTATT ACCGCCTTTG AGTGAGCTGA
       61 TACCGCTCGC CGCAGCCGAA CGACCGAGCG CAGCGAGTCA GTGAGCGAGG AAGCGGAAGA
      121 GCGCCCAATA CGCAAACCGC CTCTCCCCGC GCGTTGGCCG ATTCATTAAT GCAGCTGGCA
      181 CGACAGGTTT CCCGACTGGA AAGCGGGCAG TGAGCGCAAC GCAATTAATA CGCGTACCGc
      241 tagCGGGGGG CCCGGTACCA ACTTTATTAT ACaaagttgt ctcgagaaaa tgtggccatt
      301 aacagcagtt ttttttaaaa cgatgtatca accatctaac atgcatattt tacaattttc
      361 aggaaaataa tcaagctcta atattgtact aaagaaacat atagatatag taataatact
```

```
 421 atagatatag aaatttcaaa tattctcctt tttggaatag ttagacggtc atggtggcct
 481 atactggctt ttggaattta ctgagtttac gccgccagtt acaagggatc ggtccatttg
 541 aggtaagaac cctcgatggg catgttgtca agtctagcag tatgacgttt gtaccatggt
 601 accgctccgt aaattgaata tgttattagt attttcttag ccaaacgaga cataaacgat
 661 aagccactag ataaaggata catcaagatg gtgctctaag cggcccgcgg gctgataata
 721 aagtttgaag acccctgaac gatatgttga caatgaacgg gctttgtgca gttacgctca
 781 attcgtcgtt atcttttttg aaacctgggt tagtctaagt actttgtaca actaccttca
 841 cttgtgtatt ttatcctgga atggacctaa aggtgacgta ttgttagaat acttcagttc
 901 tagagtatac aagtgcgaaa aatcaccttt cagtacatta cattgaataa tgaaagacaa
 961 tcggtgcaag ctttcacgaa ttatagaata aaagtgcaag ttcttatgtt ttcaaagttt
1021 tcatcaacgt tttattaacg aacaattctt tgctgtgtgt catgcttctt ctgtgtgttt
1081 tacatcgtta cgtcagtaaa tgaaagaata atcatgcatg tcacaaaagc aaggaaagga
1141 gtttgactcc tcgtttcgaa tgaggaaact tctcttccga agtttcgttg taagtgttaa
1201 caatagaaaa aaaaaataag gaaatgtcga gagagatcca ctctcgttcg caacacataa
1261 caaccccacc ccaagctcga gctcttattt caggaaagtt tcggaggaga tagtgttcgg
1321 cagtttgtac atcatctgcg ggatcaggta cggtttgatc aggttgtaga agatcaggta
1381 agacatagaa tcgatgtaga tgatcggttt gtttttgttg atttttacgt aacagttcag
1441 ttggaatttg ttacgcagac ccttaaccag gtattctact tcttcgaaag tgaaagactg
1501 ggtgttcagt acgatcgatt tgttggtaga gtttttgttg taatcccatt taccaccatc
1561 atccatgaac cagtatgcca gagacatcgg ggtcaggtag ttttcaacca ggttgttcgg
1621 gatggttttt ttgttgttaa cgatgaacag gttagccagt ttgttgaaag cttggtgttt
1681 gaaagtctgg gcgccccagg tgattaccag gttacccagg tggttaacac gttctttttt
1741 gtgcggcggg gacagtaccc actgatcgta cagcagacat acgtggtcca tgtatgcttt
1801 gtttttccac tcgaactgca tacagtaggt tttaccttca tcacgagaac ggatgtaagc
1861 atcacccagg atcagaccga tacctgcttc gaactgttcg atgttcagtt cgatcagctg
1921 ggatttgtat tctttcagca gtttagagtt cggacccagg ttcattacct ggtttttttt
1981 gatgttttc atacccgcat agtcaggaac atcgtatggg tacataaatc gtgggtcttc
2041 tacttttcgt ttttttttg gtggtgctgc atgctgcgca tcggccgtag cttcgtcgtc
2101 tgatgatgat cCTGGTCCGG GATTCGACTC CACGTCTCCA GCCAGCTTCA GCAGGTCGAA
2161 GTTCAGGGTC TGCTTCACCG GAGCACGCTT GGCGCGATCG CCATCTTCCA GCAGGCGCAC
2221 CATTGCCCCT GTTTCACTAT CCAGGTTACG GATATAGTTC ATGACAATAT TTACATTGGT
2281 CCAGCCACCA GCTTGCATGA TCTCCGGTAT TGAAACTCCA GCGCGGGCCA TATCTCGCGC
2341 GGCTCCGACA CGGGCACTGT GTCCAGACCA GGCCAGGTAT CTCTGACCAG AGTCATCCTT
2401 AGCGCCGTAA ATCAATCGAT GAGTTGCTTC AAAAATCCCT TCCAGGGCGC GAGTTGATAG
2461 CTGGCTGGTG GCAGATGGCG CGGCAACACC ATTTTTTCTG ACCCGGCAAA ACAGGTAGTT
2521 ATTCGGATCA TCAGCTACAC CAGAGACGGA AATCCATCGC TCGACCAGTT TAGTTACCCC
2581 CAGGCTAAGT GCCTTCTCTA CACCTGCGGT GCTAACCAGC GTTTTCGTTC TGCCAATATG
2641 GATTAACATT CTCCCACCGT CAGTACGTGA GATATCTTTA ACCCTGATCC TGGCAATTTC
2701 GGCTATACGT AACAGGGTGT TATAAGCAAT CCCCAGAAAT GCCAGATTAC GTATATCCTG
2761 GCAGCGATCG CTATTTTCCA TGAGTGAACG AACCTGGTCG AAATCAGTGC GTTCGAACGC
2821 TAGAGCCTGT TTTGCACGTT CACCGGCATC AACGTTTTCT TTTCGGATCC GCCGCATAAC
2881 CAGTGAAACA GCATTGCTGT CACTTGGTCG TGGCAGCCCG GACCGACGAT GAAGCATGTT
2941 TAGCTGGCCC AAATGTTGCT GGATAGTTTT TACTGCCAGA CCGCGCGCCT GAAGATATAG
3001 AAGATAATCG CGAACATCTT CAGGTTCTGC GGGAAACCAT TTCCGGTTAT TCAACTTGCA
3061 CCATGCCGCC CACGACCGGC AAACGGACAG AAGCATTTTC CAGGTATGCT CAGAAAACGC
3121 CTGGCGATCC CTGAACATGT CCATCAGGTT CTTGCGAACC TCATCACTCG TTGCATCGAC
3181 CGGTAATGCA GGCAAATTTT GGTGTACGGT CAGTAAATTG GACATGCGGC CGCCCGGGTC
3241 TTCTACCTTT CTCTTCTTTT TTGGAGGGCC GCTCATGAAA GGGCGAATTC cctggttttt
3301 tttgatgttt ttcatacccg catagtcagg aacatcgtat gggtacataa atcgtgggtc
3361 ttctactttt cgtttttttt ttggtggtgc tgcatgctgc gcatcggccg tagcttcgtc
3421 gtctgatgat gatcccatgt ttccggatcc gtttcctttc tttattcacc ggaagatgac
3481 aattctcctt atgggaatgt acgattcgag atctgtgtgt ctcccgaaga tgaaaaaagc
3541 tgttggtttc aaccaaaaaa cgtgtccgtt gctcgaagcg cgactaacgg cgactctctt
3601 tcgactagca atggcgtccg caggcacaaa ccgagaggaa tagtggaaca tgcaatgcag
3661 cagcagcagc aacagcagca gcacgtggag agagagaaag aaagatggtc gaaatattac
3721 aaaaaataat tgccgttgca cacacacaca cacacacaca cacacacaca cacacacaca
3781 cacacacaca cacacacaca cacacacaca cacacacaca cacacacaca cactacacca
3841 ccaatatcga cgaccaccgc tctacccgca cccattgcaa cctaaagaga cgatggtatt
3901 ttccagcgaa attttcattg tccattgcaa gcgacctcac acaccgccac cgaagtagcc
3961 tttgaagcac gcgcgcgtgt gaaggcgtgt gcctctgtgt gtgtaggtga atatcactgt
4021 gtacactgcg ctaagccagt ggcgcgcgcg cttttcgcat caattattgc cggatgaacg
4081 acaacgagat gctcttcgcg gggcccgctg tcccggaacg tgcacgaaag caccatttct
4141 cctctcggaa actccacatc tcggaaaggc ttcccaacta tgccccaacg actctcttat
4201 ctattgcata tcgaagactt ttgaatccgc tccagtgcca actgcggttc atgaaacacg
4261 aaactgtttt tctcccctga ttcgagcaaa caagagcacc atggtaagct gtggaaataa
```

4321 cgccatcctt ctggatatct tgtaacgaaa aaggccaagg actgtgattc tactaacctc
4381 actctggaac tgatatgaga gcacacagag ccacacaacc actccacgac gggatacgct
4441 tggattaaaa ctaaaccgct tccgtacgtc gacaccgact ggcccctata ccgcttctca
4501 aataacgacg atctcagcta aaatgccccc cacacacatg catgcgcagc acacttgttc
4561 acttccactt ccttgcctgc tctgtgtgcg gtggtggata cgtgctgtgc tgtaagaaca
4621 cttcacacag aaaggaaggg acagagcaaa ccaacacact gccaccgctc ttctcgtgcc
4681 atttccttag aaaattaatc caaaaactca actgcgccac acaccacggc agccacggca
4741 gcctgctatc aaaagggggg aggcgcgcaa catgcacaac acacgataca caccacggaa
4801 tgtttacgaa cagcgtgcga acaggcaatt tcggtaccgg cggcaacagc cgattttgac
4861 agagcgctca acacacacac gcacacactc taataccagc aacgcgtgcg aaggcttttg
4921 tttcgcactg ccagtgtgtt agtgggagca ggtacaaaat gaaacaaagg gcgaatggcg
4981 cgtgctactg aacaatacaa gcgaattcga gaaaaattaa ataagccaag agattatttt
5041 atgagtaaat gaaatgaaat ttaatttaac tgtattttga acataactaa gaaaatacta
5101 atcaactgga tgaaataata taacatcaca aaacaataat tcaatttgaa atcagttacg
5161 ttagttttgc cgaaatgaga gattttttc acaaacactt cactaacttc taacaaacaa
5221 acatttgttc tataaaacta gttcacttac ctgagttttg aagtactatt cggctggtta
5281 tttcacttgc aaaagcaatt ggctaattta gcgctaagca cctcagcaga aacctccgac
5341 tcgccgcgag tgctccgagc aaaagactgc taaaagctgg aatttgcacg gacacggcct
5401 actcgtccgt cgtactgtgc aactaggatg gaagatttcg atacgggact ctacagggag
5461 ggtttatatg tgttttataa tgctgcatgt atgtttttat gtataaaata tgcaaaaagt
5521 atgtcttttt agtttattat aacaaataaa atacaataca aattataaaa caatcaattt
5581 ctcaacggtt ttgtttgcag cgagttgtga tttgcttgaa aacaaagcgc acgctgtaat
5641 tttcccgccc agttcggcat gtcaaacacg tctgacgttc ggcttcccac tgtagctgct
5701 gctgtcatgg aaggaaaaga atttgctcgc gttctacatc gATACCGTCG ACGCTAGACA
5761 TAATCAGCCA TACCACATTT GTAGAGGTTT TACTTGCTTT AAAAAACCTC CCACACCTCC
5821 CCCTGAACCT GAAACATAAA ATGAATGCAA TTGTTGTTGT TAACTTGTTT ATTGCAGCTT
5881 ATAATGGTTA CAAATAAAGC AATAGCATCA CAAATTTCAC AAATAAAGCA TTTTTCTTCA
5941 CTGCATTCTA GTTGTGGTTT GTCCAAACTC ATCAATGTAT CtcgaCGATG TAGGTCACaG
6001 TCTCGAAGCC GCGGTGCGGG TGCCAGGGCG TGCCCTTGGG CTCCCCGGGC GCGTACTCCA
6061 CCTCACCCAT CTGGTCCATC ATGATGAACG GGTCGAGGTG GCGGTAGTTG ATCCCGGCGA
6121 ACGCGCGGCG CACCGGGAAG CCCTCGCCCT CGAAACCGCT GGGCGCGGTG GTCACGGTGA
6181 GCACGGGACG TGCGACGGCG TCGGCGGGTG CGGATACGCG GGGCAGCGTC AGCGGGTTCT
6241 CGACGGTCAC GGCGGGCAat tCCTGCAGAC TTCCGGTATC TCGCGTTTGT TTGATCGCAC
6301 GGTTCCCACA ATGGTTAATT CGAGCTCGCC CGGGGATCTA ATTCAATTAG AGACTAATTC
6361 AATTAGAGCT AATTCAATTA GGATCCAAGC TTATCGATTT CGAACCCTCG ACCGCCGGAG
6421 TATAAATAGA GGCGCTTCGT CTACGGAGCG ACAATTCAAT TCAAACAAGC AAAGTGAACA
6481 CGTCGCTAAG CGAAAGCTAA GCAAATAAAC AAGCGCAGCT GAACAAGCTA AACAATCGGG
6541 GTACCGCTAG AGTCGACGGT ACCGCGGGCC CGGGATCCAC CGGTCGCCAC CATGGTGCGC
6601 TCCTCCAAGA ACGTCATCAA GGAGTTCATG CGCTTCAAGG TGCGCATGGA GGGCACCGTG
6661 AACGGCCACG AGTTCGAGAT CGAGGGCGAG GGCGAGGGCC GCCCCTACGA GGGCCACAAC
6721 ACCGTGAAGC TGAAGGTGAC CAAGGGCGGC CCCCTGCCCT TCGCCTGGGA CATCCTGTCC
6781 CCCCAGTTCC AGTACGGCTC CAAGGTGTAC GTGAAGCACC CCGCCGACAT CCCCGACTAC
6841 AAGAAGCTGT CCTTCCCCGA GGGCTTCAAG TGGGAGCGCG TGATGAACTT CGAGGACGGC
6901 GGCGTGGTGA CCGTGACCCA GGACTCCTCC CTGCAGGACG GCTGCTTCAT CTACAAGGTG
6961 AAGTTCATCG GCGTGAACTT CCCCTCCGAC GGCCCCGTAA TGCAGAAGAA GACCATGGGC
7021 TGGGAGGCCT CCACCGAGCG CCTGTACCCC CGCGACGGCG TGCTGAAGGG CGAGATCCAC
7081 AAGGCCCTGA AGCTGAAGGA CGGCGGCCAC TACCTGGTGG AGTTCAAGTC CATCTACATG
7141 GCCAAGAAGC CCGTGCAGCT GCCCGGCTAC TACTACGTGG ACTCCAAGCT GGACATCACC
7201 TCCCACAACG AGGACTACAC CATCGTGGAg CAGTACGAGC GCACCGAGGG CCGCCACCAC
7261 CTGTTCCTGT AGCGGCCGCG ACTCTAGATC ATAATCAGCC ATACCACATT TGTAGAGGTT
7321 TTACTTGCTT TAAAAAACCT CCCACACCTC CCCCTGAACC TGAAACATAA AATGAATGCA
7381 ATTGTTGTTG TTAACTTGTT TATTGCAGCT TATAATGGTT ACAAATAAAG CAATAGCATC
7441 ACAAATTTCA CAAATAAAGC ATTTTTTTCA CTGCATTCTA GTTGTGGTTT GTCCAAACTC
7501 ATCAATGTAT CTTAAAGCTT ATCGATACGC GTACGGCGCG CCTAGAGCGG CCGCCACCGC
7561 GGTGGAGCTC GAGTACCCAG CTTTCTTGTA CAAAGTTGGC ATTATAAGAA AGCATTGCTT
7621 ATCAATTTGT TGCAACGAAC AGGTCACTAT CAGTCAAAAT AAAATCATTA TTTGCCATCC
7681 AGCTGCAGGG CGGCCGCGAT ATCCCCTATA GTGAGTCGTA TTACATGGTC ATAGCTGTTT
7741 CCTGGCAGCT CTGGCCCGTG TCTCAAAATC TCTGATGTTA CATTGCACAA GATAAAAATA
7801 TATCATCATG AACAATAAAA CTGTCTGCTT ACATAAACAG TAATACAAGG GGTGTTATGA
7861 GCCATATTCA ACGGGAAACG TCGAGGCCGC GATTAAATTC CAACATGGAT GCTGATTTAT
7921 ATGGGTATAA ATGGGCTCGC GATAATGTCG GGCAATCAGG TGCGACAATC TATCGCTTGT
7981 ATGGGAAGCC CGATGCGCCA GAGTTGTTTC TGAAACATGG CAAAGGTAGC GTTGCCAATG
8041 ATGTTACAGA TGAGATGGTC AGACTAAACT GGCTGACGGA ATTTATGCCT CTTCCGACCA
8101 TCAAGCATTT TATCCGTACT CCTGATGATG CATGGTTACT CACCACTGCG ATCCCCGGAA
8161 AAACAGCATT CCAGGTATTA GAAGAATATC CTGATTCAGG TGAAAATATT GTTGATGCGC

```
8221 TGGCAGTGTT CCTGCGCCGG TTGCATTCGA TTCCTGTTTG TAATTGTCCT TTTAACAGCG
8281 ATCGCGTATT TCGTCTCGCT CAGGCGCAAT CACGAATGAA TAACGGTTTG GTTGATGCGA
8341 GTGATTTTGA TGACGAGCGT AATGGCTGGC CTGTTGAACA AGTCTGGAAA GAAATGCATA
8401 AACTTTTGCC ATTCTCACCG GATTCAGTCG TCACTCATGG TGATTTCTCA CTTGATAACC
8461 TTATTTTTGA CGAGGGGAAA TTAATAGGTT GTATTGATGT TGGACGAGTC GGAATCGCAG
8521 ACCGATACCA GGATCTTGCC ATCCTATGGA ACTGCCTCGG TGAGTTTTCT CCTTCATTAC
8581 AGAAACGGCT TTTTCAAAAA TATGGTATTG ATAATCCTGA TATGAATAAA TTGCAGTTTC
8641 ATTTGATGCT CGATGAGTTT TTCTAATCAG AATTGGTTAA TTGGTTGTAA CACTGGCAGA
8701 GCATTACGCT GACTTGACGG GACGGCGCAA GCTCATGACC AAAATCCCTT AACGTGAGTT
8761 ACGCGTCGTT CCACTGAGCG TCAGACCCCG TAGAAAAGAT CAAAGGATCT TCTTGAGATC
8821 CTTTTTTTCT GCGCGTAATC TGCTGCTTGC AAACAAAAAA ACCACCGCTA CCAGCGGTGG
8881 TTTGTTTGCC GGATCAAGAG CTACCAACTC TTTTTCCGAA GGTAACTGGC TTCAGCAGAG
8941 CGCAGATACC AAATACTGTT CTTCTAGTGT AGCCGTAGTT AGGCCACCAC TTCAAGAACT
9001 CTGTAGCACC GCCTACATAC CTCGCTCTGC TAATCCTGTT ACCAGTGGCT GCTGCCAGTG
9061 GCGATAAGTC GTGTCTTACC GGGTTGGACT CAAGACGATA GTTACCGGAT AAGGCGCAGC
9121 GGTCGGGCTG AACGGGGGGT TCGTGCACAC AGCCCAGCTT GGAGCGAACG ACCTACACCG
9181 AACTGAGATA CCTACAGCGT GAGCTATGAG AAAGCGCCAC GCTTCCCGAA GGGAGAAAGG
9241 CGGACAGGTA TCCGGTAAGC GGCAGGGTCG GAACAGGAGA GCGCACGAGG GAGCTTCCAG
9301 GGGGAAACGC CTGGTATCTT TATAGTCCTG TCGGGTTTCG CCACCTCTGA CTTGAGCGTC
9361 GATTTTTGTG ATGCTCGTCA GGGGGGCGGA GCCTATGGAA AAACGCCAGC AACGCGGCCT
9421 TTTTACGGTT CCTGGCCTTT TGCTGGCCTT TTGCTCACAT GTT
//
```
