# Supplementary material for: Tools for Anopheles gambiae Transgenesis
Source: G3 (Bethesda). 2015 Apr 13;5(6):1151–63. doi: 10.1534/g3.115.016808 (PMC4478545; doi:10.1534/g3.115.016808)
Supplement: Supporting Information [file supp_g3.115.016808_FileS3.pdf]

File S3

DNA sequence of transgenesis plasmids used to generate reporter lines.

pAttBRfB2-ppo6Tom: Plasmid used to generate *PPO6*-tdTomato line

```
LOCUS       attBRfB2PPO6_Tom        7852 bp    DNA     circular
FEATURES             Location/Qualifiers
     misc_feature    complement(2216..2500)
                     /note="attB"
     misc_feature    2138..2156
                     /note="M13R"
     misc_feature    7167..7186
                     /note="attB2"
     misc_feature    complement(2541..3492)
                     /note="PPO9 mRNA"
     misc_feature    4014..4052
                     /note="PPO6 5'UTR"
     misc_feature    3968..3975
                     /note="putative TATA box"
     misc_feature    4057..4078
                     /note="attB4"
     misc_feature    5671..5870
                     /note="sv40 terminator"
     gene            6179..6895
                     /note="EYFP (Clontech)"
     misc_feature    6008..6015
                     /note="TATA"
     misc_feature    6902..7156
                     /note="SV40 term sequences"
     misc_feature    4108..5536
                     /note="tdTomato"
     misc_feature    5602..5623
                     /note="attB3"
     misc_feature    2516..2537
                     /note="attB1"
     source          1..7852
                     /dnas_title="attBRfB2PPO6_Tomato_Sv40YFP"
     misc_feature    5919..5968
                     /note="3xP3 binding site"
ORIGIN
        1 GTGGCACTTT TCGGGGAAAT GTGCGCGGAA CCCCTATTTG TTTATTTTTC TAAATACATT
       61 CAAATATGTA TCCGCTCATG AGACAATAAC CCTGATAAAT GCTTCAATAA TATTGAAAAA
      121 GGAAGAGTAT GAGTATTCAA CATTTCCGTG TCGCCCTTAT TCCCTTTTTT GCGGCATTTT
      181 GCCTTCCTGT TTTTGCTCAC CCAGAAACGC TGGTGAAAGT AAAAGATGCT GAAGATCAGT
      241 TGGGTGCACG AGTGGGTTAC ATCGAACTGG ATCTCAACAG CGGTAAGATC CTTGAGAGTT
      301 TTCGCCCCGA AGAACGTTTT CCAATGATGA GCACTTTTAA AGTTCTGCTA TGTGGCGCGG
      361 TATTATCCCG TATTGACGCC GGGCAAGAGC AACTCGGTCG CCGCATACAC TATTCTCAGA
      421 ATGACTTGGT TGAGTACTCA CCAGTCACAG AAAAGCATCT TACGGATGGC ATGACAGTAA
      481 GAGAATTATG CAGTGCTGCC ATAACCATGA GTGATAACAC TGCGGCCAAC TTACTTCTGA
      541 CAACGATCGG AGGACCGAAG GAGCTAACCG CTTTTTTGCA CAACATGGGG GNATCATGTA
      601 ACTCGCCTTN GATCGTTGGG AACCGGAGCT GAATGAAGCC ATACCAAACG ACGAGCGTGA
      661 CACCACGATG CCTGTAGCAA TGGCAACAAC GTTGCGCAAA CTATTAACTG GCGAACTACT
      721 TACTCTAGCT TCCCGGCAAC AATTAATAGA CTGGATGGAG GCGGATAAAG TTGCAGGACC
      781 ACTTCTGCGC TCGGCCCTTC CGGCTGGCTG GTTTATTGCT GATAAATCTG GAGCCGGTGA
      841 GCGTGGGTCT CGCGGTATCA TTGCAGCACT GGGGCCAGAT GGTAAGCCCT CCCGTATCGT
      901 AGTTATCTAC ACGACGGGGA GTCAGGCAAC TATGGATGAA CGAAATAGAC AGATCGCTGA
      961 GATAGGTGCC TCACTGATTA AGCATTGGTA ACTGTCAGAC CAAGTTTACT CATATATACT
     1021 TTAGATTGAT TTAAAACTTC ATTTTTAATT TAAAAGGATC TAGGTGAAGA TCCTTTTTGA
     1081 TAATCTCATG ACCAAAATCC CTTAACGTGA GTTTTCGTTC CACTGAGCGT CAGACCCCGT
     1141 AGAAAAGATC AAAGGATCTT CTTGAGATCC TTTTTTTCTG CGCGTAATCT GCTGCTTGCA
     1201 AACAAAAAAA CCACCGCTAC CAGCGGTGGT TTGTTTGCCG GATCAAGAGC TACCAACTCT
     1261 TTTTCCGAAG GTAACTGGCT TCAGCAGAGC GCAGATACCA AATACTGTCC TTCTAGTGTA
     1321 GCCGTAGTTA GGCCACCACT TCAAGAACTC TGTAGCACCG CCTACATACC TCGCTCTGCT
     1381 AATCCTGTTA CCAGTGGCTG CTGCCAGTGG CGATAAGTCG TGTCTTACCG GGTTGGACTC
     1441 AAGACGATAG TTACCGGATA AGGCGCAGCG GTCGGGCTGA ACGGGGGGTT CGTGCACACA
     1501 GCCCAGCTTG GAGCGAACGA CCTACACCGA ACTGAGATAC CTACAGCGTG AGCTATGAGA
     1561 AAGCGCCACG CTTCCCGAAG GGAGAAAGGC GGACAGGTAT CCGGTAAGCG GCAGGGTCGG
     1621 AACAGGAGAG CGCACGAGGG AGCTTCCAGG GGGAAACGCC TGGTATCTTT ATAGTCCTGT
     1681 CGGGTTTCGC CACCTCTGAC TTGAGCGTCG ATTTTTGTGA TGCTCGTCAG GGGGGCGGAG
     1741 CCTATGGAAA AACGCCAGCA ACGCGGCCTT TTTACGGTTC CTGGCCTTTT GCTGGCCTTT
     1801 TGCTCACATG TTCTTTCCTG CGTTATCCCC TGATTCTGTG GATAACCGTA TTACCGCCTT
     1861 TGAGTGAGCT GATACCGCTC GCCGCAGCCG AACGACCGAG CGCAGCGAGT CAGTGAGCGA
     1921 GGAAGCGGAA GAGCGCCCAA TACGCAAACC GCCTCTCCCC GCGCGTTGGC CGATTCATTA
     1981 ATGCAGCTGG CACGACAGGT TTCCCGACTG GAAAGCGGGC AGTGAGCGCA ACGCAATTAA
     2041 TGTGAGTTAG CTCACTCATT AGGCACCCCA GGCTTTACAC TTTATGCTTC CGGCTCGTAT
```

```
2101 GTTGTGTGGA ATTGTGAGCG GATAACAATT TCACACAGGA AACAGCTATG ACCATGATTA
2161 CGCCAAGCTC GAAATTAACC CTCACTAAAG GGAACAAAAG CTGGCTAGAA CTAGTGTCGA
2221 CATGCCCGCC GTGACCGTCG AGAACCCGCT GACGCTGCCC CGCGTATCCG CACCCGCCGA
2281 CGCCGTCGCA CGTCCCGTGC TCACCGTGAC CACCGCGCCC AGCGGTTTCG AGGGCGAGGG
2341 CTTCCCGGTG CGCCGCGCGT TCGCCGGGAT CAACTACCGC CACCTCGACC CGTTCATCAT
2401 GATGGACCAG ATGGGTGAGG TGGAGTACGC GCCCGGGGAG CCCAAGGGCA CGCCCTGGCA
2461 CCCGCACCGC GGCTTCGAGA CCGTGACCTA CATCGTCGAC ACTAGTGgat catcaacaag
2521 tttGTACAAA AAAGCAGGCT GGACGCAACG GTGCAGATTA CGAGCGGTAA GGCGGCCAGA
2581 AATAGATTGC TAACCTTCTG GCAACGGACG CAAGTTGATC TGGGAACGGG GCTAGATTTC
2641 GGACCGCAAG GTAACGTGTT GGCAACgTTC ACCCACATCC AGCACGCACC GTTTGCGTAC
2701 CAAATTATGG TACAAAACGA AACGGCGGAG CAAAAGAAGG GAACTGTTCG CATTTTCCTC
2761 GCCCCGATCT ACGATGCGAA CGGAGAGCAA CTGTTACTGA GCCAGCAGCG TCGGTACATG
2821 CTGGAGATGG ACAAATTTGT CGTCAAGTGT AAGTATACAT TAAGTTGAGC AATACTGTTA
2881 TGGTGCTGCA ATCGTATCGT GTGTTTCCTT CAAGTACATC CTGGCGATAA cCGGATCATT
2941 CGACGATCGG ACCAGTCAAG CGTAACCATA CCGTACGAAA GGACCTTCCG GCGAGTTGAt
3001 GCTTCCAACA TGCCGGGCAC GGAGAGCTTC CGCTTCTGCA ACTGTGGCTG GCCCGATCAT
3061 ATGCTGCTGC CCAAGGGACA TCCCGATGGT CAACCGTTCG ATCTGTTTAT CATGATTTCT
3121 GATTACAAGG ACGATGCTGT AAGCACCGGA TTtAATGAGT GAGTACATTA CGGAATCAGT
3181 ATTGATGAAC TCGGTTTTAA CTTTTGAGTT TTTTTTAAAT ATCTTATGgT CATGTAGGAA
3241 TGAAAACTGT AACGATTCAC ATTCATACTG TGGTCTACGC GATCAGCTGT ATCCGGACCG
3301 TCGTGCGATG GGTTTTCCCT TTGACCGACA GCCGGTTGCC CAGGATCACT TGATGAAGGA
3361 CTTTGTGGGC AGGTTCCCCA ATATGAGTCG TACCGTAGCG GAAGTTATGT TCACCAACAC
3421 TATCATTTCA CGCACGTAAA TGGCATCACG ATAACCGATC GATGAACGAT ACTTGTGGCT
3481 GACCCGTATG ATCGTTTTTT TTATCAACGA GGAACATTAT ATTAAAATGC AATGGAAAAA
3541 TGAGCAATAA AATATATTCA TAcTCAAGCA AATATATACC GCATTgCATT aCtTTCTTAA
3601 AGTTATTTCT ATTTTTTGCA AGTATTATGA TTTTTTTCAA CAATGCGTtT TATTTTCCTC
3661 AACCACCGAT ATGCAATATG GATAAGGCAC ACACATTCCC AGTTATCGTA ATCGCTTACC
3721 AAATGATTGG AATACCGCAA ACATCCACAA AAATAGAACG GATTGATATG TTTTATCAAA
3781 GCTTCATGCA TGTGCATACG CaTGGTTTAC ATTTGCTTCC CATGTACTGC AACAATTGCA
3841 GATTACAATT GCATTGTACC ATAgCATTAT CGCAGATGCA GTGGTCGTAA ACCGCAAAAA
3901 TGGCGATTGG AATTGGAAGA AATTGCATAC AACCAGGCAC CGGCACGTCA AGTGCAAATC
3961 AGAATGATAT ATAAACTATA CCAACTACTT CTGTAGCATC ACAAACGTGA CTGTAACAGT
4021 GACTGGTGGT TCTCGTTTCG TTCTCGCGCG TCcaccCAAC TTTTCTATAC AAAGTTGGTA
4081 CCGGGCCCCC CGCTAGCGTC GACAACCATG GTGAGCAAGG GCGAGGAGGT CATCAAAGAG
4141 TTCATGCGCT TCAAGGTGCG CATGGAGGGC TCCATGAACG GCCACGAGTT CGAGATCGAG
4201 GGCGAGGGCG AGGGCCGCCC CTACGAGGGC ACCCAGACCG CCAAGCTGAA GGTGACCAAG
4261 GGCGGCCCCC TGCCCTTCGC CTGGGACATC CTGTCCCCCC AGTTCATGTA CGGCTCCAAG
4321 GCGTACGTGA AGCACCCCGC CGACATCCCC GATTACAAGA AGCTGTCCTT CCCCGAGGGC
4381 TTCAAGTGGG AGCGCGTGAT GAACTTCGAG GACGGCGGTC TGGTGACCGT GACCCAGGAC
4441 TCCTCCCTGC AGGACGGCAC GCTGATCTAC AAGGTGAAGA TGCGCGGCAC CAACTTCCCC
4501 CCCGACGGCC CCGTAATGCA GAAGAAGACC ATGGGCTGGG AGGCCTCCAC CGAGCGCCTG
4561 TACCCCCGCG ACGGCGTGCT GAAGGGCGAG ATCCACCAGG CCCTGAAGCT GAAGGACGGC
4621 GGCCACTACC TGGTGGAGTT CAAGACCATC TACATGGCCA AGAAGCCCGT GCAACTGCCC
4681 GGCTACTACT ACGTGGACAC CAAGCTGGAC ATCACCTCCC ACAACGAGGA CTACACCATC
4741 GTGGAACAGT ACGAGCGCTC CGAGGGCCGC CACCACCTGT TCCTGGGGCA TGGCACCGGC
4801 AGCACCGGCA GCGGCAGCTC CGGCACCGCC TCCTCCGAGG ACAACAACAT GGCCGTCATC
4861 AAAGAGTTCA TGCGCTTCAA GGTGCGCATG GAGGGCTCCA TGAACGGCCA CGAGTTCGAG
4921 ATCGAGGGCG AGGGCGAGGG CCGCCCCTAC GAGGGCACCC AGACCGCCAA GCTGAAGGTG
4981 ACCAAGGGCG GCCCCCTGCC CTTCGCCTGG GACATCCTGT CCCCCCAGTT CATGTACGGC
5041 TCCAAGGCGT ACGTGAAGCA CCCCGCCGAC ATCCCCGATT ACAAGAAGCT GTCCTTCCCC
5101 GAGGGCTTCA AGTGGGAGCG CGTGATGAAC TTCGAGGACG GCGGTCTGGT GACCGTGACC
5161 CAGGACTCCT CCCTGCAGGA CGGCACGCTG ATCTACAAGG TGAAGATGCG CGGCACCAAC
5221 TTCCCCCCCG ACGGCCCCGT AATGCAGAAG AAGACCATGG GCTGGGAGGC CTCCACCGAG
5281 CGCCTGTACC CCCGCGACGG CGTGCTGAAG GGCGAGATCC ACCAGGCCCT GAAGCTGAAG
5341 GACGGCGGCC ACTACCTGGT GGAGTTCAAG ACCATCTACA TGGCCAAGAA GCCCGTGCAA
5401 CTGCCCGGCT ACTACTACGT GGACACCAAG CTGGACATCA CCTCCCACAA CGAGGACTAC
5461 ACCATCGTGG AACAGTACGA GCGCTCCGAG GGCCGCCACC ACCTGTTCCT GTACGGCATG
5521 GACGAGCTGT ACAAGTAAGA ATTCCTGCAG CCCGGGGGAT CCACTAGTTC TAGAGCGGCC
5581 GCCACCGCGG TGGAGCTCGA GACAACTTTG TATAATAAAG TTGGTACCGG GCCCCCCGCT
5641 AGCGTCGACG GTATCGATAA GCTTGATATC GAATTCTCTA GATCATAATC AGCCATACCA
5701 CATTTGTAGA GGTTTTACTT GCTTTAAAAA ACCTCCCACA CCTCCCCCTG AACCTGAAAC
5761 ATAAAATGAA TGCAATTGTT GTTGTTAACT TGTTTATTGC AGCTTATAAT GGTTACAAAT
5821 AAAGCAATAG CATCACAAAT TTCACAAATA AAGCATTTTT CTTCACTGCA TTCTAGTTGT
5881 GGTTTGTCCA AACTCATCAA TGTATCAAGG GCGAATTCGG GGATCTAATT CAATTAGAGA
5941 CTAATTCAAT TAGAGCTAAT TCAATTAGGA TCCAAGCTTA TCGATTTCGA ACCCTCGACC
6001 GCCGGAGTAT AAATAGAGGC GCTTCGTCTA CGGAGCGACA ATTCAATTCA AACAAGCAAA
6061 GTGAACACGT CGCTAAGCGA AAGCTAAGCA AATAAACAAG CGCAGCTGAA CAAGCTAAAC
6121 AATCGGGGTA CCGCTAGAGT CGACGGTACC GCGGGCCCGG GATCCACCGG TCGCCACCAT
6181 GGTGAGCAAG GGCGAGGAGC TGTTCACCGG GGTGGTGCCC ATCCTGGTCG AGCTGGACGG
6241 CGACGTAAAC GGCCACAAGT TCAGCGTGTC CGGCGAGGGC GAGGGCGATG CCACCTACGG
6301 CAAGCTGACC CTGAAGTTCA TCTGCACCAC CGGCAAGCTG CCCGTGCCCT GGCCCACCCT
6361 CGTGACCACC TTCGGCTACG GCCTGCAGTG CTTCGCCCGC TACCCCGACC ACATGAAGCA
6421 GCACGACTTC TTCAAGTCCG CCATGCCCGA AGGCTACGTC CAGGAGCGCA CCATCTTCTT
6481 CAAGGACGAC GGCAACTACA AGACCCGCGC CGAGGTGAAG TTCGAGGGCG ACACCCTGGT
6541 GAACCGCATC GAGCTGAAGG GCATCGACTT CAAGGAGGAC GGCAACATCC TGGGGCACAA
6601 GCTGGAGTAC AACTACAACA GCCACAACGT CTATATCATG GCCGACAAGC AGAAGAACGG
6661 CATCAAGGTG AACTTCAAGA TCCGCCACAA CATCGAGGAC GGCAGCGTGC AGCTCGCCGA
6721 CCACTACCAG CAGAACACCC CCATCGGCGA CGGCCCCGTG CTGCTGCCCG ACAACCACTA
6781 CCTGAGCTAC CAGTCCGCCC TGAGCAAAGA CCCCAACGAG AAGCGCGATC ACATGGTCCT
6841 GCTGGAGTTC GTGACCGCCG CCGGGATCAC TCTCGGCATG GACGAGCTGT ACAAGTAAAG
6901 CGGCCGCGAC TCTAGATCAT AATCAGCCAT ACCACATTTG TAGAGGTTTT ACTTGCTTTA
```

```
     6961 AAAAACCTCC CACACCTCCC CCTGAACCTG AAACATAAAA TGAATGCAAT TGTTGTTGTT
     7021 AACTTGTTTA TTGCAGCTTA TAATGGTTAC AAATAAAGCA ATAGCATCAC AAATTTCACA
     7081 AATAAAGCAT TTTTTTCACT GCATTCTAGT TGTGGTTTGT CCAAACTCAT CAATGTATCT
     7141 TAAAGCTTAT CGATACctcg agTACCCAGC TTTcttgtac aaagtggttg atCGGTACGT
     7201 ACCCAATTCG CCCTATAGTG AGTCGTATTA CAATTCACTG GCCGTCGTTT TACAACGTCG
     7261 TGACTGGGAA AACCCTGGCG TTACCCAACT TAATCGCCTT GCAGCACATC CCCCTTTCGC
     7321 CAGCTGGCGT AATAGCGAAG AGGCCCGCAC CGATCGCCCT TCCCAACAGT TGCGCAGCCT
     7381 GAATGGCGAA TGGAAATTGT AAGCGTTAAT ATTTTGTTAA AATTCGCGTT AAATTTTTGT
     7441 TAAATCAGCT CATTTTTTAA CCAATAGGCC GAAATCGGCA AAATCCCTTA TAAATCAAAA
     7501 GAATAGACCG AGATAGGGTT GAGTGTTGTT CCAGTTTGGA ACAAGAGTCC ACTATTAAAG
     7561 AACGTGGACT CCAACGTCAA AGGGCGAAAA ACCGTCTATC AGGGCGATGG CCCACTACGT
     7621 GAACCATCAC CCTAATCAAG TTTTTTGGGG TCGAGGTGCC GTAAAGCACT AAATCGGAAC
     7681 CCTAAAGGGA GCCCCCGATT TAGAGCTTGA CGGGGAAAGC CGGCGAACGT GGCGAGAAAG
     7741 GAAGGGAAGA AAGCGAAAGG AGCGGGCGCT AGGGCGCTGG CAAGTGTAGC GGTCACGCTG
     7801 CGCGTAACCA CCACACCCGC CGCGCTTAAT GCGCCGCTAC AGGGCGCGTC AG
//
```

pAttBRfB2-VgGFP: Plasmid used to generate *Vg*-GFP line

```
LOCUS       attBrfB2Vg_GFP_Sv       7475 bp    DNA     circular
FEATURES             Location/Qualifiers
     misc_feature    complement(6834..6854)
                     /note="T7"
     misc_feature    complement(2216..2500)
                     /note="attB"
     misc_feature    2173..2192
                     /note="T3"
     misc_feature    2138..2156
                     /note="M13R"
     gene            5223..5422
                     /note="sv40 terminator"
     misc_feature    5543..5592
                     /note="3xP3 binding sites"
     misc_feature    5154..5175
                     /note="attB3"
     misc_feature    5802..6483
                     /note="DsRed"
     misc_feature    5632..5639
                     /note="TATA"
     misc_feature    6790..6809
                     /note="attB2"
     misc_feature    2516..2537
                     /note="attB1"
     misc_feature    complement(4378..5126)
                     /note="GFP"
     misc_feature    2563..4264
                     /note="Vitellogenin promoter"
     misc_feature    4279..4300
                     /note="attB4"
     source          1..7475
                     /dnas_title="attBrf2Vg_GFP_Sv40dsRED"
     misc_feature    6496..6687
                     /note="SV40 terminator"
ORIGIN
        1 GTGGCACTTT TCGGGGAAAT GTGCGCGGAA CCCCTATTTG TTTATTTTTC TAAATACATT
       61 CAAATATGTA TCCGCTCATG AGACAATAAC CCTGATAAAT GCTTCAATAA TATTGAAAAA
      121 GGAAGAGTAT GAGTATTCAA CATTTCCGTG TCGCCCTTAT TCCCTTTTTT GCGGCATTTT
      181 GCCTTCCTGT TTTTGCTCAC CCAGAAACGC TGGTGAAAGT AAAAGATGCT GAAGATCAGT
      241 TGGGTGCACG AGTGGGTTAC ATCGAACTGG ATCTCAACAG CGGTAAGATC CTTGAGAGTT
      301 TTCGCCCCGA AGAACGTTTT CCAATGATGA GCACTTTTAA AGTTCTGCTA TGTGGCGCGG
      361 TATTATCCCG TATTGACGCC GGGCAAGAGC AACTCGGTCG CCGCATACAC TATTCTCAGA
      421 ATGACTTGGT TGAGTACTCA CCAGTCACAG AAAAGCATCT TACGGATGGC ATGACAGTAA
      481 GAGAATTATG CAGTGCTGCC ATAACCATGA GTGATAACAC TGCGGCCAAC TTACTTCTGA
      541 CAACGATCGG AGGACCGAAG GAGCTAACCG CTTTTTTGCA CAACATGGGG GNATCATGTA
      601 ACTCGCCTTN GATCGTTGGG AACCGGAGCT GAATGAAGCC ATACCAAACG ACGAGCGTGA
      661 CACCACGATG CCTGTAGCAA TGGCAACAAC GTTGCGCAAA CTATTAACTG GCGAACTACT
      721 TACTCTAGCT TCCCGGCAAC AATTAATAGA CTGGATGGAG GCGGATAAAG TTGCAGGACC
      781 ACTTCTGCGC TCGGCCCTTC CGGCTGGCTG GTTTATTGCT GATAAATCTG GAGCCGGTGA
      841 GCGTGGGTCT CGCGGTATCA TTGCAGCACT GGGGCCAGAT GGTAAGCCCT CCCGTATCGT
      901 AGTTATCTAC ACGACGGGGA GTCAGGCAAC TATGGATGAA CGAAATAGAC AGATCGCTGA
      961 GATAGGTGCC TCACTGATTA AGCATTGGTA ACTGTCAGAC CAAGTTTACT CATATATACT
     1021 TTAGATTGAT TTAAAACTTC ATTTTTAATT TAAAAGGATC TAGGTGAAGA TCCTTTTTGA
     1081 TAATCTCATG ACCAAAATCC CTTAACGTGA GTTTTCGTTC CACTGAGCGT CAGACCCCGT
     1141 AGAAAAGATC AAAGGATCTT CTTGAGATCC TTTTTTTCTG CGCGTAATCT GCTGCTTGCA
     1201 AACAAAAAAA CCACCGCTAC CAGCGGTGGT TTGTTTGCCG GATCAAGAGC TACCAACTCT
     1261 TTTTCCGAAG GTAACTGGCT TCAGCAGAGC GCAGATACCA AATACTGTCC TTCTAGTGTA
     1321 GCCGTAGTTA GGCCACCACT TCAAGAACTC TGTAGCACCG CCTACATACC TCGCTCTGCT
```

```
1381 AATCCTGTTA CCAGTGGCTG CTGCCAGTGG CGATAAGTCG TGTCTTACCG GGTTGGACTC
1441 AAGACGATAG TTACCGGATA AGGCGCAGCG GTCGGGCTGA ACGGGGGGTT CGTGCACACA
1501 GCCCAGCTTG GAGCGAACGA CCTACACCGA ACTGAGATAC CTACAGCGTG AGCTATGAGA
1561 AAGCGCCACG CTTCCCGAAG GGAGAAAGGC GGACAGGTAT CCGGTAAGCG GCAGGGTCGG
1621 AACAGGAGAG CGCACGAGGG AGCTTCCAGG GGGAAACGCC TGGTATCTTT ATAGTCCTGT
1681 CGGGTTTCGC CACCTCTGAC TTGAGCGTCG ATTTTTGTGA TGCTCGTCAG GGGGGCGGAG
1741 CCTATGGAAA AACGCCAGCA ACGCGGCCTT TTTACGGTTC CTGGCCTTTT GCTGGCCTTT
1801 TGCTCACATG TTCTTTCCTG CGTTATCCCC TGATTCTGTG GATAACCGTA TTACCGCCTT
1861 TGAGTGAGCT GATACCGCTC GCCGCAGCCG AACGACCGAG CGCAGCGAGT CAGTGAGCGA
1921 GGAAGCGGAA GAGCGCCCAA TACGCAAACC GCCTCTCCCC GCGCGTTGGC CGATTCATTA
1981 ATGCAGCTGG CACGACAGGT TTCCCGACTG GAAAGCGGGC AGTGAGCGCA ACGCAATTAA
2041 TGTGAGTTAG CTCACTCATT AGGCACCCCA GGCTTTACAC TTTATGCTTC CGGCTCGTAT
2101 GTTGTGTGGA ATTGTGAGCG GATAACAATT TCACACAGGA AACAGCTATG ACCATGATTA
2161 CGCCAAGCTC GAAATTAACC CTCACTAAAG GGAACAAAAG CTGGCTAGAA CTAGTGTCGA
2221 CATGCCCGCC GTGACCGTCG AGAACCCGCT GACGCTGCCC CGCGTATCCG CACCCGCCGA
2281 CGCCGTCGCA CGTCCCGTGC TCACCGTGAC CACCGCGCCC AGCGGTTTCG AGGGCGAGGG
2341 CTTCCCGGTG CGCCGCGCGT TCGCCGGGAT CAACTACCGC CACCTCGACC CGTTCATCAT
2401 GATGGACCAG ATGGGTGAGG TGGAGTACGC GCCCGGGGAG CCCAAGGGCA CGCCCTGGCA
2461 CCCGCACCGC GGCTTCGAGA CCGTGACCTA CATCGTCGAC ACTAGTGgat catcaacaag
2521 tttGTACAAA AAAGCAGGCT GGTACCGGGC CCCCCGCTAG CGTCGAGTTC AACTCGACCA
2581 TAATAATTGA TCCGTCAATC CATATTGGTC CGCAATAATG AAAGTTGCAA GAGTACGACG
2641 GTATGAAAAG AGGTTCAGTA AGTTGTAAAC TAATAGTTTC TTCCCAACGT TCAAATGCTG
2701 GCAAATCTTT TCGCGGGCCG CACTTCGTCG ATCGCTAGTC TTAATGATAA TTTCTGAGAA
2761 AAAGGTGCTA CTGCATCTAC TATATTCTAC TGGATATAAA TGAAATAACA ACGTGAGACT
2821 CACCTACAAC ATGTAATTTA TTGATGGTTT AGTTTAACCA ACCTATGAAA TAATTTGATA
2881 TAGAAATTTG TAGTCGTTTT TCTATGAAGT AAAATTCTAA AATCAAACAT TAAACTGTTT
2941 TGTAGTACCC GGACTCATGG TATGGCTTCT ATTAGCCGTA AACAAAGATT TACAATTGAC
3001 TAAGGTTAGG TCCGACACTG TAGGAGCCAG CGCGTCCTTT CAATACATCA ACGGACCATC
3061 TCGTGTTGTT AAATACTTAT TATTATTATG GTTTGCTAAT TGATATGTTC CAAGACCGAT
3121 TTGGATTTCG AAATAAGTAT TCTCTGATTC ATTTTGGGAG CCGGTCTCGT GATACAGTCG
3181 TCAGCCCGTA CGACTTAACT ACATTCTCGT CATGGGTTCA AGCCCCAGAT GGACCGTGCC
3241 GCCATACGTA GAGTCAGTCC TATCCTGTTA TGGGGGGTAA TACATAAGAC ACTGAAAGCC
3301 AACCCCACAA GTGGTACAGA CAAGCCTTGa CCGACAATTG TTGTTGAGCC AAACAGAAGA
3361 AGAATCCATT TCGGGAAATG ATTTTATCAT TCAATCAAAC CAGTCAATCA TAAACATCAT
3421 AGTTTTAAAT ACTCAAAACT AGTTGAGATC TTTAAAACAC ATTATTTTAG TTTAATTAAA
3481 TGATCTGTTA GCTAGAAGGT AGATACGATA TTTTAGACAT TTCGTAATAG ATCGCAAATC
3541 TCTATTATGT TGGTAATTCA CTTCGTAAAA CTCTTAGGCA AAACTCTTAT TAGTAAACAA
3601 AATACTAATC AAACACTGAT AAACTAACGC GATTTATACA TTGGACAAAG AAGAGGCTGA
3661 TTTTAAAAAT ACTCGCTTTA AAATTTGCTT CATTCATCAA TGTATTGTAA AGCACATAAA
3721 GAACACAATC ATTGACTGAA AACAATTCCA CGTCTCAGCC AACTTCCAGG ATCAATGAAA
3781 TAGCAAGTTC CAAGTTCCAT TTCATTGATT ATGGTAACTA CTGATTATTT TCAATAACAA
3841 ATACTTCGAA GACTGCACAA TTCAAAAGTA TGCCAGAAAG AAAGGATTAC TATCAATTGT
3901 GGGTTAATCA AACTAAGACA GGTGGCAAAA ATGGAACCAT TGATTAAGGC AGCCACTGAC
3961 CGATTTCATT TAAAAAACAC ACTCTTGGAA GTTTCCACAC AATCTCACCT TTTGCCAATT
4021 TTAGCAAAGA CGTTGTGCTG CACTGATAAG AATCGAACTG TAAACATGTG GGCAGTAAAA
4081 ATTATTTCAT CGTTCAACAC GGCGGTCATT ACACTATTCG AAGCAGCTGA AAAGATTTGA
4141 TGATAGCAGG ACCGTGAGAT CAGCAAATTT GAGGTATAAA AGATGATCCT GCGACCACCA
4201 GAAGGCACAT TCGAGCTTTG GAGTGCATTC AAAGCATCCG GGCAACTGCG AACAACCGAA
4261 CCatCGATCT CGAGCACCCA ACTTTTCTAT ACAAAGTTGG TACCGGGCCC CCCGCTAGCG
4321 TCGACGGTAT CGATAAGCTT GATATCGAAT TCGTTAACAG ATCCACCGGT CGCCACCATG
4381 GTGAGCAAGG GCGAGGAGCT GTTCACCGGG GTGGTGCCCA TCCTGGTCGA GCTGGACGGC
4441 GACGTAAACG GCCACAAGTT CAGCGTGTCC GGCGAGGGCG AGGGCGATGC CACCTACGGC
4501 AAGCTGACCC TGAAGTTCAT CTGCACCACC GGCAAGCTGC CCGTGCCCTG GCCCACCCTC
4561 GTGACCACCC TGACCTACGG CGTGCAGTGC TTCAGCCGCT ACCCCGACCA CATGAAGCAG
4621 CACGACTTCT TCAAGTCCGC CATGCCCGAA GGCTACGTCC AGGAGCGCAC CATCTTCTTC
4681 AAGGACGACG GCAACTACAA GACCCGCGCC GAGGTGAAGT TCGAGGGCGA CACCCTGGTG
4741 AACCGCATCG AGCTGAAGGG CATCGACTTC AAGGAGGACG GCAACATCCT GGGGCACAAG
4801 CTGGAGTACA ACTACAACAG CCACAACGTC TATATCATGG CCGacAAGCA GAAGAACGGC
4861 ATCAAGGTGA ACTTCAAGAT CCGCCACAAC ATCGAGGaCG GCAGCGTGCA GCTCGCCGAC
4921 CACTACCAGC AGAACACCCC CATCGGCGac GGCCCCGTGC TGCTGCCCGa CAACCACTAC
4981 CTGAGCACCC AGTCCGCCCT GAGCAAagaC CCCAACGAga agCGCGATCA CATGGTCCTG
5041 CTGgaGTTCG TGACCGCCGC CGGgatCacT CTCGGCATGG acGAgcTGTa caAGTaAAgC
5101 gGCCgcGGcT cGAGGGtACC TctAGaGCGG CCGCCACCGC GGTGGAGCTC GAGACAACTT
5161 TGTATAATAA AGTTGGTACC GGGCCCCCCG CTAGCGTCGA CGGTATCGAT AAGCTTGATA
5221 TCGAATTCTC TAGATCATAA TCAGCCATAC CACATTTGTA GAGGTTTTAC TTGCTTTAAA
5281 AAACCTCCCA CACCTCCCCC TGAACCTGAA ACATAAAATG AATGCAATTG TTGTTGTTAA
5341 CTTGTTTATT GCAGCTTATA ATGGTTACAA ATAAAGCAAT AGCATCACAA ATTTCACAAA
5401 TAAAGCATTT TTCTTCACTG CATTCTAGTT GTGGTTTGTC CAAACTCATC AATGTATCAA
5461 GGGCGAATTC CTGCAGCCCA CTTCCGGTAT CTCGCGTTTG TTTGATCGCA CGGTTCCCAC
5521 AATGGTTAAT TCGAGCTCGC CCGGGGATCT AATTCAATTA GAGACTAATT CAATTAGAGC
5581 TAATTCAATT AGGATCCAAG CTTATCGATT TCGAACCCTC GACCGCCGGA GTATAAATAG
5641 AGGCGCTTCG TCTACGGAGC GACAATTCAA TTCAAACAAG CAAAGTGAAC ACGTCGCTAA
5701 GCGAAAGCTA AGCAAATAAA CAAGCGCAGC TGAACAAGCT AAACAATCGG GGTACCGCTA
5761 GAGTCGACGG TACCGCGGGC CCGGGATCCA CCGGTCGCCA CCATGGTGCG CTCCTCCAAG
5821 AACGTCATCA AGGAGTTCAT GCGCTTCAAG GTGCGCATGG AGGGCACCGT GAACGGCCAC
5881 GAGTTCGAGA TCGAGGGCGA GGGCGAGGGC CGCCCCTACG AGGGCCACAA CACCGTGAAG
5941 CTGAAGGTGA CCAAGGGCGG CCCCCTGCCC TTCGCCTGGG ACATCCTGTC CCCCCAGTTC
6001 CAGTACGGCT CCAAGGTGTA CGTGAAGCAC CCCGCCGACA TCCCCGACTA CAAGAAGCTG
6061 TCCTTCCCCG AGGGCTTCAA GTGGGAGCGC GTGATGAACT TCGAGGACGG CGGCGTGGTG
6121 ACCGTGACCC AGGACTCCTC CCTGCAGGAC GGCTGCTTCA TCTACAAGGT GAAGTTCATC
6181 GGCGTGAACT TCCCCTCCGA CGGCCCCGTA ATGCAGAAGA AGACCATGGG CTGGGAGGCC
```

```
     6241 TCCACCGAGC GCCTGTACCC CCGCGACGGC GTGCTGAAGG GCGAGATCCA CAAGGCCCTG
     6301 AAGCTGAAGG ACGGCGGCCA CTACCTGGTG GAGTTCAAGT CCATCTACAT GGCCAAGAAG
     6361 CCCGTGCAGC TGCCCGGCTA CTACTACGTG GACTCCAAGC TGGACATCAC CTCCCACAAC
     6421 GAGGACTACA CCATCGTGGA CCAGTACGAG CGCACCGAGG GCCGCCACCA CCTGTTCCTG
     6481 TAGCGGCCGC GACTCTAGAT CATAATCAGC CATACCACAT TTGTAGAGGT TTTACTTGCT
     6541 TTAAAAAACC TCCCACACCT CCCCCTGAAC CTGAAACATA AAATGAATGC AATTGTTGTT
     6601 GTTAACTTGT TTATTGCAGC TTATAATGGT TACAAATAAA GCAATAGCAT CACAAATTTC
     6661 ACAAATAAAG CATTTTTCTT CACTGCATTC TAGTTGTGGT TTGTCCAAAC TCATCAATGT
     6721 ATCTTAAAGC TTATCGATAC GCGTACGGCG CGCCTAGAGC GGCCGCCACC GCGGTGGAGC
     6781 TCGAGTACCC AGCTTTcttg tacaaagtgg ttgatCGGTA CGTACCCAAT TCGCCCTATA
     6841 GTGAGTCGTA TTACAATTCA CTGGCCGTCG TTTTACAACG TCGTGACTGG GAAAACCCTG
     6901 GCGTTACCCA ACTTAATCGC CTTGCAGCAC ATCCCCCTTT CGCCAGCTGG CGTAATAGCG
     6961 AAGAGGCCCG CACCGATCGC CCTTCCCAAC AGTTGCGCAG CCTGAATGGC GAATGGAAAT
     7021 TGTAAGCGTT AATATTTTGT TAAAATTCGC GTTAAATTTT TGTTAAATCA GCTCATTTTT
     7081 TAACCAATAG GCCGAAATCG GCAAAATCCC TTATAAATCA AAAGAATAGA CCGAGATAGG
     7141 GTTGAGTGTT GTTCCAGTTT GGAACAAGAG TCCACTATTA AAGAACGTGG ACTCCAACGT
     7201 CAAAGGGCGA AAAACCGTCT ATCAGGGCGA TGGCCCACTA CGTGAACCAT CACCCTAATC
     7261 AAGTTTTTTG GGGTCGAGGT GCCGTAAAGC ACTAAATCGG AACCCTAAAG GGAGCCCCCG
     7321 ATTTAGAGCT TGACGGGGAA AGCCGGCGAA CGTGGCGAGA AAGGAAGGGA AGAAAGCGAA
     7381 AGGAGCGGGC GCTAGGGCGC TGGCAAGTGT AGCGGTCACG CTGCGCGTAA CCACCACACC
     7441 CGCCGCGCTT AATGCGCCGC TACAGGGCGC GTCAG
//
```

pDSAT-LpRFP: Plasmid used to generate *Lp*-tdTomato line

```
LOCUS       pDSAT                   7466 bp    DNA     circular
FEATURES             Location/Qualifiers
     misc_feature    complement(5727..5745)
                     /note="M13R"
     misc_feature    4291..4340
                     /note="3x Pax6 binding sites"
     misc_feature    4380..4387
                     /note="TATA"
     misc_feature    271..286
                     /note="M13F"
     misc_feature    3714..3905
                     /note="SV40 term"
     misc_feature    3941..4217
                     /note="attB"
                     /note="SV40 term"
     misc_feature    4551..5267
                     /note="mTurquoise2"
     misc_feature    468..2098
                     /note="LpPromoter"
     misc_feature    2117..3707
                     /note="tdTomato"
     misc_feature    3509..3682
                     /note="ADDED seq"
     source          1..7466
                     /dnas_title="pDSAT Lp_Tomat"
ORIGIN
        1 CTTTCCTGCG TTATCCCCTG ATTCTGTGGA TAACCGTATT ACCGCCTTTG AGTGAGCTGA
       61 TACCGCTCGC CGCAGCCGAA CGACCGAGCG CAGCGAGTCA GTGAGCGAGG AAGCGGAAGA
      121 GCGCCCAATA CGCAAACCGC CTCTCCCCGC GCGTTGGCCG ATTCATTAAT GCAGCTGGCA
      181 CGACAGGTTT CCCGACTGGA AAGCGGGCAG TGAGCGCAAC GCAATTAATA CGCGTACCGC
      241 TAGCATGGAT GTTTTCCCAG TCACGACGTT GTAAAACGAC GGCCAGTCTT AAGCTCGGGC
      301 CCCTACAGGT CACTAATACC ATCTAAGTAG TTGATTCATA GTGACTGGAT ATGTTGTGTT
      361 TTACAGTATT ATGTAGTCTG TTTTTTATGC AAAATCTAAT TTAATATATT GATATTTATA
      421 TCATTTTACG TTTCTCGTTC AACTTTTCTA TACAAAGTTg gtACcggATC CAgcTTGCGG
      481 GGAAGACACA TTCGAGATAC GCTAAGTGAT TGAGCGATTA CGATCTAGCA AAACATACGT
      541 TCAGCTGTGA GAATAATCAT CCATCTTCCT GCAATGAGCA GTTCATTCCC GATTGAGGGA
      601 TTTTATTCCC CGGGGGCCTT TTCAAACGGC TTAATATAAG CAATTAATAG TATTTTTTCT
      661 TTCAGGTTAG TTTACTGTAA TGGTGTAATT GTCATCTTAC ACCTCCGTCT GATAAGAGAT
      721 TACGAAGCTC AGTATGATGA AATAAATAAG ATAAATTTAT TTAAAAAAGA ACAATTGCTA
      781 TGAGAGTGAA ATACAACAGT GGCGTTCACA ATATTCGAAA AACAATAAAA TTAAAAAAAA
      841 AACAAGAAAA CATTCACAAA CATATCAATC TGCTTTCATC GACACCGAAC TGCTAGCCTC
      901 CCCAGTCTAA CCGCGGTGGG GACGTTTAAT TGCCTTTGTT CTCGCACCCG GTCAAACATA
      961 CACTTCGGAC CTTGCTCCGA ACCCCACTGT GATCCCTAGC TCGTCATCAT CATTGCCGGC
     1021 ATCATGCTAA GCGTGCATTA TTTTCACAAC TTAGCGTAAT GCTAGCGTGC GCTAGCAACA
     1081 AACTCGCCCG CAGACTCGTC ACAGCACCGG TACGATCGAT CGTTTACCGT TCCCTTTCCC
     1141 GATCGGGTTG GCTGCGATAT CCGTGTCCGG GTAGAAAACT TCCCCTTTTA CACACACACA
     1201 CTCACATACA CACAGAGCTG AATAGCAACT TACCTTATCT GTTCGTCATC GCTCGGGCGG
     1261 ATCTGGACGA ATCTTCGCAC CGATAACCAT GTGGATCTAC GACCTCCGCT TGGCTGTCTC
     1321 TCTGCTCATG TGTATGTCTG TGTGTGTGTG TATGTGAGCT TCTTCCCTCA AATCCCTCGA
     1381 TCTCGCTGTG SCAACAATCA AACGTGCAAG TGCAAACATT GCACCCCATT GATTATACAC
     1441 CCAACACCAA CCAATTCCCC TTGCGGAGGC ATCTCTGTGC TCGGCAGCAT GTTTACCGCA
     1501 GATCTACAGA GAACTTCAAT TGAGGTCCTT TCCACCCCCA GCCCTCAACC GGCAATCCGG
     1561 CAGCCACTGG ATCATCAGCG AAAGAGAGAG AGAGCAGAGC AGAACAGAGG TGACCAACTG
     1621 TGGTATCGCT TCCCGCGCGC CGGTGTGTTG GTGTCCATTT CGGTGATCGC GATCCCGGCC
```

1681 GCTTCCAGCA CCGTCCACCG ATCAGTCACA AAAACGCTCT CCAAACCCCT TATCAGCACC
1741 GTTCGCTGAT GTGAACCCCC GTTCAAACCC CAAATGCAGT GTTTGTATTG CTGTGTGTAT
1801 GTGTACGTGC GTGTGTGTGG AAATTCTATA AAAGTAGGCA CCCGTGGCCG GGATCCGTTA
1861 TTCCCGTCCT GAGGCCCGCC CGGGATCGCT GGTGACGACA GACGAGCCGC TGTGTGACGT
1921 ACGTAGTGCC CGATCGGTAA AGAGTGAACC GTCTTCTCTG CAGTGTAGGA GAGAACGGTT
1981 TCATCTTTTT CGCCCACACC CCCCCGGTTA CATTCCATGT TGAACTGTAA GGTCTAGTGA
2041 ACATTTCCTG AGTGTGGAAA GTGTGGTTTA GTGCGTGAGA GTGCACGGAC ACGACACTAC
2101 CACCATGGTG AGCAAGGGCG AGGAGGTCAT CAAAGAGTTC ATGCGCTTCA AGGTGCGCAT
2161 GGAGGGCTCC ATGAACGGCC ACGAGTTCGA GATCGAGGGC GAGGGCGAGG GCCGCCCCTA
2221 CGAGGGCACC CAGACCGCCA AGCTGAAGGT GACCAAGGGC GGCCCCCTGC CCTTCGCCTG
2281 GGACATCCTG TCCCCCCAGT TCATGTACGG CTCCAAGGCG TACGTGAAGC ACCCCGCCGA
2341 CATCCCCGAT TACAAGAAGC TGTCCTTCCC CGAGGGCTTC AAGTGGGAGC GCGTGATGAA
2401 CTTCGAGGAC GGCGGTCTGG TGACCGTGAC CCAGGACTCC TCCCTGCAGG ACGGCACGCT
2461 GATCTACAAG GTGAAGATGC GCGGCACCAA CTTCCCCCCC GACGGCCCCG TAATGCAGAA
2521 GAAGACCATG GGCTGGGAGG CCTCCACCGA GCGCCTGTAC CCCCGCGACG GCGTGCTGAA
2581 GGGCGAGATC CACCAGGCCC TGAAGCTGAA GGACGGCGGC CACTACCTGG TGGAGTTCAA
2641 GACCATCTAC ATGGCCAAGA AGCCCGTGCA ACTGCCCGGC TACTACTACG TGGACACCAA
2701 GCTGGACATC ACCTCCCACA ACGAGGACTA CACCATCGTG GAACAGTACG AGCGCTCCGA
2761 GGGCCGCCAC CACCTGTTCC TGGGGCATGG CACCGGCAGC ACCGGCAGCG GCAGCTCCGG
2821 CACCGCCTCC TCCGAGGACA ACAACATGGC CGTCATCAAA GAGTTCATGC GCTTCAAGGT
2881 GCGCATGGAG GGCTCCATGA ACGGCCACGA GTTCGAGATC GAGGGCGAGG GCGAGGGCCG
2941 CCCCTACGAG GGCACCCAGA CCGCCAAGCT GAAGGTGACC AAGGGCGGCC CCCTGCCCTT
3001 CGCCTGGGAC ATCCTGTCCC CCCAGTTCAT GTACGGCTCC AAGGCGTACG TGAAGCACCC
3061 CGCCGACATC CCCGATTACA AGAAGCTGTC CTTCCCCGAG GGCTTCAAGT GGGAGCGCGT
3121 GATGAACTTC GAGGACGGCG GTCTGGTGAC CGTGACCCAG GACTCCTCCC TGCAGGACGG
3181 CACGCTGATC TACAAGGTGA AGATGCGCGG CACCAACTTC CCCCCCGACG GCCCCGTAAT
3241 GCAGAAGAAG ACCATGGGCT GGGAGGCCTC CACCGAGCGC CTGTACCCCC GCGACGGCGT
3301 GCTGAAGGGC GAGATCCACC AGGCCCTGAA GCTGAAGGAC GGCGGCCACT ACCTGGTGGA
3361 GTTCAAGACC ATCTACATGG CCAAGAAGCC CGTGCAACTG CCCGGCTACT ACTACGTGGA
3421 CACCAAGCTG GACATCACCT CCCACAACGA GGACTACACC ATCGTGGAAC AGTACGAGCG
3481 CTCCGAGGGC CGCCACCACC TGTTCCTGGG GCATGGCACC GGCAGCACCG GCAGCGGCAG
3541 CTCCGGCACC GCCTCCTCCG AGGACAACAA CATGGCCGTC ATCAAAGAGT TCATGCGCTT
3601 CAAGGTGCGC ATGGAGGGCT CCATGAACGG CCACGAGTTC GAGATCGAGG GCGAGGGCGA
3661 GGGCCGCCAC CACCTGTTCC TGTACGGCAT GGACGAGCTG TACAAGTAAc ttCTAGACAT
3721 AATCAGCCAT ACCACATTTG TAGAGGTTTT ACTTGCTTTA AAAAACCTCC CACACCTCCC
3781 CCTGAACCTG AAACATAAAA TGAATGCAAT TGTTGTTGTT AACTTGTTTA TTGCAGCTTA
3841 TAATGGTTAC AAATAAAGCA ATAGCATCAC AAATTTCACA AATAAAGCAT TTTTCTTCAC
3901 TGCATTCTAG TTGTGGTTTG TCCAAACTCA TCAATGTATC tcgaCGATGT AGGTCACaGT
3961 CTCGAAGCCG CGGTGCGGGT GCCAGGGCGT GCCCTTGGGC TCCCCGGGCG CGTACTCCAC
4021 CTCACCCATC TGGTCCATCA TGATGAACGG GTCGAGGTGG CGGTAGTTGA TCCCGGCGAA
4081 CGCGCGGCGC ACCGGGAAGC CCTCGCCCTC GAAACCGCTG GGCGCGGTGG TCACGGTGAG
4141 CACGGGACGT GCGACGGCGT CGGCGGGTGC GGATACGCGG GGCAGCGTCA GCGGGTTCTC
4201 GACGGTCACG GCGGGCAatt CCTGCAGACT TCCGGTATCT CGCGTTTGTT TGATCGCACG
4261 GTTCCCACAA TGGTTAATTC GAGCTCGCCC GGGGATCTAA TTCAATTAGA GACTAATTCA
4321 ATTAGAGCTA ATTCAATTAG GATCCAAGCT TATCGATTTC GAACCCTCGA CCGCCGGAGT
4381 ATAAATAGAG GCGCTTCGTC TACGGAGCGA CAATTCAATT CAAACAAGCA AAGTGAACAC
4441 GTCGCTAAGC GAAAGCTAAG CAAATAAACA AGCGCAGCTG AACAAGCTAA ACAATCGGGG
4501 TACCGCTAGA GTCGACGGTA CCGCGGGCCC GGGATCCACC GGTCGCCACc atggtgagca
4561 agggcgagga gctgttcacc ggggtggtgc ccatcctggt cgagctggac ggcgacgtaa
4621 acggccacaa gttcagcgtg tccggcgagg gcgagggcga tgccacctac GGCAAGCTGA
4681 CCCTGAAGTT Catctgcacc accggcaagc tgcccgtgcc ctggcccacc ctcgtgacca
4741 ccctgtcctg ggcgtgcag tgcttcgccc gctaccccga ccacatgaag cagcacgact
4801 tcttcaagtc cgccatgccc gaaggctacg tccaggagcg caccatcttc ttcaaggacg
4861 acggcaacta caagacccgc gccgaggtga agttcgaggg cgacaccctg gtgaaccgca
4921 tcgagctgaa gggcatcgac ttcaaggagg acggcaacat cctggggcac aagctggagt
4981 acaactacTt Tagcgacaac gtctatatca ccgccgacaa gcagaagaac ggcatcaagg
5041 ccaacttcaa gatccgccac aacatcgagg acggcggcgt gcagctcgcc gaccactacc
5101 agcagaacac ccccatcggc gacggccccg tgctgctgcc cgacaaccac tacctgagca
5161 cccagtccaa gctgagcaaa gaccccaacg agaagcgcga tcacatggtc ctgctggagt
5221 tcgtgaccgc cgccgggatc actctcggca tggacgagct gtacaagtcc ggaTGAtaga
5281 tctgacgggt gatcaAATCA GCCATACCAC ATTTGTAGAG GTTTTACTTG CTTTAAAAAA
5341 CCTCCCACAC CTCCCCCTGA ACCTGAAACA TAAAATGAAT GCAATTGTTG TTGTTAACTT
5401 GTTTATTGCA GCTTATAATG GTTACAAATA AAGCAATAGC ATCACAAATT TCACAAATAA
5461 AGCATTTTTT TCACTGCATT CTAGTTGTGG TTTGTCCAAA CTCATCAATG TATCTTAAAG
5521 CTTATCGATA CGCGTACGGC GCGCCTAGAG CGGCCGCCAC CGCGGTGGAG CTCGAGTACC
5581 CAGCTTTCTT GTACAAAGTT GGCATTATAA GAAAGCATTG CTTATCAATT TGTTGCAACG
5641 AACAGGTCAC TATCAGTCAA AATAAAATCA TTATTTGCCA TCCAGCTGCA GGGCGGCCGC
5701 GATATCCCCT ATAGTGAGTC GTATTACATG GTCATAGCTG TTTCCTGGCA GCTCTGGCCC
5761 GTGTCTCAAA ATCTCTGATG TTACATTGCA CAAGATAAAA ATATATCATC ATGAACAATA
5821 AAACTGTCTG CTTACATAAA CAGTAATACA AGGGGTGTTA TGAGCCATAT TCAACGGGAA
5881 ACGTCGAGGC CGCGATTAAA TTCCAACATG GATGCTGATT TATATGGGTA TAAATGGGCT
5941 CGCGATAATG TCGGGCAATC AGGTGCGACA ATCTATCGCT TGTATGGGAA GCCCGATGCG
6001 CCAGAGTTGT TTCTGAAACA TGGCAAAGGT AGCGTTGCCA ATGATGTTAC AGATGAGATG
6061 GTCAGACTAA ACTGGCTGAC GGAATTTATG CCTCTTCCGA CCATCAAGCA TTTTATCCGT
6121 ACTCCTGATG ATGCATGGTT ACTCACCACT GCGATCCCCG GAAAAACAGC ATTCCAGGTA
6181 TTAGAAGAAT ATCCTGATTC AGGTGAAAAT ATTGTTGATG CGCTGGCAGT GTTCCTGCGC
6241 CGGTTGCATT CGATTCCTGT TTGTAATTGT CCTTTTAACA GCGATCGCGT ATTTCGTCTC
6301 GCTCAGGCGC AATCACGAAT GAATAACGGT TTGGTTGATG CGAGTGATTT TGATGACGAG
6361 CGTAATGGCT GGCCTGTTGA ACAAGTCTGG AAAGAAATGC ATAAACTTTT GCCATTCTCA
6421 CCGGATTCAG TCGTCACTCA TGGTGATTTC TCACTTGATA ACCTTATTTT TGACGAGGGG
6481 AAATTAATAG GTTGTATTGA TGTTGGACGA GTCGGAATCG CAGACCGATA CCAGGATCTT

```
     6541 GCCATCCTAT GGAACTGCCT CGGTGAGTTT TCTCCTTCAT TACAGAAACG GCTTTTTCAA
     6601 AAATATGGTA TTGATAATCC TGATATGAAT AAATTGCAGT TTCATTTGAT GCTCGATGAG
     6661 TTTTTCTAAT CAGAATTGGT TAATTGGTTG TAACACTGGC AGAGCATTAC GCTGACTTGA
     6721 CGGGACGGCG CAAGCTCATG ACCAAAATCC CTTAACGTGA GTTACGCGTC GTTCCACTGA
     6781 GCGTCAGACC CCGTAGAAAA GATCAAAGGA TCTTCTTGAG ATCCTTTTTT TCTGCGCGTA
     6841 ATCTGCTGCT TGCAAACAAA AAAACCACCG CTACCAGCGG TGGTTTGTTT GCCGGATCAA
     6901 GAGCTACCAA CTCTTTTTCC GAAGGTAACT GGCTTCAGCA GAGCGCAGAT ACCAAATACT
     6961 GTTCTTCTAG TGTAGCCGTA GTTAGGCCAC CACTTCAAGA ACTCTGTAGC ACCGCCTACA
     7021 TACCTCGCTC TGCTAATCCT GTTACCAGTG GCTGCTGCCA GTGGCGATAA GTCGTGTCTT
     7081 ACCGGGTTGG ACTCAAGACG ATAGTTACCG GATAAGGCGC AGCGGTCGGG CTGAACGGGG
     7141 GGTTCGTGCA CACAGCCCAG CTTGGAGCGA ACGACCTACA CCGAACTGAG ATACCTACAG
     7201 CGTGAGCTAT GAGAAAGCGC CACGCTTCCC GAAGGGAGAA AGGCGGACAG GTATCCGGTA
     7261 AGCGGCAGGG TCGGAACAGG AGAGCGCACG AGGGAGCTTC CAGGGGGAAA CGCCTGGTAT
     7321 CTTTATAGTC CTGTCGGGTT TCGCCACCTC TGACTTGAGC GTCGATTTTT GTGATGCTCG
     7381 TCAGGGGGGC GGAGCCTATG GAAAAACGCC AGCAACGCGG CCTTTTTACG GTTCCTGGCC
     7441 TTTTGCTGGC CTTTTGCTCA CATGTT
//
```

pAttbRfB2-actGFP: Plasmid used to generate *act5c*-GFP line

```
LOCUS       attBRfB2act_GFP        8432 bp    DNA     circular
FEATURES             Location/Qualifiers
     misc_feature    complement(7791..7811)
                     /note="T7"
     misc_feature    complement(2216..2500)
                     /note="attB"
     misc_feature    2173..2192
                     /note="T3"
     misc_feature    2138..2156
                     /note="M13R"
     misc_feature    6180..6379
                     /note="sv40 terminator"
     misc_feature    6500..6549
                     /note="3xP3 binding sites"
     misc_feature    6759..7684
                     /note="DsRed"
     misc_feature    6589..6596
                     /note="TATA"
     misc_feature    7747..7766
                     /note="attB2"
     misc_feature    2516..2537
                     /note="attB1"
     misc_feature    complement(5304..6083)
                     /note="GFP"
     misc_feature    2613..5181
                     /note="actin5C promoter"
     misc_feature    complement(5236..5257)
                     /note="attB4"
     misc_feature    6111..6132
                     /note="attB3"
     source          1..8432
                     /dnas_title="attBRfB2act_GFP_Sv40dsRED"
ORIGIN
        1 GTGGCACTTT TCGGGGAAAT GTGCGCGGAA CCCCTATTTG TTTATTTTTC TAAATACATT
       61 CAAATATGTA TCCGCTCATG AGACAATAAC CCTGATAAAT GCTTCAATAA TATTGAAAAA
      121 GGAAGAGTAT GAGTATTCAA CATTTCCGTG TCGCCCTTAT TCCCTTTTTT GCGGCATTTT
      181 GCCTTCCTGT TTTTGCTCAC CCAGAAACGC TGGTGAAAGT AAAAGATGCT GAAGATCAGT
      241 TGGGTGCACG AGTGGGTTAC ATCGAACTGG ATCTCAACAG CGGTAAGATC CTTGAGAGTT
      301 TTCGCCCCGA AGAACGTTTT CCAATGATGA GCACTTTTAA AGTTCTGCTA TGTGGCGCGG
      361 TATTATCCCG TATTGACGCC GGGCAAGAGC AACTCGGTCG CCGCATACAC TATTCTCAGA
      421 ATGACTTGGT TGAGTACTCA CCAGTCACAG AAAAGCATCT TACGGATGGC ATGACAGTAA
      481 GAGAATTATG CAGTGCTGCC ATAACCATGA GTGATAACAC TGCGGCCAAC TTACTTCTGA
      541 CAACGATCGG AGGACCGAAG GAGCTAACCG CTTTTTTGCA CAACATGGGG GNATCATGTA
      601 ACTCGCCTTN GATCGTTGGG AACCGGAGCT GAATGAAGCC ATACCAAACG ACGAGCGTGA
      661 CACCACGATG CCTGTAGCAA TGGCAACAAC GTTGCGCAAA CTATTAACTG GCGAACTACT
      721 TACTCTAGCT TCCCGGCAAC AATTAATAGA CTGGATGGAG GCGGATAAAG TTGCAGGACC
      781 ACTTCTGCGC TCGGCCCTTC CGGCTGGCTG GTTTATTGCT GATAAATCTG GAGCCGGTGA
      841 GCGTGGGTCT CGCGGTATCA TTGCAGCACT GGGGCCAGAT GGTAAGCCCT CCCGTATCGT
      901 AGTTATCTAC ACGACGGGGA GTCAGGCAAC TATGGATGAA CGAAATAGAC AGATCGCTGA
      961 GATAGGTGCC TCACTGATTA AGCATTGGTA ACTGTCAGAC CAAGTTTACT CATATATACT
     1021 TTAGATTGAT TTAAAACTTC ATTTTTAATT TAAAAGGATC TAGGTGAAGA TCCTTTTTGA
     1081 TAATCTCATG ACCAAAATCC CTTAACGTGA GTTTTCGTTC CACTGAGCGT CAGACCCCGT
     1141 AGAAAAGATC AAAGGATCTT CTTGAGATCC TTTTTTTCTG CGCGTAATCT GCTGCTTGCA
     1201 AACAAAAAAA CCACCGCTAC CAGCGGTGGT TTGTTTGCCG GATCAAGAGC TACCAACTCT
     1261 TTTTCCGAAG GTAACTGGCT TCAGCAGAGC GCAGATACCA AATACTGTCC TTCTAGTGTA
     1321 GCCGTAGTTA GGCCACCACT TCAAGAACTC TGTAGCACCG CCTACATACC TCGCTCTGCT
     1381 AATCCTGTTA CCAGTGGCTG CTGCCAGTGG CGATAAGTCG TGTCTTACCG GGTTGGACTC
     1441 AAGACGATAG TTACCGGATA AGGCGCAGCG GTCGGGCTGA ACGGGGGGTT CGTGCACACA
     1501 GCCCAGCTTG GAGCGAACGA CCTACACCGA ACTGAGATAC CTACAGCGTG AGCTATGAGA
```

1561 AAGCGCCACG CTTCCCGAAG GGAGAAAGGC GGACAGGTAT CCGGTAAGCG GCAGGGTCGG
1621 AACAGGAGAG CGCACGAGGG AGCTTCCAGG GGGAAACGCC TGGTATCTTT ATAGTCCTGT
1681 CGGGTTTCGC CACCTCTGAC TTGAGCGTCG ATTTTTGTGA TGCTCGTCAG GGGGGCGGAG
1741 CCTATGGAAA AACGCCAGCA ACGCGGCCTT TTTACGGTTC CTGGCCTTTT GCTGGCCTTT
1801 TGCTCACATG TTCTTTCCTG CGTTATCCCC TGATTCTGTG GATAACCGTA TTACCGCCTT
1861 TGAGTGAGCT GATACCGCTC GCCGCAGCCG AACGACCGAG CGCAGCGAGT CAGTGAGCGA
1921 GGAAGCGGAA GAGCGCCCAA TACGCAAACC GCCTCTCCCC GCGCGTTGGC CGATTCATTA
1981 ATGCAGCTGG CACGACAGGT TTCCCGACTG GAAAGCGGGC AGTGAGCGCA ACGCAATTAA
2041 TGTGAGTTAG CTCACTCATT AGGCACCCCA GGCTTTACAC TTTATGCTTC CGGCTCGTAT
2101 GTTGTGTGGA ATTGTGAGCG GATAACAATT TCACACAGGA AACAGCTATG ACCATGATTA
2161 CGCCAAGCTC GAAATTAACC CTCACTAAAG GGAACAAAAG CTGGCTAGAA CTAGTGTCGA
2221 CATGCCCGCC GTGACCGTCG AGAACCCGCT GACGCTGCCC CGCGTATCCG CACCCGCCGA
2281 CGCCGTCGCA CGTCCCGTGC TCACCGTGAC CACCGCGCCC AGCGGTTTCG AGGGCGAGGG
2341 CTTCCCGGTG CGCCGCGCGT TCGCCGGGAT CAACTACCGC CACCTCGACC CGTTCATCAT
2401 GATGGACCAG ATGGGTGAGG TGGAGTACGC GCCCGGGGAG CCCAAGGGCA CGCCCTGGCA
2461 CCCGCACCGC GGCTTCGAGA CCGTGACCTA CATCGTCGAC ACTAGTGGat catcaacaag
2521 tttGTACAAA AAAGCAGGCT GGTACCGGGC CCCCCGCTAG CGTCGACGGT ATCGATAAGC
2581 TTGATATCGA ATTCCTGCAG CCCGCGGCCG CAGCATGCAA TTCTATATTC TAAAAACACA
2641 AATGATACTT CTAAAAAAAA ATCATGAATG GCATCAACTC TGAATCAAAT CTTTGCAGAT
2701 GCACCTACTT CTCATTTCCA CTGTCACATC ATTTTTCCAG ATCTCGCTGC CTGTTATGTG
2761 GCCCACAAAC CAAGACACGT TTTATGGCCA TTAAAGCTGG CTGATCGTCG CCAAACACCA
2821 AATACATAAT GAATATGTAC ACATTCGAGA AAGAAGCGAT CAAAGAAGCG TCTTCGGGCG
2881 GAGTAGGAGA ATGCGGAGGA GAAGGAGAAC GAGCTGATCT AGTATCTCTC CACAATCCAA
2941 TGCCAACTGA CCAACTGGCC ATATTCGGAG CAATTTGAAG CCAATTTCCA TCGCCTGGCG
3001 ATCGCTCCAT TCTTGGCTAT ATGTTTTTCA CCGTTACCCG GGGCCATTTT CAAAGACTCG
3061 TCGGCAAGAT AAGATTGTGT CACTCGCTGT CTCTCTTCAT TTGTCGAAGA ATGCTGAGGA
3121 ATTTCGCGAT GACGTCGGCG AGTATTTTGA AGAATGAGAA TAATTTGTAT TTATACGAAA
3181 ATCAGTTAGT GGAATTTTCT ACAAAAACAT GTTATCTATA GATAATTTTG TTGCAAAATA
3241 TGTTGACTAT GACAAAGATT GTATGTATAT ACCTTTAATG TATTCTCATT TTCTTATGTA
3301 TTTATAATGG CAATGATGAT ACTGATGATA TTTTAAGATG ATGCCAGACC AAAAGGCTTG
3361 AATTTCTGCG TCTTTTGCCG AACGCAGTGC ATGTGCAATT GTTGTTTTTT GGAATATTCA
3421 ATTTTCGGAC TGTCCGCTTT GATTTCAGTT TCTTGGCTTA TTCAAAAAGC AAAGTAAAGC
3481 CAAAAAAGCG AGATGGCAAT ACCAAATGCG GCAAAACGGT AGTGGAAGGA AAGGGGTGCG
3541 GGGCAGCGGA AGGAAGGGTG GGGCGGGGCG TGGCGGGGTC TGTGGCTGGG CGCGACGTCA
3601 CCGACGTTGG AGCCACTCCT TTGACCATGT GTGCGTGTGT GTATTATTCG TGTCTCGCCA
3661 CTCGCCGGTT GTTTTTTTCT TTTTATGCTG CGCTCTCTCT AGCGCCATCT CGCTTACGCA
3721 TGCTCAACGC ACCGCATGTT GCCGTTTCCT TTTATGCGTC ATTTTGGCTC GAAATAGGCA
3781 ATTATTTAAA CAAAGATTAG TCAACGAAAA CGCTAAAATA AATAAGTCTA CAATATGGTT
3841 ACTTATTGCC ATGTGTGTGC AGCCAACGAT AGCAACAAAA GCAACAACAC AGGTGGCTTT
3901 CCCTCTTTCA CTTTTTGTTT GCAAGCCGCG TGCGAGCAAG ACGGCACGAC CGGCAAACGC
3961 AATTACGCTG ACAAAGAGCA GACGAAGTTT TGGCGAAAAA CATCAAGGCG CCTGATACGA
4021 ATGCATTTGC AATAACAATT GCGATATTTA ATATTGTTTA TGAAGCTGTT TGACTTCAAA
4081 ACACACAAAA AAAAAAATAA AACAAATTAT TTGAAAGAGA ATTAGGAATC GGACGCTTAT
4141 CGTTAGGGTA ACAACAAGAA ATGCTTACTG AGTCACAGCC TCTGGAAAAC TGCCGCAAGC
4201 CAGAGAGAGA GAGAAAAAGA GGGAGAGCAG CTTAGACCGC ATGTGCTTGT GTGTGAGGCG
4261 TCTCTCTCTT CGTCTCTGTT GCGCAAACGC ATAGACTGCA CTGAGAAAAT CGATTACCTA
4321 TTTTTTATGA ATGAATATTT GCACTATTAC TATTCAAAAC TATTAAGATA GCAATCACAT
4381 TCAATAGCCA AATACTATAC CACCTGAGCG ATGCAACGAA ATGATCAATT TGAGCAAAAA
4441 TGCTGCATAT TTAGGACGGC ATCATTATAG AAATGCTTCT TGCTGTGTAC TTTTCTCTCG
4501 TCTGGCAGCT GTTTCGCCGT TATTGTTAAA ACCGGCTTAA GTTAGGTGTG TTTTCTACGA
4561 CTAGTGAATG CCCTACTAGA AGATGTGTGT TGCACAAAAT GTCCCTGGAA TAACCAATTT
4621 GAAGTGCAGA TAGCAGTAAA CGTAAGCTAA TATGAATATT ATTTAACTGT AATGTTTTAA
4681 TATCGCTGGA CATTACTAAT AAACCCACTA TAAACACATG TACATATGTA TGTTTTGGCA
4741 TACAATGAGT AGTTGGGGAA AAAATGTGTA AAAGCACCGT GACCATCACA GCATAAAGAT
4801 AACCAGCTGA AGTATCGAAT ATGAGTAACC CCCAAATTGA ATCACATGCC GCAACTGATA
4861 GGACCCATGG AAGTACACTC TTCATGGCGA TATACAAGAC ACACACAAGC ACGAACACCC
4921 AGTTGCGGAG GAAATTCTCC GTAAATGAAA ACCCAATCGG CGAACAATTC ATACCCATAT
4981 ATGGTAAAAG TTTTGAACGC GACTTGAGAG CGGAGAGCAT TGCGGCTGAT AAGGTTTTAG
5041 CGCTAAGCGG GCTTTATAAA ACGGGCTGCG GGACCAGTTT TCATATCACT ACCGTTTGAG
5101 TTCTTGTGCT GTGTGGATAC TCCTCCCGAC ACAAAGCCGC TCCATCAGCC AGCAGTCGTC
5161 TAATCCAGAG ACCCCGGATC CCCGGGCTGC AGGAATTCGA TATCAAGCTT ATCGATACCG
5221 TCGACCTCGA GCACCCAACT TTTCTATACA AAGTTGGTAC CGGGCCCCCC GCTAGCGTCG
5281 ACGGTATCGA TAAGCTTGAT ATCGAATTCG TTAACAGATC CACCGGTCGC CACCATGGTG
5341 AGCAAGGGCG AGGAGCTGTT CACCGGGGTG GTGCCCATCC TGGTCGAGCT GGACGGCGAC
5401 GTAAACGGCC ACAAGTTCAG CGTGTCCGGC GAGGGCGAGG GCGATGCCAC CTACGGCAAG
5461 CTGACCCTGA AGTTCATCTG CACCACCGGC AAGCTGCCCG TGCCCTGGCC CACCCTCGTG
5521 ACCACCCTGA CCTACGGCGT GCAGTGCTTC AGCCGCTACC CCGACCACAT GAAGCAGCAC
5581 GACTTCTTCA AGTCCGCCAT GCCCGAAGGC TACGTCCAGG AGCGCACCAT CTTCTTCAAG
5641 GACGACGGCA ACTACAAGAC CCGCGCCGAG GTGAAGTTCG AGGGCGACAC CCTGGTGAAC
5701 CGCATCGAGC TGAAGGGCAT CGACTTCAAG GAGGACGGCA ACATCCTGGG GCACAAGCTG
5761 GAGTACAACT ACAACAGCCA CAACGTCTAT ATCATGGCCG acAAGCAGAA GAACGGCATC
5821 AAGGTGAACT TCAAGATCCG CCACAACATC GAGGaCGGCA GCGTGCAGCT CGCCGACCAC
5881 TACCAGCAGA ACACCCCCAT CGGCGacGGC CCCGTGCTGC TGCCCGaCAA CCACTACCTG
5941 AGCACCCAGT CCGCCCTGAG CAAagaCCCC AACGAgaagC GCGATCACAT GGTCCTGCTG
6001 gaGTTCGTGA CCGCCGCCGG gatCacTCTC GGCATGGacG AgcTGTacaA GTaAAgCgGC
6061 CgcGGcTcGA GGGtACCTct AGaGCGGCCG CCACCGCGGT GGAGCTCGAG ACAACTTTGT
6121 ATAATAAAGT TGGTACCGGG CCCCCCGCTA GCGTCGACGG TATCGATAAG CTTGATATCG
6181 AATTCTCTAG ATCATAATCA GCCATACCAC ATTTGTAGAG GTTTTACTTG CTTTAAAAAA
6241 CCTCCCACAC CTCCCCCTGA ACCTGAAACA TAAAATGAAT GCAATTGTTG TTGTTAACTT
6301 GTTTATTGCA GCTTATAATG GTTACAAATA AAGCAATAGC ATCACAAATT TCACAAATAA
6361 AGCATTTTTC TTCACTGCAT TCTAGTTGTG GTTTGTCCAA ACTCATCAAT GTATCAAGGG

```
     6421 CGAATTCCTG CAGCCCACTT CCGGTATCTC GCGTTTGTTT GATCGCACGG TTCCCACAAT
     6481 GGTTAATTCG AGCTCGCCCG GGGATCTAAT TCAATTAGAG ACTAATTCAA TTAGAGCTAA
     6541 TTCAATTAGG ATCCAAGCTT ATCGATTTCG AACCCTCGAC CGCCGGAGTA TAAATAGAGG
     6601 CGCTTCGTCT ACGGAGCGAC AATTCAATTC AAACAAGCAA AGTGAACACG TCGCTAAGCG
     6661 AAAGCTAAGC AAATAAACAA GCGCAGCTGA ACAAGCTAAA CAATCGGGGT ACCGCTAGAG
     6721 TCGACGGTAC CGCGGGCCCG GGATCCACCG GTCGCCACCA TGGTGCGCTC CTCCAAGAAC
     6781 GTCATCAAGG AGTTCATGCG CTTCAAGGTG CGCATGGAGG GCACCGTGAA CGGCCACGAG
     6841 TTCGAGATCG AGGGCGAGGG CGAGGGCCGC CCCTACGAGG GCCACAACAC CGTGAAGCTG
     6901 AAGGTGACCA AGGGCGGCCC CCTGCCCTTC GCCTGGGACA TCCTGTCCCC CCAGTTCCAG
     6961 TACGGCTCCA AGGTGTACGT GAAGCACCCC GCCGACATCC CCGACTACAA GAAGCTGTCC
     7021 TTCCCCGAGG GCTTCAAGTG GGAGCGCGTG ATGAACTTCG AGGACGGCGG CGTGGTGACC
     7081 GTGACCCAGG ACTCCTCCCT GCAGGACGGC TGCTTCATCT ACAAGGTGAA GTTCATCGGC
     7141 GTGAACTTCC CCTCCGACGG CCCCGTAATG CAGAAGAAGA CCATGGGCTG GGAGGCCTCC
     7201 ACCGAGCGCC TGTACCCCCG CGACGGCGTG CTGAAGGGCG AGATCCACAA GGCCCTGAAG
     7261 CTGAAGGACG GCGGCCACTA CCTGGTGGAG TTCAAGTCCA TCTACATGGC CAAGAAGCCC
     7321 GTGCAGCTGC CCGGCTACTA CTACGTGGAC TCCAAGCTGG ACATCACCTC CCACAACGAG
     7381 GACTACACCA TCGTGGACCA GTACGAGCGC ACCGAGGGCC GCCACCACCT GTTCCTGTAG
     7441 CGGCCGCGAC TCTAGATCAT AATCAGCCAT ACCACATTTG TAGAGGTTTT ACTTGCTTTA
     7501 AAAAACCTCC CACACCTCCC CCTGAACCTG AAACATAAAA TGAATGCAAT TGTTGTTGTT
     7561 AACTTGTTTA TTGCAGCTTA TAATGGTTAC AAATAAAGCA ATAGCATCAC AAATTTCACA
     7621 AATAAAGCAT TTTTCTTCAC TGCATTCTAG TTGTGGTTTG TCCAAACTCA TCAATGTATC
     7681 TTAAAGCTTA TCGATACGCG TACGGCGCGC CTAGAGCGGC CGCCACCGCG GTGGAGCTCG
     7741 AGTACCCAGC TTTcttgtac aaagtggttg atCGGTACGT ACCCAATTCG CCCTATAGTG
     7801 AGTCGTATTA CAATTCACTG GCCGTCGTTT TACAACGTCG TGACTGGGAA AACCCTGGCG
     7861 TTACCCAACT TAATCGCCTT GCAGCACATC CCCCTTTCGC CAGCTGGCGT AATAGCGAAG
     7921 AGGCCCGCAC CGATCGCCCT TCCCAACAGT TGCGCAGCCT GAATGGCGAA TGGAAATTGT
     7981 AAGCGTTAAT ATTTTGTTAA AATTCGCGTT AAATTTTTGT TAAATCAGCT CATTTTTTAA
     8041 CCAATAGGCC GAAATCGGCA AAATCCCTTA TAAATCAAAA GAATAGACCG AGATAGGGTT
     8101 GAGTGTTGTT CCAGTTTGGA ACAAGAGTCC ACTATTAAAG AACGTGGACT CCAACGTCAA
     8161 AGGGCGAAAA ACCGTCTATC AGGGCGATGG CCCACTACGT GAACCATCAC CCTAATCAAG
     8221 TTTTTTGGGG TCGAGGTGCC GTAAAGCACT AAATCGGAAC CCTAAAGGGA GCCCCCGATT
     8281 TAGAGCTTGA CGGGGAAAGC CGGCGAACGT GGCGAGAAAG GAAGGGAAGA AAGCGAAAGG
     8341 AGCGGGCGCT AGGGCGCTGG CAAGTGTAGC GGTCACGCTG CGCGTAACCA CCACACCCGC
     8401 CGCGCTTAAT GCGCCGCTAC AGGGCGCGTC AG
//
```

pAttbRfB2-G12GFP: Plasmid used to generate *G12*-GFP line

```
LOCUS       attBrf2G12_GFP_S        7012 bp    DNA     circular
FEATURES             Location/Qualifiers
     misc_feature    complement(6371..6391)
                     /note="T7"
     misc_feature    complement(2216..2500)
                     /note="attB"
     misc_feature    2173..2192
                     /note="T3"
     misc_feature    2138..2156
                     /note="M13R"
     gene            4760..4959
                     /note="sv40 terminator"
     misc_feature    5080..5129
                     /note="3xP3 binding sites"
     misc_feature    4691..4712
                     /note="attB3"
     misc_feature    5339..6264
                     /note="DsRed"
     misc_feature    5169..5176
                     /note="TATA"
     misc_feature    6327..6346
                     /note="attB2"
     misc_feature    2516..2537
                     /note="attB1"
     misc_feature    2541..3811
                     /note="G12 promoter"
     misc_feature    3723..3805
                     /note="G12 Exon1"
     misc_feature    complement(3884..4663)
                     /note="GFP"
     misc_feature    complement(3816..3837)
                     /note="attB4"
     source          1..7012
                     /dnas_title="attBrf2G12_GFP_Sv40dsRED"
ORIGIN
        1 GTGGCACTTT TCGGGGAAAT GTGCGCGGAA CCCCTATTTG TTTATTTTTC TAAATACATT
       61 CAAATATGTA TCCGCTCATG AGACAATAAC CCTGATAAAT GCTTCAATAA TATTGAAAAA
      121 GGAAGAGTAT GAGTATTCAA CATTTCCGTG TCGCCCTTAT TCCCTTTTTT GCGGCATTTT
      181 GCCTTCCTGT TTTTGCTCAC CCAGAAACGC TGGTGAAAGT AAAAGATGCT GAAGATCAGT
      241 TGGGTGCACG AGTGGGTTAC ATCGAACTGG ATCTCAACAG CGGTAAGATC CTTGAGAGTT
      301 TTCGCCCCGA AGAACGTTTT CCAATGATGA GCACTTTTAA AGTTCTGCTA TGTGGCGCGG
```

```
 361 TATTATCCCG TATTGACGCC GGGCAAGAGC AACTCGGTCG CCGCATACAC TATTCTCAGA
 421 ATGACTTGGT TGAGTACTCA CCAGTCACAG AAAAGCATCT TACGGATGGC ATGACAGTAA
 481 GAGAATTATG CAGTGCTGCC ATAACCATGA GTGATAACAC TGCGGCCAAC TTACTTCTGA
 541 CAACGATCGG AGGACCGAAG GAGCTAACCG CTTTTTTGCA CAACATGGGG GNATCATGTA
 601 ACTCGCCTTN GATCGTTGGG AACCGGAGCT GAATGAAGCC ATACCAAACG ACGAGCGTGA
 661 CACCACGATG CCTGTAGCAA TGGCAACAAC GTTGCGCAAA CTATTAACTG GCGAACTACT
 721 TACTCTAGCT TCCCGGCAAC AATTAATAGA CTGGATGGAG GCGGATAAAG TTGCAGGACC
 781 ACTTCTGCGC TCGGCCCTTC CGGCTGGCTG GTTTATTGCT GATAAATCTG GAGCCGGTGA
 841 GCGTGGGTCT CGCGGTATCA TTGCAGCACT GGGGCCAGAT GGTAAGCCCT CCCGTATCGT
 901 AGTTATCTAC ACGACGGGGA GTCAGGCAAC TATGGATGAA CGAAATAGAC AGATCGCTGA
 961 GATAGGTGCC TCACTGATTA AGCATTGGTA ACTGTCAGAC CAAGTTTACT CATATATACT
1021 TTAGATTGAT TTAAAACTTC ATTTTTAATT TAAAAGGATC TAGGTGAAGA TCCTTTTTGA
1081 TAATCTCATG ACCAAAATCC CTTAACGTGA GTTTTCGTTC CACTGAGCGT CAGACCCCGT
1141 AGAAAAGATC AAAGGATCTT CTTGAGATCC TTTTTTTCTG CGCGTAATCT GCTGCTTGCA
1201 AACAAAAAAA CCACCGCTAC CAGCGGTGGT TTGTTTGCCG GATCAAGAGC TACCAACTCT
1261 TTTTCCGAAG GTAACTGGCT TCAGCAGAGC GCAGATACCA AATACTGTCC TTCTAGTGTA
1321 GCCGTAGTTA GGCCACCACT TCAAGAACTC TGTAGCACCG CCTACATACC TCGCTCTGCT
1381 AATCCTGTTA CCAGTGGCTG CTGCCAGTGG CGATAAGTCG TGTCTTACCG GGTTGGACTC
1441 AAGACGATAG TTACCGGATA AGGCGCAGCG GTCGGGCTGA ACGGGGGGTT CGTGCACACA
1501 GCCCAGCTTG GAGCGAACGA CCTACACCGA ACTGAGATAC CTACAGCGTG AGCTATGAGA
1561 AAGCGCCACG CTTCCCGAAG GGAGAAAGGC GGACAGGTAT CCGGTAAGCG GCAGGGTCGG
1621 AACAGGAGAG CGCACGAGGG AGCTTCCAGG GGGAAACGCC TGGTATCTTT ATAGTCCTGT
1681 CGGGTTTCGC CACCTCTGAC TTGAGCGTCG ATTTTTGTGA TGCTCGTCAG GGGGGCGGAG
1741 CCTATGGAAA AACGCCAGCA ACGCGGCCTT TTTACGGTTC CTGGCCTTTT GCTGGCCTTT
1801 TGCTCACATG TTCTTTCCTG CGTTATCCCC TGATTCTGTG GATAACCGTA TTACCGCCTT
1861 TGAGTGAGCT GATACCGCTC GCCGCAGCCG AACGACCGAG CGCAGCGAGT CAGTGAGCGA
1921 GGAAGCGGAA GAGCGCCCAA TACGCAAACC GCCTCTCCCC GCGCGTTGGC CGATTCATTA
1981 ATGCAGCTGG CACGACAGGT TTCCCGACTG GAAAGCGGGC AGTGAGCGCA ACGCAATTAA
2041 TGTGAGTTAG CTCACTCATT AGGCACCCCA GGCTTTACAC TTTATGCTTC CGGCTCGTAT
2101 GTTGTGTGGA ATTGTGAGCG GATAACAATT TCACACAGGA AACAGCTATG ACCATGATTA
2161 CGCCAAGCTC GAAATTAACC CTCACTAAAG GGAACAAAAG CTGGCTAGAA CTAGTGTCGA
2221 CATGCCCGCC GTGACCGTCG AGAACCCGCT GACGCTGCCC CGCGTATCCG CACCCGCCGA
2281 CGCCGTCGCA CGTCCCGTGC TCACCGTGAC CACCGCGCCC AGCGGTTTCG AGGGCGAGGG
2341 CTTCCCGGTG CGCCGCGCGT TCGCCGGGAT CAACTACCGC CACCTCGACC CGTTCATCAT
2401 GATGGACCAG ATGGGTGAGG TGGAGTACGC GCCCGGGGAG CCCAAGGGCA CGCCCTGGCA
2461 CCCGCACCGC GGCTTCGAGA CCGTGACCTA CATCGTCGAC ACTAGTGgat catcaacaag
2521 tttGTACAAA AAGCAGGCT ggtaccCACA ATACCGGCCC TGAATCTTTA AAGACCAGG
2581 TAATAATCCT AAACTTAAAC TAGTTTAGCG ATCCAGCAAC TAATTAGGTC CTAGAACTCA
2641 ATCCATTCTA GTCTTGGAAC TCTATCCTAC TCACGTACCA GCCCTGAAAA TGATTAAGAA
2701 ACTGAGCCCA GGTCAGTCCT GGAACCGACC ATGAAACGAT ACCATTCTTT GAAATCCCGG
2761 AAGTCCCAGA AGTACCCATA CCAGTCCTGG AACAGATACT GTACCCATCC TAGGAACTTG
2821 TCCAAATTTG ATAATATTCC CGACACTGAT AGTAAACTCA TATTGGTCTA CGAAATGATC
2881 CCTAAGCCAG CCATACCGAT TCAGGAACTT GTACGGAATC CTCACCGGTT CTCAAACTAA
2941 TTCCTTATCC ATATAGATCC TGGAATTGAT CCATAACCTA TACTGGTCCT GGATTTTTCC
3001 CCATGTCAAA CTGATCCTAG AACTCATTCC TACTTTATTC CAATTCATGG AAAACTGTAC
3061 CATAGACCAA TCTCAGTGAA AGAACGAATC GTGAACCTGT CCCGAAACAA GAAGCCGATA
3121 CCAAACCTCC TGCTCTAATC CGGCAACTGA TCTCCCTACT GGTGAATCAA CCATTCCCGA
3181 CGGTACTTTA GAGGAAAAAC TCAGCGTAGA AATTTAGCTA CGACCAAGTA GCCAGCCACT
3241 AGACTGAAGA TCTCCATCCA GAAACTAAAT CATGTGGAAA TCATTCCATT GCAACGAACT
3301 GTGTCCAAGG TCCAGGTTGG CCTTATCGGT TGCCTTAGAA ACATCTGTTC GCAGAAGAAG
3361 TACTGGATTG AACCGTAAGT WGAGAGTTCC AAGGGAAKAA GTTCCCATCC AAACTAAAAG
3421 GGCGTGATCA GTAATCAATT AAGCACACAA ATTCCATGCT AAATCATCAT TATCGCTAAT
3481 CGGATTATGT TATTATAATA AGTATGGACC ACTTGACCAG TGATGGGTAG TGGTCGGTGG
3541 ACTTCTTGAG AGTGAGTGTG GGACAGACGG GCTGAAAGTC CAGTACCTGC CAACAGATGT
3601 ACACTCGAAA GGCACAGATA ACAGCACTCC ACTCCTTSGA CAGCTGGTTA ATCGACTGTT
3661 GACCGCTCAA GATACTGACA GCAGYGGACG TATAAAAGCT ACTGGCTGGT TGGTAGAGAA
3721 ATCATTCCCA AGCCTACTAC AGCTCAGTAC AGCAGTACCA CAGCATCGRA ACCAGTGCAA
3781 AAGCAACCCA ATCAAATCTA GCATCctcga gCACCCAACT TTTCTATACA AAGTTGGTAC
3841 CGGGCCCCCC GCTAGCGTCG ACGGTATCGA TAAGCTTGAT ATCGAATTCG TTAACAGATC
3901 CACCGGTCGC CACCATGGTG AGCAAGGGCG AGGAGCTGTT CACCGGGGTG GTGCCCATCC
3961 TGGTCGAGCT GGACGGCGAC GTAAACGGCC ACAAGTTCAG CGTGTCCGGC GAGGGCGAGG
4021 GCGATGCCAC CTACGGCAAG CTGACCCTGA AGTTCATCTG CACCACCGGC AAGCTGCCCG
4081 TGCCCTGGCC CACCCTCGTG ACCACCCTGA CCTACGGCGT GCAGTGCTTC AGCCGCTACC
4141 CCGACCACAT GAAGCAGCAC GACTTCTTCA AGTCCGCCAT GCCCGAAGGC TACGTCCAGG
4201 AGCGCACCAT CTTCTTCAAG GACGACGGCA ACTACAAGAC CCGCGCCGAG GTGAAGTTCG
4261 AGGGCGACAC CCTGGTGAAC CGCATCGAGC TGAAGGGCAT CGACTTCAAG GAGGACGGCA
4321 ACATCCTGGG GCACAAGCTG GAGTACAACT ACAACAGCCA CAACGTCTAT ATCATGGCCG
4381 acAAGCAGAA GAACGGCATC AAGGTGAACT TCAAGATCCG CCACAACATC GAGGaCGGCA
4441 GCGTGCAGCT CGCCGACCAC TACCAGCAGA ACACCCCCAT CGGCGacGGC CCCGTGCTGC
4501 TGCCCGaCAA CCACTACCTG AGCACCCAGT CCGCCCTGAG CAAagaCCCC AACGAgaagC
4561 GCGATCACAT GGTCCTGCTG gaGTTCGTGA CCGCCGCCGG gatCacTCTC GGCATGGacG
4621 AgcTGTacaA GTaAAgCgGC CgcGGcTcGA GGGtACCTct AGaGCGGCCG CCACCGCGGT
4681 GGAGCTCGAG ACAACTTTGT ATAATAAAGT TGGTACCGGG CCCCCCGCTA GCGTCGACGG
4741 TATCGATAAG CTTGATATCG AATTCTCTAG ATCATAATCA GCCATACCAC ATTTGTAGAG
4801 GTTTTACTTG CTTTAAAAAA CCTCCCACAC CTCCCCCTGA ACCTGAAACA TAAAATGAAT
4861 GCAATTGTTG TTGTTAACTT GTTTATTGCA GCTTATAATG GTTACAAATA AAGCAATAGC
4921 ATCACAAATT TCACAAATAA AGCATTTTTC TTCACTGCAT TCTAGTTGTG GTTTGTCCAA
4981 ACTCATCAAT GTATCAAGGG CGAATTCCTG CAGCCCACTT CCGGTATCTC GCGTTTGTTT
5041 GATCGCACGG TTCCCACAAT GGTTAATTCG AGCTCGCCCG GGGATCTAAT TCAATTAGAG
5101 ACTAATTCAA TTAGAGCTAA TTCAATTAGG ATCCAAGCTT ATCGATTTCG AACCCTCGAC
5161 CGCCGGAGTA TAAATAGAGG CGCTTCGTCT ACGGAGCGAC AATTCAATTC AAACAAGCAA
```

```
     5221 AGTGAACACG TCGCTAAGCG AAAGCTAAGC AAATAAACAA GCGCAGCTGA ACAAGCTAAA
     5281 CAATCGGGGT ACCGCTAGAG TCGACGGTAC CGCGGGCCCG GGATCCACCG GTCGCCACCA
     5341 TGGTGCGCTC CTCCAAGAAC GTCATCAAGG AGTTCATGCG CTTCAAGGTG CGCATGGAGG
     5401 GCACCGTGAA CGGCCACGAG TTCGAGATCG AGGGCGAGGG CGAGGGCCGC CCCTACGAGG
     5461 GCCACAACAC CGTGAAGCTG AAGGTGACCA AGGGCGGCCC CCTGCCCTTC GCCTGGGACA
     5521 TCCTGTCCCC CCAGTTCCAG TACGGCTCCA AGGTGTACGT GAAGCACCCC GCCGACATCC
     5581 CCGACTACAA GAAGCTGTCC TTCCCCGAGG GCTTCAAGTG GGAGCGCGTG ATGAACTTCG
     5641 AGGACGGCGG CGTGGTGACC GTGACCCAGG ACTCCTCCCT GCAGGACGGC TGCTTCATCT
     5701 ACAAGGTGAA GTTCATCGGC GTGAACTTCC CCTCCGACGG CCCCGTAATG CAGAAGAAGA
     5761 CCATGGGCTG GGAGGCCTCC ACCGAGCGCC TGTACCCCCG CGACGGCGTG CTGAAGGGCG
     5821 AGATCCACAA GGCCCTGAAG CTGAAGGACG GCGGCCACTA CCTGGTGGAG TTCAAGTCCA
     5881 TCTACATGGC CAAGAAGCCC GTGCAGCTGC CCGGCTACTA CTACGTGGAC TCCAAGCTGG
     5941 ACATCACCTC CCACAACGAG GACTACACCA TCGTGGACCA GTACGAGCGC ACCGAGGGCC
     6001 GCCACCACCT GTTCCTGTAG CGGCCGCGAC TCTAGATCAT AATCAGCCAT ACCACATTTG
     6061 TAGAGGTTTT ACTTGCTTTA AAAAACCTCC CACACCTCCC CCTGAACCTG AAACATAAAA
     6121 TGAATGCAAT TGTTGTTGTT AACTTGTTTA TTGCAGCTTA TAATGGTTAC AAATAAAGCA
     6181 ATAGCATCAC AAATTTCACA AATAAAGCAT TTTTTCACT TGCATTCTAG TTGTGGTTTG
     6241 TCCAAACTCA TCAATGTATC TTAAAGCTTA TCGATACGCG TACGGCGCGC CTAGAGCGGC
     6301 CGCCACCGCG GTGGAGCTCG AGTACCCAGC TTTcttgtac aaagtggttg atCGGTACGT
     6361 ACCCAATTCG CCCTATAGTG AGTCGTATTA CAATTCACTG GCCGTCGTTT TACAACGTCG
     6421 TGACTGGGAA AACCCTGGCG TTACCCAACT TAATCGCCTT GCAGCACATC CCCCTTTCGC
     6481 CAGCTGGCGT AATAGCGAAG AGGCCCGCAC CGATCGCCCT TCCCAACAGT TGCGCAGCCT
     6541 GAATGGCGAA TGGAAATTGT AAGCGTTAAT ATTTTGTTAA AATTCGCGTT AAATTTTTGT
     6601 TAAATCAGCT CATTTTTTAA CCAATAGGCC GAAATCGGCA AAATCCCTTA TAAATCAAAA
     6661 GAATAGACCG AGATAGGGTT GAGTGTTGTT CCAGTTTGGA ACAAGAGTCC ACTATTAAAG
     6721 AACGTGGACT CCAACGTCAA AGGGCGAAAA ACCGTCTATC AGGGCGATGG CCCACTACGT
     6781 GAACCATCAC CCTAATCAAG TTTTTTGGGG TCGAGGTGCC GTAAAGCACT AAATCGGAAC
     6841 CCTAAAGGGA GCCCCCGATT TAGAGCTTGA CGGGGAAAGC CGGCGAACGT GGCGAGAAAG
     6901 GAAGGGAAGA AAGCGAAAGG AGCGGGCGCT AGGGCGCTGG CAAGTGTAGC GGTCACGCTG
     6961 CGCGTAACCA CCACACCCGC CGCGCTTAAT GCGCCGCTAC AGGGCGCGTC AG
//
```

pAttbRfB2-vas2RFP: Plasmid used to generate *vasa*-tdTomato line

```
LOCUS       pattB-vas2Tomato        8675 bp    DNA     circular
FEATURES             Location/Qualifiers
     misc_feature    complement(8034..8054)
                     /note="T7"
     misc_feature    complement(2216..2500)
                     /note="attB"
     misc_feature    2173..2192
                     /note="T3"
     misc_feature    2138..2156
                     /note="M13R"
     misc_feature    2575..4865
                     /dnas_title="5'vasa 5' region"
                     /vntifkey="21"
                     /label=5'vasa 5' region
     misc_feature    4931..6359
                     /note="tdTomato"
     misc_feature    6494..6693
                     /note="sv40 terminator"
     gene            7002..7718
                     /note="EYFP (Clontech)"
     misc_feature    6831..6838
                     /note="TATA"
     misc_feature    7725..7979
                     /note="SV40 term sequences"
     source          1..8675
                     /dnas_title="pattB-vas2Tomato-YFP"
ORIGIN
        1 GTGGCACTTT TCGGGGAAAT GTGCGCGGAA CCCCTATTTG TTTATTTTTC TAAATACATT
       61 CAAATATGTA TCCGCTCATG AGACAATAAC CCTGATAAAT GCTTCAATAA TATTGAAAAA
      121 GGAAGAGTAT GAGTATTCAA CATTTCCGTG TCGCCCTTAT TCCCTTTTTT GCGGCATTTT
      181 GCCTTCCTGT TTTTGCTCAC CCAGAAACGC TGGTGAAAGT AAAAGATGCT GAAGATCAGT
      241 TGGGTGCACG AGTGGGTTAC ATCGAACTGG ATCTCAACAG CGGTAAGATC CTTGAGAGTT
      301 TTCGCCCCGA AGAACGTTTT CCAATGATGA GCACTTTTAA AGTTCTGCTA TGTGGCGCGG
      361 TATTATCCCG TATTGACGCC GGGCAAGAGC AACTCGGTCG CCGCATACAC TATTCTCAGA
      421 ATGACTTGGT TGAGTACTCA CCAGTCACAG AAAAGCATCT TACGGATGGC ATGACAGTAA
      481 GAGAATTATG CAGTGCTGCC ATAACCATGA GTGATAACAC TGCGGCCAAC TTACTTCTGA
      541 CAACGATCGG AGGACCGAAG GAGCTAACCG CTTTTTTGCA CAACATGGGG GNATCATGTA
      601 ACTCGCCTTN GATCGTTGGG AACCGGAGCT GAATGAAGCC ATACCAAACG ACGAGCGTGA
      661 CACCACGATG CCTGTAGCAA TGGCAACAAC GTTGCGCAAA CTATTAACTG GCGAACTACT
      721 TACTCTAGCT TCCCGGCAAC AATTAATAGA CTGGATGGAG GCGGATAAAG TTGCAGGACC
      781 ACTTCTGCGC TCGGCCCTTC CGGCTGGCTG GTTTATTGCT GATAAATCTG GAGCCGGTGA
      841 GCGTGGGTCT CGCGGTATCA TTGCAGCACT GGGGCCAGAT GGTAAGCCCT CCCGTATCGT
      901 AGTTATCTAC ACGACGGGGA GTCAGGCAAC TATGGATGAA CGAAATAGAC AGATCGCTGA
      961 GATAGGTGCC TCACTGATTA AGCATTGGTA ACTGTCAGAC CAAGTTTACT CATATATACT
```

```
1021 TTAGATTGAT TTAAAACTTC ATTTTTAATT TAAAAGGATC TAGGTGAAGA TCCTTTTTGA
1081 TAATCTCATG ACCAAAATCC CTTAACGTGA GTTTTCGTTC CACTGAGCGT CAGACCCCGT
1141 AGAAAAGATC AAAGGATCTT CTTGAGATCC TTTTTTTCTG CGCGTAATCT GCTGCTTGCA
1201 AACAAAAAAA CCACCGCTAC CAGCGGTGGT TTGTTTGCCG GATCAAGAGC TACCAACTCT
1261 TTTTCCGAAG GTAACTGGCT TCAGCAGAGC GCAGATACCA AATACTGTCC TTCTAGTGTA
1321 GCCGTAGTTA GGCCACCACT TCAAGAACTC TGTAGCACCG CCTACATACC TCGCTCTGCT
1381 AATCCTGTTA CCAGTGGCTG CTGCCAGTGG CGATAAGTCG TGTCTTACCG GGTTGGACTC
1441 AAGACGATAG TTACCGGATA AGGCGCAGCG GTCGGGCTGA ACGGGGGGTT CGTGCACACA
1501 GCCCAGCTTG GAGCGAACGA CCTACACCGA ACTGAGATAC CTACAGCGTG AGCTATGAGA
1561 AAGCGCCACG CTTCCCGAAG GGAGAAAGGC GGACAGGTAT CCGGTAAGCG GCAGGGTCGG
1621 AACAGGAGAG CGCACGAGGG AGCTTCCAGG GGGAAACGCC TGGTATCTTT ATAGTCCTGT
1681 CGGGTTTCGC CACCTCTGAC TTGAGCGTCG ATTTTTGTGA TGCTCGTCAG GGGGGCGGAG
1741 CCTATGGAAA AACGCCAGCA ACGCGGCCTT TTTACGGTTC CTGGCCTTTT GCTGGCCTTT
1801 TGCTCACATG TTCTTTCCTG CGTTATCCCC TGATTCTGTG GATAACCGTA TTACCGCCTT
1861 TGAGTGAGCT GATACCGCTC GCCGCAGCCG AACGACCGAG CGCAGCGAGT CAGTGAGCGA
1921 GGAAGCGGAA GAGCGCCCAA TACGCAAACC GCCTCTCCCC GCGCGTTGGC CGATTCATTA
1981 ATGCAGCTGG CACGACAGGT TTCCCGACTG GAAAGCGGGC AGTGAGCGCA ACGCAATTAA
2041 TGTGAGTTAG CTCACTCATT AGGCACCCCA GGCTTTACAC TTTATGCTTC CGGCTCGTAT
2101 GTTGTGTGGA ATTGTGAGCG GATAACAATT TCACACAGGA AACAGCTATG ACCATGATTA
2161 CGCCAAGCTC GAAATTAACC CTCACTAAAG GGAACAAAAG CTGGCTAGAA CTAGTGTCGA
2221 CATGCCCGCC GTGACCGTCG AGAACCCGCT GACGCTGCCC CGCGTATCCG CACCCGCCGA
2281 CGCCGTCGCA CGTCCCGTGC TCACCGTGAC CACCGCGCCC AGCGGTTTCG AGGGCGAGGG
2341 CTTCCCGGTG CGCCGCGCGT TCGCCGGGAT CAACTACCGC CACCTCGACC CGTTCATCAT
2401 GATGGACCAG ATGGGTGAGG TGGAGTACGC GCCCGGGGAG CCCAAGGGCA CGCCCTGGCA
2461 CCCGCACCGC GGCTTCGAGA CCGTGACCTA CATCGTCGAC ACTAGTGgat catcaacaag
2521 TTTGTACAAA AAAGCAGGCT GGTACCGGGC CCCCCGCTAG CGTCGACGGT ATcgatgtag
2581 aacgcgagca aattcttttc cttccatgac agcagcagct acagtgggaa gccgaacgtc
2641 agacgtgttt gacatgccga actgggcggg aaaattacag cgtgcgcttt gttttcaagc
2701 aaatcacaac tcgctgcaaa caaaaccgtt gagaaattga ttgttttata atttgtattg
2761 tattttattt gttataataa actaaaaaga catacttttt gcatatttta tacataaaaa
2821 catacatgca gcattataaa acacatataa accctccctg tagagtcccg tatcgaaatc
2881 ttccatccta gttgcacagt acgacggacg agtaggccgt gtccgtgcaa attccagctt
2941 ttagcagtct tttgctcgga gcactcgcgg cgagtcggag gtttctgctg aggtgcttag
3001 cgctaaatta gccaattgct tttgcaagtg aaataaccag ccgaatagta cttcaaaact
3061 caggtaagtg aactagtttt atagaacaaa tgtttgtttg ttagaagtta gtgaagtgtt
3121 tgtgaaaaaa atctctcatt tcggcaaaac taacgtaact gatttcaaat tgaattattg
3181 ttttgtgatg ttatattatt tcatccagtt gattagtatt ttcttagtta tgttcaaaat
3241 acagttaaat taaatttcat ttcatttact cataaaataa tctcttggct tatttaattt
3301 ttctcgaatt cgcttgtatt gttcagtagc acgcgccatt cgccctttgt ttcattttgt
3361 acctgctccc actaacacac tggcagtgcg aaacaaaagc cttcgcacgc gttgctggta
3421 ttagagtgtg tgcgtgtgtg tgttgagcgc tctgtcaaaa tcggctgttg ccgccggtac
3481 cgaaattgcc tgttcgcacg ctgttcgtaa acattccgtg gtgtgtatcg tgtgttgtgc
3541 atgttgcgcg cctccccccct tttgatagca ggctgccgtg gctgccgtgg tgtgtggcgc
3601 agttgagttt ttggattaat tttctaagga aatggcacga gaagagcggt ggcagtgtgt
3661 tggtttgctc tgtcccttcc tttctgtgtg aagtgttctt acagcacagc acgtatccac
3721 caccgcacac agagcaggca aggaagtgga agtgaacaag tgtgctgcgc atgcatgtgt
3781 gtgggggggca ttttagctga gatcgtcgtt atttgagaag cggtataggg gccagtcggt
3841 gtcgacgtac ggaagcggtt tagttttaat ccaagcgtat cccgtcgtgg agtggttgtg
3901 tggctctgtg tgctctcata tcagttccag agtgaggtta gtagaatcac agtccttggc
3961 ctttttcgtt acaagatatc cagaaggatg gcgttatttc cacagcttac catggtgctc
4021 ttgtttgctc gaatcagggg agaaaaacag tttcgtgttt catgaaccgc agttggcact
4081 ggagcggatt caaaagtctt cgatatgcaa tagataagag agtcgttggg gcatagttgg
4141 gaagcctttc cgagatgtgg agtttccgag aggagaaatg gtgctttcgt gcacgttccg
4201 ggacagcggg ccccgcgaag agcatctcgt tgtcgttcat ccggcaataa ttgatgcgaa
4261 aagcgcgcgc gccactggct tagcgcagtg tacacagtga tattcaccta cacacacaga
4321 ggcacacgcc ttcacacgcg cgcgtgcttc aaaggctact tcggtggcgg tgtgtgaggt
4381 cgcttgcaat ggacaatgaa aatttcgctg gaaaatacca tcgtctcttt aggttgcaat
4441 gggtgcgggt agagcggtgg tcgtcgatat tggtggtgta gtgtgtgtgt gtgtgtgtgt
4501 gtgtgtgtgt gtgtgtgtgt gtgtgtgtgt gtgtgtgtgt gtgtgtgtgt gtgtgtgtgt
4561 gtgtgtgtgt gtgtgtgcaa cggcaattat tttttgtaat atttcgacca tctttctttc
4621 tctctctcca cgtgctgctg ctgttgctgc tgctgctgca ttgcatgttc cactattcct
4681 ctcggtttgt gcctgcggac gccattgcta gtcgaaagag agtcgccgtt agtcgcgctt
4741 cgagcaacgg acacgttttt tggttgaaac caacagcttt tttcatcttc gggagacaca
4801 cagatctcga atcgtacatt cccataagga gaattgtcat cttccggtga ataaagaaag
4861 gaaacggatc TCGAGCACCC AACTTTTCTA TACAAAGTTG GTACCGGGCC CCCCGCTAGC
4921 GTCGACAACC ATGGTGAGCA AGGGCGAGGA GGTCATCAAA GAGTTCATGC GCTTCAAGGT
4981 GCGCATGGAG GGCTCCATGA ACGGCCACGA GTTCGAGATC GAGGGCGAGG GCGAGGGCCG
5041 CCCCTACGAG GGCACCCAGA CCGCCAAGCT GAAGGTGACC AAGGGCGGCC CCCTGCCCTT
5101 CGCCTGGGAC ATCCTGTCCC CCCAGTTCAT GTACGGCTCC AAGGCGTACG TGAAGCACCC
5161 CGCCGACATC CCCGATTACA AGAAGCTGTC CTTCCCCGAG GGCTTCAAGT GGGAGCGCGT
5221 GATGAACTTC GAGGACGGCG GTCTGGTGAC CGTGACCCAG GACTCCTCCC TGCAGGACGG
5281 CACGCTGATC TACAAGGTGA AGATGCGCGG CACCAACTTC CCCCCCGACG GCCCCGTAAT
5341 GCAGAAGAAG ACCATGGGCT GGGAGGCCTC CACCGAGCGC CTGTACCCCC GCGACGGCGT
5401 GCTGAAGGGC GAGATCCACC AGGCCCTGAA GCTGAAGGAC GGCGGCCACT ACCTGGTGGA
5461 GTTCAAGACC ATCTACATGG CCAAGAAGCC CGTGCAACTG CCCGGCTACT ACTACGTGGA
5521 CACCAAGCTG GACATCACCT CCCACAACGA GGACTACACC ATCGTGGAAC AGTACGAGCG
5581 CTCCGAGGGC CGCCACCACC TGTTCCTGGG GCATGGCACC GGCAGCACCG GCAGCGGCAG
5641 CTCCGGCACC GCCTCCTCCG AGGACAACAA CATGGCCGTC ATCAAAGAGT TCATGCGCTT
5701 CAAGGTGCGC ATGGAGGGCT CCATGAACGG CCACGAGTTC GAGATCGAGG GCGAGGGCGA
5761 GGGCCGCCCC TACGAGGGCA CCCAGACCGC CAAGCTGAAG GTGACCAAGG GCGGCCCCCT
5821 GCCCTTCGCC TGGGACATCC TGTCCCCCCA GTTCATGTAC GGCTCCAAGG CGTACGTGAA
```

```
5881 GCACCCCGCC GACATCCCCG ATTACAAGAA GCTGTCCTTC CCCGAGGGCT TCAAGTGGGA
5941 GCGCGTGATG AACTTCGAGG ACGGCGGTCT GGTGACCGTG ACCCAGGACT CCTCCCTGCA
6001 GGACGGCACG CTGATCTACA AGGTGAAGAT GCGCGGCACC AACTTCCCCC CCGACGGCCC
6061 CGTAATGCAG AAGAAGACCA TGGGCTGGGA GGCCTCCACC GAGCGCCTGT ACCCCCGCGA
6121 CGGCGTGCTG AAGGGCGAGA TCCACCAGGC CCTGAAGCTG AAGGACGGCG GCCACTACCT
6181 GGTGGAGTTC AAGACCATCT ACATGGCCAA GAAGCCCGTG CAACTGCCCG GCTACTACTA
6241 CGTGGACACC AAGCTGGACA TCACCTCCCA CAACGAGGAC TACACCATCG TGGAACAGTA
6301 CGAGCGCTCC GAGGGCCGCC ACCACCTGTT CCTGTACGGC ATGGACGAGC TGTACAAGTA
6361 AGAATTCCTG CAGCCCGGGG GATCCACTAG TTCTAGAGCG GCCGCCACCG CGGTGGAGCT
6421 CGAGACAACT TTGTATAATA AAGTTGGTAC CGGGCCCCCC GCTAGCGTCG ACGGTATCGA
6481 TAAGCTTGAT ATCGAATTCT CTAGATCATA ATCAGCCATA CCACATTTGT AGAGGTTTTA
6541 CTTGCTTTAA AAAACCTCCC ACACCTCCCC CTGAACCTGA AACATAAAAT GAATGCAATT
6601 GTTGTTGTTA ACTTGTTTAT TGCAGCTTAT AATGGTTACA AATAAAGCAA TAGCATCACA
6661 AATTTCACAA ATAAAGCATT TTTCTTCACT GCATTCTAGT TGTGGTTTGT CCAAACTCAT
6721 CAATGTATCA AGGGCGAATT CGGGGATCTA ATTCAATTAG AGACTAATTC AATTAGAGCT
6781 AATTCAATTA GGATCCAAGC TTATCGATTT CGAACCCTCG ACCGCCGGAG TATAAATAGA
6841 GGCGCTTCGT CTACGGAGCG ACAATTCAAT TCAAACAAGC AAAGTGAACA CGTCGCTAAG
6901 CGAAAGCTAA GCAAATAAAC AAGCGCAGCT GAACAAGCTA AACAATCGGG GTACCGCTAG
6961 AGTCGACGGT ACCGCGGGCC CGGGATCCAC CGGTCGCCAC CATGGTGAGC AAGGGCGAGG
7021 AGCTGTTCAC CGGGGTGGTG CCCATCCTGG TCGAGCTGGA CGGCGACGTA AACGGCCACA
7081 AGTTCAGCGT GTCCGGCGAG GGCGAGGGCG ATGCCACCTA CGGCAAGCTG ACCCTGAAGT
7141 TCATCTGCAC CACCGGCAAG CTGCCCGTGC CCTGGCCCAC CCTCGTGACC ACCTTCGGCT
7201 ACGGCCTGCA GTGCTTCGCC CGCTACCCCG ACCACATGAA GCAGCACGAC TTCTTCAAGT
7261 CCGCCATGCC CGAAGGCTAC GTCCAGGAGC GCACCATCTT CTTCAAGGAC GACGGCAACT
7321 ACAAGACCCG CGCCGAGGTG AAGTTCGAGG GCGACACCCT GGTGAACCGC ATCGAGCTGA
7381 AGGGCATCGA CTTCAAGGAG GACGGCAACA TCCTGGGGCA CAAGCTGGAG TACAACTACA
7441 ACAGCCACAA CGTCTATATC ATGGCCGACA AGCAGAAGAA CGGCATCAAG GTGAACTTCA
7501 AGATCCGCCA CAACATCGAG GACGGCAGCG TGCAGCTCGC CGACCACTAC CAGCAGAACA
7561 CCCCCATCGG CGACGGCCCC GTGCTGCTGC CCGACAACCA CTACCTGAGC TACCAGTCCG
7621 CCCTGAGCAA AGACCCCAAC GAGAAGCGCG ATCACATGGT CCTGCTGGAG TTCGTGACCG
7681 CCGCCGGGAT CACTCTCGGC ATGGACGAGC TGTACAAGTA AAGCGGCCGC GACTCTAGAT
7741 CATAATCAGC CATACCACAT TTGTAGAGGT TTTACTTGCT TTAAAAAACC TCCCACACCT
7801 CCCCCTGAAC CTGAAACATA AAATGAATGC AATTGTTGTT GTTAACTTGT TTATTGCAGC
7861 TTATAATGGT TACAAATAAA GCAATAGCAT CACAAATTTC ACAAATAAAG CATTTTTTTC
7921 ACTGCATTCT AGTTGTGGTT TGTCCAAACT CATCAATGTA TCTTAAAGCT TATCGATACc
7981 tcgagTACCC AGCTTTcttg tacaaagtgg ttgatCGGTA CGTACCCAAT TCGCCCTATA
8041 GTGAGTCGTA TTACAATTCA CTGGCCGTCG TTTTACAACG TCGTGACTGG GAAAACCCTG
8101 GCGTTACCCA ACTTAATCGC CTTGCAGCAC ATCCCCCTTT CGCCAGCTGG CGTAATAGCG
8161 AAGAGGCCCG CACCGATCGC CCTTCCCAAC AGTTGCGCAG CCTGAATGGC GAATGGAAAT
8221 TGTAAGCGTT AATATTTTGT TAAAATTCGC GTTAAATTTT TGTTAAATCA GCTCATTTTT
8281 TAACCAATAG GCCGAAATCG GCAAAATCCC TTATAAATCA AAAGAATAGA CCGAGATAGG
8341 GTTGAGTGTT GTTCCAGTTT GGAACAAGAG TCCACTATTA AAGAACGTGG ACTCCAACGT
8401 CAAAGGGCGA AAAACCGTCT ATCAGGGCGA TGGCCCACTA CGTGAACCAT CACCCTAATC
8461 AAGTTTTTTG GGGTCGAGGT GCCGTAAAGC ACTAAATCGG AACCCTAAAG GGAGCCCCCG
8521 ATTTAGAGCT TGACGGGGAA AGCCGGCGAA CGTGGCGAGA AAGGAAGGGA AGAAAGCGAA
8581 AGGAGCGGGC GCTAGGGCGC TGGCAAGTGT AGCGGTCACG CTGCGCGTAA CCACCACACC
8641 CGCCGCGCTT AATGCGCCGC TACAGGGCGC GTCAG
//
```
