# Supplementary material for: Tools for Anopheles gambiae Transgenesis
Source: G3 (Bethesda). 2015 Apr 13;5(6):1151–63. doi: 10.1534/g3.115.016808 (PMC4478545; doi:10.1534/g3.115.016808)
Supplement: Supporting Information [file supp_g3.115.016808_FileS4.pdf]

File S4

perl script analyzing codon usage in an input sequence to evaluate how efficiently it will be expressed in *Anopheles gambiae*. The input codon usage table derived from highly-expressed *A. gambiae* genes is appended below.

```
#!/usr/bin/perl -w


use strict;


##########################################################################
#Assessment of the codon usage status of a coding sequence

#Input : codon usage table - the threshold frequency - the heterologous coding sequence from ATG to STOP

#

#Output : results.TXT

#Vittu Ana•Øs 28-02-2013

##########################################################################



if ($#ARGV != 2){

     print " Syntaxe : codon_usage.pl input_table_codon_usage.tab    coding_sequence.fa threshold_frequency_in_%   \n" ;

     exit(-1) ;

}


###################################

##             Codon usage              ##

###################################
```

```
my %codonUsageTab = ();
my ($codon_tab, $codon_usage);
my $threshold = $ARGV[2];

if ($threshold < 1 || $threshold > 100)
{
     print "The frequency threshold must be between 1% and 
100%\nPlease, enter a new value\n";
     $threshold = <STDIN>;
}
if ($threshold >= 1 && $threshold <= 100)
{
     $threshold = $threshold / 100;
}

open(TAB, $ARGV[0]) || die ($ARGV[0]."can't be open! Exit\n");
while(<TAB>)
{
     if ($_ =~ /^[DEFGAC\*LMNHIKTWVQPSRY]/)
     {
          next;
     }else
     {
          chomp;
          ($codon_tab, $codon_usage) = split(" ", $_);
          if($codon_usage <= $threshold)
          {
               chop($codon_tab);
               $codonUsageTab{$codon_tab} = $codon_usage;
          }
```

```
     }
}
close(TAB);


###################################
##             Sequence            ##
###################################

my $sequence = "";
my $name_seq;

open(SEQ, $ARGV[1]) || die ($ARGV[1]."can't be open! Exit\n");
while(<SEQ>)
{
     if ($_ =~ /^[atcg]/)
     {
          chomp;
          $sequence .= $_;
     }
     elsif ($_ =~ /^[ATCG]/)
     {
          chomp;
          $sequence .= lc($_);
     }
     else
     {
          $name_seq = $_;
     }
}
```

```
close SEQ;



###################################
##             Count                    ##
###################################

my @seq = ();
my $nb = 0;
my $codon = "";
my %count = ();
my $m;

@seq = split (/ */, $sequence);
for ($m = 0; $m <= $#seq; $m++) {
     $codon .= $seq[$m];
     $nb += 1;
     if ($nb == 3)
     {
          if (exists $count{$codon})
          {
                    $count{$codon} += 1;
          }else
          {
               $count{$codon} = 1;
          }
          $nb = 0;
          $codon = "";
     }
```

```
}

###################################
##             Frequency           ##
###################################

my %freqcodon = ();
my $nbcodon;
my $codonFreq;

$nbcodon = length($sequence) / 3 ;
foreach my $codoncount (keys(%count))
{
     $codonFreq = $count{$codoncount} / $nbcodon;
     $freqcodon{$codoncount} = $codonFreq;
}

###################################
##             Penalty                  ##
###################################

my $penalty;
my $allpenalties = 0;
my %penalties = ();
my $proteinLength;
my $densityOfProblems;

foreach my $codoncount (keys(%count))
{
     foreach my $codontab (keys(%codonUsageTab))
```

```
     {
          if ($codoncount eq $codontab)
          {
               $penalty = $count{$codoncount} /
$codonUsageTab{$codontab};
               $penalties{$codoncount} = $penalty;
               $allpenalties += $penalty;
          }
     }
}

$proteinLength = length($sequence) / 3 ;
$densityOfProblems = $allpenalties / $proteinLength;
$allpenalties = $allpenalties / 100;
$densityOfProblems = $densityOfProblems * 10;


###################################
##            Output                  ##
###################################

$name_seq =~ s/^.//;
chomp($name_seq);

open(OUT, ">".$name_seq."_codus_results.txt") || die
($name_seq."_codus_results.txt can't be open! Exit\n");

print OUT "Protein length :\t$proteinLength\n";
print OUT "Sum of all penalties :\t";
printf OUT "%0.2f", $allpenalties;
```

```
print OUT "\nDensity of problems :\t";
printf OUT "%0.2f", $densityOfProblems;


print OUT "\nCodon\tFreq in model\tCount\tFreq in
seq\tPenalty\n";
foreach my $codonpenalty (keys(%penalties))
{
     print OUT "$codonpenalty\t$codonUsageTab{$codonpenalty}\t";
     print OUT "$count{$codonpenalty}\t";
     printf OUT "%0.2f", $freqcodon{$codonpenalty};
     print OUT "\t";
     printf OUT "%0.2f", $penalties{$codonpenalty};
     print OUT "\n";
}

close OUT;
```

Codon usage table:

```
D
gat: 0.36
gac: 0.64
E
gaa: 0.23
gag: 0.77
F
ttt: 0.16
```

ttc: 0.84

G

ggt: 0.27

ggc: 0.45

gga: 0.22

ggg: 0.07

A

gct: 0.19

gcc: 0.45

gca: 0.09

gcg: 0.28

C

tgt: 0.33

tgc: 0.67

*

taa: 0.57

tag: 0.29

tga: 0.14

L

tta: 0.01

ttg: 0.06

ctt: 0.07

ctc: 0.17

cta: 0.03

ctg: 0.66

M

atg: 1.00

N

aat: 0.14

aac: 0.86

H

cat: 0.21

cac: 0.79

I

att: 0.18

atc: 0.79

ata: 0.04

K

aaa: 0.08

aag: 0.92

T

act: 0.06

acc: 0.48

aca: 0.05

acg: 0.42

W

tgg: 1.00

V

gtt: 0.11

gtc: 0.35

gta: 0.04

gtg: 0.51

Q

caa: 0.09

cag: 0.91

P

cct: 0.05

ccc: 0.26

cca: 0.13

ccg: 0.56

S

tct: 0.08

tcc: 0.17

tca: 0.01

tcg: 0.40

agt: 0.05

agc: 0.29

R

cgt: 0.30

cgc: 0.51

cga: 0.05

cgg: 0.10

aga: 0.02

agg: 0.02

Y

tat: 0.11

tac: 0.89
