# Supplementary material for: Tools for Anopheles gambiae Transgenesis
Source: G3 (Bethesda). 2015 Apr 13;5(6):1151–63. doi: 10.1534/g3.115.016808 (PMC4478545; doi:10.1534/g3.115.016808)
Supplement: Supporting Information [file supp_g3.115.016808_FileS5.pdf]

File S5

Annotated DNA sequence of the four plasmids used to generate transgenic *A. gambiae* lines expressing codon-optimized CSP (with and without Fasciclin gpi anchor, with Lipophorin (Lp) or Vitellogenin (Vg) promoter)

<u>pAttBRfB1CFP-Vg-OptCSPΔgpi:</u>

```
LOCUS       pattB-VgP-OptCSP        7510 bp    DNA     circular
FEATURES             Location/Qualifiers
     misc_feature    complement(6869..6889)
                     /note="T7"
     misc_feature    complement(2216..2500)
                     /note="attB"
     misc_feature    2173..2192
                     /note="T3"
     misc_feature    2138..2156
                     /note="M13R"
     misc_feature    2516..2538
                     /note="attB1"
     misc_feature    4311..4385
                     /note="lipophorin signal peptide"
     misc_feature    4303..4310
                     /note="Vg Kozak"
     misc_feature    complement(4280..4299)
                     /note="attB4"
     misc_feature    5247..5476
                     /note="SV40 term"
     misc_feature    5539..5744
                     /note="3xP3-hsp70"
     misc_feature    5481..5502
                     /note="attB3"
     misc_feature    6534..6755
                     /note="SV40 term"
     misc_feature    complement(6824..6845)
                     /note="attB2"
     misc_feature    2564..4265
                     /note="Vitellogenin promoter"
     gene            5797..6513
                     /note="ECFP (Clontech)"
ORIGIN
        1 GTGGCACTTT TCGGGGAAAT GTGCGCGGAA CCCCTATTTG TTTATTTTTC TAAATACATT
       61 CAAATATGTA TCCGCTCATG AGACAATAAC CCTGATAAAT GCTTCAATAA TATTGAAAAA
      121 GGAAGAGTAT GAGTATTCAA CATTTCCGTG TCGCCCTTAT TCCCTTTTTT GCGGCATTTT
      181 GCCTTCCTGT TTTTGCTCAC CCAGAAACGC TGGTGAAAGT AAAAGATGCT GAAGATCAGT
      241 TGGGTGCACG AGTGGGTTAC ATCGAACTGG ATCTCAACAG CGGTAAGATC CTTGAGAGTT
      301 TTCGCCCCGA AGAACGTTTT CCAATGATGA GCACTTTTAA AGTTCTGCTA TGTGGCGCGG
      361 TATTATCCCG TATTGACGCC GGGCAAGAGC AACTCGGTCG CCGCATACAC TATTCTCAGA
      421 ATGACTTGGT TGAGTACTCA CCAGTCACAG AAAAGCATCT TACGGATGGC ATGACAGTAA
      481 GAGAATTATG CAGTGCTGCC ATAACCATGA GTGATAACAC TGCGGCCAAC TTACTTCTGA
      541 CAACGATCGG AGGACCGAAG GAGCTAACCG CTTTTTTGCA CAACATGGGG GNATCATGTA
      601 ACTCGCCTTN GATCGTTGGG AACCGGAGCT GAATGAAGCC ATACCAAACG ACGAGCGTGA
      661 CACCACGATG CCTGTAGCAA TGGCAACAAC GTTGCGCAAA CTATTAACTG GCGAACTACT
      721 TACTCTAGCT TCCCGGCAAC AATTAATAGA CTGGATGGAG GCGGATAAAG TTGCAGGACC
      781 ACTTCTGCGC TCGGCCCTTC CGGCTGGCTG GTTTATTGCT GATAAATCTG GAGCCGGTGA
      841 GCGTGGGTCT CGCGGTATCA TTGCAGCACT GGGGCCAGAT GGTAAGCCCT CCCGTATCGT
      901 AGTTATCTAC ACGACGGGGA GTCAGGCAAC TATGGATGAA CGAAATAGAC AGATCGCTGA
      961 GATAGGTGCC TCACTGATTA AGCATTGGTA ACTGTCAGAC CAAGTTTACT CATATATACT
     1021 TTAGATTGAT TTAAAACTTC ATTTTTAATT TAAAAGGATC TAGGTGAAGA TCCTTTTTGA
     1081 TAATCTCATG ACCAAAATCC CTTAACGTGA GTTTTCGTTC CACTGAGCGT CAGACCCCGT
     1141 AGAAAAGATC AAAGGATCTT CTTGAGATCC TTTTTTTCTG CGCGTAATCT GCTGCTTGCA
     1201 AACAAAAAAA CCACCGCTAC CAGCGGTGGT TTGTTTGCCG GATCAAGAGC TACCAACTCT
     1261 TTTTCCGAAG GTAACTGGCT TCAGCAGAGC GCAGATACCA AATACTGTCC TTCTAGTGTA
     1321 GCCGTAGTTA GGCCACCACT TCAAGAACTC TGTAGCACCG CCTACATACC TCGCTCTGCT
     1381 AATCCTGTTA CCAGTGGCTG CTGCCAGTGG CGATAAGTCG TGTCTTACCG GGTTGGACTC
     1441 AAGACGATAG TTACCGGATA AGGCGCAGCG GTCGGGCTGA ACGGGGGGTT CGTGCACACA
     1501 GCCCAGCTTG GAGCGAACGA CCTACACCGA ACTGAGATAC CTACAGCGTG AGCTATGAGA
     1561 AAGCGCCACG CTTCCCGAAG GGAGAAAGGC GGACAGGTAT CCGGTAAGCG GCAGGGTCGG
     1621 AACAGGAGAG CGCACGAGGG AGCTTCCAGG GGGAAACGCC TGGTATCTTT ATAGTCCTGT
     1681 CGGGTTTCGC CACCTCTGAC TTGAGCGTCG ATTTTTGTGA TGCTCGTCAG GGGGGCGGAG
     1741 CCTATGGAAA AACGCCAGCA ACGCGGCCTT TTTACGGTTC CTGGCCTTTT GCTGGCCTTT
     1801 TGCTCACATG TTCTTTCCTG CGTTATCCCC TGATTCTGTG GATAACCGTA TTACCGCCTT
     1861 TGAGTGAGCT GATACCGCTC GCCGCAGCCG AACGACCGAG CGCAGCGAGT CAGTGAGCGA
     1921 GGAAGCGGAA GAGCGCCCAA TACGCAAACC GCCTCTCCCC GCGCGTTGGC CGATTCATTA
     1981 ATGCAGCTGG CACGACAGGT TTCCCGACTG GAAAGCGGGC AGTGAGCGCA ACGCAATTAA
```

2041 TGTGAGTTAG CTCACTCATT AGGCACCCCA GGCTTTACAC TTTATGCTTC CGGCTCGTAT
2101 GTTGTGTGGA ATTGTGAGCG GATAACAATT TCACACAGGA AACAGCTATG ACCATGATTA
2161 CGCCAAGCTC GAAATTAACC CTCACTAAAG GGAACAAAAG CTGGCTAGAA CTAGTGTCGA
2221 CATGCCCGCC GTGACCGTCG AGAACCCGCT GACGCTGCCC CGCGTATCCG CACCCGCCGA
2281 CGCCGTCGCA CGTCCCGTGC TCACCGTGAC CACCGCGCCC AGCGGTTTCG AGGGCGAGGG
2341 CTTCCCGGTG CGCCGCGCGT TCGCCGGGAT CAACTACCGC CACCTCGACC CGTTCATCAT
2401 GATGGACCAG ATGGGTGAGG TGGAGTACGC GCCCGGGGAG CCCAAGGGCA CGCCCTGGCA
2461 CCCGCACCGC GGCTTCGAGA CCGTGACCTA CATCGTCGAC ACTAGTGgat catcaaacaa
2521 gtttGTACAA AAAAGCAGGC TGGTACCGGG CCCCCCGCTA GCGTCGAGTT CAACTCGACC
2581 ATAATAATTG ATCCGTCAAT CCATATTGGT CCGCAATAAT GAAAGTTGCA AGAGTACGAC
2641 GGTATGAAAA GAGGTTCAGT AAGTTGTAAA CTAATAGTTT CTTCCCAACG TTCAAATGCT
2701 GGCAAATCTT TTCGCGGGCC GCACTTCGTC GATCGCTAGT CTTAATGATA ATTTCTGAGA
2761 AAAAGGTGCT ACTGCATCTA CTATATTCTA CTGGATATAA ATGAAATAAC AACGTGAGAC
2821 TCACCTACAA CATGTAATTT ATTGATGGTT TAGTTTAACC AACCTATGAA ATAATTTGAT
2881 ATAGAAATTT GTAGTCGTTT TTCTATGAAG TAAAATTCTA AAATCAAACA TTAAACTGTT
2941 TTGTAGTACC CGGACTCATG GTATGGCTTC TATTAGCCGT AAACAAAGAT TTACAATTGA
3001 CTAAGGTTAG GTCCGACACT GTAGGAGCCA GCGCGTCCTT TCAATACATC AACGGACCAT
3061 CTCGTGTTGT TAAATACTTA TTATTATTAT GGTTTGCTAA TTGATATGTT CCAAGACCGA
3121 TTTGGATTTC GAAATAAGTA TTCTCTGATT CATTTTGGGA GCCGGTCTCG TGATACAGTC
3181 GTCAGCCCGT ACGACTTAAC TACATTCTCG TCATGGGTTC AAGCCCCAGA TGGACCGTGC
3241 CGCCATACGT AGAGTCAGTC CTATCCTGTT ATGGGGGGTA ATACATAAGA CACTGAAAGC
3301 CAACCCCACA AGTGGTACAG ACAAGCCTTG aCCGACAATT GTTGTTGAGC CAAACAGAAG
3361 AAGAATCCAT TTCGGGAAAT GATTTTATCA TTCAATCAAA CCAGTCAATC ATAAACATCA
3421 TAGTTTTAAA TACTCAAAAC TAGTTGAGAT CTTTAAAACA CATTATTTTA GTTTAATTAA
3481 ATGATCTGTT AGCTAGAAGG TAGATACGAT ATTTTAGACA TTTCGTAATA GATCGCAAAT
3541 CTCTATTATG TTGGTAATTC ACTTCGTAAA ACTCTTAGGC AAAACTCTTA TTAGTAAACA
3601 AAATACTAAT CAAACACTGA TAAACTAACG CGATTTATAC ATTGGACAAA GAAGAGGCTG
3661 ATTTTAAAAA TACTCGCTTT AAAATTTGCT TCATTCATCA ATGTATTGTA AAGCACATAA
3721 AGAACACAAT CATTGACTGA AAACAATTCC ACGTCTCAGC CAACTTCCAG GATCAATGAA
3781 ATAGCAAGTT CCAAGTTCCA TTTCATTGAT TATGGTAACT ACTGATTATT TTCAATAACA
3841 AATACTTCGA AGACTGCACA ATTCAAAAGT ATGCCAGAAA GAAAGGATTA CTATCAATTG
3901 TGGGTTAATC AAACTAAGAC AGGTGGCAAA AATGGAACCA TTGATTAAGG CAGCCACTGA
3961 CCGATTTCAT TTAAAAAACA CACTCTTGGA AGTTTCCACA CAATCTCACC TTTTGCCAAT
4021 TTTAGCAAAG ACGTTGTGCT GCACTGATAA GAATCGAACT GTAAACATGT GGGCAGTAAA
4081 AATTATTTCA TCGTTCAACA CGGCGGTCAT TACACTATTC GAAGCAGCTG AAAAGATTTG
4141 ATGATAGCAG GACCGTGAGA TCAGCAAATT TGAGGTATAA AAGATGATCC TGCGACCACC
4201 AGAAGGCACA TTCGAGCTTT GGAGTGCATT CAAAGCATCC GGGCAACTGC GAACAACCGA
4261 ACCatCGATC TCGAGCACCC AACTTTTCTA TACAAAGTTg gtACCGAACC ATGTGGGTGC
4321 TGGGTGGCCG TCGGCTCCTG TGGAGCTTCC TGGTGAGCCT GGTGTTGATC CAAAGCGTGT
4381 CCGCCAAGAT CATCCAGGCG CAGCGCAACT TGAATGAGCT GTGTTATAAT GAAGGAAACG
4441 ATAACAAATT GTACCATGTG TTGAACAGCA AGAATGGTAA GATCTATAAT AGAAACACGG
4501 TCAACCGACT CTTGGCTGAT GCTCCGGAAG GAAAGAAAAA CGAGAAGAAA AATGAGAAGA
4561 TCGAGCGGAA TAACAAATTG AAGCAGCCTC CTCCGCCGCC CAACCCCAAC GATCCACCTC
4621 CACCAAACCC AAACGACCCG CCACCTCCCA ATCCAAATGA TCCGCCGCCG CCGAACCCGA
4681 ACGACCCTGC GCCTCCTAAC GCAAACGATC CGGCCCCACC GAATGCCAAC GATCCAGCCC
4741 CGCCGAACGC GAACGACCCT GCACCGCCTA ATGCCAACGA TCCGGCTCCG CCGAATGCAA
4801 ATGATCCAGC TCCACCGAAT GCAAATGACC CCGCGCCGCC TAACGCGAAT GATCCGCCTC
4861 CGCCGAATCC AAACGACCCG GCACCTCCGC AGGGAAACAA CAACCCGCAG CCGCAACCGC
4921 GTCCGCAGCC ACAACCCCAG CCCCAACCGC AGCCGCAGCC GCAGCCACAA CCTCAGCCGC
4981 GTCCGCAGCC GCAGCCTCAG CCGGGAGGAA ACAACAACAA CAAGAATAAT AACAATGATG
5041 ACTCCTACAT TCCTAGCGCA GAAAAGATCC TGGAGTTTGT GAAGCAAATC CGCGACTCGA
5101 TCACTGAGGA GTGGAGCCAG TGTAACGTGA CGTGCGGTTC GGGCATCCGC GTGCGCAAGC
5161 GTAAAGGCTC GAATAAGAAG GCTGAGGACC TGACGCTGGA AGATATCGAC ACGGAAATTT
5221 GCAAGATGGA CAAATGCGAA TTCtaaTCTA GACATAATCA GCCATACCAC ATTTGTAGAG
5281 GTTTTACTTG CTTTAAAAAA CCTCCCACAC CTCCCCCTGA ACCTGAAACA TAAAATGAAT
5341 GCAATTGTTG TTGTTAACTT GTTTATTGCA GCTTATAATG GTTACAAATA AAGCAATAGC
5401 ATCACAAATT TCACAAATAA AGCATTTTTC TTCACTGCAT TCTAGTTGTG GTTTGTCCAA
5461 ACTCATCAAT GTATCTcgag ACAACTTTGT ATAATAAAGT TGGTACCGGG CCCCCCGCTA
5521 GCGtcgaGTA CCGGGCCCGG ATCTAATTCA ATTAGAGACT AATTCAATTA GAGCTAATTC
5581 AATTAGGATC CAAGCTTATC GATTTCGAAC CCTCGACCGC CGGAGTATAA ATAGAGGCGC
5641 TTCGTCTACG GAGCGACAAT TCAATTCAAA CAAGCAAAGT GAACACGTCG CTAAGCGAAA
5701 GCTAAGCAAA TAAACAAGCG CAGCTGAACA AGCTAAACAA TCGGGGTACC GCTAGAGTCG
5761 ACGGTACCGC GGGCCCGGGA TCCACCGGTC GCCACCATGG TGAGCAAGGG CGAGGAGCTG
5821 TTCACCGGGG TGGTGCCCAT CCTGGTCGAG CTGGACGGCG ACGTAAACGG CCACAgGTTC
5881 AGCGTGTCCG GCGAGGGCGA GGGCGATGCC ACCTACGGCA AGCTGACCCT GAAGTTCATC
5941 TGCACCACCG GCAAGCTGCC CGTGCCCTGG CCCACCCTCG TGACCACCCT GACCTGGGGC
6001 GTGCAGTGCT TCAGCCGCTA CCCCGACCAC ATGAAGCAGC ACGACTTCTT CAAGTCCGCC
6061 ATGCCCGAAG GCTACGTCCA GGAGCGtACC ATCTTCTTCA AGGACGACGG CAACTACAAG
6121 ACCCGCGCCG AGGTGAAGTT CGAGGGCGAC ACCCTGGTGA ACCGCATCGA GCTGAAGGGC
6181 ATCGACTTCA AGGAGGACGG CAACATCCTG GGGCACAAGC TGGAGTACAA CTACATCAGC
6241 CACAACGTCT ATATCACCGC CGACAAGCAG AAGAACGGCA TCAAGGCCcA CTTCAAGATC
6301 CGCCACAACA TCGAGGACGG CAGCGTGCAG CTCGCCGACC ACTACCAGCA GAACACCCCC
6361 ATCGGCGACG GCCCCGTGCT GCTGCCCGAC AACCACTACC TGAGCACCCA GTCCGCCCTG
6421 AGCAAAGACC CCAACGAGAA GCGCGATCAC ATGGTCCTGC TGGAGTTCGT GACCGCCGCC
6481 GGGATCACTC TCGGCATGGA CGAGCTGTAC AAGTAAAGCG GCCGCGACTC TAGATCATAA
6541 TCAGCCATAC CACATTTGTA GAGGTTTTAC TTGCTTTAAA AAACCTCCCA CACCTCCCCC
6601 TGAACCTGAA ACATAAAATG AATGCAATTG TTGTTGTTAA CTTGTTTATT GCAGCTTATA
6661 ATGGTTACAA ATAAAGCAAT AGCATCACAA ATTTCACAAA TAAAGCATTT TTTCACTGC
6721 ATTCTAGTTG TGGTTTGTCC AAACTCATCA ATGTATCTTA AAGCTTATCG ATACGCGTAC
6781 GGCGCGCCTA GAGCGGCCGC CACCGCGGTG GAGCTCGAGT ACCCAGCTTT cttgtacaaa
6841 gtggtttgat CGGTACGTAC CCAATTCGCC CTATAGTGAG TCGTATTACA ATTCACTGGC

```
     6901 CGTCGTTTTA CAACGTCGTG ACTGGGAAAA CCCTGGCGTT ACCCAACTTA ATCGCCTTGC
     6961 AGCACATCCC CCTTTCGCCA GCTGGCGTAA TAGCGAAGAG GCCCGCACCG ATCGCCCTTC
     7021 CCAACAGTTG CGCAGCCTGA ATGGCGAATG GAAATTGTAA GCGTTAATAT TTTGTTAAAA
     7081 TTCGCGTTAA ATTTTTGTTA AATCAGCTCA TTTTTTAACC AATAGGCCGA AATCGGCAAA
     7141 ATCCCTTATA AATCAAAAGA ATAGACCGAG ATAGGGTTGA GTGTTGTTCC AGTTTGGAAC
     7201 AAGAGTCCAC TATTAAAGAA CGTGGACTCC AACGTCAAAG GGCGAAAAAC CGTCTATCAG
     7261 GGCGATGGCC CACTACGTGA ACCATCACCC TAATCAAGTT TTTTGGGGTC GAGGTGCCGT
     7321 AAAGCACTAA ATCGGAACCC TAAAGGGAGC CCCCGATTTA GAGCTTGACG GGGAAAGCCG
     7381 GCGAACGTGG CGAGAAAGGA AGGGAAGAAA GCGAAAGGAG CGGGCGCTAG GGCGCTGGCA
     7441 AGTGTAGCGG TCACGCTGCG CGTAACCACC ACACCCGCCG CGCTTAATGC GCCGCTACAG
     7501 GGCGCGTCAG
//
```

pAttBRfB1GFP-Lp-OptCSP:

```
LOCUS       pattB-LpP-OptCSP        7496 bp    DNA     linear       18-NOV-2014
FEATURES             Location/Qualifiers
     misc_feature    complement(6855..6875)
                     /note="T7"
     misc_feature    complement(2216..2500)
                     /note="attB"
     misc_feature    2173..2192
                     /note="T3"
     misc_feature    2138..2156
                     /note="M13R"
     promoter        2525..4220
                     /source="Lp promoter"
     misc_feature    2516..2538
                     /note="attB1"
     misc_feature    4245..4319
                     /note="lipophorin signal peptide"
     misc_feature    4237..4244
                     /note="Vg Kozak"
     clone           4221..5533
                     /source="pENTR R4-optCSP-R3"
                     /type="Entry Clone"
     misc_feature    complement(4214..4233)
                     /note="attB4"
     misc_feature    5175..5279
                     /note="AgFas1 gpi anchoring signal"
     misc_feature    5202..5204
                     /note="gpi-modified serine"
     misc_feature    5292..5521
                     /note="SV40 term"
     misc_feature    5584..5789
                     /note="3xP3-hsp70"
     misc_feature    5809..6528
                     /note="GFP"
     misc_feature    5526..5547
                     /note="attB3"
     misc_feature    6546..6767
                     /note="SV40 term"
     misc_feature    complement(6810..6831)
                     /note="attB2"
ORIGIN
        1 GTGGCACTTT TCGGGGAAAT GTGCGCGGAA CCCCTATTTG TTTATTTTTC TAAATACATT
       61 CAAATATGTA TCCGCTCATG AGACAATAAC CCTGATAAAT GCTTCAATAA TATTGAAAAA
      121 GGAAGAGTAT GAGTATTCAA CATTTCCGTG TCGCCCTTAT TCCCTTTTTT GCGGCATTTT
      181 GCCTTCCTGT TTTTGCTCAC CCAGAAACGC TGGTGAAAGT AAAAGATGCT GAAGATCAGT
      241 TGGGTGCACG AGTGGGTTAC ATCGAACTGG ATCTCAACAG CGGTAAGATC CTTGAGAGTT
      301 TTCGCCCCGA AGAACGTTTT CCAATGATGA GCACTTTTAA AGTTCTGCTA TGTGGCGCGG
      361 TATTATCCCG TATTGACGCC GGGCAAGAGC AACTCGGTCG CCGCATACAC TATTCTCAGA
      421 ATGACTTGGT TGAGTACTCA CCAGTCACAG AAAAGCATCT TACGGATGGC ATGACAGTAA
      481 GAGAATTATG CAGTGCTGCC ATAACCATGA GTGATAACAC TGCGGCCAAC TTACTTCTGA
      541 CAACGATCGG AGGACCGAAG GAGCTAACCG CTTTTTTGCA CAACATGGGG GNATCATGTA
      601 ACTCGCCTTN GATCGTTGGG AACCGGAGCT GAATGAAGCC ATACCAAACG ACGAGCGTGA
      661 CACCACGATG CCTGTAGCAA TGGCAACAAC GTTGCGCAAA CTATTAACTG GCGAACTACT
      721 TACTCTAGCT TCCCGGCAAC AATTAATAGA CTGGATGGAG GCGGATAAAG TTGCAGGACC
      781 ACTTCTGCGC TCGGCCCTTC CGGCTGGCTG GTTTATTGCT GATAAATCTG GAGCCGGTGA
      841 GCGTGGGTCT CGCGGTATCA TTGCAGCACT GGGGCCAGAT GGTAAGCCCT CCCGTATCGT
      901 AGTTATCTAC ACGACGGGGA GTCAGGCAAC TATGGATGAA CGAAATAGAC AGATCGCTGA
      961 GATAGGTGCC TCACTGATTA AGCATTGGTA ACTGTCAGAC CAAGTTTACT CATATATACT
     1021 TTAGATTGAT TTAAAACTTC ATTTTTAATT TAAAAGGATC TAGGTGAAGA TCCTTTTTGA
     1081 TAATCTCATG ACCAAAATCC CTTAACGTGA GTTTTCGTTC CACTGAGCGT CAGACCCCGT
     1141 AGAAAAGATC AAAGGATCTT CTTGAGATCC TTTTTTTCTG CGCGTAATCT GCTGCTTGCA
     1201 AACAAAAAAA CCACCGCTAC CAGCGGTGGT TTGTTTGCCG GATCAAGAGC TACCAACTCT
     1261 TTTTCCGAAG GTAACTGGCT TCAGCAGAGC GCAGATACCA AATACTGTCC TTCTAGTGTA
```

```
1321 GCCGTAGTTA GGCCACCACT TCAAGAACTC TGTAGCACCG CCTACATACC TCGCTCTGCT
1381 AATCCTGTTA CCAGTGGCTG CTGCCAGTGG CGATAAGTCG TGTCTTACCG GGTTGGACTC
1441 AAGACGATAG TTACCGGATA AGGCGCAGCG GTCGGGCTGA ACGGGGGGTT CGTGCACACA
1501 GCCCAGCTTG GAGCGAACGA CCTACACCGA ACTGAGATAC CTACAGCGTG AGCTATGAGA
1561 AAGCGCCACG CTTCCCGAAG GGAGAAAGGC GGACAGGTAT CCGGTAAGCG GCAGGGTCGG
1621 AACAGGAGAG CGCACGAGGG AGCTTCCAGG GGGAAACGCC TGGTATCTTT ATAGTCCTGT
1681 CGGGTTTCGC CACCTCTGAC TTGAGCGTCG ATTTTTGTGA TGCTCGTCAG GGGGGCGGAG
1741 CCTATGGAAA AACGCCAGCA ACGCGGCCTT TTTACGGTTC CTGGCCTTTT GCTGGCCTTT
1801 TGCTCACATG TTCTTTCCTG CGTTATCCCC TGATTCTGTG GATAACCGTA TTACCGCCTT
1861 TGAGTGAGCT GATACCGCTC GCCGCAGCCG AACGACCGAG CGCAGCGAGT CAGTGAGCGA
1921 GGAAGCGGAA GAGCGCCCAA TACGCAAACC GCCTCTCCCC GCGCGTTGGC CGATTCATTA
1981 ATGCAGCTGG CACGACAGGT TTCCCGACTG GAAAGCGGGC AGTGAGCGCA ACGCAATTAA
2041 TGTGAGTTAG CTCACTCATT AGGCACCCCA GGCTTTACAC TTTATGCTTC CGGCTCGTAT
2101 GTTGTGTGGA ATTGTGAGCG GATAACAATT TCACACAGGA AACAGCTATG ACCATGATTA
2161 CGCCAAGCTC GAAATTAACC CTCACTAAAG GGAACAAAAG CTGGCTAGAA CTAGTGTCGA
2221 CATGCCCGCC GTGACCGTCG AGAACCCGCT GACGCTGCCC CGCGTATCCG CACCCGCCGA
2281 CGCCGTCGCA CGTCCCGTGC TCACCGTGAC CACCGCGCCC AGCGGTTTCG AGGGCGAGGG
2341 CTTCCCGGTG CGCCGCGCGT TCGCCGGGAT CAACTACCGC CACCTCGACC CGTTCATCAT
2401 GATGGACCAG ATGGGTGAGG TGGAGTACGC GCCCGGGGAG CCCAAGGGCA CGCCCTGGCA
2461 CCCGCACCGC GGCTTCGAGA CCGTGACCTA CATCGTCGAC ACTAGTGgat catcaaacaa
2521 gtttGTACAA AAAAGCAGGC TGGTACCGGG CCCCCCGCTA GCGTCGACGG TATCGATAAg
2581 cTTGCGGGGA AGACACATTC GAGATACGCT AAGTGATTGA GCGATTACGA TCTAGCAAAA
2641 CATACGTTCA GCTGTGAGAA TAATCATCCA TCTTCCTGCA ATGAGCAGTT CATTCCCGAT
2701 TGAGGGATTT TATTCCCCGG GGGCCTTTTC AAACGGCTTA ATATAAGCAA TTAATAGTAT
2761 TTTTTCTTTC AGGTTAGTTT ACTGTAATGG TGTAATTGTC ATCTTACACC TCCGTCTGAT
2821 AAGAGATTAC GAAGCTCAGT ATGATGAAAT AAATAAGATA AATTTATTTA AAAAAGAACA
2881 ATTGCTATGA GAGTGAAATA CAACAGTGGC GTTCACAATA TTCGAAAAAC AATAAAATTA
2941 AAAAAAAAAC AAGAAAACAT TCACAAACAT ATCAATCTGC TTTCATCGAC ACCGAACTGC
3001 TAGCCTCCCC AGTCTAACCG CGGTGGGGAC GTTTAATTGC CTTTGTTCTC GCACCCGGTC
3061 AAACATACAC TTCGGACCTT GCTCCGAACC CCACTGTGAT CCCTAGCTCG TCATCATCAT
3121 TGCCGGCATC ATGCTAAGCG TGCATTATTT TCACAACTTA GCGTAATGCT AGCGTGCGCT
3181 AGCAACAAAC TCGCCCGCAG ACTCGTCACA GCACCGGTAC GATCGATCGT TTACCGTTCC
3241 CTTTCCCGAT CGGGTTGGCT GCGATATCCG TGTCCGGGTA GAAAACTTCC CCTTTTACAC
3301 ACACACACTC ACATACACAC AGAGCTGAAT AGCAACTTAC CTTATCTGTT CGTCATCGCT
3361 CGGGCGGATC TGGACGAATC TTCGCACCGA TAACCATGTG GATCTACGAC CTCCGCTTGG
3421 CTGTCTCTCT GCTCATGTGT ATGTCTGTGT GTGTGTGTAT GTGAGCTTCT TCCCTCAAAT
3481 CCCTCGATCT CGCTGTGSCA ACAATCAAAC GTGCAAGTGC AAACATTGCA CCCCATTGAT
3541 TATACACCCA ACACCAACCA ATTCCCCTTG CGGAGGCATC TCTGTGCTCG GCAGCATGTT
3601 TACCGCAGAT CTACAGAGAA CTTCAATTGA GGTCCTTTCC ACCCCCAGCC CTCAACCGGC
3661 AATCCGGCAG CCACTGGATC ATCAGCGAAA GAGAGAGAGA GCAGAGCAGA ACAGAGGTGA
3721 CCAACTGTGG TATCGCTTCC CGCGCGCCGG TGTGTTGGTG TCCATTTCGG TGATCGCGAT
3781 CCCGGCCGCT TCCAGCACCG TCCACCGATC AGTCACAAAA ACGCTCTCCA AACCCCTTAT
3841 CAGCACCGTT CGCTGATGTG AACCCCCGTT CAAACCCCAA ATGCAGTGTT TGTATTGCTG
3901 TGTGTATGTG TACGTGCGTG TGTGTGGAAA TTCTATAAAA GTAGGCACCC GTGGCCGGGA
3961 TCCGTTATTC CCGTCCTGAG GCCCGCCCGG GATCGCTGGT GACGACAGAC GAGCCGCTGT
4021 GTGACGTACG TAGTGCCCGA TCGGTAAAGA GTGAACCGTC TTCTCTGCAG TGTAGGAGAG
4081 AACGGTTTCA TCTTTTTCGC CCACACCCCC CCGGTTACAT TCCATGTTGA ACTGTAAGGT
4141 CTAGTGAACA TTTCCTGAGT GTGGAAAGTG TGGTTTAGTG CGTGAGAGTG CACGGACACG
4201 ACACTCGAGC ACCCAACTTT TCTATACAAA GTTggtACCG AACCATGTGG GTGCTGGGTG
4261 GCCGTCGGCT CCTGTGGAGC TTCCTGGTGA GCCTGGTGTT GATCCAAAGC GTGTCCGCCA
4321 AGATCATCCA GGCGCAGCGC AACTTGAATG AGCTGTGTTA TAATGAAGGA AACGATAACA
4381 AATTGTACCA TGTGTTGAAC AGCAAGAATG GTAAGATCTA TAATAGAAAC ACGGTCAACC
4441 GACTCTTGGC TGATGCTCCG GAAGGAAAGA AAAACGAGAA GAAAAATGAG AAGATCGAGC
4501 GGAATAACAA ATTGAAGCAG CCTCCTCCGC CGCCCAACCC CAACGATCCA CCTCCACCAA
4561 ACCCAAACGA CCCGCCACCT CCCAATCCAA ATGATCCGCC GCCGCCGAAC CCGAACGACC
4621 CTGCGCCTCC TAACGCAAAC GATCCGGCCC CACCGAATGC CAACGATCCA GCCCCGCCGA
4681 ACGCGAACGA CCCTGCACCG CCTAATGCCA ACGATCCGGC TCCGCCGAAT GCAAATGATC
4741 CAGCTCCACC GAATGCAAAT GACCCCGCGC CGCCTAACGC GAATGATCCG CCTCCGCCGA
4801 ATCCAAACGA CCCGGCACCT CCGCAGGGAA ACAACAACCC GCAGCCGCAA CCGCGTCCGC
4861 AGCCACAACC CCAGCCCCAA CCGCAGCCGC AGCCGCAGCC ACAACCTCAG CCGCGTCCGC
4921 AGCCGCAGCC TCAGCCGGGA GGAAACAACA ACAACAAGAA TAATAACAAT GATGACTCCT
4981 ACATTCCTAG CGCAGAAAAG ATCCTGGAGT TTGTGAAGCA AATCCGCGAC TCGATCACTG
5041 AGGAGTGGAG CCAGTGTAAC GTGACGTGCG GTTCGGGCAT CCGCGTGCGC AAGCGTAAAG
5101 GCTCGAATAA GAAGGCTGAG GACCTGACGC TGGAAGATAT CGACACGGAA ATTTGCAAGA
5161 TGGACAAATG CGAATTCGTT CACGAGTACG ACATGATTAC GTCGGACGGC CATCACCTGT
5221 ACGGTATCGA ACCGTTGACC CTGCTGCTGG TGACCGTTCT CACCGCACTG GGAGCAGTTt
5281 aaGAATTCta aTCTAGACAT AATCAGCCAT ACCACATTTG TAGAGGTTTT ACTTGCTTTA
5341 AAAAACCTCC CACACCTCCC CCTGAACCTG AAACATAAAA TGAATGCAAT TGTTGTTGTT
5401 AACTTGTTTA TTGCAGCTTA TAATGGTTAC AAATAAAGCA ATAGCATCAC AAATTTCACA
5461 AATAAAGCAT TTTTCTTCAC TGCATTCTAG TTGTGGTTTG TCCAAACTCA TCAATGTATC
5521 TcgagACAAC TTTGTATAAT AAAGTTGGTA CCGGGCCCCC CGCTAGCGtc gaGTACCGGG
5581 CCCGGATCTA ATTCAATTAG AGACTAATTC AATTAGAGCT AATTCAATTA GGATCCAAGC
5641 TTATCGATTT CGAACCCTCG ACCGCCGGAG TATAAATAGA GGCGCTTCGT CTACGGAGCG
5701 ACAATTCAAT TCAAACAAGC AAAGTGAACA CGTCGCTAAG CGAAAGCTAA GCAAATAAAC
5761 AAGCGCAGCT GAACAAGCTA AACAATCGGC TCGAGACCGG TCGCCACCAT GGTGAGCAAG
5821 GGCGAGGAGC TGTTCACCGG GGTGGTGCCC ATCCTGGTCG AGCTGGACGG CGACGTAAAC
5881 GGCCACAAGT TCAGCGTGTC CGGCGAGGGC GAGGGCGATG CCACCTACGG CAAGCTGACC
5941 CTGAAGTTCA TCTGCACCAC CGGCAAGCTG CCCGTGCCCT GGCCCACCCT CGTGACCACC
6001 CTGACCTACG GCGTGCAGTG CTTCAGCCGC TACCCCGACC ACATGAAGCA GCACGACTTC
6061 TTCAAGTCCG CCATGCCCGA AGGCTACGTC CAGGAGCGCA CCATCTTCTT CAAGGACGAC
6121 GGCAACTACA AGACCCGCGC CGAGGTGAAG TTCGAGGGCG ACACCCTGGT GAACCGCATC
```

```
     6181 GAGCTGAAGG GCATCGACTT CAAGGAGGAC GGCAACATCC TGGGGCACAA GCTGGAGTAC
     6241 AACTACAACA GCCACAACGT CTATATCATG GCCGACAAGC AGAAGAACGG CATCAAGGTG
     6301 AACTTCAAGA TCCGCCACAA CATCGAGGAC GGCAGCGTGC AGCTCGCCGA CCACTACCAG
     6361 CAGAACACCC CCATCGGCGA CGGCCCCGTG CTGCTGCCCG ACAACCACTA CCTGAGCACC
     6421 CAGTCCGCCC TGAGCAAAGA CCCCAACGAG AAGCGCGATC ACATGGTCCT GCTGGAGTTC
     6481 GTGACCGCCG CCGGGATCAC TCTCGGCATG GACGAGCTGT ACAAGTAAAG CGGCCGCGAC
     6541 TCTAGATCAT AATCAGCCAT ACCACATTTG TAGAGGTTTT ACTTGCTTTA AAAAACCTCC
     6601 CACACCTCCC CCTGAACCTG AAACATAAAA TGAATGCAAT TGTTGTTGTT AACTTGTTTA
     6661 TTGCAGCTTA TAATGGTTAC AAATAAAGCA ATAGCATCAC AAATTTCACA AATAAAGCAT
     6721 TTTTTTCACT GCATTCTAGT TGTGGTTTGT CCAAACTCAT CAATGTATCT TAGCTAGAGC
     6781 GGCCGCCACC GCGGTGGAGC TCGAGTACCC AGCTTTcttg tacaaagtgg tttgatCGGT
     6841 ACGTACCCAA TTCGCCCTAT AGTGAGTCGT ATTACAATTC ACTGGCCGTC GTTTTACAAC
     6901 GTCGTGACTG GGAAAACCCT GGCGTTACCC AACTTAATCG CCTTGCAGCA CATCCCCCTT
     6961 TCGCCAGCTG GCGTAATAGC GAAGAGGCCC GCACCGATCG CCCTTCCCAA CAGTTGCGCA
     7021 GCCTGAATGG CGAATGGAAA TTGTAAGCGT TAATATTTTG TTAAAATTCG CGTTAAATTT
     7081 TTGTTAAATC AGCTCATTTT TTAACCAATA GGCCGAAATC GGCAAAATCC CTTATAAATC
     7141 AAAAGAATAG ACCGAGATAG GGTTGAGTGT TGTTCCAGTT TGGAACAAGA GTCCACTATT
     7201 AAAGAACGTG GACTCCAACG TCAAAGGGCG AAAAACCGTC TATCAGGGCG ATGGCCCACT
     7261 ACGTGAACCA TCACCCTAAT CAAGTTTTTT GGGGTCGAGG TGCCGTAAAG CACTAAATCG
     7321 GAACCCTAAA GGGAGCCCCC GATTTAGAGC TTGACGGGGA AAGCCGGCGA ACGTGGCGAG
     7381 AAAGGAAGGG AAGAAAGCGA AAGGAGCGGG CGCTAGGGCG CTGGCAAGTG TAGCGGTCAC
     7441 GCTGCGCGTA ACCACCACAC CCGCCGCGCT TAATGCGCCG CTACAGGGCG CGTCAG
//
```

pAttBRfB1YFP-Vg-OptCSP:

```
LOCUS       pattB-VgP-OptCSP        7562 bp    DNA     circular
FEATURES             Location/Qualifiers
     misc_feature    complement(6921..6941)
                     /note="T7"
     misc_feature    complement(2216..2500)
                     /note="attB"
     misc_feature    2173..2192
                     /note="T3"
     misc_feature    2138..2156
                     /note="M13R"
     misc_feature    2516..2538
                     /note="attB1"
     misc_feature    4311..4385
                     /note="lipophorin signal peptide"
     misc_feature    4303..4310
                     /note="Vg Kozak"
     clone           4287..5599
                     /source="pENTR R4-optCSP-R3"
                     /type="Entry Clone"
     misc_feature    complement(4280..4299)
                     /note="attB4"
     misc_feature    5241..5345
                     /note="AgFas1 gpi anchoring signal"
     misc_feature    5268..5270
                     /note="gpi-modified serine"
     misc_feature    5358..5587
                     /note="SV40 term"
     misc_feature    5650..5855
                     /note="3xP3-hsp70"
     misc_feature    5592..5613
                     /note="attB3"
     misc_feature    6612..6833
                     /note="SV40 term"
     misc_feature    complement(6876..6897)
                     /note="attB2"
     misc_feature    2516..2524
                     /note="RfB"
     misc_feature    2564..4265
                     /note="Vitellogenin promoter"
     gene            5875..6591
                     /note="EYFP (Clontech)"

ORIGIN
        1 GTGGCACTTT TCGGGGAAAT GTGCGCGGAA CCCCTATTTG TTTATTTTTC TAAATACATT
       61 CAAATATGTA TCCGCTCATG AGACAATAAC CCTGATAAAT GCTTCAATAA TATTGAAAAA
      121 GGAAGAGTAT GAGTATTCAA CATTTCCGTG TCGCCCTTAT TCCCTTTTTT GCGGCATTTT
      181 GCCTTCCTGT TTTTGCTCAC CCAGAAACGC TGGTGAAAGT AAAAGATGCT GAAGATCAGT
      241 TGGGTGCACG AGTGGGTTAC ATCGAACTGG ATCTCAACAG CGGTAAGATC CTTGAGAGTT
      301 TTCGCCCCGA AGAACGTTTT CCAATGATGA GCACTTTTAA AGTTCTGCTA TGTGGCGCGG
      361 TATTATCCCG TATTGACGCC GGGCAAGAGC AACTCGGTCG CCGCATACAC TATTCTCAGA
      421 ATGACTTGGT TGAGTACTCA CCAGTCACAG AAAAGCATCT TACGGATGGC ATGACAGTAA
```

```
 481 GAGAATTATG CAGTGCTGCC ATAACCATGA GTGATAACAC TGCGGCCAAC TTACTTCTGA
 541 CAACGATCGG AGGACCGAAG GAGCTAACCG CTTTTTTGCA CAACATGGGG GNATCATGTA
 601 ACTCGCCTTN GATCGTTGGG AACCGGAGCT GAATGAAGCC ATACCAAACG ACGAGCGTGA
 661 CACCACGATG CCTGTAGCAA TGGCAACAAC GTTGCGCAAA CTATTAACTG GCGAACTACT
 721 TACTCTAGCT TCCCGGCAAC AATTAATAGA CTGGATGGAG GCGGATAAAG TTGCAGGACC
 781 ACTTCTGCGC TCGGCCCTTC CGGCTGGCTG GTTTATTGCT GATAAATCTG GAGCCGGTGA
 841 GCGTGGGTCT CGCGGTATCA TTGCAGCACT GGGGCCAGAT GGTAAGCCCT CCCGTATCGT
 901 AGTTATCTAC ACGACGGGGA GTCAGGCAAC TATGGATGAA CGAAATAGAC AGATCGCTGA
 961 GATAGGTGCC TCACTGATTA AGCATTGGTA ACTGTCAGAC CAAGTTTACT CATATATACT
1021 TTAGATTGAT TTAAAACTTC ATTTTTAATT TAAAAGGATC TAGGTGAAGA TCCTTTTTGA
1081 TAATCTCATG ACCAAAATCC CTTAACGTGA GTTTTCGTTC CACTGAGCGT CAGACCCCGT
1141 AGAAAAGATC AAAGGATCTT CTTGAGATCC TTTTTTTCTG CGCGTAATCT GCTGCTTGCA
1201 AACAAAAAAA CCACCGCTAC CAGCGGTGGT TTGTTTGCCG GATCAAGAGC TACCAACTCT
1261 TTTTCCGAAG GTAACTGGCT TCAGCAGAGC GCAGATACCA AATACTGTCC TTCTAGTGTA
1321 GCCGTAGTTA GGCCACCACT TCAAGAACTC TGTAGCACCG CCTACATACC TCGCTCTGCT
1381 AATCCTGTTA CCAGTGGCTG CTGCCAGTGG CGATAAGTCG TGTCTTACCG GGTTGGACTC
1441 AAGACGATAG TTACCGGATA AGGCGCAGCG GTCGGGCTGA ACGGGGGGTT CGTGCACACA
1501 GCCCAGCTTG GAGCGAACGA CCTACACCGA ACTGAGATAC CTACAGCGTG AGCTATGAGA
1561 AAGCGCCACG CTTCCCGAAG GGAGAAAGGC GGACAGGTAT CCGGTAAGCG GCAGGGTCGG
1621 AACAGGAGAG CGCACGAGGG AGCTTCCAGG GGGAAACGCC TGGTATCTTT ATAGTCCTGT
1681 CGGGTTTCGC CACCTCTGAC TTGAGCGTCG ATTTTTGTGA TGCTCGTCAG GGGGGCGGAG
1741 CCTATGGAAA AACGCCAGCA ACGCGGCCTT TTTACGGTTC CTGGCCTTTT GCTGGCCTTT
1801 TGCTCACATG TTCTTTCCTG CGTTATCCCC TGATTCTGTG GATAACCGTA TTACCGCCTT
1861 TGAGTGAGCT GATACCGCTC GCCGCAGCCG AACGACCGAG CGCAGCGAGT CAGTGAGCGA
1921 GGAAGCGGAA GAGCGCCCAA TACGCAAACC GCCTCTCCCC GCGCGTTGGC CGATTCATTA
1981 ATGCAGCTGG CACGACAGGT TTCCCGACTG GAAAGCGGGC AGTGAGCGCA ACGCAATTAA
2041 TGTGAGTTAG CTCACTCATT AGGCACCCCA GGCTTTACAC TTTATGCTTC CGGCTCGTAT
2101 GTTGTGTGGA ATTGTGAGCG GATAACAATT TCACACAGGA AACAGCTATG ACCATGATTA
2161 CGCCAAGCTC GAAATTAACC CTCACTAAAG GGAACAAAAG CTGGCTAGAA CTAGTGTCGA
2221 CATGCCCGCC GTGACCGTCG AGAACCCGCT GACGCTGCCC CGCGTATCCG CACCCGCCGA
2281 CGCCGTCGCA CGTCCCGTGC TCACCGTGAC CACCGCGCCC AGCGGTTTCG AGGGCGAGGG
2341 CTTCCCGGTG CGCCGCGCGT TCGCCGGGAT CAACTACCGC CACCTCGACC CGTTCATCAT
2401 GATGGACCAG ATGGGTGAGG TGGAGTACGC GCCCGGGGAG CCCAAGGGCA CGCCCTGGCA
2461 CCCGCACCGC GGCTTCGAGA CCGTGACCTA CATCGTCGAC ACTAGTGgat catcaaacaa
2521 gtttGTACAA AAAAGCAGGC TGGTACCGGG CCCCCCGCTA GCGTCGAGTT CAACTCGACC
2581 ATAATAATTG ATCCGTCAAT CCATATTGGT CCGCAATAAT GAAAGTTGCA AGAGTACGAC
2641 GGTATGAAAA GAGGTTCAGT AAGTTGTAAA CTAATAGTTT CTTCCCAACG TTCAAATGCT
2701 GGCAAATCTT TTCGCGGGCC GCACTTCGTC GATCGCTAGT CTTAATGATA ATTTCTGAGA
2761 AAAAGGTGCT ACTGCATCTA CTATATTCTA CTGGATATAA ATGAAATAAC AACGTGAGAC
2821 TCACCTACAA CATGTAATTT ATTGATGGTT TAGTTTAACC AACCTATGAA ATAATTTGAT
2881 ATAGAAATTT GTAGTCGTTT TTCTATGAAG TAAAATTCTA AAATCAAACA TTAAACTGTT
2941 TTGTAGTACC CGGACTCATG GTATGGCTTC TATTAGCCGT AAACAAAGAT TTACAATTGA
3001 CTAAGGTTAG GTCCGACACT GTAGGAGCCA GCGCGTCCTT TCAATACATC AACGGACCAT
3061 CTCGTGTTGT TAAATACTTA TTATTATTAT GGTTTGCTAA TTGATATGTT CCAAGACCGA
3121 TTTGGATTTC GAAATAAGTA TTCTCTGATT CATTTTGGGA GCCGGTCTCG TGATACAGTC
3181 GTCAGCCCGT ACGACTTAAC TACATTCTCG TCATGGGTTC AAGCCCCAGA TGGACCGTGC
3241 CGCCATACGT AGAGTCAGTC CTATCCTGTT ATGGGGGGTA ATACATAAGA CACTGAAAGC
3301 CAACCCCACA AGTGGTACAG ACAAGCCTTG aCCGACAATT GTTGTTGAGC CAAACAGAAG
3361 AAGAATCCAT TTCGGGAAAT GATTTTATCA TTCAATCAAA CCAGTCAATC ATAAACATCA
3421 TAGTTTTAAA TACTCAAAAC TAGTTGAGAT CTTTAAAACA CATTATTTTA GTTTAATTAA
3481 ATGATCTGTT AGCTAGAAGG TAGATACGAT ATTTTAGACA TTTCGTAATA GATCGCAAAT
3541 CTCTATTATG TTGGTAATTC ACTTCGTAAA ACTCTTAGGC AAAACTCTTA TTAGTAAACA
3601 AAATACTAAT CAAACACTGA TAAACTAACG CGATTTATAC ATTGGACAAA GAAGAGGCTG
3661 ATTTTAAAAA TACTCGCTTT AAAATTTGCT TCATTCATCA ATGTATTGTA AAGCACATAA
3721 AGAACACAAT CATTGACTGA AAACAATTCC ACGTCTCAGC CAACTTCCAG GATCAATGAA
3781 ATAGCAAGTT CCAAGTTCCA TTTCATTGAT TATGGTAACT ACTGATTATT TTCAATAACA
3841 AATACTTCGA AGACTGCACA ATTCAAAAGT ATGCCAGAAA GAAAGGATTA CTATCAATTG
3901 TGGGTTAATC AAACTAAGAC AGGTGGCAAA AATGGAACCA TTGATTAAGG CAGCCACTGA
3961 CCGATTTCAT TTAAAAAACA CACTCTTGGA AGTTTCCACA CAATCTCACC TTTTGCCAAT
4021 TTTAGCAAAG ACGTTGTGCT GCACTGATAA GAATCGAACT GTAAACATGT GGGCAGTAAA
4081 AATTATTTCA TCGTTCAACA CGGCGGTCAT TACACTATTC GAAGCAGCTG AAAAGATTTG
4141 ATGATAGCAG GACCGTGAGA TCAGCAAATT TGAGGTATAA AAGATGATCC TGCGACCACC
4201 AGAAGGCACA TTCGAGCTTT GGAGTGCATT CAAAGCATCC GGGCAACTGC GAACAACCGA
4261 ACCatCGATC TCGAGCACCC AACTTTTCTA TACAAAGTTg gtACCGAACC ATGTGGGTGC
4321 TGGGTGGCCG TCGGCTCCTG TGGAGCTTCC TGGTGAGCCT GGTGTTGATC CAAAGCGTGT
4381 CCGCCAAGAT CATCCAGGCG CAGCGCAACT TGAATGAGCT GTGTTATAAT GAAGGAAACG
4441 ATAACAAATT GTACCATGTG TTGAACAGCA AGAATGGTAA GATCTATAAT AGAAACACGG
4501 TCAACCGACT CTTGGCTGAT GCTCCGGAAG GAAAGAAAAA CGAGAAGAAA AATGAGAAGA
4561 TCGAGCGGAA TAACAAATTG AAGCAGCCTC CTCCGCCGCC CAACCCCAAC GATCCACCTC
4621 CACCAAACCC AAACGACCCG CCACCTCCCA ATCCAAATGA TCCGCCGCCG CCGAACCCGA
4681 ACGACCCTGC GCCTCCTAAC GCAAACGATC CGGCCCCACC GAATGCCAAC GATCCAGCCC
4741 CGCCGAACGC GAACGACCCT GCACCGCCTA ATGCCAACGA TCCGGCTCCG CCGAATGCAA
4801 ATGATCCAGC TCCACCGAAT GCAAATGACC CCGCGCCGCC TAACGCGAAT GATCCGCCTC
4861 CGCCGAATCC AAACGACCCG GCACCTCCGC AGGGAAACAA CAACCCGCAG CCGCAACCGC
4921 GTCCGCAGCC ACAACCCCAG CCCCAACCGC AGCCGCAGCC GCAGCCACAA CCTCAGCCGC
4981 GTCCGCAGCC GCAGCCTCAG CCGGGAGGAA ACAACAACAA CAAGAATAAT AACAATGATG
5041 ACTCCTACAT TCCTAGCGCA GAAAAGATCC TGGAGTTTGT GAAGCAAATC CGCGACTCGA
5101 TCACTGAGGA GTGGAGCCAG TGTAACGTGA CGTGCGGTTC GGGCATCCGC GTGCGCAAGC
5161 GTAAAGGCTC GAATAAGAAG GCTGAGGACC TGACGCTGGA AGATATCGAC ACGGAAATTT
5221 GCAAGATGGA CAAATGCGAA TTCGTTCACG AGTACGACAT GATTACGTCG GACGGCCATC
5281 ACCTGTACGG TATCGAACCG TTGACCCTGC TGCTGGTGAC CGTTCTCACC GCACTGGGAG
```

```
     5341 CAGTTtaaGA ATTCtaaTCT AGACATAATC AGCCATACCA CATTTGTAGA GGTTTTACTT
     5401 GCTTTAAAAA ACCTCCCACA CCTCCCCCTG AACCTGAAAC ATAAAATGAA TGCAATTGTT
     5461 GTTGTTAACT TGTTTATTGC AGCTTATAAT GGTTACAAAT AAAGCAATAG CATCACAAAT
     5521 TTCACAAATA AAGCATTTTT CTTCACTGCA TTCTAGTTGT GGTTTGTCCA AACTCATCAA
     5581 TGTATCTcga gACAACTTTG TATAATAAAG TTGGTACCGG GCCCCCCGCT AGCGtcgaGT
     5641 ACCGGGCCCG GATCTAATTC AATTAGAGAC TAATTCAATT AGAGCTAATT CAATTAGGAT
     5701 CCAAGCTTAT CGATTTCGAA CCCTCGACCG CCGGAGTATA AATAGAGGCG CTTCGTCTAC
     5761 GGAGCGACAA TTCAATTCAA ACAAGCAAAG TGAACACGTC GCTAAGCGAA AGCTAAGCAA
     5821 ATAAACAAGC GCAGCTGAAC AAGCTAAACA ATCGGCTCGA GACCGGTCGC CACCATGGTG
     5881 AGCAAGGGCG AGGAGCTGTT CACCGGGGTG GTGCCCATCC TGGTCGAGCT GGACGGCGAC
     5941 GTAAACGGCC ACAAGTTCAG CGTGTCCGGC GAGGGCGAGG GCGATGCCAC CTACGGCAAG
     6001 CTGACCCTGA AGTTCATCTG CACCACCGGC AAGCTGCCCG TGCCCTGGCC CACCCTCGTG
     6061 ACCACCTTCG GCTACGGCCT GCAGTGCTTC GCCCGCTACC CCGACCACAT GAAGCAGCAC
     6121 GACTTCTTCA AGTCCGCCAT GCCCGAAGGC TACGTCCAGG AGCGCACCAT CTTCTTCAAG
     6181 GACGACGGCA ACTACAAGAC CCGCGCCGAG GTGAAGTTCG AGGGCGACAC CCTGGTGAAC
     6241 CGCATCGAGC TGAAGGGCAT CGACTTCAAG GAGGACGGCA ACATCCTGGG GCACAAGCTG
     6301 GAGTACAACT ACAACAGCCA CAACGTCTAT ATCATGGCCG ACAAGCAGAA GAACGGCATC
     6361 AAGGTGAACT TCAAGATCCG CCACAACATC GAGGACGGCA GCGTGCAGCT CGCCGACCAC
     6421 TACCAGCAGA ACACCCCCAT CGGCGACGGC CCCGTGCTGC TGCCCGACAA CCACTACCTG
     6481 AGCTACCAGT CCGCCCTGAG CAAAGACCCC AACGAGAAGC GCGATCACAT GGTCCTGCTG
     6541 GAGTTCGTGA CCGCCGCCGG GATCACTCTC GGCATGGACG AGCTGTACAA GTAAAGCGGC
     6601 CGCGACTCTA GATCATAATC AGCCATACCA CATTTGTAGA GGTTTTACTT GCTTTAAAAA
     6661 ACCTCCCACA CCTCCCCCTG AACCTGAAAC ATAAAATGAA TGCAATTGTT GTTGTTAACT
     6721 TGTTTATTGC AGCTTATAAT GGTTACAAAT AAAGCAATAG CATCACAAAT TTCACAAATA
     6781 AAGCATTTTT TTCACTGCAT TCTAGTTGTG GTTTGTCCAA ACTCATCAAT GTATCTTAGC
     6841 TAGAGCGGCC GCCACCGCGG TGGAGCTCGA GTACCCAGCT TTcttgtaca aagtggtttg
     6901 atCGGTACGT ACCCAATTCG CCCTATAGTG AGTCGTATTA CAATTCACTG GCCGTCGTTT
     6961 TACAACGTCG TGACTGGGAA AACCCTGGCG TTACCCAACT TAATCGCCTT GCAGCACATC
     7021 CCCCTTTCGC CAGCTGGCGT AATAGCGAAG AGGCCCGCAC CGATCGCCCT TCCCAACAGT
     7081 TGCGCAGCCT GAATGGCGAA TGGAAATTGT AAGCGTTAAT ATTTTGTTAA AATTCGCGTT
     7141 AAATTTTTGT TAAATCAGCT CATTTTTTAA CCAATAGGCC GAAATCGGCA AAATCCCTTA
     7201 TAAATCAAAA GAATAGACCG AGATAGGGTT GAGTGTTGTT CCAGTTTGGA ACAAGAGTCC
     7261 ACTATTAAAG AACGTGGACT CCAACGTCAA AGGGCGAAAA ACCGTCTATC AGGGCGATGG
     7321 CCCACTACGT GAACCATCAC CCTAATCAAG TTTTTTGGGG TCGAGGTGCC GTAAAGCACT
     7381 AAATCGGAAC CCTAAAGGGA GCCCCCGATT TAGAGCTTGA CGGGGAAAGC CGGCGAACGT
     7441 GGCGAGAAAG GAAGGGAAGA AAGCGAAAGG AGCGGGCGCT AGGGCGCTGG CAAGTGTAGC
     7501 GGTCACGCTG CGCGTAACCA CCACACCCGC CGCGCTTAAT GCGCCGCTAC AGGGCGCGTC
     7561 AG
//
```

pAttBRfB1RFP-Lp-OptCSPΔgpi:

```
LOCUS       pattB-LpP-OptCSP        7370 bp    DNA     circular
FEATURES             Location/Qualifiers
     misc_feature    complement(6729..6749)
                     /note="T7"
     misc_feature    complement(2216..2500)
                     /note="attB"
     misc_feature    2173..2192
                     /note="T3"
     misc_feature    2138..2156
                     /note="M13R"
     clone           2525..4220
                     /source="pENTR L1-lpProm-L4 4"
                     /type="Entry Clone"
     misc_feature    2516..2538
                     /note="attB1"
     misc_feature    4245..4319
                     /note="lipophorin signal peptide"
     misc_feature    4237..4244
                     /note="Vg Kozak"
     clone           4221..5422
                     /source="pENTR R4-optCSP-R3"
                     /type="Entry Clone"
     misc_feature    complement(4214..4233)
                     /note="attB4"
     misc_feature    5181..5410
                     /note="SV40 term"
     misc_feature    5473..5678
                     /note="3xP3-hsp70"
     misc_feature    5415..5436
                     /note="attB3"
     misc_feature    6390..6615
                     /note="SV40 term"
     misc_feature    complement(6684..6705)
                     /note="attB2"
```

```
     misc_feature    5697..6379
                     /note="DsRed"
     misc_feature    6621..6646
                     /note="insertion"

ORIGIN
        1 GTGGCACTTT TCGGGGAAAT GTGCGCGGAA CCCCTATTTG TTTATTTTTC TAAATACATT
       61 CAAATATGTA TCCGCTCATG AGACAATAAC CCTGATAAAT GCTTCAATAA TATTGAAAAA
      121 GGAAGAGTAT GAGTATTCAA CATTTCCGTG TCGCCCTTAT TCCCTTTTTT GCGGCATTTT
      181 GCCTTCCTGT TTTTGCTCAC CCAGAAACGC TGGTGAAAGT AAAAGATGCT GAAGATCAGT
      241 TGGGTGCACG AGTGGGTTAC ATCGAACTGG ATCTCAACAG CGGTAAGATC CTTGAGAGTT
      301 TTCGCCCCGA AGAACGTTTT CCAATGATGA GCACTTTTAA AGTTCTGCTA TGTGGCGCGG
      361 TATTATCCCG TATTGACGCC GGGCAAGAGC AACTCGGTCG CCGCATACAC TATTCTCAGA
      421 ATGACTTGGT TGAGTACTCA CCAGTCACAG AAAAGCATCT TACGGATGGC ATGACAGTAA
      481 GAGAATTATG CAGTGCTGCC ATAACCATGA GTGATAACAC TGCGGCCAAC TTACTTCTGA
      541 CAACGATCGG AGGACCGAAG GAGCTAACCG CTTTTTTGCA CAACATGGGG GNATCATGTA
      601 ACTCGCCTTN GATCGTTGGG AACCGGAGCT GAATGAAGCC ATACCAAACG ACGAGCGTGA
      661 CACCACGATG CCTGTAGCAA TGGCAACAAC GTTGCGCAAA CTATTAACTG GCGAACTACT
      721 TACTCTAGCT TCCCGGCAAC AATTAATAGA CTGGATGGAG GCGGATAAAG TTGCAGGACC
      781 ACTTCTGCGC TCGGCCCTTC CGGCTGGCTG GTTTATTGCT GATAAATCTG GAGCCGGTGA
      841 GCGTGGGTCT CGCGGTATCA TTGCAGCACT GGGGCCAGAT GGTAAGCCCT CCCGTATCGT
      901 AGTTATCTAC ACGACGGGGA GTCAGGCAAC TATGGATGAA CGAAATAGAC AGATCGCTGA
      961 GATAGGTGCC TCACTGATTA AGCATTGGTA ACTGTCAGAC CAAGTTTACT CATATATACT
     1021 TTAGATTGAT TTAAAACTTC ATTTTTAATT TAAAAGGATC TAGGTGAAGA TCCTTTTTGA
     1081 TAATCTCATG ACCAAAATCC CTTAACGTGA GTTTTCGTTC CACTGAGCGT CAGACCCCGT
     1141 AGAAAAGATC AAAGGATCTT CTTGAGATCC TTTTTTTCTG CGCGTAATCT GCTGCTTGCA
     1201 AACAAAAAAA CCACCGCTAC CAGCGGTGGT TTGTTTGCCG GATCAAGAGC TACCAACTCT
     1261 TTTTCCGAAG GTAACTGGCT TCAGCAGAGC GCAGATACCA AATACTGTCC TTCTAGTGTA
     1321 GCCGTAGTTA GGCCACCACT TCAAGAACTC TGTAGCACCG CCTACATACC TCGCTCTGCT
     1381 AATCCTGTTA CCAGTGGCTG CTGCCAGTGG CGATAAGTCG TGTCTTACCG GGTTGGACTC
     1441 AAGACGATAG TTACCGGATA AGGCGCAGCG GTCGGGCTGA ACGGGGGGTT CGTGCACACA
     1501 GCCCAGCTTG GAGCGAACGA CCTACACCGA ACTGAGATAC CTACAGCGTG AGCTATGAGA
     1561 AAGCGCCACG CTTCCCGAAG GGAGAAAGGC GGACAGGTAT CCGGTAAGCG GCAGGGTCGG
     1621 AACAGGAGAG CGCACGAGGG AGCTTCCAGG GGGAAACGCC TGGTATCTTT ATAGTCCTGT
     1681 CGGGTTTCGC CACCTCTGAC TTGAGCGTCG ATTTTTGTGA TGCTCGTCAG GGGGGCGGAG
     1741 CCTATGGAAA AACGCCAGCA ACGCGGCCTT TTTACGGTTC CTGGCCTTTT GCTGGCCTTT
     1801 TGCTCACATG TTCTTTCCTG CGTTATCCCC TGATTCTGTG GATAACCGTA TTACCGCCTT
     1861 TGAGTGAGCT GATACCGCTC GCCGCAGCCG AACGACCGAG CGCAGCGAGT CAGTGAGCGA
     1921 GGAAGCGGAA GAGCGCCCAA TACGCAAACC GCCTCTCCCC GCGCGTTGGC CGATTCATTA
     1981 ATGCAGCTGG CACGACAGGT TTCCCGACTG GAAAGCGGGC AGTGAGCGCA ACGCAATTAA
     2041 TGTGAGTTAG CTCACTCATT AGGCACCCCA GGCTTTACAC TTTATGCTTC CGGCTCGTAT
     2101 GTTGTGTGGA ATTGTGAGCG GATAACAATT TCACACAGGA AACAGCTATG ACCATGATTA
     2161 CGCCAAGCTC GAAATTAACC CTCACTAAAG GGAACAAAAG CTGGCTAGAA CTAGTGTCGA
     2221 CATGCCCGCC GTGACCGTCG AGAACCCGCT GACGCTGCCC CGCGTATCCG CACCCGCCGA
     2281 CGCCGTCGCA CGTCCCGTGC TCACCGTGAC CACCGCGCCC AGCGGTTTCG AGGGCGAGGG
     2341 CTTCCCGGTG CGCCGCGCGT TCGCCGGGAT CAACTACCGC CACCTCGACC CGTTCATCAT
     2401 GATGGACCAG ATGGGTGAGG TGGAGTACGC GCCCGGGGAG CCCAAGGGCA CGCCCTGGCA
     2461 CCCGCACCGC GGCTTCGAGA CCGTGACCTA CATCGTCGAC ACTAGTGgat catcaaacaa
     2521 gtttGTACAA AAAAGCAGGC TGGTACCGGG CCCCCCGCTA GCGTCGACGG TATCGATAAg
     2581 cTTGCGGGGA AGACACATTC GAGATACGCT AAGTGATTGA GCGATTACGA TCTAGCAAAA
     2641 CATACGTTCA GCTGTGAGAA TAATCATCCA TCTTCCTGCA ATGAGCAGTT CATTCCCGAT
     2701 TGAGGGATTT TATTCCCCGG GGGCCTTTTC AAACGGCTTA ATATAAGCAA TTAATAGTAT
     2761 TTTTTCTTTC AGGTTAGTTT ACTGTAATGG TGTAATTGTC ATCTTACACC TCCGTCTGAT
     2821 AAGAGATTAC GAAGCTCAGT ATGATGAAAT AAATAAGATA AATTTATTTA AAAAAGAACA
     2881 ATTGCTATGA GAGTGAAATA CAACAGTGGC GTTCACAATA TTCGAAAAAC AATAAAATTA
     2941 AAAAAAAAAC AAGAAAACAT TCACAAACAT ATCAATCTGC TTTCATCGAC ACCGAACTGC
     3001 TAGCCTCCCC AGTCTAACCG CGGTGGGGAC GTTTAATTGC CTTTGTTCTC GCACCCGGTC
     3061 AAACATACAC TTCGGACCTT GCTCCGAACC CCACTGTGAT CCCTAGCTCG TCATCATCAT
     3121 TGCCGGCATC ATGCTAAGCG TGCATTATTT TCACAACTTA GCGTAATGCT AGCGTGCGCT
     3181 AGCAACAAAC TCGCCCGCAG ACTCGTCACA GCACCGGTAC GATCGATCGT TTACCGTTCC
     3241 CTTTCCCGAT CGGGTTGGCT GCGATATCCG TGTCCGGGTA GAAAACTTCC CCTTTTACAC
     3301 ACACACACTC ACATACACAC AGAGCTGAAT AGCAACTTAC CTTATCTGTT CGTCATCGCT
     3361 CGGGCGGATC TGGACGAATC TTCGCACCGA TAACCATGTG GATCTACGAC CTCCGCTTGG
     3421 CTGTCTCTCT GCTCATGTGT ATGTCTGTGT GTGTGTGTAT GTGAGCTTCT TCCCTCAAAT
     3481 CCCTCGATCT CGCTGTGSCA ACAATCAAAC GTGCAAGTGC AAACATTGCA CCCCATTGAT
     3541 TATACACCCA ACACCAACCA ATTCCCCTTG CGGAGGCATC TCTGTGCTCG GCAGCATGTT
     3601 TACCGCAGAT CTACAGAGAA CTTCAATTGA GGTCCTTTCC ACCCCCAGCC CTCAACCGGC
     3661 AATCCGGCAG CCACTGGATC ATCAGCGAAA GAGAGAGAGA GCAGAGCAGA ACAGAGGTGA
     3721 CCAACTGTGG TATCGCTTCC CGCGCGCCGG TGTGTTGGTG TCCATTTCGG TGATCGCGAT
     3781 CCCGGCCGCT TCCAGCACCG TCCACCGATC AGTCACAAAA ACGCTCTCCA AACCCCTTAT
     3841 CAGCACCGTT CGCTGATGTG AACCCCCGTT CAAACCCCAA ATGCAGTGTT TGTATTGCTG
     3901 TGTGTATGTG TACGTGCGTG TGTGTGGAAA TTCTATAAAA GTAGGCACCC GTGGCCGGGA
     3961 TCCGTTATTC CCGTCCTGAG GCCCGCCCGG GATCGCTGGT GACGACAGAC GAGCCGCTGT
     4021 GTGACGTACG TAGTGCCCGA TCGGTAAAGA GTGAACCGTC TTCTCTGCAG TGTAGGAGAG
     4081 AACGGTTTCA TCTTTTTCGC CCACACCCCC CCGGTTACAT TCCATGTTGA ACTGTAAGGT
     4141 CTAGTGAACA TTTCCTGAGT GTGGAAAGTG TGGTTTAGTG CGTGAGAGTG CACGGACACG
     4201 ACACTCGAGC ACCCAACTTT TCTATACAAA GTTggtACCG AACCATGTGG GTGCTGGGTG
     4261 GCCGTCGGCT CCTGTGGAGC TTCCTGGTGA GCCTGGTGTT GATCCAAAGC GTGTCCGCCA
     4321 AGATCATCCA GGCGCAGCGC AACTTGAATG AGCTGTGTTA TAATGAAGGA AACGATAACA
     4381 AATTGTACCA TGTGTTGAAC AGCAAGAATG GTAAGATCTA TAATAGAAAC ACGGTCAACC
     4441 GACTCTTGGC TGATGCTCCG GAAGGAAAGA AAAACGAGAA GAAAAATGAG AAGATCGAGC
```

```
     4501 GGAATAACAA ATTGAAGCAG CCTCCTCCGC CGCCCAACCC CAACGATCCA CCTCCACCAA
     4561 ACCCAAACGA CCCGCCACCT CCCAATCCAA ATGATCCGCC GCCGCCGAAC CCGAACGACC
     4621 CTGCGCCTCC TAACGCAAAC GATCCGGCCC CACCGAATGC CAACGATCCA GCCCCGCCGA
     4681 ACGCGAACGA CCCTGCACCG CCTAATGCCA ACGATCCGGC TCCGCCGAAT GCAAATGATC
     4741 CAGCTCCACC GAATGCAAAT GACCCCGCGC CGCCTAACGC GAATGATCCG CCTCCGCCGA
     4801 ATCCAAACGA CCCGGCACCT CCGCAGGGAA ACAACAACCC GCAGCCGCAA CCGCGTCCGC
     4861 AGCCACAACC CCAGCCCCAA CCGCAGCCGC AGCCGCAGCC ACAACCTCAG CCGCGTCCGC
     4921 AGCCGCAGCC TCAGCCGGGA GGAAACAACA ACAACAAGAA TAATAACAAT GATGACTCCT
     4981 ACATTCCTAG CGCAGAAAAG ATCCTGGAGT TTGTGAAGCA AATCCGCGAC TCGATCACTG
     5041 AGGAGTGGAG CCAGTGTAAC GTGACGTGCG GTTCGGGCAT CCGCGTGCGC AAGCGTAAAG
     5101 GCTCGAATAA GAAGGCTGAG GACCTGACGC TGGAAGATAT CGACACGGAA ATTTGCAAGA
     5161 TGGACAAATG CGAATTCtaa TCTAGACATA ATCAGCCATA CCACATTTGT AGAGGTTTTA
     5221 CTTGCTTTAA AAAACCTCCC ACACCTCCCC CTGAACCTGA AACATAAAAT GAATGCAATT
     5281 GTTGTTGTTA ACTTGTTTAT TGCAGCTTAT AATGGTTACA AATAAAGCAA TAGCATCACA
     5341 AATTTCACAA ATAAAGCATT TTTCTTCACT GCATTCTAGT TGTGGTTTGT CCAAACTCAT
     5401 CAATGTATCT cgagACAACT TTGTATAATA AAGTTGGTAC CGGGCCCCCC GCTAGCGtcg
     5461 aGTACCGGGC CCGGATCTAA TTCAATTAGA GACTAATTCA ATTAGAGCTA ATTCAATTAG
     5521 GATCCAAGCT TATCGATTTC GAACCCTCGA CCGCCGGAGT ATAAATAGAG GCGCTTCGTC
     5581 TACGGAGCGA CAATTCAATT CAAACAAGCA AAGTGAACAC GTCGCTAAGC GAAAGCTAAG
     5641 CAAATAAACA AGCGCAGCTG AACAAGCTAA ACAATCGGCT CGAGACCGGT CGCCACCATG
     5701 GTGCGCTCCT CCAAGAACGT CATCAAGGAG TTCATGCGCT TCAAGGTGCG CATGGAGGGC
     5761 ACCGTGAACG GCCACGAGTT CGAGATCGAG GGCGAGGGCG AGGGCCGCCC CTACGAGGGC
     5821 CACAACACCG TGAAGCTGAA GGTGACCAAG GGCGGCCCCC TGCCCTTCGC CTGGGACATC
     5881 CTGTCCCCCC AGTTCCAGTA CGGCTCCAAG GTGTACGTGA AGCACCCCGC CGACATCCCC
     5941 GACTACAAGA AGCTGTCCTT CCCCGAGGGC TTCAAGTGGG AGCGCGTGAT GAACTTCGAG
     6001 GACGGCGGCG TGGTGACCGT GACCCAGGAC TCCTCCCTGC AGGACGGCTG CTTCATCTAC
     6061 AAGGTGAAGT TCATCGGCGT GAACTTCCCC TCCGACGGCC CCGTAATGCA GAAGAAGACC
     6121 ATGGGCTGGG AGGCCTCCAC CGAGCGCCTG TACCCCCGCG ACGGCGTGCT GAAGGGCGAG
     6181 ATCCACAAGG CCCTGAAGCT GAAGGACGGC GGCCACTACC TGGTGGAGTT CAAGTCCATC
     6241 TACATGGCCA AGAAGCCCGT GCAGCTGCCC GGCTACTACT ACGTGGACTC CAAGCTGGAC
     6301 ATCACCTCCC ACAACGAGGA CTACACCATC GTGGAgCAGT ACGAGCGCAC CGAGGGCCGC
     6361 CACCACCTGT TCCTGTAGCG GCCGCGACTC TAGATCATAA TCAGCCATAC CACATTTGTA
     6421 GAGGTTTTAC TTGCTTTAAA AAACCTCCCA CACCTCCCCC TGAACCTGAA ACATAAAATG
     6481 AATGCAATTG TTGTTGTTAA CTTGTTTATT GCAGCTTATA ATGGTTACAA ATAAAGCAAT
     6541 AGCATCACAA ATTTCACAAA TAAAGCATTT TTTTCACTGC ATTCTAGTTG TGGTTTGTCC
     6601 AAACTCATCA ATGTATCTTA AAGCTTATCG ATACGCGTAC GGCGCGcCTA GAGCGGCCGC
     6661 CACCGCGGTG GAGCTCGAGT ACCCAGCTTT cttgtacaaa gtggtttgat CGGTACGTAC
     6721 CCAATTCGCC CTATAGTGAG TCGTATTACA ATTCACTGGC CGTCGTTTTA CAACGTCGTG
     6781 ACTGGGAAAA CCCTGGCGTT ACCCAACTTA ATCGCCTTGC AGCACATCCC CCTTTCGCCA
     6841 GCTGGCGTAA TAGCGAAGAG GCCCGCACCG ATCGCCCTTC CCAACAGTTG CGCAGCCTGA
     6901 ATGGCGAATG GAAATTGTAA GCGTTAATAT TTTGTTAAAA TTCGCGTTAA ATTTTTGTTA
     6961 AATCAGCTCA TTTTTTAACC AATAGGCCGA AATCGGCAAA ATCCCTTATA AATCAAAAGA
     7021 ATAGACCGAG ATAGGGTTGA GTGTTGTTCC AGTTTGGAAC AAGAGTCCAC TATTAAAGAA
     7081 CGTGGACTCC AACGTCAAAG GGCGAAAAAC CGTCTATCAG GGCGATGGCC CACTACGTGA
     7141 ACCATCACCC TAATCAAGTT TTTTGGGGTC GAGGTGCCGT AAAGCACTAA ATCGGAACCC
     7201 TAAAGGGAGC CCCCGATTTA GAGCTTGACG GGGAAAGCCG GCGAACGTGG CGAGAAAGGA
     7261 AGGGAAGAAA GCGAAAGGAG CGGGCGCTAG GGCGCTGGCA AGTGTAGCGG TCACGCTGCG
     7321 CGTAACCACC ACACCCGCCG CGCTTAATGC GCCGCTACAG GGCGCGTCAG
//
```
