# Supplementary material for: Tools for Anopheles gambiae Transgenesis
Source: G3 (Bethesda). 2015 Apr 13;5(6):1151–63. doi: 10.1534/g3.115.016808 (PMC4478545; doi:10.1534/g3.115.016808)
Supplement: Supporting Information [file supp_g3.115.016808_TableS1.pdf]

Table S1 Comparison of transgenesis efficiency using two promoters controlling *piggyBac* transposase expression. The data summarize many transgenesis experiments in which *piggyBac* constructs were micro-injected into *A. gambiae* embryos together with helper plasmid expressing transposase under control of either the Drosophila *hsp70* or the *A. gambiae vasa* (*Vas2*) promoter. Using the *Vas2* promoter, 3.4 x more transiently expressing survivor larvae gave transgenic progeny and 15 x more transgenic larvae could be recovered.

Transposase promoter in helper plasmid	*hsp70*	*Vas2*
Number of injected eggs	1421	950
Surviving larvae (%)	155 (10.9%)	137 (14.4%)
Transiently expressing larvae (%)	71 (45.8%)	79 (57.6%)
% transiently expressing larvae that gave rise to transgenics	5.6%	18.9%
Average number of transgenics per transient	1.75	27.1
